# Copper-Catalyzed Enantioselective Synthesis of Chiral Selenium-Based Versatile Synthons

**DOI:** 10.1021/acscentsci.6c00281

**Published:** 2026-04-29

**Authors:** Qingqin Huang, Jin-Xun Chen, Yu-Ping Tang, Tian-Hao Shui, Jia-Xin Yi, Chao-Gang Zhang, Lei Dai

**Affiliations:** † Chongqing Key Laboratory of Natural Product Synthesis and Drug Research, School of Pharmaceutical Sciences, 47913 Chongqing University, Chongqing 401331, China

## Abstract

Enantioenriched organoselenium compounds have found diverse applications across organic synthesis, asymmetric catalysis, and medicinal chemistry, driving a growing demand for efficient catalytic approaches to access novel chiral organoselenium molecules. However, in contrast to their oxygen- and sulfur-based counterparts, catalytic asymmetric routes to selenium-based chiral compounds remain underdeveloped, and no catalytic method has been reported for preparing chiral selenium-based molecules capable of serving multiple functional roles. Here, we describe a mild and simple Cu­(II)/diamine catalytic system that delivers chiral selenium-based versatile synthons with broad downstream utility, including applications as valuable synthetic intermediates, chiral organocatalysts, and bioactive agents. Computational studies explored the possibility of enantioselectivity-determining factors and a selenium-coordinating tetradentate copper catalyst model. A broad substrate scope was performed that supported this mechanistic proposal. We anticipate that this work will furnish a robust selenium-based synthetic toolbox and expand the accessible chemical space in chiral organoselenium chemistry.

## Graphical abstract

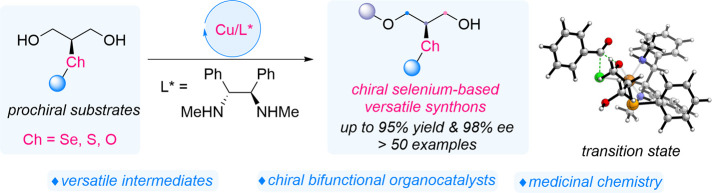

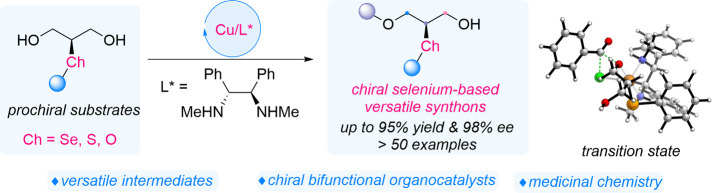

## Introduction

Selenium is an essential trace element for human health,^1,2^ yet its reactivity and behavior within organic molecules remain far less explored compared to its chalcogen counterparts, oxygen and sulfur. The development of organoselenium chemistry did not gain substantial scientific momentum until the seminal contributions of Klayman and Günther in the 1970s.^3^ More recently, chiral organoselenium compounds have emerged as highly versatile functional molecules with broad applications across biochemistry,^4–7^ medicinal chemistry,^8–11^ and synthetic chemistry^12–14^ (Figure 1a). Within biological systems, for example, selenocysteine, as the 21st proteinogenic amino acid, constitutes a key component of numerous selenoproteins.^15–17^ In medicinal chemistry, chiral organoselenium motifs have demonstrated diverse bioactivities including antibacterial, anti-inflammatory, and antitumor properties.^18^ In catalysis sciences,^19,20^ organoselenides can function as chiral ligands^21,22^ for transition-metal catalysts and as privileged scaffolds in organocatalysis.^23–29^ They also serve as valuable intermediates and building blocks in organic synthesis,^30,31^ owing to selenium’s unique redox properties and its more facile removability relative to other chalcogens. These broad and expanding applications underscore a pressing need for efficient methods to access structurally diverse chiral organoselenium compounds, which poses an outstanding challenge in modern synthetic chemistry.

**Figure 1:**
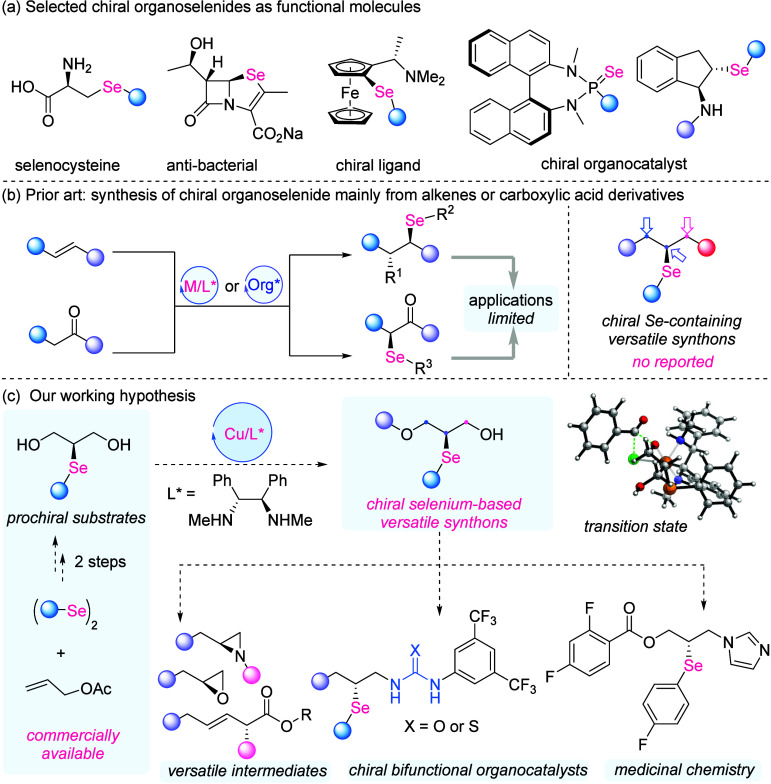
Background and our working hypothesis. (a) Selected chiral organoselenides as functional molecules. (b) Prior art: synthesis of chiral organoselenide mainly from alkenes or carboxylic acid derivatives. (c) Our working hypothesis.

In contrast to the extensive advances achieved in the asymmetric synthesis of oxygen- and sulfur-containing chiral molecules,^32–45^ the catalytic enantioselective construction of chiral organoselenides remains comparatively underdeveloped (Figure 1b). The reported elegant examples mainly involved catalytic asymmetric reactions between an organoselenium reagent and an alkene or a carbonyl derivative, such as additions to alkenes^46–54^ and selenenylation of carbonyl derivatives.^55–58^ Most existing methods focus on construction of specific molecular architectures containing a chiral organoselenide moiety while overlooking their downstream transformations and broader synthetic applications as functional molecules; some of these methods produce chiral organoselenides that only served as a single type of functional molecule. However, a catalytic asymmetric strategy to access chiral organoselenium synthons with multiple potential derivatizable sites, one amenable to diverse derivatizations and capable of accessing multiple types of functional molecules, has not been reported. Consequently, the full synthetic potential of selenium-based chiral molecules remains largely untapped. Motivated by this long-standing gap, we aimed to develop a conceptually simple yet robust catalytic system that could be readily scaled and broadly applicable. Here, we describe an asymmetric synthesis of chiral selenium-based versatile synthons through a mild and operationally simple Cu­(II)/diamine catalytic system, in which the selenium atom in the substrate potentially coordinated to the copper center (Figure 1c). The prochiral selenium-containing diols are prepared on multigram scale from commercially available diselenides and allylic acetates, and the resulting enantioenriched organoselenides exhibit broad functional relevance as valuable synthetic intermediate, potential chiral organocatalyst, and bioactive agent. This method is anticipated to expand the accessible chemical space of chiral organoselenium chemistry and establish a valuable synthetic platform for both medicinal and synthetic chemistry.

## Results and Discussion

At the outset of this study, our objective was to establish a simple yet robust catalytic synthetic method that would enable the scalable preparation of enantioenriched selenium-based versatile synthons from readily accessible starting materials. Notably, allylic acetate is commercially available at modest cost, and diphenyl diselenide can be either purchased directly or obtained in a single synthetic step. From these inexpensive inputs, the prochiral organoselenium diol **1a** was readily obtained in two steps and 75% overall yield (Figure 2a). We next sought an efficient catalytic asymmetric protocol to transform **1a**. After a series of reaction screenings, Cu-catalyzed O–H bond functionalization was identified to be a mild and operationally simple method suitable for accessing the chiral organoselenium products. Under the optimized conditions using chiral ligand **L1** (see Table S1 in the Supporting Information for optimization details), the resulting chiral product **3a** could be obtained in 83% yield and with 96% ee, and the reaction scaled smoothly to 5 mmol without erosion of efficiency. Oxazoline-based ligands were widely applied in copper-catalyzed asymmetric O–H functionalization, and standard commonly utilized BOX ligands **L2**–**L6** only gave moderate enantioselectivities under the standard conditions. Employment of PyBOX ligand **L7** and PHOX ligand **L8** yielded the product in <25% ee values. 1,8-Naphthyridine-based ligand **L9** was also evaluated and only racemic product was obtained. With the chiral organoselenide product in hand, its synthetic applications in accessing multiple functional molecules were carefully investigated. Given the presence of several potential reactive sites, we first demonstrated its derivatization as a versatile organic intermediate through several representative transformations (Figure 2b). Sequential protection–deprotection furnished the silyl-protected organoselenium intermediate **4**, which underwent smooth transformation in the presence of *m*-CPBA to deliver the masked chiral oxirane **5**. Notably, **5** serves as a key intermediate in the synthesis of several naturally occurring bioactive molecules, including (*R*)-Goniothalamin and (*R*)-Argentilactone.^59^ From the same intermediate **4**, synthetically valuable aziridine **7** was obtained by leveraging the distinctive removability of the selenium substituent. Such chiral aziridine have been employed in the synthesis of potent antiviral agents, exemplified by the hepatitis C virus NS5B polymerase inhibitor MK-3281.^60^ Beyond the formation of chiral oxirane and aziridine, chiral organoselenide **3a** also underwent smooth conversion to allyl selenide **9**, a privileged precursor for [2,3]-sigmatropic rearrangements. The resulting chiral allyl alcohol **10** and allyl chloride **11** constitute synthetically useful intermediates suitable for diverse downstream derivatizations. Considering the active studies on organoselenides as chiral organocatalysts, two potential selenium-based chiral organocatalysts were also derived from **3a** (Figure 2c). Enantioenriched amine **12** was readily obtained via a Mitsunobu reaction followed by deprotection, and subsequent coupling with isothiocyanate or thiocyanate furnished the corresponding bifunctional organocatalysts **13** and **14**, respectively. Collectively, these transformations underscore the potential of **3a** to function as an enantioenriched selenium-based versatile synthon, enabling access to several synthetically useful and biologically relevant architectures.

**Figure 2:**
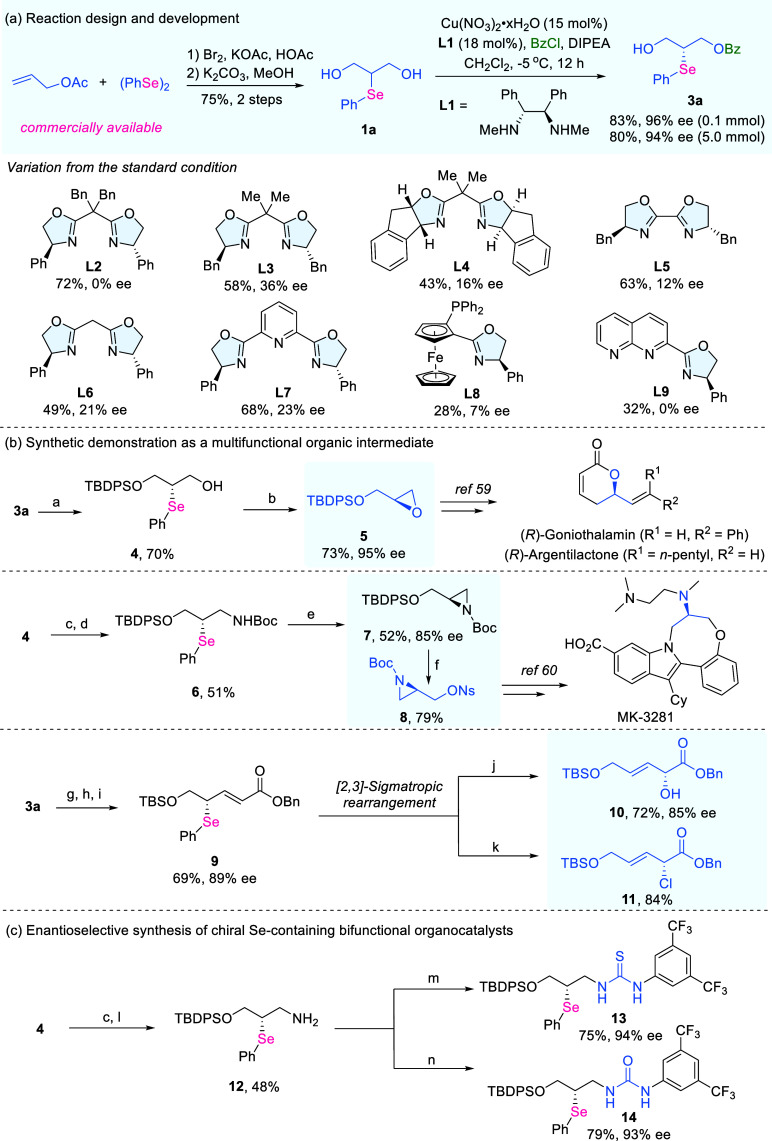
Reaction development and synthetic demonstration as functional molecules for organic synthesis. (a) Reaction design and development. (b) Synthetic demonstration as a multifunctional organic intermediate. (c) Enantioselective synthesis of chiral Se-containing bifunctional organocatalysts. a. **3a** (1.0 equiv), TBDPSCl (1.5 equiv), imidazole (1.5 equiv), DMF (0.2 M); then K_2_CO_3_ (0.3 equiv), MeOH (0.2 M). b. **4** (1.0 equiv), *m*-CPBA (5.0 equiv), 10% KOH aq. (0.5 M), IPA (0.05 M). c. **4** (1.0 equiv), phthalimide (1.5 equiv), PPh_3_ (1.5 equiv), DEAD (1.5 equiv), THF (0.05 M). d. N_2_H_4_·H_2_O (5 equiv), MeOH (0.1 M); then TEA (1.5 equiv), (Boc)_2_O (1.5 equiv), DMAP (0.1 equiv), DCM (0.1 M). e. **6** (1.0 equiv) and *m*-CPBA (5.0 equiv) in THF at −60 °C for 1 h; then add *t*-BuOK (3.0 equiv) at 0 °C. f. **7** (1.0 equiv), TBAF (2.0 equiv), THF (0.1 M), 0 °C and then NsCl (1.5 equiv), TEA (1.5 equiv), DMAP (0.1 equiv), DCM (0.1 M). g. **3a** (1.0 equiv), TBSCl (1.5 equiv), imidazole (1.5 equiv), DMF (0.2 M), then K_2_CO_3_ (0.3 equiv), MeOH (0.2 M). h. DMP (2.0 equiv), DCM (0.1 M), 0 °C. i. Ph_3_P=COOBn (1.2 equiv), DCM (0.1 M). j. **9** (1.0 equiv), pyridine (0.3 M), THF (0.1 M), H_2_O_2_ (2.0 equiv), −20 °C. k. **9** (1.0 equiv), ethyl vinyl ether (12.0 equiv), sulfuryl chloride (2.0 equiv), hexane (0.1 M). l. N_2_H_4_·H_2_O (5 equiv), MeOH (0.1 M). m. **12** (1.0 equiv), 3,5-di­(trifluoromethyl)­phenyl isothiocyanate (1.2 equiv), THF (0.1 M), 0 °C to rt. n. **12** (1.0 equiv), 3,5-di­(trifluoromethyl)­phenyl isocyanate (1.2 equiv), THF (0.1 M), 0 °C to rt.

While the chemical properties of organoselenides are captivating, studies on selenium as potential bioactive agents are also highly sought after. It has been well supported that daily Se supplementation could benefit human health^4^ through its antioxidant, anti-inflammatory and antitumoral properties. In medicinal chemistry, incorporation of Se moieties into a bioactive molecule is a promising strategy to acquire new therapeutic agents. For instance, a Se-containing imidazole derivative **15** exhibits promising performance for the treatment of fungal infections as a potent CYP51 inhibitor^61,62^ (Figure 3a). The enantioenriched structural analogues were subjected to molecular docking studies with CYP51 protein, and (*S*)-**16** showed a higher binding affinity with the target protein. The high binding affinity may be attributed to multiple intermolecular interactions, including hydrogen bonding with TYR132 residue, hydrophobic interaction with PRO230, LEU376 and THR311 residues, chalcogen bonding with TYR505 residue, and the coordination of fluoro on the phenylselanyl moiety to Fe^3+^ ion in the target protein. A concise synthesis of (*S*)-**16** was then investigated, starting from our synthesized Se-based chiral synthon **3aj** (Figure 3b). The silyl-masked alcohol intermediate **17** was smoothly afforded, which was then applied to the Mitsunobu reaction to deliver phthalimide derivative **18**. The hydrazinolysis and imidazole synthesis formed intermediate **19**, and (*S*)-**16** was readily prepared via the deprotection and benzoylation reaction of **20**, without any loss of enantioenrichment throughout the synthetic process. This concise synthetic route further highlights the practical utility of our Se-based versatile synthons and may inspire the rational design and development of selenium-containing therapeutic agents.

**Figure 3:**
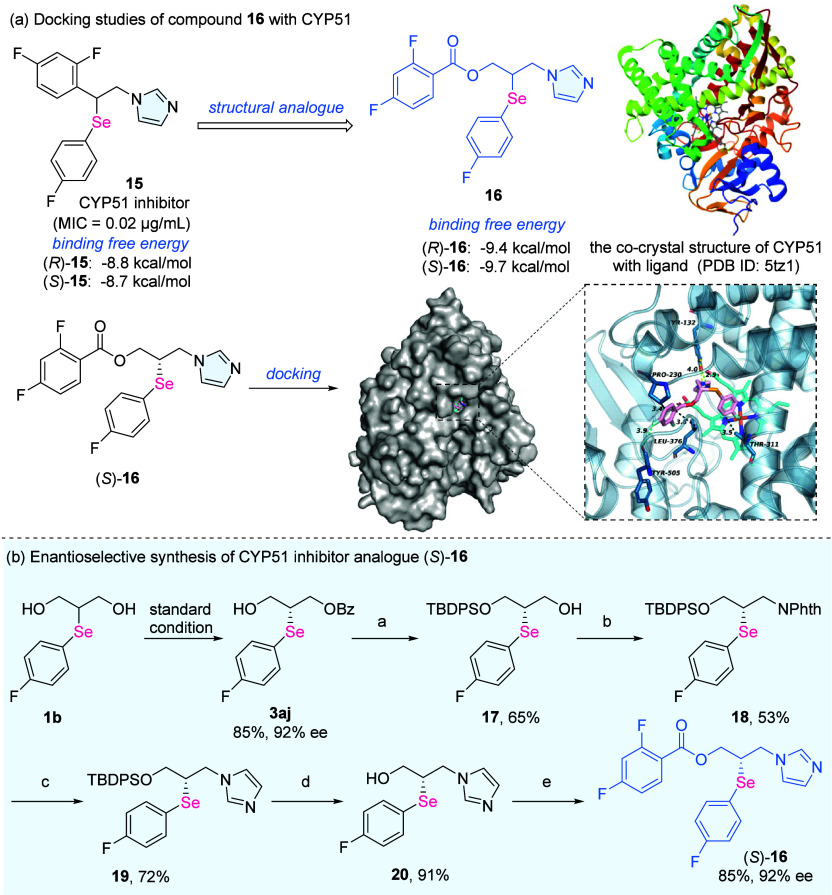
Synthetic demonstration as a potential functional molecule in medicinal chemistry. (a) Docking studies of compound **16** with CYP51. (b) Enantioselective synthesis of the CYP51 inhibitor analogue (*S*)-**16**. Standard condition: diol (0.1 mmol, 1.0 equiv), *p*-fluorobenzoyl chloride (0.12 mmol. 1.2 equiv), Cu­(NO_3_)_2_·*x*H_2_O (0.015 mmol, 0.15 equiv), (*R*,*R*)-**L1** (0.018 mmol, 0.18 equiv) and DIPEA (0.12 mmol, 1.2 equiv) in CH_2_Cl_2_ (1.0 mL) at −5 °C. a. **3aj** (1.0 equiv), TBDPSCl (1.5 equiv) and imidazole (1.5 equiv) in DMF (0.2 M); then K_2_CO_3_ (0.3 equiv), MeOH (0.2 M). b. **17** (1.0 equiv), phthalimide (1.5 equiv), PPh_3_ (1.5 equiv), and DEAD (1.5 equiv) in THF (0.05 M) at 0 °C. c. **18** (1.0 equiv), N_2_H_4_·H_2_O (5 equiv) in MeOH (0.1 M); then HCHO (3.0 equiv), glyoxal (3.0 equiv) and NH_4_Cl (3.0 equiv) in MeOH (0.1 M) under reflux. d. **19** (1.0 equiv) and TBAF (2.0 equiv) in THF (0.1 M) at 0 °C. e. **20** (1.0 equiv), 2,4-difluorobenzoyl chloride (1.2 equiv), TEA (1.5 equiv), and DMAP (0.1 equiv) in DCM (0.05 M).

As incorporation of the Se motif imparts our synthesized synthon with multiple functional potentials, we became interested in uncovering the mechanistic role of selenium during the catalytic process. Copper-catalyzed asymmetric O–H functionalization is a widely applicable strategy for accessing enantioenriched molecules, typically employing oxazoline-based ligands as the chiral source.^63–71^ In the present work, 1,2-diphenyl-1,2-ethylenediamine (DPEN) was used in this transformation for the first time, and it is generally inexpensive, structurally flexible, and well suited for large-scale industrial production. As indicated by preliminary studies, no obvious kinetic resolution was observed for the catalytic reaction (see Table S2 in the Supporting Information), and the mononuclear copper catalyst may be an active catalytic species (see Table S3 in the Supporting Information). To investigate the interaction between the selenium moiety and Cu­(II)/DPEN catalyst, DFT calculations were conducted. The deprotonation step was barrierless (see the Supporting Information, Figures S1–S4), indicating that the enantioselectivity was mostly determined by the acyl transfer process. Through transition state conformational analysis for the acyl transfer step, we identified the optimal transition states for the formation of (*R*)-**3a** and (*S*)-**3a** (Figure 4a). The results showed that the *R*-configured transition state was more favorable. Distortion-interaction analysis for unimolecular reactions^72^ was then performed by fragmenting the transition states into benzoyl chloride and the tetradentate copper complex to examine structural changes along the intrinsic reaction coordinate (IRC)^73,74^ path during acylation process (Figure 4b). It indicated that steric effects were the dominant factor, while the interaction differences between the two transition states were minor (Supporting Information, Table S4). Subsequently, we analyzed the steric distribution of the ligand **L1** using a steric map^75^ (Figure 4c). Although **L1** exhibited moderate overall steric bulkiness (%V_Bur = 36.8%) with relatively greater hindrance in the second and fourth quadrants, the key distinction lay in the position of the hydroxy group. In **TS2-** ***S***, this group was situated closer to the ligand; albeit with weak interactions present in the hydroxy group, they were insufficient to compensate for the extra steric repulsion. This seemingly subtle positional difference forced the entire system into a more strained configuration, thereby increasing the steric hindrance between benzoyl chloride and the tetradentate copper complex in **TS2-** ***S***. Studies on weak interactions^76^ indicated no significant difference between the two transition states (see the Supporting Information, Figure S9). The key difference was that the phenylselanyl group in **TS1-** ***R*** engaged in C–H···π and vdW interactions, whereas in **TS2-** ***S*** the ligand formed hydrogen bonding and vdW interactions with the hydroxy group (Figure 4d). Subsequently, a more detailed analysis of the interaction energy via energy decomposition analysis (EDA)^77^ was performed (Figure 4e). By dividing the transition state into different components (see the Supporting Information, Table S5–S7), we found that the electrostatic interaction between **1a** and the other parts of the transition state may also contribute to the enantioselectivity, and electrostatic potential-colored molecular penetration maps for these two parts were presented (Figure 4f). It was observed that a larger molecular penetration volume was present in **TS1-** ***R***, and therefore stronger electrostatic interactions. This conclusion was further supported by Hirshfeld charge calculations^78^ (see the Supporting Information, Figure S11). In the favored transition state, the charge on O is −0.26 and on Cu is +0.44, whereas in the disfavored transition state, the charge on O is −0.23 and on Cu is +0.43. Less charge difference in the disfavored transition state could be related to weakened electrostatic interactions. We proposed that this may be due to the hydrogen bonding of the hydroxy group in **TS2-** ***S***, which could reduce the nucleophilic nature of the oxyanion during the acyl transfer process (see the Supporting Information, Figure S11), and this may also account for the higher transition state energy. In summary, distortion–interaction analysis indicates that steric effects are the origin of enantioselectivity, with the interaction energy being slightly unfavorable for the favored transition state. However, a more in-depth EDA analysis using alternative fragmenting methods reveals that electrostatic interactions, components of the interaction energy, may also play a role in governing the enantioselectivity. The computational studies indicate that the selenium atom may participate in the coordination to the copper center and potentially influences the enantioselectivity via weak interactions (such as chalcogen bond and chalcogen-bonding-assisted hydrogen bond in Figure S9), and the following generality studies also support this proposed catalyst model (Figure 5c). In the long run, these findings could provide guidance in the design of catalytic asymmetric synthesis of more novel selenium-based versatile synthons.

**Figure 4:**
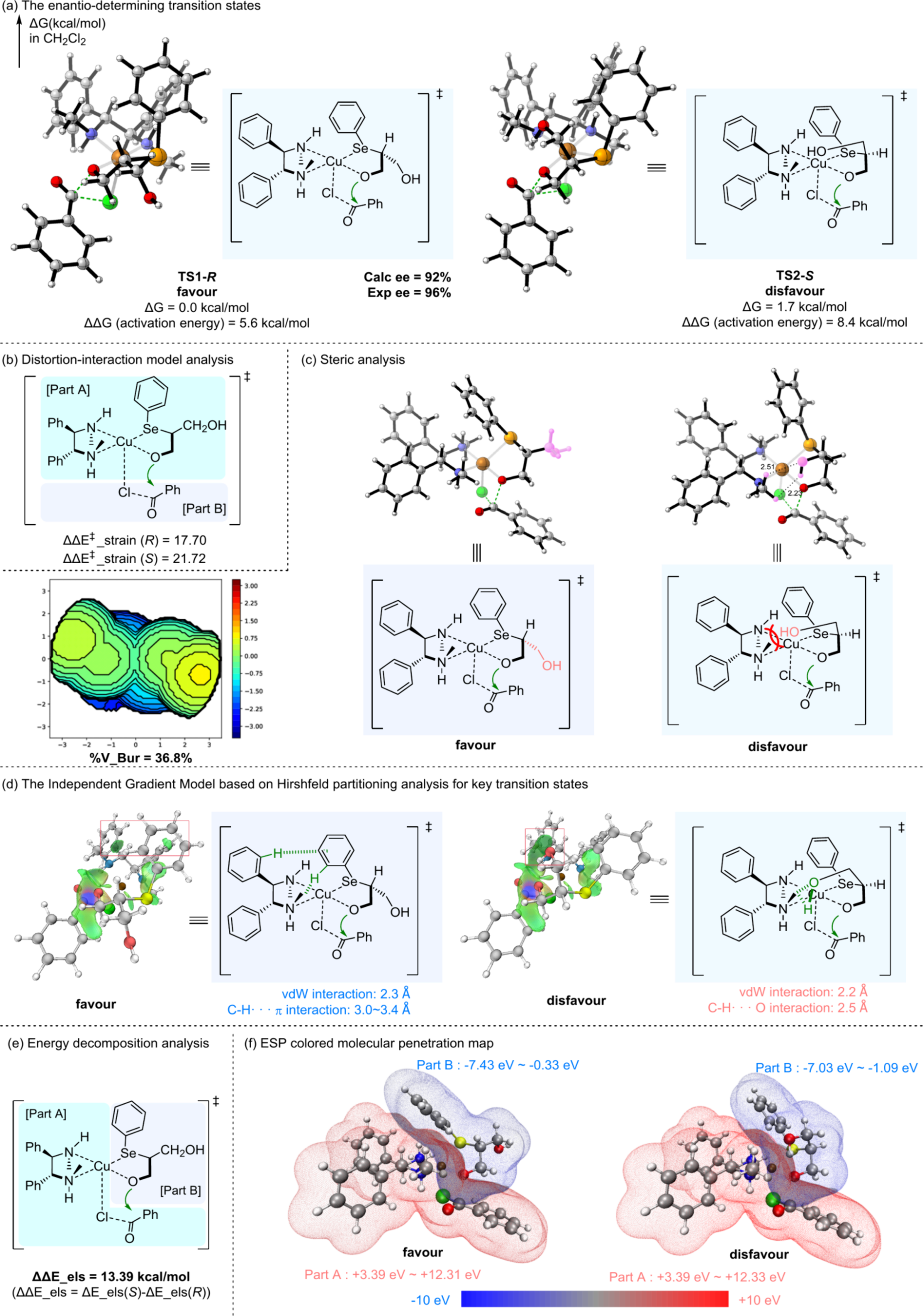
Computational studies of the catalytic system. Computed at B3LYP-D3­(BJ)/def2-TZVPP­(SDD for Cu and Se)/SMD­(CH_2_Cl_2_)//B3LYP-D3­(BJ)/def2-SVP­(SDD for Cu and Se). (a) The enantio-determining transition states. (b) Distortion-interaction model analysis. (c) Steric analysis. (d) The Independent Gradient Model based on Hirshfeld partitioning analysis for key transition states. (e) Energy decomposition analysis. (f) ESP colored molecular penetration map.

**Figure 5:**
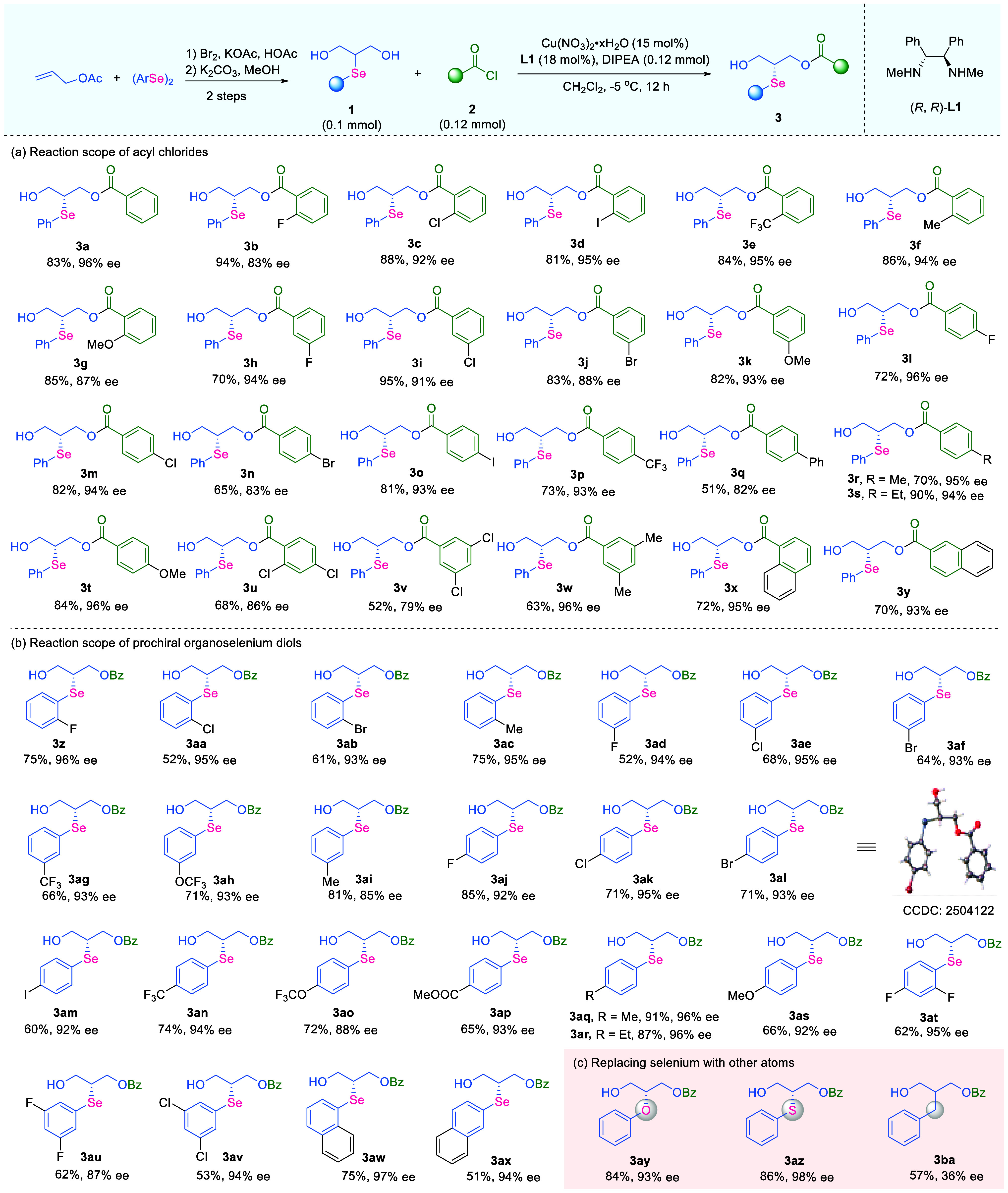
Substrate scope for the synthesis of chiral selenium-based synthons. (a) Reaction scope of acyl chlorides. (b) Reaction scope of prochiral organoselenium diols. Reaction conditions: **1** (0.1 mmol, 1.0 equiv), **2** (0.12 mmol. 1.2 equiv), Cu­(NO_3_)_2_·*x*H_2_O (0.015 mmol, 0.15 equiv), (*R*,*R*)-**L1** (0.018 mmol, 0.18 equiv) and DIPEA (0.12 mmol, 1.2 equiv) in CH_2_Cl_2_ (1.0 mL) at −5 °C; isolated yields reported.

To meet the diverse structural requirements of selenium-based synthons for various practical applications, we carefully evaluated the substrate generality of this transformation (Figure 5). A series of selenium-containing diol substrates **1** could be readily obtained in two steps from commercially available allylic acetate and diaryl diselenides. The scope was then investigated by varying the acyl chloride component (Figure 5a). A wide array of benzoyl chlorides bearing a monosubstituted aryl group was well accommodated, delivering the corresponding products in reliably high yields and with great enantioselectivities. Steric effects, electronic properties, and substitution patterns on the phenyl ring had minimal impact on the reaction outcomes (**3a**–**3t**). Among them, a range of chiral selenium-containing products bearing halides or strong electron-withdrawing groups were all obtained in good yields and with high ee values (**3b**–**3e**, **3h**–**3j**, and **3l**–**3p**). Benzoyl chlorides with an electron-donating phenyl ring were proved equally compatible substrates for the synthesis of chiral products (**3g**, **3k**, **3t**). It was worth noting that disubstituted benzoyl chlorides and naphthoyl chlorides underwent smooth Cu-catalyzed O–H bond benzoylation to afford the corresponding chiral organoselenium products in good yields and with decent enantioselectivities (**3u**–**3y**). Next, the synthetic efforts were directed toward the evaluation of organoselenium diols (Figure 5b). A structurally diverse collection of Se-containing diols bearing mono- or disubstituted aryl groups was examined, an essential variation for the rapid construction of an enantioenriched selenium-based molecular library. In most cases, the desired products were isolated in consistently high yields and with excellent enantioselectivities (**3z**–**3ax**), indicating that the method was largely insensitive to electronic or steric variations in the arylselanyl unit. The absolute configuration of the chiral Se-based molecules was unambiguously confirmed by the X-ray crystallographic analysis of **3al**. The relatively lower yields in some cases are attributed to the low conversion of diols, and extending the reaction time is a potential solution. The reporting catalytic reaction also displayed great chemoselectivity: substrates containing labile functional groups, such as iodides or esters, were well tolerated and delivered the corresponding products without significantly compromising yield or ee values (**3am**, **3ap**). Additionally, diol substrates bearing a phenylselanyl motif with a disubstituted phenyl group or fused ring systems afforded the corresponding chiral products with good yields and with excellent ee values (**3at**–**3ax**), highlighting the potential applicability of this approach to more complex molecular architectures.

To further examine the generality and outreach of this asymmetric synthetic approach, the selenium atom in substrate **1a** was replaced with its chalcogen analogues or a carbon atom (Figure 5c, **3ay**–**3ba**). To our delight, the enantioenriched oxygen-based product **3ay** was delivered in 84% yield and with 93% ee, and the chiral sulfur-based product **3az** was delivered in 86% yield and with 98% ee, while **3ba** was only obtained in low yield and with 36% ee. These results imply the plausibility of the Se-coordinating tetradentate copper-centered catalyst complex proposed in the computational studies (Figure 4c): oxygen and sulfur could similarly donate a lone electron pair to the copper center, so the enantioselectivity-determining transition states were possibly analogous to the calculated results; such coordination was absent in the transition state for the formation of **3ba**, likely resulting in a lower enantioselectivity under the standard reaction conditions. The scope studies provided extra information for the catalytic mechanism of this method, and more importantly, the synthesis of other chiral chalcogen-based products could potentially inspire the development of more enantioenriched heteroatom-based synthons with versatile utility.

## Conclusions

Catalytic asymmetric synthesis of chiral organoselenium compounds has emerged as a promising area in modern synthetic chemistry. However, prior efforts have largely focused on preparing chiral Se-containing molecules with specific structures without fully exploring their downstream synthetic potential. To address this gap, we report a mild Cu­(II)/diamine-catalyzed asymmetric system for the synthesis of versatile chiral selenium-based versatile synthons. The demonstrated downstream transformations, from versatile synthetic intermediates to potential organocatalysts and bioactive agents, highlight the broad potential utility embedded within this Se-based molecular scaffold. A comprehensive substrate scope further establishes the generality of the method. While the present study focuses on the enantioselective desymmetrization of Se-containing 1,3-diols, extension to other Se-based prochiral substrates, such as 1,3-diamines and 1,5-diols, is appealing but requires further catalyst development. Preliminary experiments toward these substrates are currently underway in our laboratory, and we anticipate that the catalytic design principles established in this work could be adapted to enable the desymmetrization of other prochiral motifs. More broadly, this work delivers a simple and broadly applicable synthetic toolkit that may accelerate the discovery of new chiral organoselenium molecules and is poised to enrich the chemical space available to both synthetic and medicinal chemistry.

## Supplementary Material



## Notes

Reaction optimization, reaction procedure, product characterization, computational methods and various analyses, scans, structures, energy diagrams, maps, ESP, energies, coordinates, and NMR spectra (PDF)

## Notes

Deposition Number 2504122 (**3al**) contains the supplementary crystallographic data for this paper. These data can be obtained free of charge via the joint Cambridge Crystallographic Data Centre.
