# Supplementary material for: Copper-Catalyzed Enantioselective Synthesis of Chiral Selenium-Based Versatile Synthons
Source: ACS Cent Sci. 2026 Apr 29;12(5):627–37. doi: 10.1021/acscentsci.6c00281 (PMC13220204; doi:10.1021/acscentsci.6c00281)
Supplement: Supplementary file 1 [file oc6c00281_si_001.pdf]

Supporting Information for

Copper-Catalyzed Enantioselective Synthesis of Chiral Selenium-Based Versatile Synthons

Qingqin Huang[1,2], Jin-Xun Chen[1,2], Yu-Ping Tang,[1,2], Tian-Hao Shui[1], Jia-Xin Yi[1], Chao-Gang Zhang [1], and Lei Dai*,[1]

[1]Chongqing Key Laboratory of Natural Product Synthesis and Drug Research, School of Pharmaceutical Sciences, Chongqing University, Chongqing, 401331, China.

[2]These authors contributed equally to this work.

*Corresponding author: L. Dai, E-mail: dailei@cqu.edu.cn

Table of Contents

1. General Information S3

2. Experimental Procedures S3

3. Mechanistic and Computational Studies S38

4. Single Crystal Structure X-ray Analysis of **3al** S90

5. References S95

6. NMR Spectra S97

7. HPLC Spectra S228

1. General Information

All starting materials were obtained from commercial suppliers (Mreda, Macklin, Chemxyz, Adamas, J&K Sigma Aldrich, TCI, and BLD Pharm etc.) and directly used without further purification unless otherwise stated. Analytical thin layer chromatography was carried out with silica gel pre-coated glass plates (TLC-Silica gel GF254, coating thickness: 0.25 mm) purchased from XINNUO. Visualization was accomplished with short wave UV light (254nm) and/or 10% phosphomolybdic acid in ethanol or $KMnO_4$ staining solutions followed by heating. Column chromatography was performed on silica gel 200~300 mesh. ^1H NMR and ^{13}C NMR spectra were recorded on Agilent 400MR DD2 (400 MHz) spectrometer or Agilent 600MR DD2 (600 MHz) spectrometer. Chemical shifts were calibrated using residual solvent as an internal reference ($CDCl_3$: 7.26 ppm ^1H NMR, 77.00 ppm ^{13}C NMR). ^1H NMR Spectroscopy splitting patterns were designated as singlet (s), doublet (d), triplet (t), quartet (q). Splitting patterns that could not be interpreted or easily visualized were designated as multiplet (m). X-ray crystallography analysis of single crystal was performed on an Agilent SuperNova-CCD X-Ray diffractometer. Chiral HPLC analyses were performed on an Agilent 1100 Series using a Daicel Chiralpak column (IA, IB, IC, OJ-H, AS-H and AD-H) with hexanes/*i*-PrOH as the eluent.

2. Experimental Procedures

(1) Synthesis of prochiral substrate 1a

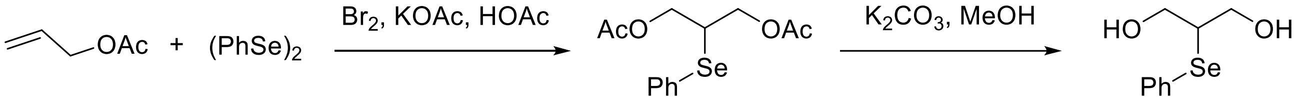

1a', 84% **1a**, 91%

Step 1: To glacial AcOH (50 mL) contained in a 100-mL flask equipped with a magnetic stirring bar, Br_2 (10.0 mmol, 1.6 g) was added dropwise and $(PhSe)_2$ (10.0 mmol, 3.2 g) was added subsequently. After stirring the mixture for 30 min, allyl acetate (34.0 mmol, 3.4 g) and anhydrous KOAc (40.0 mmol, 3.9 g) was added sequentially. After 5 min a white precipitate was observed, and the mixture was then stirred for 12 hours till completion. The resulting reaction mixture was added to H_2O (100 mL) and extracted with EtOAc (3*50 mL). The organic phase was washed with H_2O (50 mL and 10% aqueous Na_2CO_3 (3*50 mL), brine (50 mL), dried with Na_2SO_4, and rotary evaporated. The residue was purified by column chromatography on silica using a mixture of *n*-hexane/ EtOAc (*v*/*v* = 8:1) to afford **1a'** (2.7 g, 84%) as a colorless oil.

Step 2: **1a'** (5.0 mmol, 1.0 eq) was dissolved with 25 mL MeOH in a 50-mL flask equipped with a magnetic stirring bar, and K_2CO_3 (1.0 mmol, 0.2 eq) was added in one portion. And the mixture was stirred till completion (typically 3 hours). The organic phase was rotary evaporated. The residue was purified by column chromatography on silica using a mixture of *n*-hexane/ EtOAc (*v*/*v* = 3:1) to afford **1a** (1.1 g, 91%) as a colorless foam.

(2) Additional reaction optimization for the catalytic asymmetric synthesis of 3a

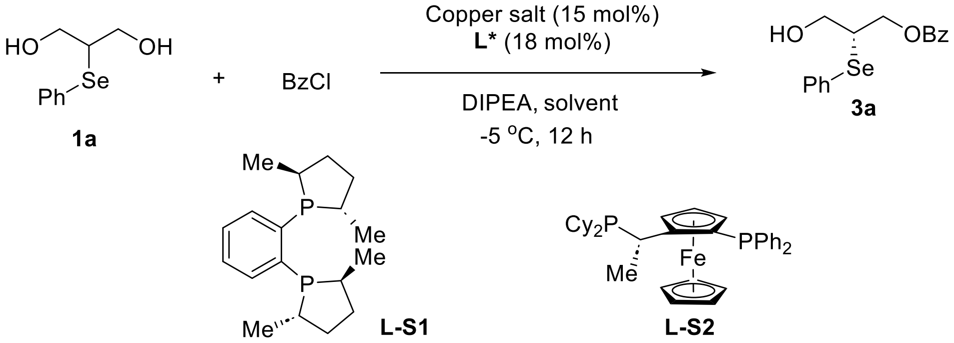

General procedure at 0.1 mmol: Copper salt (0.015 mmol, 0.15 eq) and **L*** (0.018 mmol, 0.18 eq) were added to solvent (2.0 mL) in a 10-mL tube equipped with a magnetic stirring bar. After stirring for 30 min, **1a** (0.1 mmol, 1.0 eq) was added to the solution and the mixture was cooled to -5 °C. Benzoyl chloride (0.12 mmol, 1.2 eq) was then added to the mixture, followed by the addition of DIPEA (0.12 mmol, 1.2 eq). Then the mxiture was allowed to stir at -5 °C for 12 hours. 5 mL H_2O was poured into the reaction and the mixture was extracted with CH_2Cl_2, washed with brine and dried with Na_2SO_4. The combined organic phase was rotary evaporated. The residue was purified by column chromatography on silica to afford the product.

Table S1. Additional reaction optimization data

Entry	Copper precat.	L*	Solvent	Results
1	$Cu(NO_3)_2$•xH_2O	**L-S1**	CH_2Cl_2	23%, 5% ee
2	$Cu(NO_3)_2$•xH_2O	**L-S2**	CH_2Cl_2	35%, 8% ee
3	$Cu(MeCN)_4BF_4$	**L1**	CH_2Cl_2	76%, 89% ee
4	$Cu(MeCN)_4PF_6$	**L1**	CH_2Cl_2	87%, 82% ee
5	CuCN	**L1**	CH_2Cl_2	63%, 75% ee
6	$Cu(acac)_2$	**L1**	CH_2Cl_2	59%, 2% ee
7	$Cu(NO_3)_2$•xH_2O	**L1**	DCE	77%, 78% ee
8	$Cu(NO_3)_2$•xH_2O	**L1**	PhMe	67%, 13% ee
9	$Cu(NO_3)_2$•xH_2O	**L1**	Acetone	52%, 62% ee
10	$Cu(NO_3)_2$•xH_2O	**L1**	Et_2O	N.D.

(3) Standard conditions for the catalytic asymmetric synthesis of 3a

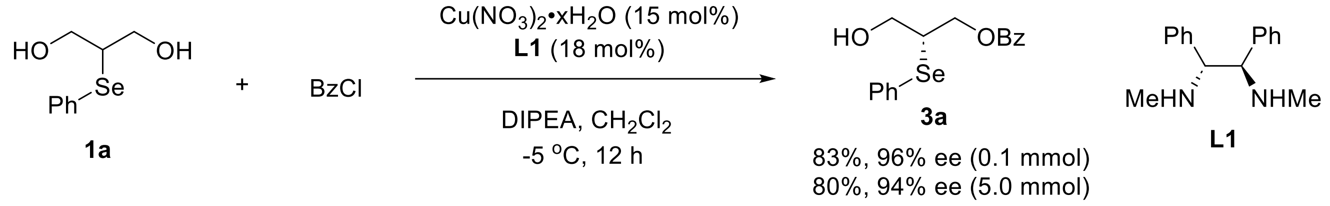

General procedure at 0.1 mmol: $Cu(NO_3)_2{\bullet}xH_2O$ (CAS#13778-31-9 from Macklin) (0.015 mmol, 0.15 eq) and **L1** (0.018 mmol, 0.18 eq) were added to DCM (2.0 mL) in a 10-mL tube equipped with a magnetic stirring bar. After stirring for 30 min, **1a** (0.1 mmol, 1.0 eq) was added to the solution and the mixture was cooled to -5 °C. Benzoyl chloride (0.12 mmol, 1.2 eq) was then added to the mixture, followed by the addition of DIPEA (0.12 mmol, 1.2 eq). Then the mxiture was allowed to stir at -5 °C for 12 hours. 5 mL H_2O was poured into the reaction and the mixture was extracted with CH_2Cl_2. The combined organic phase was rotary evaporated. The residue was purified by column chromatography on silica.

General procedure at 5.0 mmol: $Cu(NO_3)_2{\bullet}xH_2O$ (0.75 mmol, 0.15 eq) and **L1** (0.90 mmol, 0.18 eq) were added to DCM (10 mL) in a 25-mL flask equipped with a magnetic stirring bar. After solid was completely dissolved, **1a** (5.0 mmol, 1.0 eq) was added to the solution and the mixture was cooled to -5 °C. After 5 min, benzoyl chloride (0.60 mmol, 1.2 eq) and DIPEA (0.12 mmol, 1.2 eq) were sequentially added dropwise to the solution. Then the mxiture was allowed to stir at -5 °C till complete consumption of **1a**. 5 mL H_2O was poured into the reaction and the mixture was extracted with CH_2Cl_2. The combined organic phase was rotary evaporated. The residue was purified by column chromatography on silica.

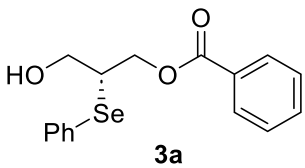

3a

(*R*)-3-hydroxy-2-(phenylselanyl)propyl benzoate (3a)

83% yield, a pale-yellow oil, R_f = 0.3 (PE : DCM : Acetone = 5:5:0.2).

^1H NMR (400 MHz, $CDCl_3$) δ 8.01 (d, *J* = 7.9 Hz, 2H), 7.63 (d, *J* = 7.4 Hz, 2H), 7.58 (t, *J* = 7.4 Hz, 1H), 7.44 (t, *J* = 7.6 Hz, 2H), 7.33 – 7.28 (m, 3H), 4.74 (dd, *J* = 11.6, 4.9 Hz, 1H), 4.57 (dd, *J* = 11.5, 7.8 Hz, 1H), 3.96 – 3.74 (m, 2H), 3.65 – 3.47 (m, 1H), 2.50 (s, 1H).

^{13}C NMR (101 MHz, $CDCl_3$) δ 166.6, 135.1, 133.3, 129.7, 129.6, 129.3, 128.4, 128.2, 127.1, 64.4, 62.1, 46.5.

HRMS (ESI) m/z calcd for $C_{16}H_{17}O_3Se^+$ $[M+H]^+$ = 337.0337, found = 337.0335.

The ee value was 96%, t_R (major) = 10.7 min, t_R (minor) = 12.3 min (Chiralpak IB, λ = 254 nm, 10% *i*-PrOH/Hexane, flow rate = 1 mL/min).

(4) Asymmetric synthesis of 5 from 3a

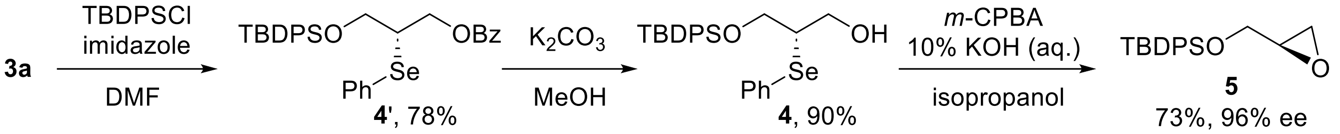

Step 1: To the solution of **3a** (1 mmol, 1.0 eq) in anhydrous DMF (3.0 mL) under Argon atomosphere in a two-neck 25-mL flask equipped with a magnetic stirring bar, a solution of TBDPSCl (1.5 mmol, 1.5 eq) in 2 mL anhydrous DMF was added dropwise over 5 min. Then imidazole (1.5 mmol, 1.5 eq) was added in one portion, and the mixture was then allowed to stir for 6 hours till completion. The resulting reaction mixture was added to H_2O (10 mL) and extracted with EtOAc (3*20 mL), and the combine orgranic phase was washed with brine (20 mL), dried with Na_2SO_4, and rotary evaporated. The curde product **4'** was used in next step without further purification.

Step 2: **4a'** (0.5 mmol, 1.0 eq) was dissolved with 3 mL MeOH in a 50-mL flask equipped with a magnetic stirring bar, and K_2CO_3 (0.1 mmol, 0.2 eq) was added in one portion. And the mixture was stirred till completion (typically 3 hours). The organic phase was rotary evaporated. The residue was purified by column chromatography on silica using a mixture of *n*-hexane/ EtOAc (*v*/*v* = 8:1) to afford **4** (212 mg, 90%) as a colorless oil.

Step 3: To the solution of **4** (0.1 mmol, 1.0 eq) in 2.0 mL IPA, *m*-CPBA (0.5 mmol, 5.0 eq) was added in one portion. After 5 min, 0.2 mL 10% KOH (aq.) was added dropwise. And the mixture was stirred till completion (monitored by TLC). The organic phase was rotary evaporated. The residue was purified by column chromatography on silica using a mixture of *n*-hexane/EtOAc (*v*/*v* = 8:1) to afford **5** (23 mg, 73%) as a pale-yellow oil.

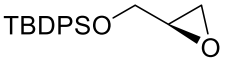

5

(*R*)-*tert*-butyl(oxiran-2-ylmethoxy)diphenylsilane (5)

23mg, 73% yield, a pale-yellow oil, R_f = 0.4 (PE : EtOAc = 10:1).

^{1}H NMR (400 MHz, $CDCl_3$) δ 7.70 (d, *J* = 6.5 Hz, 4H), 7.41 (q, *J* = 8.6 Hz, 6H), 3.86 (dd, *J* = 11.8, 2.8 Hz, 1H), 3.72 (dd, *J* = 11.8, 4.6 Hz, 1H), 3.18 – 3.10 (m, 1H), 2.75 (t, *J* = 4.5 Hz, 1H), 2.66 – 2.59 (m, 1H), 1.07 (s, 9H).

^{13}C NMR (101 MHz, $CDCl_3$) δ 135.6, 135.5, 133.2, 129.7, 127.7, 64.3, 52.3, 44.4, 26.7, 19.2.

HRMS (ESI) m/z calcd for $C_{19}H_{25}O_2Si^+$ $[M+H]^+$ = 313.1618, found = 313.1619.

The ee value was 95%, t_R (major) = 10.0 min, t_R (minor) = 9.0 min (Chiralpak AS-H, λ = 254 nm, 1% *i*-PrOH/Hexane, flow rate = 0.5 mL/min).

(5) Asymmetric synthesis of 8 from 4

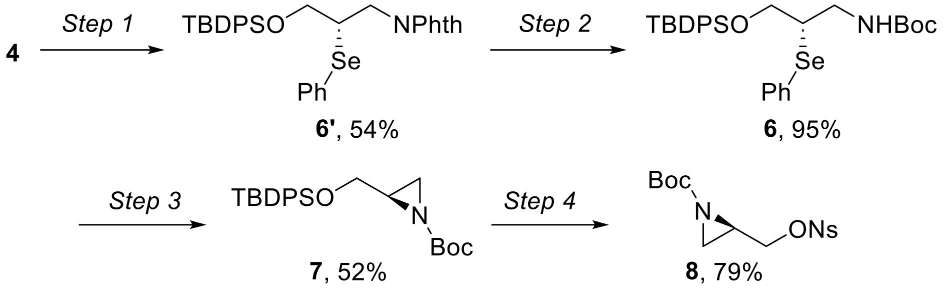

Step 1: To a flame-dried 25-mL two-neck flask equipped with a magnetic stirring bar, phthalimide (1.5 mmol, 1.5 eq) and PPh_3 (1.5 mmol, 1.5 eq) were dissolved in 10 mL THF (freshly distilled). The solution was then cooled to 0 °C and **4** (1.0 mmol, 1.0 eq) was added in one portion, followed by the addition of DEAD (1.5 mmol, 1.5 eq) over 30 min. The organic phase was rotary evaporated, and the residue was purified by column chromatography on silica using a mixture of *n*-hexane/ EtOAc (*v*/*v* = 10:1) to afford **6'** (324 mg, 54%) as a colorless oil.

Step 2: To the solution of **6'** (0.5 mmol, 1.0 eq) in 5 mL MeOH, $N_2H_4 \cdot H_2O$ (2.5 mmol, 5 eq) was added dropwise at room temperaure. Then the mixture was allows to warm to 65 °C under reflux for 5 hours till complete consumption of **6'** (monitored by TLC). The mixture was added 10 mL 10% KOH (aq.) and extraced with DCM (20 mL*3). The combined organic phase was washed with brine, dried with Na_2SO_4, and rotary evaporated. The crude product was dissolved with 5 mL DCM, and TEA (1.5 eq), $(Boc)_2O$ (1.5 eq) and DMAP (0.1 eq) were added sequentially. After complete consumption of the starting material, The organic phase was rotary evaporated, and the residue was purified by column chromatography on silica using a mixture of *n*-hexane/ EtOAc (*v*/*v* = 10:1) to afford **6** (270 mg, 95%) as a colorless oil.

Step 3: To the solution of **6** (0.2 mmol, 1.0 eq) in dry THF at -60 °C, *m*-CPBA (0.6 mmol, 3.0 eq) was added and the solution was stirred at -60 °C for 1 hour. *t*-BuOK (1.0 mmol. 5.0 eq) was added in three portions, and then the yellowish suspension was allowed to warm to 0 °C and stirred for another hour. After complete consumption of **6**, 2 mL $Na_2S_2O_3$ (1.0 M, aq) and 3 mL $NaHCO_3$ (sat. aq.) were added sequentially to the mixture and the mixture was extracted with DCM. The orgainc phase was wahsed with 10% NaOH aq. and brine, dried over Na_2SO_4, and rotary evaporated. The residue was purified by column chromatography on silica using a mixture of *n*-hexane/ EtOAc (*v*/*v* = 15:1) to afford **7** (43 mg, 52%) as a colorless oil.

Step 4: To the solution of **7** (0.05 mmol, 1.0 eq) in dry THF (0.50 ml) at 0 °C, TBAF (0.1 mmol, 2.0 eq) was added dropwise to the solution and the resulting mixture was stirred for 3 hours. 3 mL $NaHCO_3$ (sat. aq.) were added sequentially to the mixture and the mixture was extracted with DCM. The orgainc phase was wahsed with brine, dried over Na_2SO_4, and rotary evaporated. The curde product was dissolved in DCM (0.1 M), and NsCl (1.5 eq), TEA (1.5 eq) and DMAP (0.1 eq) were added sequentilly to the mixture. The organic phase was rotary evaporated, and the residue was purified by column chromatography on silica using a mixture of *n*-hexane/ EtOAc (*v*/*v* = 4:1) to afford **8** (14 mg, 79%) as a colorless oil.

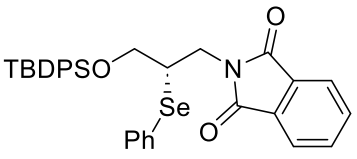

6'

(*S*)-2-(3-((*tert*-butyldiphenylsilyl)oxy)-2-(phenylselanyl)propyl)isoindoline-1,3-dione (6')

54% yield, a colorless oil, R_f = 0.3 (PE : EtOAc = 8:1).

^{1}H NMR (400 MHz, $CDCl_3$) δ 7.80 – 7.75 (m, 2H), 7.71 – 7.64 (m, 6H), 7.42 – 7.33 (m, 8H), 7.07 (d, *J* = 6.8 Hz, 3H), 4.23 (dd, *J* = 14.1, 6.3 Hz, 1H), 4.10 (dd, *J* = 12.8, 10.0 Hz, 1H), 4.00 – 3.80 (m, 3H), 1.07 (s, 9H).

^{13}C NMR (101 MHz, $CDCl_3$) δ 168.1, 135.6, 135.6, 133.8, 133.6, 133.0, 133.0, 131.9, 129.7, 129.7, 128.9, 128.3, 127.7, 127.1, 123.1, 65.4, 44.7, 40.8, 26.7, 19.2.

HRMS (ESI) m/z calcd for $C_{33}H_{34}NO_3SeSi^+$ $[M+H]^+$ = 600.1468, found = 600.1467.

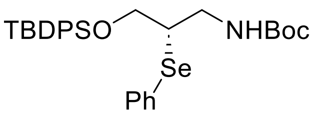

6

***Tert*-butyl (*S*)-(3-((tert-butyldiphenylsilyl)oxy)-2-(phenylselanyl)propyl)carbamate (6)**

270 mg, 95% yield, a colorless oil, R_f = 0.5 (PE : EtOAc = 10:1).

^{1}H NMR (400 MHz, $CDCl_3$) δ 7.71 – 7.58 (m, 4H), 7.51 – 7.32 (m, 8H), 7.25 – 7.17 (m, 3H), 5.06 (s, 1H), 4.00 – 3.84 (m, 2H), 3.64 – 3.42 (m, 2H), 3.35 (s, 1H), 1.42 (s, 9H), 1.07 (s, 9H).

^{13}C NMR (101 MHz, $CDCl_3$) δ 155.7, 135.6, 135.5, 134.3, 133.0, 132.9, 129.8, 129.1, 128.5, 127.8, 127.8, 127.5, 79.1, 65.9, 46.7, 43.3, 28.4, 26.8, 19.2.

HRMS (ESI) m/z calcd for $C_{30}H_{40}NO_3SeSi^+$ $[M+H]^+$ = 570.1937, found = 570.1939.

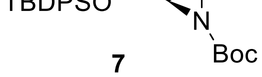

7

***Tert*-butyl (*R*)-2-(((tert-butyldiphenylsilyl)oxy)methyl)aziridine-1-carboxylate (7)**

43 mg, 52% yield, a colorless oil, R_f = 0.6 (PE : EtOAc = 10:1).

^{1}H NMR (600 MHz, $CDCl_3$) δ 7.68 (d, *J* = 8.1 Hz, 4H), 7.52 – 7.33 (m, 6H), 3.81 (dd, *J* = 11.3, 5.0 Hz, 1H), 3.64 (dd, *J* = 11.3, 5.0 Hz, 1H), 2.64 – 2.53 (m, 1H), 2.23 (d, *J* = 6.0 Hz, 1H), 1.97 (d, 1H), 1.44 (s, 9H), 1.06 (s, 9H).

^{13}C NMR (151 MHz, $CDCl_3$) δ 162.2, 135.6, 133.4, 133.3, 129.7, 127.7, 127.7, 81.1, 64.4, 38.4, 29.3, 27.9, 26.8, 19.2.

HRMS (ESI) m/z calcd for $C_{24}H_{34}NO_3Si^+$ $[M+H]^+$ = 412.2302, found = 412.2300.

The ee value was 85%, t_R (major) = 4.4 min, t_R (minor) = 4.7 min (Chiralpak IB, λ = 254 nm, 2% *i*-PrOH/Hexane, flow rate = 1 mL/min).

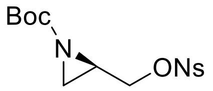

8

***Tert*-butyl (*R*)-2-((((4-nitrophenyl)sulfonyl)oxy)methyl)aziridine-1-carboxylate (8)**

14 mg, 79% yield, a colorless oil, R_f = 0.3 (PE : EtOAc = 4:1).

^{1}H NMR (600 MHz, $CDCl_3$) δ 8.39 (d, *J* = 8.8 Hz, 2H), 8.13 (d, *J* = 7.3 Hz, 2H), 4.25 – 4.09 (m, 2H), 2.72 (s, 1H), 2.33 (d, *J* = 6.2 Hz, 1H), 2.06 (d, *J* = 5.0 Hz, 1H), 1.42 (s, 9H).

HRMS (ESI) m/z calcd for $C_{14}H_{19}N_2O_7S^+$ $[M+H]^+$ = 359.0907, found = 359.0910.

(6) Asymmetric synthesis of 10 and 11 from 3a

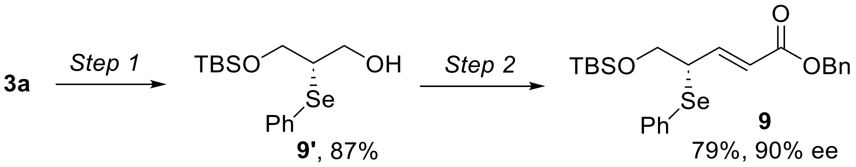

Step 1: To the solution of **3a** (1 mmol, 1.0 eq) in anhydrous DMF (3.0 mL) under Argon atomosphere in a two-neck 25-mL flask, a solution of TBSCl (1.5 mmol, 1.5 eq) in 2 mL anhydrous DMF was added dropwise over 5 min. Then imidazole (1.5 mmol, 1.5 eq) was added in one portion, and the mixture was then allowed to stir for 6 hours till completion. The resulting reaction mixture was added to H_2O (10 mL) and extracted with EtOAc (3*20 mL), and the combine orgranic phase was washed with brine (20 mL), dried with Na_2SO_4, and rotary evaporated. The curde product was dissolved with 5 mL MeOH in a 25-mL flask, and K_2CO_3 (0.2 mmol, 0.2 eq) was added in one portion. And the mixture was stirred till completion (typically 3 hours). The organic phase was rotary evaporated. The residue was purified by column chromatography on silica using a mixture of *n*-hexane/EtOAc (*v*/*v* = 10:1) to afford **9'** (300 mg, 87%) as a pale-yellow oil.

Step 2: To the solution of **9'** (0.5 mmol,1.0 eq) in 5 mL DCM at 0 °C, Dess-Martin peroxide (1.0 mmol, 2.0 eq) was added in three portions over 30 min. 3 mL $NaHCO_3$ (sat. aq.) was added to the mixture and the mixture was extracted with DCM. The orgainc phase was wahsed with brine, dried over Na_2SO_4, and rotary evaporated. Dissolved the crude product in DCM (0.1 M) and added Ph_3P=COOBn (1.2 eq) in one portion. After the complete consumption of the starting material, The organic phase was rotary evaporated. The residue was purified by column chromatography on silica using a mixture of *n*-hexane/ EtOAc (*v*/*v* = 20:1) to afford **9** (188 mg, 79%) as a pale-yellow oil.

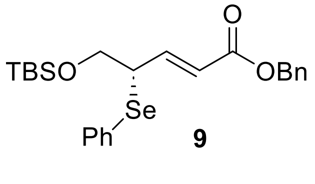

9

Benzyl (*S,E*)-5-((tert-butyldimethylsilyl)oxy)-4-(phenylselanyl)pent-2-enoate (9)

188 mg, 79% yield, a pale-yellow oil, R_f = 0.5 (PE : EtOAc = 15:1).

^{1}H NMR (600 MHz, $CDCl_3$) δ 7.51 (d, *J* = 7.6 Hz, 2H), 7.43 – 7.18 (m, 8H), 7.02 (dd, *J* = 15.6, 9.1 Hz, 1H), 5.54 (d, *J* = 15.5 Hz, 1H), 5.15 (m, 2H), 3.96 – 3.79 (m, 3H), 0.88 (s, 9H), 0.04 (s, 6H).

^{13}C NMR (151 MHz, $CDCl_3$) δ 165.9, 146.1, 136.1, 135.9, 129.0, 128.5, 128.3, 128.1, 128.0, 127.7, 120.7, 66.0, 64.8, 47.8, 25.8, 18.3, -5.4, -5.4.

HRMS (ESI) m/z calcd for $C_{24}H_{33}O_3SeSi^+$ $[M+H]^+$ = 477.1359, found = 477.1361.

The ee value was 89%, t_R (major) = 5.5 min, t_R (minor) = 5.2 min (Chiralpak AD-H, λ = 254 nm, 2% *i*-PrOH/Hexane, flow rate = 1 mL/min).

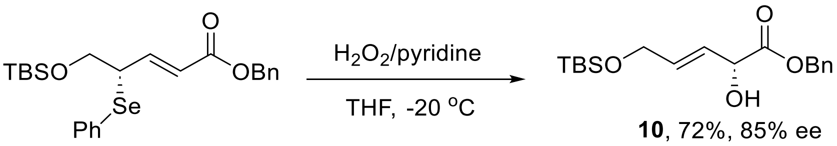

Synthesis of 10: To the solution of **3aa** (0.1 mmol, 1.0 equiv) in dry THF (1.0 mL) under Ar atmosphere, pyridine (0.3 mL) and H_2O_2 (0.2 mmol, 2.0 eq) were added slowly at - 20 °C and then the solution was allowed to stir at -20 °C for 2 hours. After the complete consumption of **9**, the solution was then added NH_4Cl (sat. aq., 5 mL) and extracted with DCM (10 mL x 3). The combined organic layers were washed with brine, dried with Na_2SO_4 and rotary evaporated. The residue was purified by silica gel column chromatography *n*-hexane/EtOAc (*v*/*v* = 4:1) to give **10** (72%, 85% ee) as a colorless oil.

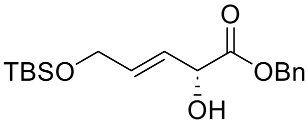

10

Benzyl (*R,E*)-5-((tert-butyldimethylsilyl)oxy)-2-hydroxypent-3-enoate (10)

24 mg, 72% yield, a pale-yellow oil, R_f = 0.3 (PE : EtOAc = 4:1).

^{1}H NMR (600 MHz, $CDCl_3$) δ 7.42 – 7.29 (m, 5H), 5.99 (dd, *J* = 15.4, 4.3 Hz, 1H), 5.82 (dd, *J* = 15.3, 5.3 Hz, 1H), 5.30 – 5.17 (m, 2H), 4.73 (s, 1H), 4.19 (s, 2H), 2.93 (s, 1H), 0.89 (s, 9H), 0.04 (s, 6H).

^{13}C NMR (151 MHz, $CDCl_3$) δ 176.0, 137.7, 135.2, 131.3, 131.2, 130.9, 128.0, 73.5, 70.3, 65.3, 32.3, 28.5, -2.7.

HRMS (ESI) m/z calcd for $C_{18}H_{29}O_4Si^+$ $[M+H]^+$ = 377.1830, found = 377.1826.

The ee value was 85%, t_R (major) = 6.1 min, t_R (minor) = 5.6 min (Chiralpak AD-H, λ = 210 nm, 10% *i*-PrOH/Hexane, flow rate = 1 mL/min).

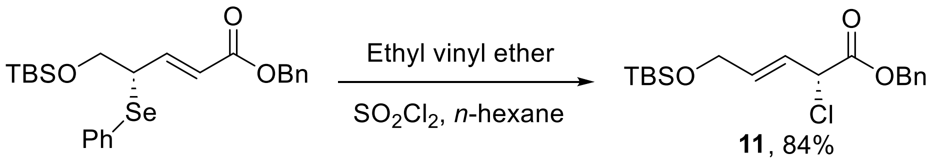

11, 84%

Synthesis of 11: To the solution of **9** (0.2 mmol, 1.0 equiv) in 1.0 mL *n*-hexane. Ethyl vinyl ether (2.4 mmol, 12.0 equiv) and a solution of sulfuryl chloride (0.4 mmol, 2.0 equiv) in 1.0 mL *n*-hexane were sequentially dropwise added. The reaction mixture was then allowed to stir at room temperature for 10 minutes. The reaction mixture was directly rotary evaporated, and the residue was purified column chromatography on silica using a *n*-hexane/EtOAc (*v*/*v* = 20:1) to afford **11** (84%) as a colorless oil.

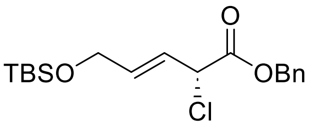

11

Benzyl (*R*,*E*)-5-((tert-butyldimethylsilyl)oxy)-2-chloropent-3-enoate (11)

60 mg, 84% yield, a colorless oil, R_f = 0.4 (PE : EtOAc = 20:1).

^{1}H NMR (600 MHz, $CDCl_3$) δ 7.42 – 7.29 (m, 5H), 5.96 (d, *J* = 5.3 Hz, 2H), 5.21 (d, *J* = 4.0 Hz, 2H), 4.86 (d, *J* = 7.3 Hz, 1H), 4.20 (s, 2H), 0.90 (s, 9H), 0.06 (s, 6H).

^{13}C NMR (151 MHz, $CDCl_3$) δ 170.8, 138.9, 137.7, 131.3, 131.2, 130.9, 126.2, 70.4, 65.0, 60.0, 28.5, 21.0, -2.7.

HRMS (ESI) m/z calcd for $C_{18}H_{28}ClO_3Si^+$ $[M+H]^+$ = 355.1491, found = 355.1490.

(7) Asymmetric synthesis of organocatalysts 13 and 14 from 4

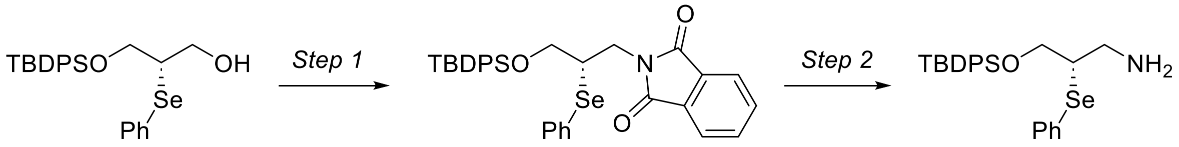

4 **6'**, 54% **12,** 91%

Step 1: see **Asymmetric synthesis of 8 from 4** in **Experimental Procedures** for detailed method. **12'** (324 mg, 0.54 mmol, 54%) was afford as a colorless oil.

Step 2: To the solution of **6'** (0.5 mmol, 1.0 eq) in 5 mL MeOH, $N_2H_4 \cdot H_2O$ (2.5 mmol, 5 eq) was added dropwise at room temperaure. Then the mixture was allows to warm to 65 °C under reflux for 5 hours till complete consumption of **6'** (monitored by TLC). The mixture was added 10 mL 10% KOH (aq.) and extraced with DCM (20 mL*3). The combined organic phase was washed with brine, dried with Na_2SO_4, and rotary evaporated. **12** (91%, pale-yellow oil) could be used in the next step without further purification.

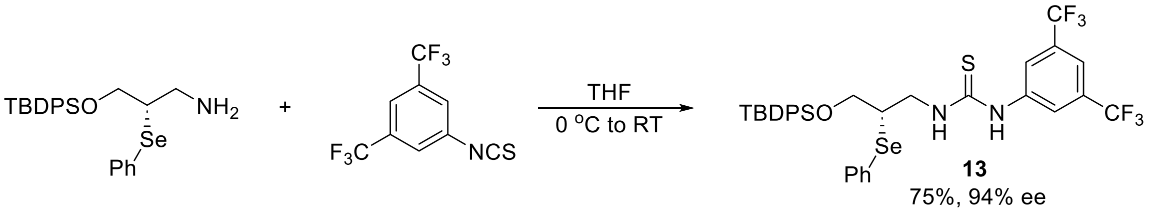

Synthesis of 13: To the solution of **12** (0.1 mmol, 1.0 eq) in 5 mL dry THF at 0 °C, 3,5-di(trifluoromethyl)phenyl isothiocyanate (0.12 mmol, 1.2 eq) added slowly. Then the reaction mixture was alloed to stir at room temperature for 1 hour. The reaction mixture was directly rotary evaporated, and the residue was purified column chromatography on silica using a *n*-hexane/EtOAc (*v*/*v* = 6:1) to afford **13** (75%, 94% ee) as a colorless oil.

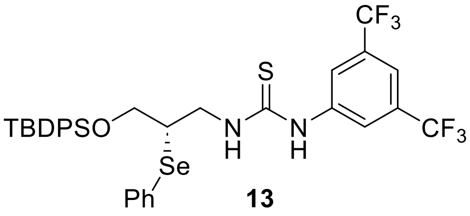

13

(*S*)-1-(3,5-bis(trifluoromethyl)phenyl)-3-(3-((tert-butyldiphenylsilyl)oxy)-2-(phenylselanyl)propyl)thiourea (13)

56 mg, 75% yield, a colorless oil, R_f = 0.2 (PE : EtOAc = 3:1).

^{1}H NMR (400 MHz, $CDCl_3$) δ 7.98 (s, 1H), 7.79 – 7.52 (m, 7H), 7.52 – 7.29 (m, 8H), 7.27 – 7.16 (m, 3H), 6.72 (s, 1H), 4.17 (d, *J* = 17.4 Hz, 1H), 4.03 (dd, *J* = 10.8, 3.5 Hz, 1H), 3.98 – 3.81 (m, 2H), 3.58 (s, 1H), 1.04 (s, 9H).

^{13}C NMR (151 MHz, $cdcl_3$) δ 180.5, 139.0, 135.5, 135.5, 134.0, 133.0, 132.8, 132.6, 132.6, 130.2, 130.0, 129.9, 129.3, 127.9, 127.9, 125.5, 123.7, 121.9, 120.1, 119.2, 65.9, 47.5, 45.4, 26.8, 19.2.

HRMS (ESI) m/z calcd for $C_{34}H_{35}F_6N_2OSSeSi^+$ $[M+H]^+$ = 741.1303, found = 741.1306.

The ee value was 94%, t_R (major) = 9.1 min, t_R (minor) = 10.3 min (Chiralpak IB, λ = 254 nm, 5% *i*-PrOH/Hexane, flow rate = 1 mL/min).

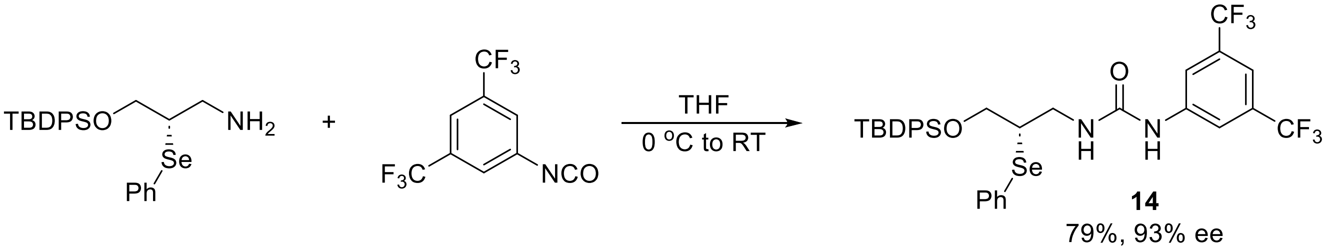

Synthesis of 14: To the solution of **12** (0.1 mmol, 1.0 eq) in 5 mL dry THF at 0 °C, 3,5-di(trifluoromethyl)phenyl isocyanate (0.12 mmol, 1.2 eq) added slowly. Then the reaction mixture was alloed to stir at room temperature for 1 hour. The reaction mixture was directly rotary evaporated, and

the residue was purified column chromatography on silica using a *n*-hexane/EtOAc (*v*/*v* = 6:1) to afford **14** (79%, 93% ee) as a colorless oil.

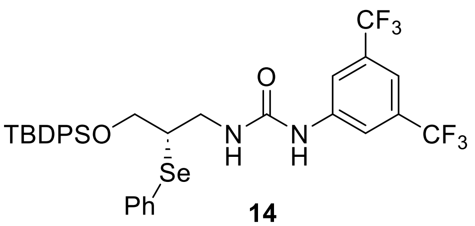

14

(*S*)-1-(3,5-bis(trifluoromethyl)phenyl)-3-(3-((tert-butyldiphenylsilyl)oxy)-2-(phenylselanyl)propyl)urea (14)

57 mg, 79% yield, a colorless oil, R_f = 0.2 (PE : EtOAc = 2:1).

^{1}H NMR (400 MHz, $CDCl_3$) δ 7.76 – 7.61 (m, 6H), 7.52 – 7.34 (m, 9H), 7.27 – 7.16 (m, 3H), 6.35 (s, 1H), 5.28 (t, *J* = 5.4 Hz, 1H), 4.12 – 3.93 (m, 2H), 3.74 – 3.57 (m, 2H), 3.45 – 3.35 (m, 1H), 1.10 (s, 8H).

^{13}C NMR (151 MHz, $CDCl_3$) δ 156.7, 143.0, 138.3, 138.3, 137.4, 136.8, 136.1, 135.5, 134.8, 134.6, 132.7, 132.3, 131.9, 130.8, 130.7, 130.6, 130.4, 130.4, 126.7, 124.9, 121.1, 118.4, 68.7, 48.8, 45.8, 29.6, 22.1.

HRMS (ESI) m/z calcd for $C_{34}H_{35}F_6N_2O_2SeSi^+$ $[M+H]^+$ = 725.1532, found = 725.1533.

The ee value was 93%, t_R (major) = 9.6 min, t_R (minor) = 11.1 min (Chiralpak IB, λ = 254 nm, 5% *i*-PrOH/Hexane, flow rate = 1 mL/min).

(8) Asymmetric synthesis of (*S*)-16

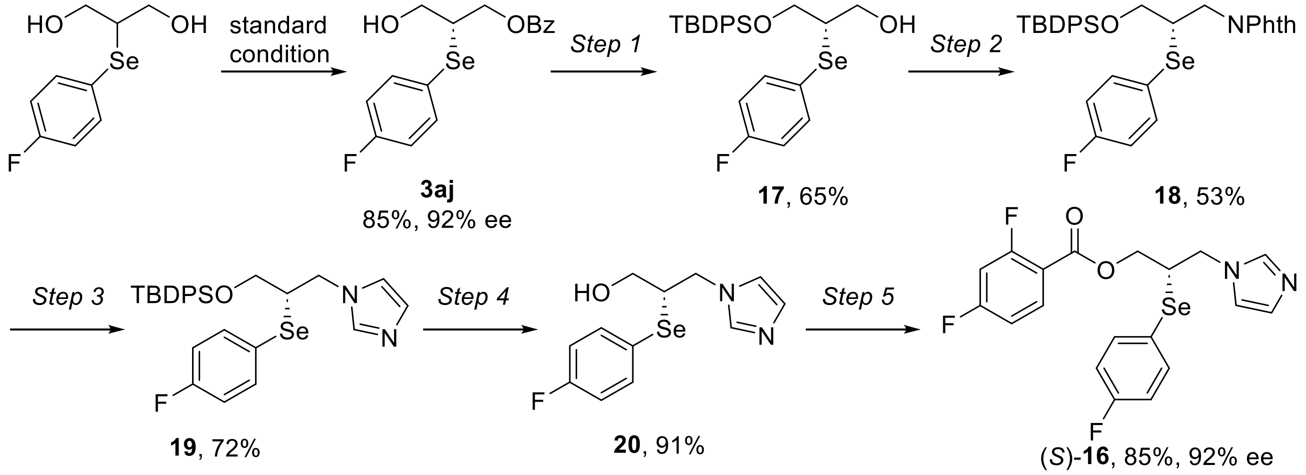

Step 1: To the solution of **3aj** (1 mmol, 1.0 eq) in anhydrous DMF (3.0 mL) under Argon atomosphere in a two-neck 25-mL flask equipped with a magnetic stirring bar, a solution of TBDPSCl (1.5 mmol, 1.5 eq) in 2 mL anhydrous DMF was added dropwise over 5 min. Then imidazole (1.5 mmol, 1.5 eq) was added in one portion, and the mixture was then allowed to stir for 6 hours till completion. The resulting reaction mixture was added to H_2O (10 mL) and extracted with EtOAc (3*20 mL), and the combine orgranic phase was washed with brine (20 mL), dried with Na_2SO_4, and rotary evaporated. The residue was

dissolved with 3 mL MeOH in a 50-mL flask equipped with a magnetic stirring bar, and K_2CO_3 (0.1 mmol, 0.2 eq) was added in one portion. And the mixture was stirred till completion (typically 3 hours). The organic phase was rotary evaporated. The residue was purified by column chromatography on silica using a mixture of *n*-hexane/EtOAc (*v*/*v* = 8:1) to afford **17** (317 mg, 65%) as a colorless oil.

Step 2: To a flame-dried 25-mL two-neck flask equipped with a magnetic stirring bar, phthalimide (0.75 mmol, 1.5 eq) and PPh_3 (0.75 mmol, 1.5 eq) were dissolved in 10 mL THF (freshly distilled). The solution was then cooled to 0 °C and **4** (0.5 mmol, 1.0 eq) was added in one portion, followed by the addition of DEAD (0.75 mmol, 1.5 eq) over 30 min. The organic phase was rotary evaporated, and the residue was purified by column chromatography on silica using a mixture of *n*-hexane/EtOAc (*v*/*v* = 10:1) to afford **18** (164 mg, 53%) as a colorless oil.

Step 3: To the solution of **18** (0.2 mmol, 1.0 eq) in 2 mL MeOH, $N_2H_4 \cdot H_2O$ (1.0 mmol, 5 eq) was added dropwise at room temperaure. Then the mixture was allows to warm to 65 °C under reflux for 5 hours till complete consumption of **18** (monitored by TLC). The mixture was added 2 mL 10% KOH (aq.) and extraced with DCM (5 mL*3). The combined organic phase was washed with brine, dried with Na_2SO_4, and rotary evaporated. The residue was dissolved in 4 mL dry MeOH under Ar atmosphere, and formaldehyde (36 wt%, 2.0 eq), glyoxal (40 wt%, 2.0 eq), anhydrous NH_4Cl (2.0 eq) were added to the solution. The mixture was warmed to 65 °C and stirred for 24 hours. After the completie consumption of the starting material, the reationc was cooled to room temperature. The solvent was rotary evaporated, and KOH (sat. aq.) was added until pH = 10. Then the mixture was extracted with DCM, dried with Na_2SO_4. The combined organic phase was rotary evaporated, and the The crude product **19** was used directly in the next step without further purification.

Step 4: To a 10-mL tube equipped with a magnetic stirring bar, **19** (0.1 mmol, 1.0 eq) was dissolved in 1 mL dry THF at 0 °C, followed by the dropwise addition of TBAF (1.0 M in THF, 0.2 mmol, 2.0 eq). The mixture was stirred at 0 °C till completion, and then was added with 2 mL $NaHCO_3$ (sat. aq.), extracted with DCM, and rotary evaporated. The crude product **20** was used directly in the next step without further purification.

Step 5: To a 10-mL tube equipped with a magnetic stirring bar, **20** (0.1 mmol, 1.0 eq), 2,4-difluorobenzoyl chloride (0.12 mmol, 1.2 eq), TEA (0.15 mmol, 1.5 eq) and DMAP (0.01 mmol, 0.1 eq) was dissolved in 2 mL DCM and stirred till completion. The organic phase was rotary evaporated, and the residue was purified by column chromatography on silica using a mixture of DCM/MeOH (*v*/*v* = 10:1) to afford **16** (37 mg, 85%, 92% ee) as a colorless foam.

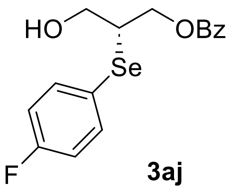

3aj

(*R*)-2-((4-fluorophenyl)selanyl)-3-hydroxypropyl benzoate (3aj)

85% yield, a pale-yellow oil, R_f = 0.3 (PE : DCM : Acetone = 5:5:0.2).

^{1}H NMR (400 MHz, $CDCl_3$) δ 7.99 (d, *J* = 7.5 Hz, 2H), 7.66 – 7.50 (m, 3H), 7.43 (t, *J* = 7.7 Hz, 2H), 6.98 (t, *J* = 8.6 Hz, 2H), 5.29 (s, 1H), 4.69 (dd, *J* = 11.6, 5.0 Hz, 1H), 4.55 (dd, *J* = 11.5, 7.6 Hz, 1H), 3.92 – 3.74 (m, 2H), 3.56 – 3.43 (m, 1H).

^{13}C NMR (101 MHz, $CDCl_3$) δ 166.7, 164.4, 161.9, 137.8, 137.7, 133.5, 129.8, 128.6, 121.7, 116.5, 64.5, 62.1, 46.9.

HRMS (ESI) m/z calcd for $C_{16}H_{16}FO_3Se^+$ $[M+H]^+$ = 355.0243, found = 355.0240.

The ee value was 92%, t_R (major) = 10.4 min, t_R (minor) = 11.6 min (Chiralpak IB, λ = 254 nm, 10% *i*-PrOH/Hexane, flow rate = 1 mL/min).

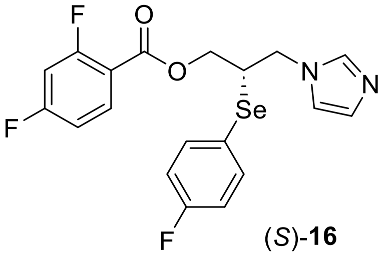

(*S*)-**16**

(*S*)-2-((4-fluorophenyl)selanyl)-3-(1H-imidazol-1-yl)propyl 2,4-difluorobenzoate (16)

37 mg, 85% yield, a pale-yellow oil, R_f = 0.5 (DCM : MeOH = 10:1).

^{1}H NMR (400 MHz, $CDCl_3$) δ 8.01 – 7.90 (m, 1H), 7.65 (s, 1H), 7.60 – 7.45 (m, 2H), 7.08 (s, 1H), 7.05 – 6.86 (m, 4H), 4.53 (dd, *J* = 11.9, 4.5 Hz, 1H), 4.46 – 4.37 (m, 2H), 4.32 (dd, *J* = 14.6, 6.7 Hz, 1H), 3.61 – 3.48 (m, 1H).

^{13}C NMR (151 MHz, $CDCl_3$) δ 165.2, 164.1, 163.7, 163.1, 162.4, 162.0, 137.8, 137.7, 134.2, 129.7, 121.3, 119.2, 116.9, 116.7, 114.3, 112.1, 112.0, 105.6, 105.4, 105.2, 64.8, 48.4, 43.6.

HRMS (ESI) m/z calcd for $C_{19}H_{16}F_3N_2O_2Se^+$ $[M+H]^+$ = 441.0324, found = 441.0326.

The ee value was 92%, t_R (major) = 17.9 min, t_R (minor) = 12.6 min (Chiralpak AD-H, λ = 210 nm, 30% *i*-PrOH/Hexane, flow rate = 1 mL/min).

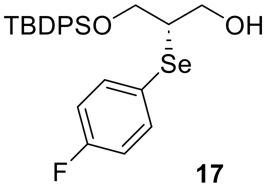

17

(*S*)-3-((*tert*-butyldiphenylsilyl)oxy)-2-((4-fluorophenyl)selanyl)propan-1-ol (17)

317 mg, 65% yield, a colorless oil, R_f = 0.2 (DCM : MeOH = 10:1).

^{1}H NMR (600 MHz, $CDCl_3$) δ 7.66 (dd, *J* = 15.6, 7.4 Hz, 4H), 7.57 – 7.31 (m, 8H), 6.91 (t, *J* = 8.6 Hz, 2H), 4.01 (d, *J* = 6.3 Hz, 1H), 3.98 – 3.79 (m, 3H), 3.35 – 3.24 (m, 1H), 2.42 (s, 1H), 1.07 (s, 9H).

^{13}C NMR (151 MHz, $CDCl_3$) δ 163.5, 161.9, 137.0, 137.0, 135.6, 135.5, 132.9, 132.8, 129.9, 127.8, 122.4, 116.3, 116.2, 65.2, 63.7, 49.7, 26.8, 19.2.

HRMS (ESI) m/z calcd for $C_{25}H_{30}FO_2SeSi^+$ $[M+H]^+$ = 489.1159, found = 489.1155.

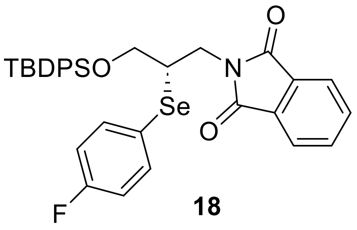

18

(*S*)-2-(3-((*tert*-butyldiphenylsilyl)oxy)-2-((4-fluorophenyl)selanyl)propyl)isoindoline-1,3-dione (18)

164 mg, 53%, a colorless oil, R_f = 0.4 (PE : EtOAc = 10:1).

^{1}H NMR (600 MHz, $CDCl_3$) δ 7.85 – 7.75 (m, 2H), 7.75 – 7.60 (m, 6H), 7.48 – 7.27 (m, 8H), 6.74 (t, *J* = 8.7 Hz, 2H), 4.18 (dd, *J* = 14.2, 6.2 Hz, 1H), 4.07 (dd, *J* = 14.2, 9.0 Hz, 1H), 3.94 – 3.85 (m, 2H), 3.80 – 3.72 (m, 1H), 1.06 (s, 9H).

^{13}C NMR (151 MHz, $CDCl_3$) δ 170.8, 139.0, 139.0, 138.3, 138.3, 138.1, 136.6, 135.7, 135.6, 134.6, 132.4, 132.4, 130.4, 125.9, 125.2, 118.8, 118.6, 68.1, 48.0, 43.5, 29.4, 21.9.

HRMS (ESI) m/z calcd for $C_{33}H_{33}FNO_3SeSi^+$ $[M+H]^+$ = 618.1373, found = 618.1374.

(9) Substrate scope for the synthesis of chiral selenium-based platform molecules

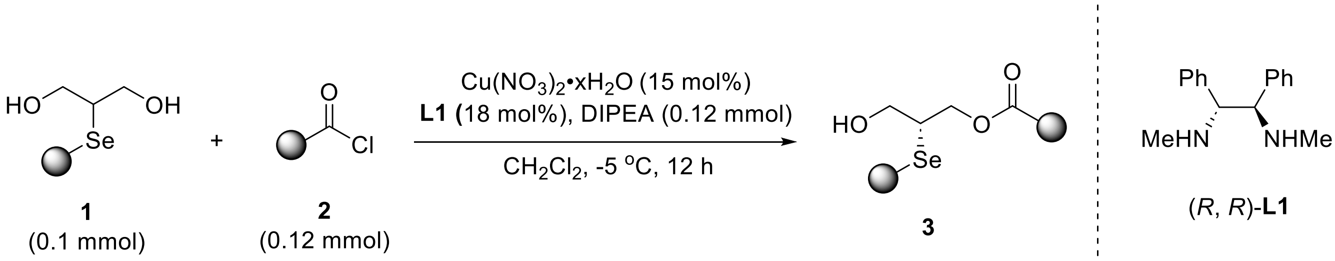

General procedure: $Cu(NO_3)_2 \cdot xH_2O$ (0.015 mmol, 0.15 eq) and **L1** (0.018 mmol, 0.18 eq) were added to DCM (2.0 mL) in a 10-mL tube equipped with a magnetic stirring bar. After stirring for 30 min, **1a** (0.1 mmol, 1.0 eq) was added to the solution and the mixture was cooled to -5 °C. Acyl chloride (0.12 mmol, 1.2 eq) was then added to the mixture, followed by the addition of DIPEA (0.12 mmol, 1.2 eq). Then the mxiture was allowed to stir at -5 °C for 12 hours or till completion. 5 mL H_2O was poured into the reaction and the mixture was extracted with CH_2Cl_2, washed with brine and dried with Na_2SO_4. The combined organic phase was rotary evaporated. The residue was purified by column chromatography on silica to afford the product.

(***The characterization data of 3a and 3aj were presented in the previous section***)

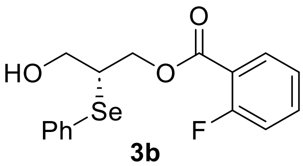

3b

(*R*)-3-hydroxy-2-(phenylselanyl)propyl 2-fluorobenzoate (3b)

33 mg, 94% yield, a colorless foam, R_f = 0.4 (PE : DCM : Acetone = 5:5:0.2).

^{1}H NMR (400 MHz, $CDCl_3$) δ 7.91 (t, *J* = 6.9 Hz, 1H), 7.72 – 7.58 (m, 2H), 7.58 – 7.48 (m, 1H), 7.38 – 7.24 (m, 3H), 7.23 – 7.17 (m, 1H), 7.17 – 7.05 (m, 1H), 4.74 (dd, *J* = 11.1, 4.0 Hz, 1H), 4.63 – 4.43 (m, 1H), 4.05 – 3.74 (m, 2H), 3.56 (s, 1H), 2.56 (s, 1H).

^{13}C NMR (101 MHz, $CDCl_3$) δ 164.4, 163.4, 160.8, 135.2, 135.1, 135.0, 132.4, 129.4, 128.4, 127.2, 124.2, 118.2, 118.1, 117.3, 117.1, 65.1, 62.4, 46.1.

HRMS (ESI) m/z calcd for $C_{16}H_{15}FNaO_3Se^+$ $[M+Na]^+$ = 377.0063, found = 377.0062.

The ee value was 83%, t_R (major) = 15.3 min, t_R (minor) = 17.4 min (Chiralpak IB, λ = 254 nm, 10% *i*-PrOH/Hexane, flow rate = 0.8 mL/min).

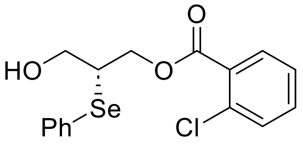

3c

(*R*)-3-hydroxy-2-(phenylselanyl)propyl 2-chlorobenzoate (3c)

33 mg, 88% yield, a colorless foam, R_f = 0.4 (PE : DCM : Acetone = 5:5:0.2).

^{1}H NMR (400 MHz, $CDCl_3$) δ 7.82 (d, *J* = 7.5 Hz, 1H), 7.62 (d, *J* = 6.5 Hz, 2H), 7.49 – 7.37 (m, 2H), 7.37 – 7.19 (m, 4H), 4.73 (dd, *J* = 11.3, 4.5 Hz, 1H), 4.64 – 4.49 (m, 1H), 3.98 – 3.78 (m, 2H), 3.64 – 3.47 (m, 1H), 2.52 (s, 1H).

^{13}C NMR (101 MHz, $CDCl_3$) δ 165.8, 135.2, 133.9, 133.0, 131.8, 131.3, 129.6, 129.4, 128.4, 127.1, 126.8, 65.2, 62.3, 46.2.

HRMS (ESI) m/z calcd for $C_{16}H_{16}ClO_3Se^+$ $[M+H]^+$ = 370.9948, found = 370.9951.

The ee value was 92%, t_R (major) = 15.6 min, t_R (minor) = 18.4 min (Chiralpak IB, λ = 254 nm, 10% *i*-PrOH/Hexane, flow rate = 0.8 mL/min).

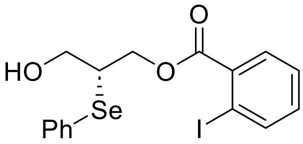

3d

(*R*)-3-hydroxy-2-(phenylselanyl)propyl 2-iodobenzoate (3d)

37 mg, 81% yield, a yellow foam, R_f = 0.5 (PE : DCM : Acetone = 5:5:0.2).

^1H NMR (400 MHz, $CDCl_3$) δ 7.99 (d, *J* = 7.4 Hz, 1H), 7.78 (d, *J* = 7.1 Hz, 1H), 7.62 (d, *J* = 5.8 Hz, 2H), 7.45 – 7.36 (m, 1H), 7.35 – 7.22 (m, 3H), 7.21 – 7.11 (m, 1H), 4.80 – 4.64 (m, 1H), 4.63 – 4.50 (m, 1H), 4.02 – 3.75 (m, 2H), 3.57 (s, 1H), 2.54 (s, 1H).

^{13}C NMR (101 MHz, $CDCl_3$) δ 166.5, 141.5, 135.3, 134.5, 133.1, 131.3, 129.5, 128.4, 128.1, 127.0, 94.4, 65.2, 62.3, 46.2.

HRMS (ESI) m/z calcd for $C_{16}H_{15}INaO_3Se^+$ $[M+Na]^+$ = 484.9123, found = 484.9122.

The ee value was 95%, t_R (major) = 12.6 min, t_R (minor) = 14.1 min (Chiralpak IB, λ = 254 nm, 10% *i*-PrOH/Hexane, flow rate = 0.8 mL/min).

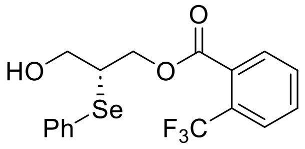

3e

(*R*)-3-hydroxy-2-(phenylselanyl)propyl 2-(trifluoromethyl)benzoate (3e)

34 mg, 84% yield, a yellow foam, R_f = 0.4 (PE : DCM : Acetone = 5:5:0.2).

^1H NMR (400 MHz, $CDCl_3$) δ 7.86 – 7.71 (m, 2H), 7.68 – 7.56 (m, 4H), 7.36 – 7.25 (m, 3H), 4.73 (dd, *J* = 11.5, 5.0 Hz, 1H), 4.56 (dd, *J* = 11.5, 8.1 Hz, 1H), 3.96 – 3.76 (m, 2H), 3.62 – 3.48 (m, 1H), 2.40 (s, 1H).

^{13}C NMR (101 MHz, $CDCl_3$) δ 166.8, 135.3, 131.9, 131.6, 130.7, 130.6, 129.5, 128.4, 127.0, 127.0, 126.9, 126.9, 126.8, 65.6, 62.1, 46.1.

HRMS (ESI) m/z calcd for $C_{17}H_{16}F_3O_3Se^+$ $[M+H]^+$ = 405.0211, found = 405.0215.

The ee value was 95%, t_R (major) = 19.8 min, t_R (minor) = 27.7 min (Chiralpak OJ-H, λ = 254 nm, 10% *i*-PrOH/Hexane, flow rate = 0.8 mL/min).

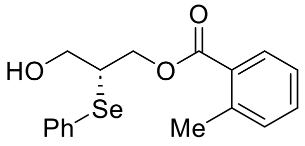

3f

(*R*)-3-hydroxy-2-(phenylselanyl)propyl 2-methylbenzoate (3f)

30 mg, 86% yield, a yellow foam, R_f = 0.5 (PE : DCM : Acetone = 5:5:0.2).

^1H NMR (400 MHz, $CDCl_3$) δ 7.90 (d, *J* = 7.5 Hz, 1H), 7.62 (d, *J* = 6.4 Hz, 2H), 7.41 (t, *J* = 7.3 Hz, 1H), 7.36 – 7.12 (m, 5H), 4.71 (dd, *J* = 11.6, 4.8 Hz, 1H), 4.54 (dd, *J* = 11.6, 7.6 Hz, 1H), 3.96 – 3.73 (m, 2H), 3.63 – 3.46 (m, 1H), 2.60 (s, 3H).

^{13}C NMR (101 MHz, $CDCl_3$) δ 167.6, 140.6, 135.2, 132.5, 131.9, 130.9, 129.4, 129.0, 128.4, 127.3, 125.9, 64.3, 62.3, 46.6, 22.0.

HRMS (ESI) m/z calcd for $C_{17}H_{19}O_3Se^+$ $[M+H]^+$ = 351.0494, found = 351.0494.

The ee value was 94%, t_R (major) = 20.8 min, t_R (minor) = 25.2 min (Chiralpak OJ-H, λ = 254 nm, 10% *i*-PrOH/Hexane, flow rate = 0.8 mL/min).

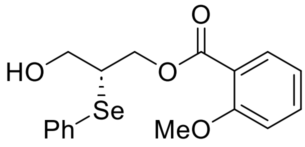

3g

(*R*)-3-hydroxy-2-(phenylselanyl)propyl 2-methoxybenzoate (3g)

31 mg, 85% yield, a yellow oil, R_f = 0.4 (PE : DCM : Acetone = 5:5:0.3).

^{1}H NMR (400 MHz, $CDCl_3$) δ 7.84 (dd, *J* = 7.7, 1.5 Hz, 1H), 7.68 – 7.56 (m, 2H), 7.53 – 7.43 (m, 1H), 7.34 – 7.23 (m, 3H), 7.04 – 6.91 (m, 2H), 4.76 (dd, *J* = 11.5, 4.5 Hz, 1H), 4.48 (dd, *J* = 11.5, 8.4 Hz, 1H), 3.98 – 3.81 (m, 5H), 3.63 – 3.50 (m, 1H), 3.08 (t, *J* = 6.5 Hz, 1H).

^{13}C NMR (101 MHz, $CDCl_3$) δ 166.2, 159.0, 135.0, 134.1, 132.2, 129.2, 128.1, 127.1, 120.3, 119.0, 111.9, 65.8, 63.6, 55.8, 45.2.

HRMS (ESI) m/z calcd for $C_{17}H_{18}NaO_4Se^+$ $[M+Na]^+$ = 389.0263, found = 389.0261.

The ee value was 87%, t_R (major) = 13.5 min, t_R (minor) = 15.1 min (Chiralpak IB, λ = 254 nm, 20% *i*-PrOH/Hexane, flow rate = 0.8 mL/min).

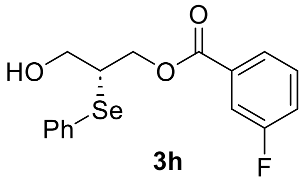

3h

(*R*)-3-hydroxy-2-(phenylselanyl)propyl 3-fluorobenzoate (3h)

25 mg, 70% yield, a colorless oil, R_f = 0.4 (PE : DCM : Acetone = 5:5:0.2).

^{1}H NMR (400 MHz, $CDCl_3$) δ 7.80 (d, *J* = 7.5 Hz, 1H), 7.71 – 7.57 (m, 3H), 7.42 (q, *J* = 7.6 Hz, 1H), 7.36 – 7.23 (m, 4H), 4.72 (dd, *J* = 11.4, 4.9 Hz, 1H), 4.62 – 4.51 (m, 1H), 3.94 – 3.77 (m, 2H), 3.63 – 3.47 (m, 1H), 2.47 (s, 1H).

^{13}C NMR (101 MHz, $CDCl_3$) δ 163.9, 161.4, 135.3, 130.3, 130.2, 129.5, 128.5, 127.1, 125.6, 120.6, 120.4, 116.8, 116.6, 64.9, 62.2, 46.4.

HRMS (ESI) m/z calcd for $C_{16}H_{16}FO_3Se^+$ $[M+H]^+$ = 355.0243, found = 355.0240.

The ee value was 94%, t_R (major) = 12.6 min, t_R (minor) = 14.1 min (Chiralpak IB, λ = 254 nm, 10% *i*-PrOH/Hexane, flow rate = 0.8 mL/min).

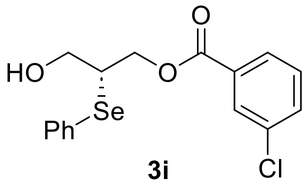

3i

(*R*)-3-hydroxy-2-(phenylselanyl)propyl 3-chlorobenzoate (3i)

35 mg, 95% yield, a colorless oil, R_f = 0.5 (PE : DCM : Acetone = 5:5:0.2).

^{1}H NMR (400 MHz, $CDCl_3$) δ 7.96 (s, 1H), 7.88 (d, *J* = 7.6 Hz, 1H), 7.62 (d, *J* = 6.5 Hz, 2H), 7.54 (d, *J* = 7.8 Hz, 1H), 7.37 (t, *J* = 7.9 Hz, 1H), 7.31 (s, 3H), 4.76 – 4.67 (m, 1H), 4.62 – 4.53 (m, 1H), 3.94 – 3.78 (m, 2H), 3.61 – 3.51 (m, 1H), 2.53 (s, 1H).

^{13}C NMR (101 MHz, $CDCl_3$) δ 165.5, 135.2, 134.7, 133.4, 133.3, 131.4, 129.9, 129.8, 129.5, 128.4, 128.0, 65.0, 62.2, 46.3.

HRMS (ESI) m/z calcd for $C_{16}H_{16}ClO_3Se^+$ $[M+H]^+$ = 370.9948, found = 370.9950.

The ee value was 91%, t_R (major) = 8.4 min, t_R (minor) = 9.0 min (Chiralpak OJ-H, λ = 254 nm, 30% *i*-PrOH/Hexane, flow rate = 0.8 mL/min).

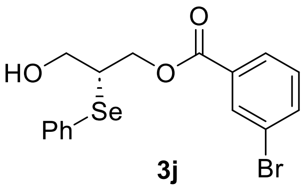

3j

(*R*)-3-hydroxy-2-(phenylselanyl)propyl 3-bromobenzoate (3j)

34 mg, 83% yield, a colorless oil, R_f = 0.4 (PE : DCM : Acetone = 5:5:0.3).

^{1}H NMR (400 MHz, $CDCl_3$) δ 8.12 (s, 1H), 7.93 (d, *J* = 7.6 Hz, 1H), 7.69 (d, *J* = 7.8 Hz, 1H), 7.62 (d, *J* = 6.5 Hz, 2H), 7.36 – 7.21 (m, 4H), 4.71 (dd, *J* = 11.5, 5.1 Hz, 1H), 4.58 (dd, *J* = 11.4, 7.8 Hz, 1H), 3.94 – 3.76 (m, 2H), 3.64 – 3.49 (m, 1H), 2.47 (s, 1H).

^{13}C NMR (101 MHz, $CDCl_3$) δ 165.4, 136.3, 135.2, 132.8, 131.7, 130.1, 129.5, 128.4, 128.4, 127.2, 122.7, 65.0, 62.2, 46.4.

HRMS (ESI) m/z calcd for $C_{16}H_{16}BrO_3Se^+$ $[M+H]^+$ = 414.9443, found = 414.9445.

The ee value was 88%, t_R (major) = 8.3 min, t_R (minor) = 9.0 min (Chiralpak OJ-H, λ = 254 nm, 30% *i*-PrOH/Hexane, flow rate = 0.8 mL/min).

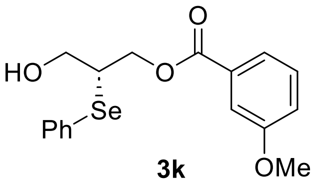

3k

(*R*)-3-hydroxy-2-(phenylselanyl)propyl 3-methoxybenzoate (3k)

30 mg, 82% yield, a colorless oil, R_f = 0.4 (PE : DCM : Acetone = 5:5:0.4).

^{1}H NMR (400 MHz, $CDCl_3$) δ 7.61 (t, *J* = 8.4 Hz, 3H), 7.54 (s, 1H), 7.40 – 7.22 (m, 4H), 7.11 (dd, *J* = 8.2, 3.0 Hz, 1H), 4.71 (dd, *J* = 11.6, 4.9 Hz, 1H), 4.56 (dd, *J* = 11.5, 7.9 Hz, 1H), 3.89 (s, 1H), 3.84 (s, 3H), 3.56 (dd, *J* = 7.9, 5.3 Hz, 1H), 2.59 (t, *J* = 6.4 Hz, 1H).

^{13}C NMR (101 MHz, $CDCl_3$) δ 166.6, 159.7, 135.2, 131.0, 129.6, 129.5, 128.4, 127.3, 122.2, 119.9, 114.3, 64.6, 62.2, 55.6, 46.5.

^{13}C NMR (101 MHz, $CDCl_3$) δ 166.8, 136.5, 134.8, 133.5, 129.8, 129.6, 128.6, 125.5, 64.5, 62.2, 46.8.
HRMS (ESI) m/z calcd for $C_{17}H_{19}O_4Se^+$ $[M+H]^+$ = 367.0443, found = 367.0442.
The ee value was 93%, t_R (major) = 11.1 min, t_R (minor) = 13.6 min (Chiralpak OJ-H, λ = 254 nm, 30% *i*-PrOH/Hexane, flow rate = 0.8 mL/min).

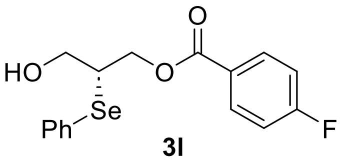

3l

(*R*)-3-hydroxy-2-(phenylselanyl)propyl 4-fluorobenzoate (3l)

25 mg, 72% yield, a colorless oil, R_f = 0.3 (PE : DCM : Acetone = 5:5:0.2).
^1H NMR (400 MHz, $CDCl_3$) δ 8.15 – 7.87 (m, 2H), 7.62 (d, *J* = 6.6 Hz, 2H), 7.30 (d, *J* = 7.2 Hz, 3H), 7.10 (t, *J* = 8.4 Hz, 2H), 4.71 (dd, *J* = 11.5, 4.9 Hz, 1H), 4.56 (dd, *J* = 11.3, 7.8 Hz, 1H), 3.93 – 3.74 (m, 2H), 3.62 – 3.46 (m, 1H), 2.56 (s, 1H).
^{13}C NMR (101 MHz, $CDCl_3$) δ 167.2, 165.6, 164.6, 135.0, 132.3, 132.2, 129.3, 128.2, 127.1, 125.8, 115.7, 115.5, 64.5, 62.0, 46.3.
HRMS (ESI) m/z calcd for $C_{16}H_{15}FNaO_3Se^+$ $[M+Na]^+$ = 377.0063, found = 377.0061.
The ee value was 96%, t_R (major) = 13.0 min, t_R (minor) = 14.1 min (Chiralpak IB, λ = 254 nm, 10% *i*-PrOH/Hexane, flow rate = 0.8 mL/min).

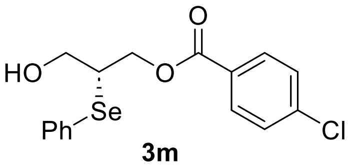

3m

(*R*)-3-hydroxy-2-(phenylselanyl)propyl 4-chlorobenzoate (3m)

30 mg, 82% yield, a colorless foam, R_f = 0.4 (PE : DCM : Acetone = 5:5:0.2).
^1H NMR (400 MHz, $CDCl_3$) δ 7.92 (d, *J* = 8.2 Hz, 2H), 7.62 (d, *J* = 6.7 Hz, 2H), 7.41 (d, *J* = 8.3 Hz, 2H), 7.35 – 7.26 (m, 3H), 4.71 (dd, *J* = 11.5, 5.0 Hz, 1H), 4.57 (dd, *J* = 11.3, 7.8 Hz, 1H), 3.93 – 3.73 (m, 2H), 3.63 – 3.47 (m, 1H), 2.51 (s, 1H).
^{13}C NMR (101 MHz, $CDCl_3$) δ 165.9, 139.9, 135.2, 131.2, 129.5, 128.9, 128.4, 128.2, 127.2, 64.8, 62.2, 46.4.
HRMS (ESI) m/z calcd for $C_{16}H_{15}ClNaO_3Se^+$ $[M+Na]^+$ = 392.9767, found = 392.9768.
The ee value was 94%, t_R (major) = 11.4 min, t_R (minor) = 13.4 min (Chiralpak IA, λ = 254 nm, 15% *i*-PrOH/Hexane, flow rate = 0.8 mL/min).

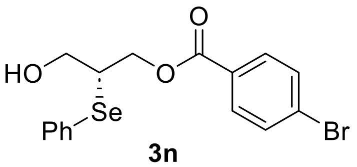

3n

(*R*)-3-hydroxy-2-(phenylselanyl)propyl 4-bromobenzoate (3n)

27 mg, 65% yield, a yellow oil, R_f = 0.3 (PE : DCM : Acetone = 5:5:0.2).

^{1}H NMR (400 MHz, $CDCl_3$) δ 7.85 (d, *J* = 8.4 Hz, 2H), 7.62 (d, *J* = 7.5 Hz, 2H), 7.57 (d, *J* = 8.4 Hz, 2H), 7.37 – 7.26 (m, 3H), 4.71 (dd, *J* = 11.6, 5.1 Hz, 1H), 4.57 (dd, *J* = 11.5, 7.7 Hz, 1H), 3.91 – 3.77 (m, 2H), 3.56 (dt, *J* = 11.1, 5.5 Hz, 1H), 2.33 (s, 1H).

^{13}C NMR (101 MHz, $CDCl_3$) δ 165.8, 135.1, 131.8, 131.2, 129.3, 128.5, 128.4, 128.3, 127.1, 64.7, 62.1, 46.3.

HRMS (ESI) m/z calcd for $C_{16}H_{15}BrNaO_3Se^+$ $[M+Na]^+$ = 436.9262, found = 436.9264.

The ee value was 83%, t_R (major) = 13.7 min, t_R (minor) = 17.9 min (Chiralpak IA, λ = 254 nm, 10% *i*-PrOH/Hexane, flow rate = 1 mL/min).

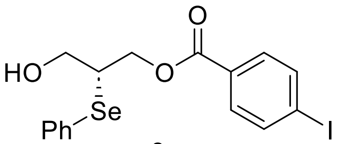

3o

(*R*)-3-hydroxy-2-(phenylselanyl)propyl 4-iodobenzoate (3o)

37 mg, 81% yield, a pale-yellow oil, R_f = 0.3 (PE : DCM : Acetone = 5:5:0.4).

^{1}H NMR (400 MHz, $CDCl_3$) δ 7.78 (d, *J* = 7.6 Hz, 2H), 7.68 (d, *J* = 7.6 Hz, 2H), 7.61 (d, *J* = 5.9 Hz, 2H), 7.29 (d, *J* = 6.6 Hz, 2H), 4.69 (dd, *J* = 10.8, 4.2 Hz, 1H), 4.61 – 4.50 (m, 1H), 3.83 (s, 2H), 3.55 (s, 1H), 2.53 (s, 1H).

^{13}C NMR (101 MHz, $CDCl_3$) δ 166.2, 137.9, 135.2, 131.2, 129.5, 129.2, 128.4, 127.2, 101.3, 64.8, 62.2, 46.4.

HRMS (ESI) m/z calcd for $C_{16}H_{15}INaO_3Se^+$ $[M+Na]^+$ = 484.9123, found = 484.9126.

The ee value was 93%, t_R (major) = 22.0 min, t_R (minor) = 19.1 min (Chiralpak IB, λ = 254 nm, 10% *i*-PrOH/Hexane, flow rate = 1 mL/min).

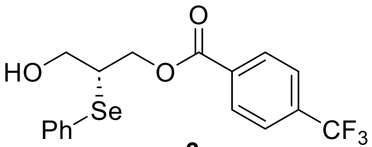

3p

(*R*)-3-hydroxy-2-(phenylselanyl)propyl 4-(trifluoromethyl)benzoate (3p)

29 mg, 73% yield, a yellow foam, R_f = 0.2 (PE : DCM : Acetone = 5:5:0.3).

^{1}H NMR (400 MHz, $CDCl_3$) δ 8.11 (d, *J* = 8.0 Hz, 2H), 7.70 (d, *J* = 8.1 Hz, 2H), 7.62 (d, *J* = 7.2 Hz, 2H), 7.37 – 7.24 (m, 3H), 4.74 (dd, *J* = 11.5, 5.2 Hz, 1H), 4.61 (dd, *J* = 11.4, 7.7 Hz, 1H), 3.87 (s, 2H), 3.64 – 3.52 (m, 1H), 2.45 (s, 1H).

^{13}C NMR (101 MHz, $CDCl_3$) δ 165.3, 135.1, 132.9, 130.1, 129.4, 128.3, 127.0, 125.5, 125.4, 125.4, 124.9, 122.2, 65.0, 62.1, 46.2.

HRMS (ESI) m/z calcd for $C_{17}H_{16}F_3O_3Se^+$ $[M+H]^+$ = 405.0211, found = 405.0212.

The ee value was 93%, t_R (major) = 10.5 min, t_R (minor) = 11.3 min (Chiralpak IB, λ = 254 nm, 10% *i*-PrOH/Hexane, flow rate = 1 mL/min).

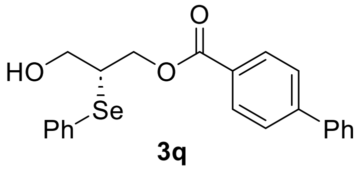

3q

(*R*)-3-hydroxy-2-(phenylselanyl)propyl [1,1'-biphenyl]-4-carboxylate (3q)

21 mg, 51% yield, a colorless foam, R_f = 0.2 (PE : DCM : Acetone = 5:5:0.3).

^{1}H NMR (400 MHz, $CDCl_3$) δ 8.08 (d, *J* = 7.9 Hz, 2H), 7.76 – 7.54 (m, 6H), 7.48 (t, *J* = 7.1 Hz, 2H), 7.41 (d, *J* = 6.9 Hz, 1H), 7.31 (d, *J* = 6.2 Hz, 3H), 4.76 (dd, *J* = 11.4, 4.5 Hz, 1H), 4.67 – 4.50 (m, 1H), 3.97 – 3.76 (m, 2H), 3.72 – 3.50 (m, 1H), 2.55 (s, 1H).

^{13}C NMR (101 MHz, $CDCl_3$) δ 166.7, 146.2, 140.0, 135.2, 130.4, 129.5, 129.1, 128.4, 127.4, 127.3, 64.6, 62.2, 46.6.

HRMS (ESI) m/z calcd for $C_{22}H_{21}O_3Se^+$ $[M+H]^+$ = 413.0650, found = 413.0652.

The ee value was 82%, t_R (major) = 11.5 min, t_R (minor) = 16.1 min (Chiralpak IB, λ = 254 nm, 20% *i*-PrOH/Hexane, flow rate = 1 mL/min).

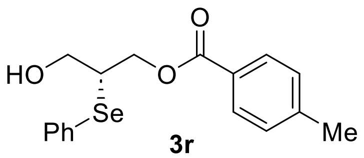

3r

(*R*)-3-hydroxy-2-(phenylselanyl)propyl 4-methylbenzoate (3r)

24 mg, 70% yield, a colorless oil, R_f = 0.5 (PE : DCM : Acetone = 5:5:0.3).

^{1}H NMR (400 MHz, $CDCl_3$) δ 7.90 (d, *J* = 8.1 Hz, 2H), 7.62 (d, *J* = 7.7 Hz, 2H), 7.30 (d, *J* = 7.3 Hz, 3H), 7.27 – 7.20 (m, 2H), 4.71 (dd, *J* = 11.6, 4.9 Hz, 1H), 4.55 (dd, *J* = 11.6, 7.8 Hz, 1H), 3.94 – 3.76 (m, 2H), 3.66 – 3.47 (m, 1H), 2.66 (s, 1H), 2.41 (s, 3H).

^{13}C NMR (101 MHz, $CDCl_3$) δ 166.7, 144.0, 135.0, 129.7, 129.3, 129.1, 128.2, 127.2, 126.8, 64.2, 62.0, 46.4, 21.7.

HRMS (ESI) m/z calcd for $C_{17}H_{19}O_3Se^+$ $[M+H]^+$ = 351.0494, found = 351.0498.

The ee value was 95%, t_R (major) = 15.8 min, t_R (minor) = 19.8 min (Chiralpak IA, λ = 254 nm, 10% *i*-PrOH/Hexane, flow rate = 0.8 mL/min).

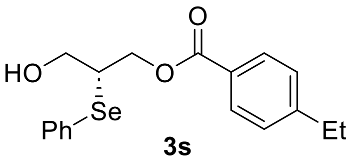

3s

(*R*)-3-hydroxy-2-(phenylselanyl)propyl 4-ethylbenzoate (3s)

33 mg, 90% yield, a colorless oil, R_f = 0.5 (PE : DCM : Acetone = 5:5:0.3).

^{1}H NMR (400 MHz, $CDCl_3$) δ 7.93 (d, *J* = 7.9 Hz, 2H), 7.63 (d, *J* = 6.4 Hz, 2H), 7.38 – 7.17 (m, 5H), 4.72 (dd, *J* = 11.5, 4.7 Hz, 1H), 4.55 (dd, *J* = 11.3, 8.0 Hz, 1H), 3.97 – 3.71 (m, 2H), 3.65 – 3.48 (m, 1H), 2.71 (q, *J* = 7.5 Hz, 2H), 2.64 (t, *J* = 6.3 Hz, 1H), 1.25 (t, *J* = 7.5 Hz, 3H).

^{13}C NMR (101 MHz, $CDCl_3$) δ 166.9, 150.4, 135.2, 130.0, 129.4, 128.3, 128.1, 127.4, 127.1, 64.3, 62.2, 46.6, 29.1, 15.4.

HRMS (ESI) m/z calcd for $C_{18}H_{21}O_3Se^+$ $[M+H]^+$ = 365.0650, found = 365.0653.

The ee value was 94%, t_R (major) = 11.5 min, t_R (minor) = 13.8 min (Chiralpak IA, λ = 254 nm, 15% *i*-PrOH/Hexane, flow rate = 0.8 mL/min).

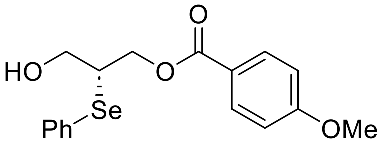

3t

(*R*)-3-hydroxy-2-(phenylselanyl)propyl 4-methoxybenzoate (3t)

31 mg, 84% yield, a pale-yellow oil, R_f = 0.5 (PE : DCM : Acetone = 5:5:0.5).

^{1}H NMR (400 MHz, $CDCl_3$) δ 7.96 (d, *J* = 8.7 Hz, 2H), 7.62 (d, *J* = 7.2 Hz, 2H), 7.34 – 7.24 (m, 3H), 6.91 (d, *J* = 8.7 Hz, 2H), 4.71 (dd, *J* = 11.6, 4.8 Hz, 1H), 4.54 (dd, *J* = 11.5, 7.8 Hz, 1H), 3.86 (s, 3H), 3.82 (d, *J* = 6.1 Hz, 2H), 3.62 – 3.49 (m, 1H), 2.65 (t, *J* = 5.8 Hz, 1H).

^{13}C NMR (101 MHz, $CDCl_3$) δ 166.5, 163.8, 135.1, 131.9, 129.4, 128.3, 127.4, 122.0, 113.8, 64.2, 62.2, 55.6, 46.7.

HRMS (ESI) m/z calcd for $C_{17}H_{19}O_4Se^+$ $[M+H]^+$ = 367.0443, found = 367.0444.

The ee value was 96%, t_R (major) = 15.8 min, t_R (minor) = 21.5 min (Chiralpak IA, λ = 254 nm, 15% *i*-PrOH/Hexane, flow rate = 0.8 mL/min).

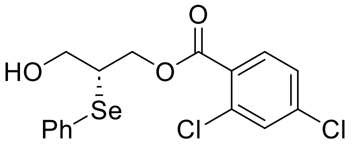

3u

(*R*)-3-hydroxy-2-(phenylselanyl)propyl 2,4-dichlorobenzoate (3u)

27 mg, 68% yield, a pale-yellow oil, R_f = 0.5 (PE : DCM : Acetone = 5:3:0.5).

^{1}H NMR (400 MHz, $CDCl_3$) δ 7.79 (d, *J* = 8.4 Hz, 1H), 7.62 (d, *J* = 7.0 Hz, 2H), 7.48 (s, 1H), 7.38 – 7.22 (m, 4H), 4.73 (dd, *J* = 11.5, 4.8 Hz, 1H), 4.56 (dd, *J* = 11.3, 8.0 Hz, 1H), 3.99 – 3.72 (m, 2H), 3.63 – 3.47 (m, 1H), 2.32 (s, 1H).

^{13}C NMR (101 MHz, $CDCl_3$) δ 164.8, 138.9, 135.3, 132.9, 131.3, 129.5, 128.5, 127.9, 127.2, 127.1, 65.4, 62.4, 46.3.

HRMS (ESI) m/z calcd for $C_{16}H_{15}Cl_2O_3Se^+$ $[M+H]^+$ = 404.9558, found = 404.9559.

The ee value was 86%, t_R (major) = 15.8 min, t_R (minor) = 18.0 min (Chiralpak IB, λ = 254 nm, 10% *i*-PrOH/Hexane, flow rate = 0.8 mL/min).

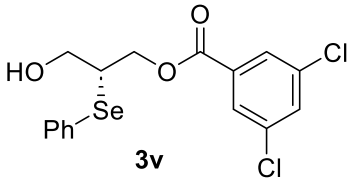

3v

(*R*)-3-hydroxy-2-(phenylselanyl)propyl 3,5-dichlorobenzoate (3v)

21 mg, 52% yield, a pale-yellow foam, R_f = 0.5 (PE : DCM : Acetone = 5:4:0.3).

^{1}H NMR (400 MHz, $CDCl_3$) δ 7.84 (s, 2H), 7.70 – 7.47 (m, 3H), 7.39 – 7.27 (m, 3H), 4.71 (dd, *J* = 11.3, 5.0 Hz, 1H), 4.65 – 4.52 (m, 1H), 3.96 – 3.73 (m, 2H), 3.63 – 3.49 (m, 1H), 2.28 (s, 1H).

^{13}C NMR (101 MHz, $CDCl_3$) δ 164.4, 135.5, 135.3, 133.2, 132.6, 129.5, 128.5, 128.2, 127.0, 65.5, 62.2, 46.3.

HRMS (ESI) m/z calcd for $C_{16}H_{15}Cl_2O_3Se^+$ $[M+H]^+$ = 404.9558, found = 404.9557.

The ee value was 79%, t_R (major) = 21.2 min, t_R (minor) = 23.1 min (Chiralpak IB, λ = 254 nm, 5% *i*-PrOH/Hexane, flow rate = 0.8 mL/min).

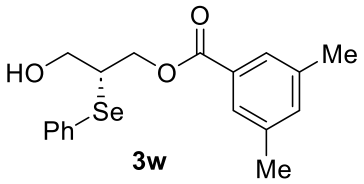

3w

(*R*)-3-hydroxy-2-(phenylselanyl)propyl 3,5-dimethylbenzoate (3w)

23 mg, 63% yield, a colorless oil, R_f = 0.5 (PE : DCM : Acetone = 5:5:0.3).

^{1}H NMR (400 MHz, $CDCl_3$) δ 7.63 (d, *J* = 6.5 Hz, 4H), 7.35 – 7.25 (m, 3H), 7.20 (s, 1H), 4.71 (dd, *J* = 11.6, 4.9 Hz, 1H), 4.55 (dd, *J* = 11.5, 8.0 Hz, 1H), 3.95 – 3.76 (m, 2H), 3.63 – 3.50 (m, 1H), 2.35 (s, 6H).

^{13}C NMR (101 MHz, $CDCl_3$) δ 167.2, 138.2, 135.1, 129.6, 129.4, 128.3, 127.6, 127.5, 127.4, 64.5, 62.2, 46.6, 21.3.

HRMS (ESI) m/z calcd for $C_{18}H_{21}O_3Se^+$ $[M+H]^+$ = 365.0650, found = 365.0653.

The ee value was 96%, t_R (major) = 10.4 min, t_R (minor) = 11.3 min (Chiralpak IB, λ = 254 nm, 10% *i*-PrOH/Hexane, flow rate = 0.8 mL/min).

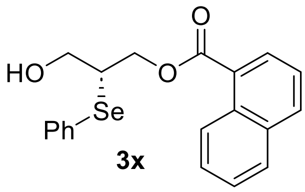

3x

(*R*)-3-hydroxy-2-(phenylselanyl)propyl 1-naphthoate (3x)

28 mg, 72% yield, a colorless oil, R_f = 0.4 (PE : DCM : Acetone = 5:3:0.2).

^{1}H NMR (400 MHz, $CDCl_3$) δ 8.92 (d, *J* = 8.6 Hz, 1H), 8.18 (d, *J* = 7.1 Hz, 1H), 8.04 (d, *J* = 8.1 Hz, 1H), 7.89 (d, *J* = 8.0 Hz, 1H), 7.72 – 7.59 (m, 3H), 7.58 – 7.46 (m, 2H), 7.30 (d, *J* = 6.9 Hz, 3H), 4.82 (dd, *J* = 11.5, 4.8 Hz, 1H), 4.67 (dd, *J* = 11.2, 7.7 Hz, 1H), 3.99 – 3.81 (m, 2H), 3.75 – 3.55 (m, 1H), 2.62 (s, 1H).

^{13}C NMR (101 MHz, $CDCl_3$) δ 167.6, 135.3, 133.9, 131.5, 130.7, 128.7, 128.4, 128.1, 127.3, 126.4, 125.8, 124.6, 64.6, 62.4, 46.6.

HRMS (ESI) m/z calcd for $C_{20}H_{19}O_3Se^+$ $[M+H]^+$ = 387.0494, found = 387.0494.

The ee value was 95%, t_R (major) = 8.7 min, t_R (minor) = 9.9 min (Chiralpak IB, λ = 254 nm, 20% *i*-PrOH/Hexane, flow rate = 1 mL/min).

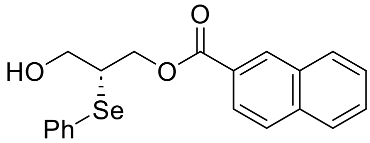

3y

(*R*)-3-hydroxy-2-(phenylselanyl)propyl 2-naphthoate (3y)

27 mg, 70% yield, a colorless oil, R_f = 0.4 (PE : DCM : Acetone = 5:3:0.2).

^{1}H NMR (400 MHz, $CDCl_3$) δ 8.57 (s, 1H), 8.10 – 7.79 (m, 4H), 7.70 – 7.48 (m, 4H), 7.31 (s, 3H), 4.86 – 4.70 (m, 1H), 4.70 – 4.52 (m, 1H), 4.00 – 3.79 (m, 2H), 3.62 (s, 1H), 2.55 (s, 1H).

^{13}C NMR (101 MHz, $CDCl_3$) δ 167.0, 135.8, 135.3, 132.6, 131.5, 129.5, 129.5, 128.6, 128.4, 127.9, 127.3, 126.9, 125.3, 64.7, 62.3, 46.7.

HRMS (ESI) m/z calcd for $C_{20}H_{19}O_3Se^+$ $[M+H]^+$ = 387.0494, found = 387.0491.

The ee value was 93%, t_R (major) = 17.4 min, t_R (minor) = 21.2 min (Chiralpak IA, λ = 254 nm, 10% *i*-PrOH/Hexane, flow rate = 1 mL/min).

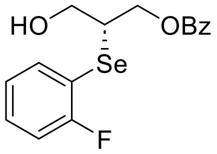

3z

(*R*)-2-((2-fluorophenyl)selanyl)-3-hydroxypropyl benzoate (3z)

26 mg, 75% yield, a colorless oil, R_f = 0.3 (PE : DCM : Acetone = 5:4:0.4).

^{1}H NMR (400 MHz, $CDCl_3$) δ 8.01 (d, *J* = 7.4 Hz, 2H), 7.58 (t, *J* = 7.0 Hz, 1H), 7.52 – 7.30 (m, 4H), 7.30 – 7.20 (m, 1H), 7.00 (t, *J* = 7.8 Hz, 1H), 4.74 (dd, *J* = 11.4, 4.6 Hz, 1H), 4.67 – 4.53 (m, 1H), 3.89 (s, 2H), 3.61 (s, 1H), 2.58 (s, 1H).

^{13}C NMR (101 MHz, $CDCl_3$) δ 166.6, 163.7, 161.2, 133.3, 130.6, 130.5, 130.1, 130.1, 129.7, 129.4, 129.2, 129.1, 128.4, 121.4, 121.2, 115.3, 115.0, 64.3, 62.1, 46.6.

HRMS (ESI) m/z calcd for $C_{16}H_{16}FO_3Se^+$ $[M+H]^+$ = 355.0243, found = 355.0244.

The ee value was 96%, t_R (major) = 10.8 min, t_R (minor) = 12.6 min (Chiralpak IB, λ = 254 nm, 10% *i*-PrOH/Hexane, flow rate = 1 mL/min).

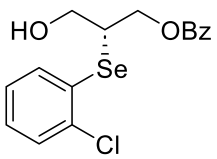

3aa

(*R*)-2-((2-chlorophenyl)selanyl)-3-hydroxypropyl benzoate (3aa)

19 mg, 52% yield, a colorless oil, R_f = 0.5 (PE : DCM : Acetone = 5:5:0.4).

^{1}H NMR (400 MHz, $CDCl_3$) δ 8.03 (d, *J* = 7.4 Hz, 2H), 7.70 – 7.54 (m, 2H), 7.50 – 7.37 (m, 3H), 7.25 – 7.10 (m, 2H), 4.79 (dd, *J* = 11.4, 4.2 Hz, 1H), 4.71 – 4.56 (m, 1H), 3.92 (s, 2H), 3.73 (s, 1H), 2.58 (s, 1H).

^{13}C NMR (101 MHz, $CDCl_3$) δ 166.7, 137.4, 134.7, 133.3, 129.9, 129.7, 129.5, 129.1, 128.5, 127.5, 64.2, 62.1, 45.5.

HRMS (ESI) m/z calcd for $C_{16}H_{16}ClO_3Se^+$ $[M+H]^+$ = 370.9948, found = 370.9949.

The ee value was 95%, t_R (major) = 17.3 min, t_R (minor) = 14.9 min (Chiralpak IC, λ = 254 nm, 10% *i*-PrOH/Hexane, flow rate = 1 mL/min).

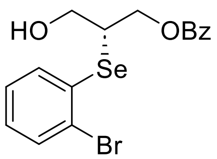

3ab

(*R*)-2-((2-bromophenyl)selanyl)-3-hydroxypropyl benzoate (3ab)

25 mg, 61% yield, a colorless oil, R_f = 0.3 (PE : DCM : Acetone = 5:5:0.4).

^{1}H NMR (400 MHz, $CDCl_3$) δ 8.07 – 7.98 (m, 2H), 7.69 – 7.53 (m, 3H), 7.45 (t, *J* = 7.7 Hz, 2H), 7.24 (dd, *J* = 7.7, 1.1 Hz, 1H), 7.19 – 7.10 (m, 1H), 4.81 (dd, *J* = 11.7, 4.7 Hz, 1H), 4.65 (dd, *J* = 11.6, 7.7 Hz, 1H), 3.93 (s, 2H), 3.81 – 3.66 (m, 1H), 2.63 (s, 1H).

^{13}C NMR (101 MHz, $CDCl_3$) δ 166.7, 134.2, 133.3, 133.2, 131.1, 129.7, 129.5, 129.0, 128.5, 128.1, 64.1, 62.1, 45.9.

HRMS (ESI) m/z calcd for $C_{16}H_{16}BrO_3Se^+$ $[M+H]^+$ = 414.9443, found = 414.9446.

The ee value was 93%, t_R (major) = 19.5 min, t_R (minor) = 15.5 min (Chiralpak IC, λ = 254 nm, 10% *i*-PrOH/Hexane, flow rate = 1 mL/min).

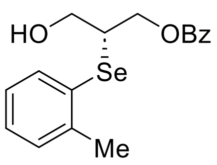

3ac

(*R*)-3-hydroxy-2-(o-tolylselanyl)propyl benzoate (3ac)

26 mg, 75% yield, a colorless oil, R_f = 0.5 (PE : DCM : Acetone = 5:5:0.2).

^{1}H NMR (400 MHz, $CDCl_3$) δ 8.02 (d, *J* = 7.3 Hz, 2H), 7.65 – 7.53 (m, 2H), 7.44 (t, *J* = 7.7 Hz, 2H), 7.25 – 7.17 (m, 2H), 7.17 – 7.08 (m, 1H), 4.73 (dd, *J* = 11.6, 4.9 Hz, 1H), 4.60 (dd, *J* = 11.6, 7.7 Hz, 1H), 3.88 (d, *J* = 5.4 Hz, 2H), 3.61 – 3.52 (m, 1H), 2.50 (s, 3H).

^{13}C NMR (101 MHz, $CDCl_3$) δ 166.7, 141.1, 134.9, 133.3, 130.3, 129.7, 129.6, 128.7, 128.4, 128.3, 126.7, 64.4, 62.2, 45.7, 23.0.

HRMS (ESI) m/z calcd for $C_{17}H_{19}O_3Se^+$ $[M+H]^+$ = 351.0494, found = 351.0497.

The ee value was 95%, t_R (major) = 10.6 min, t_R (minor) = 12.2 min (Chiralpak IB, λ = 254 nm, 10% *i*-PrOH/Hexane, flow rate = 1 mL/min).

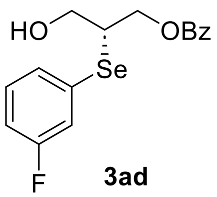

3ad

(*R*)-2-((3-fluorophenyl)selanyl)-3-hydroxypropyl benzoate (3ad)

20 mg, 52% yield, a colorless oil, R_f = 0.4 (PE : DCM : Acetone = 5:3:0.3).

^{1}H NMR (400 MHz, $CDCl_3$) δ 8.01 (d, *J* = 7.5 Hz, 2H), 7.70 – 7.51 (m, 2H), 7.50 – 7.28 (m, 3H), 7.10 (q, *J* = 8.2 Hz, 2H), 4.73 (dd, *J* = 11.4, 4.6 Hz, 1H), 4.64 – 4.51 (m, 1H), 3.95 – 3.73 (m, 2H), 3.72 – 3.56 (m, 1H), 2.58 (s, 1H).

^{13}C NMR (101 MHz, $CDCl_3$) δ 166.6, 163.9, 137.2, 137.2, 133.3, 130.8, 130.7, 129.7, 129.5, 128.4, 124.9, 124.9, 116.0, 115.7, 64.4, 62.2, 45.8.

HRMS (ESI) m/z calcd for $C_{16}H_{16}FO_3Se^+$ $[M+H]^+$ = 355.0243, found = 355.0240.

The ee value was 94%, t_R (major) = 10.8 min, t_R (minor) = 12.3 min (Chiralpak IB, λ = 254 nm, 10% *i*-PrOH/Hexane, flow rate = 1 mL/min).

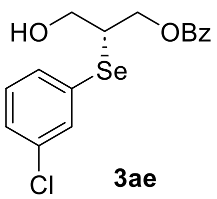

3ae

(*R*)-2-((3-chlorophenyl)selanyl)-3-hydroxypropyl benzoate (3ae)

25 mg, 68% yield, a yellow foam, R_f = 0.5 (PE : DCM : Acetone = 5:3:0.3).

^{1}H NMR (400 MHz, $CDCl_3$) δ 8.02 (d, *J* = 7.2 Hz, 2H), 7.70 – 7.55 (m, 2H), 7.54 – 7.35 (m, 3H), 7.34 – 7.21 (m, 2H), 4.84 – 4.57 (m, 2H), 3.88 (s, 2H), 3.59 (s, 1H), 2.58 (s, 1H).
^{13}C NMR (101 MHz, $CDCl_3$) δ 166.6, 134.7, 134.2, 133.3, 132.6, 130.3, 129.7, 129.4, 129.1, 128.5, 128.3, 64.3, 62.1, 46.7.
HRMS (ESI) m/z calcd for $C_{16}H_{16}ClO_3Se^+$ $[M+H]^+$ = 370.9948, found = 370.9945.
The ee value was 95%, t_R (major) = 10.9 min, t_R (minor) = 12.7 min (Chiralpak IB, λ = 254 nm, 10% *i*-PrOH/Hexane, flow rate = 1 mL/min).

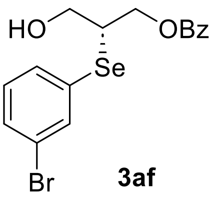

3af

(*R*)-2-((3-bromophenyl)selanyl)-3-hydroxypropyl benzoate (3af)
27 mg, 64% yield, a yellow oil, R_f = 0.3 (PE : DCM : Acetone = 5:5:0.3).
^{1}H NMR (400 MHz, $CDCl_3$) δ 8.00 (d, *J* = 7.4 Hz, 2H), 7.78 (s, 1H), 7.57 (dd, *J* = 18.9, 7.4 Hz, 2H), 7.44 (t, *J* = 7.7 Hz, 3H), 7.15 (t, *J* = 7.7 Hz, 1H), 4.73 (dd, *J* = 11.4, 4.6 Hz, 1H), 4.66 – 4.56 (m, 1H), 3.88 (s, 2H), 3.65 – 3.52 (m, 1H), 2.57 (s, 1H).
^{13}C NMR (101 MHz, $CDCl_3$) δ 166.6, 137.0, 133.4, 133.1, 131.2, 130.6, 129.7, 129.5, 129.4, 128.5, 122.9, 64.3, 62.1, 46.7.
HRMS (ESI) m/z calcd for $C_{16}H_{16}BrO_3Se^+$ $[M+H]^+$ = 414.9443, found = 414.9440.
The ee value was 93%, t_R (major) = 11.2 min, t_R (minor) = 13.1 min (Chiralpak IB, λ = 254 nm, 10% *i*-PrOH/Hexane, flow rate = 1 mL/min).

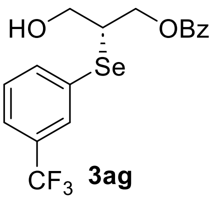

3ag

(*R*)-3-hydroxy-2-((3-(trifluoromethyl)phenyl)selanyl)propyl benzoate (3ag)
27 mg, 66% yield, a yellow foam, R_f = 0.5 (PE : DCM : Acetone = 5:5:0.3).
^{1}H NMR (400 MHz, $CDCl_3$) δ 8.05 – 7.93 (m, 2H), 7.91 – 7.76 (m, 2H), 7.63 – 7.50 (m, 2H), 7.49 – 7.36 (m, 3H), 4.74 (dd, *J* = 11.4, 4.7 Hz, 1H), 4.63 (dd, *J* = 11.1, 7.7 Hz, 1H), 3.97 – 3.85 (m, 2H), 3.70 – 3.55 (m, 1H), 2.60 (s, 1H).
^{13}C NMR (101 MHz, $CDCl_3$) δ 172.7, 166.7, 137.7, 133.4, 131.1, 131.1, 129.7, 129.6, 129.4, 128.8, 128.5, 124.9, 124.8, 64.2, 62.2, 46.8.
HRMS (ESI) m/z calcd for $C_{17}H_{16}F_3O_3Se^+$ $[M+H]^+$ = 405.0211, found = 405.0210.
The ee value was 93%, t_R (major) = 9.3 min, t_R (minor) = 10.5 min (Chiralpak IB, λ = 254 nm, 10% *i*-PrOH/Hexane, flow rate = 1 mL/min).

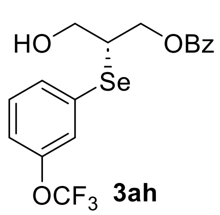
3ah

(*R*)-3-hydroxy-2-((3-(trifluoromethoxy)phenyl)selanyl)propyl benzoate (3ah)

30 mg, 71% yield, a colorless foam, R_f = 0.3 (PE : DCM : Acetone = 5:5:0.4).

^{1}H NMR (400 MHz, acetone-D$_6$) δ 7.97 (d, *J* = 7.6 Hz, 2H), 7.75 – 7.56 (m, 3H), 7.56 – 7.38 (m, 3H), 7.26 (d, *J* = 7.9 Hz, 1H), 4.80 – 4.62 (m, 2H), 4.43 (t, *J* = 5.6 Hz, 1H), 4.12 – 3.92 (m, 2H), 3.89 – 3.77 (m, 1H).

^{13}C NMR (151 MHz, CDCl$_3$) δ 166.6, 149.3, 133.4, 132.7, 130.4, 129.7, 129.4, 129.4, 128.5, 126.8, 120.5, 119.5, 64.2, 62.2, 46.7.

HRMS (ESI) m/z calcd for $C_{17}H_{15}F_3NaO_4Se^+$ $[M+H]^+$ = 442.9980, found = 442.9982.

The ee value was 93%, t_R (major) = 8.7 min, t_R (minor) = 9.9 min (Chiralpak IB, λ = 254 nm, 10% *i*-PrOH/Hexane, flow rate = 1 mL/min).

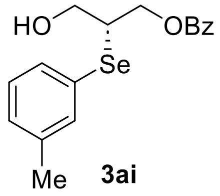
3ai

(*R*)-3-hydroxy-2-(m-tolylselanyl)propyl benzoate (3ai)

28 mg, 81% yield, a yellow foam, R_f = 0.5 (PE : DCM : Acetone = 5:4:0.3).

^{1}H NMR (400 MHz, CDCl$_3$) δ 8.01 (d, *J* = 7.7 Hz, 2H), 7.58 (t, *J* = 6.9 Hz, 1H), 7.51 – 7.38 (m, 4H), 7.22 – 7.08 (m, 2H), 4.78 – 4.69 (m, 1H), 4.62 – 4.53 (m, 1H), 3.92 – 3.80 (m, 2H), 3.61 – 3.52 (m, 1H), 2.62 (s, 1H), 2.32 (s, 3H).

^{13}C NMR (101 MHz, CDCl$_3$) δ 166.6, 139.2, 135.6, 133.2, 132.0, 129.7, 129.6, 129.1, 129.0, 128.4, 126.9, 64.5, 62.1, 46.4, 21.2.

HRMS (ESI) m/z calcd for $C_{17}H_{19}O_3Se^+$ $[M+H]^+$ = 351.0494, found = 351.0495.

The ee value was 85%, t_R (major) = 9.2 min, t_R (minor) = 10.4 min (Chiralpak IB, λ = 254 nm, 10% *i*-PrOH/Hexane, flow rate = 1 mL/min).

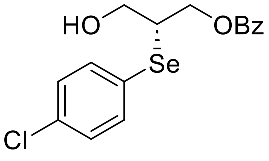
3ak

(*R*)-2-((4-chlorophenyl)selanyl)-3-hydroxypropyl benzoate (3ak)

26 mg, 71% yield, a colorless foam, R_f = 0.3 (PE : DCM : Acetone = 5:5:0.3).

^{1}H NMR (400 MHz, acetone-D_6) δ 7.92 (d, *J* = 7.6 Hz, 2H), 7.68 – 7.55 (m, 3H), 7.46 (t, *J* = 7.5 Hz, 2H), 7.29 (d, *J* = 8.2 Hz, 2H), 4.62 (d, *J* = 6.0 Hz, 2H), 4.31 (t, *J* = 5.4 Hz, 1H), 4.03 – 3.85 (m, 2H), 3.73 – 3.62 (m, 1H).

^{13}C NMR (101 MHz, $CDCl_3$) δ 166.8, 136.5, 134.8, 133.5, 129.8, 129.6, 128.6, 125.5, 64.5, 62.2, 46.8.

HRMS (ESI) m/z calcd for $C_{16}H_{15}ClNaO_3Se^+$ $[M+Na]^+$ = 392.9767, found = 392.9768.

The ee value was 95%, t_R (major) = 10.7 min, t_R (minor) = 12.2 min (Chiralpak IB, λ = 254 nm, 10% *i*-PrOH/Hexane, flow rate = 1 mL/min).

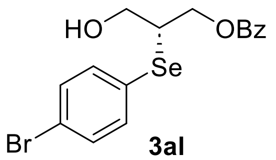

3al

(*R*)-2-((4-bromophenyl)selanyl)-3-hydroxypropyl benzoate (3al)

31 mg, 71% yield, a colorless foam, R_f = 0.4 (PE : DCM : Acetone = 5:5:0.4).

^{1}H NMR (400 MHz, CD_3OD) δ 7.92 (d, *J* = 7.2 Hz, 2H), 7.60 (t, *J* = 6.9 Hz, 1H), 7.56 – 7.49 (m, 2H), 7.49 – 7.35 (m, 4H), 4.69 – 4.58 (m, 2H), 3.98 – 3.84 (m, 2H), 3.62 (s, 1H).

^{13}C NMR (101 MHz, $CDCl_3$) δ 166.8, 136.7, 133.5, 132.6, 129.8, 129.6, 128.6, 126.3, 122.9, 64.5, 62.2, 46.7.

HRMS (ESI) m/z calcd for $C_{16}H_{15}BrNaO_3Se^+$ $[M+Na]^+$ = 436.9262, found = 436.9266.

The ee value was 93%, t_R (major) = 12.0 min, t_R (minor) = 14.2 min (Chiralpak IB, λ = 254 nm, 10% *i*-PrOH/Hexane, flow rate = 1 mL/min).

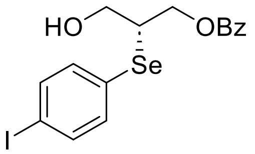

3am

(*R*)-3-hydroxy-2-((4-iodophenyl)selanyl)propyl benzoate (3am)

28 mg, 60% yield, a colorless foam, R_f = 0.3 (PE : DCM : Acetone = 5:5:0.3).

^{1}H NMR (600 MHz, $CDCl_3$) δ 7.99 (d, *J* = 7.9 Hz, 2H), 7.65 – 7.54 (m, 3H), 7.45 (t, *J* = 7.6 Hz, 2H), 7.34 (d, *J* = 8.1 Hz, 2H), 4.71 (dd, *J* = 11.6, 4.9 Hz, 1H), 4.58 (dd, *J* = 11.5, 7.7 Hz, 1H), 3.93 – 3.78 (m, 2H), 3.61 – 3.51 (m, 1H), 2.46 (s, 1H).

^{13}C NMR (151 MHz, $CDCl_3$) δ 166.6, 138.4, 138.3, 136.6, 136.5, 133.3, 129.7, 129.5, 128.5, 127.2, 94.2, 64.4, 62.1, 46.6.

HRMS (ESI) m/z calcd for $C_{16}H_{15}INaO_3Se^+$ $[M+Na]^+$ = 484.9123, found = 484.9124.

The ee value was 92%, t_R (major) = 12.9 min, t_R (minor) = 15.6 min (Chiralpak IB, λ = 254 nm, 10% *i*-PrOH/Hexane, flow rate = 1 mL/min).

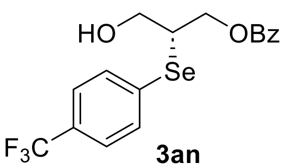

3an

(*R*)-3-hydroxy-2-((4-(trifluoromethyl)phenyl)selanyl)propyl benzoate (3an)

30 mg, 74% yield, a yellow foam, R_f = 0.5 (PE : DCM : Acetone = 5:5:0.4).

^{1}H NMR (400 MHz, $CDCl_3$) δ 7.99 (d, *J* = 7.6 Hz, 2H), 7.71 (d, *J* = 7.8 Hz, 2H), 7.58 (t, *J* = 7.3 Hz, 1H), 7.52 (d, *J* = 7.9 Hz, 2H), 7.44 (t, *J* = 7.5 Hz, 2H), 4.76 (dd, *J* = 11.6, 4.8 Hz, 1H), 4.63 (dd, *J* = 11.4, 7.7 Hz, 1H), 3.92 (d, *J* = 4.9 Hz, 2H), 3.72 – 3.63 (m, 1H), 2.56 (s, 1H).

^{13}C NMR (151 MHz, $CDCl_3$) δ 166.6, 133.9, 133.4, 133.1, 130.1, 129.7, 129.4, 128.5, 126.0, 126.0, 64.3, 62.2, 46.4.

HRMS (ESI) m/z calcd for $C_{17}H_{16}F_3O_3Se^+$ $[M+H]^+$ = 405.0211, found = 405.0215.

The ee value was 94%, t_R (major) = 10.9 min, t_R (minor) = 11.7 min (Chiralpak IB, λ = 254 nm, 10% *i*-PrOH/Hexane, flow rate = 1 mL/min).

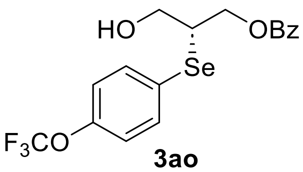

3ao

(*R*)-3-hydroxy-2-((4-(trifluoromethoxy)phenyl)selanyl)propyl benzoate (3ao)

30 mg, 72% yield, a pale-yellow foam, R_f = 0.5 (PE : DCM : Acetone = 5:5:0.5).

^{1}H NMR (400 MHz, $CDCl_3$) δ 8.01 (d, *J* = 7.7 Hz, 2H), 7.69 – 7.53 (m, 3H), 7.44 (t, *J* = 7.7 Hz, 2H), 7.14 (d, *J* = 8.2 Hz, 2H), 4.73 (dd, *J* = 11.6, 5.0 Hz, 1H), 4.59 (dd, *J* = 11.6, 7.6 Hz, 1H), 3.96 – 3.76 (m, 2H), 3.64 – 3.47 (m, 1H), 2.54 (s, 1H).

^{13}C NMR (101 MHz, $CDCl_3$) δ 166.6, 149.3, 136.5, 133.4, 129.7, 129.4, 128.5, 125.6, 121.7, 119.1, 64.3, 62.1, 46.8.

HRMS (ESI) m/z calcd for $C_{17}H_{16}F_3O_4Se^+$ $[M+H]^+$ = 421.0160, found = 421.0162.

The ee value was 88%, t_R (major) = 9.5 min, t_R (minor) = 10.3 min (Chiralpak IB, λ = 254 nm, 10% *i*-PrOH/Hexane, flow rate = 1 mL/min).

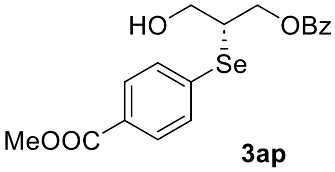

3ap

Methyl (*R*)-4-((1-(benzoyloxy)-3-hydroxypropan-2-yl)selanyl)benzoate (3ap)

26 mg, 65% yield, a pale-yellow foam, R_f = 0.5 (PE : EtOAc = 5:1).

^{1}H NMR (400 MHz, $CDCl_3$) δ 7.99 (d, *J* = 7.4 Hz, 2H), 7.92 (d, *J* = 7.8 Hz, 2H), 7.64 (d, *J* = 7.8 Hz, 2H), 7.57 (t, *J* = 7.0 Hz, 1H), 7.43 (t, *J* = 7.3 Hz, 2H), 4.75 (dd, *J* = 11.4, 4.5 Hz, 1H), 4.68 – 4.56 (m, 1H), 4.01 – 3.79 (m, 5H), 3.75 – 3.64 (m, 1H), 2.66 (s, 1H).
^{13}C NMR (151 MHz, $CDCl_3$) δ 166.6, 166.6, 134.9, 133.3, 133.0, 130.2, 129.7, 129.4, 129.2, 128.4, 64.3, 62.2, 52.2, 46.0.
HRMS (ESI) m/z calcd for $C_{18}H_{19}O_5Se^+$ $[M+H]^+$ = 395.0392, found = 395.0391.
The ee value was 93%, t_R (major) = 10.0 min, t_R (minor) = 13.5 min (Chiralpak IB, λ = 254 nm, 20% *i*-PrOH/Hexane, flow rate = 1 mL/min).

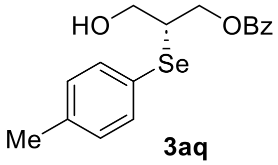

3aq

(*R*)-3-hydroxy-2-(*p*-tolylselanyl)propyl benzoate (3aq)
32 mg, 91% yield, a colorless oil, R_f = 0.3 (PE : DCM : Acetone = 5:5:0.3).
^{1}H NMR (400 MHz, $CDCl_3$) δ 7.90 (d, *J* = 8.0 Hz, 2H), 7.63 (d, *J* = 7.1 Hz, 2H), 7.36 – 7.15 (m, 5H), 4.72 (dd, *J* = 11.6, 4.9 Hz, 1H), 4.55 (dd, *J* = 11.5, 7.9 Hz, 1H), 3.93 – 3.75 (m, 2H), 3.61 – 3.50 (m, 1H), 2.41 (s, 3H).
^{13}C NMR (101 MHz, $CDCl_3$) δ 166.7, 144.0, 135.0, 129.7, 129.3, 129.1, 128.2, 127.2, 126.8, 64.2, 62.1, 46.5, 21.7.
HRMS (ESI) m/z calcd for $C_{17}H_{19}O_3Se^+$ $[M+H]^+$ = 351.0494, found = 351.0491.
The ee value was 96%, t_R (major) = 9.6 min, t_R (minor) = 11.3 min (Chiralpak IB, λ = 254 nm, 10% *i*-PrOH/Hexane, flow rate = 1 mL/min).

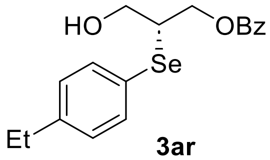

3ar

(*R*)-2-((4-ethylphenyl)selanyl)-3-hydroxypropyl benzoate (3ar)
29 mg, 87% yield, a colorless oil, R_f = 0.3 (PE : DCM : Acetone = 5:5:0.3).
^{1}H NMR (400 MHz, $CDCl_3$) δ 8.08 – 7.94 (m, 2H), 7.61 – 7.50 (m, 3H), 7.43 (t, *J* = 7.7 Hz, 2H), 7.12 (d, *J* = 7.9 Hz, 2H), 4.71 (dd, *J* = 11.5, 5.0 Hz, 1H), 4.55 (dd, *J* = 11.5, 7.8 Hz, 1H), 3.91 – 3.75 (m, 2H), 3.61 – 3.41 (m, 1H), 2.63 (q, *J* = 7.6 Hz, 2H), 1.22 (t, *J* = 7.6 Hz, 3H).
^{13}C NMR (101 MHz, $CDCl_3$) δ 166.7, 144.9, 135.7, 133.4, 129.8, 129.1, 128.5, 123.5, 64.6, 62.2, 46.5, 28.6, 15.5.
HRMS (ESI) m/z calcd for $C_{18}H_{21}O_3Se^+$ $[M+H]^+$ = 365.0650, found = 365.0652.
The ee value was 96%, t_R (major) = 9.0 min, t_R (minor) = 10.2 min (Chiralpak IB, λ = 254 nm, 10% *i*-PrOH/Hexane, flow rate = 1 mL/min).

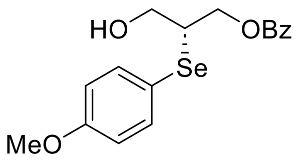

3as

(*R*)-3-hydroxy-2-((4-methoxyphenyl)selanyl)propyl benzoate (3as)

24 mg, 66% yield, a colorless foam, R_f = 0.2 (PE : DCM : Acetone = 5:5:0.4).

^{1}H NMR (400 MHz, $CDCl_3$) δ 8.00 (d, *J* = 7.5 Hz, 2H), 7.61 – 7.49 (m, 3H), 7.43 (t, *J* = 7.7 Hz, 2H), 6.82 (d, *J* = 8.6 Hz, 2H), 4.68 (dd, *J* = 11.6, 5.1 Hz, 1H), 4.52 (dd, *J* = 11.5, 7.8 Hz, 1H), 3.84 (dd, *J* = 11.9, 5.7 Hz, 1H), 3.79 (s, 3H), 3.78 – 3.73 (m, 1H), 3.51 – 3.35 (m, 1H), 2.48 (s, 1H).

^{13}C NMR (151 MHz, $CDCl_3$) δ 166.5, 160.1, 137.7, 133.2, 129.7, 128.4, 116.5, 115.0, 64.5, 61.9, 55.3, 46.6.

HRMS (ESI) m/z calcd for $C_{17}H_{19}O_4Se^+$ $[M+H]^+$ = 367.0443, found = 367.0446.

The ee value was 92%, t_R (major) = 13.8 min, t_R (minor) = 16.7 min (Chiralpak IB, λ = 254 nm, 10% *i*-PrOH/Hexane, flow rate = 1 mL/min).

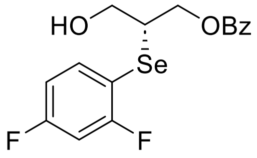

3at

(*R*)-2-((2,4-difluorophenyl)selanyl)-3-hydroxypropyl benzoate (3at)

23 mg, 62% yield, a yellow foam, R_f = 0.3 (PE : DCM : Acetone = 5:5:0.3).

^{1}H NMR (400 MHz, $CDCl_3$) δ 7.99 (d, *J* = 7.5 Hz, 2H), 7.70 – 7.51 (m, 2H), 7.44 (t, *J* = 7.5 Hz, 2H), 6.85 (q, *J* = 8.8 Hz, 2H), 4.70 (dd, *J* = 11.6, 4.9 Hz, 1H), 4.57 (dd, *J* = 11.4, 7.6 Hz, 1H), 3.98 – 3.72 (m, 2H), 3.65 – 3.46 (m, 1H), 2.58 (s, 1H).

^{13}C NMR (101 MHz, $CDCl_3$) δ 166.7, 139.0, 138.9, 138.9, 138.9, 133.5, 129.8, 129.6, 128.6, 112.6, 112.6, 112.4, 112.4, 104.9, 104.7, 104.7, 104.4, 64.5, 62.3, 46.3.

HRMS (ESI) m/z calcd for $C_{16}H_{15}F_2O_3Se^+$ $[M+H]^+$ = 373.0149, found = 373.0145.

The ee value was 95%, t_R (major) = 9.9 min, t_R (minor) = 11.1 min (Chiralpak IB, λ = 254 nm, 10% *i*-PrOH/Hexane, flow rate = 1 mL/min).

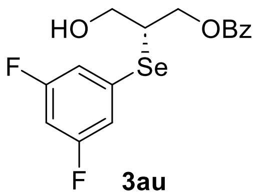

3au

(*R*)-2-((3,5-difluorophenyl)selanyl)-3-hydroxypropyl benzoate (3au)

23 mg, 62% yield, a colorless foam, R_f = 0.3 (PE : DCM : Acetone = 5:5:0.3).

^{1}H NMR (400 MHz, $CDCl_3$) δ 8.01 (d, *J* = 7.5 Hz, 2H), 7.59 (t, *J* = 7.4 Hz, 1H), 7.45 (t, *J* = 7.6 Hz, 2H), 7.14 (d, *J* = 5.0 Hz, 2H), 6.73 (t, *J* = 8.9 Hz, 1H), 4.74 (dd, *J* = 11.7, 5.0 Hz, 1H), 4.64 (dd, *J* = 11.7, 7.4 Hz, 1H), 3.92 (d, *J* = 5.5 Hz, 2H), 3.72 – 3.58 (m, 1H).

^{13}C NMR (101 MHz, $CDCl_3$) δ 166.7, 164.1, 163.9, 161.5, 161.4, 133.5, 133.4, 129.7, 129.3, 128.5, 128.4, 116.7, 116.6, 116.5, 116.4, 103.8, 103.6, 103.3, 64.2, 62.2, 46.6.

HRMS (ESI) m/z calcd for $C_{16}H_{15}F_2O_3Se^+$ $[M+H]^+$ = 373.0149, found = 373.0146.

The ee value was 87%, t_R (major) = 10.2 min, t_R (minor) = 11.3 min (Chiralpak IB, λ = 254 nm, 10% *i*-PrOH/Hexane, flow rate = 1 mL/min).

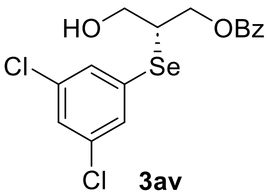

3av

(*R*)-2-((3,5-dichlorophenyl)selanyl)-3-hydroxypropyl benzoate (3av)

21 mg, 53% yield, a colorless oil, R_f = 0.4 (PE : DCM : Acetone = 5:5:0.3).

^{1}H NMR (400 MHz, $CDCl_3$) δ 8.00 (d, *J* = 7.5 Hz, 2H), 7.59 (t, *J* = 7.1 Hz, 1H), 7.54 – 7.35 (m, 4H), 7.27 (s, 1H), 4.81 – 4.55 (m, 2H), 3.99 – 3.81 (m, 2H), 3.69 – 3.54 (m, 1H), 2.56 (s, 1H).

^{13}C NMR (101 MHz, $CDCl_3$) δ 166.6, 135.3, 133.4, 132.0, 130.7, 129.7, 129.3, 128.5, 128.1, 64.2, 62.2, 46.9.

HRMS (ESI) m/z calcd for $C_{16}H_{15}Cl_2O_3Se^+$ $[M+H]^+$ = 404.9558, found = 404.9557.

The ee value was 94%, t_R (major) = 9.6 min, t_R (minor) = 10.7 min (Chiralpak IB, λ = 254 nm, 10% *i*-PrOH/Hexane, flow rate = 1 mL/min).

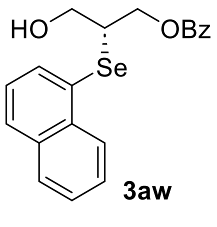

3aw

(*R*)-3-hydroxy-2-(naphthalen-1-ylselanyl)propyl benzoate (3aw)

29 mg, 75% yield, a colorless foam, R_f = 0.3 (PE : DCM : Acetone = 5:5:0.4).

^{1}H NMR (400 MHz, $CDCl_3$) δ 8.53 (d, *J* = 8.0 Hz, 1H), 8.06 – 7.70 (m, 5H), 7.66 – 7.33 (m, 6H), 4.78 – 4.55 (m, 2H), 3.87 (s, 2H), 3.65 – 3.48 (m, 1H), 2.60 (s, 1H).

^{13}C NMR (101 MHz, $CDCl_3$) δ 166.6, 135.6, 135.0, 134.1, 133.2, 129.8, 129.7, 129.5, 128.7, 128.4, 127.9, 127.1, 126.8, 126.4, 125.7, 64.4, 62.3, 46.3.

HRMS (ESI) m/z calcd for $C_{20}H_{19}O_3Se^+$ $[M+H]^+$ = 387.0494, found = 387.0491.

The ee value was 97%, t_R (major) = 13.8 min, t_R (minor) = 17.4 min (Chiralpak IB, λ = 254 nm, 10% *i*-PrOH/Hexane, flow rate = 1 mL/min).

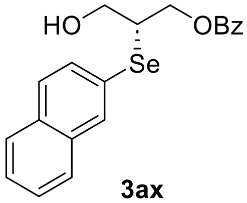

3ax

(*R*)-3-hydroxy-2-(naphthalen-2-ylselanyl)propyl benzoate (3ax)

20 mg, 51% yield, a colorless foam, R_f = 0.3 (PE : DCM : Acetone = 5:5:0.4).

^{1}H NMR (600 MHz, $CDCl_3$) δ 8.14 (s, 1H), 7.99 (d, *J* = 7.8 Hz, 2H), 7.85 – 7.72 (m, 3H), 7.67 (d, *J* = 8.4 Hz, 1H), 7.55 (t, *J* = 7.2 Hz, 1H), 7.53 – 7.44 (m, 2H), 7.40 (t, *J* = 7.5 Hz, 2H), 4.78 (dd, *J* = 11.5, 4.7 Hz, 1H), 4.63 (dd, *J* = 11.2, 7.9 Hz, 1H), 3.98 – 3.82 (m, 2H), 3.74 – 3.62 (m, 1H), 2.59 (s, 1H).

^{13}C NMR (151 MHz, $CDCl_3$) δ 166.6, 133.8, 133.2, 132.7, 131.8, 129.7, 129.6, 128.8, 128.4, 127.7, 127.5, 126.6, 126.6, 124.6, 64.6, 62.2, 46.5.

HRMS (ESI) m/z calcd for $C_{20}H_{19}O_3Se^+$ $[M+H]^+$ = 387.0494, found = 387.0493.

The ee value was 94%, t_R (major) = 16.2 min, t_R (minor) = 21.7 min (Chiralpak IB, λ = 254 nm, 10% *i*-PrOH/Hexane, flow rate = 1 mL/min).

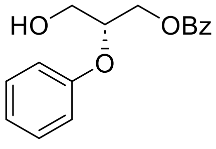

3ay

(*R*)-3-hydroxy-2-phenoxypropyl benzoate (3ay)

23 mg, 84% yield, a yellow foam, R_f = 0.4 (PE : EtOAc = 5:1).

^{1}H NMR (400 MHz, $CDCl_3$) δ 7.99 (d, *J* = 7.8 Hz, 2H), 7.57 (t, *J* = 7.4 Hz, 1H), 7.43 (t, *J* = 7.7 Hz, 2H), 7.31 (t, *J* = 7.9 Hz, 2H), 7.17 – 6.87 (m, 3H), 4.75 – 4.59 (m, 2H), 4.58 – 4.47 (m, 1H), 4.01 – 3.87 (m, 2H), 2.33 (s, 1H).

^{13}C NMR (101 MHz, $CDCl_3$) δ 166.5, 157.6, 133.3, 129.7, 129.4, 128.4, 121.9, 116.3, 76.4, 62.8, 61.9.

HRMS (ESI) m/z calcd for $C_{16}H_{17}O_4^+$ $[M+H]^+$ = 273.1121, found = 273.1124.

The ee value was 93%, t_R (major) = 15.7 min, t_R (minor) = 18.3 min (Chiralpak IB, λ = 254 nm, 10% *i*-PrOH/Hexane, flow rate = 0.8 mL/min).

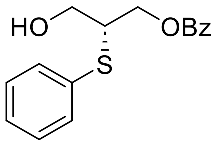

3az

(*R*)-3-hydroxy-2-(phenylthio)propyl benzoate (3az)

25 mg, 86% yield, a yellow foam, R_f = 0.3 (PE : EtOAc = 5:1).

^{1}H NMR (400 MHz, $CDCl_3$) δ 8.01 (d, *J* = 7.6 Hz, 2H), 7.57 (t, *J* = 7.2 Hz, 1H), 7.53 – 7.39 (m, 4H), 7.38 – 7.26 (m, 3H), 4.63 (dd, *J* = 11.4, 5.1 Hz, 1H), 4.48 (dd, *J* = 11.4, 7.4 Hz, 1H), 3.90 – 3.74 (m, 2H), 3.63 – 3.48 (m, 1H), 2.63 (s, 1H).

^{13}C NMR (101 MHz, $cdcl_3$) δ 166.7, 133.4, 132.9, 132.8, 129.8, 129.7, 129.4, 128.6, 127.9, 64.0, 61.7, 50.7.

HRMS (ESI) m/z calcd for $C_{16}H_{17}O_3S^+$ $[M+H]^+$ = 289.0893, found = 289.0896.

The ee value was 98%, t_R (major) = 16.6 min, t_R (minor) = 14.9 min (Chiralpak IB, λ = 254 nm, 10% *i*-PrOH/Hexane, flow rate = 1 mL/min).

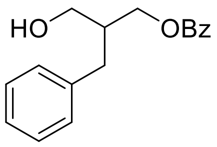

3ba

2-Benzyl-3-hydroxypropyl benzoate (3ba)

15 mg, 57% yield, a yellow foam, R_f = 0.4 (PE : EtOAc = 10:1).

^{1}H NMR (400 MHz, $CDCl_3$) δ 8.05 (d, *J* = 7.6 Hz, 2H), 7.59 (t, *J* = 7.2 Hz, 1H), 7.51 – 7.36 (m, 3H), 7.34 – 7.23 (m, 4H), 4.46 (dd, *J* = 11.2, 4.2 Hz, 1H), 4.33 (dd, *J* = 11.1, 6.2 Hz, 1H), 3.69 (dd, *J* = 11.3, 4.4 Hz, 1H), 3.59 (dd, *J* = 11.2, 6.4 Hz, 1H), 2.84 (s, 1H), 2.76 (dt, *J* = 14.5, 5.8 Hz, 2H), 2.27 (s, 1H).

^{13}C NMR (101 MHz, $CDCl_3$) δ 167.1, 139.3, 133.2, 129.8, 129.6, 129.6, 129.1, 128.5, 126.3, 64.1, 62.0, 42.8, 34.4.

HRMS (ESI) m/z calcd for $C_{17}H_{19}O_3^+$ $[M+H]^+$ = 271.1329, found = 271.1325.

The ee value was 36%, t_R (major) = 8.0 min, t_R (minor) = 7.4 min (Chiralpak IB, λ = 254 nm, 30% *i*-PrOH/Hexane, flow rate = 0.8 mL/min).

3. Mechanistic and Computational Studies

(1) Mechanistic studies

Evidence for enantioselective desymmetrization

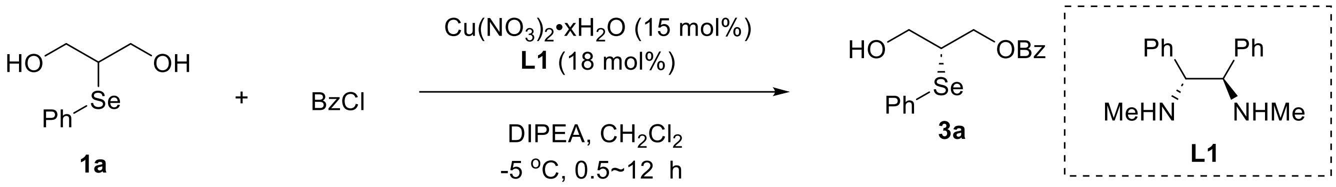

General procedure at 0.1 mmol: $Cu(NO_3)_2$•xH_2O (CAS#13778-31-9 from Macklin) (0.015 mmol, 0.15 eq) and **L1** (0.018 mmol, 0.18 eq) were added to DCM (2.0 mL) in a 10-mL tube equipped with a magnetic stirring bar. After stirring for 30 min, **1a** (0.1 mmol, 1.0 eq) was added to the solution and the mixture was cooled to -5 °C. Benzoyl chloride (0.12 mmol, 1.2 eq) was then added to the mixture, followed by the addition of DIPEA (0.12 mmol, 1.2 eq). Then the mxiture was allowed to stir at -5 °C for 0.5~12 hours. The ee value and yield of **3a** at given time were tested via crude ^{1}H NMR and HPLC, respectively.

Table S2. Relationship between the yield and ee value of 3a over reaction time

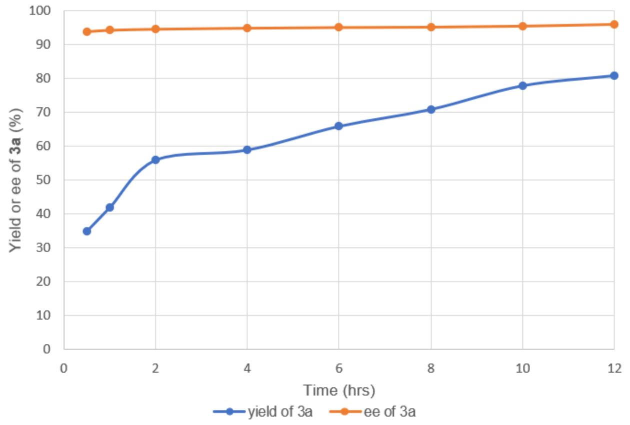

Time (h)	yield of **3a**	ee of **3a**
0.5	35	94
1	42	94.4
2	56	94.7
4	59	95
6	66	95.2
8	71	95.3
10	78	95.6
12	81	96.1

Nonlinear effect study on the ee values of 3a and L1

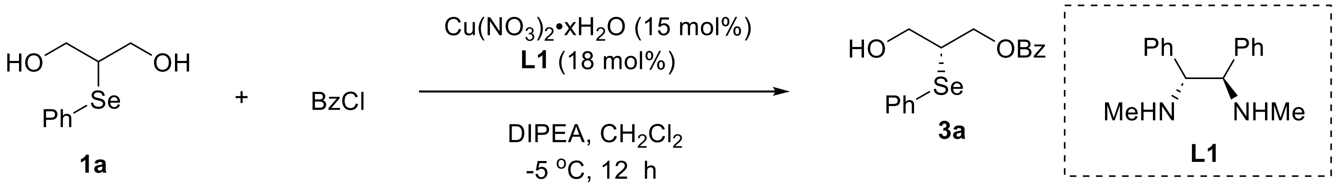

ee of **L1** = 0%, 20%, 40%, 60%, 80%, >99%

General procedure at 0.1 mmol: $Cu(NO_3)_2$•xH_2O (CAS#13778-31-9 from Macklin) (0.015 mmol, 0.15 eq) and **L1** of gradient ee values (0.018 mmol, 0.18 eq) were added to CH_2Cl_2 (2.0 mL) in a 10-mL tube equipped with a magnetic stirring bar. After stirring for 30 min, **1a** (0.1 mmol, 1.0 eq) was added to the solution and the mixture was cooled to -5 °C. Benzoyl chloride (0.12 mmol, 1.2 eq) was then added to

the mixture, followed by the addition of DIPEA (0.12 mmol, 1.2 eq). Then the mxiture was allowed to stir at -5 °C for 12 hours.

Table S3. Relationship between the ee value of L1 and 3a

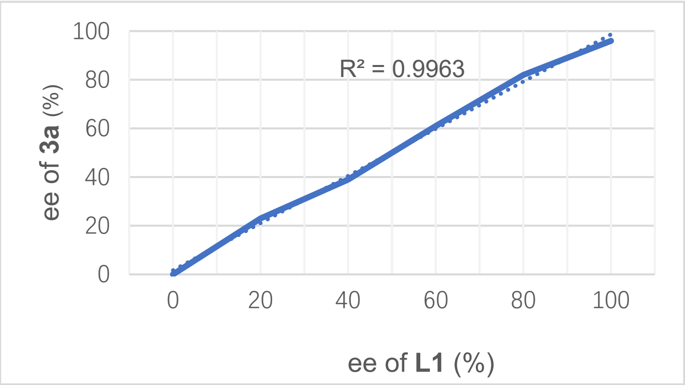

ee of **L1**	ee of **3a**
0	0
20	23
40	39
60	61
80	82
100	96

(2) Computational methods

All DFT calculations were carried out by using Gaussian16 Rev. C.01.[1] The B3LYP[2] functional, in combination with D3(BJ) correction[3-4], was used throughout the DFT calculations. With this protocol, the structures of the intermediates and transition states were optimized and characterized by frequency analyses with a mixed basis set of SDD for the Cu and Se and the def2-SVP[5] basis set for all other atoms in the gas phase. It was confirmed that the optimized transition states have unique imaginary frequency. Intrinsic reaction coordinate (IRC) calculations[6-7] are additionally carried out to further characterize the true nature of transition states. On the basis of the optimized structures, single-point energies were computed under the SMD(CH_2Cl_2)[8-9] solvation model with the functional B3LYP-D3(BJ) using the def2-TZVPP[5, 10] basis set (SDD basis set for Cu and Se) to improve the energies. Using the single-point energies and the thermal corrections from frequency analysis calculations, the free energies were calculated at 268.15 K and 1 M, and a correction factor of 1.89 kcal/mol for the standard state change from 1 atm to 1 M was applied. The wave function analysis was proceeded by using Multiwfn software[11] in which the IGMH[12] and electrostatic potential colored molecular van der Waals surface penetration map between molecules were operated. The energy decomposition based on dispersion-corrected density functional theory (DFT) (sobEDA)[13] was used. Distortion-interaction model analysis[14-15] was employed to analyze the steric effect and the intermolecular interaction in transition states. To further quantify the steric hindrance of the C_2-symmetric diamine ligands, the steric map and the percent buried volumes (%V_{bur}) were calculated using SambVca 2.1[16] based on the optimized structure. 3D molecular structures were prepared by CYLview[17], IGMH and ESP colored molecular penetration map was further visualized using VMD 1.9.3 program[18].

(3) The barrierless deprotonation of Int1 by DIPEA

Efforts to locate a transition state of the deprotonation (O-H bond cleavage, Figure S1) from Int1 failed. Then, we did a relaxed scan of the O-H bond at BP86-D3/def2-SVP level with density fitting techniques in Figure S2-S3. The single-point energies only decrease with the O-H bond increasing from 1.01 Å to 1.55 Å, which indicates that it is a barrierless process. Further structural optimizations and single-point energy calculations revealed that this deprotonation process is exothermic, consistent with our scan results. (Figure S4-S5).

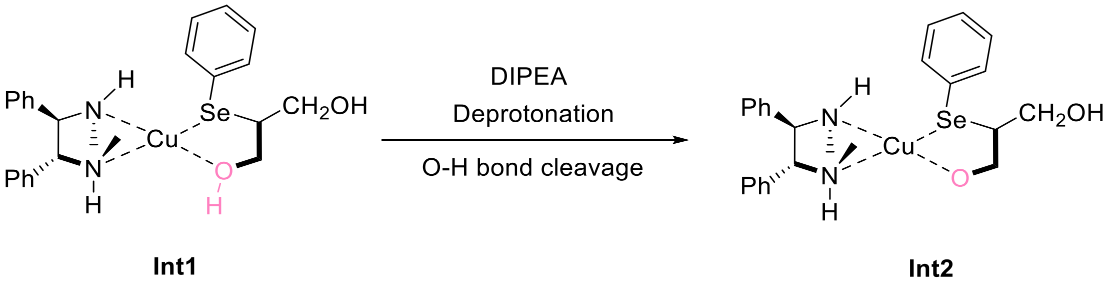

Figure S1. The deprotonation of **Int1**

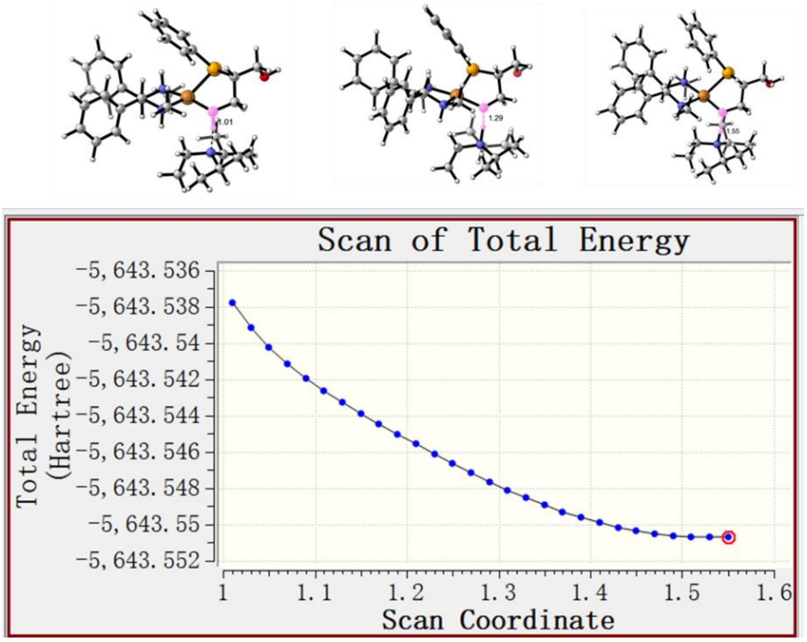

Figure S2. The scan of O-H bond in **Int1(*R*)**

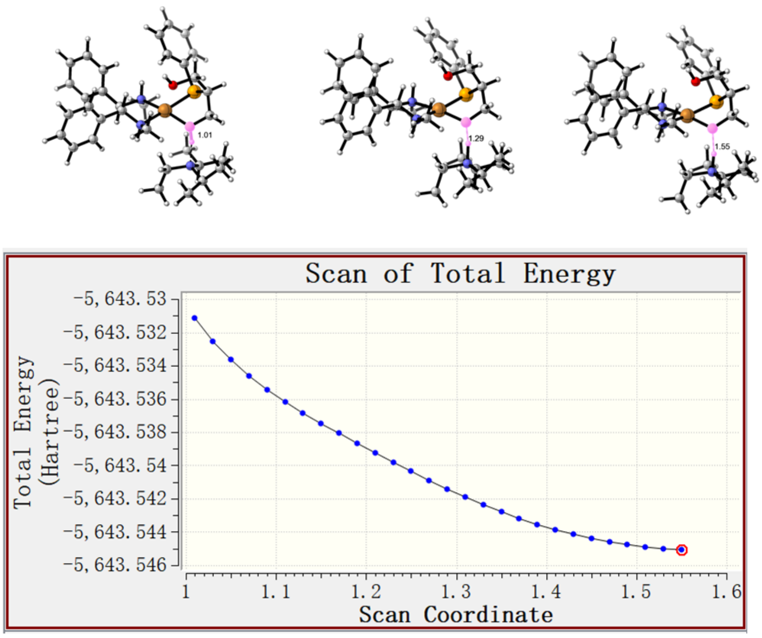

Figure S3. The scan of O-H bond in **Int1(*S*)**

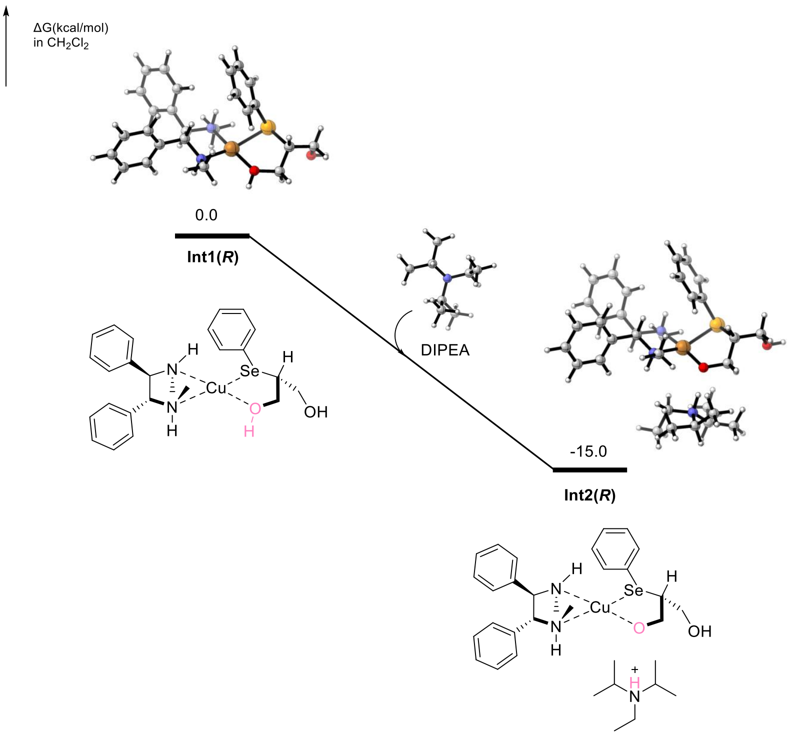

Figure S4. DFT-Computed free energy changes of the deprotonation of **Int1(*R*)** with B3LYP-D3(BJ)/def2-TZVPP(SDD for Cu and Se)/SMD(CH_2Cl_2)// B3LYP-D3(BJ)/def2-SVP(SDD for Cu and Se) level of theory at 268.15 K.

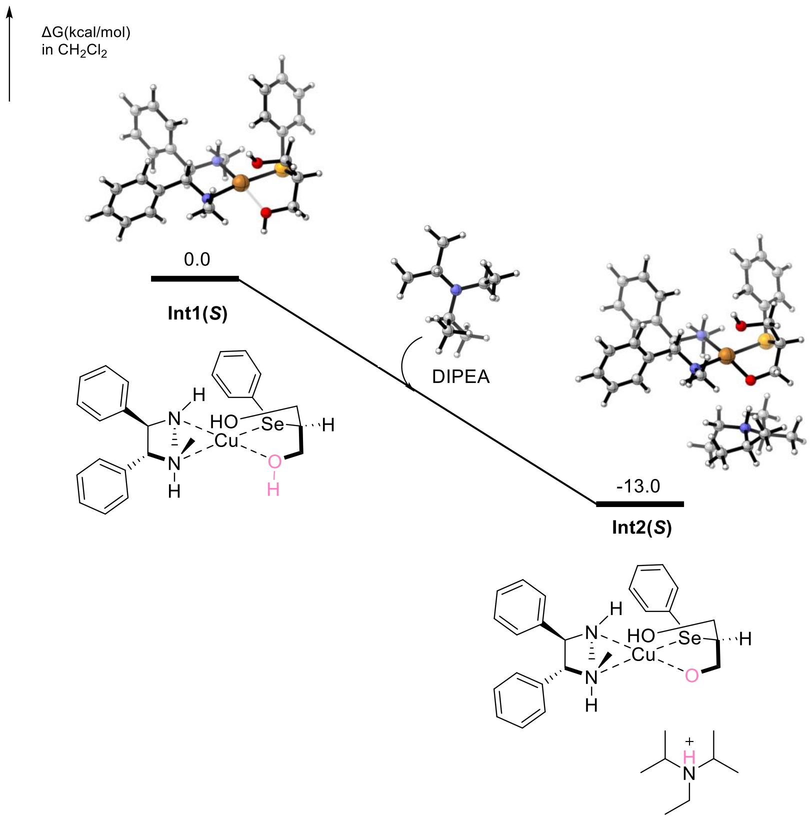

Figure S5. DFT-Computed free energy changes of the deprotonation of **Int1(*S*)** with B3LYP-D3(BJ)/def2-TZVPP(SDD for Cu and Se)/SMD(CH_2Cl_2)// B3LYP-D3(BJ)/def2-SVP(SDD for Cu and Se) level of theory at 268.15 K.

(4) Transition states of acyl transfer step

Conformational searches have been performed for the transition states of the acyl transfer step with benzoyl chloride. The optimized structures and relative Gibbs free energies of these transition states are listed in Figure. S6. The lowest-energy transition states leading to the major (*R*) and minor (*S*) enantiomers, respectively, are shown in the main text.

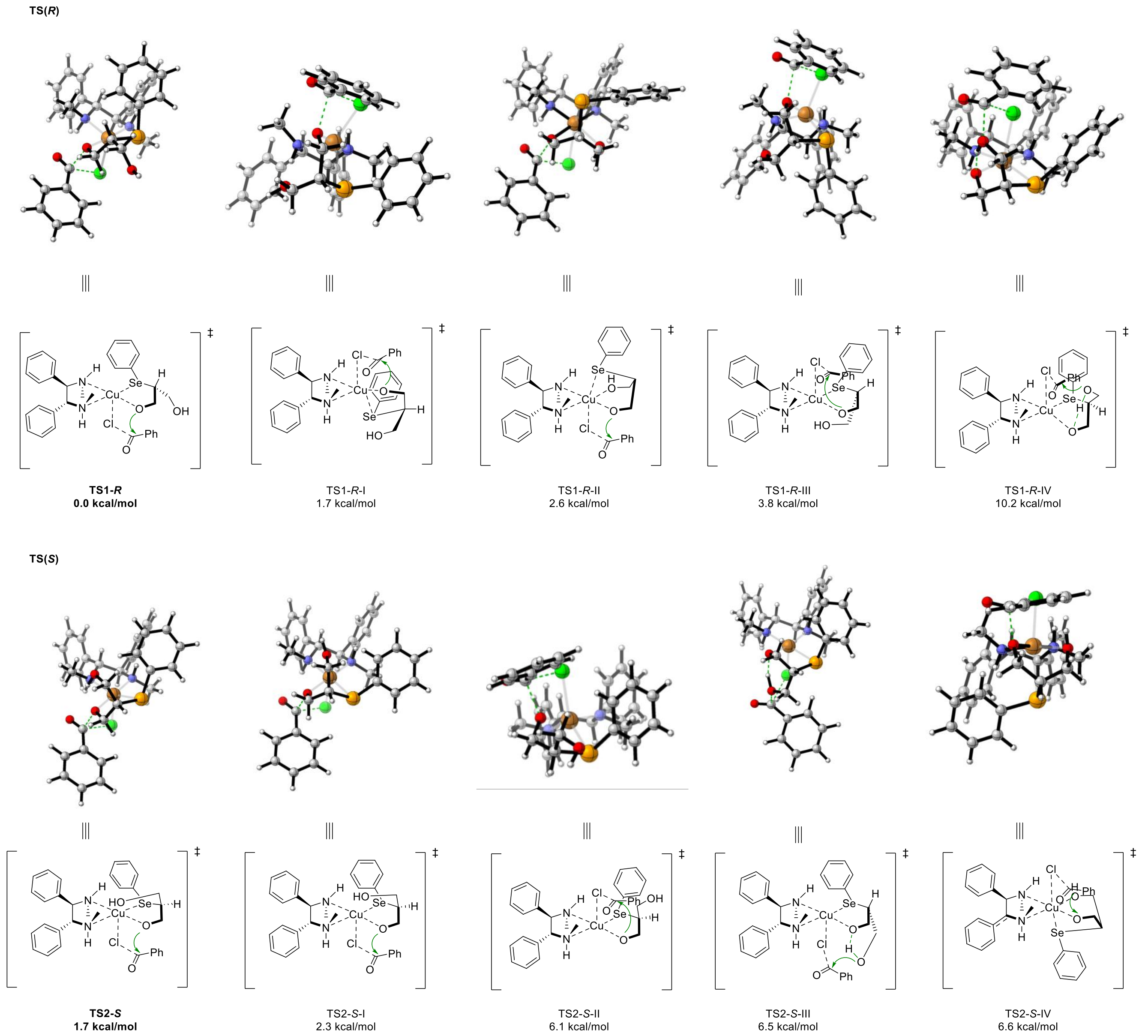

Figure S6. Optimized structures and relative Gibbs free energies (in kcal/mol) of different conformers for the transition states of the acyl transfer step with B3LYP-D3(BJ)/def2-TZVPP(SDD for Cu and Se)/SMD(CH_2Cl_2)//B3LYP-D3(BJ)/ def2-SVP(SDD for Cu and Se) level of theory at 268.15 K.

(5) Activation energy of the enantio-determining transition states

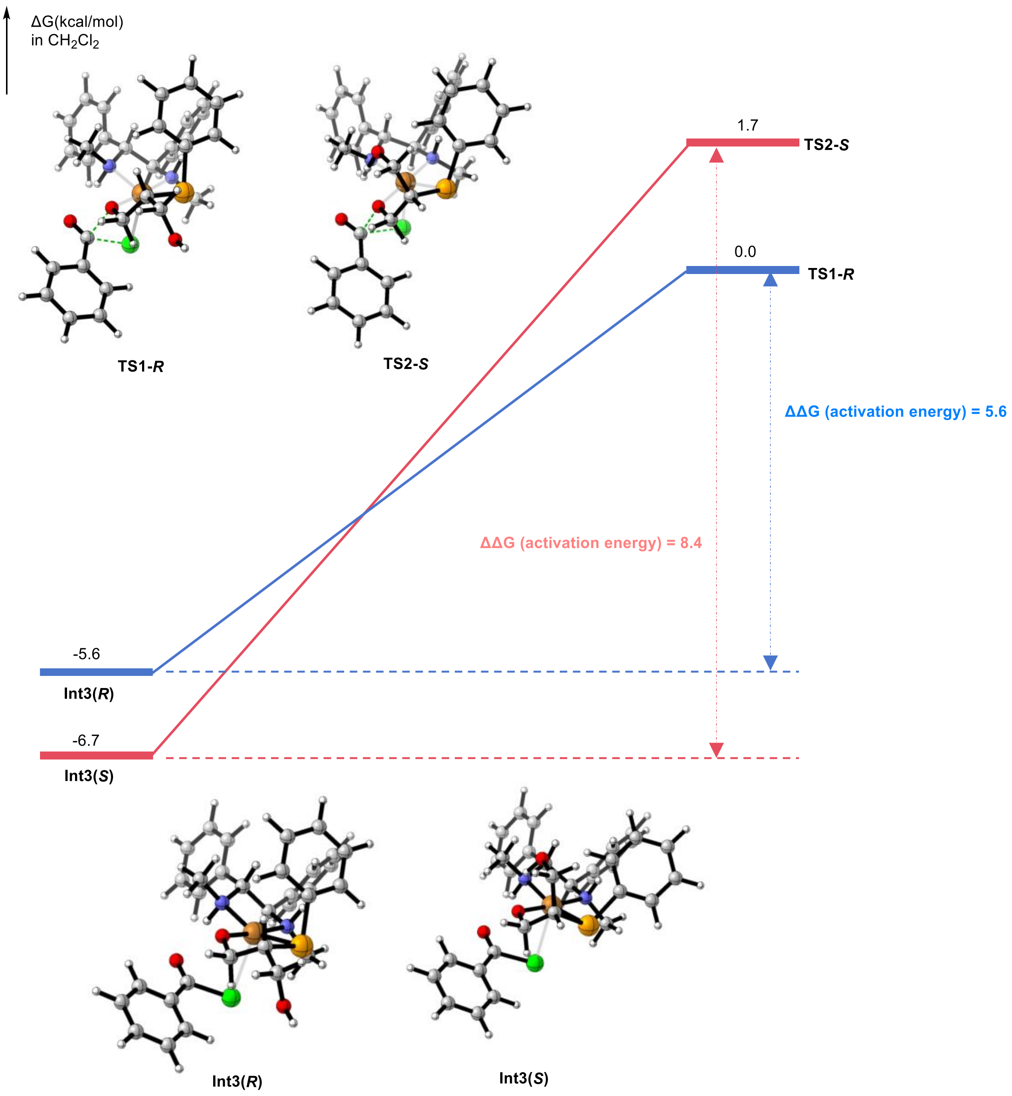

Figure S7. DFT-Computed free energy changes of the asymmetric acyl transfer of **Int3** with B3LYP-D3(BJ)/def2-TZVPP(SDD for Cu and Se)/SMD(CH_2Cl_2)// B3LYP-D3(BJ)/def2-SVP(SDD for Cu and Se) level of theory at 268.15 K.

(6) Steric map

Computation of the catalyst's steric map and buried volume was performed employing SambVca 2.1, with the structure of the optimized lowest-energy transition state.

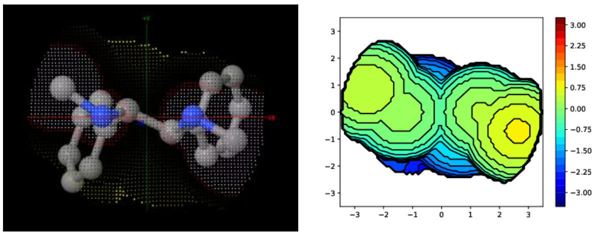

V Free	V Buried	V Total	V Exact
113.4	66.1	179.5	179.6

%V_Free	%V_Bur	% Tot/Ex
63.2	36.8	100.0

Quadrant	V_f	V_b	V_t	%V_f	%V_b
SW	31.0	13.9	44.9	69.0	31.0
NW	26.9	17.9	44.9	60.0	40.0
NE	30.4	14.5	44.8	67.8	32.2
SE	25.1	19.8	44.9	56.0	44.0

Figure S8. Topographic steric maps of Cu-complexes based on the density functional theory (DFT)-optimized structure.

(7) Distortion-interaction analysis

We performed a distortion-interaction analysis as proposed by Fernández *et al.* for unimolecular reactions[19] by fragmentation into a four-coordinate copper complex as part A and the benzoyl chloride as part B. In this model, the activation energy ($\Delta E^{\ddagger}$) is decomposed into the difference in the distortion energy ($\Delta\Delta E$_strain) and the difference in the interaction energy ($\Delta\Delta E$_int) between the two fragments, during the transition from relaxed structures to the transition-state geometry.

In detail, based on the gas-phase optimized structures of both the transition states and the relaxed reactants, we decomposed the structures into part A and part B. The distortion energy (ΔE_strain) and interaction energy (ΔE_int) were then computed respectively—for both the transition states and relaxed structures—using the B3LYP-D3(BJ)/def2-TZVPP(SDD for Cu and Se)/SMD(CH_2Cl_2) method.

Table S4. Distortion-interaction analysis

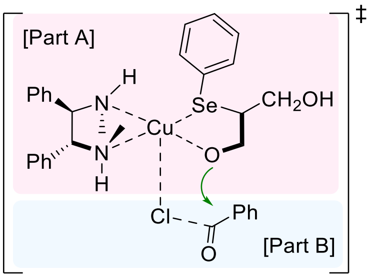

TS1-*R*	TS2-*S*
favour	disfavour
ΔΔE‡ = 4.6 kcal/mol	ΔΔE‡ = 7.3 kcal/mol
ΔΔG‡ = 5.6 kcal/mol	ΔΔG‡ = 8.4 kcal/mol

	**TS1-*R*(favour)**	**TS2-*S*(disfavour)**	**disfavour-favour**
**$\Delta\Delta E^{\ddagger}$**	**4.58**	**7.31**	**2.73**
**$\Delta\Delta E^{\ddagger}$_strain**	**17.70**	**21.72**	**4.02**
**$\Delta\Delta E^{\ddagger}$_strain_A**	**1.05**	**2.48**	**1.43**
**$\Delta\Delta E^{\ddagger}$_strain_B**	**16.65**	**19.24**	**2.59**
**$\Delta\Delta E^{\ddagger}$_int**	**-13.11**	**-14.41**	**-1.29**
ΔE^{TS}_strain_TS	17.72	21.93	4.21
ΔE^{TS}_strain_TS_A	0.99	2.56	1.56
ΔE^{TS}_strain_TS_B	16.73	19.38	2.65
ΔE^{R}_strain_Relax	0.02	0.22	0.19
ΔE^{R}_strain_Relax_A	-0.06	0.08	0.13
ΔE^{R}_strain_Relax_B	0.08	0.14	0.06
ΔE^{TS}_int_TS	-21.74	-23.10	-1.37
ΔE^{R}_int_Relax	-8.63	-8.70	-0.07

E: electronic energy, *E*_int: interaction energy, *E*_strain: distortion energy

$\Delta\Delta E^{\ddagger} = \Delta\Delta E^{\ddagger}$_strain + $\Delta\Delta E^{\ddagger}$_int

$$\Delta\Delta E^{\ddagger}\text{\_strain} = \Delta E\text{TS\_strain\_TS} - \Delta E\text{R\_strain\_Relax}$$

$$\Delta\Delta E^{\ddagger}\text{\_int} = \Delta E\text{TS\_int\_TS} - \Delta E\text{R\_int\_Relax}$$

(8) IGMH analysis

Based on the gas-phase optimized structures of the enantiodetermining transition states, the transition states were divided into three fragments (Part ABC), and a weak interaction analysis (IGMH method) was performed on each fragment using Multiwfn software, which was further visualized using the VMD 1.9.3 program.

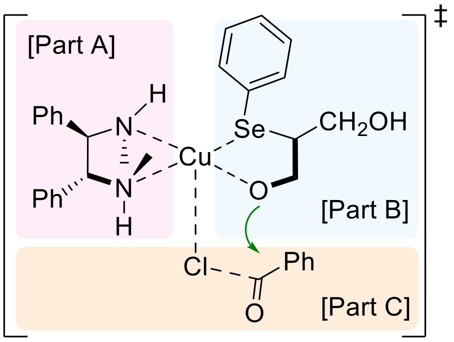

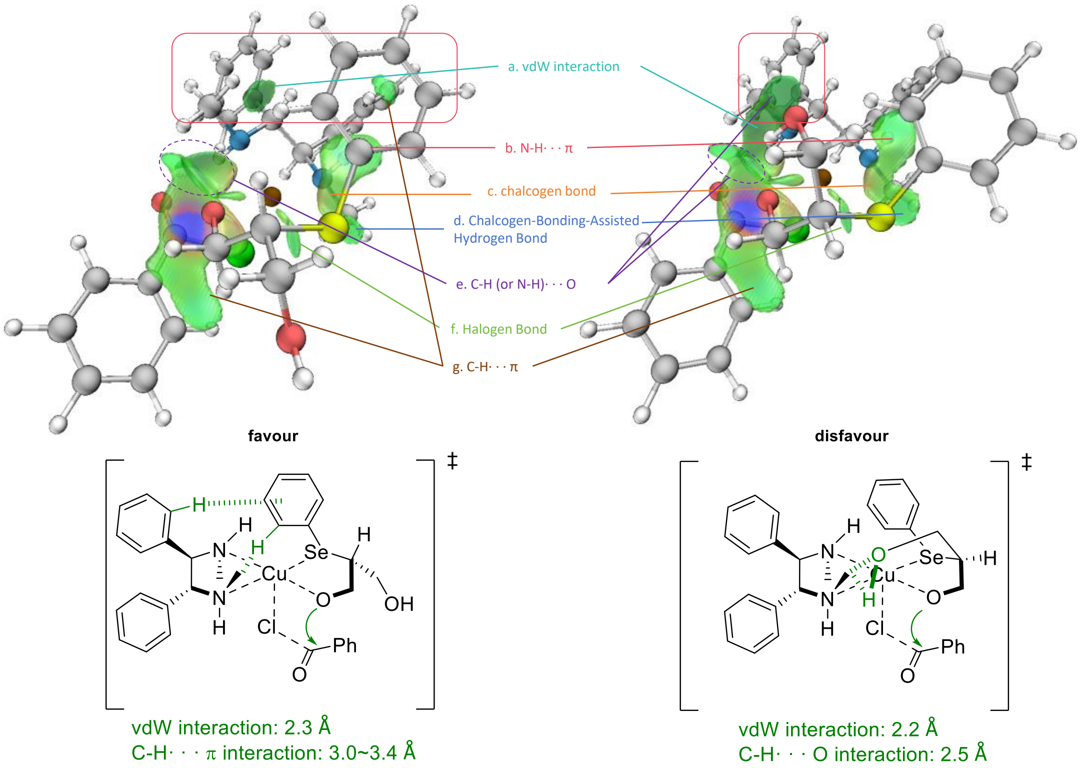

Figure S9. IGMH analysis of the non-covalent interactions for transition states

(9) Energy decomposition analysis (EDA)

We performed a more detailed decomposition of the interaction energy using the sobEDA program, and the relevant decomposition results are:

$$\Delta E_{int} = \Delta E_{rep} + \Delta E_{x} + \Delta E_{elstat} + \Delta E_{orb} + \Delta E_{DFTc} + \Delta E_{dc}$$

Among these terms, the Pauli repulsion energy (ΔE_rep) and Exchange interaction energy (ΔE_x) can be considered as the contribution part of steric effects, and are collectively referred to as the exchange-repulsion interaction energy (ΔE_xrep). The sum of dispersion correction energy (ΔE_dc) and DFT correlation energy (ΔE_DFTc) can be considered to represent the dispersion effects (ΔE_disp). So:

$$\Delta E_{int} = \Delta E_{xrep} + \Delta E_{elstat} + \Delta E_{orb} + \Delta E_{disp}$$

Based on the gas-phase optimized structures, energy decomposition analysis was performed at the B3LYP-D3BJ/def2-TZVP level of theory with basis set superposition error (BSSE) correction. The sobEDAw method (c = 0.538, a = 0.066, r = 2.724) was applied to ensure that the dispersion/electrostatic interaction results closely match those from SAPT calculation[20-21]. The raw data obtained from applying the above decomposition schemes to **TS1-*R*** and **TS1-*S*** are provided in **Tab. S5-S7**, and the difference (ΔΔE) between the two transition states is used for comparison in subsequent analyses. Furthermore, different partitioning schemes were applied to the components of the transition states. The calculations revealed that when the seleno-diol substrate is treated as an individual fragment, electrostatic interactions are a contributing factor. This suggests that the electrostatic interactions between the seleno-diol substrate and the catalyst may also contribute partially to enantioselective control.

Table S5. Two-fragment EDA with benzoyl chloride treated as an individual fragment

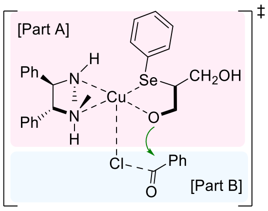

kcal/mol	Total interaction energy(ΔE_int)				Total interaction energy (ΔE_int)
	Exchange-repulsion(ΔE_xrep)	Electrostatic (ΔE_els)	Orbital (ΔE_orb)	Dispersion (ΔE_disp)	
TS_favour	133.63	-81.26	-55.39	-30.96	-33.98
TS_disfavour	141.59	-85.32	-60.15	-31.84	-35.72
	ΔΔE_xrep	ΔΔE_els	ΔΔE_orb	ΔΔE_disp	ΔΔE_int
Disfavour-Favour	7.96	-4.06	-4.76	-0.88	-1.74

Table S6. Two-fragment EDA with seleno-diol treated as an individual fragment

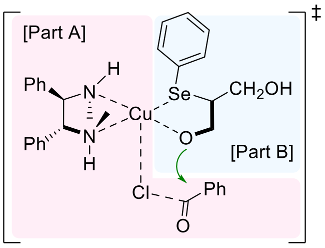

kcal/mol	Total interaction energy(ΔE_int)				Total interaction energy (ΔE_int)
	Exchange-repulsion(ΔE_xrep)	Electrostatic (ΔE_els)	Orbital (ΔE_orb)	Dispersion (ΔE_disp)	
TS_favour	250.79	-316.75	-161.98	-53.23	-281.17
TS_disfavour	246.87	-303.36	-161.12	-52.04	-269.65
	ΔΔE_xrep	ΔΔE_els	ΔΔE_orb	ΔΔE_disp	ΔΔE_int
Disfavour-Favour	-3.92	13.39	0.86	1.19	11.52

Table S7. Three-fragment EDA

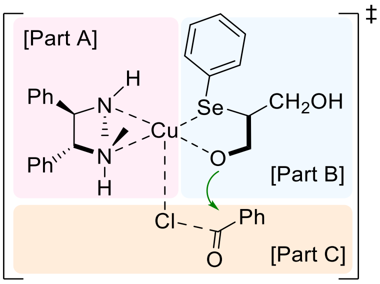

kcal/mol	Total interaction energy(ΔE_int)				Total interaction energy (ΔE_int)
	Exchange-repulsion(ΔE_xrep)	Electrostatic (ΔE_els)	Orbital (ΔE_orb)	Dispersion (ΔE_disp)	
TS_favour	290.14	-362.9	-184.11	-70.07	-326.94
TS_disfavour	286.01	-349.28	-183.83	-68.93	-316.03
	ΔΔE_xrep	ΔΔE_els	ΔΔE_orb	ΔΔE_disp	ΔΔE_int
Disfavour-Favour	-4.13	**13.62**	0.28	1.14	**10.91**

(10) Electrostatic potential analysis

The gas-phase optimized structures of the enantiodetermining transition states were partitioned into two fragments (denoted as Part A and B, with Part A carrying a charge of +1 and Part B a charge of -1). Subsequently, an electrostatic potential-colored molecular penetration map was then computed using the Multiwfn software and visualized using the VMD 1.9.3 program.

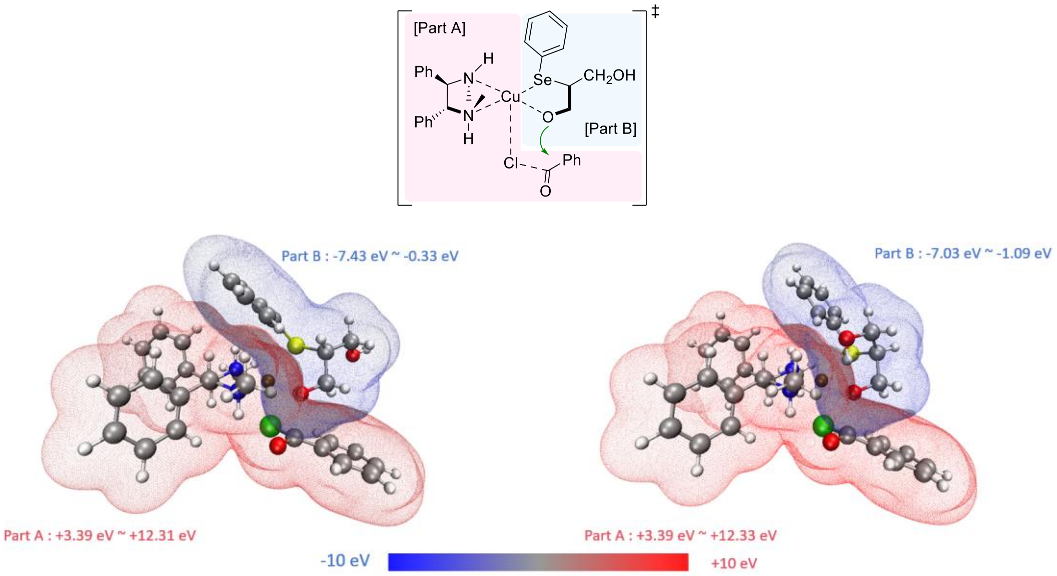

Figure S10. ESP colored molecular penetration map of key transition states

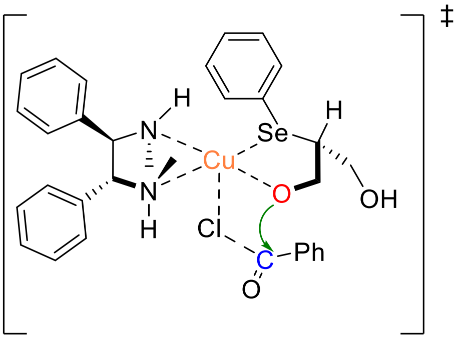

Hirshfeld Charge
O : -0.26
Cu : +0.44
C : +0.23

Favour

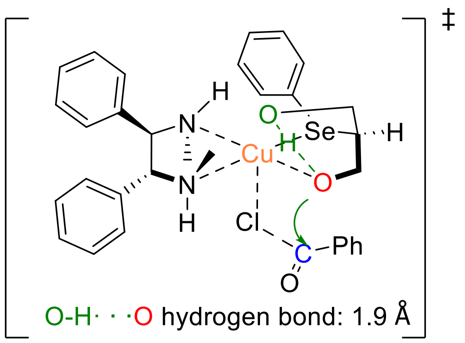

Hirshfeld Charge
O : -0.24
Cu : +0.43
C : +0.23

Disfavour

Figure S11. Hirshfeld Charge of key transition states

(11) Free energy

Table S8. Computed Energies of All of the Stationary Points: Thermal corrections to Gibbs energies (TCGs), single-point energies (SPEs) at high/low level

	Imaginary Frequencies (cm^{-1})	TCGs[a] (hartree)	SPEs[a] (hartree)	SPEs[b] (hartree)
DIPEA	-	0.224422	-370.8114091	-371.2360569
Int1(*R*)	-	0.463057	-1437.368436	-1438.982293
Int2(*R*)	-	0.713569	-1808.255367	-1810.263208
Int1(*S*)	-	0.464207	-1437.378328	-1438.983814
Int2(*S*)	-	0.713618	-1808.261958	-1810.260175
**TS1-*R***	-198.1609	0.543037	-2241.948765	-2243.936146
TS1-*R*-I	-141.608	0.543245	-2241.951443	-2243.933691
TS1-*R*-II	-197.4007	0.544005	-2241.953433	-2243.932927
TS1-*R*-III	-173.2553	0.543150	-2241.946067	-2243.930224
TS1-*R*-IV	-204.6736	0.545126	-2241.939905	-2243.921663
**TS2-*S***	-207.6232	0.545619	-2241.954528	-2243.935832
TS2-*S*-I	-201.3408	0.543472	-2241.949779	-2243.932897
TS2-*S*-II	-182.7342	0.543134	-2241.938108	-2243.926483
TS2-*S*-III	-207.2905	0.542099	-2241.940715	-2243.924816
TS2-*S*-IV	-200.1918	0.54683	-2241.950552	-2243.929103
Int3(*R*)	-	0.54119	-2241.958958	-2243.94345
Int3(*S*)	-	0.543612	-2241.966372	-2243.947486

[a]Calculated at B3LYP-D3(BJ)/def2-SVP(SDD for Cu and Se) level of theory and T = 268.15 K.

[b]Calculated at B3LYP-D3(BJ)/def2-TZVPP(SDD for Cu and Se)/SMD(CH_2Cl_2) level of theory.

(12) Cartesian Coordinates for the Stationary Points

DIPEA

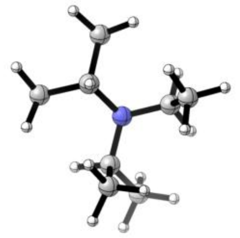

0 1

N	-0.05029700	0.02471600	-0.43082800
C	-1.28594000	-0.40168400	0.23815900
H	-1.28639800	-0.10070200	1.31136300
C	0.10672600	1.46845300	-0.57881800
H	1.05936500	1.65374800	-1.09331100
H	-0.66478700	1.83583100	-1.27511600
C	1.16106100	-0.74484300	-0.11618800
H	0.88459900	-1.79887600	-0.25919000
C	2.28725600	-0.48447100	-1.12107200
H	3.07283800	-1.24751700	-1.00644200
H	1.90239800	-0.53261800	-2.15103800
H	2.76802300	0.49571800	-0.97470700
C	1.67784000	-0.61517800	1.32945200
H	2.12593200	0.37338100	1.51376900
H	0.87030800	-0.76175200	2.06350500
H	2.45386700	-1.37172000	1.53057300
C	0.05530200	2.33136200	0.69272000
H	0.15221100	3.39651000	0.42550700
H	-0.89797800	2.21533000	1.23169300
H	0.86411400	2.08773300	1.39623000
C	-1.46771800	-1.92382700	0.20815200
H	-1.39962500	-2.29950400	-0.82589400
H	-0.72793700	-2.45818700	0.82052300
H	-2.46129200	-2.18315900	0.60343600
C	-2.51231700	0.25231400	-0.41205600
H	-2.52649900	1.34496600	-0.29742300
H	-2.53721800	0.02010900	-1.48916800
H	-3.43310500	-0.13506700	0.04938300

Int1(*R*)

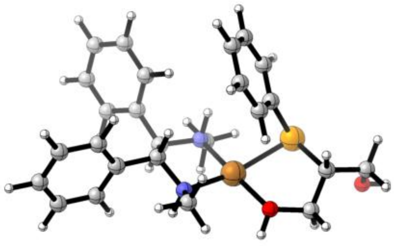

2 2

N	-0.21051500	0.45907300	-1.27822600
N	-0.64702700	-1.94739500	0.03003000
C	-1.63038200	0.01162400	-1.08541200
C	-1.70509400	-0.88681100	0.15688900
C	-2.56941800	1.19072200	-1.00293700
C	-3.08589800	-1.46036000	0.36352200
C	-3.59566600	1.35261800	-1.94069200
C	-4.46558800	2.44239900	-1.85048700
C	-4.31012200	3.37950500	-0.82571100
C	-3.28465900	3.22503000	0.11355000
C	-2.41986900	2.13365500	0.02793100
C	-3.65979500	-2.30205700	-0.60293200
C	-4.94061200	-2.82259600	-0.41369100
C	-5.66250400	-2.49825000	0.73994100
C	-5.09936700	-1.65690800	1.70310500
C	-3.81404800	-1.14275500	1.51637300
Cu	1.03272700	-0.93257500	-0.52927100
H	-1.88496400	-0.59405200	-1.96834400
H	-1.42719700	-0.29196900	1.04097500
H	-3.72476700	0.61886300	-2.74026200
H	-5.26684700	2.55948900	-2.58280000
H	-4.98961900	4.23143800	-0.75678700
H	-3.16616000	3.95412400	0.91777600
H	-1.63302000	2.02170300	0.78051100
H	-3.11943700	-2.55655400	-1.52096800
H	-5.38041100	-3.47685300	-1.16908300
H	-6.66641900	-2.90197700	0.88623700
H	-5.66250200	-1.40033000	2.60261100
H	-3.37842500	-0.48058900	2.26903200
O	5.67820800	-0.45803800	-1.39944200

C	5.63763500	-0.39281500	0.00395100
C	4.21004200	-0.72785300	0.41286200
Se	2.98904500	0.61452400	-0.50899100
C	1.99566100	1.34901100	1.01979200
C	1.56165800	0.54562100	2.08358200
C	0.74746700	1.10634000	3.07305900
C	0.36534400	2.45038500	2.99415000
C	0.80651900	3.24202300	1.92989100
C	1.62658800	2.69622700	0.93493400
C	3.81387700	-2.12248100	-0.03283200
H	6.56371900	-0.24344800	-1.72504900
H	6.31286700	-1.13090600	0.48257500
H	5.90861100	0.60345500	0.39818500
H	4.05742200	-0.57908000	1.49069000
H	1.86330200	-0.50118100	2.16316000
H	0.42048300	0.49200200	3.91507400
H	-0.26448200	2.88458500	3.77319800
H	0.52674400	4.29622500	1.87551300
H	1.98436400	3.32538000	0.11622800
H	4.33072600	-2.86149900	0.59936700
H	4.09284100	-2.28338400	-1.08397900
C	-0.52682600	-2.77815000	1.24990100
H	-1.50310300	-3.19866400	1.53142700
H	0.17155400	-3.60697100	1.07638500
H	-0.15538500	-2.16002600	2.07781800
C	0.06515700	0.91064600	-2.66258100
H	-0.67417700	1.66502300	-2.97102400
H	1.06995900	1.34750800	-2.72146200
H	0.00729400	0.05412600	-3.34810400
H	-0.06848300	1.25974200	-0.65155400
H	-0.93039800	-2.56246800	-0.74013500
O	2.38254800	-2.30852900	0.13396500
H	2.16543300	-3.25069300	0.06157900

Int2(*R*)

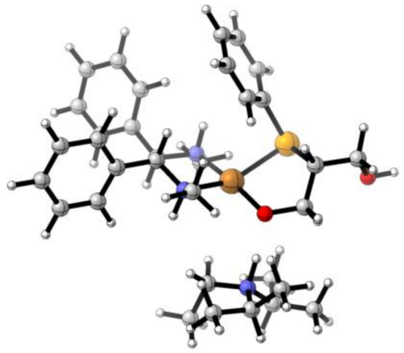

2 2

N	-1.17142100	-0.57853700	1.27454100
N	-0.39431400	1.20261700	-0.68646700
C	-2.09187100	0.55849300	0.96231400
C	-1.85784200	0.99290400	-0.49416600
C	-3.53745500	0.20729700	1.22678500
C	-2.68186300	2.20284300	-0.86934200
C	-4.30717600	0.96177300	2.11866000
C	-5.64799900	0.63749300	2.34282600
C	-6.22620900	-0.44701300	1.67876900
C	-5.46205100	-1.20647700	0.78652100
C	-4.12528800	-0.87837300	0.55828900
C	-2.46236100	3.43591700	-0.23608900
C	-3.23156700	4.55131700	-0.57066400
C	-4.23483600	4.44136200	-1.53921700
C	-4.46223300	3.21621300	-2.17067000
C	-3.68670200	2.10262400	-1.83769800
Cu	0.55259100	-0.38586200	0.24410900
H	-1.79613600	1.38854500	1.62340800
H	-2.13433500	0.15392000	-1.15089000
H	-3.86042100	1.81442500	2.63628800
H	-6.24191300	1.23325700	3.03886500
H	-7.27337600	-0.70117400	1.85519500
H	-5.91229000	-2.05278300	0.26343300
H	-3.54919400	-1.47639100	-0.15398300
H	-1.69444800	3.53716800	0.53881100
H	-3.05635500	5.50641300	-0.07109000
H	-4.84064000	5.31189300	-1.79886100
H	-5.24670500	3.12648900	-2.92482900

H	-3.87221700	1.14362800	-2.32791100
O	4.10344300	-3.70672400	0.25863800
C	3.50306900	-3.63619900	-1.01213100
C	2.52314100	-2.47695600	-0.99606900
Se	1.12856900	-2.81596800	0.44754100
C	-0.48681300	-3.17282200	-0.60406600
C	-0.76416900	-2.50793300	-1.80567200
C	-1.97907200	-2.74157200	-2.45610400
C	-2.91562400	-3.62371500	-1.90524500
C	-2.63383600	-4.27855800	-0.70353300
C	-1.41654200	-4.05908600	-0.04768900
C	3.16617200	-1.13421700	-0.65909700
H	4.69002000	-4.47365400	0.30478700
H	4.24429000	-3.43484600	-1.81294300
H	2.97342100	-4.56770700	-1.28471300
H	1.96504000	-2.42709600	-1.94122000
H	-0.04139700	-1.81876500	-2.24703200
H	-2.19152000	-2.23661200	-3.40121700
H	-3.86126500	-3.80689800	-2.41926100
H	-3.35582500	-4.97697400	-0.27533700
H	-1.19410400	-4.58970900	0.88115900
H	3.86400900	-0.91262200	-1.48932500
H	3.78312800	-1.27560200	0.24722900
C	-0.00412700	1.39933900	-2.09524800
H	-0.48151400	2.29450200	-2.52193000
H	1.08743300	1.47495200	-2.15623100
H	-0.31893400	0.52512600	-2.68056500
C	-1.02477400	-0.81530600	2.72696700
H	-2.00855800	-0.92650800	3.20775400
H	-0.43559900	-1.72603600	2.89555100
H	-0.50070900	0.03437100	3.18718500
H	-1.59599400	-1.41840500	0.86733800
H	-0.13805400	2.04626800	-0.16935200
O	2.25788900	-0.07120600	-0.51726800
H	3.06313700	1.30139500	-0.05333500
N	3.47295000	2.21010400	0.33431100
C	4.46267600	1.81959300	1.44052800

H	4.76728000	2.77450800	1.89209100
C	2.27087100	2.91122900	0.91227800
H	1.65545200	3.21489400	0.05913300
H	1.70674100	2.14361100	1.45854700
C	4.14253800	2.94849200	-0.84748500
H	5.03973100	3.41823000	-0.42022700
C	4.53469600	1.93795300	-1.92531400
H	5.10132500	2.45534300	-2.71201500
H	5.16114000	1.12280200	-1.55008500
H	3.63651000	1.50045700	-2.38629500
C	3.28342100	4.04495800	-1.47482700
H	2.37272100	3.64108200	-1.94284900
H	3.00722500	4.84831600	-0.78158200
H	3.87548600	4.50666700	-2.27779200
C	2.56085900	4.10140800	1.81410700
H	1.61575100	4.62350100	2.02537100
H	2.99161700	3.80462200	2.77970700
H	3.23875300	4.82854500	1.34619600
C	5.71840200	1.12219100	0.92474700
H	5.49284200	0.15555100	0.45349500
H	6.30443600	1.73503100	0.22950900
H	6.36421500	0.92129200	1.79110000
C	3.76494700	0.95136300	2.48861200
H	2.97282000	1.47721700	3.03756200
H	3.34005000	0.04579900	2.02961600
H	4.50697400	0.62979500	3.23189300

Int1(*S*)

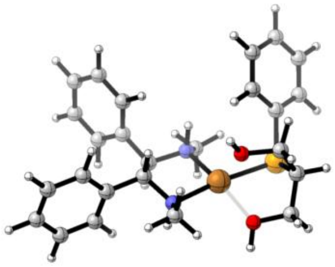

2 2

N	-0.19763000	0.63537800	-0.74525400
N	-1.18622300	-1.85302300	-0.02005700

C	-1.68743900	0.44615900	-0.71984700
C	-2.03653300	-0.65762700	0.28857000
C	-2.41155000	1.74060300	-0.43731200
C	-3.51281300	-0.97585600	0.30178000
C	-3.31815400	2.26458700	-1.36630500
C	-3.99656200	3.45538200	-1.09464100
C	-3.76824100	4.13306700	0.10575200
C	-2.86199200	3.61744400	1.03846600
C	-2.18969700	2.42491700	0.76888500
C	-4.14187600	-1.47540200	-0.84990500
C	-5.50785800	-1.76057500	-0.84042600
C	-6.25895100	-1.53987800	0.31894800
C	-5.64028800	-1.03775300	1.46668800
C	-4.27128000	-0.75976800	1.45823200
Cu	0.68427700	-1.15129600	-0.36453600
H	-1.96464900	0.09312100	-1.72500600
H	-1.73662600	-0.32438200	1.29488700
H	-3.50489800	1.73449200	-2.30359200
H	-4.70553000	3.85450300	-1.82272200
H	-4.29799400	5.06412300	0.31724200
H	-2.68688000	4.14318500	1.97928600
H	-1.49742800	2.02800900	1.51954900
H	-3.57713700	-1.64039200	-1.77378000
H	-5.99002800	-2.14926700	-1.73962000
H	-7.32860900	-1.75900900	0.32556500
H	-6.22501700	-0.86203400	2.37182500
H	-3.79259400	-0.36188200	2.35674900
C	2.97102100	-3.24591800	-0.15493200
C	3.50287200	-1.95369000	0.44989700
Se	3.03075500	-0.51799700	-0.92157700
C	3.41232200	1.15402800	0.00696500
C	2.63034300	1.61795000	1.07407300
C	2.92346400	2.86093200	1.63937400
C	3.97978500	3.63114300	1.13989400
C	4.75118700	3.15870400	0.07531000
C	4.47200700	1.91477900	-0.50039500
C	2.97717400	-1.61157300	1.83002000

H	1.19922300	-1.29802100	2.65091400
H	3.05614900	-4.06260000	0.58068500
H	3.55107500	-3.51704900	-1.05039600
H	4.60090100	-2.00047100	0.48814600
H	1.82145000	1.00909000	1.47889900
H	2.32635400	3.22920300	2.47650000
H	4.20266300	4.60289000	1.58507300
H	5.57757400	3.75703300	-0.31376900
H	5.07592900	1.55023100	-1.33412600
H	3.49144400	-0.71919400	2.22167600
H	3.19588900	-2.45822700	2.50400500
C	-1.29240100	-2.92300400	0.99687200
H	-2.33252900	-3.26538800	1.09596600
H	-0.64904200	-3.76439600	0.71405000
H	-0.95516600	-2.53543700	1.96639200
C	0.25379900	1.35532900	-1.96032600
H	-0.35867900	2.25581000	-2.11618600
H	1.30049600	1.66073700	-1.84504500
H	0.15627200	0.70067900	-2.83693600
H	0.04789700	1.22127600	0.05944900
H	-1.52733500	-2.23707200	-0.90747400
O	1.56371200	-1.38832300	1.75911600
O	1.58028000	-3.07071800	-0.51323700
H	1.30211700	-3.75812900	-1.13672500

Int2(*S*)

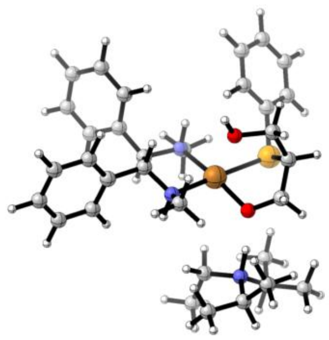

2 2

N	1.30876500	0.65533100	0.77130100
N	0.40539900	-1.54514300	-0.64158500
C	2.18999000	-0.55295100	0.71190300

C	1.87903800	-1.34439400	-0.57032900
C	3.65517200	-0.20134900	0.82745800
C	2.66153700	-2.63628100	-0.64226900
C	4.44504600	-0.75716600	1.83985500
C	5.80423300	-0.44482200	1.92824000
C	6.38300300	0.42963300	1.00551600
C	5.60082500	0.99067600	-0.00935800
C	4.24494000	0.67314600	-0.09880300
C	2.44087300	-3.65229000	0.30043200
C	3.17400600	-4.83868300	0.24859400
C	4.14233600	-5.01753400	-0.74485300
C	4.37074200	-4.00912100	-1.68405700
C	3.63116800	-2.82470500	-1.63305700
Cu	-0.44579000	0.26494000	-0.16473000
H	1.90664400	-1.18045400	1.57154300
H	2.14584400	-0.72035200	-1.43766700
H	3.99821200	-1.44723700	2.55991700
H	6.41227400	-0.88565900	2.72076200
H	7.44481900	0.67449400	1.07491800
H	6.05200200	1.67097300	-0.73465400
H	3.65221500	1.10972200	-0.91005800
H	1.69988400	-3.52445000	1.09709600
H	2.99720300	-5.62314700	0.98739800
H	4.71980800	-5.94339700	-0.78408000
H	5.12765500	-4.14412500	-2.45932800
H	3.81864700	-2.03541500	-2.36545100
C	-2.91434800	0.74574400	-1.53731100
C	-2.22076300	2.11423300	-1.65432500
Se	-1.57492900	2.47891400	0.24483500
C	-0.29721200	3.93918400	0.04865400
C	0.92168300	3.78871600	-0.62739600
C	1.82718500	4.85190600	-0.65233500
C	1.52178200	6.05282800	-0.00347000
C	0.30476800	6.19513200	0.66740600
C	-0.61200200	5.13915800	0.69712900
C	-1.10357100	2.18834000	-2.67371800
H	0.54621900	1.18863800	-3.08866300

H	-3.05149800	0.36708600	-2.57016100
H	-3.92951000	0.91606600	-1.13019100
H	-2.95184200	2.90052900	-1.89156900
H	1.15073100	2.86200400	-1.15423700
H	2.77551500	4.74246000	-1.18316600
H	2.23373600	6.88034000	-0.02393000
H	0.06116500	7.13296800	1.17086900
H	-1.56236900	5.25525600	1.22244700
H	-0.68818700	3.20882500	-2.71746300
H	-1.54134200	1.95777300	-3.66136700
C	-0.06216600	-2.10763700	-1.92152200
H	0.39595800	-3.08796900	-2.12129100
H	-1.15462000	-2.18654300	-1.89172400
H	0.20010000	-1.41615900	-2.73177400
C	1.21696900	1.20937200	2.14077200
H	2.21899600	1.32710200	2.58044300
H	0.72762500	2.19011300	2.11100000
H	0.62745100	0.53197100	2.77437000
H	1.74357400	1.37757200	0.19043500
H	0.15034800	-2.19886300	0.10216300
O	-0.08095000	1.24026900	-2.35454400
O	-2.22346100	-0.18174100	-0.74421100
H	-3.12449100	-1.26471700	0.02359000
N	-3.58820000	-2.01803900	0.63436000
C	-4.54965400	-1.29574100	1.58313000
H	-4.92659300	-2.07995800	2.25579600
C	-2.42073900	-2.60444800	1.38377000
H	-1.83109200	-3.15776200	0.64526400
H	-1.80896800	-1.75072900	1.70463400
C	-4.30692100	-2.99971900	-0.31771900
H	-5.24067800	-3.26656500	0.19705400
C	-4.61524400	-2.29721400	-1.64028400
H	-5.24986200	-2.95123200	-2.25463700
H	-5.14243100	-1.34591200	-1.51669400
H	-3.68709500	-2.10636200	-2.19878700
C	-3.53792300	-4.28886000	-0.60115600
H	-2.59745600	-4.10099200	-1.14110900

H	-3.32681900	-4.88860100	0.29202500
H	-4.16465200	-4.90686100	-1.25990100
C	-2.76499000	-3.48998500	2.57198100
H	-1.84696000	-3.98345000	2.92439000
H	-3.17389000	-2.92085500	3.41750800
H	-3.48307300	-4.28049700	2.31429000
C	-5.74429400	-0.66585500	0.87240800
H	-5.43497900	0.13094200	0.17999300
H	-6.36669100	-1.38877200	0.33185100
H	-6.38392400	-0.20448400	1.63772900
C	-3.80210500	-0.23411600	2.38995300
H	-3.04299400	-0.64825500	3.06612100
H	-3.32156300	0.49586400	1.72191900
H	-4.52419300	0.30828400	3.01522800

TS1-*R*

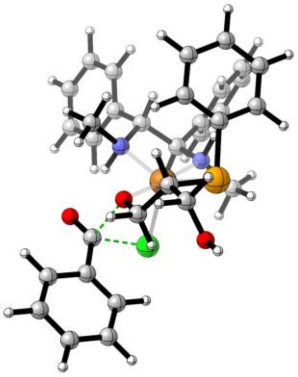

1 2

N	-26.85233500	2.01972500	0.68572900
N	-27.53171200	1.53983600	-1.95800600
C	-26.46703100	0.70870300	0.09730200
C	-26.32305400	0.87644600	-1.42828500
C	-25.20371400	0.14305800	0.70835100
C	-26.01498500	-0.44495800	-2.09975200
C	-25.18602300	-1.15251100	1.23490900
C	-24.00720300	-1.68738800	1.76098300
C	-22.83563000	-0.92683700	1.76878400
C	-22.84539400	0.37037400	1.24621700
C	-24.02225300	0.90031000	0.71523400
C	-26.99131000	-1.45020500	-2.15997200

C	-26.70154000	-2.68311000	-2.74682600
C	-25.42932100	-2.92509800	-3.27449200
C	-24.45152400	-1.92915800	-3.21556700
C	-24.74547600	-0.69444000	-2.63186300
Cu	-28.27882400	2.83717500	-0.48953100
H	-27.30740700	0.02631400	0.29446300
H	-25.48570100	1.56930700	-1.60908300
H	-26.09991200	-1.75131700	1.22369600
H	-24.00495000	-2.70136900	2.16648400
H	-21.91436700	-1.34362600	2.18139700
H	-21.93134100	0.96841300	1.24837600
H	-24.00711200	1.91238600	0.29923600
H	-27.98971300	-1.28068800	-1.74470600
H	-27.46977500	-3.45813800	-2.79045000
H	-25.20160500	-3.89024300	-3.73206700
H	-23.45624100	-2.11341200	-3.62576800
H	-23.97681200	0.08062200	-2.57854700
O	-30.38963600	7.14501400	0.32211400
C	-29.54822500	7.26094700	-0.80113300
C	-29.00721900	5.88065000	-1.12679800
Se	-27.98968500	5.17726000	0.48357900
C	-26.14584300	5.07474900	-0.16772100
C	-25.82504100	4.80340600	-1.50226500
C	-24.49176400	4.59080000	-1.86294800
C	-23.48124300	4.63580500	-0.89637100
C	-23.80697000	4.91037500	0.43440100
C	-25.13824000	5.13672100	0.80303700
C	-30.09769700	4.85646200	-1.43586100
H	-30.72417300	8.01645900	0.56923100
H	-30.09925900	7.62314600	-1.69355500
H	-28.70657000	7.95737100	-0.62731000
H	-28.27635900	5.94009900	-1.94483700
H	-26.60532000	4.73640300	-2.26116700
H	-24.24327600	4.38395200	-2.90655500
H	-22.44149900	4.46530100	-1.18254900
H	-23.02346100	4.95964600	1.19364000
H	-25.38532200	5.36275100	1.84319400

H	-30.65127000	5.22223200	-2.32213600
H	-30.81591700	4.85066200	-0.59777500
C	-33.95693200	3.81029400	-0.44550100
C	-34.63985500	4.00546000	-1.64977200
C	-34.09166100	3.54484900	-2.85169900
C	-32.86096400	2.89234100	-2.85242500
C	-32.17315500	2.70249800	-1.63784600
C	-32.72220200	3.16248900	-0.43341000
C	-30.87328400	2.03611100	-1.76511300
Cl	-30.18202800	1.45053200	0.28163700
O	-30.40550400	1.30745300	-2.55534700
H	-34.38998100	4.16099600	0.49326100
H	-35.60737300	4.51234600	-1.65183200
H	-34.62874900	3.68738400	-3.79166400
H	-32.42617400	2.52306200	-3.78249800
H	-32.18644200	2.99494000	0.49919900
C	-27.37837900	2.04225100	-3.33038300
H	-27.19992700	1.21984800	-4.04135600
H	-28.28858300	2.58496700	-3.60922400
H	-26.52125600	2.72752200	-3.37230300
C	-27.20642700	1.93386400	2.11521900
H	-26.39289500	1.47698500	2.69870200
H	-27.40915600	2.94182300	2.50026900
H	-28.11782600	1.33205900	2.22301300
H	-26.05137400	2.65109200	0.59348900
H	-28.31299600	0.87810300	-1.96319300
O	-29.58032800	3.58500700	-1.70290700

TS1-*R*-I

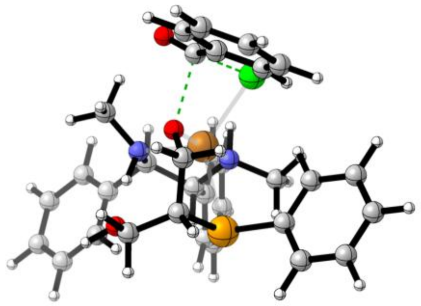

1 2

N	-28.04505900	1.22790600	0.72948200

N	-29.38416600	-0.14666600	-1.23531800
C	-27.30608200	0.15447300	0.03512700
C	-27.90621000	-0.05956200	-1.37123800
C	-25.81382200	0.40269300	-0.03275500
C	-27.28401800	-1.25117700	-2.06544100
C	-24.90891800	-0.50422200	0.52830200
C	-23.53208400	-0.27805600	0.44402600
C	-23.04789000	0.85999100	-0.20431000
C	-23.94536500	1.77220700	-0.76932300
C	-25.31958000	1.54397200	-0.68445800
C	-27.50389100	-2.55170000	-1.58808200
C	-26.90301700	-3.64683300	-2.21080000
C	-26.06730100	-3.45142300	-3.31489600
C	-25.84015500	-2.15916000	-3.79388600
C	-26.44864000	-1.06583000	-3.17248700
Cu	-29.95474300	1.42079700	-0.08064200
H	-27.48166600	-0.76912000	0.60944000
H	-27.71210000	0.84053200	-1.97480200
H	-25.28361500	-1.40092100	1.02804600
H	-22.83629100	-0.99495000	0.88523000
H	-21.97228400	1.03639000	-0.27245800
H	-23.57015400	2.65998200	-1.28386400
H	-26.01287900	2.25925100	-1.13586600
H	-28.14806800	-2.72406000	-0.72014900
H	-27.08410400	-4.65503000	-1.83198100
H	-25.59426200	-4.30742400	-3.80084000
H	-25.18744600	-2.00061600	-4.65498900
H	-26.26424200	-0.05512300	-3.54501900
O	-27.66654000	3.98044900	-0.06508600
C	-28.58127900	5.07134500	-0.11332000
C	-29.99037900	4.61393500	-0.46589400
C	-30.85534900	4.03178000	0.65060700
H	-26.77342700	4.32688900	0.06508900
H	-28.62687200	5.58709200	0.86421700
H	-28.24520400	5.80165400	-0.86939200
H	-30.52219900	5.47539000	-0.89730800
H	-31.89252900	3.97978800	0.26310000

H	-30.87346400	4.77715900	1.47259100
C	-34.86334900	3.63122800	1.37138200
C	-34.98243800	4.45095100	2.49767500
C	-34.07942900	4.32415400	3.55953900
C	-33.05595300	3.38234000	3.49758200
C	-32.93804500	2.55916600	2.35865900
C	-33.84279400	2.68534400	1.29589800
C	-31.80666200	1.64078800	2.38781300
Cl	-31.96303600	0.25782700	0.63439400
O	-31.09066300	1.19025800	3.18243800
H	-35.56804700	3.72805000	0.54333000
H	-35.78467600	5.19016200	2.55167600
H	-34.17632600	4.95904400	4.44251400
H	-32.34644600	3.27441400	4.31931200
H	-33.74294900	2.04074000	0.42705600
C	-30.10039200	-0.31480800	-2.51169700
H	-29.79212600	-1.23637500	-3.02758900
H	-31.17944900	-0.34936100	-2.31444700
H	-29.88617400	0.54105100	-3.16539600
C	-27.95818200	1.16883000	2.19417900
H	-26.90967100	1.19398700	2.53614100
H	-28.50798500	2.02186700	2.60794200
H	-28.43104000	0.24646900	2.55985400
H	-27.71012800	2.15127600	0.43121500
H	-29.61398700	-0.94594400	-0.63845300
O	-30.42178000	2.80799600	1.14467000
Se	-29.86995600	3.26742900	-1.98079700
C	-31.71723500	3.44779200	-2.59338300
C	-32.04341500	4.51630200	-3.43700700
C	-32.69114100	2.52076400	-2.21069100
C	-33.35927400	4.66376500	-3.88399400
H	-31.27591100	5.22851100	-3.74833500
C	-34.00353400	2.67193700	-2.66935400
H	-32.44024000	1.69158200	-1.54886700
C	-34.33948600	3.74286600	-3.50134500
H	-33.61588900	5.49770200	-4.54094900
H	-34.76413500	1.94589000	-2.37219400

H	-35.36516800	3.85782400	-3.85841500

TS1-*R*-II

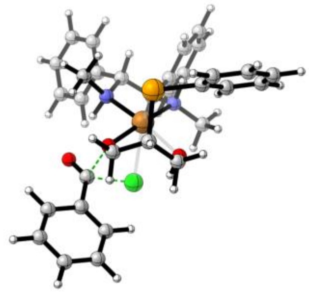

1 2

N	1.17189200	1.07377800	0.88495000
N	1.03165400	-0.77721800	-1.15683600
C	2.37562300	0.22757100	0.63580200
C	2.36303400	-0.21312900	-0.84032200
C	3.67548900	0.90516800	1.00637800
C	3.49716400	-1.16748800	-1.14469300
C	4.56603800	0.28505600	1.88950900
C	5.78655500	0.88735300	2.20467300
C	6.12470700	2.12028000	1.64189500
C	5.23976200	2.74877000	0.76012900
C	4.02424900	2.14102500	0.44184900
C	3.49339000	-2.45951200	-0.59722600
C	4.55421700	-3.33243300	-0.84344700
C	5.63155900	-2.92012300	-1.63397900
C	5.64200800	-1.63418200	-2.17944200
C	4.57752800	-0.76271800	-1.93602800
Cu	-0.38179600	0.07160000	0.04007200
H	2.24318700	-0.66771500	1.26115900
H	2.47905800	0.68819100	-1.46341200
H	4.30574600	-0.68238900	2.32570900
H	6.47487200	0.39158600	2.89243000
H	7.07786500	2.59268800	1.88879200
H	5.50034400	3.71177100	0.31569200
H	3.35101100	2.64199300	-0.26139400
H	2.66163600	-2.79716200	0.02846600
H	4.54089200	-4.33697700	-0.41520000
H	6.46231500	-3.60281300	-1.82509200

H	6.48097500	-1.30746200	-2.79755100
H	4.59167400	0.24568200	-2.35696400
C	-4.55602900	-3.74615600	1.79569700
C	-5.04687500	-4.64058100	0.83904100
C	-4.26657200	-4.97627200	-0.27180200
C	-2.99930100	-4.41821300	-0.43067900
C	-2.50937300	-3.51423800	0.53003000
C	-3.29084900	-3.17930100	1.64390900
C	-1.18664100	-2.94644500	0.23156400
Cl	-0.42304400	-1.93112800	2.00114900
O	-0.28667800	-3.32024800	-0.42868200
H	-5.15894800	-3.49538600	2.67079700
H	-6.03763600	-5.08297400	0.96348400
H	-4.64388200	-5.68094600	-1.01566800
H	-2.38151200	-4.67452700	-1.29258900
H	-2.89163300	-2.50009400	2.39507900
C	0.71521500	-0.79631800	-2.59315200
H	1.48436700	-1.34529300	-3.15944500
H	-0.25858000	-1.28194200	-2.72620900
H	0.65825000	0.23360200	-2.97253200
C	1.01043800	1.43247200	2.30826100
H	1.89197300	1.97371500	2.68250300
H	0.11112800	2.04699900	2.42951300
H	0.88069200	0.51135100	2.89114200
H	1.27512400	1.93736800	0.34539400
H	0.97882300	-1.74542500	-0.82179000
O	-1.81561100	0.70285400	1.47644700
C	-3.14539700	0.92267100	0.98120200
C	-3.26400900	0.64262400	-0.50905900
C	-3.00168200	-0.81518600	-0.88650300
H	-1.75585800	-0.13282200	1.98282300
H	-3.84963600	0.26934400	1.52246000
H	-3.40958300	1.96634400	1.20186100
H	-4.26573700	0.94543000	-0.84824200
H	-3.22597300	-0.96422600	-1.96026700
H	-3.72730600	-1.43503900	-0.32350800
O	-1.68971900	-1.21135100	-0.59963400

Se	-1.92932000	1.75999600	-1.57292600
C	-2.26295200	3.48805900	-0.73358500
C	-1.48531000	3.91564000	0.34907700
C	-3.27974100	4.30627500	-1.23996400
C	-1.73079300	5.16203900	0.93100600
H	-0.70040300	3.27270300	0.74625200
C	-3.52136700	5.55110900	-0.65140200
H	-3.87704800	3.97612000	-2.09242400
C	-2.74924400	5.97914000	0.43228300
H	-1.12508900	5.49428000	1.77717000
H	-4.31434200	6.19000800	-1.04620300
H	-2.93988200	6.95324400	0.88764600

TS1-*R*-III

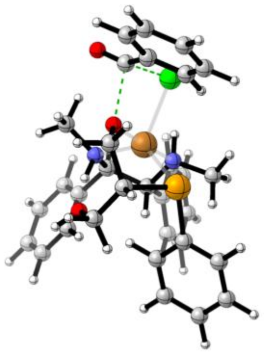

1 2

N	-27.57619900	1.23228500	0.70173100
N	-28.38883900	0.22748600	-1.71690300
C	-26.91444000	-0.00012400	0.22939200
C	-26.99018400	-0.06679200	-1.31114300
C	-25.48795700	-0.14553800	0.71641800
C	-26.46254000	-1.37852100	-1.84705200
C	-25.08214400	-1.28791900	1.41414100
C	-23.75648600	-1.43195000	1.83262500
C	-22.82200000	-0.43152100	1.55672500
C	-23.21777600	0.71475400	0.86000100
C	-24.54194600	0.85434300	0.44192300
C	-27.11522800	-2.58606400	-1.55911700
C	-26.61058700	-3.79564700	-2.03913700
C	-25.44217400	-3.81034100	-2.80767500
C	-24.78609600	-2.61180900	-3.09789200

C	-25.29751200	-1.40214800	-2.62158400
Cu	-28.99111100	1.86520100	-0.67927400
H	-27.50674100	-0.84005800	0.62497800
H	-26.37851600	0.74583600	-1.72652300
H	-25.80807100	-2.07696400	1.62470900
H	-23.45362200	-2.32952300	2.37603400
H	-21.78594300	-0.54334600	1.88336800
H	-22.48945100	1.49868500	0.63970000
H	-24.83747800	1.75227300	-0.10777600
H	-28.02692400	-2.59418000	-0.95378800
H	-27.12792500	-4.72996000	-1.81072500
H	-25.04516700	-4.75689600	-3.18061500
H	-23.87378600	-2.61815500	-3.69823500
H	-24.78448000	-0.46416600	-2.84855800
O	-26.53334400	3.91379000	0.12016000
C	-27.00206200	5.08880300	-0.53043600
C	-28.45792000	5.00233800	-0.94274100
C	-29.46855700	4.65747800	0.16589300
H	-25.70685900	4.12905900	0.57310200
H	-26.93526400	5.95120700	0.15870800
H	-26.37639900	5.32343900	-1.41017400
H	-28.71076500	5.98677300	-1.36361100
H	-30.44023000	5.09736200	-0.13118600
H	-29.14208700	5.20241600	1.07501700
C	-33.32509800	4.68725800	-1.49012400
C	-33.96783800	5.58473000	-0.62903100
C	-33.80339600	5.47088000	0.75431500
C	-32.99603000	4.46345800	1.28137000
C	-32.35319900	3.56205900	0.41249000
C	-32.51789900	3.67556500	-0.97544800
C	-31.48944200	2.58022700	1.06824700
Cl	-31.20851400	0.88498600	-0.37820000
O	-31.30295300	2.21473500	2.15549200
H	-33.46054400	4.77248800	-2.57012100
H	-34.60335100	6.37277400	-1.03894200
H	-34.30864200	6.16601200	1.42785800
H	-32.85964900	4.36499900	2.35942600

H	-32.02386600	2.96250100	-1.63231200
C	-28.61429100	0.22869800	-3.17257600
H	-28.40690200	-0.75898500	-3.61093400
H	-29.66004500	0.50114700	-3.36840100
H	-27.95168100	0.96538200	-3.64404800
C	-28.04360900	1.17094300	2.09364300
H	-27.21721700	0.94205000	2.78707800
H	-28.49899000	2.13433600	2.34918200
H	-28.81698100	0.39568200	2.18689000
H	-26.95632900	2.04033900	0.59917400
H	-29.00763100	-0.47601600	-1.30368400
O	-29.61038700	3.30047600	0.44188500
Se	-28.88936100	3.69525700	-2.44770600
C	-27.23902100	3.56926400	-3.47761800
C	-26.04239800	3.10388900	-2.91895400
C	-27.31670600	3.86289400	-4.84411400
C	-24.92156200	2.93108100	-3.73413400
H	-25.98566800	2.89445500	-1.85205800
C	-26.19167100	3.67646000	-5.65420600
H	-28.24886000	4.23178600	-5.27713900
C	-24.99581600	3.20999200	-5.10266100
H	-23.98685000	2.57238700	-3.29619100
H	-26.25316000	3.90204200	-6.72097100
H	-24.11978400	3.06713700	-5.73871200

TS1-*R*-IV

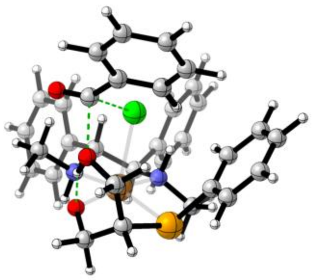

1 2

N	-26.99131900	1.39839100	1.18438700
N	-27.53292700	1.11773700	-1.52886600
C	-26.97733800	0.02849900	0.59713000
C	-26.63792900	0.11984600	-0.90167500

C	-26.02944000	-0.90114500	1.32373000
C	-26.68126200	-1.24115700	-1.56319000
C	-26.48305800	-2.10992900	1.86207400
C	-25.59467900	-2.97791300	2.50238400
C	-24.24374400	-2.64048500	2.61165500
C	-23.78236700	-1.43341600	2.07664900
C	-24.66993100	-0.57003400	1.43284200
C	-27.89208700	-1.93680600	-1.69311000
C	-27.92359000	-3.20490700	-2.27569000
C	-26.73961800	-3.79455100	-2.72926900
C	-25.52906600	-3.10932700	-2.60127700
C	-25.50213200	-1.83809100	-2.02277000
Cu	-27.60934700	2.74107600	-0.25429700
H	-28.00406600	-0.35966000	0.69478700
H	-25.62340600	0.53246300	-0.99723000
H	-27.53787700	-2.38053700	1.76997400
H	-25.95961700	-3.92033400	2.91629500
H	-23.54870200	-3.31802800	3.11222500
H	-22.72599500	-1.16739900	2.15537800
H	-24.29058200	0.36307100	1.00403800
H	-28.82820800	-1.49550300	-1.33623900
H	-28.87292900	-3.73594400	-2.37371100
H	-26.76235900	-4.78759800	-3.18318800
H	-24.60147200	-3.56501400	-2.95432000
H	-24.55402200	-1.30489100	-1.91930200
O	-28.55619300	3.74260300	-1.61552200
C	-29.41671600	4.79123400	-1.25434900
C	-28.67125400	5.78856000	-0.35842900
Se	-28.30469000	4.66796200	1.30148200
C	-26.79760200	5.58751700	2.12030000
C	-25.65893800	4.87509100	2.50577700
C	-24.62267900	5.54146200	3.16669500
C	-24.71815700	6.91075200	3.42687900
C	-25.85828100	7.61798800	3.03236700
C	-26.90772300	6.95721300	2.38956100
C	-27.44285200	6.38167900	-1.05421900
H	-27.44638100	4.62301900	-2.00324000

H	-29.74905400	5.32818700	-2.16475000
H	-30.33543000	4.42991700	-0.75327300
H	-29.32592500	6.60157300	-0.01511900
H	-25.55513900	3.81957100	2.26419200
H	-23.73214600	4.98505700	3.46650000
H	-23.90551600	7.42746800	3.94200700
H	-25.94095300	8.68738400	3.23794800
H	-27.80739900	7.50987300	2.11059600
H	-26.68708800	6.72130100	-0.32905100
H	-27.78486500	7.27564700	-1.60675400
C	-23.39445000	7.48497900	-0.03898200
C	-22.76635300	8.28549100	-0.99912100
C	-22.86999400	7.97001800	-2.35762700
C	-23.60566900	6.85804200	-2.76195000
C	-24.24114900	6.06027300	-1.78926800
C	-24.13417700	6.37013500	-0.42689600
C	-25.01747100	4.95204900	-2.33010000
Cl	-25.12371400	3.32226100	-0.51094800
O	-25.08517700	4.32162000	-3.29818900
H	-23.30475100	7.72203600	1.02199900
H	-22.18569500	9.15640500	-0.68706200
H	-22.37278400	8.58931000	-3.10679100
H	-23.69447700	6.60233400	-3.81894800
H	-24.60143300	5.72153700	0.30905400
C	-27.14683900	1.47014300	-2.90799300
H	-27.11515400	0.57542600	-3.54886200
H	-27.87257200	2.19169800	-3.29905300
H	-26.15544800	1.94001800	-2.88863100
C	-27.61194600	1.42767700	2.51963200
H	-27.18462000	0.65236400	3.17480200
H	-27.45134500	2.40816700	2.98450700
H	-28.69408200	1.25803600	2.42740300
H	-26.01713900	1.70325500	1.26537700
H	-28.48809000	0.75273700	-1.54587600
O	-26.85914800	5.48361200	-1.98597200

TS2-*S*

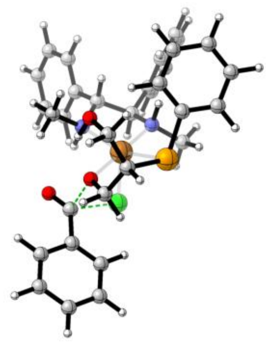

1 2

N	-27.10702600	1.40299800	1.49078500
N	-28.01809000	0.94540000	-1.09926800
C	-26.59154000	0.20883800	0.77093600
C	-26.66088100	0.47152800	-0.74453000
C	-25.19165700	-0.17629200	1.19844400
C	-26.23038900	-0.75136500	-1.52727300
C	-24.90287400	-1.49367900	1.57024500
C	-23.60085500	-1.86279600	1.91664900
C	-22.57585600	-0.91384500	1.89900900
C	-22.85634700	0.40594800	1.53191000
C	-24.15683300	0.77080400	1.17980300
C	-27.03090800	-1.90329000	-1.52844100
C	-26.62186100	-3.04747300	-2.21529400
C	-25.40416800	-3.05280100	-2.90287900
C	-24.60114800	-1.90977900	-2.90342800
C	-25.01488200	-0.76404700	-2.21939700
Cu	-28.77364700	2.03467100	0.52653500
H	-27.28457900	-0.61150200	1.00967400
H	-25.97216400	1.30030600	-0.97552700
H	-25.70173900	-2.23896700	1.57687900
H	-23.38722800	-2.89503300	2.20197500
H	-21.55813300	-1.20112800	2.17169600
H	-22.05855100	1.15173000	1.51671100
H	-24.35409700	1.80714200	0.88662300
H	-27.98236600	-1.91976000	-0.98750100
H	-27.25363300	-3.93830300	-2.21181500
H	-25.08278700	-3.94846300	-3.43880400
H	-23.64930600	-1.90885000	-3.43887200
H	-24.38133100	0.12639200	-2.21419300

C	-31.06379700	3.78359200	0.08343700
C	-30.08590300	4.90474200	0.44407800
Se	-29.01495200	4.07729200	1.97021200
C	-27.20100400	4.79368700	1.87243500
C	-26.38574000	4.62252500	0.74442900
C	-25.05319200	5.04210000	0.79757700
C	-24.53688000	5.61734300	1.96353200
C	-25.35889300	5.78815400	3.08003200
C	-26.69617000	5.38054300	3.03888800
C	-29.25197700	5.40535000	-0.72653700
H	-31.76967000	4.17036700	-0.67557900
H	-31.67025500	3.52404200	0.97007300
H	-30.62816400	5.74798900	0.89633200
H	-26.79790200	4.19992500	-0.17389700
H	-24.41818900	4.92378000	-0.08364500
H	-23.49419200	5.93985400	1.99884700
H	-24.96301800	6.24466600	3.98970000
H	-27.33776100	5.51751200	3.91205000
H	-28.49933700	6.13315600	-0.38589500
H	-29.94869700	5.95658000	-1.39081700
C	-34.51014700	1.85073100	1.37929600
C	-35.38616100	2.08345500	0.31451500
C	-34.94562200	1.93068300	-1.00432100
C	-33.63054400	1.54890500	-1.26132300
C	-32.74893300	1.32301500	-0.18613700
C	-33.18971100	1.47366700	1.13561700
C	-31.38333900	0.95912100	-0.58048500
Cl	-30.30107300	0.22074400	1.29401000
O	-30.91707600	0.42336600	-1.51120800
H	-34.85884500	1.95658200	2.40846000
H	-36.41929900	2.37712500	0.51274700
H	-35.63146100	2.10089800	-1.83672700
H	-33.27906200	1.41865600	-2.28610200
H	-32.50143100	1.27302500	1.95494400
C	-28.08592500	1.50095300	-2.46082000
H	-27.71884300	0.77428500	-3.20275200
H	-29.12905000	1.74287200	-2.69386800

H	-27.47748600	2.41265100	-2.51945200
C	-27.28788800	1.15875300	2.93559500
H	-26.37171100	0.74250200	3.38026500
H	-27.53199100	2.10140400	3.44056400
H	-28.11981000	0.45708900	3.07752800
H	-26.43162900	2.16481700	1.37761600
H	-28.66796200	0.15537600	-1.05692500
O	-28.58202100	4.38687900	-1.42942600
O	-30.39075000	2.65429000	-0.42983900
H	-29.21313100	3.65132900	-1.53694100

TS2-*S*-I

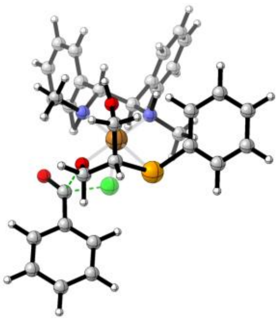

1 2

N	-26.99926900	1.74246800	1.27667800
N	-27.17147000	1.43483700	-1.47772600
C	-26.75101800	0.38501300	0.70753800
C	-26.25905500	0.53004300	-0.74643400
C	-25.79498500	-0.44405400	1.53606700
C	-26.10806600	-0.82236800	-1.40988600
C	-26.16954900	-1.71675400	1.98103900
C	-25.27588200	-2.50476400	2.71043500
C	-23.99826500	-2.02363300	3.00620400
C	-23.61535500	-0.75223000	2.56776300
C	-24.50860900	0.02947600	1.83338200
C	-27.24622200	-1.59567900	-1.68532200
C	-27.11703800	-2.85691200	-2.26873000
C	-25.84891500	-3.35972600	-2.57691300
C	-24.71225700	-2.59571600	-2.30220200
C	-24.84314800	-1.33139800	-1.72199900
Cu	-28.01644700	2.76496900	-0.14695200
H	-27.73283700	-0.10810300	0.68562800

H	-25.27488200	1.02616500	-0.72369300
H	-27.16603000	-2.09729400	1.74446500
H	-25.57938700	-3.49772000	3.04884400
H	-23.29945300	-2.63835700	3.57755000
H	-22.61665200	-0.37242700	2.79418800
H	-24.18555600	1.01629500	1.48577600
H	-28.24476000	-1.21977400	-1.44134900
H	-28.00891000	-3.45025600	-2.48139500
H	-25.74804100	-4.34739400	-3.03202100
H	-23.71975200	-2.98418500	-2.54056300
H	-23.95203900	-0.73879400	-1.49998000
O	-29.07359200	3.57062800	-1.55604000
C	-29.32429800	4.94416200	-1.61568500
C	-28.59747000	5.73352300	-0.51817900
Se	-29.01548100	4.69044400	1.17925500
C	-27.75575500	5.44301300	2.46355700
C	-26.38175300	5.17586700	2.40069600
C	-25.53775600	5.67599300	3.39498000
C	-26.06096100	6.43002600	4.45014900
C	-27.43209000	6.69074400	4.50905100
C	-28.28514700	6.19895700	3.51609700
C	-27.10717900	5.92307000	-0.72750500
H	-28.99953400	5.35834600	-2.59162300
H	-30.40780200	5.14660800	-1.52887700
H	-29.05507400	6.72658400	-0.39935600
H	-25.97599200	4.60424300	1.56557200
H	-24.46508400	5.47469800	3.34568300
H	-25.39779800	6.81508800	5.22757200
H	-27.84455100	7.28009500	5.33073900
H	-29.35714300	6.40166600	3.56432700
H	-26.69356600	6.57412600	0.06254400
H	-26.97910100	6.44364900	-1.69423700
C	-33.39745700	4.24764400	-0.81379000
C	-34.00364700	4.51421900	-2.04679600
C	-33.44711600	4.00982500	-3.22568500
C	-32.28419300	3.24139800	-3.17658200
C	-31.67753900	2.97536600	-1.93625200

C	-32.23528200	3.48078000	-0.75363000
C	-30.42884100	2.20326100	-1.99627000
Cl	-30.14677500	1.24696700	-0.02166700
O	-29.92289000	1.54379300	-2.82614700
H	-33.83832600	4.63320000	0.10774900
H	-34.91691500	5.11195100	-2.08744700
H	-33.92280000	4.21007700	-4.18795600
H	-31.84097300	2.84006500	-4.08929200
H	-31.76365700	3.25084200	0.20037000
C	-26.60661700	1.98998400	-2.71356700
H	-26.29938700	1.19070100	-3.40709200
H	-27.36753100	2.62158900	-3.18561200
H	-25.72917800	2.60553800	-2.47170100
C	-27.66811500	1.68633700	2.59365600
H	-27.14156200	1.00024900	3.27379800
H	-27.68201900	2.68855200	3.03796700
H	-28.69977000	1.33893600	2.45108200
H	-26.09665300	2.20816700	1.39798300
H	-28.02165500	0.92264800	-1.73052300
O	-26.43496300	4.66750700	-0.74539200
H	-25.55748000	4.78319900	-1.13172300

TS2-*S*-II

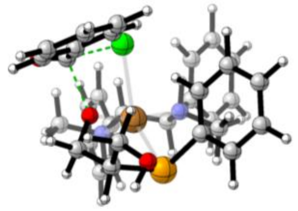

1 2

N	-26.74876500	1.98549700	0.54962600
N	-27.95864700	0.90685800	-1.73703800
C	-26.29477800	0.65300600	0.08971400
C	-26.54221500	0.54782200	-1.42735500
C	-24.85279500	0.34289100	0.43354000
C	-26.16983100	-0.81844000	-1.96053400
C	-24.53286700	-0.80801500	1.16192700
C	-23.20205100	-1.11504800	1.45586300

C	-22.17722800	-0.26888500	1.02593300
C	-22.48725900	0.88541200	0.30004800
C	-23.81769100	1.18616700	0.00389900
C	-26.88409800	-1.95252100	-1.54438600
C	-26.53285000	-3.21975100	-2.01175500
C	-25.45817200	-3.36673500	-2.89453100
C	-24.74011400	-2.24267500	-3.30902100
C	-25.09674200	-0.97408300	-2.84440700
Cu	-28.64746300	2.22729700	-0.35438200
H	-26.94476200	-0.07595400	0.59604900
H	-25.91971300	1.30717600	-1.92696300
H	-25.33285200	-1.47339300	1.49564400
H	-22.96570700	-2.01749100	2.02367100
H	-21.13658300	-0.50688600	1.25631500
H	-21.68980800	1.55039000	-0.03870300
H	-24.03969800	2.08928900	-0.57373800
H	-27.71704200	-1.85718100	-0.84042200
H	-27.09493300	-4.09592900	-1.68145800
H	-25.18062800	-4.35859400	-3.25763500
H	-23.89873800	-2.35245900	-3.99652000
H	-24.52786100	-0.09731900	-3.16374000
O	-29.52307600	3.16975000	1.02661800
C	-29.53191800	4.55809300	1.03772500
C	-30.17449800	5.16366100	-0.21809600
Se	-29.38460100	4.19157300	-1.84103100
C	-30.95992000	3.37769500	-2.66170100
C	-31.49351500	2.18422000	-2.16718100
C	-32.59846200	1.61036900	-2.80202800
C	-33.16056700	2.22544300	-3.92393900
C	-32.61793000	3.41757200	-4.41244300
C	-31.51685100	4.00139200	-3.78142100
C	-31.69752100	5.13774500	-0.23336700
H	-30.10074300	4.92175600	1.91707800
H	-28.50472300	4.96187200	1.15063800
H	-29.82953800	6.20142100	-0.35287400
H	-31.05751200	1.69727400	-1.29477000
H	-33.01605700	0.67685400	-2.41719000

H	-34.02242300	1.77447000	-4.42041500
H	-33.05670500	3.90229300	-5.28710500
H	-31.10279000	4.93943200	-4.15374600
H	-32.03917500	5.54725600	0.73763500
H	-32.06427000	4.10411800	-0.27970900
C	-33.96587100	2.34605200	0.96763700
C	-34.41430700	3.35757200	1.82273700
C	-33.57663800	3.84598600	2.83062600
C	-32.28822200	3.33518000	2.97823600
C	-31.83309300	2.33409300	2.09714100
C	-32.67756900	1.83105700	1.10069600
C	-30.45310800	1.89771300	2.33150300
Cl	-29.91278800	0.33915400	0.77148400
O	-29.75418200	1.79422200	3.25317200
H	-34.62347300	1.95474500	0.18925100
H	-35.42327600	3.75975200	1.70992700
H	-33.93032500	4.62307100	3.51136900
H	-31.63313400	3.69991400	3.77105900
H	-32.32152800	1.03348200	0.45385600
C	-28.16228900	1.17892300	-3.17264000
H	-27.77806300	0.35037100	-3.78723300
H	-29.23165900	1.31279700	-3.37387400
H	-27.63111500	2.10020000	-3.44912600
C	-26.73307800	2.13712100	2.01419100
H	-25.72285400	1.98393200	2.42496100
H	-27.09454700	3.13812500	2.27837300
H	-27.42027100	1.40657200	2.45686200
H	-26.13302500	2.69324900	0.14282000
H	-28.53713100	0.09980700	-1.48675000
O	-32.25768900	5.82797800	-1.32081600
H	-32.11669000	6.77621800	-1.19719600

TS2-*S*-III

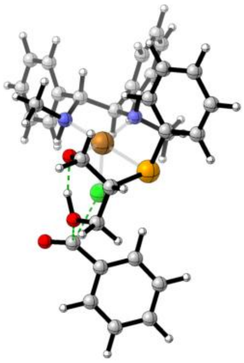

1 2

N	-26.44959700	1.65495100	1.22459700
N	-27.38093600	1.36072100	-1.37343100
C	-26.62885000	0.26871700	0.70800600
C	-26.51658400	0.28886600	-0.82828200
C	-25.66606200	-0.72902600	1.31448500
C	-26.82454500	-1.07147900	-1.41755900
C	-26.14977500	-1.89220800	1.92384800
C	-25.26627400	-2.83813100	2.44944900
C	-23.88759100	-2.62607400	2.37472100
C	-23.39452700	-1.46566200	1.77008400
C	-24.28021300	-0.52606200	1.23985100
C	-28.11918500	-1.60271400	-1.31014200
C	-28.40767900	-2.86607000	-1.82837400
C	-27.40388000	-3.61321500	-2.45304700
C	-26.11279700	-3.09096600	-2.55973100
C	-25.82617500	-1.82399800	-2.04485100
Cu	-27.61565500	2.85963200	0.02255400
H	-27.65694300	-0.01269600	0.97450600
H	-25.48154200	0.56222400	-1.09038600
H	-27.22774100	-2.06233800	1.97711100
H	-25.65672100	-3.74351700	2.91902000
H	-23.19601200	-3.36422600	2.78648100
H	-22.31745900	-1.29574400	1.70719500
H	-23.87439700	0.36973800	0.75851800
H	-28.91166800	-1.03425100	-0.81314200
H	-29.41842900	-3.27061700	-1.74135300
H	-27.62946200	-4.60266900	-2.85664300
H	-25.32524100	-3.67073800	-3.04593100
H	-24.81350300	-1.42040000	-2.12288800

O	-30.53316700	4.52725000	-0.52070200
C	-30.16417500	5.75613200	0.08963700
C	-28.64535900	5.93351200	0.12544500
Se	-27.86201400	4.80777800	1.63322600
C	-26.03880200	5.50162800	1.62375000
C	-25.08898400	5.01659300	0.71507900
C	-23.78231900	5.51000100	0.75129300
C	-23.42471500	6.47865900	1.69365600
C	-24.37442900	6.95653300	2.60057600
C	-25.68476500	6.46974800	2.57005700
C	-28.00339100	5.44990100	-1.17466700
H	-29.67605900	4.13741200	-0.98524300
H	-30.58048100	6.58631800	-0.50995100
H	-30.61191200	5.82370700	1.09461900
H	-28.39206400	6.97478400	0.37233500
H	-25.36590100	4.26471300	-0.02826600
H	-23.04301000	5.13818400	0.03832800
H	-22.40251900	6.86171900	1.72103700
H	-24.09694400	7.71225400	3.33850000
H	-26.42495700	6.84140000	3.28183500
H	-26.92452600	5.68661600	-1.18931300
H	-28.45784800	6.01734700	-2.01166800
C	-27.05458600	1.73830200	-2.75630600
H	-27.11216200	0.86810900	-3.42970400
H	-27.74802100	2.52237700	-3.07946500
H	-26.03327000	2.14477400	-2.78972100
C	-26.70508100	1.75190500	2.67538500
H	-26.14805100	0.98073100	3.22836400
H	-26.39152400	2.73967900	3.03667700
H	-27.78119300	1.62423300	2.85084500
H	-25.47987800	1.93422200	1.05532900
H	-28.35440200	1.04296400	-1.33595400
O	-28.25867700	4.07893800	-1.34245800
C	-32.13642400	4.37224600	3.60718600
C	-33.49649300	4.70169000	3.57105600
C	-34.24281000	4.48814700	2.40849300
C	-33.63401100	3.94946700	1.27593600

C	-32.26393500	3.62546800	1.31723800
C	-31.51398800	3.83485100	2.48327000
C	-31.72117200	3.11415800	0.06531000
Cl	-29.76778900	1.74268900	0.79742300
O	-32.07426300	2.59950300	-0.90596300
H	-31.55975500	4.52630100	4.52134900
H	-33.97891400	5.11990900	4.45719500
H	-35.30543100	4.73731200	2.38241200
H	-34.20690000	3.77877600	0.36303400
H	-30.46803300	3.53647800	2.50171000

TS2-*S*-IV

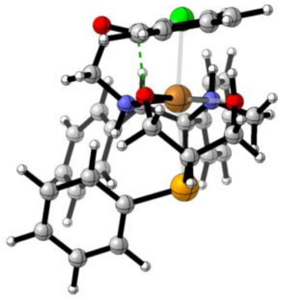

1 2

N	0.73426300	0.11324300	1.21356000
N	0.76883700	-1.86233200	-0.71066600
C	2.00413100	-0.62180800	1.01145400
C	2.05477900	-1.17126300	-0.42694000
C	3.22136100	0.22325200	1.32484800
C	3.27521000	-2.03875300	-0.64265100
C	4.21710900	-0.24798200	2.18715900
C	5.35320300	0.52313700	2.44602800
C	5.50092700	1.77620200	1.84626100
C	4.50946100	2.25505500	0.98437900
C	3.37832100	1.48108300	0.72213300
C	3.39758600	-3.27489700	0.00875600
C	4.54297600	-4.05462400	-0.16017800
C	5.58334800	-3.60077900	-0.97701500
C	5.47057700	-2.36875900	-1.62553600
C	4.31998500	-1.59400600	-1.45999400
Cu	-0.71392900	-0.64635600	-0.06889900

H	1.97968400	-1.48357100	1.69626200
H	2.09863100	-0.31854600	-1.12140600
H	4.10735500	-1.23017600	2.65313500
H	6.12412800	0.14395900	3.12023500
H	6.38745200	2.38032600	2.05093000
H	4.61503200	3.23438400	0.51271700
H	2.61956400	1.87482000	0.04024900
H	2.59858600	-3.64225300	0.66046900
H	4.62611100	-5.01685700	0.34966300
H	6.48121900	-4.20869000	-1.10706300
H	6.28074000	-2.00889000	-2.26331600
H	4.23698100	-0.62682200	-1.96197100
O	-2.16538400	0.49235700	0.55240200
C	-2.83796400	1.33647300	-0.34257800
C	-2.41582700	1.16295900	-1.79786800
Se	-0.41032200	1.34392800	-2.09000800
C	0.16215700	2.86875000	-1.01342100
C	1.06500300	3.74431800	-1.63188800
C	1.66121500	4.76732700	-0.88813100
C	1.34712400	4.93203000	0.46335500
C	0.43032000	4.07012400	1.07061100
C	-0.16071500	3.03323400	0.34192400
C	-2.76962600	-0.20960300	-2.36372600
H	-3.92804600	1.14196500	-0.31259200
H	-2.71154100	2.39455500	-0.05162900
H	-2.89429300	1.93726100	-2.41549300
H	1.30382800	3.62968800	-2.69144500
H	2.36731700	5.44370900	-1.37495800
H	1.81162000	5.73362800	1.04108100
H	0.17518600	4.19457400	2.12548500
H	-0.85212300	2.35289800	0.83797400
H	-2.44831300	-0.30051400	-3.41290900
H	-3.86441300	-0.33298100	-2.33437500
C	-5.99702500	-1.87471800	-0.27237300
C	-6.96026400	-0.89360700	-0.01332600
C	-6.68033600	0.13835900	0.88664700
C	-5.43583600	0.20365200	1.51407200

C	-4.45954100	-0.77043800	1.23468400
C	-4.74945800	-1.81865800	0.35012700
C	-3.17023400	-0.56219400	1.91545700
Cl	-1.84668900	-2.36113700	1.39642200
O	-2.85130100	-0.12483800	2.94600900
H	-6.22572600	-2.70241200	-0.94696800
H	-7.93608700	-0.94365200	-0.50099300
H	-7.43485200	0.89653100	1.10604000
H	-5.21115800	1.00701400	2.21760200
H	-4.01952300	-2.61643100	0.21144400
C	0.66128900	-2.35410100	-2.09629700
H	1.48742600	-3.03877400	-2.33885200
H	-0.29950700	-2.86461600	-2.22483800
H	0.69610000	-1.49756900	-2.78270200
C	0.35241900	0.22457800	2.63070600
H	1.17009200	0.66157000	3.22637100
H	-0.54497600	0.84667700	2.71559900
H	0.10433100	-0.77230700	3.01688100
H	0.85457900	1.06081000	0.84843600
H	0.67790200	-2.66148900	-0.07689000
O	-2.15019600	-1.27349800	-1.62732400
H	-2.80138200	-1.69232500	-1.04201200

Int3(*R*)

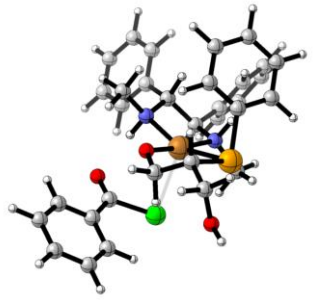

1 2

N	1.14437500	-0.71194100	-1.12157600
N	0.62856000	1.10861200	0.89874400
C	2.14699500	0.35957000	-0.87241100
C	2.04126700	0.80780800	0.59865500
C	3.55581500	-0.06581200	-1.22299900

C	2.98525000	1.95588500	0.88866700
C	4.33858200	0.70259200	-2.09067800
C	5.65358200	0.32785700	-2.37840800
C	6.19542500	-0.82346000	-1.80232500
C	5.41895300	-1.59823700	-0.93473300
C	4.10776700	-1.21881200	-0.64380700
C	2.72098000	3.23658600	0.38158300
C	3.61541600	4.28441800	0.60768500
C	4.78611900	4.06064700	1.33874700
C	5.05550800	2.78711000	1.84584600
C	4.15696700	1.74126300	1.62249800
Cu	-0.54593400	-0.29175800	-0.06892300
H	1.85497700	1.20947600	-1.50871200
H	2.31954400	-0.04942400	1.23307500
H	3.91960500	1.60835900	-2.53575200
H	6.25609300	0.93765900	-3.05496200
H	7.22266400	-1.11776300	-2.02779700
H	5.83914100	-2.49780400	-0.47941800
H	3.52057200	-1.82920000	0.04939100
H	1.81085000	3.43064000	-0.19387300
H	3.39834100	5.27874300	0.21138200
H	5.48651000	4.88002000	1.51433700
H	5.96795300	2.60678200	2.41831100
H	4.37355700	0.74373000	2.01257100
O	-4.55534800	-2.51040700	-1.00017200
C	-4.33228300	-2.83411200	0.35642300
C	-3.08854200	-2.09676800	0.81360400
Se	-1.57582100	-2.60399800	-0.44602900
C	-0.12659000	-3.05511500	0.79661400
C	0.02378700	-2.42138400	2.03678700
C	1.16914200	-2.66940800	2.79955000
C	2.16406700	-3.53224900	2.32771800
C	2.00419800	-4.16509000	1.09168100
C	0.85641300	-3.93426400	0.32450800
C	-3.21062700	-0.56904200	0.77892300
H	-5.30647300	-3.01775600	-1.33280900
H	-5.17347500	-2.50452300	1.00030600

H	-4.20417400	-3.92181400	0.50778100
H	-2.76894400	-2.45251300	1.80356000
H	-0.72793900	-1.71290200	2.38931300
H	1.28466300	-2.17879300	3.76895400
H	3.05749000	-3.71858600	2.92715300
H	2.76850400	-4.85265500	0.72267500
H	0.72928500	-4.44208800	-0.63482700
H	-3.94898600	-0.29239500	1.56046500
H	-3.67014200	-0.29035300	-0.18923800
C	-5.83985300	2.14328900	-0.84607400
C	-6.21011600	2.85352800	0.30029000
C	-5.23526500	3.45954600	1.10309400
C	-3.89154200	3.35686900	0.76014400
C	-3.51492700	2.63382700	-0.38994100
C	-4.49533600	2.02739500	-1.19297400
C	-2.07926200	2.56318700	-0.66701200
Cl	-1.61384100	1.41784300	-2.06488200
O	-1.18838000	3.13751100	-0.12517500
H	-6.60237300	1.67710700	-1.47281300
H	-7.26535100	2.93907900	0.56960000
H	-5.52803100	4.01483100	1.99634600
H	-3.11846600	3.82222100	1.37265300
H	-4.20401400	1.47296200	-2.08395900
C	0.34455400	1.29251900	2.32975800
H	0.89173400	2.15783200	2.73576800
H	-0.73631300	1.42362400	2.45371800
H	0.65324300	0.39163300	2.87733900
C	0.98319400	-1.01828400	-2.55536800
H	1.94782300	-1.27717600	-3.01825700
H	0.28534500	-1.85672100	-2.67547700
H	0.56600900	-0.14157100	-3.06763500
H	1.48108500	-1.56140300	-0.65801700
H	0.34579600	1.96786200	0.41643900
O	-2.01742200	0.08772000	1.02318500

Int3(*S*)

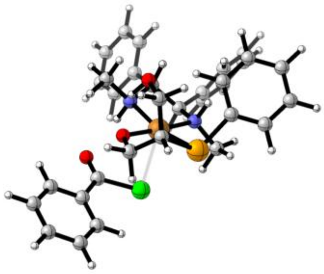

1 2

N	-1.16425500	0.60955900	-0.86381300
N	-0.39261500	-1.34000900	0.95152600
C	-2.04751700	-0.57463000	-0.69020500
C	-1.84038100	-1.15073800	0.72411600
C	-3.50540900	-0.26906000	-0.95730400
C	-2.65622100	-2.41120800	0.92256400
C	-4.25680200	-1.08975000	-1.80531700
C	-5.61637900	-0.84237000	-2.00921000
C	-6.23594700	0.23360800	-1.36898200
C	-5.49175100	1.06094100	-0.52235200
C	-4.13474500	0.80769800	-0.31513800
C	-2.28819200	-3.60180300	0.27883000
C	-3.06985700	-4.75025400	0.41695300
C	-4.23073100	-4.71771900	1.19562300
C	-4.60289700	-3.53473600	1.83877300
C	-3.81684200	-2.38809600	1.70345600
Cu	0.61488800	0.23083400	0.06291000
H	-1.69798100	-1.32670000	-1.41452100
H	-2.17695500	-0.39340600	1.45104000
H	-3.77667600	-1.93789600	-2.29921900
H	-6.19296000	-1.49232800	-2.67076900
H	-7.29837200	0.42900000	-1.52930900
H	-5.97114300	1.90362300	-0.01944600
H	-3.57241900	1.46197700	0.35897600
H	-1.38452600	-3.64691300	-0.33655300
H	-2.77228400	-5.67327600	-0.08531300
H	-4.84302800	-5.61570800	1.30249600
H	-5.50774200	-3.50394300	2.44949800
H	-4.11467200	-1.46162400	2.20048500
C	3.29206900	0.70960400	0.89773700

C	2.84000100	2.16074700	1.12232400
Se	1.61302200	2.45977100	-0.48891200
C	0.13666100	3.61039000	0.07170100
C	-0.70561400	3.28539500	1.14472700
C	-1.82323500	4.08649200	1.39894000
C	-2.10265100	5.19308400	0.59025400
C	-1.25263300	5.51250100	-0.47135900
C	-0.12676200	4.72522700	-0.73327000
C	2.17302100	2.38428700	2.47376800
H	4.04724800	0.47571900	1.67556100
H	3.82457200	0.63006900	-0.07316100
H	3.66628600	2.87416600	0.98865900
H	-0.46981900	2.43873800	1.79403600
H	-2.47384100	3.84541200	2.24306000
H	-2.97880900	5.81233300	0.79360300
H	-1.45989200	6.38152200	-1.09949500
H	0.53832600	4.97932900	-1.56156800
H	1.73574600	3.39391300	2.53554700
H	2.98976600	2.34856600	3.22421000
C	5.98301500	-1.47719400	-1.40628300
C	6.57022100	-2.15733800	-0.33449300
C	5.78040500	-2.91201900	0.54212200
C	4.40528800	-2.98797600	0.34822800
C	3.80985400	-2.29522000	-0.72500400
C	4.60466600	-1.53999300	-1.60330400
C	2.35528300	-2.41429400	-0.84178900
Cl	1.58689200	-1.30092100	-2.13574500
O	1.61111900	-3.11664400	-0.23634400
H	6.60260800	-0.89704600	-2.09278900
H	7.65056600	-2.10269700	-0.18245300
H	6.24220400	-3.44380100	1.37635100
H	3.77474400	-3.57029100	1.02096600
H	4.14410700	-1.01254500	-2.43771800
C	-0.04994400	-1.62502100	2.35538100
H	-0.58432000	-2.51660400	2.71887300
H	1.03175900	-1.78671600	2.42359800
H	-0.32262800	-0.76462200	2.98215000

C	-1.10983100	1.06652000	-2.26583100
H	-2.12166400	1.23022500	-2.66624100
H	-0.54809200	2.00606500	-2.32735500
H	-0.60173600	0.30846100	-2.87558200
H	-1.54681800	1.37858500	-0.30471500
H	-0.05893800	-2.13182000	0.39220700
O	1.17017700	1.44315300	2.77633500
O	2.22717900	-0.18752800	0.99447900
H	1.48915800	0.58835300	2.40744500

(13) Docking studies

The three-dimensional (3D) structures of sterol 14-alpha demethylase (PDBID: CYP51) were retrieved from the RCSB Protein Data Bank available at https://www. rcsb.org and used as protein targets. The choice of 5ZT1 was based on previous studies where it has been reported as a popular PDB structure for computer-aided drug discovery, featuring a co-crystallized structure as well as its prominent active site pocket.

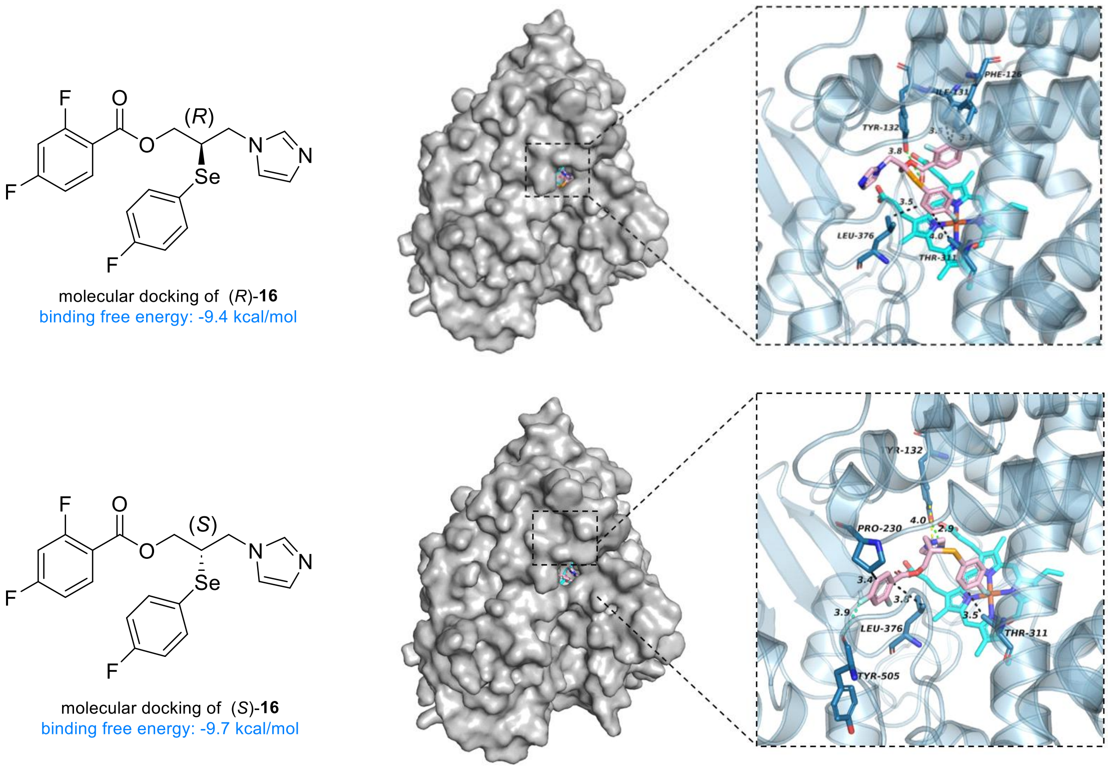

Figure S12. Molecular docking of **16** (substrate highlighted in pink) onto CYP51 to identify key amino acid residues represented as sticks and marked in blue.

All docking studies were conducted using AutoDock 4.0 software[22]. An MMFF94 force field-optimized structure of the small molecule served as the ligand model, and the receptor structure was prepared following the default receptor preparation workflow using Autodocktools. Molecular docking employed the Genetic Algorithm as the search method, employing 50 independent genetic algorithm runs and default docking parameters. The grid box was defined with the following settings: center coordinates (center_x = 70.486, center_y = 65.238, center_z = 4.453), number of grid points (x = 54, y = 62, z = 60), and a grid spacing of 0.375 Å, representing the active ligand-binding region. The resulting docked ligand conformations were evaluated based on their binding energy scores, with lower scores indicating higher rankings. The conformation exhibiting the lowest binding energy among all docking results was selected as the final output. All molecular graphics were generated using PyMOL[23] software and and Protein-Ligand Interaction Profiler.

4. Single Crystal Structure X-ray Analysis of 3al

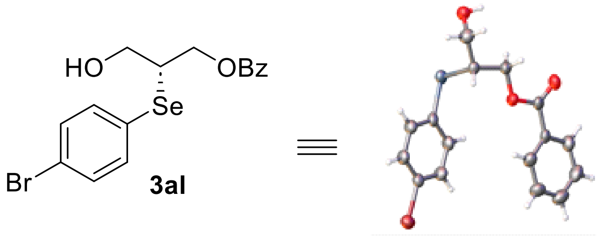

CCDC: 2504122

Table S9. Crystal data and structure refinement for 20250616-Dai Lei-CJX-2-73.

Identification code	20250616-Dai Lei-CJX-2-73
Empirical formula	$C_{16}H_{15}BrO_3Se$
Formula weight	414.15
Temperature/K	293(2)
Crystal system	monoclinic
Space group	$P2_1$
a/Å	10.1302(2)
b/Å	5.11160(10)
c/Å	15.1129(2)
α/°	90
β/°	92.438(2)
γ/°	90
Volume/Å^3	781.86(2)
Z	2
ρ_{calc}g/cm^3	1.759
μ/mm^{-1}	6.308
F(000)	408.0
Crystal size/mm^3	0.52 × 0.28 × 0.24
Radiation	Cu Kα (λ = 1.54184)
2Θ range for data collection/°	5.854 to 141.532
Index ranges	-12 ≤ h ≤ 12, -6 ≤ k ≤ 5, -18 ≤ l ≤ 18
Reflections collected	11034
Independent reflections	2772 [R_{int} = 0.0364, R_{sigma} = 0.0238]
Data/restraints/parameters	2772/1/191
Goodness-of-fit on F^2	1.095

Final R indexes [I>=2σ (I)]	R_1 = 0.0470, wR_2 = 0.1133
Final R indexes [all data]	R_1 = 0.0470, wR_2 = 0.1134
Largest diff. peak/hole / e $Å^{-3}$	0.78/-1.53
Flack parameter	-0.02(4)

Table S10. Fractional Atomic Coordinates (×10^4) and Equivalent Isotropic Displacement Parameters ($Å^2$×10^3) for 20250616-Dai Lei-CJX-2-73. U_{eq} is defined as 1/3 of the trace of the 91rthogonalized U_{IJ} tensor.

**Atom**	***x***	***y***	***z***	**U(eq)**
Br1	7480.8(6)	-544.5(14)	5562.5(3)	43.7(2)
Se1	7645.8(5)	8628.2(15)	2509.3(3)	38.8(2)
O1	8454(4)	10519(11)	674(3)	48.1(10)
O2	5408(4)	5074(10)	1463(2)	44.1(10)
O3	3721(4)	6357(12)	563(3)	51.9(11)
C6	7578(5)	5846(14)	3358(3)	35.9(12)
C7	7645(5)	6567(13)	1398(3)	34.8(11)
C1	6373(5)	4976(15)	3668(4)	42.0(15)
C3	7509(5)	2052(14)	4663(3)	36.1(12)
C11	3325(5)	3351(13)	1732(3)	34.9(11)
C2	6337(5)	3076(15)	4315(4)	41.3(14)
C5	8744(5)	4768(15)	3717(3)	40.3(13)
C10	4144(5)	5085(12)	1192(3)	37.3(12)
C12	1953(5)	3556(18)	1631(4)	47.3(14)
C16	3889(6)	1511(15)	2311(4)	44.2(13)
C15	3085(7)	-92(16)	2788(5)	53.8(16)
C8	8660(6)	7817(16)	807(5)	45.0(14)
C4	8719(5)	2848(16)	4363(4)	40.5(14)
C9	6289(6)	6578(16)	924(4)	42.0(14)
C14	1716(7)	124(18)	2685(5)	56.7(18)
C13	1152(6)	1952(19)	2102(5)	57(2)

Table S11. Anisotropic Displacement Parameters (Å^2×10^3) for 20250616-Dai Lei-CJX-2-73. The Anisotropic displacement factor exponent takes the form: $-2\pi^2[h^2a^{*2}U_{11}+2hka^*b^*U_{12}+\ldots]$.

Atom	U_{11}	U_{22}	U_{33}	U_{23}	U_{13}	U_{12}
Br1	44.6(3)	47.9(4)	38.5(3)	2.8(2)	0.6(2)	-1.0(2)
Se1	45.0(3)	35.9(4)	35.0(3)	-3.9(2)	-5.4(2)	-3.2(3)
O1	45(2)	50(3)	48(2)	11(2)	-9.7(16)	-10(2)
O2	36.7(17)	52(3)	42.6(17)	12(2)	-9.8(14)	-13(2)
O3	47(2)	62(3)	46(2)	9(2)	-15.9(16)	-8(2)
C6	31(2)	49(3)	28(2)	-7(2)	-3.9(16)	-2(2)
C7	33(2)	35(3)	36(2)	-2(2)	-3.0(18)	-2(2)
C1	27(2)	55(4)	43(3)	3(3)	-4.6(18)	3(3)
C3	33(2)	45(3)	29(2)	-5(2)	-0.9(17)	-2(2)
C11	32(2)	38(3)	34(2)	-5(2)	-7.8(17)	-4(2)
C2	26.4(19)	53(4)	45(3)	3(3)	3.3(18)	0(3)
C5	28(2)	53(4)	40(2)	1(3)	1.5(18)	0(3)
C10	39(2)	36(3)	36(2)	-3(2)	-8.6(19)	-6(2)
C12	32(2)	63(4)	46(3)	-1(3)	-4(2)	0(3)
C16	40(3)	40(3)	52(3)	1(3)	-3(2)	-3(3)
C15	54(3)	47(4)	60(4)	8(3)	3(3)	-8(3)
C8	36(2)	51(4)	48(3)	-1(3)	6(2)	0(3)
C4	25(2)	51(4)	45(3)	3(3)	-4.7(18)	1(2)
C9	41(3)	51(4)	34(2)	3(3)	-6(2)	-13(3)
C14	54(3)	55(5)	62(4)	-1(4)	12(3)	-15(4)
C13	34(3)	74(6)	62(4)	-11(4)	4(3)	-11(3)

Table S12. Bond Lengths for 20250616-Dai Lei-CJX-2-73.

Atom	Atom	Length/Å	Atom	Atom	Length/Å
Br1	C3	1.901(6)	C1	C2	1.379(9)
Se1	C6	1.919(6)	C3	C2	1.381(8)
Se1	C7	1.982(5)	C3	C4	1.386(7)
O1	C8	1.410(10)	C11	C10	1.482(8)
O2	C10	1.328(6)	C11	C12	1.396(7)
O2	C9	1.453(7)	C11	C16	1.390(9)
O3	C10	1.215(7)	C5	C4	1.385(10)

C6	C1	1.399(7)	C12	C13	1.373(11)
C6	C5	1.392(8)	C16	C15	1.381(10)
C7	C8	1.530(8)	C15	C14	1.394(10)
C7	C9	1.523(7)	C14	C13	1.390(12)

Table S13. Bond Angles for 20250616-Dai Lei-CJX-2-73.

Atom	Atom	Atom	Angle/˚	Atom	Atom	Atom	Angle/˚
C6	Se1	C7	100.0(2)	C16	C11	C12	120.0(6)
C10	O2	C9	115.6(4)	C1	C2	C3	119.2(5)
C1	C6	Se1	121.1(5)	C4	C5	C6	121.0(5)
C5	C6	Se1	120.0(4)	O2	C10	C11	112.5(4)
C5	C6	C1	118.8(6)	O3	C10	O2	123.1(5)
C8	C7	Se1	107.3(4)	O3	C10	C11	124.3(5)
C9	C7	Se1	111.4(4)	C13	C12	C11	120.4(7)
C9	C7	C8	109.8(5)	C15	C16	C11	119.7(6)
C2	C1	C6	120.7(5)	C16	C15	C14	120.1(7)
C2	C3	Br1	119.9(4)	O1	C8	C7	113.1(6)
C2	C3	C4	121.5(6)	C5	C4	C3	118.7(5)
C4	C3	Br1	118.6(5)	O2	C9	C7	107.3(5)
C12	C11	C10	118.2(6)	C13	C14	C15	120.3(6)
C16	C11	C10	121.8(5)	C12	C13	C14	119.6(6)

Table S14. Torsion Angles for 20250616-Dai Lei-CJX-2-73.

A	B	C	D	Angle/˚	A	B	C	D	Angle/˚
Br1	C3	C2	C1	179.4(5)	C10	C11	C16	C15	-178.9(6)
Br1	C3	C4	C5	-179.0(5)	C12	C11	C10	O2	167.9(6)
Se1	C6	C1	C2	-176.4(5)	C12	C11	C10	O3	-13.1(9)
Se1	C6	C5	C4	176.8(5)	C12	C11	C16	C15	-0.5(10)
Se1	C7	C8	O1	-53.5(6)	C16	C11	C10	O2	-13.7(8)
Se1	C7	C9	O2	-68.6(6)	C16	C11	C10	O3	165.3(6)
C6	C1	C2	C3	0.7(10)	C16	C11	C12	C13	0.2(10)
C6	C5	C4	C3	-1.5(10)	C16	C15	C14	C13	0.0(12)
C1	C6	C5	C4	0.7(10)	C15	C14	C13	C12	-0.4(12)

C11	C12	C13	C14	0.3(11)	C8	C7	C9	O2	172.7(5)
C11	C16	C15	C14	0.4(11)	C4	C3	C2	C1	-1.5(10)
C2	C3	C4	C5	1.9(11)	C9	O2	C10	O3	-3.5(9)
C5	C6	C1	C2	-0.3(10)	C9	O2	C10	C11	175.6(5)
C10	O2	C9	C7	173.8(5)	C9	C7	C8	O1	67.6(8)
C10	C11	C12	C13	178.6(6)					

Table S15. Hydrogen Atom Coordinates (Å×10^4) and Isotropic Displacement Parameters (Å^2×10^3) for 20250616-Dai Lei-CJX-2-73.

**Atom**	***x***	***y***	***z***	**U(eq)**
H1	7850.96	10738.26	302.26	72
H7	7907.48	4760.72	1534.25	42
H1A	5587.28	5687.45	3436.24	50
H2	5532.48	2490.09	4514.01	50
H5	9550.78	5346.25	3519.85	48
H12	1578.41	4785.47	1242.16	57
H16	4802.62	1360.81	2375.52	53
H15	3457.41	-1317.87	3179.19	65
H8A	9537.67	7549.85	1073.8	54
H8B	8622.84	6942.84	236.74	54
H4	9500.36	2105.83	4590.44	49
H9A	5967.92	8357.27	854.57	50
H9B	6336.95	5795.42	341.88	50
H14	1176.51	-957.9	3008.22	68
H13	238.48	2087.97	2031.62	68

5. References

1 Gaussian 16, Revision C.01, M. J. Frisch, G. W. Trucks, H. B. Schlegel, G. E. Scuseria, M. A. Robb, J. R. Cheeseman, G. Scalmani, V. Barone, G. A. Petersson, H. Nakatsuji, X. Li, M. Caricato, A. V. Marenich, J. Bloino, B. G. Janesko, R. Gomperts, B. Mennucci, H. P. Hratchian, J. V. Ortiz, A. F. Izmaylov, J. L. Sonnenberg, D. Williams-Young, F. Ding, F. Lipparini, F. Egidi, J. Goings, B. Peng, A. Petrone, T. Henderson, D. Ranasinghe, V. G. Zakrzewski, J. Gao, N. Rega, G. Zheng, W. Liang, M. Hada, M. Ehara, K. Toyota, R. Fukuda, J. Hasegawa, M. Ishida, T. Nakajima, Y. Honda, O. Kitao, H. Nakai, T. Vreven, K. Throssell, J. A. Montgomery, Jr., J. E. Peralta, F. Ogliaro, M. J. Bearpark, J. J. Heyd, E. N. Brothers, K. N. Kudin, V. N. Staroverov, T. A. Keith, Kobayashi, J. Normand, K. Raghavachari, A. P. Rendell, J. C. Burant, S. S. Iyengar, J. Tomasi, M. Cossi, J. M. Millam, M. Klene, C. Adamo, R. Cammi, J. W. Ochterski, R. L. Martin, K. Morokuma, O. Farkas, J. B. Foresman, and D. J. Fox, Gaussian, Inc., Wallingford CT, 2019.

2 P. J. Stephens, F. J. Devlin, C. F. Chabalowski, M. J. Frisch, *J. Chem. Phys.* **2002**, *98*, 11623-11627.

3 S. Grimme, J. Antony, S. Ehrlich, H. Krieg, *J. Chem. Phys.* **2010**, *132*, 154104.

4 S. Grimme, S. Ehrlich, L. Goerigk, *J. Comput. Chem.* **2011**, *32*, 1456-1465.

5 F. Weigend, R. Ahlrichs, *Phys. Chem. Chem. Phys.* **2005**, *7*, 3297-3305.

6 K. Fukui, *Acc. Chem. Res.* **2002**, *14*, 363-368.

7 H. P. Hratchian, H. B. Schlegel, *J. Chem. Phys.* **2004**, *120*, 9918-9924.

8 A. V. Marenich, C. J. Cramer, D. G. Truhlar, *J. Phys. Chem. B* **2009**, *113*, 4538-4543.

9 A. V. Marenich, C. J. Cramer, D. G. Truhlar, *J. Phys. Chem. B* **2009**, *113*, 6378-6396.

10 F. Weigend, *Phys. Chem. Chem. Phys.* **2006**, *8*, 1057-1065.

11 T. Lu, F. Chen, *J. Comput. Chem.* **2012**, *33*, 580-592.

12 T. Lu, Q. Chen, *J. Comput. Chem.* **2022**, *43*, 539-555.

13 T. Lu, Q. Chen, *J. Phys. Chem. A* **2023**, *127*, 7023-7035.

14 D. H. Ess, K. N. Houk, *J. Am. Chem. Soc.* **2007**, *129*, 10646-10647.

15 F. Liu, Y. Liang, K. N. Houk, *Acc. Chem. Res.* **2017**, *50*, 2297-2308.

16 L. Falivene, Z. Cao, A. Petta, L. Serra, A. Poater, R. Oliva, V. Scarano, L. Cavallo, *Nat. Chem.* **2019**, *11*, 872-879.

17 Legault, C. Y. CYLview20, Université de Sherbrooke, Canada, 2020; http://www.cylview.org.

18 W. Humphrey, A. Dalke, K. J. J. o. m. g. Schulten, *J. Mol. Graph.* **1996**, *14*, 33-38.

19 I. Fernandez, F. M. Bickelhaupt, F. P. Cossio, *Chem. Eur. J.* **2012**, *18*, 12395-12403.

20 T. M. Parker, L. A. Burns, R. M. Parrish, A. G. Ryno, C. D. Sherrill, *J. Chem. Phys.* **2014**, *140*, 094106.

21 K. Szalewicz, *WIREs: Comput. Mol. Sci.* **2011**, *2*, 254-272.

22 G. M. Morris, R. Huey, W. Lindstrom, M. F. Sanner, R. K. Belew, D. S. Goodsell, A. J. Olson, *J. Comput. Chem.* **2009**, *30*, 2785-2791.

23 W. L. DeLano, *CCP4 Newsl Protein Crystallogr* **2002**, *40*, 82-92.

6. NMR Spectra

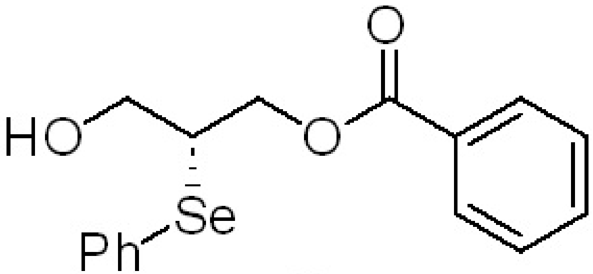

3a

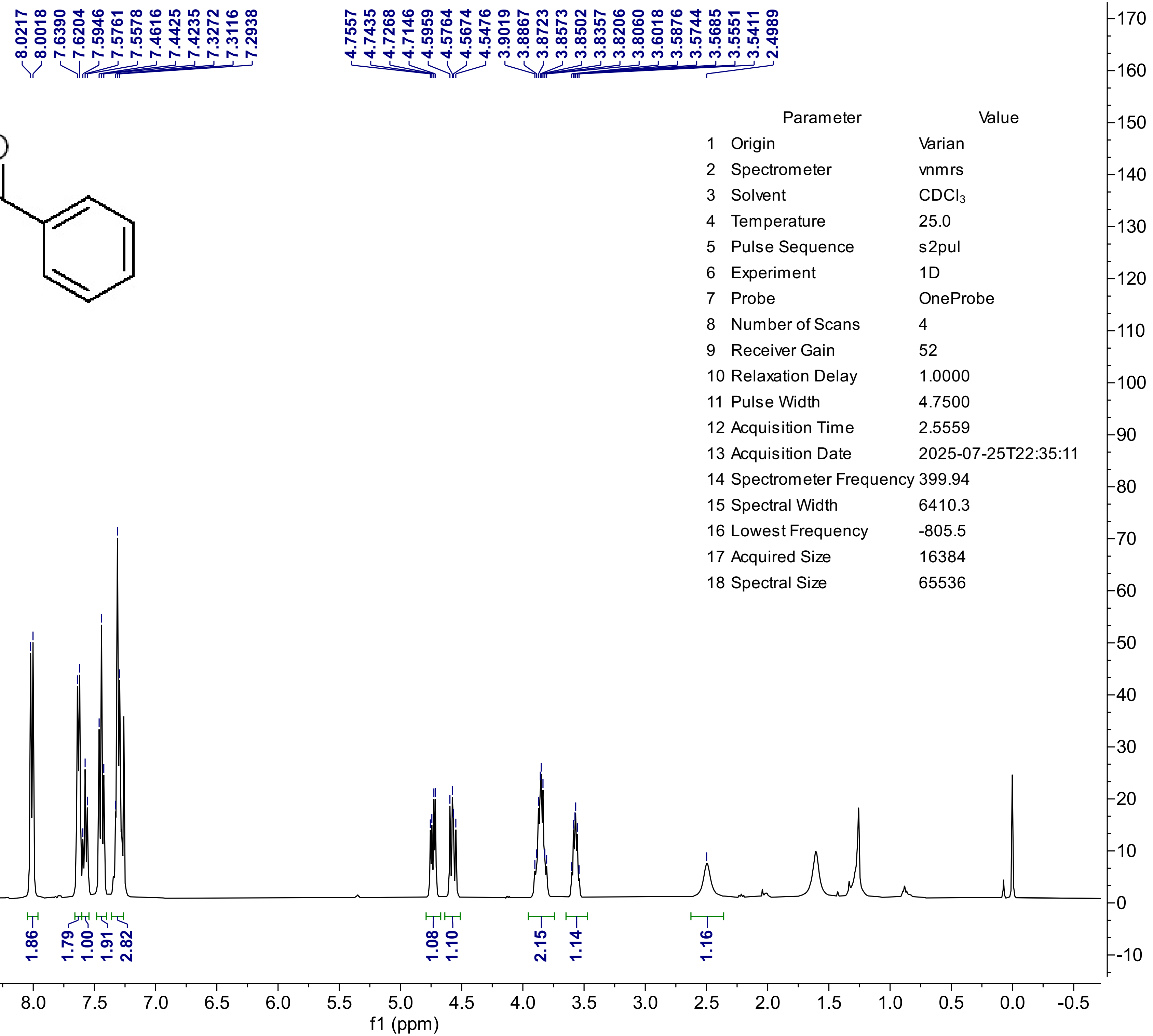

	Parameter	Value
1	Origin	Varian
2	Spectrometer	vnmrs
3	Solvent	$CDCl_3$
4	Temperature	25.0
5	Pulse Sequence	s2pul
6	Experiment	1D
7	Probe	OneProbe
8	Number of Scans	4
9	Receiver Gain	52
10	Relaxation Delay	1.0000
11	Pulse Width	4.7500
12	Acquisition Time	2.5559
13	Acquisition Date	2025-07-25T22:35:11
14	Spectrometer Frequency	399.94
15	Spectral Width	6410.3
16	Lowest Frequency	-805.5
17	Acquired Size	16384
18	Spectral Size	65536

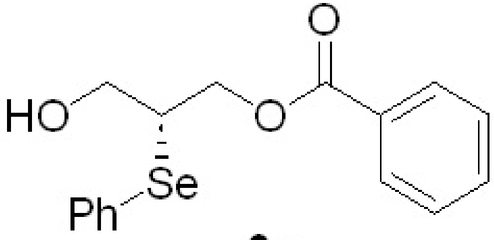

3a

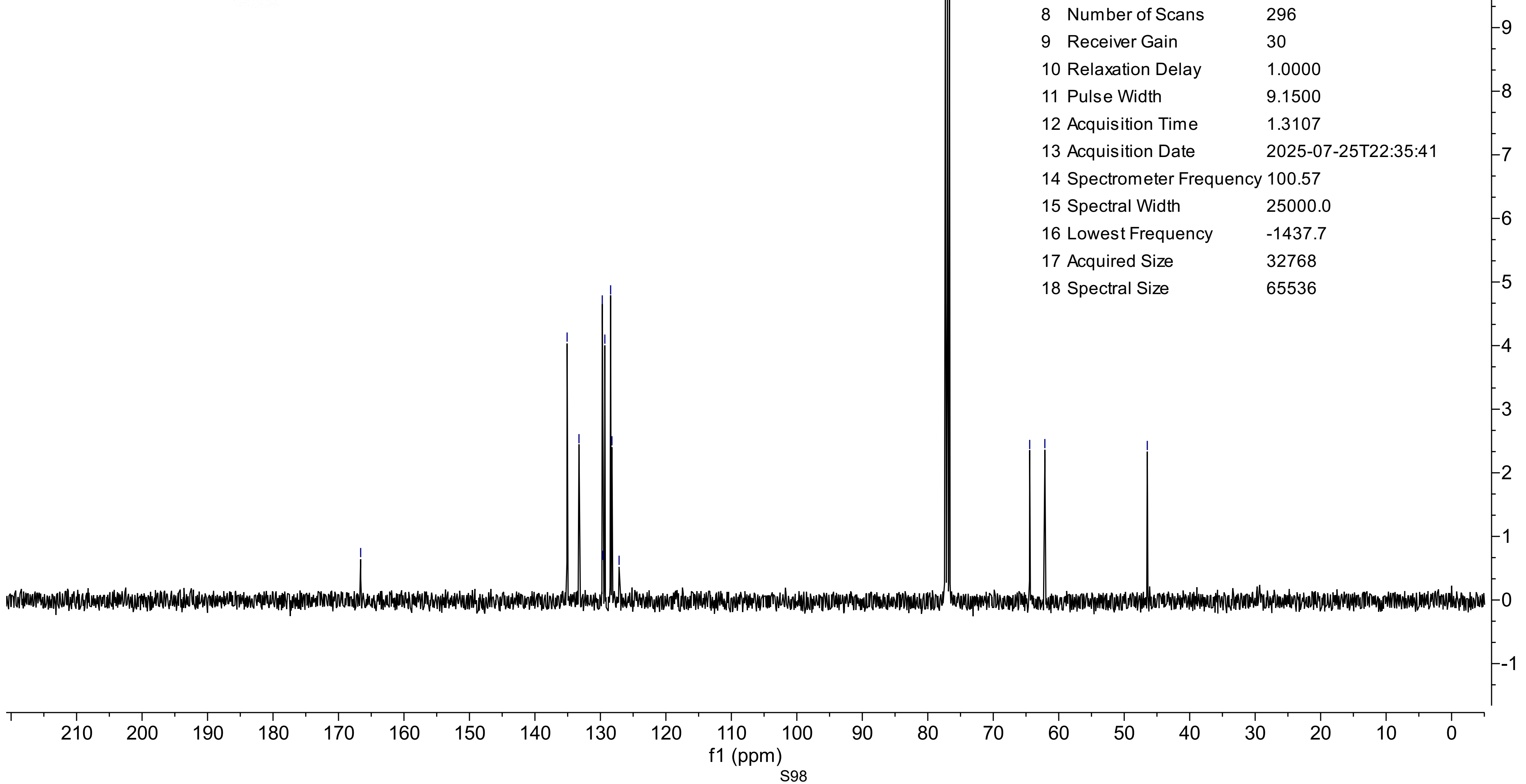

	Parameter	Value
1	Origin	Varian
2	Spectrometer	vnmrs
3	Solvent	$CDCl_3$
4	Temperature	25.0
5	Pulse Sequence	s2pul
6	Experiment	1D
7	Probe	OneProbe
8	Number of Scans	296
9	Receiver Gain	30
10	Relaxation Delay	1.0000
11	Pulse Width	9.1500
12	Acquisition Time	1.3107
13	Acquisition Date	2025-07-25T22:35:41
14	Spectrometer Frequency	100.57
15	Spectral Width	25000.0
16	Lowest Frequency	-1437.7
17	Acquired Size	32768
18	Spectral Size	65536

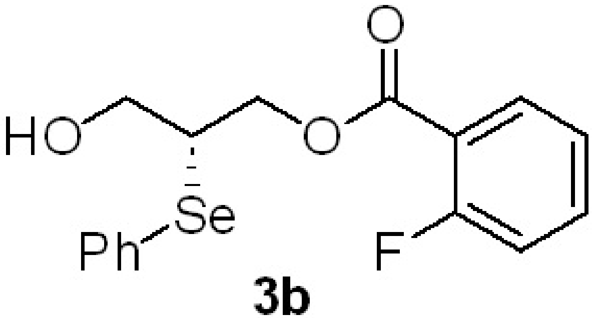

3b

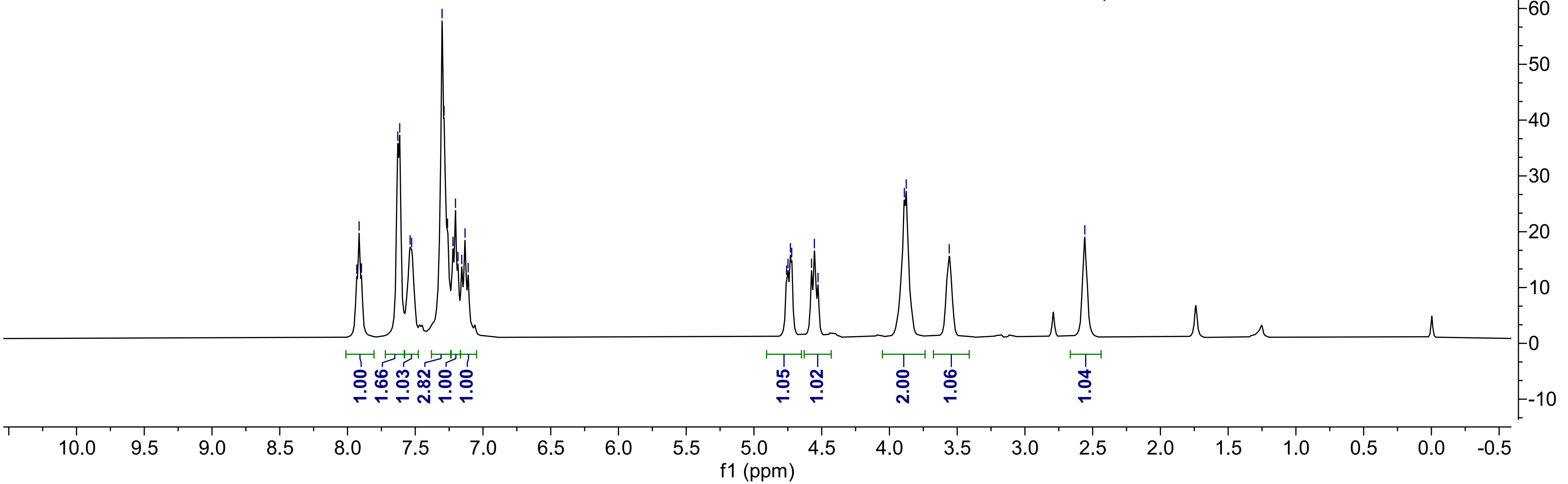

	Parameter	Value
1	Origin	Varian
2	Spectrometer	vnmrs
3	Solvent	$CDCl_3$
4	Temperature	25.0
5	Pulse Sequence	s2pul
6	Experiment	1D
7	Probe	OneProbe
8	Number of Scans	4
9	Receiver Gain	38
10	Relaxation Delay	1.0000
11	Pulse Width	4.7500
12	Acquisition Time	2.5559
13	Acquisition Date	2025-03-17T22:53:40
14	Spectrometer Frequency	399.94
15	Spectral Width	6410.3
16	Lowest Frequency	-805.5
17	Acquired Size	16384
18	Spectral Size	65536

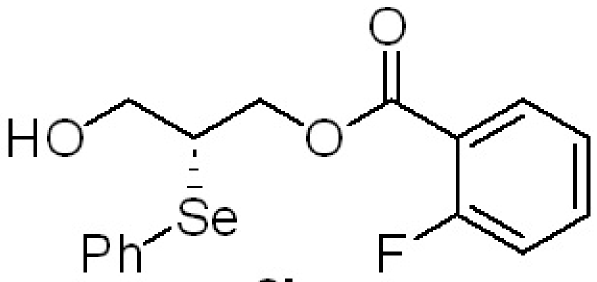

3b

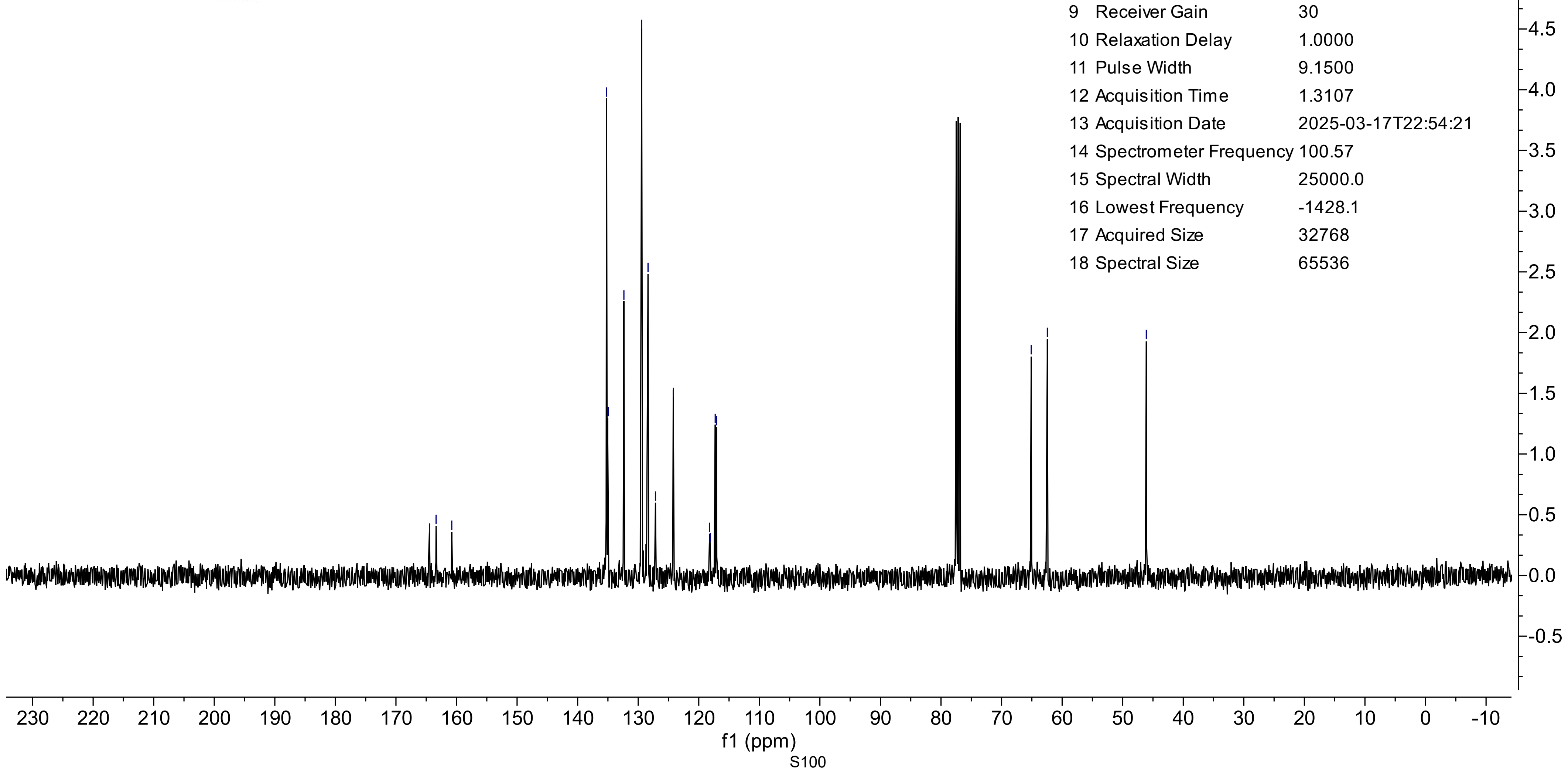

	Parameter	Value
1	Origin	Varian
2	Spectrometer	vnmrs
3	Solvent	$CDCl_3$
4	Temperature	25.0
5	Pulse Sequence	s2pul
6	Experiment	1D
7	Probe	OneProbe
8	Number of Scans	128
9	Receiver Gain	30
10	Relaxation Delay	1.0000
11	Pulse Width	9.1500
12	Acquisition Time	1.3107
13	Acquisition Date	2025-03-17T22:54:21
14	Spectrometer Frequency	100.57
15	Spectral Width	25000.0
16	Lowest Frequency	-1428.1
17	Acquired Size	32768
18	Spectral Size	65536

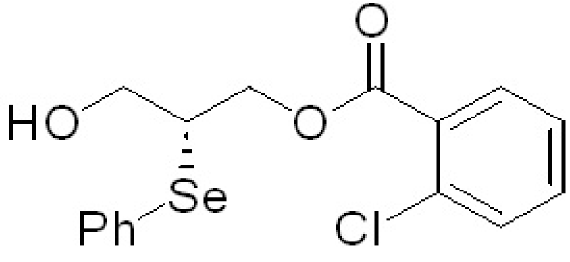

3c

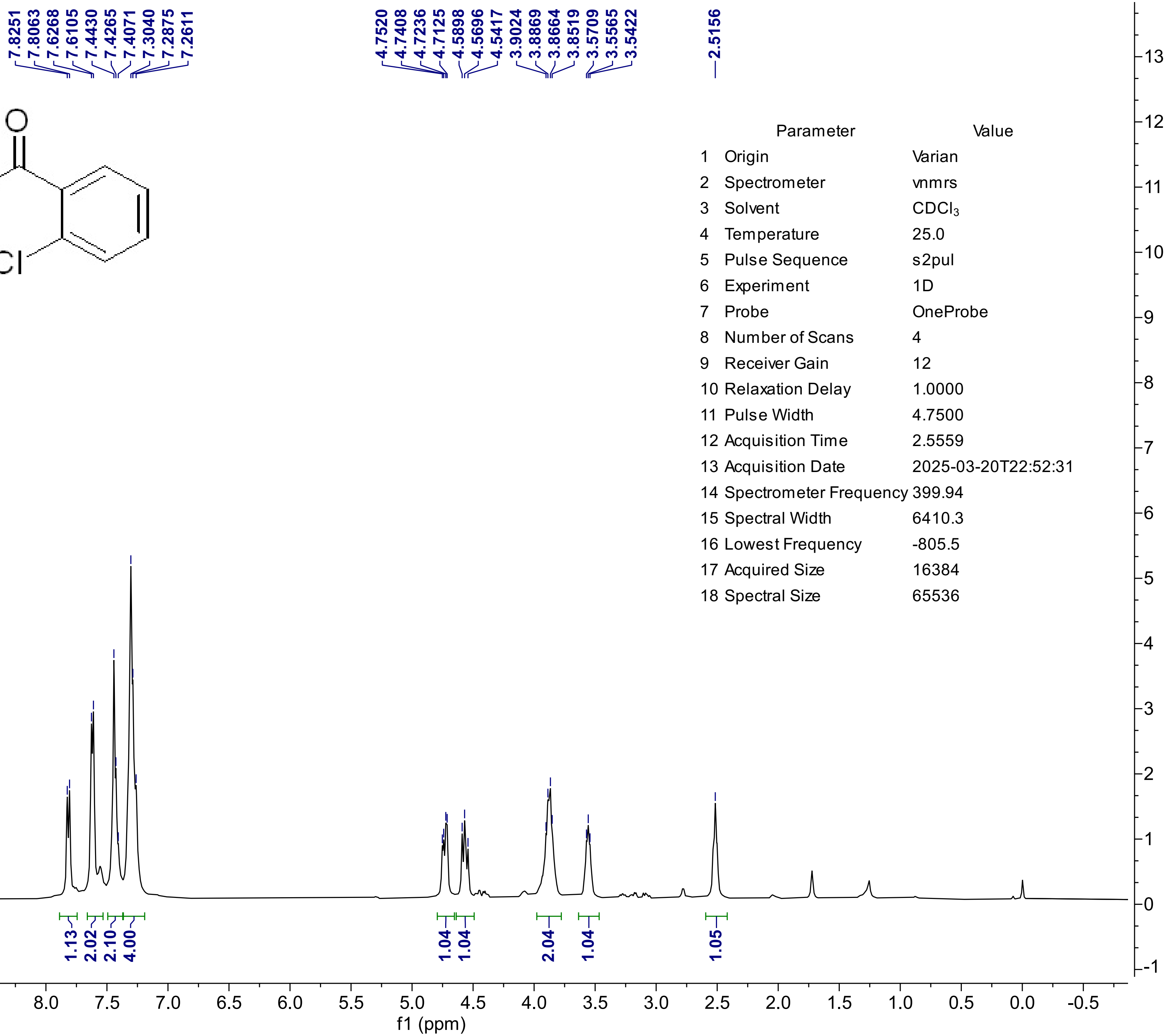

	Parameter	Value
1	Origin	Varian
2	Spectrometer	vnmrs
3	Solvent	$CDCl_3$
4	Temperature	25.0
5	Pulse Sequence	s2pul
6	Experiment	1D
7	Probe	OneProbe
8	Number of Scans	4
9	Receiver Gain	12
10	Relaxation Delay	1.0000
11	Pulse Width	4.7500
12	Acquisition Time	2.5559
13	Acquisition Date	2025-03-20T22:52:31
14	Spectrometer Frequency	399.94
15	Spectral Width	6410.3
16	Lowest Frequency	-805.5
17	Acquired Size	16384
18	Spectral Size	65536

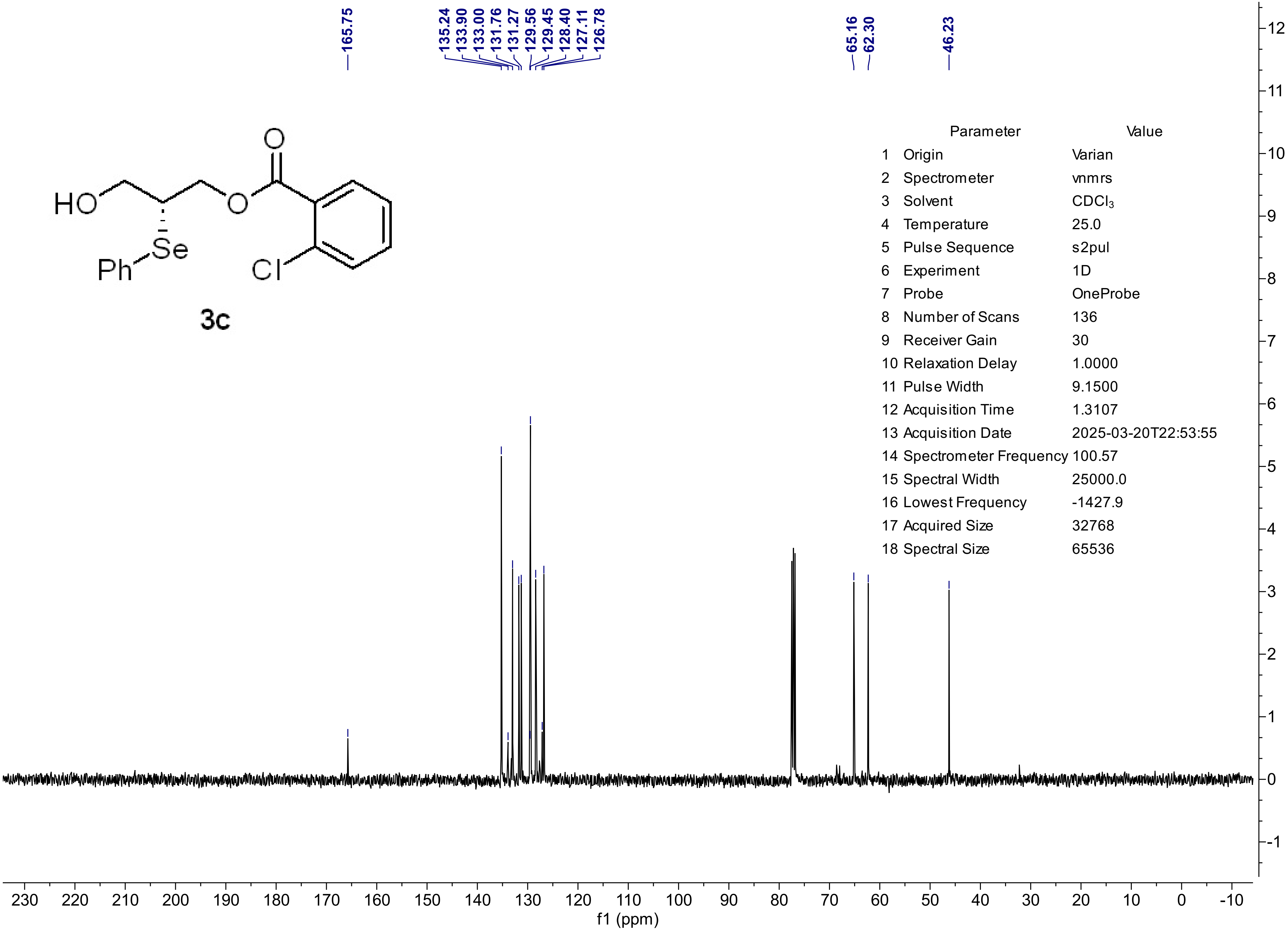

3c

	Parameter	Value
1	Origin	Varian
2	Spectrometer	vnmrs
3	Solvent	$CDCl_3$
4	Temperature	25.0
5	Pulse Sequence	s2pul
6	Experiment	1D
7	Probe	OneProbe
8	Number of Scans	136
9	Receiver Gain	30
10	Relaxation Delay	1.0000
11	Pulse Width	9.1500
12	Acquisition Time	1.3107
13	Acquisition Date	2025-03-20T22:53:55
14	Spectrometer Frequency	100.57
15	Spectral Width	25000.0
16	Lowest Frequency	-1427.9
17	Acquired Size	32768
18	Spectral Size	65536

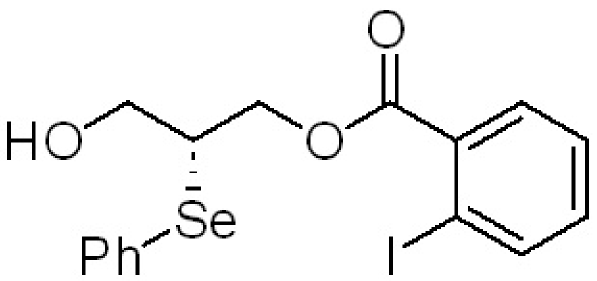

3d

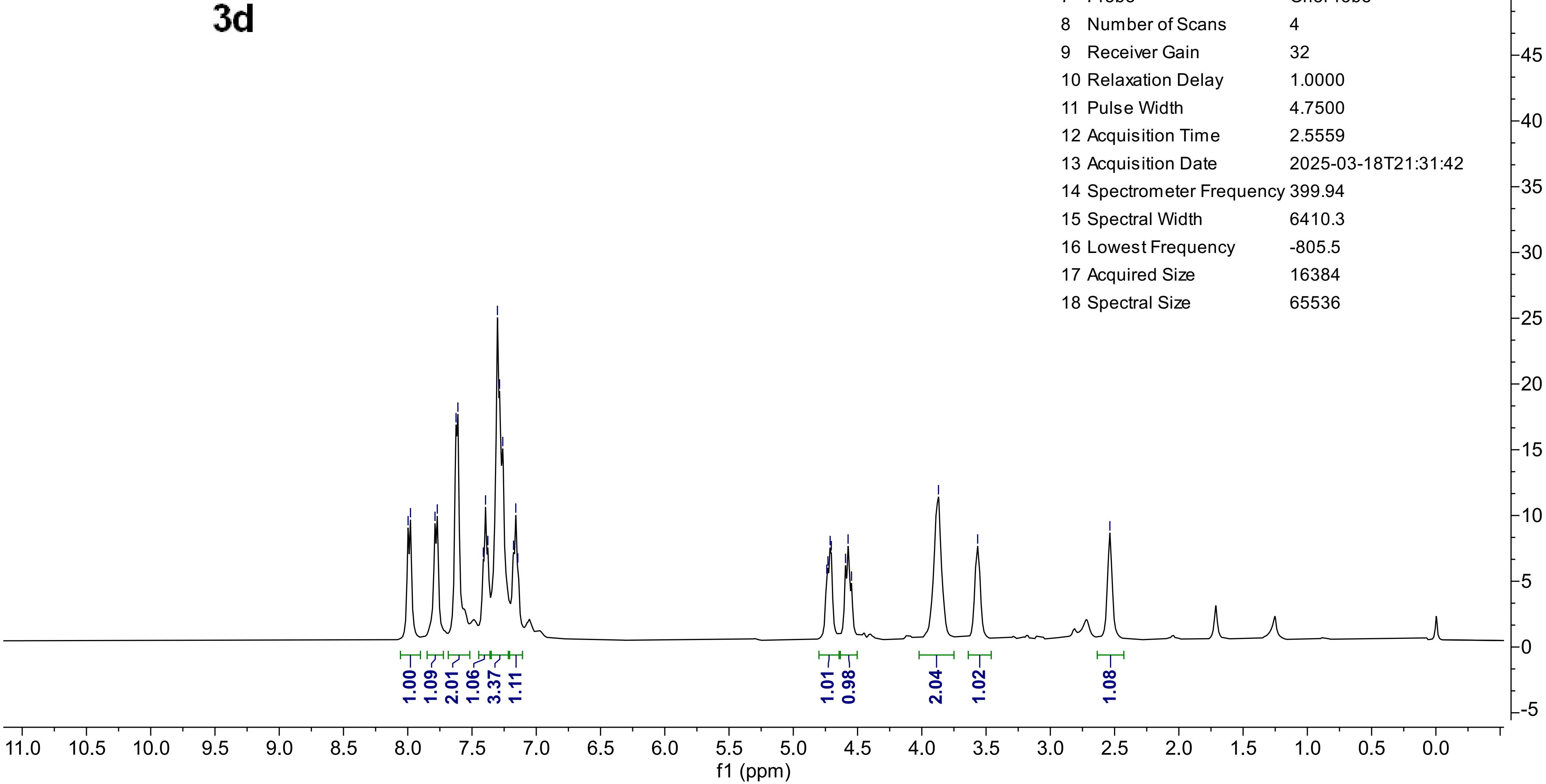

	Parameter	Value
1	Origin	Varian
2	Spectrometer	vnmrs
3	Solvent	$CDCl_3$
4	Temperature	25.0
5	Pulse Sequence	s2pul
6	Experiment	1D
7	Probe	OneProbe
8	Number of Scans	4
9	Receiver Gain	32
10	Relaxation Delay	1.0000
11	Pulse Width	4.7500
12	Acquisition Time	2.5559
13	Acquisition Date	2025-03-18T21:31:42
14	Spectrometer Frequency	399.94
15	Spectral Width	6410.3
16	Lowest Frequency	-805.5
17	Acquired Size	16384
18	Spectral Size	65536

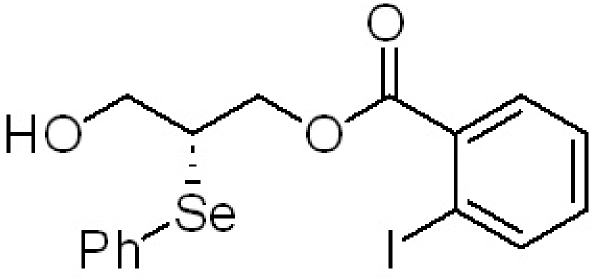

3d

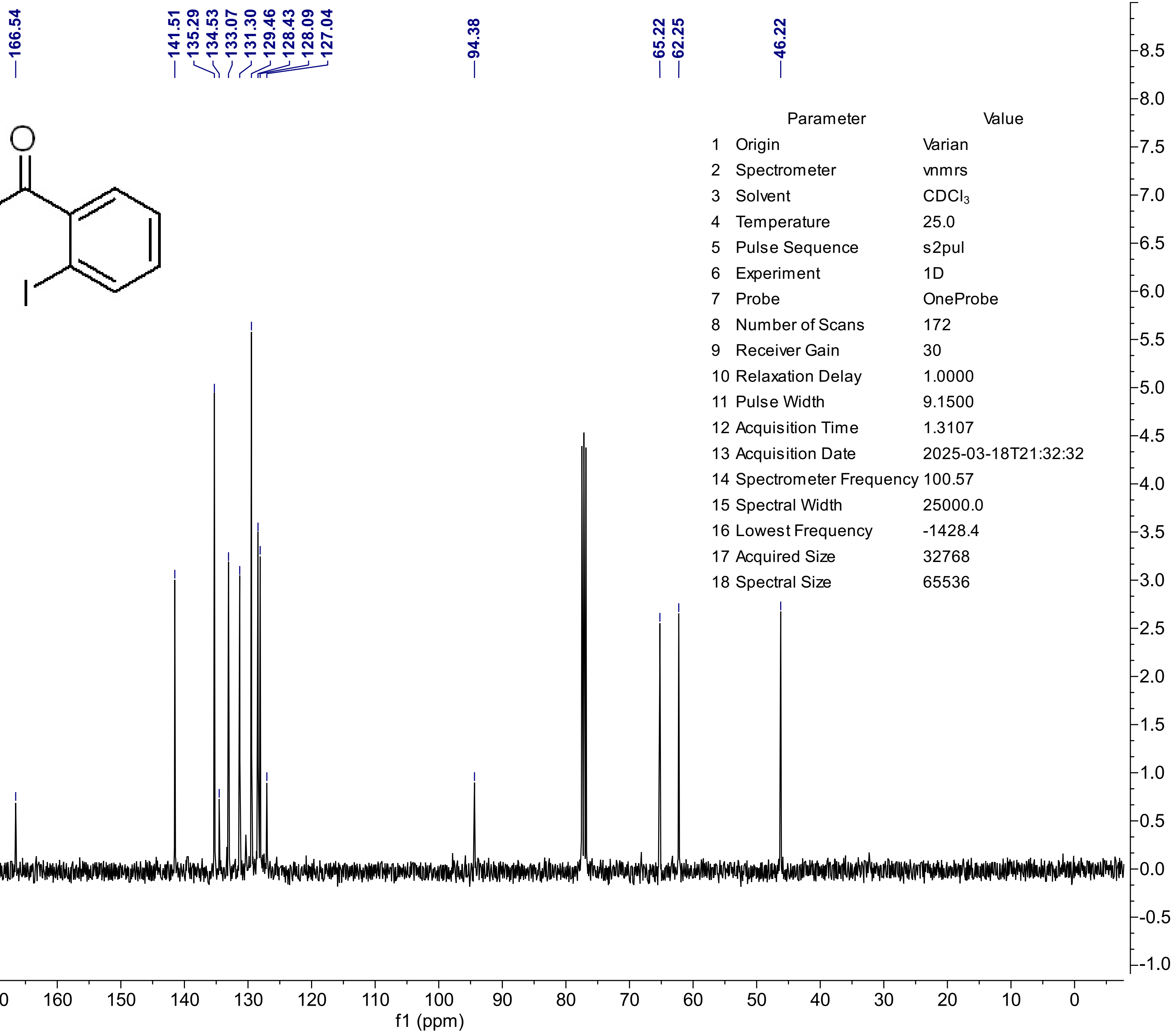

	Parameter	Value
1	Origin	Varian
2	Spectrometer	vnmrs
3	Solvent	$CDCl_3$
4	Temperature	25.0
5	Pulse Sequence	s2pul
6	Experiment	1D
7	Probe	OneProbe
8	Number of Scans	172
9	Receiver Gain	30
10	Relaxation Delay	1.0000
11	Pulse Width	9.1500
12	Acquisition Time	1.3107
13	Acquisition Date	2025-03-18T21:32:32
14	Spectrometer Frequency	100.57
15	Spectral Width	25000.0
16	Lowest Frequency	-1428.4
17	Acquired Size	32768
18	Spectral Size	65536

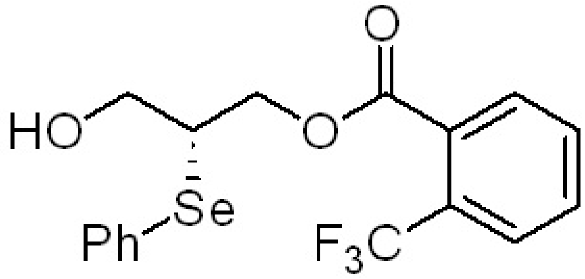

3e

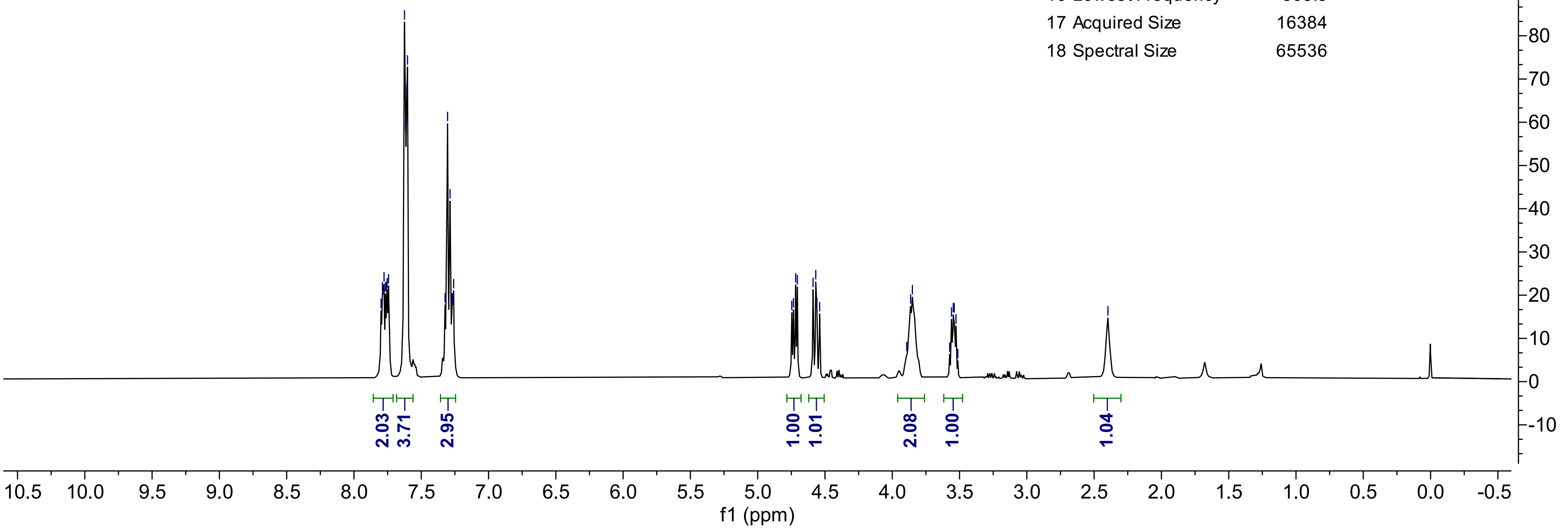

	Parameter	Value
1	Origin	Varian
2	Spectrometer	vnmrs
3	Solvent	$CDCl_3$
4	Temperature	25.0
5	Pulse Sequence	s2pul
6	Experiment	1D
7	Probe	OneProbe
8	Number of Scans	4
9	Receiver Gain	36
10	Relaxation Delay	1.0000
11	Pulse Width	4.7500
12	Acquisition Time	2.5559
13	Acquisition Date	2025-03-27T12:58:44
14	Spectrometer Frequency	399.94
15	Spectral Width	6410.3
16	Lowest Frequency	-805.5
17	Acquired Size	16384
18	Spectral Size	65536

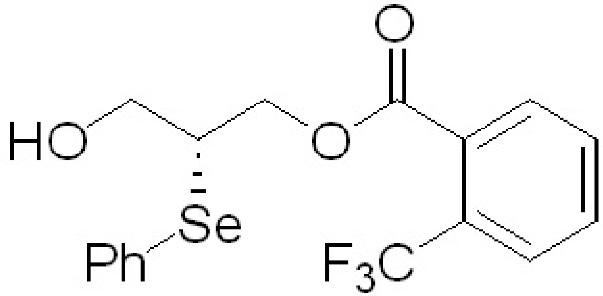

3e

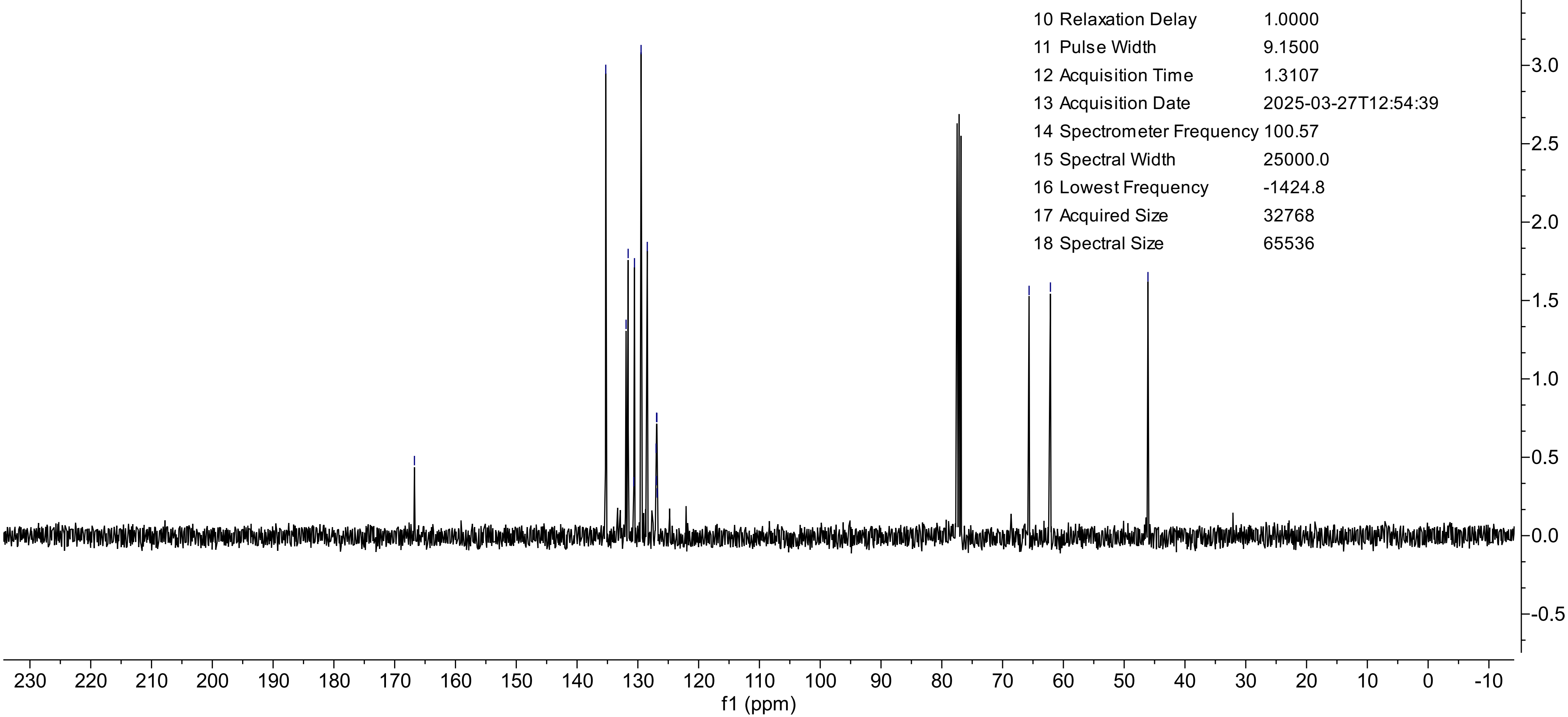

	Parameter	Value
1	Origin	Varian
2	Spectrometer	vnmrs
3	Solvent	$CDCl_3$
4	Temperature	25.0
5	Pulse Sequence	s2pul
6	Experiment	1D
7	Probe	OneProbe
8	Number of Scans	76
9	Receiver Gain	30
10	Relaxation Delay	1.0000
11	Pulse Width	9.1500
12	Acquisition Time	1.3107
13	Acquisition Date	2025-03-27T12:54:39
14	Spectrometer Frequency	100.57
15	Spectral Width	25000.0
16	Lowest Frequency	-1424.8
17	Acquired Size	32768
18	Spectral Size	65536

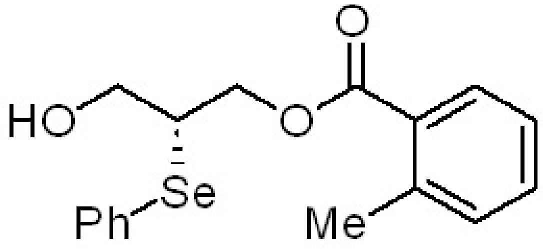

3f

	Parameter	Value
1	Origin	Varian
2	Spectrometer	vnmrs
3	Solvent	$CDCl_3$
4	Temperature	25.0
5	Pulse Sequence	s2pul
6	Experiment	1D
7	Probe	OneProbe
8	Number of Scans	4
9	Receiver Gain	36
10	Relaxation Delay	1.0000
11	Pulse Width	4.7500
12	Acquisition Time	2.5559
13	Acquisition Date	2026-03-19 T12:38:31
14	Spectrometer Frequency	399.94
15	Spectral Width	6410.3
16	Lowest Frequency	-805.5
17	Acquired Size	16384
18	Spectral Size	65536

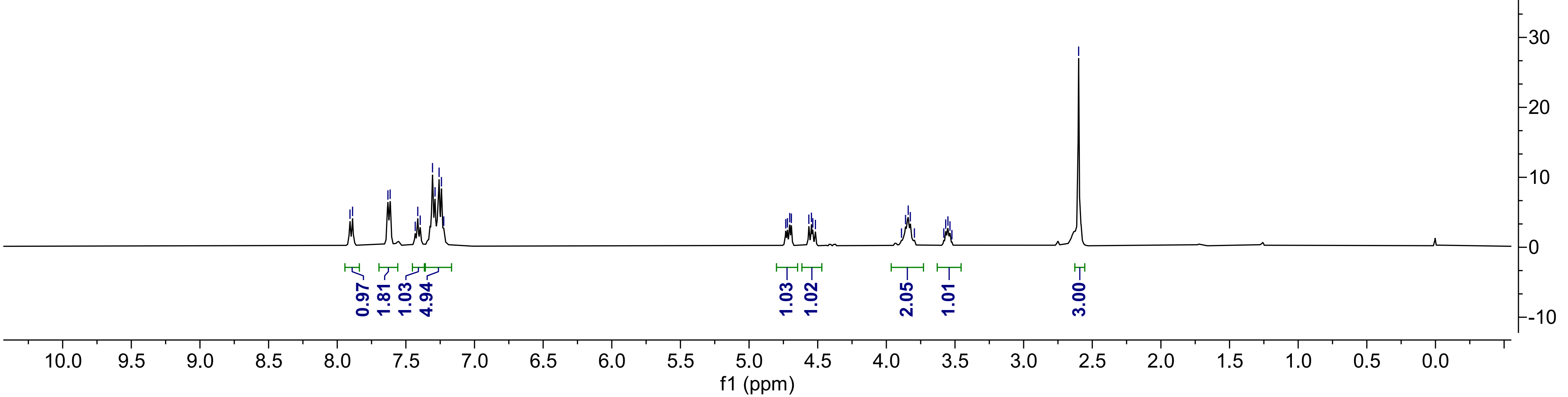

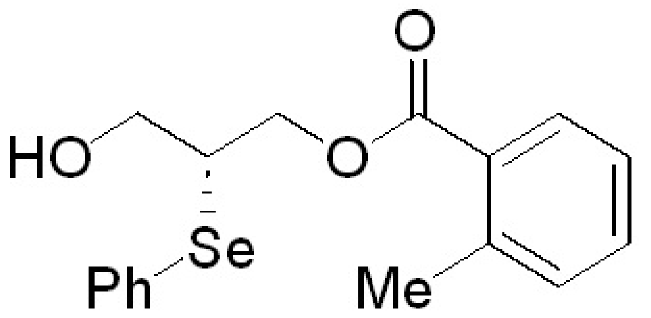

3f

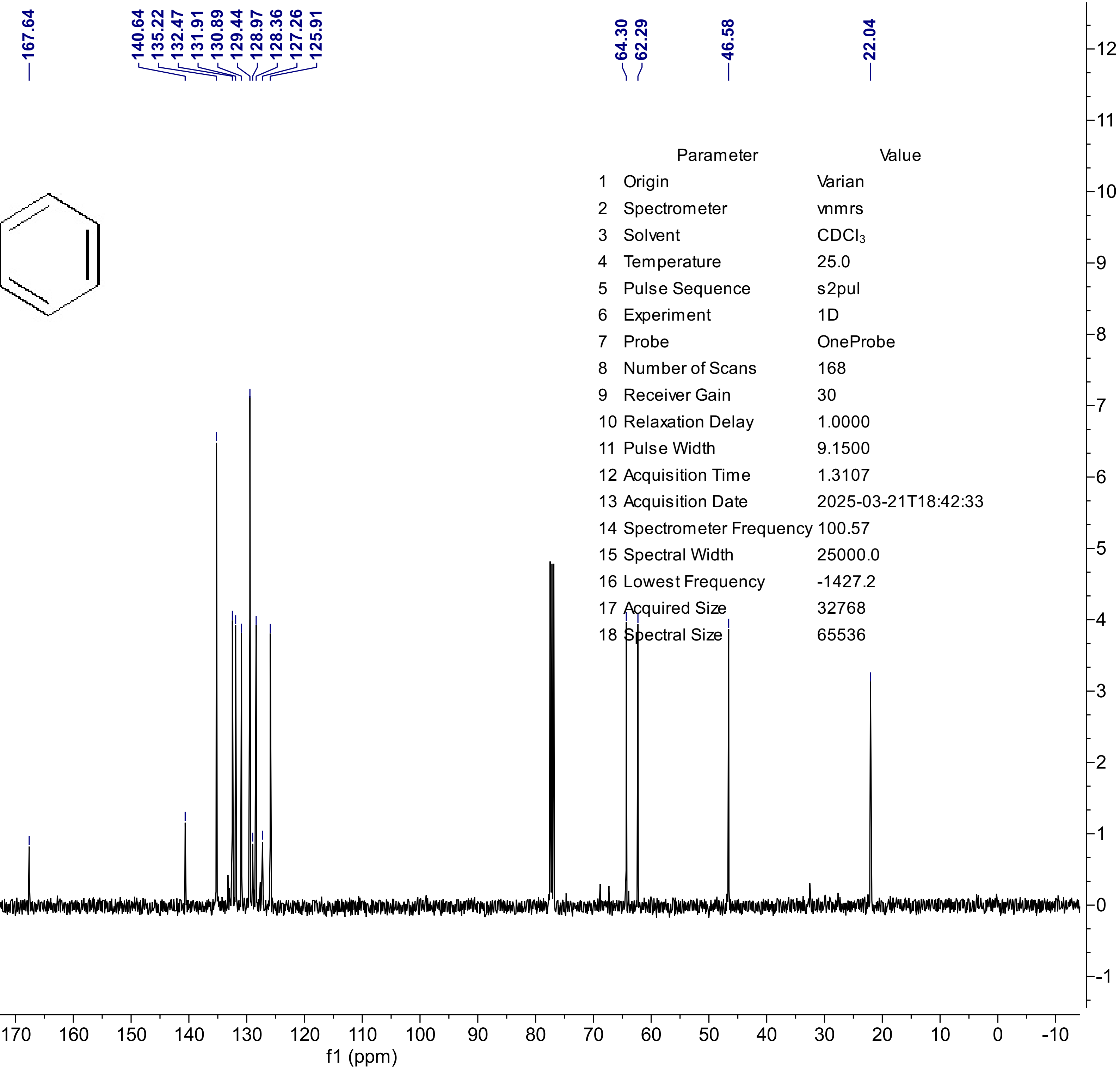

	Parameter	Value
1	Origin	Varian
2	Spectrometer	vnmrs
3	Solvent	$CDCl_3$
4	Temperature	25.0
5	Pulse Sequence	s2pul
6	Experiment	1D
7	Probe	OneProbe
8	Number of Scans	168
9	Receiver Gain	30
10	Relaxation Delay	1.0000
11	Pulse Width	9.1500
12	Acquisition Time	1.3107
13	Acquisition Date	2025-03-21T18:42:33
14	Spectrometer Frequency	100.57
15	Spectral Width	25000.0
16	Lowest Frequency	-1427.2
17	Acquired Size	32768
18	Spectral Size	65536

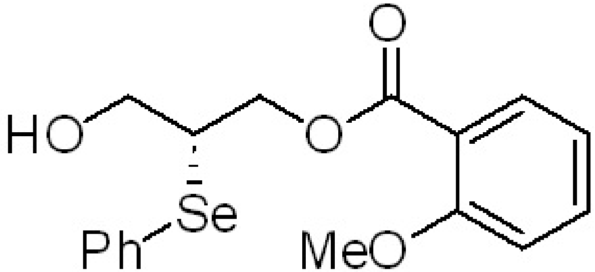

3g

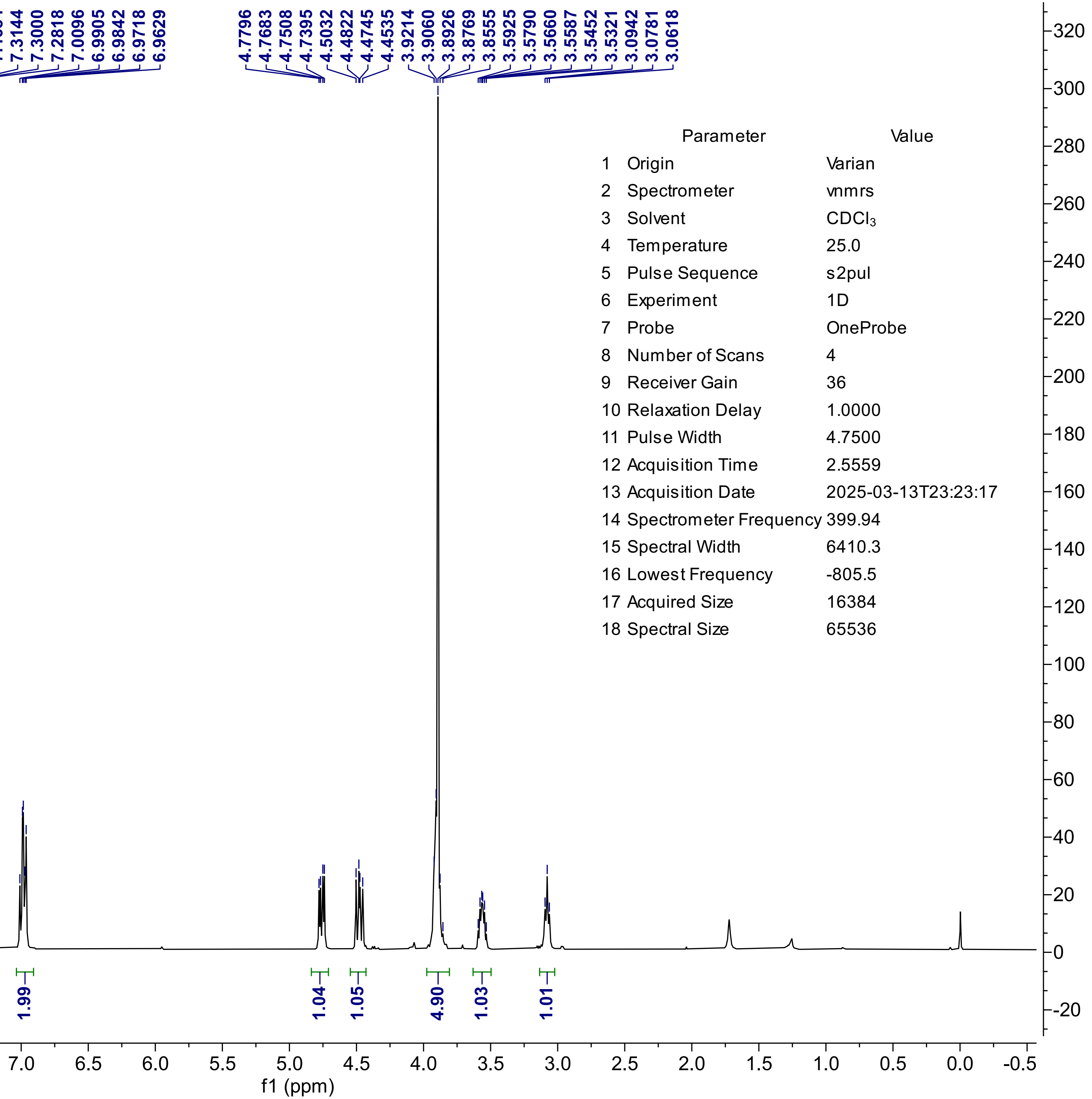

	Parameter	Value
1	Origin	Varian
2	Spectrometer	vnmrs
3	Solvent	$CDCl_3$
4	Temperature	25.0
5	Pulse Sequence	s2pul
6	Experiment	1D
7	Probe	OneProbe
8	Number of Scans	4
9	Receiver Gain	36
10	Relaxation Delay	1.0000
11	Pulse Width	4.7500
12	Acquisition Time	2.5559
13	Acquisition Date	2025-03-13T23:23:17
14	Spectrometer Frequency	399.94
15	Spectral Width	6410.3
16	Lowest Frequency	-805.5
17	Acquired Size	16384
18	Spectral Size	65536

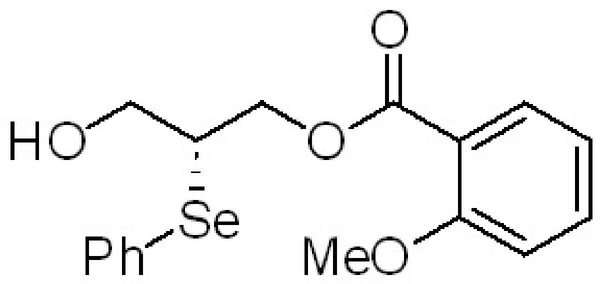

3g

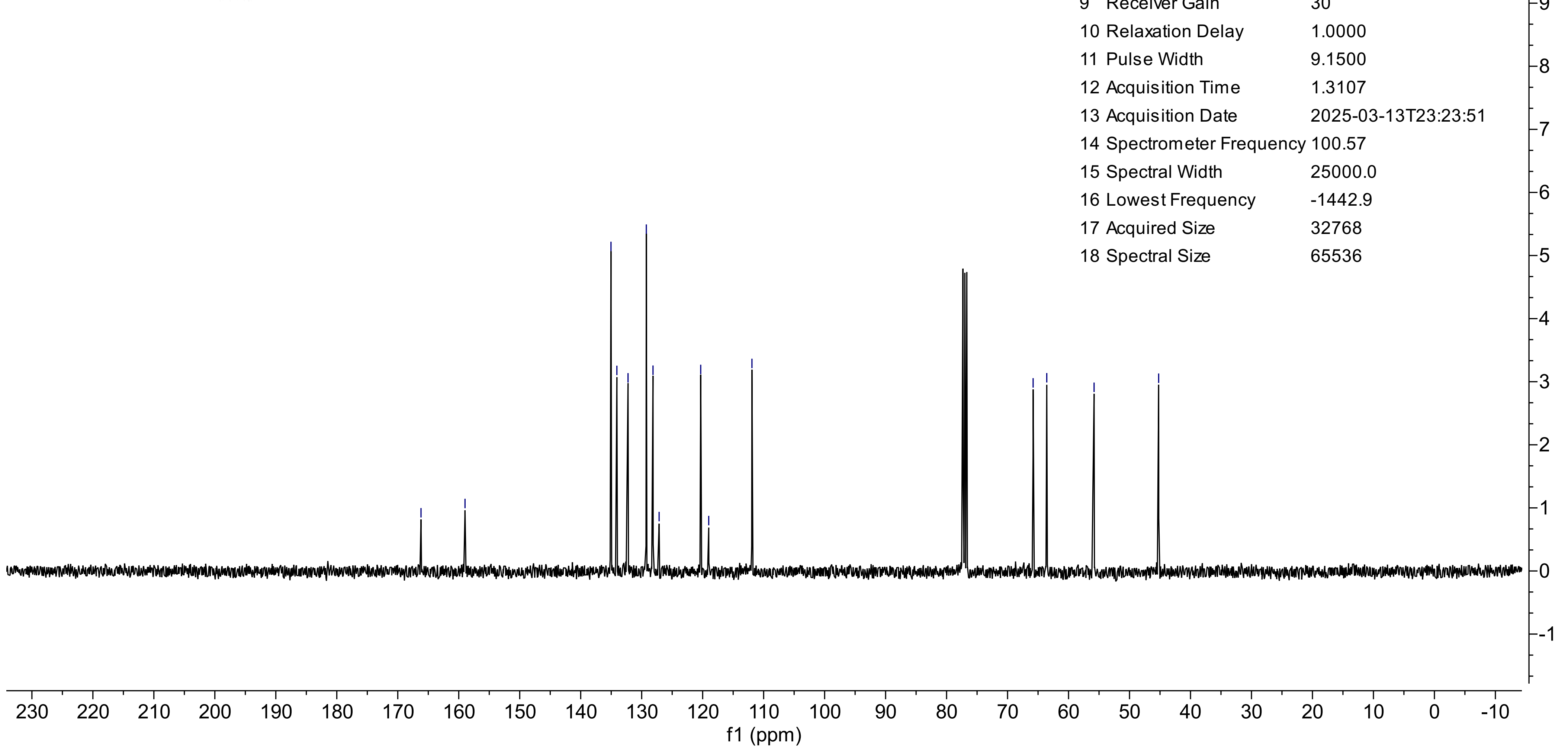

	Parameter	Value
1	Origin	Varian
2	Spectrometer	vnmrs
3	Solvent	$CDCl_3$
4	Temperature	25.0
5	Pulse Sequence	s2pul
6	Experiment	1D
7	Probe	OneProbe
8	Number of Scans	140
9	Receiver Gain	30
10	Relaxation Delay	1.0000
11	Pulse Width	9.1500
12	Acquisition Time	1.3107
13	Acquisition Date	2025-03-13T23:23:51
14	Spectrometer Frequency	100.57
15	Spectral Width	25000.0
16	Lowest Frequency	-1442.9
17	Acquired Size	32768
18	Spectral Size	65536

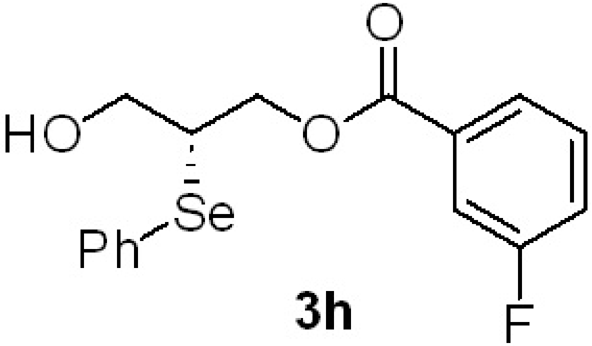

3h

	Parameter	Value
1	Origin	Varian
2	Spectrometer	vnmrs
3	Solvent	$CDCl_3$
4	Temperature	25.0
5	Pulse Sequence	s2pul
6	Experiment	1D
7	Probe	OneProbe
8	Number of Scans	4
9	Receiver Gain	28
10	Relaxation Delay	1.0000
11	Pulse Width	4.7500
12	Acquisition Time	2.5559
13	Acquisition Date	2025-03-15T23:24:39
14	Spectrometer Frequency	399.94
15	Spectral Width	6410.3
16	Lowest Frequency	-805.5
17	Acquired Size	16384
18	Spectral Size	65536

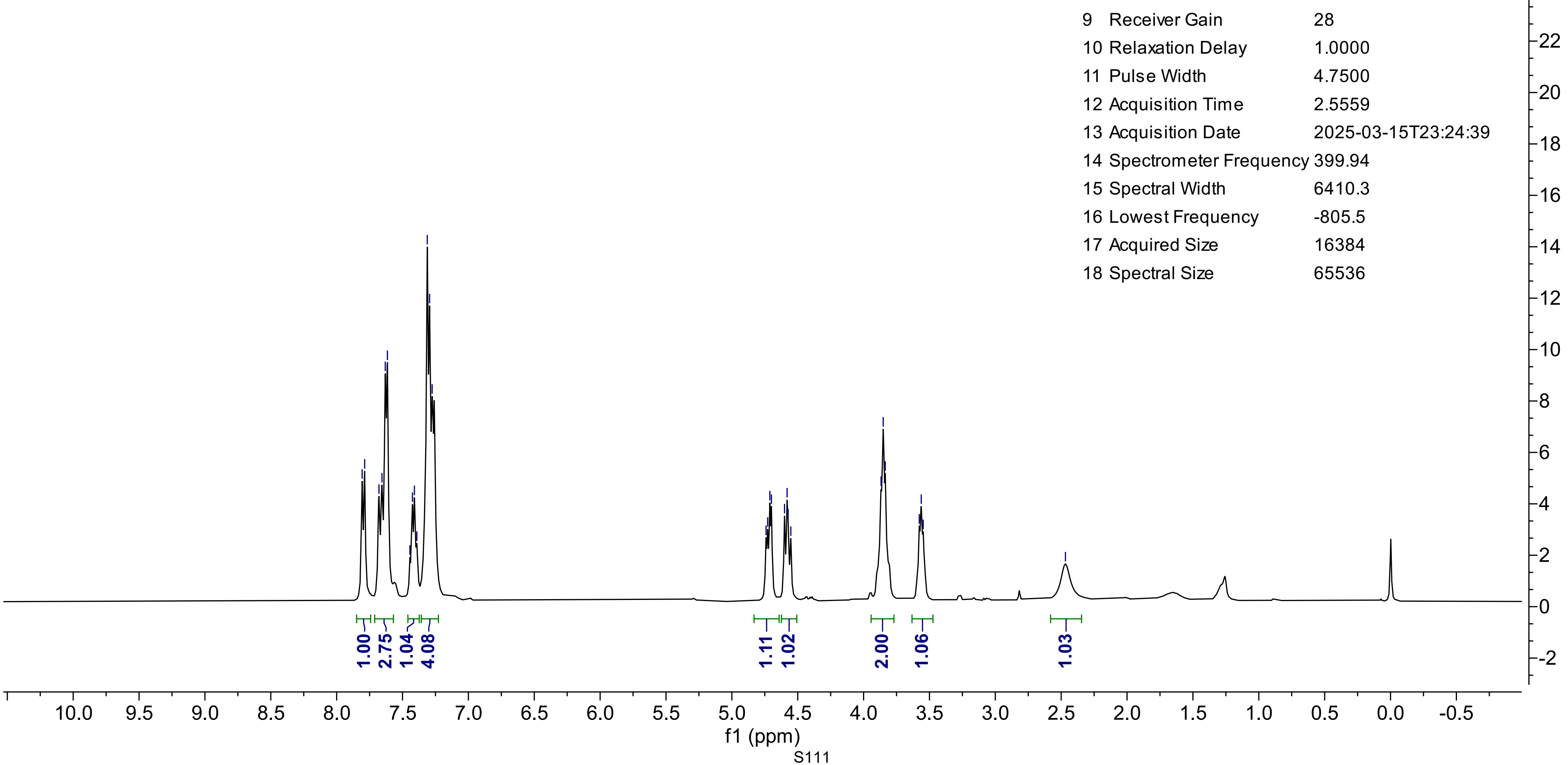

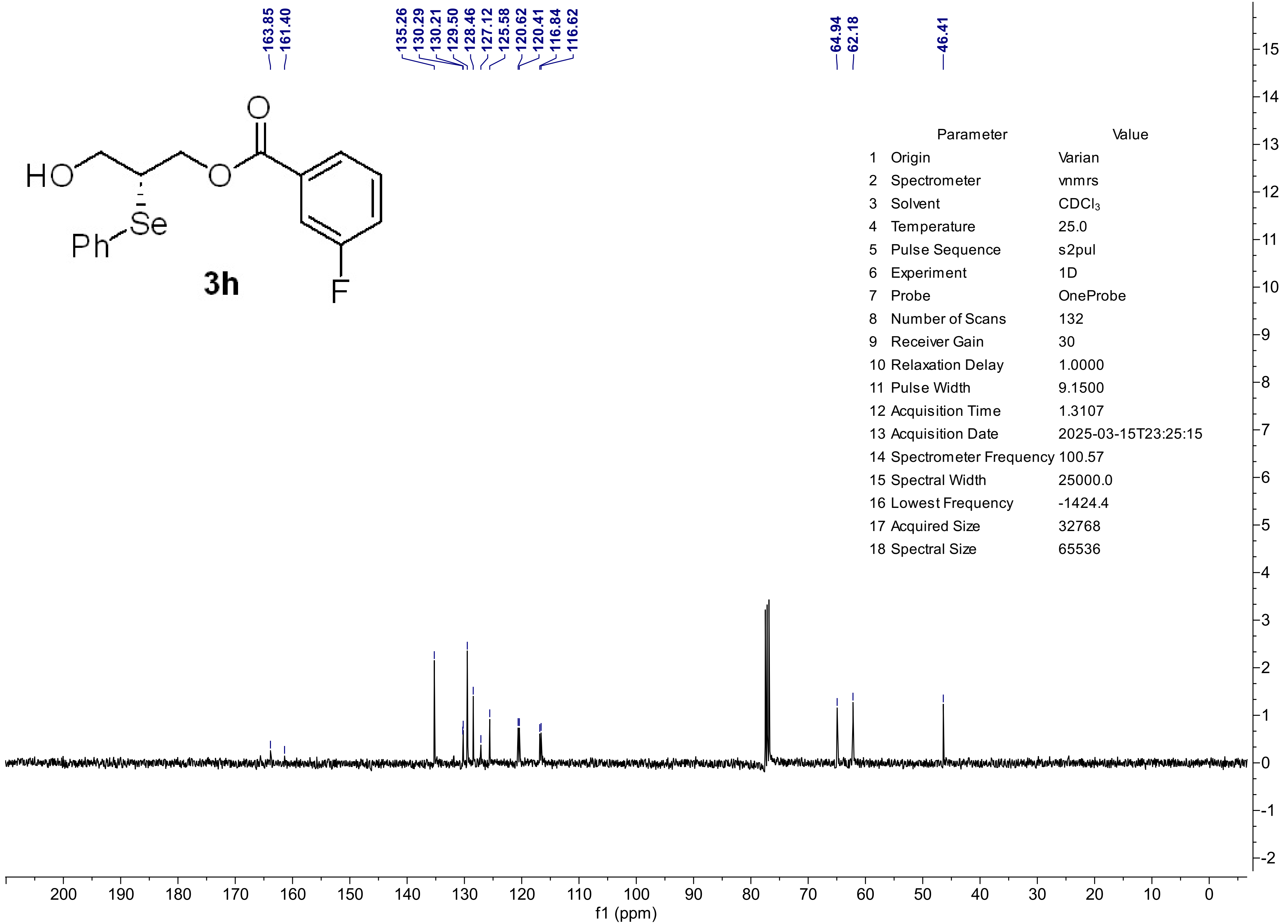

	Parameter	Value
1	Origin	Varian
2	Spectrometer	vnmrs
3	Solvent	$CDCl_3$
4	Temperature	25.0
5	Pulse Sequence	s2pul
6	Experiment	1D
7	Probe	OneProbe
8	Number of Scans	132
9	Receiver Gain	30
10	Relaxation Delay	1.0000
11	Pulse Width	9.1500
12	Acquisition Time	1.3107
13	Acquisition Date	2025-03-15T23:25:15
14	Spectrometer Frequency	100.57
15	Spectral Width	25000.0
16	Lowest Frequency	-1424.4
17	Acquired Size	32768
18	Spectral Size	65536

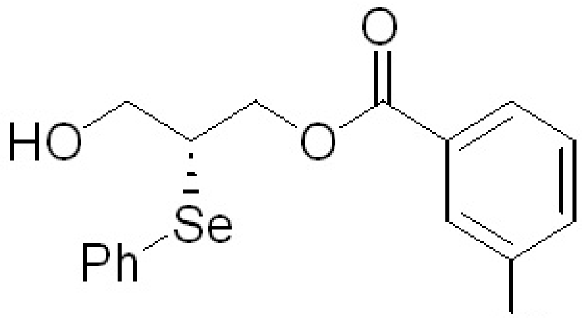

3i

	Parameter	Value
1	Origin	Varian
2	Spectrometer	vnmrs
3	Solvent	$CDCl_3$
4	Temperature	25.0
5	Pulse Sequence	s2pul
6	Experiment	1D
7	Probe	OneProbe
8	Number of Scans	4
9	Receiver Gain	32
10	Relaxation Delay	1.0000
11	Pulse Width	4.7500
12	Acquisition Time	2.5559
13	Acquisition Date	2025-03-21T16:51:35
14	Spectrometer Frequency	399.94
15	Spectral Width	6410.3
16	Lowest Frequency	-805.5
17	Acquired Size	16384
18	Spectral Size	65536

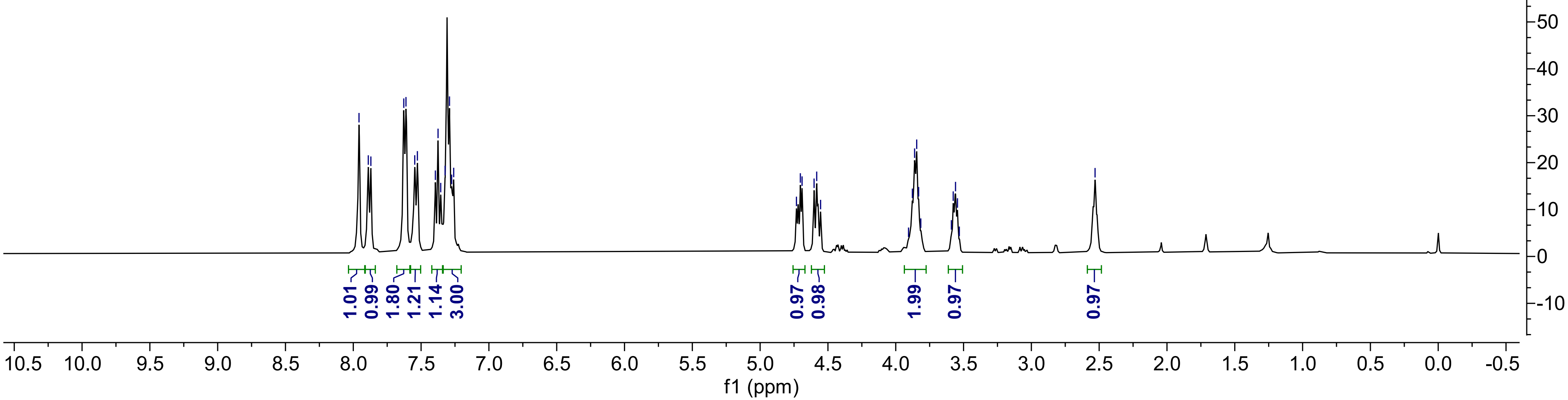

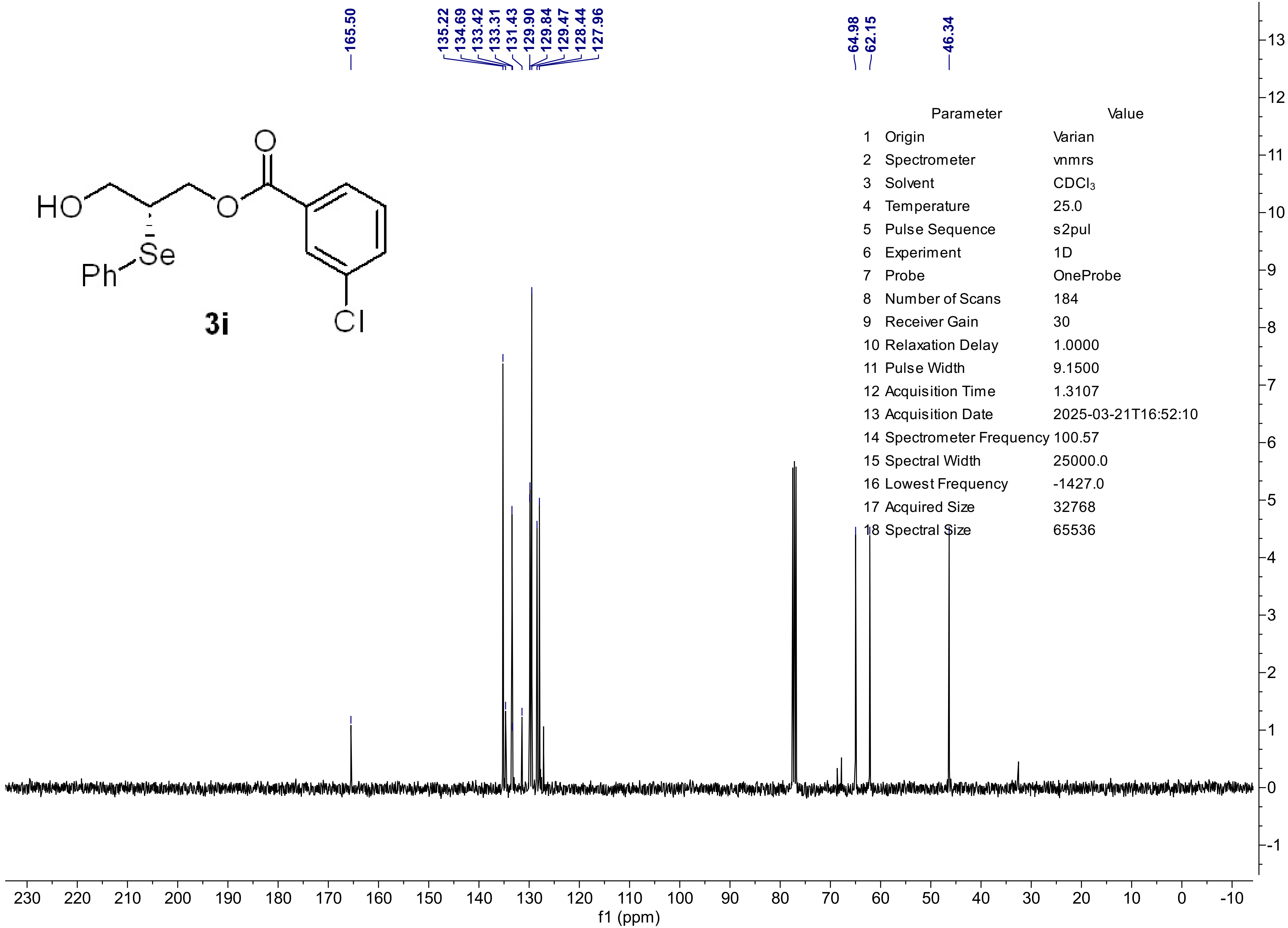
165.50
135.22
134.69
133.42
133.31
131.43
129.90
129.84
129.47
128.44
127.96
64.98
62.15
46.34
HO
O
O
Se
Ph
Cl
3i
Parameter Value
1 Origin Varian
2 Spectrometer vnmrs
3 Solvent CDCl3
4 Temperature 25.0
5 Pulse Sequence s2pul
6 Experiment 1D
7 Probe OneProbe
8 Number of Scans 184
9 Receiver Gain 30
10 Relaxation Delay 1.0000
11 Pulse Width 9.1500
12 Acquisition Time 1.3107
13 Acquisition Date 2025-03-21T16:52:10
14 Spectrometer Frequency 100.57
15 Spectral Width 25000.0
16 Lowest Frequency -1427.0
17 Acquired Size 32768
18 Spectral Size 65536
f1 (ppm)

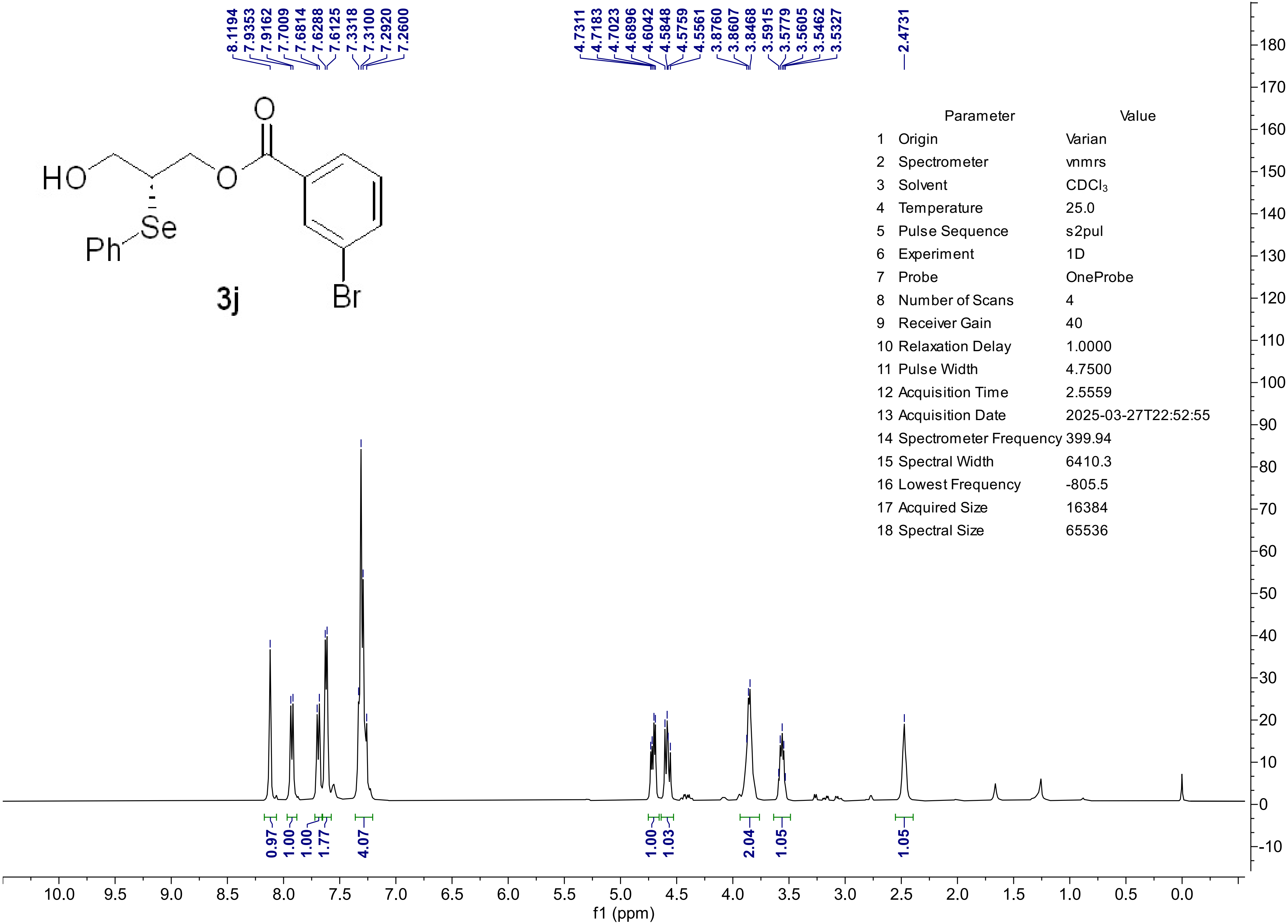

8.1194
7.9353
7.9162
7.7009
7.6814
7.6288
7.6125
7.3318
7.3100
7.2920
7.2600
4.7311
4.7183
4.7023
4.6896
4.6042
4.5848
4.5759
4.5561
3.8760
3.8607
3.8468
3.5915
3.5779
3.5605
3.5462
3.5327
2.4731
HO
O
O
Se
Ph
3j
Br
Parameter | Value
1 Origin | Varian
2 Spectrometer | vnmrs
3 Solvent | CDCl3
4 Temperature | 25.0
5 Pulse Sequence | s2pul
6 Experiment | 1D
7 Probe | OneProbe
8 Number of Scans | 4
9 Receiver Gain | 40
10 Relaxation Delay | 1.0000
11 Pulse Width | 4.7500
12 Acquisition Time | 2.5559
13 Acquisition Date | 2025-03-27T22:52:55
14 Spectrometer Frequency | 399.94
15 Spectral Width | 6410.3
16 Lowest Frequency | -805.5
17 Acquired Size | 16384
18 Spectral Size | 65536
0.97
1.00
1.00
1.77
4.07
1.00
1.03
2.04
1.05
1.05
10.0 9.5 9.0 8.5 8.0 7.5 7.0 6.5 6.0 5.5 5.0 4.5 4.0 3.5 3.0 2.5 2.0 1.5 1.0 0.5 0.0
f1 (ppm)
180 170 160 150 140 130 120 110 100 90 80 70 60 50 40 30 20 10 0 -10

HQQ-3-129-C
Gradient Shimming

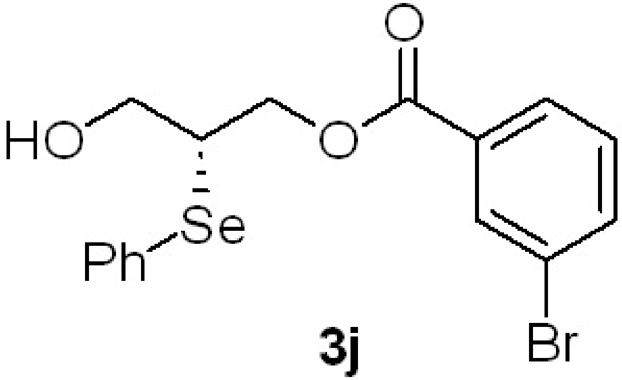

3j

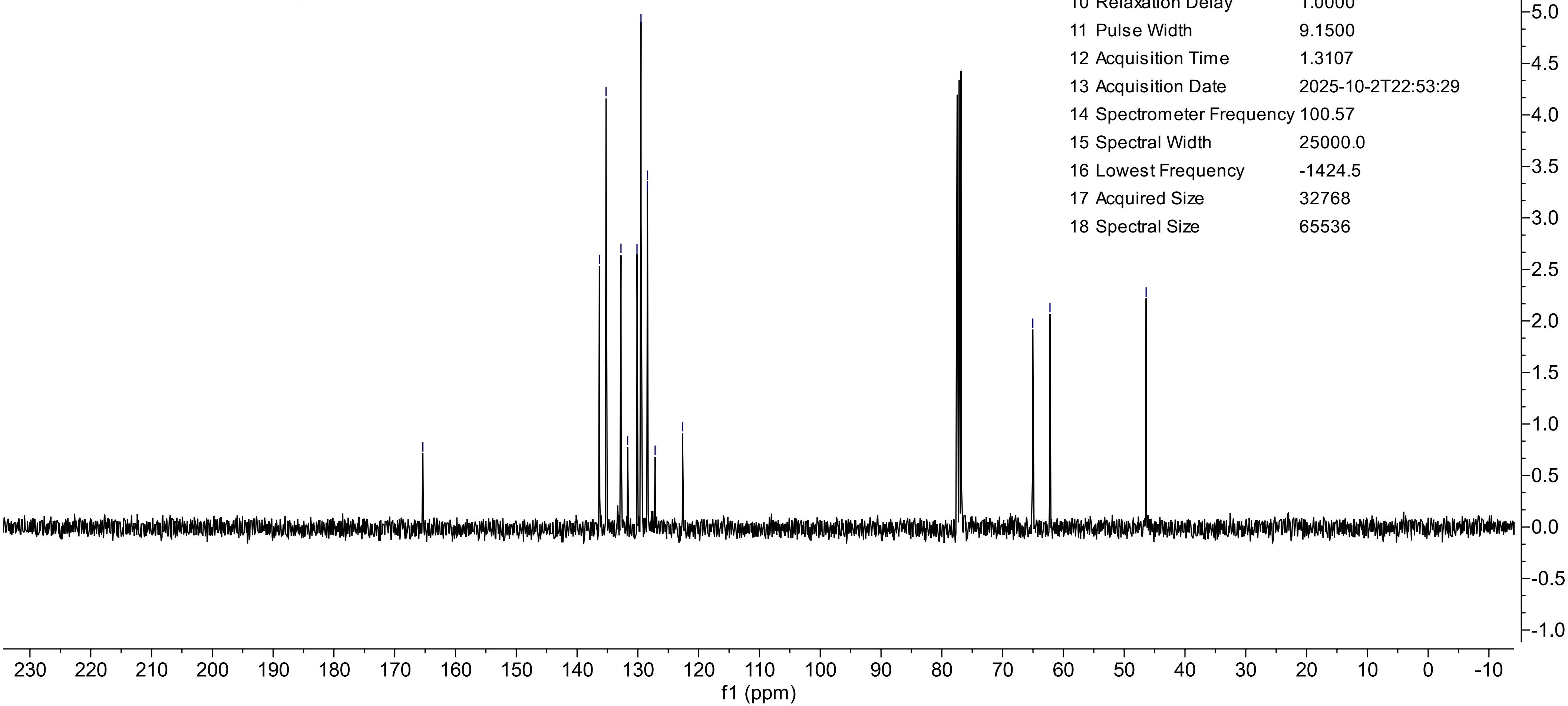

	Parameter	Value
1	Origin	Varian
2	Spectrometer	vnmrs
3	Solvent	$CDCl_3$
4	Temperature	25.0
5	Pulse Sequence	s2pul
6	Experiment	1D
7	Probe	OneProbe
8	Number of Scans	144
9	Receiver Gain	30
10	Relaxation Delay	1.0000
11	Pulse Width	9.1500
12	Acquisition Time	1.3107
13	Acquisition Date	2025-10-2T22:53:29
14	Spectrometer Frequency	100.57
15	Spectral Width	25000.0
16	Lowest Frequency	-1424.5
17	Acquired Size	32768
18	Spectral Size	65536

HQQ-2-107-H
Gradient Shimming

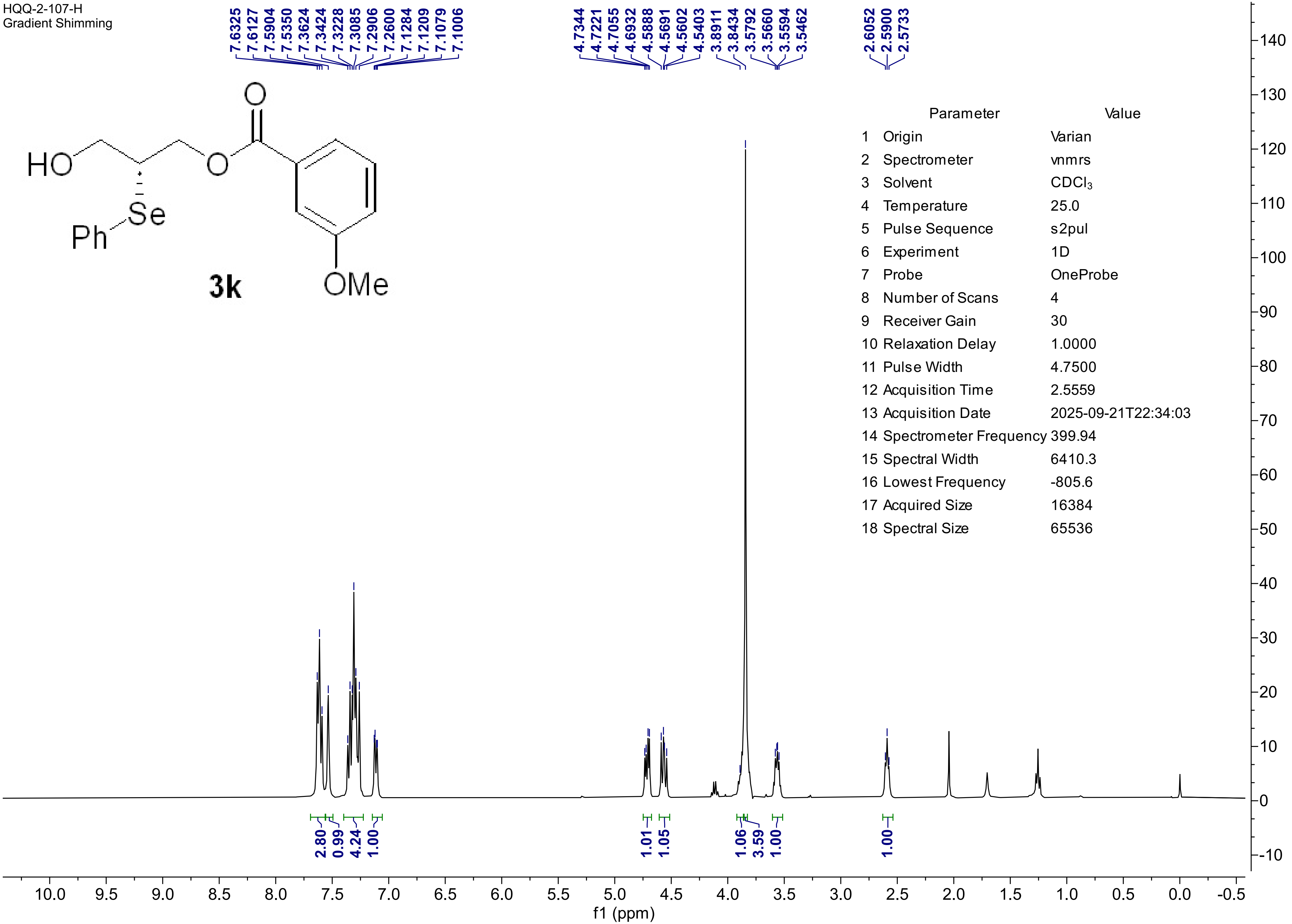

	Parameter	Value
1	Origin	Varian
2	Spectrometer	vnmrs
3	Solvent	$CDCl_3$
4	Temperature	25.0
5	Pulse Sequence	s2pul
6	Experiment	1D
7	Probe	OneProbe
8	Number of Scans	4
9	Receiver Gain	30
10	Relaxation Delay	1.0000
11	Pulse Width	4.7500
12	Acquisition Time	2.5559
13	Acquisition Date	2025-09-21T22:34:03
14	Spectrometer Frequency	399.94
15	Spectral Width	6410.3
16	Lowest Frequency	-805.6
17	Acquired Size	16384
18	Spectral Size	65536

HQQ-2-107-C
Gradient Shimming

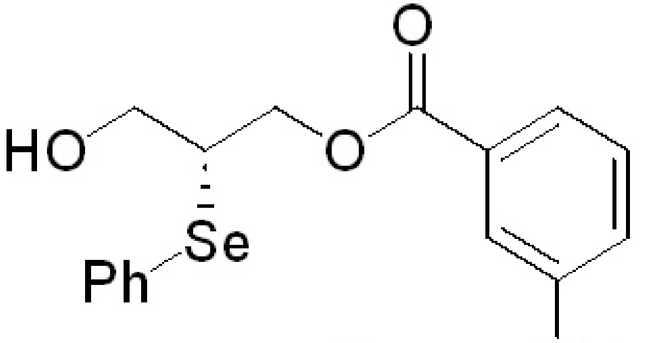

3k

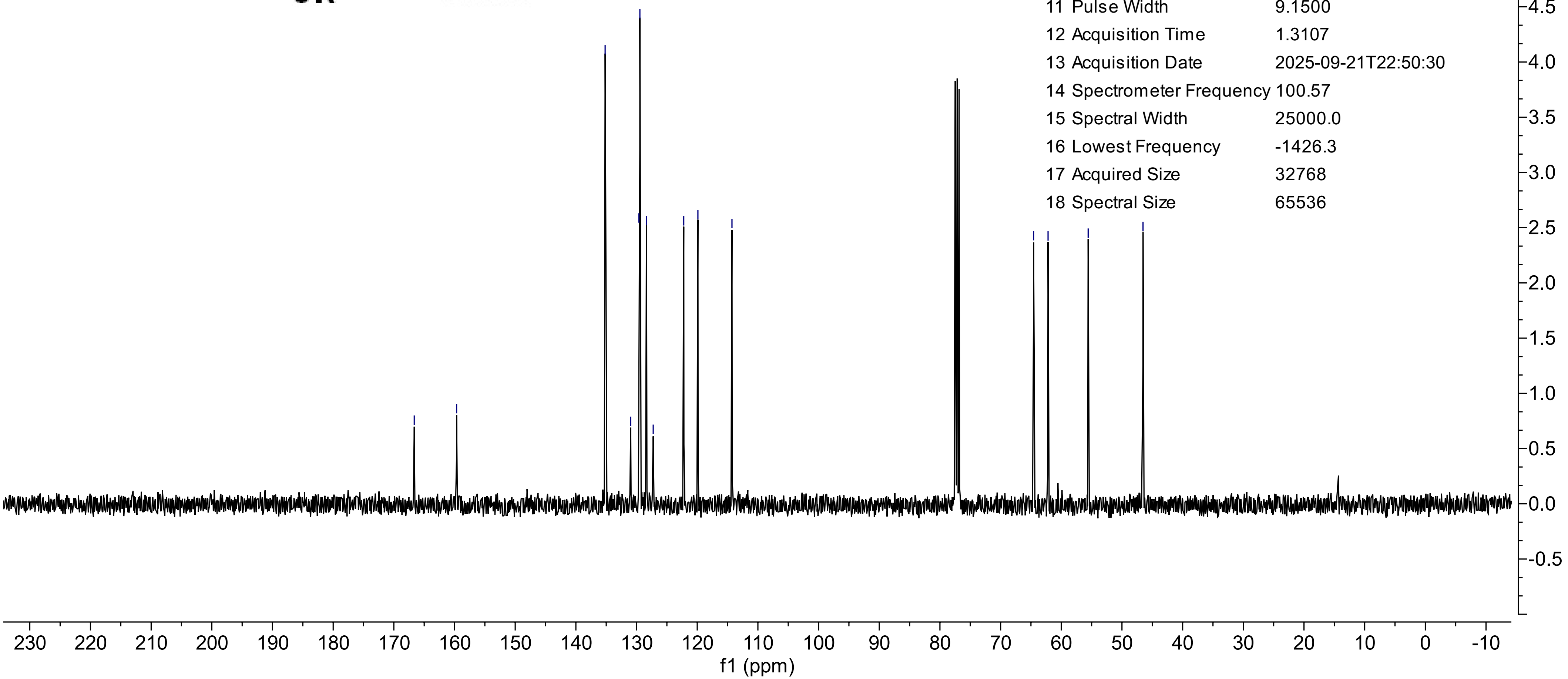

	Parameter	Value
1	Origin	Varian
2	Spectrometer	vnmrs
3	Solvent	$CDCl_3$
4	Temperature	25.0
5	Pulse Sequence	s2pul
6	Experiment	1D
7	Probe	OneProbe
8	Number of Scans	120
9	Receiver Gain	30
10	Relaxation Delay	1.0000
11	Pulse Width	9.1500
12	Acquisition Time	1.3107
13	Acquisition Date	2025-09-21T22:50:30
14	Spectrometer Frequency	100.57
15	Spectral Width	25000.0
16	Lowest Frequency	-1426.3
17	Acquired Size	32768
18	Spectral Size	65536

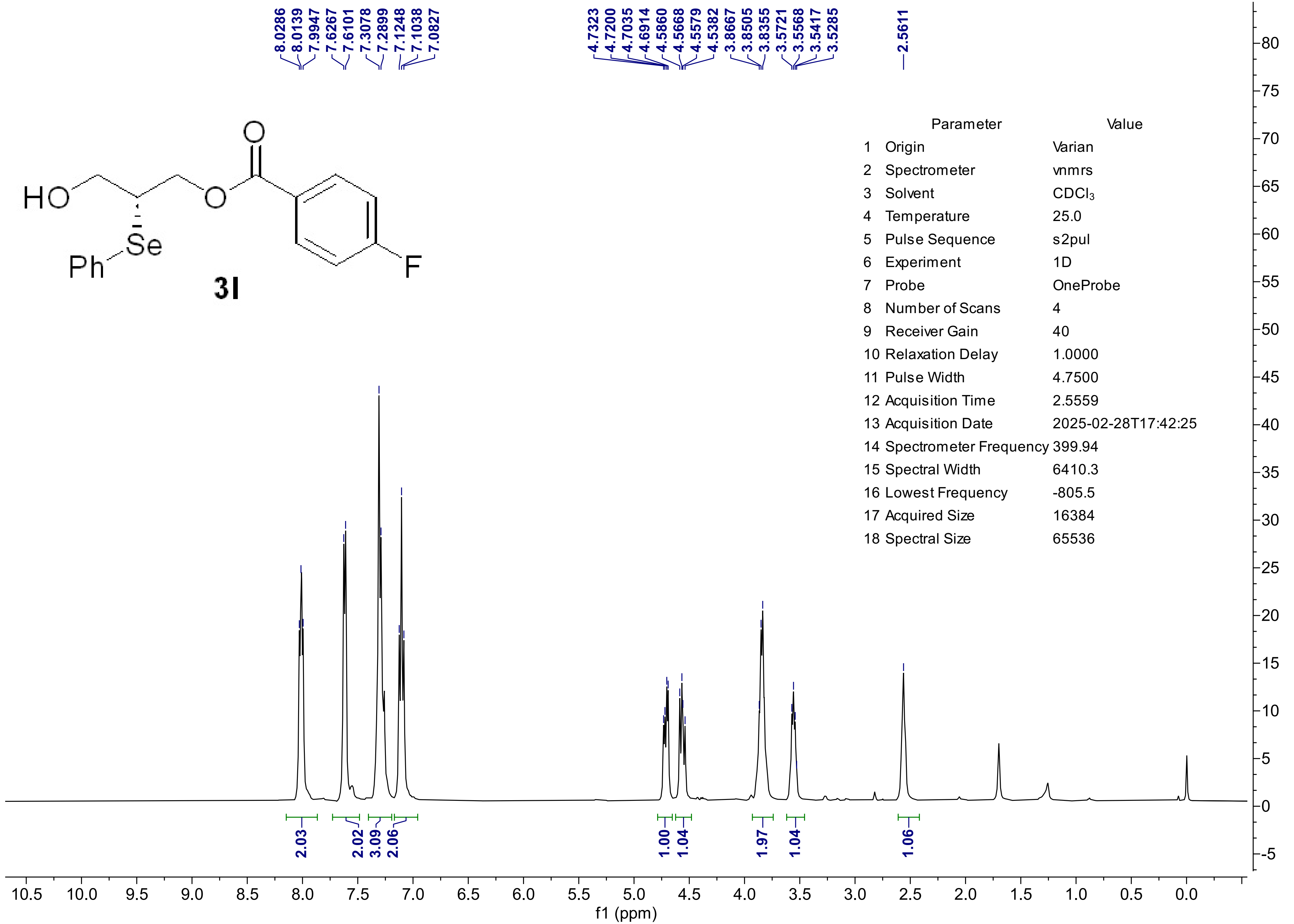

	Parameter	Value
1	Origin	Varian
2	Spectrometer	vnmrs
3	Solvent	$CDCl_3$
4	Temperature	25.0
5	Pulse Sequence	s2pul
6	Experiment	1D
7	Probe	OneProbe
8	Number of Scans	4
9	Receiver Gain	40
10	Relaxation Delay	1.0000
11	Pulse Width	4.7500
12	Acquisition Time	2.5559
13	Acquisition Date	2025-02-28T17:42:25
14	Spectrometer Frequency	399.94
15	Spectral Width	6410.3
16	Lowest Frequency	-805.5
17	Acquired Size	16384
18	Spectral Size	65536

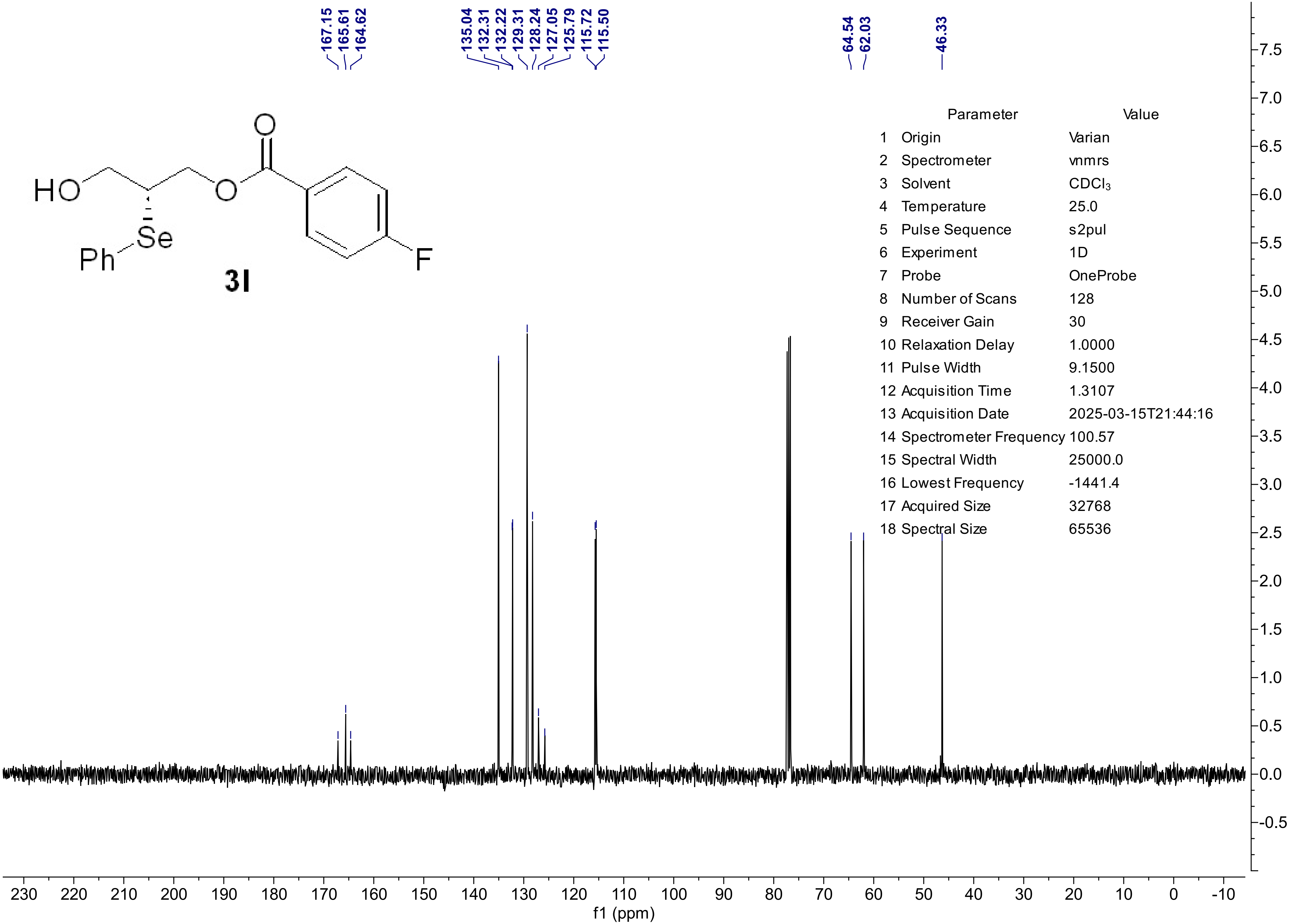

	Parameter	Value
1	Origin	Varian
2	Spectrometer	vnmrs
3	Solvent	$CDCl_3$
4	Temperature	25.0
5	Pulse Sequence	s2pul
6	Experiment	1D
7	Probe	OneProbe
8	Number of Scans	128
9	Receiver Gain	30
10	Relaxation Delay	1.0000
11	Pulse Width	9.1500
12	Acquisition Time	1.3107
13	Acquisition Date	2025-03-15T21:44:16
14	Spectrometer Frequency	100.57
15	Spectral Width	25000.0
16	Lowest Frequency	-1441.4
17	Acquired Size	32768
18	Spectral Size	65536

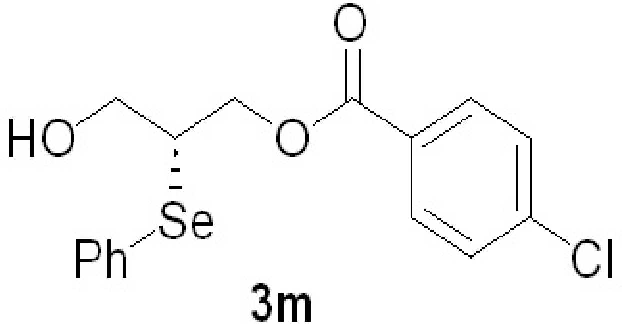

3m

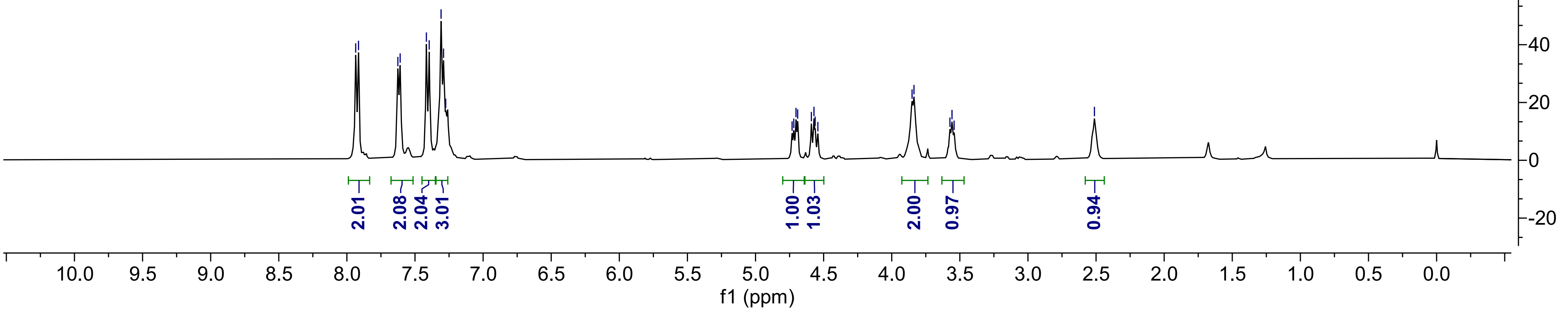

	Parameter	Value
1	Origin	Varian
2	Spectrometer	vnmrs
3	Solvent	$CDCl_3$
4	Temperature	25.0
5	Pulse Sequence	s2pul
6	Experiment	1D
7	Probe	OneProbe
8	Number of Scans	4
9	Receiver Gain	38
10	Relaxation Delay	1.0000
11	Pulse Width	4.7500
12	Acquisition Time	5.4526
13	Acquisition Date	2025-03-28T18:32:26
14	Spectrometer Frequency	399.94
15	Spectral Width	24038.5
16	Lowest Frequency	-12019.2
17	Acquired Size	131072
18	Spectral Size	262144

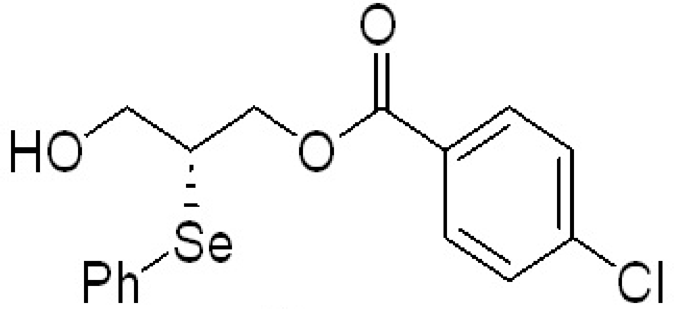

3m

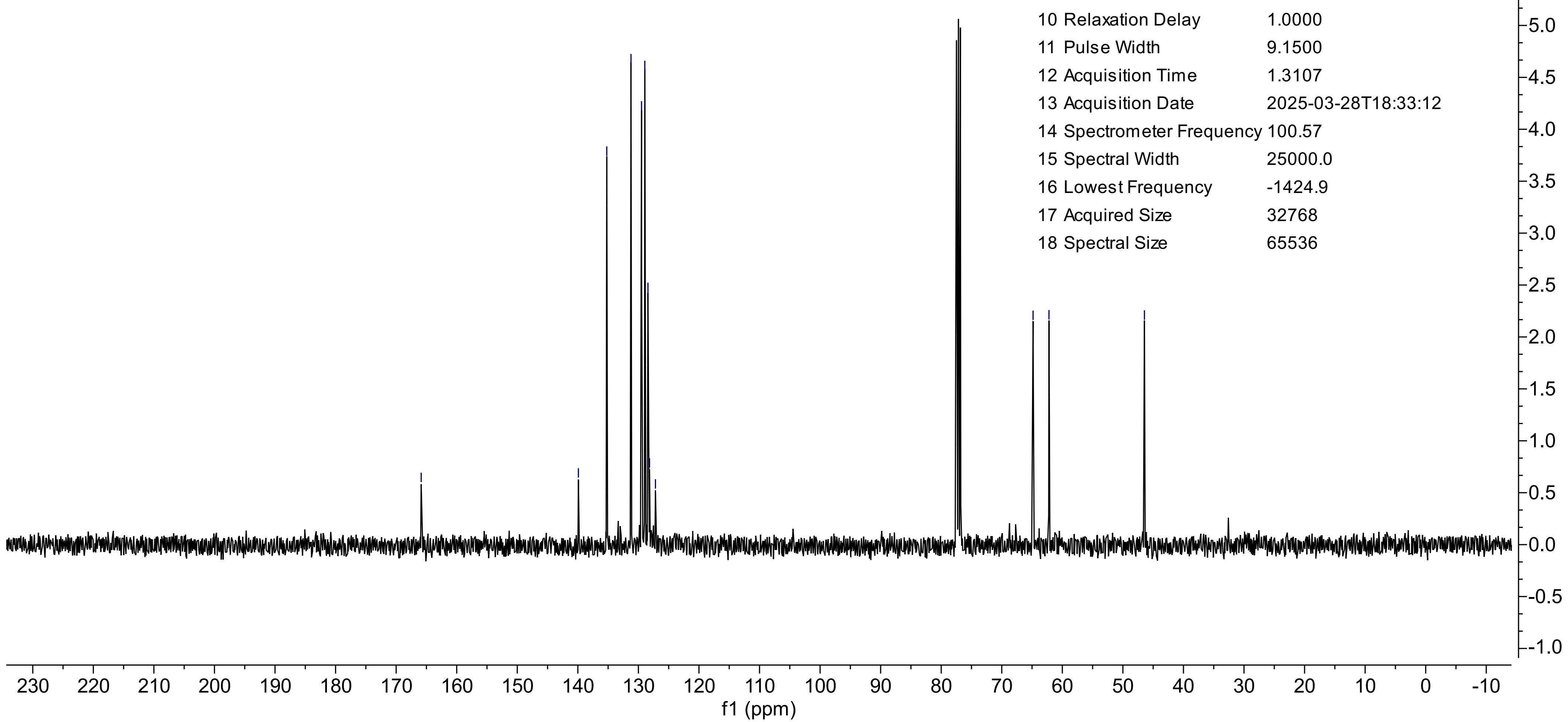

	Parameter	Value
1	Origin	Varian
2	Spectrometer	vnmrs
3	Solvent	$CDCl_3$
4	Temperature	25.0
5	Pulse Sequence	s2pul
6	Experiment	1D
7	Probe	OneProbe
8	Number of Scans	140
9	Receiver Gain	30
10	Relaxation Delay	1.0000
11	Pulse Width	9.1500
12	Acquisition Time	1.3107
13	Acquisition Date	2025-03-28T18:33:12
14	Spectrometer Frequency	100.57
15	Spectral Width	25000.0
16	Lowest Frequency	-1424.9
17	Acquired Size	32768
18	Spectral Size	65536

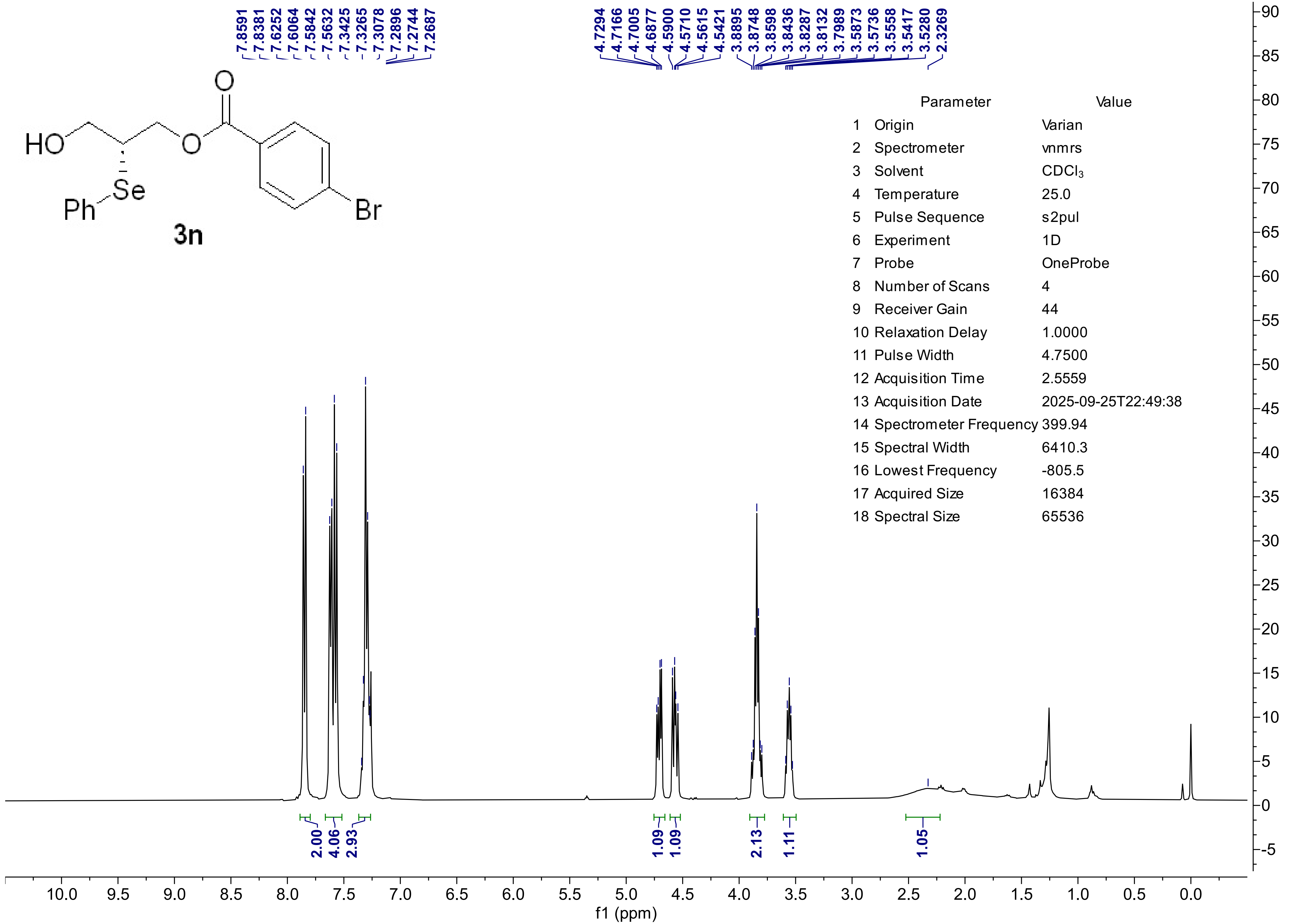

	Parameter	Value
1	Origin	Varian
2	Spectrometer	vnmrs
3	Solvent	$CDCl_3$
4	Temperature	25.0
5	Pulse Sequence	s2pul
6	Experiment	1D
7	Probe	OneProbe
8	Number of Scans	4
9	Receiver Gain	44
10	Relaxation Delay	1.0000
11	Pulse Width	4.7500
12	Acquisition Time	2.5559
13	Acquisition Date	2025-09-25T22:49:38
14	Spectrometer Frequency	399.94
15	Spectral Width	6410.3
16	Lowest Frequency	-805.5
17	Acquired Size	16384
18	Spectral Size	65536

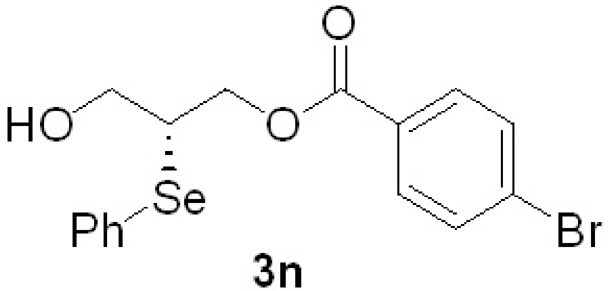

3n

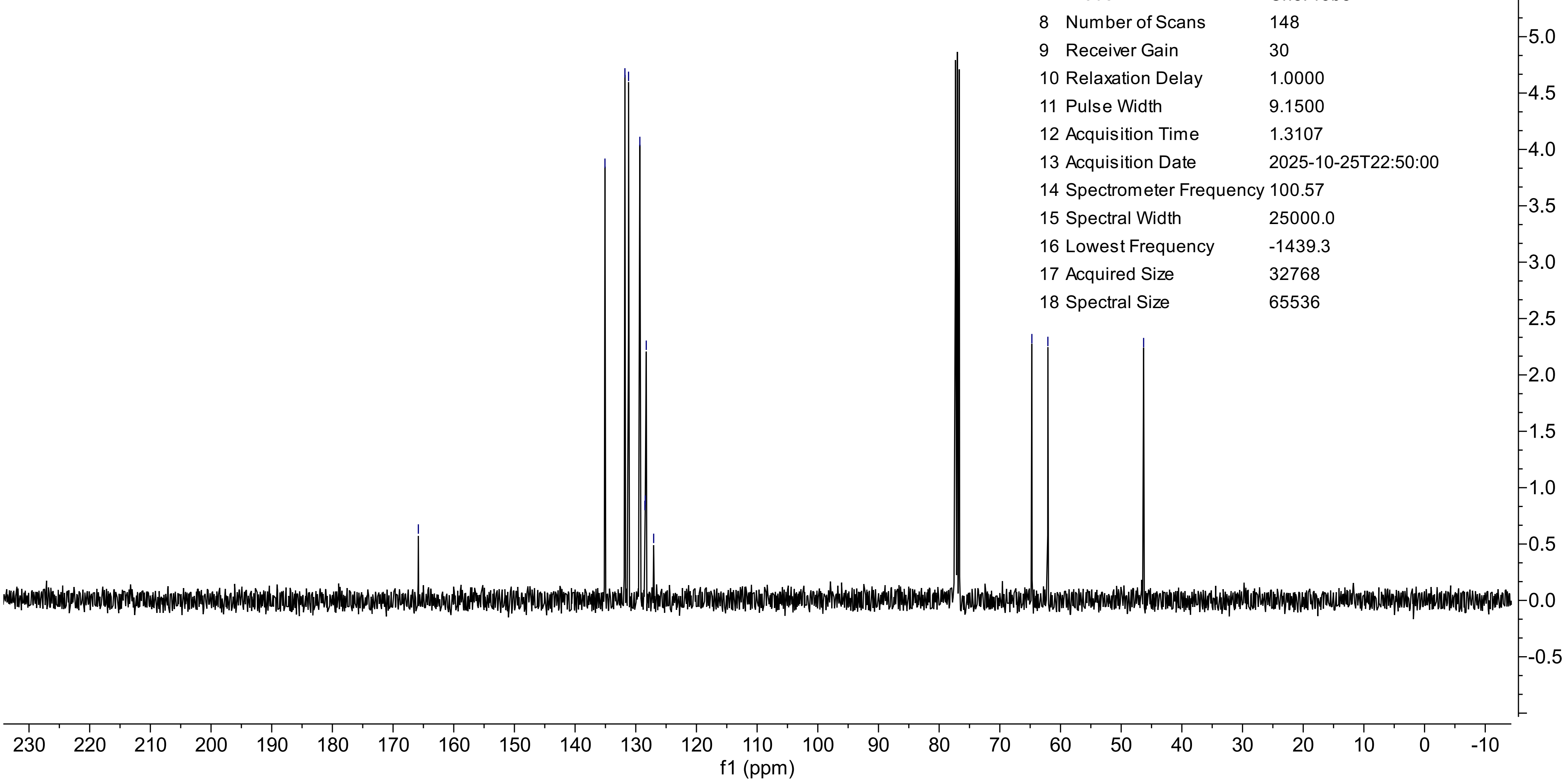

	Parameter	Value
1	Origin	Varian
2	Spectrometer	vnmrs
3	Solvent	$CDCl_3$
4	Temperature	25.0
5	Pulse Sequence	s2pul
6	Experiment	1D
7	Probe	OneProbe
8	Number of Scans	148
9	Receiver Gain	30
10	Relaxation Delay	1.0000
11	Pulse Width	9.1500
12	Acquisition Time	1.3107
13	Acquisition Date	2025-10-25T22:50:00
14	Spectrometer Frequency	100.57
15	Spectral Width	25000.0
16	Lowest Frequency	-1439.3
17	Acquired Size	32768
18	Spectral Size	65536

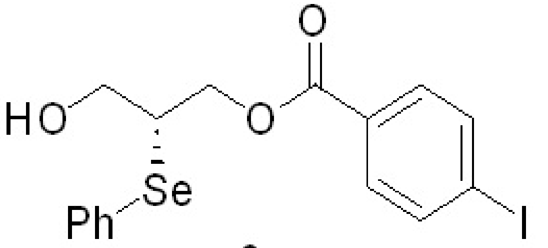

3o

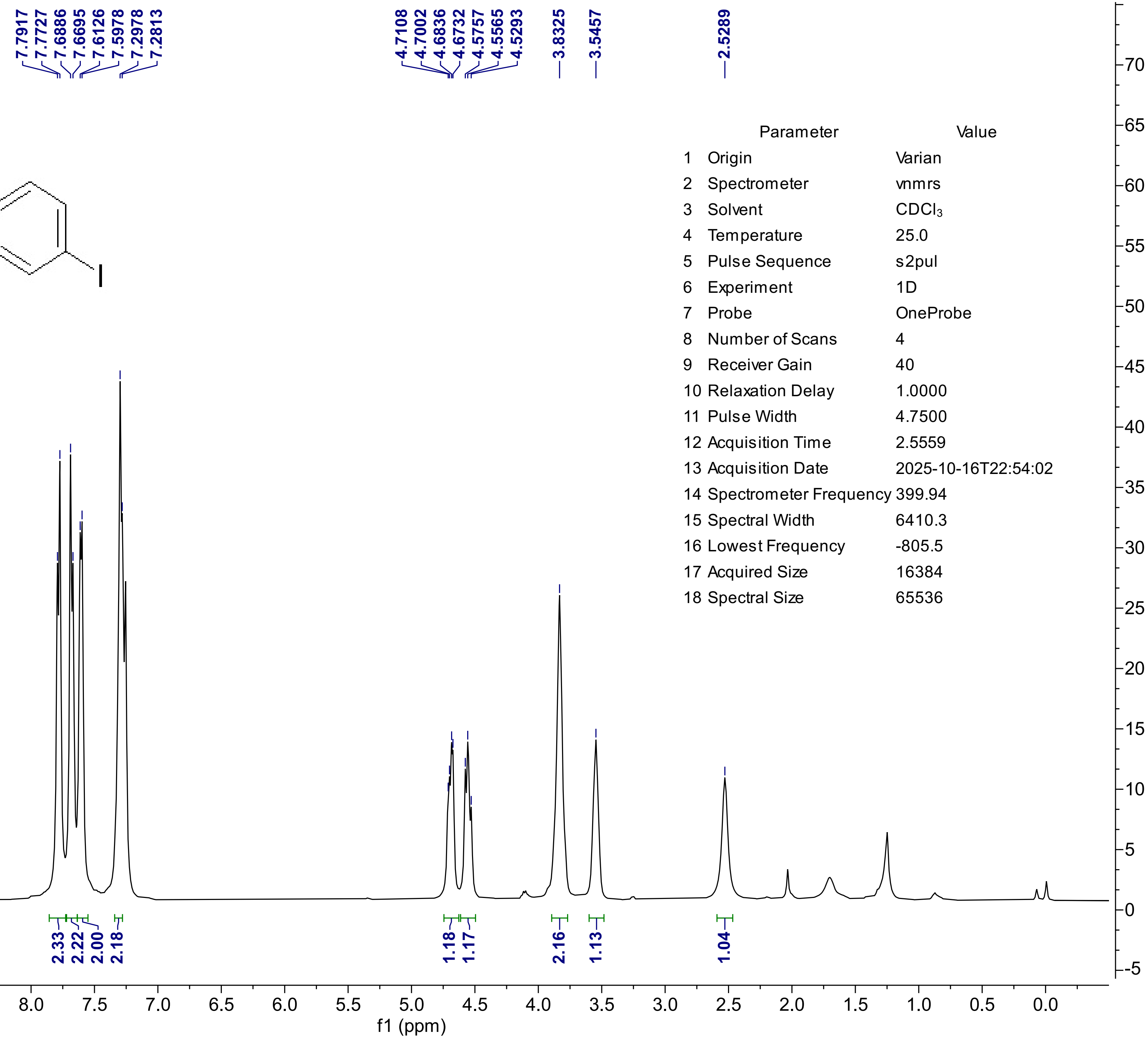

	Parameter	Value
1	Origin	Varian
2	Spectrometer	vnmrs
3	Solvent	$CDCl_3$
4	Temperature	25.0
5	Pulse Sequence	s2pul
6	Experiment	1D
7	Probe	OneProbe
8	Number of Scans	4
9	Receiver Gain	40
10	Relaxation Delay	1.0000
11	Pulse Width	4.7500
12	Acquisition Time	2.5559
13	Acquisition Date	2025-10-16T22:54:02
14	Spectrometer Frequency	399.94
15	Spectral Width	6410.3
16	Lowest Frequency	-805.5
17	Acquired Size	16384
18	Spectral Size	65536

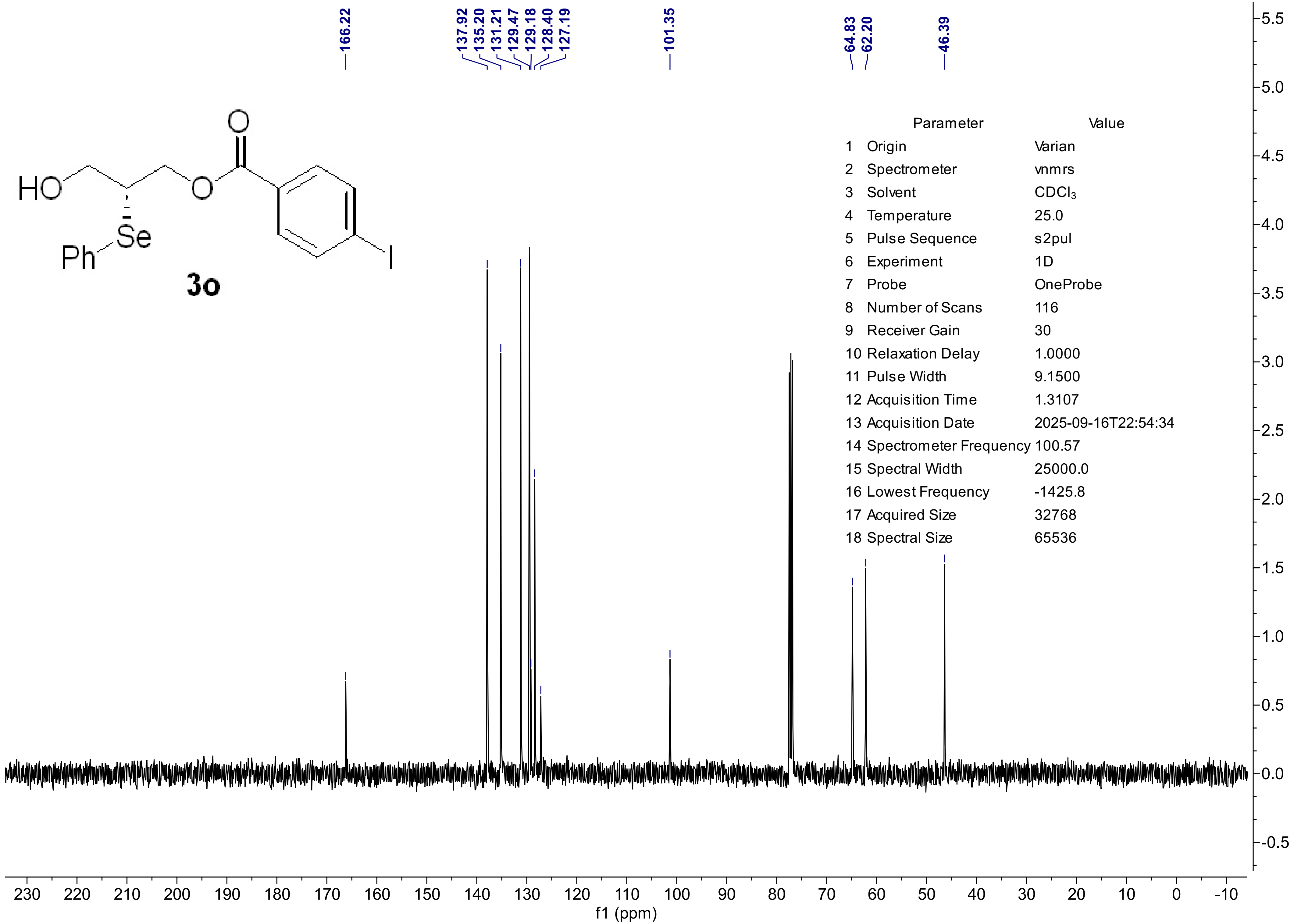
166.22
137.92
135.20
131.21
129.47
129.18
128.40
127.19
101.35
64.83
62.20
46.39
HO
O
O
Se
Ph
I
3o
Parameter
Value
1 Origin
Varian
2 Spectrometer
vnmrs
3 Solvent
CDCl3
4 Temperature
25.0
5 Pulse Sequence
s2pul
6 Experiment
1D
7 Probe
OneProbe
8 Number of Scans
116
9 Receiver Gain
30
10 Relaxation Delay
1.0000
11 Pulse Width
9.1500
12 Acquisition Time
1.3107
13 Acquisition Date
2025-09-16T22:54:34
14 Spectrometer Frequency
100.57
15 Spectral Width
25000.0
16 Lowest Frequency
-1425.8
17 Acquired Size
32768
18 Spectral Size
65536
f1 (ppm)

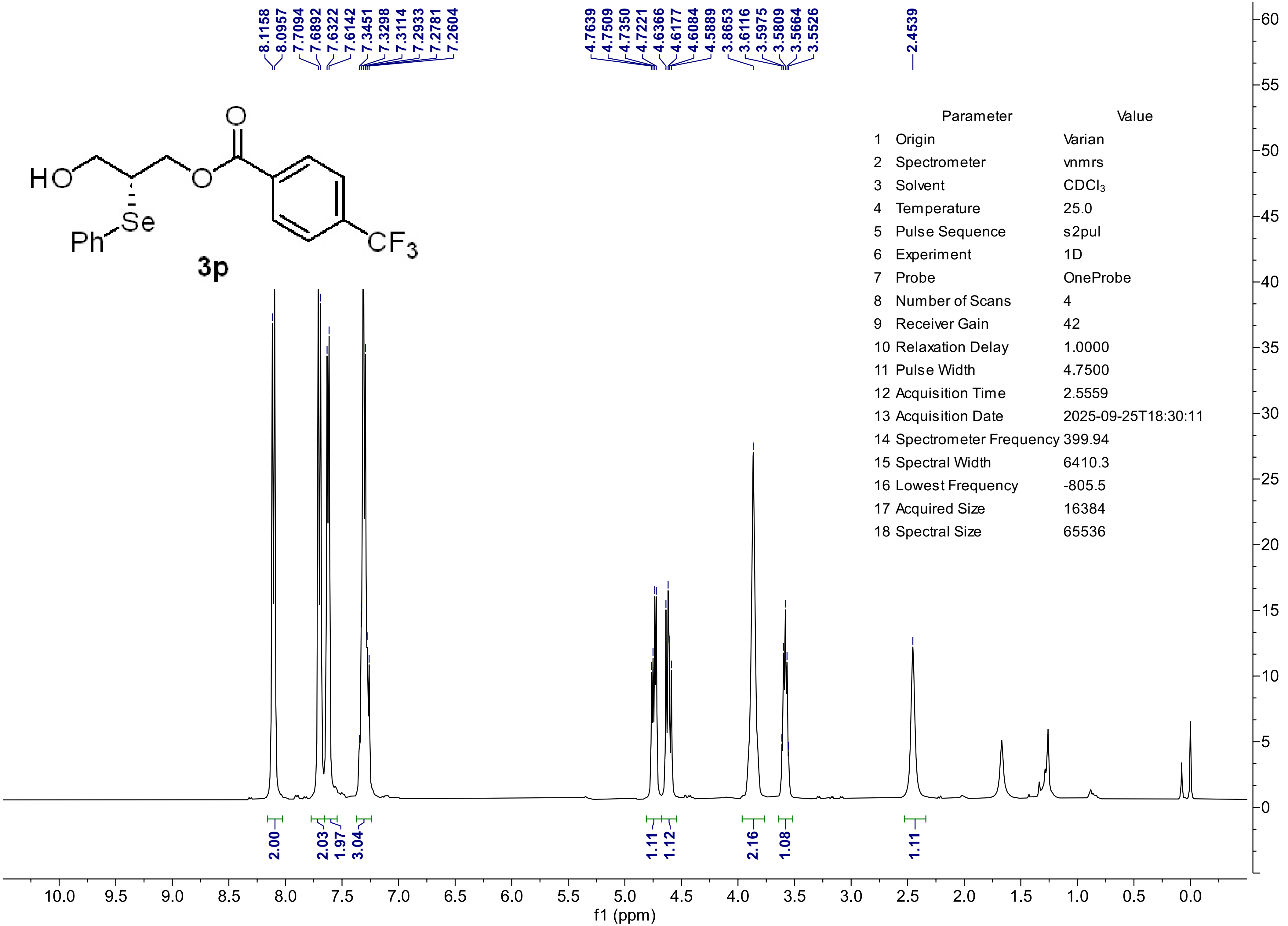

	Parameter	Value
1	Origin	Varian
2	Spectrometer	vnmrs
3	Solvent	$CDCl_3$
4	Temperature	25.0
5	Pulse Sequence	s2pul
6	Experiment	1D
7	Probe	OneProbe
8	Number of Scans	4
9	Receiver Gain	42
10	Relaxation Delay	1.0000
11	Pulse Width	4.7500
12	Acquisition Time	2.5559
13	Acquisition Date	2025-09-25T18:30:11
14	Spectrometer Frequency	399.94
15	Spectral Width	6410.3
16	Lowest Frequency	-805.5
17	Acquired Size	16384
18	Spectral Size	65536

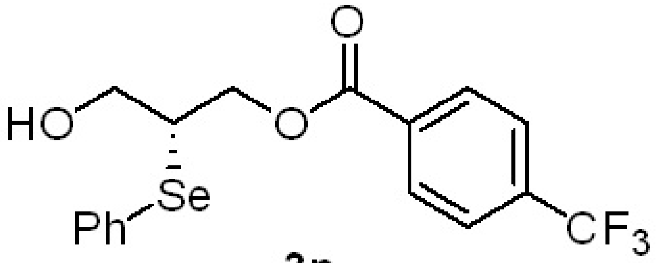

3p

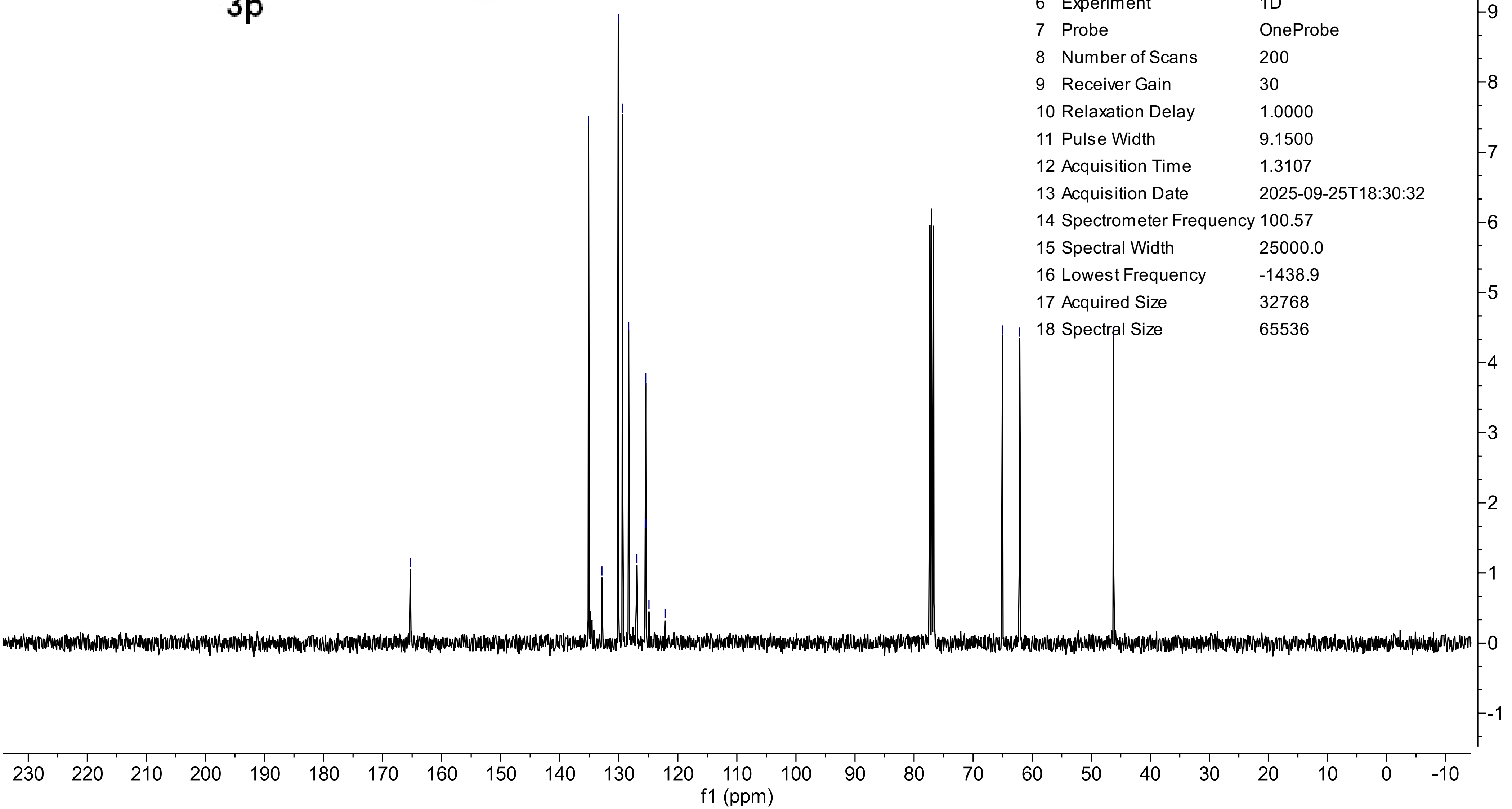

	Parameter	Value
1	Origin	Varian
2	Spectrometer	vnmrs
3	Solvent	$CDCl_3$
4	Temperature	25.0
5	Pulse Sequence	s2pul
6	Experiment	1D
7	Probe	OneProbe
8	Number of Scans	200
9	Receiver Gain	30
10	Relaxation Delay	1.0000
11	Pulse Width	9.1500
12	Acquisition Time	1.3107
13	Acquisition Date	2025-09-25T18:30:32
14	Spectrometer Frequency	100.57
15	Spectral Width	25000.0
16	Lowest Frequency	-1438.9
17	Acquired Size	32768
18	Spectral Size	65536

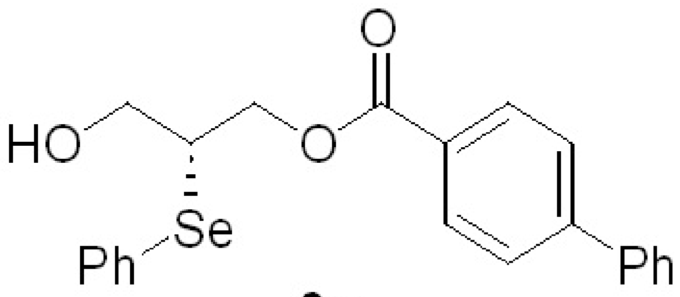

3q

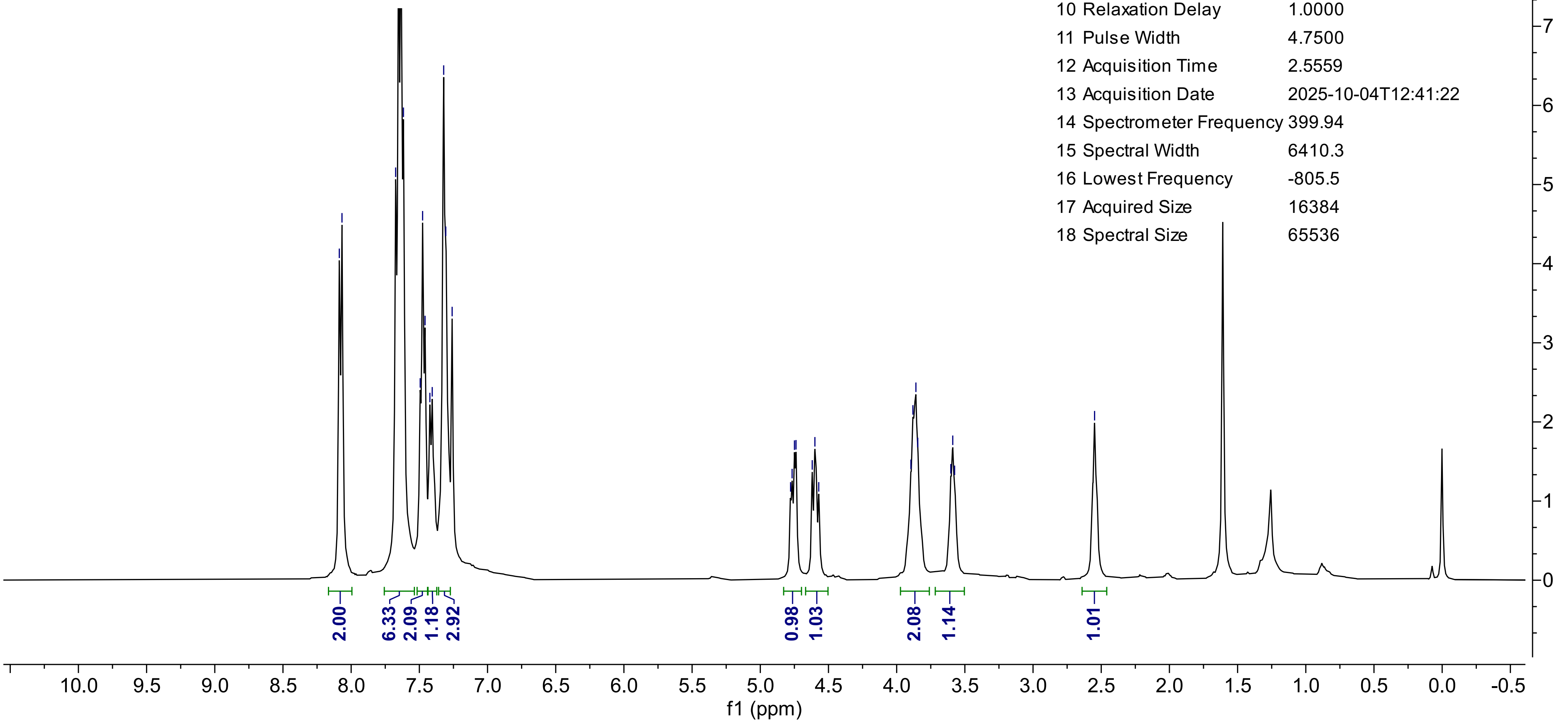

	Parameter	Value
1	Origin	Varian
2	Spectrometer	vnmrs
3	Solvent	$CDCl_3$
4	Temperature	25.0
5	Pulse Sequence	s2pul
6	Experiment	1D
7	Probe	OneProbe
8	Number of Scans	4
9	Receiver Gain	30
10	Relaxation Delay	1.0000
11	Pulse Width	4.7500
12	Acquisition Time	2.5559
13	Acquisition Date	2025-10-04T12:41:22
14	Spectrometer Frequency	399.94
15	Spectral Width	6410.3
16	Lowest Frequency	-805.5
17	Acquired Size	16384
18	Spectral Size	65536

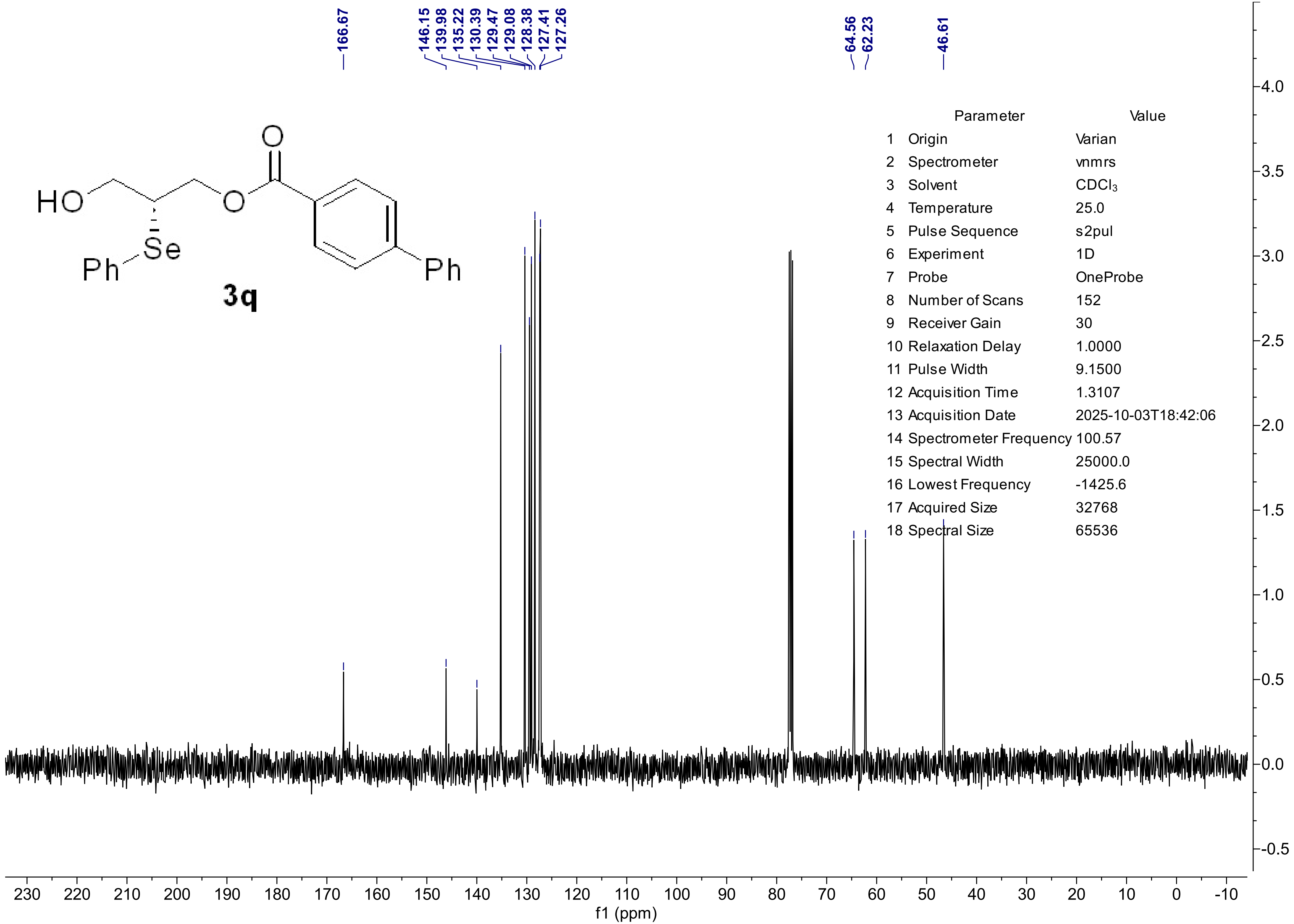

3q

	Parameter	Value
1	Origin	Varian
2	Spectrometer	vnmrs
3	Solvent	$CDCl_3$
4	Temperature	25.0
5	Pulse Sequence	s2pul
6	Experiment	1D
7	Probe	OneProbe
8	Number of Scans	152
9	Receiver Gain	30
10	Relaxation Delay	1.0000
11	Pulse Width	9.1500
12	Acquisition Time	1.3107
13	Acquisition Date	2025-10-03T18:42:06
14	Spectrometer Frequency	100.57
15	Spectral Width	25000.0
16	Lowest Frequency	-1425.6
17	Acquired Size	32768
18	Spectral Size	65536

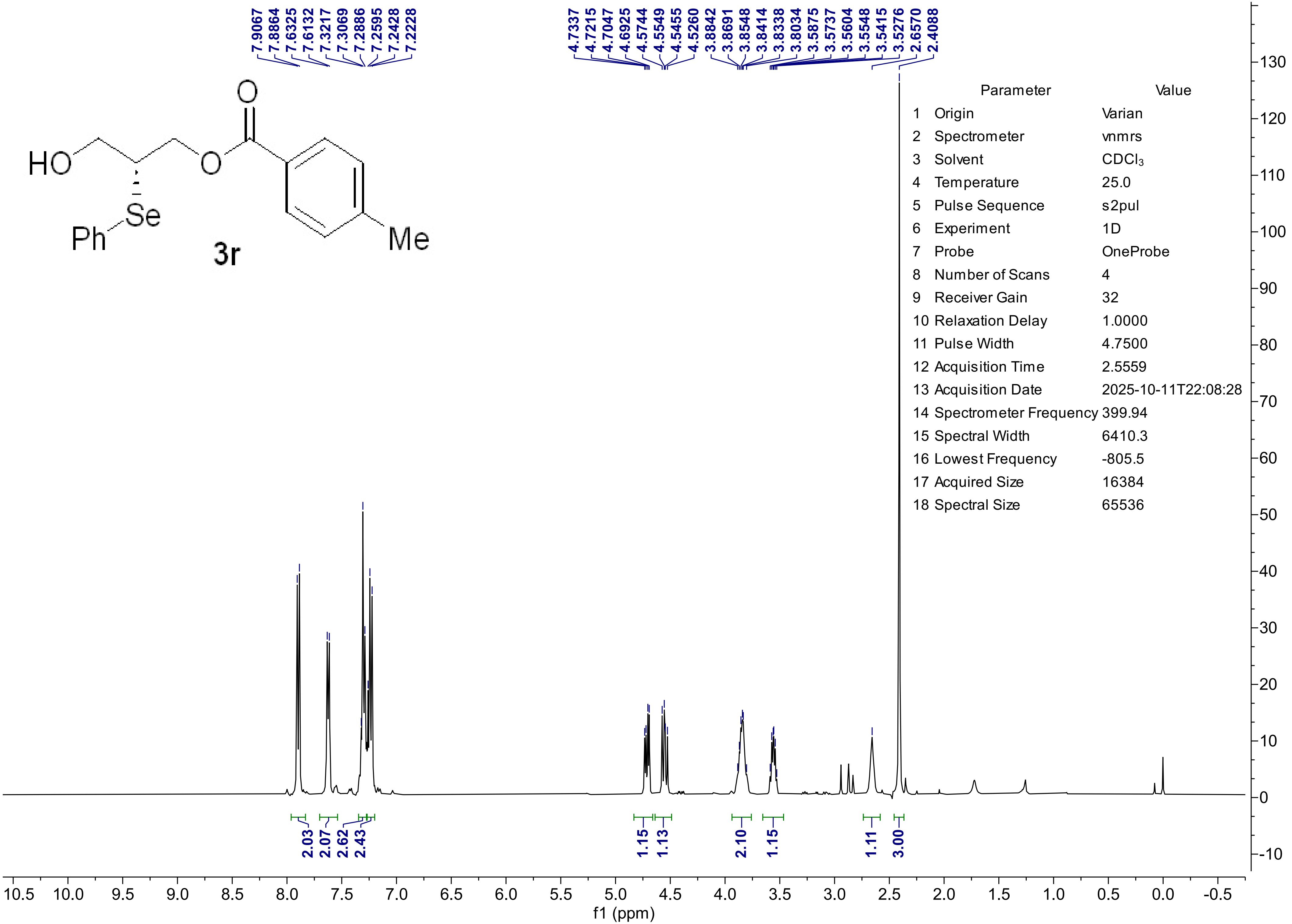

	Parameter	Value
1	Origin	Varian
2	Spectrometer	vnmrs
3	Solvent	$CDCl_3$
4	Temperature	25.0
5	Pulse Sequence	s2pul
6	Experiment	1D
7	Probe	OneProbe
8	Number of Scans	4
9	Receiver Gain	32
10	Relaxation Delay	1.0000
11	Pulse Width	4.7500
12	Acquisition Time	2.5559
13	Acquisition Date	2025-10-11T22:08:28
14	Spectrometer Frequency	399.94
15	Spectral Width	6410.3
16	Lowest Frequency	-805.5
17	Acquired Size	16384
18	Spectral Size	65536

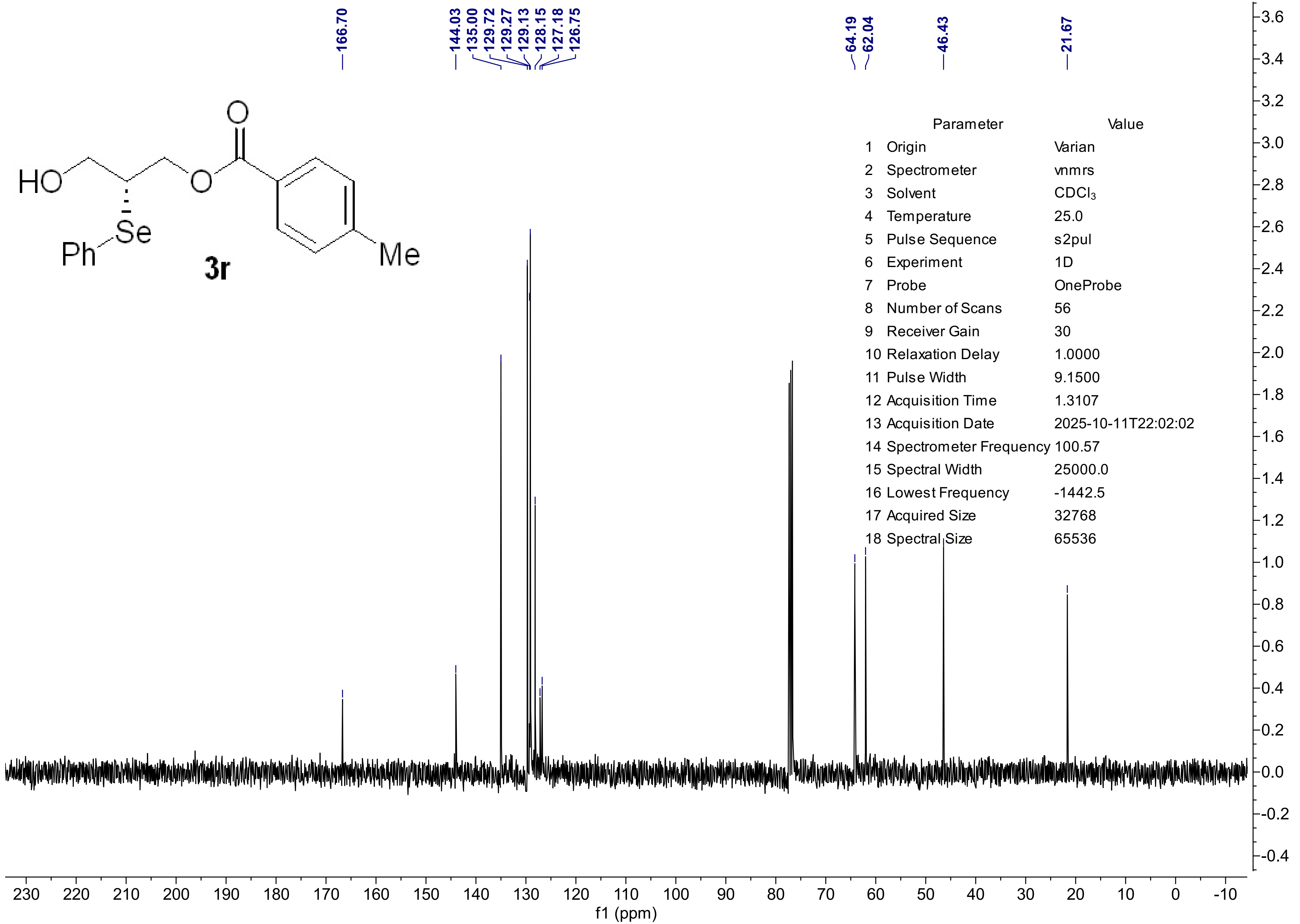
166.70
144.03
135.00
129.72
129.27
129.13
128.15
127.18
126.75
64.19
62.04
46.43
21.67
HO
Se
Ph
O
O
Me
3r
Parameter Value
1 Origin Varian
2 Spectrometer vnmrs
3 Solvent CDCl3
4 Temperature 25.0
5 Pulse Sequence s2pul
6 Experiment 1D
7 Probe OneProbe
8 Number of Scans 56
9 Receiver Gain 30
10 Relaxation Delay 1.0000
11 Pulse Width 9.1500
12 Acquisition Time 1.3107
13 Acquisition Date 2025-10-11T22:02:02
14 Spectrometer Frequency 100.57
15 Spectral Width 25000.0
16 Lowest Frequency -1442.5
17 Acquired Size 32768
18 Spectral Size 65536
f1 (ppm)

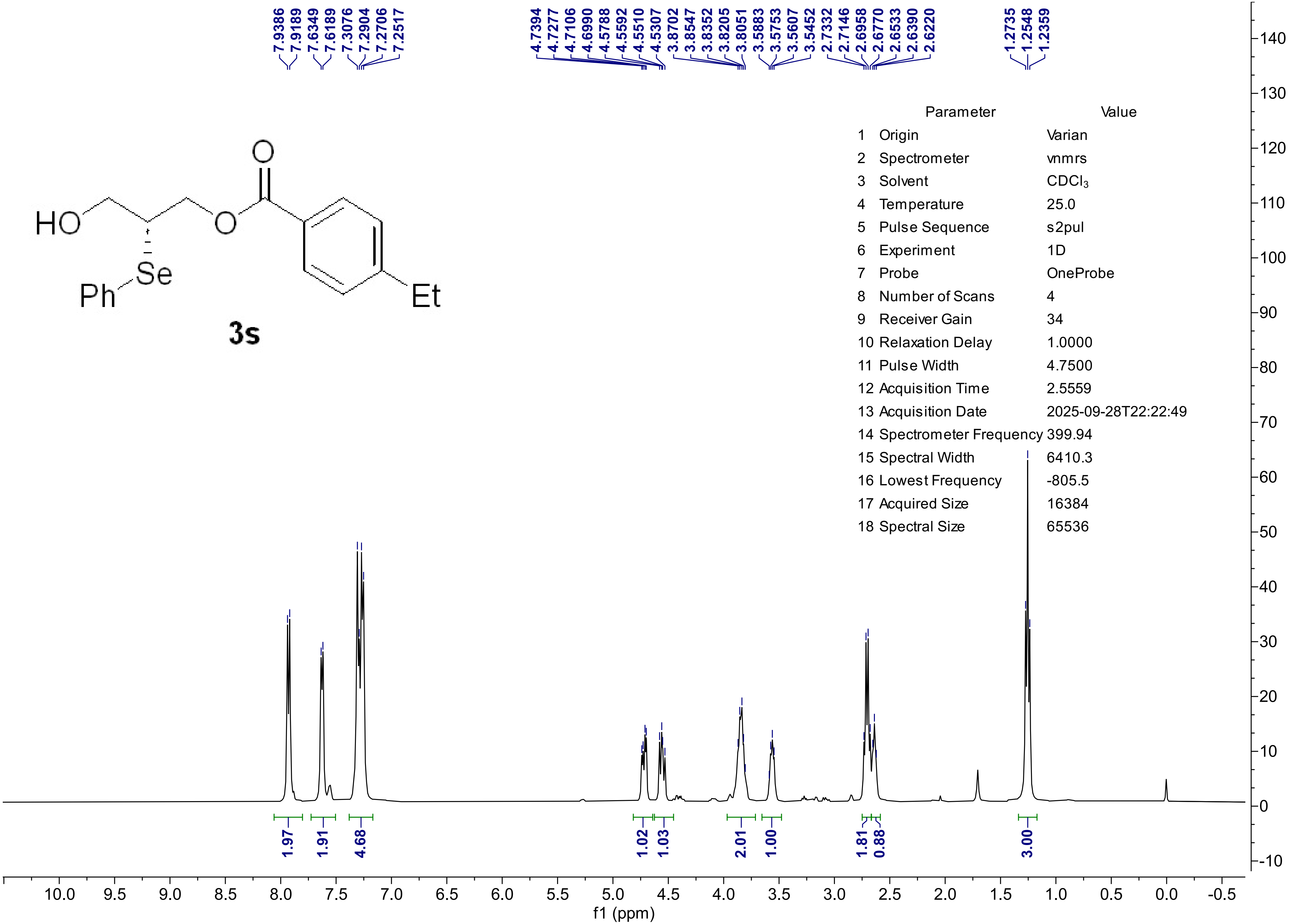

	Parameter	Value
1	Origin	Varian
2	Spectrometer	vnmrs
3	Solvent	$CDCl_3$
4	Temperature	25.0
5	Pulse Sequence	s2pul
6	Experiment	1D
7	Probe	OneProbe
8	Number of Scans	4
9	Receiver Gain	34
10	Relaxation Delay	1.0000
11	Pulse Width	4.7500
12	Acquisition Time	2.5559
13	Acquisition Date	2025-09-28T22:22:49
14	Spectrometer Frequency	399.94
15	Spectral Width	6410.3
16	Lowest Frequency	-805.5
17	Acquired Size	16384
18	Spectral Size	65536

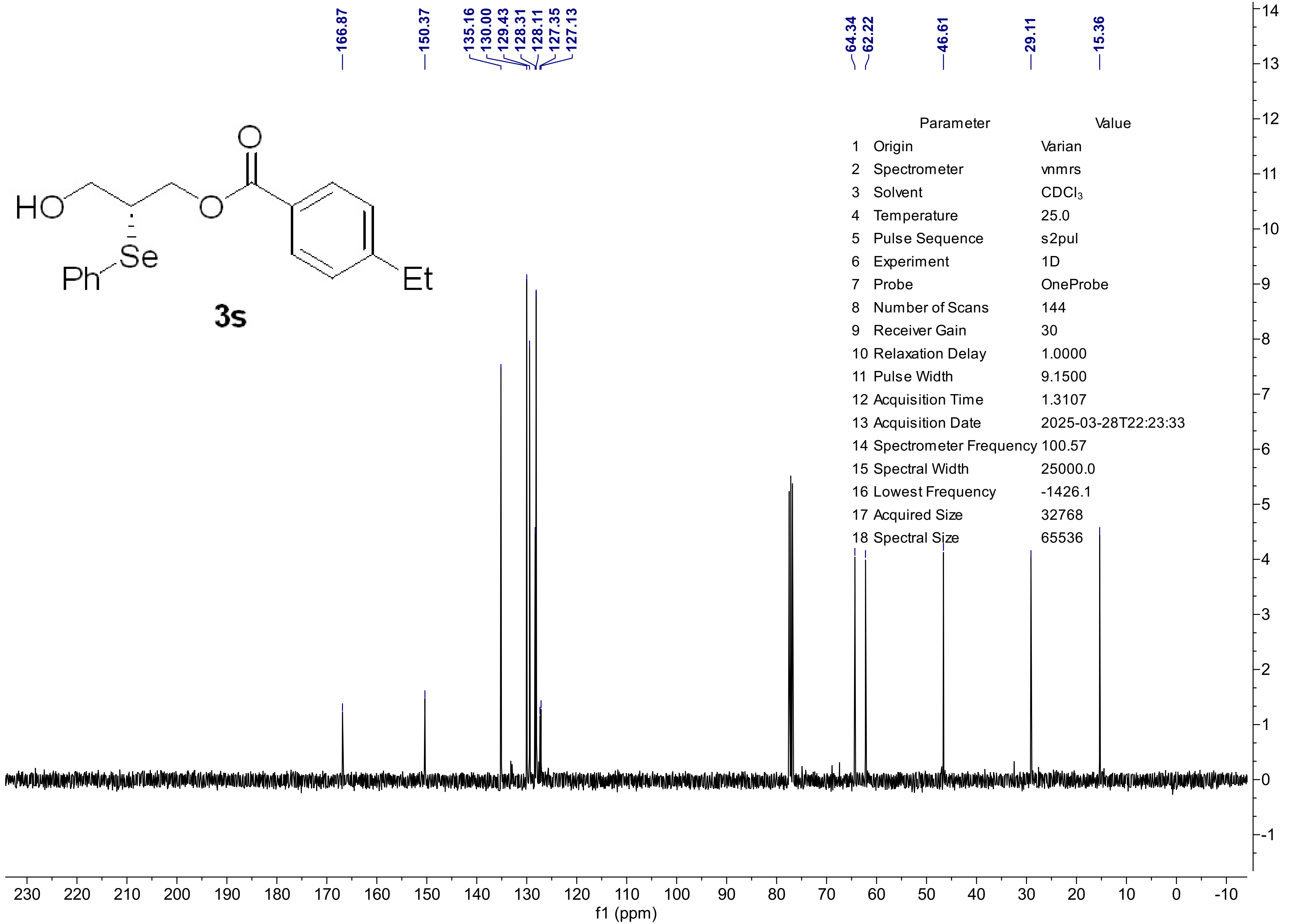
166.87
150.37
135.16
130.00
129.43
128.31
128.11
127.35
127.13
64.34
62.22
46.61
29.11
15.36
HO
O
O
Se
Ph
Et
3s
Parameter Value
1 Origin Varian
2 Spectrometer vnmrs
3 Solvent CDCl3
4 Temperature 25.0
5 Pulse Sequence s2pul
6 Experiment 1D
7 Probe OneProbe
8 Number of Scans 144
9 Receiver Gain 30
10 Relaxation Delay 1.0000
11 Pulse Width 9.1500
12 Acquisition Time 1.3107
13 Acquisition Date 2025-03-28T22:23:33
14 Spectrometer Frequency 100.57
15 Spectral Width 25000.0
16 Lowest Frequency -1426.1
17 Acquired Size 32768
18 Spectral Size 65536
f1 (ppm)

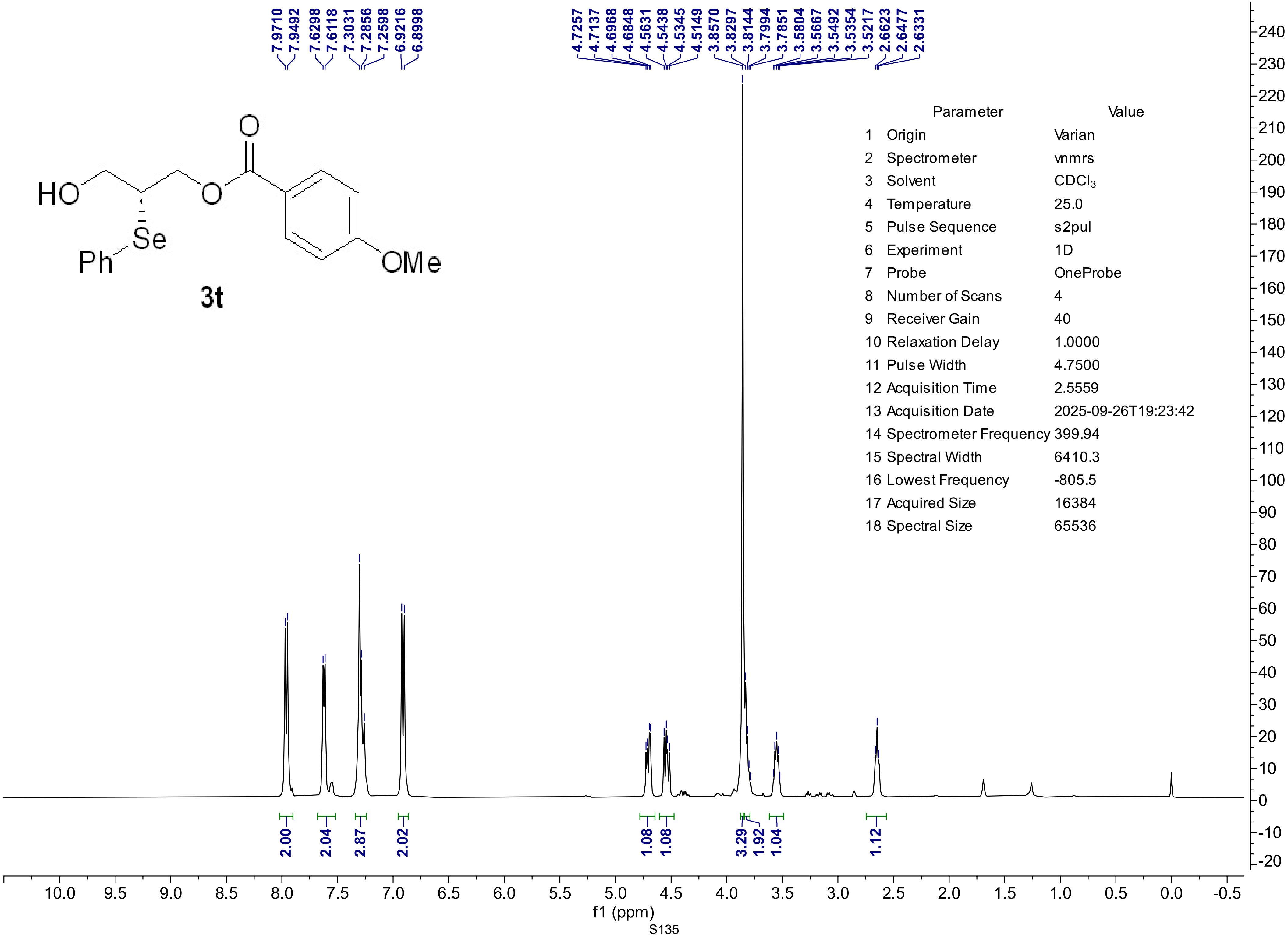

	Parameter	Value
1	Origin	Varian
2	Spectrometer	vnmrs
3	Solvent	$CDCl_3$
4	Temperature	25.0
5	Pulse Sequence	s2pul
6	Experiment	1D
7	Probe	OneProbe
8	Number of Scans	4
9	Receiver Gain	40
10	Relaxation Delay	1.0000
11	Pulse Width	4.7500
12	Acquisition Time	2.5559
13	Acquisition Date	2025-09-26T19:23:42
14	Spectrometer Frequency	399.94
15	Spectral Width	6410.3
16	Lowest Frequency	-805.5
17	Acquired Size	16384
18	Spectral Size	65536

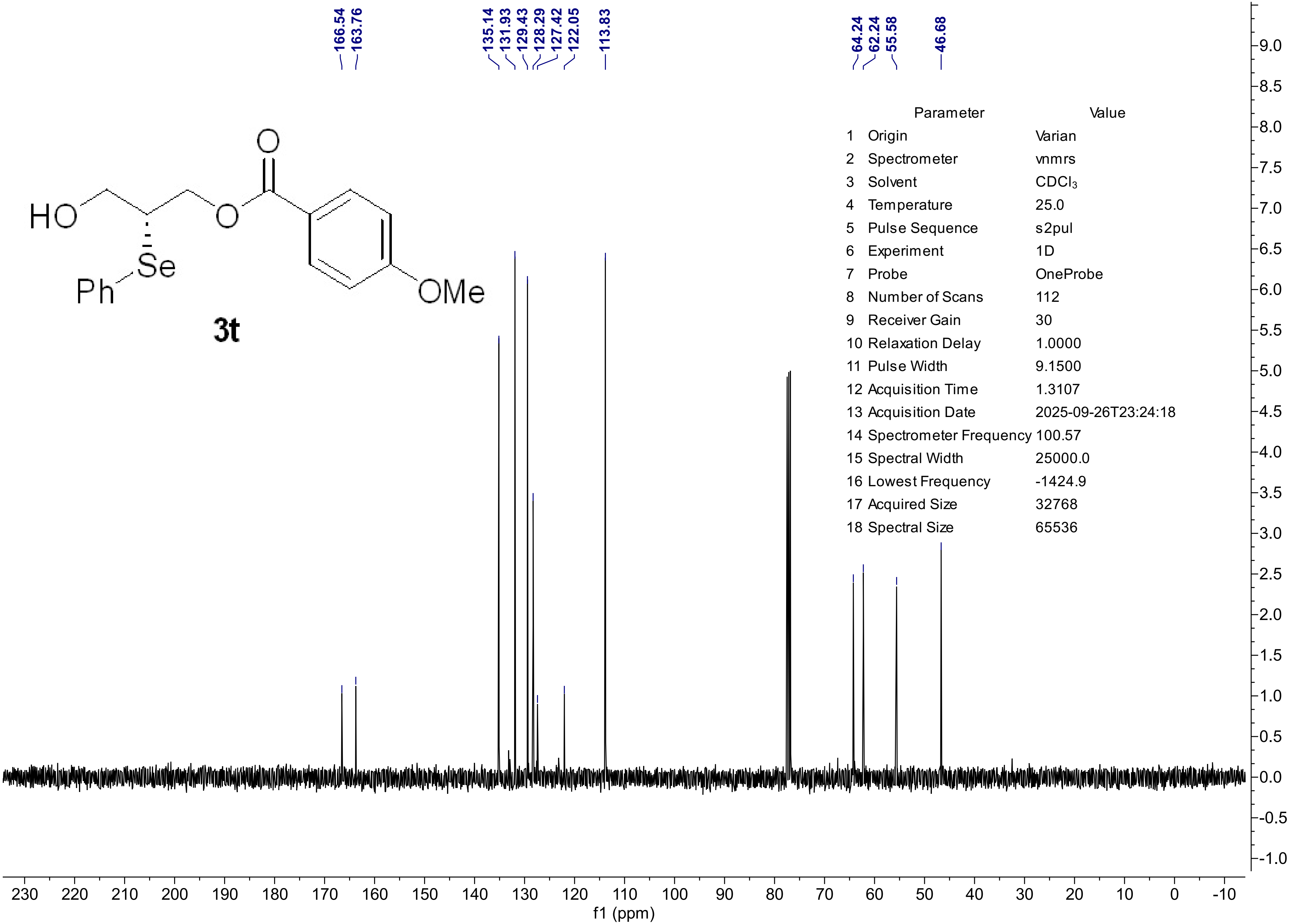

	Parameter	Value
1	Origin	Varian
2	Spectrometer	vnmrs
3	Solvent	$CDCl_3$
4	Temperature	25.0
5	Pulse Sequence	s2pul
6	Experiment	1D
7	Probe	OneProbe
8	Number of Scans	112
9	Receiver Gain	30
10	Relaxation Delay	1.0000
11	Pulse Width	9.1500
12	Acquisition Time	1.3107
13	Acquisition Date	2025-09-26T23:24:18
14	Spectrometer Frequency	100.57
15	Spectral Width	25000.0
16	Lowest Frequency	-1424.9
17	Acquired Size	32768
18	Spectral Size	65536

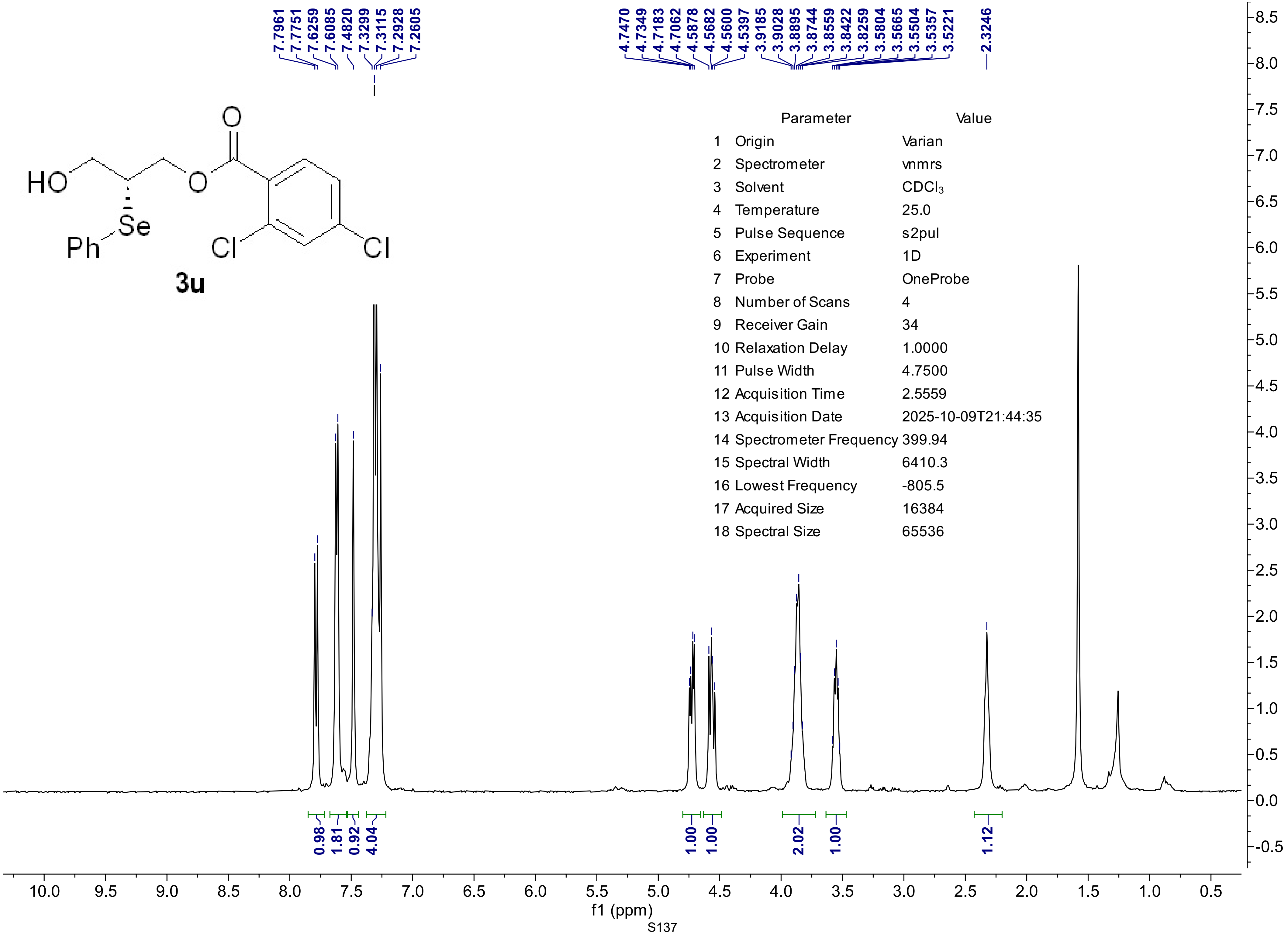

	Parameter	Value
1	Origin	Varian
2	Spectrometer	vnmrs
3	Solvent	$CDCl_3$
4	Temperature	25.0
5	Pulse Sequence	s2pul
6	Experiment	1D
7	Probe	OneProbe
8	Number of Scans	4
9	Receiver Gain	34
10	Relaxation Delay	1.0000
11	Pulse Width	4.7500
12	Acquisition Time	2.5559
13	Acquisition Date	2025-10-09T21:44:35
14	Spectrometer Frequency	399.94
15	Spectral Width	6410.3
16	Lowest Frequency	-805.5
17	Acquired Size	16384
18	Spectral Size	65536

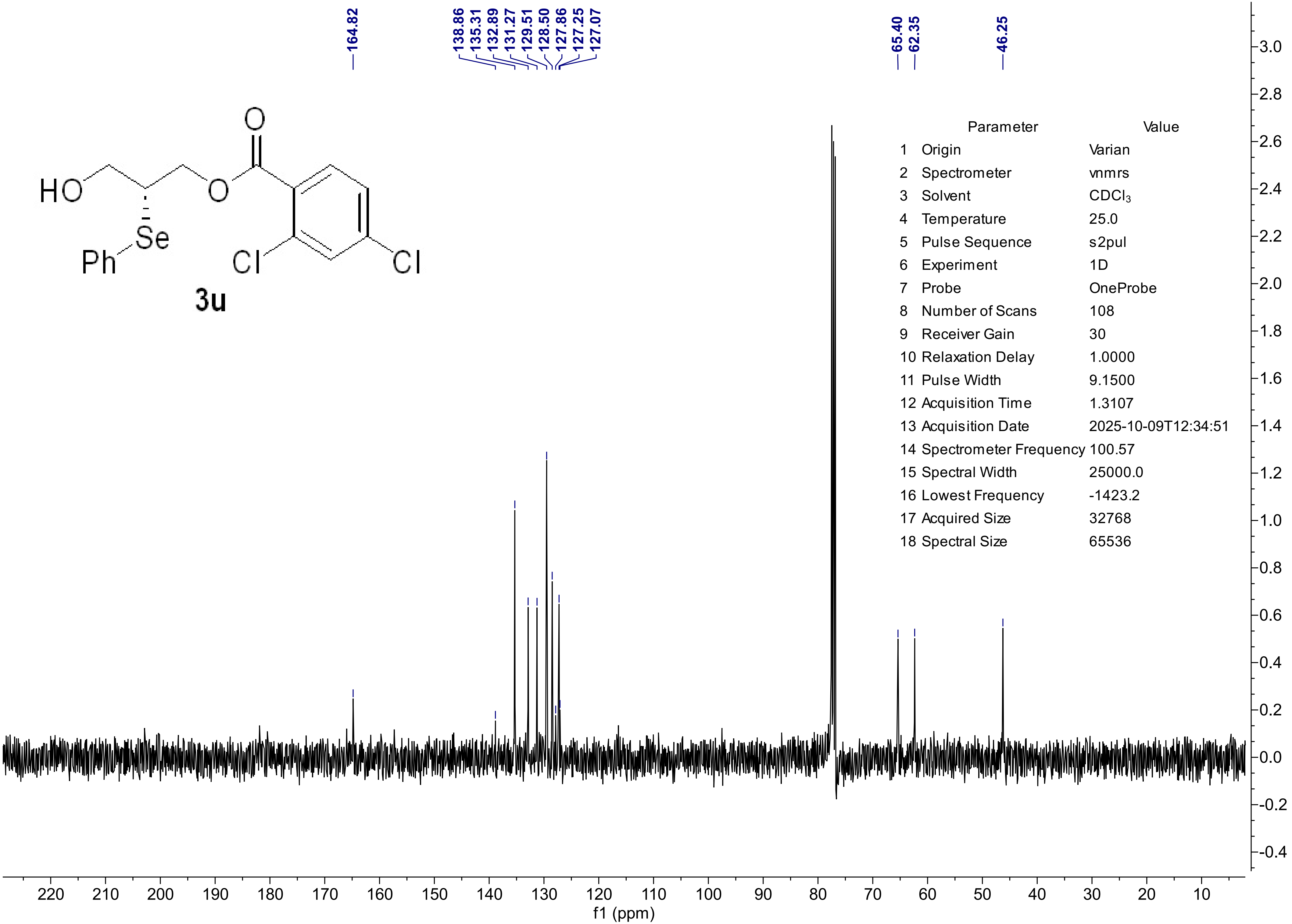

164.82
138.86
135.31
132.89
131.27
129.51
128.50
127.86
127.25
127.07
65.40
62.35
46.25
HO
Se
Ph
O
O
Cl
Cl
3u
Parameter Value
1 Origin Varian
2 Spectrometer vnmrs
3 Solvent CDCl3
4 Temperature 25.0
5 Pulse Sequence s2pul
6 Experiment 1D
7 Probe OneProbe
8 Number of Scans 108
9 Receiver Gain 30
10 Relaxation Delay 1.0000
11 Pulse Width 9.1500
12 Acquisition Time 1.3107
13 Acquisition Date 2025-10-09T12:34:51
14 Spectrometer Frequency 100.57
15 Spectral Width 25000.0
16 Lowest Frequency -1423.2
17 Acquired Size 32768
18 Spectral Size 65536
f1 (ppm)

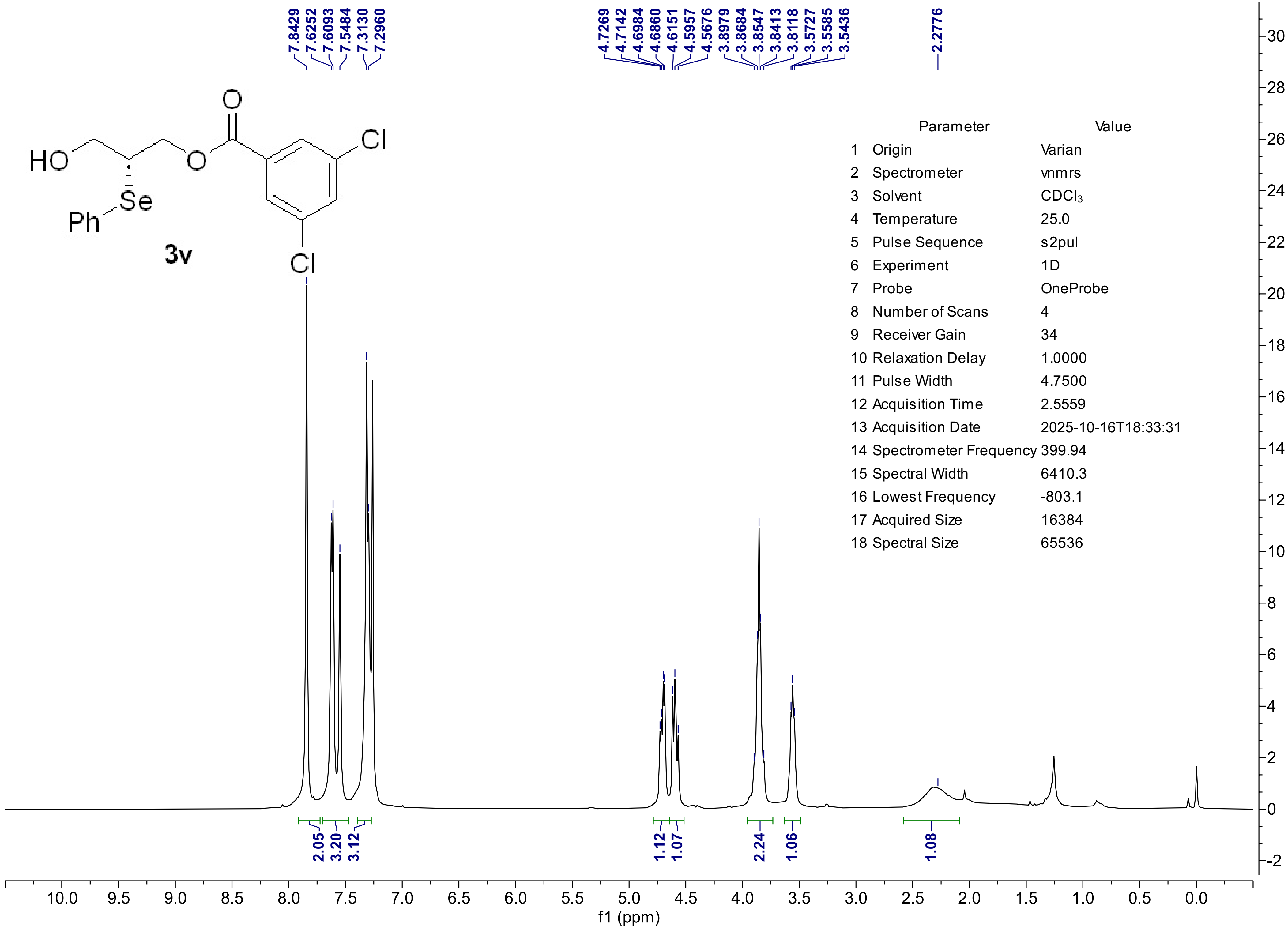

	Parameter	Value
1	Origin	Varian
2	Spectrometer	vnmrs
3	Solvent	$CDCl_3$
4	Temperature	25.0
5	Pulse Sequence	s2pul
6	Experiment	1D
7	Probe	OneProbe
8	Number of Scans	4
9	Receiver Gain	34
10	Relaxation Delay	1.0000
11	Pulse Width	4.7500
12	Acquisition Time	2.5559
13	Acquisition Date	2025-10-16T18:33:31
14	Spectrometer Frequency	399.94
15	Spectral Width	6410.3
16	Lowest Frequency	-803.1
17	Acquired Size	16384
18	Spectral Size	65536

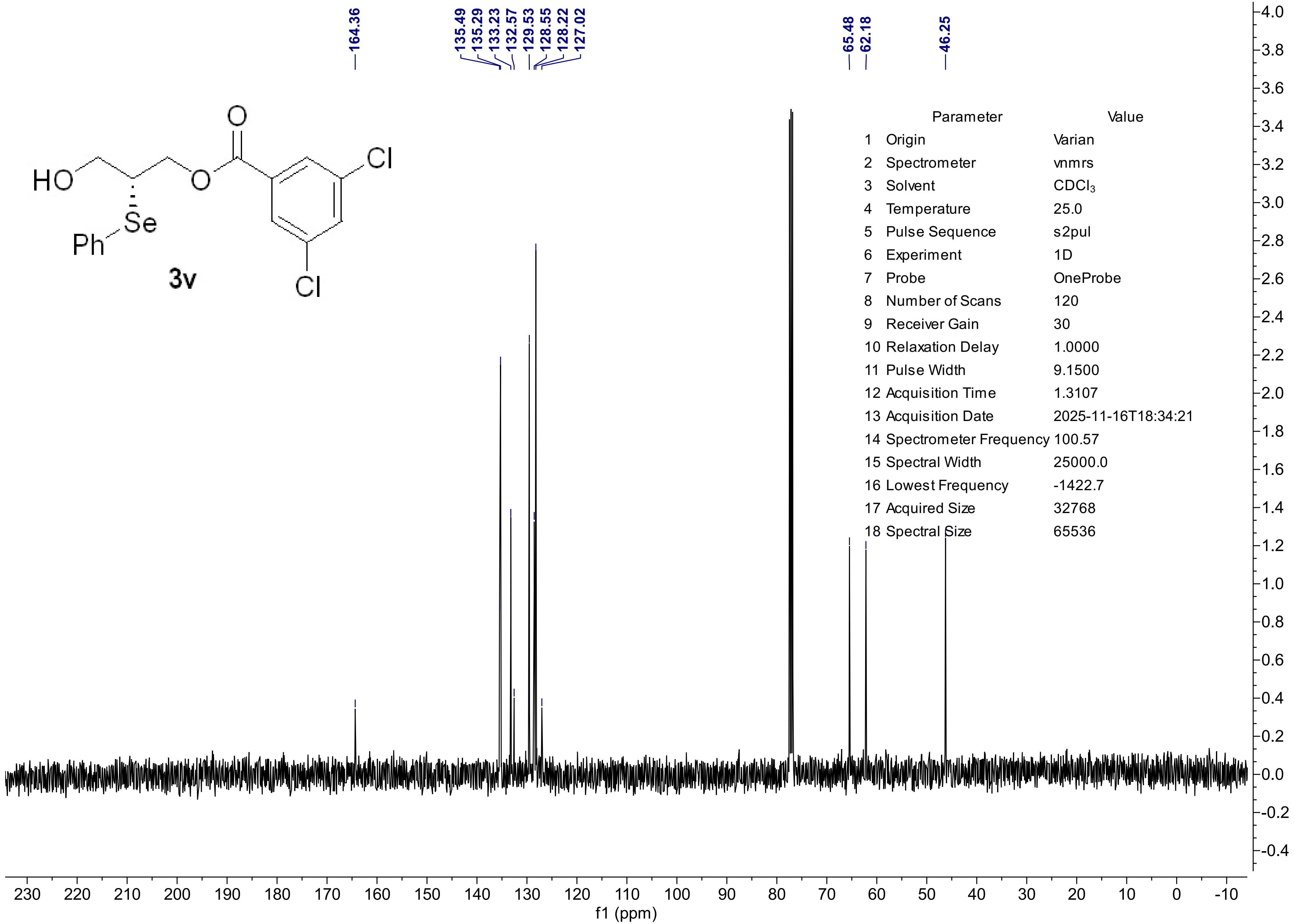
164.36
135.49
135.29
133.23
132.57
129.53
128.55
128.22
127.02
65.48
62.18
46.25
HO
O
O
Cl
Cl
Se
Ph
3v
Parameter
Value
1 Origin Varian
2 Spectrometer vnmrs
3 Solvent CDCl3
4 Temperature 25.0
5 Pulse Sequence s2pul
6 Experiment 1D
7 Probe OneProbe
8 Number of Scans 120
9 Receiver Gain 30
10 Relaxation Delay 1.0000
11 Pulse Width 9.1500
12 Acquisition Time 1.3107
13 Acquisition Date 2025-11-16T18:34:21
14 Spectrometer Frequency 100.57
15 Spectral Width 25000.0
16 Lowest Frequency -1422.7
17 Acquired Size 32768
18 Spectral Size 65536
f1 (ppm)

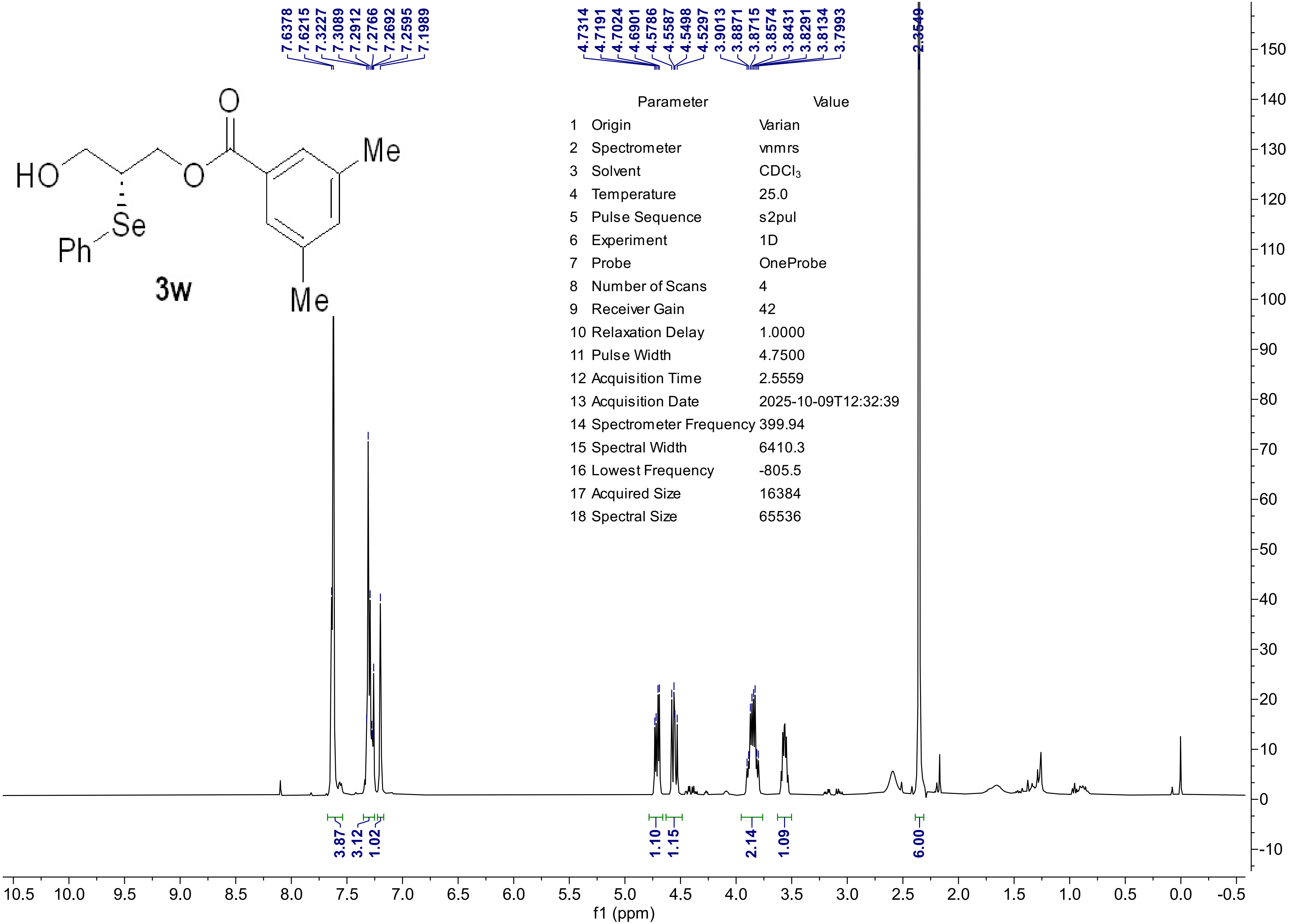

	Parameter	Value
1	Origin	Varian
2	Spectrometer	vnmrs
3	Solvent	$CDCl_3$
4	Temperature	25.0
5	Pulse Sequence	s2pul
6	Experiment	1D
7	Probe	OneProbe
8	Number of Scans	4
9	Receiver Gain	42
10	Relaxation Delay	1.0000
11	Pulse Width	4.7500
12	Acquisition Time	2.5559
13	Acquisition Date	2025-10-09T12:32:39
14	Spectrometer Frequency	399.94
15	Spectral Width	6410.3
16	Lowest Frequency	-805.5
17	Acquired Size	16384
18	Spectral Size	65536

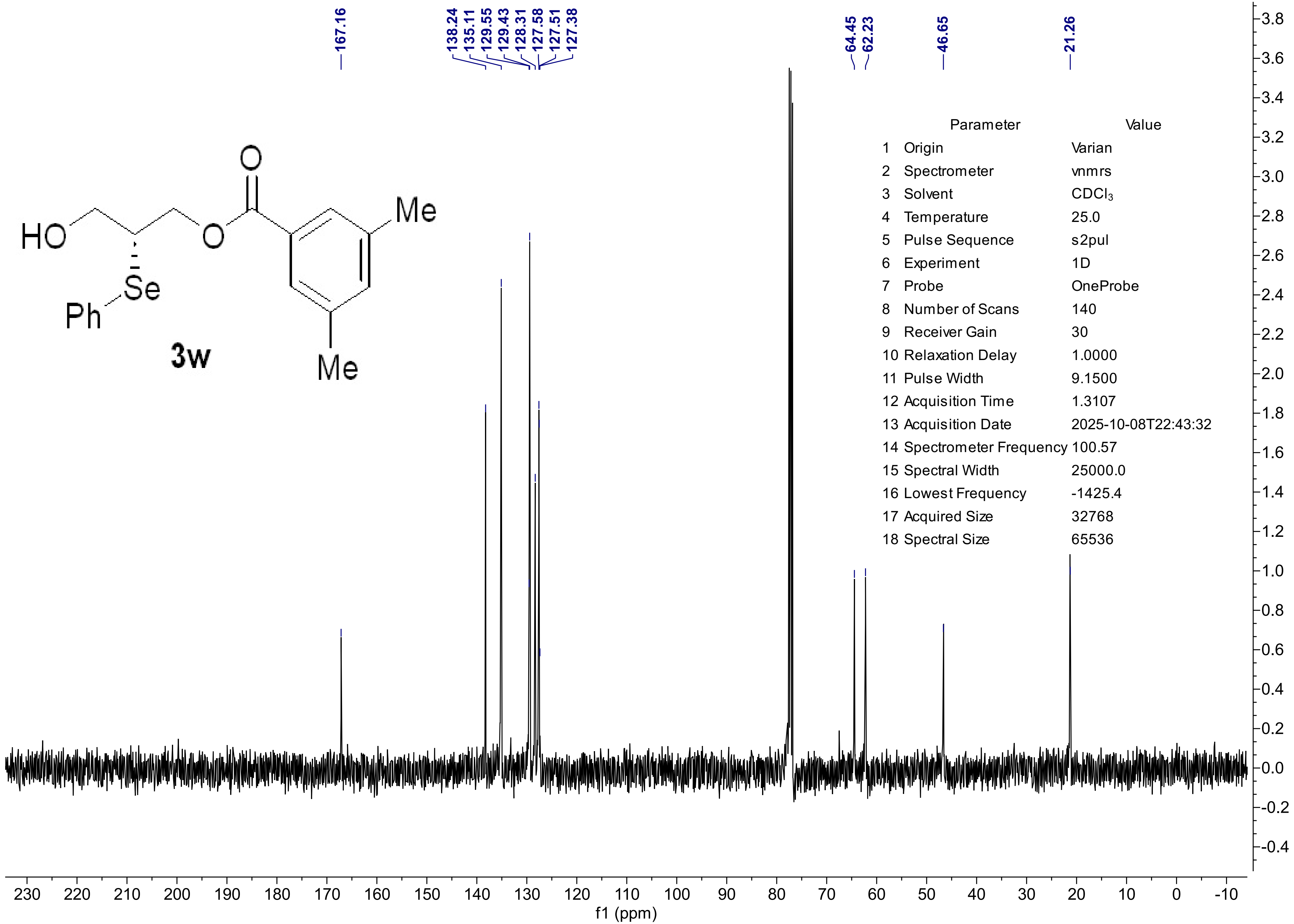

167.16
138.24
135.11
129.55
129.43
128.31
127.58
127.51
127.38
64.45
62.23
46.65
21.26
HO
O
O
Me
Me
Se
Ph
3w
Parameter Value
1 Origin Varian
2 Spectrometer vnmrs
3 Solvent CDCl3
4 Temperature 25.0
5 Pulse Sequence s2pul
6 Experiment 1D
7 Probe OneProbe
8 Number of Scans 140
9 Receiver Gain 30
10 Relaxation Delay 1.0000
11 Pulse Width 9.1500
12 Acquisition Time 1.3107
13 Acquisition Date 2025-10-08T22:43:32
14 Spectrometer Frequency 100.57
15 Spectral Width 25000.0
16 Lowest Frequency -1425.4
17 Acquired Size 32768
18 Spectral Size 65536
f1 (ppm)

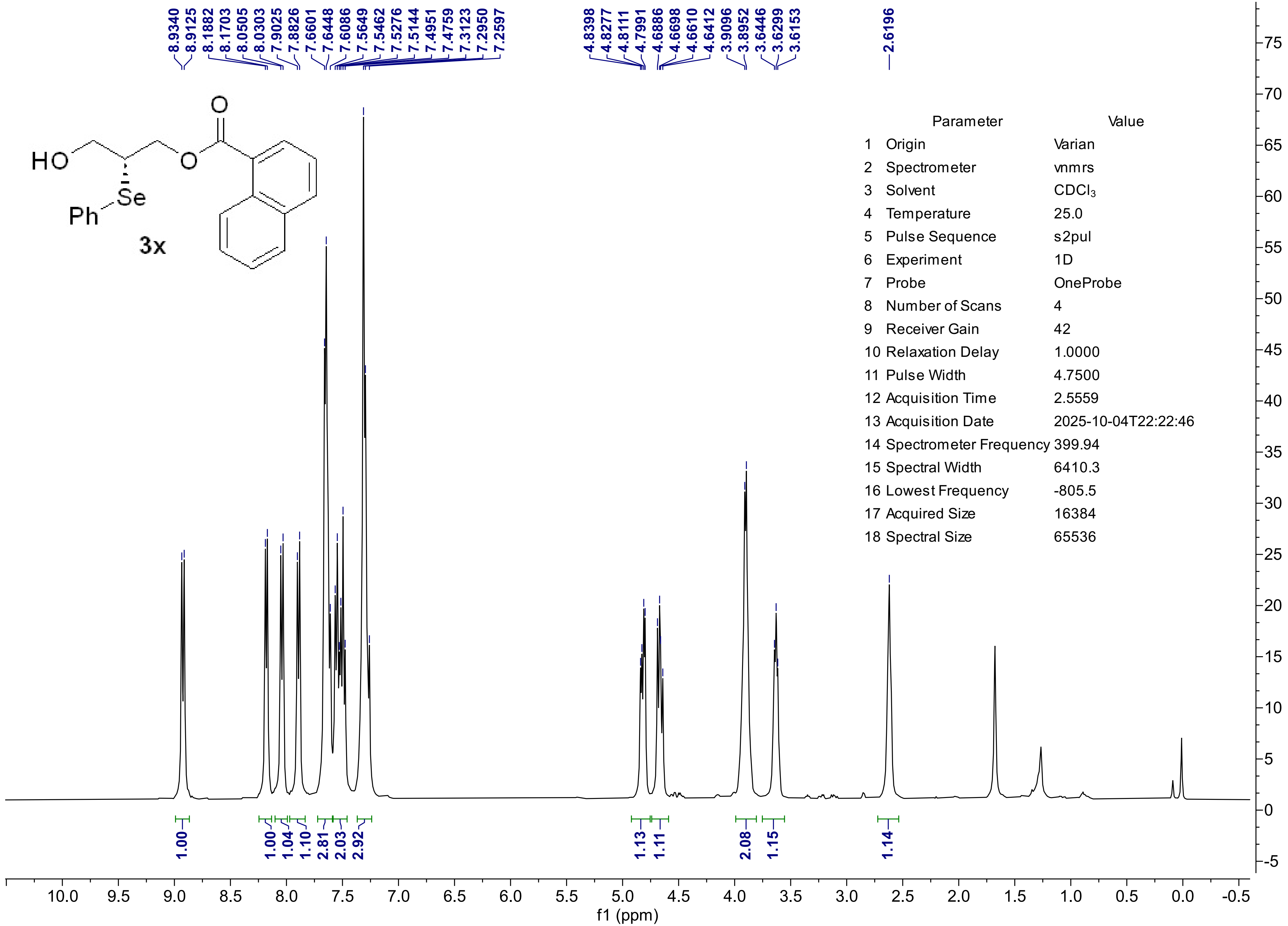
8.9340
8.9125
8.1882
8.1703
8.0505
8.0303
7.9025
7.8826
7.6601
7.6448
7.6086
7.5649
7.5462
7.5276
7.5144
7.4951
7.4759
7.3123
7.2950
7.2597
4.8398
4.8277
4.8111
4.7991
4.6886
4.6698
4.6610
4.6412
3.9096
3.8952
3.6446
3.6299
3.6153
2.6196
O
HO
O
Se
Ph
3x
Parameter Value
1 Origin Varian
2 Spectrometer vnmrs
3 Solvent $CDCl_3$
4 Temperature 25.0
5 Pulse Sequence s2pul
6 Experiment 1D
7 Probe OneProbe
8 Number of Scans 4
9 Receiver Gain 42
10 Relaxation Delay 1.0000
11 Pulse Width 4.7500
12 Acquisition Time 2.5559
13 Acquisition Date 2025-10-04T22:22:46
14 Spectrometer Frequency 399.94
15 Spectral Width 6410.3
16 Lowest Frequency -805.5
17 Acquired Size 16384
18 Spectral Size 65536
1.00
1.00
1.04
1.10
2.81
2.03
2.92
1.13
1.11
2.08
1.15
1.14
f1 (ppm)

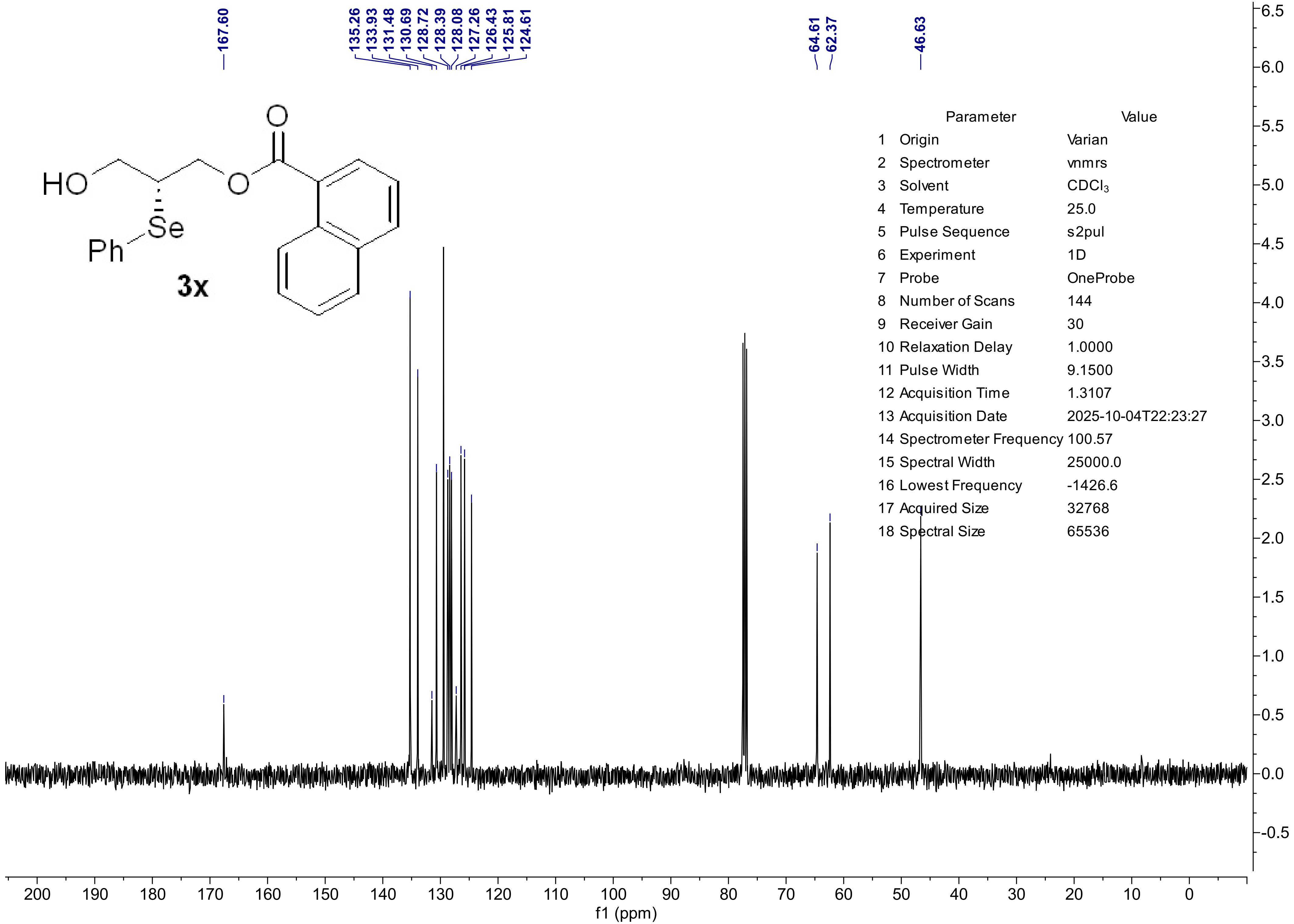

	Parameter	Value
1	Origin	Varian
2	Spectrometer	vnmrs
3	Solvent	$CDCl_3$
4	Temperature	25.0
5	Pulse Sequence	s2pul
6	Experiment	1D
7	Probe	OneProbe
8	Number of Scans	144
9	Receiver Gain	30
10	Relaxation Delay	1.0000
11	Pulse Width	9.1500
12	Acquisition Time	1.3107
13	Acquisition Date	2025-10-04T22:23:27
14	Spectrometer Frequency	100.57
15	Spectral Width	25000.0
16	Lowest Frequency	-1426.6
17	Acquired Size	32768
18	Spectral Size	65536

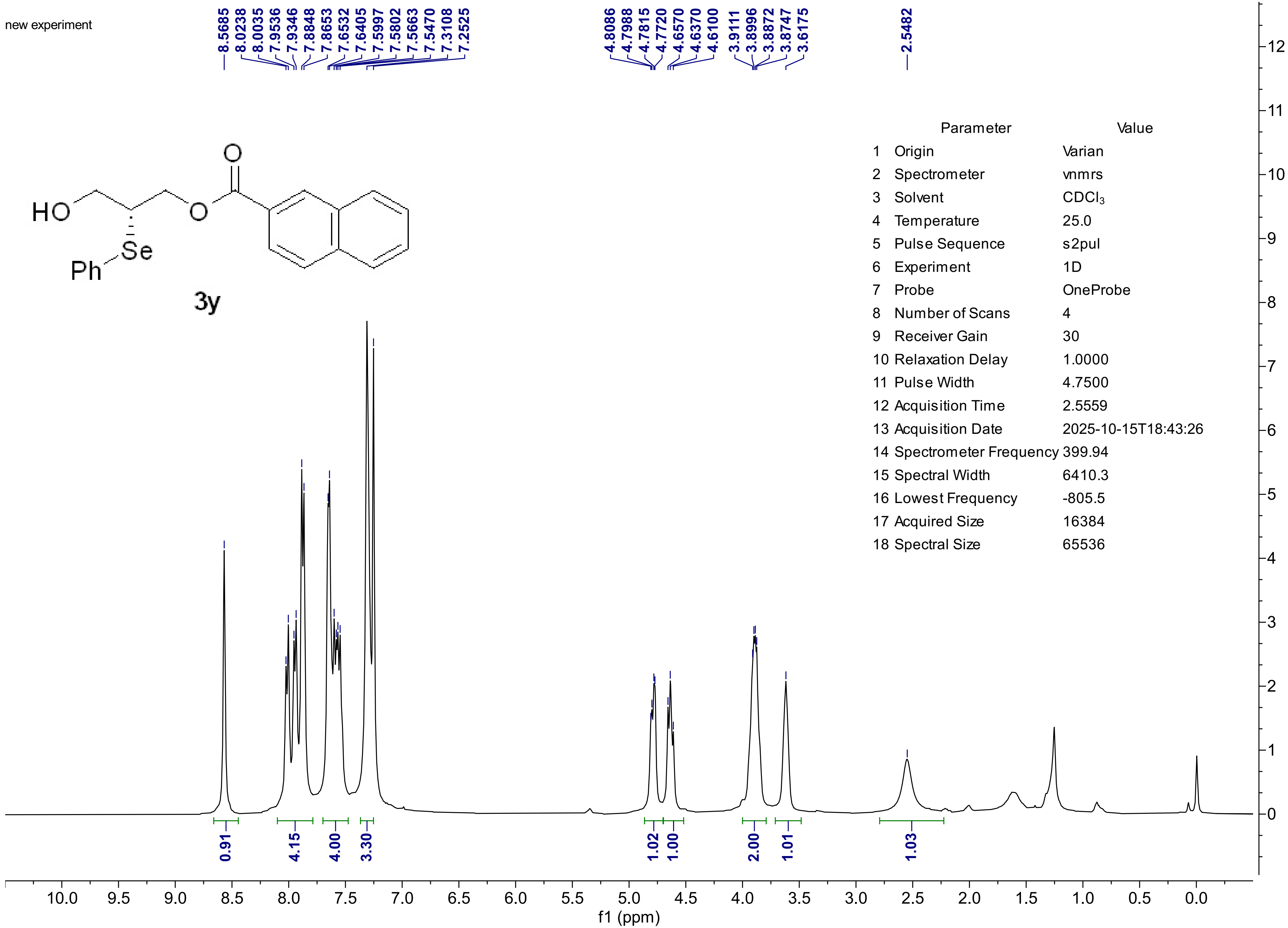

	Parameter	Value
1	Origin	Varian
2	Spectrometer	vnmrs
3	Solvent	$CDCl_3$
4	Temperature	25.0
5	Pulse Sequence	s2pul
6	Experiment	1D
7	Probe	OneProbe
8	Number of Scans	4
9	Receiver Gain	30
10	Relaxation Delay	1.0000
11	Pulse Width	4.7500
12	Acquisition Time	2.5559
13	Acquisition Date	2025-10-15T18:43:26
14	Spectrometer Frequency	399.94
15	Spectral Width	6410.3
16	Lowest Frequency	-805.5
17	Acquired Size	16384
18	Spectral Size	65536

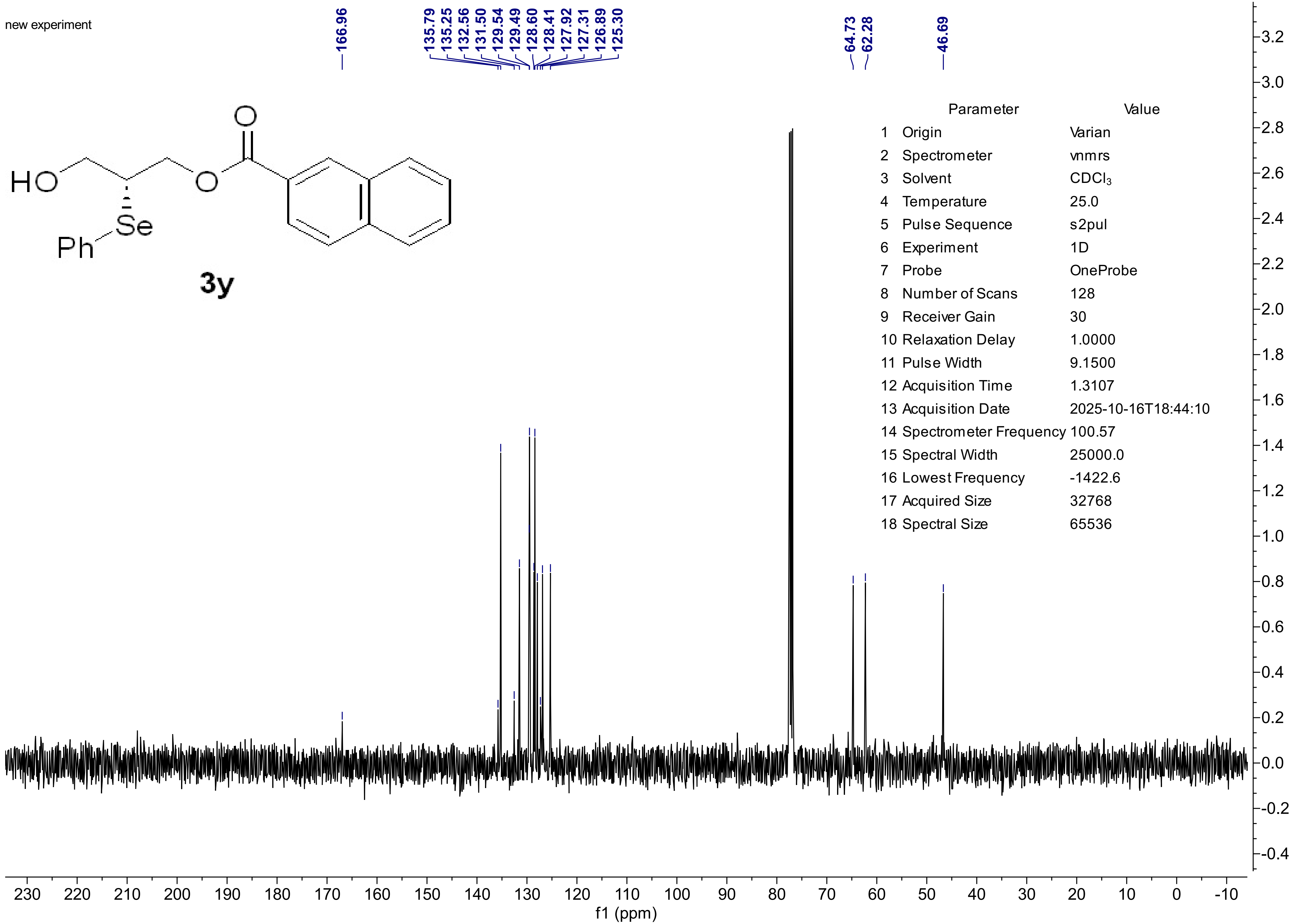

	Parameter	Value
1	Origin	Varian
2	Spectrometer	vnmrs
3	Solvent	$CDCl_3$
4	Temperature	25.0
5	Pulse Sequence	s2pul
6	Experiment	1D
7	Probe	OneProbe
8	Number of Scans	128
9	Receiver Gain	30
10	Relaxation Delay	1.0000
11	Pulse Width	9.1500
12	Acquisition Time	1.3107
13	Acquisition Date	2025-10-16T18:44:10
14	Spectrometer Frequency	100.57
15	Spectral Width	25000.0
16	Lowest Frequency	-1422.6
17	Acquired Size	32768
18	Spectral Size	65536

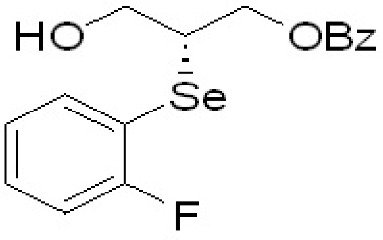

3z

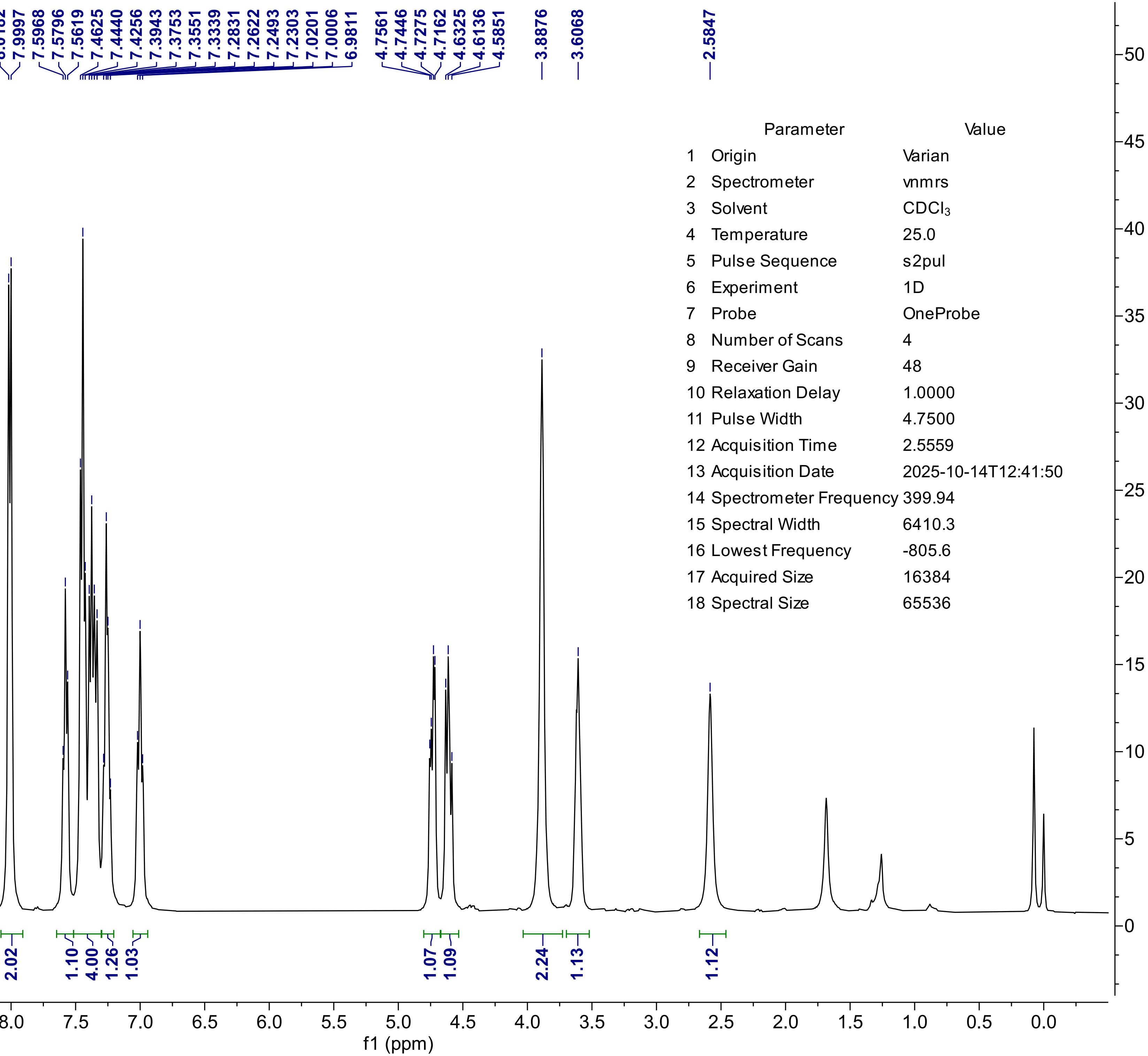

	Parameter	Value
1	Origin	Varian
2	Spectrometer	vnmrs
3	Solvent	$CDCl_3$
4	Temperature	25.0
5	Pulse Sequence	s2pul
6	Experiment	1D
7	Probe	OneProbe
8	Number of Scans	4
9	Receiver Gain	48
10	Relaxation Delay	1.0000
11	Pulse Width	4.7500
12	Acquisition Time	2.5559
13	Acquisition Date	2025-10-14T12:41:50
14	Spectrometer Frequency	399.94
15	Spectral Width	6410.3
16	Lowest Frequency	-805.6
17	Acquired Size	16384
18	Spectral Size	65536

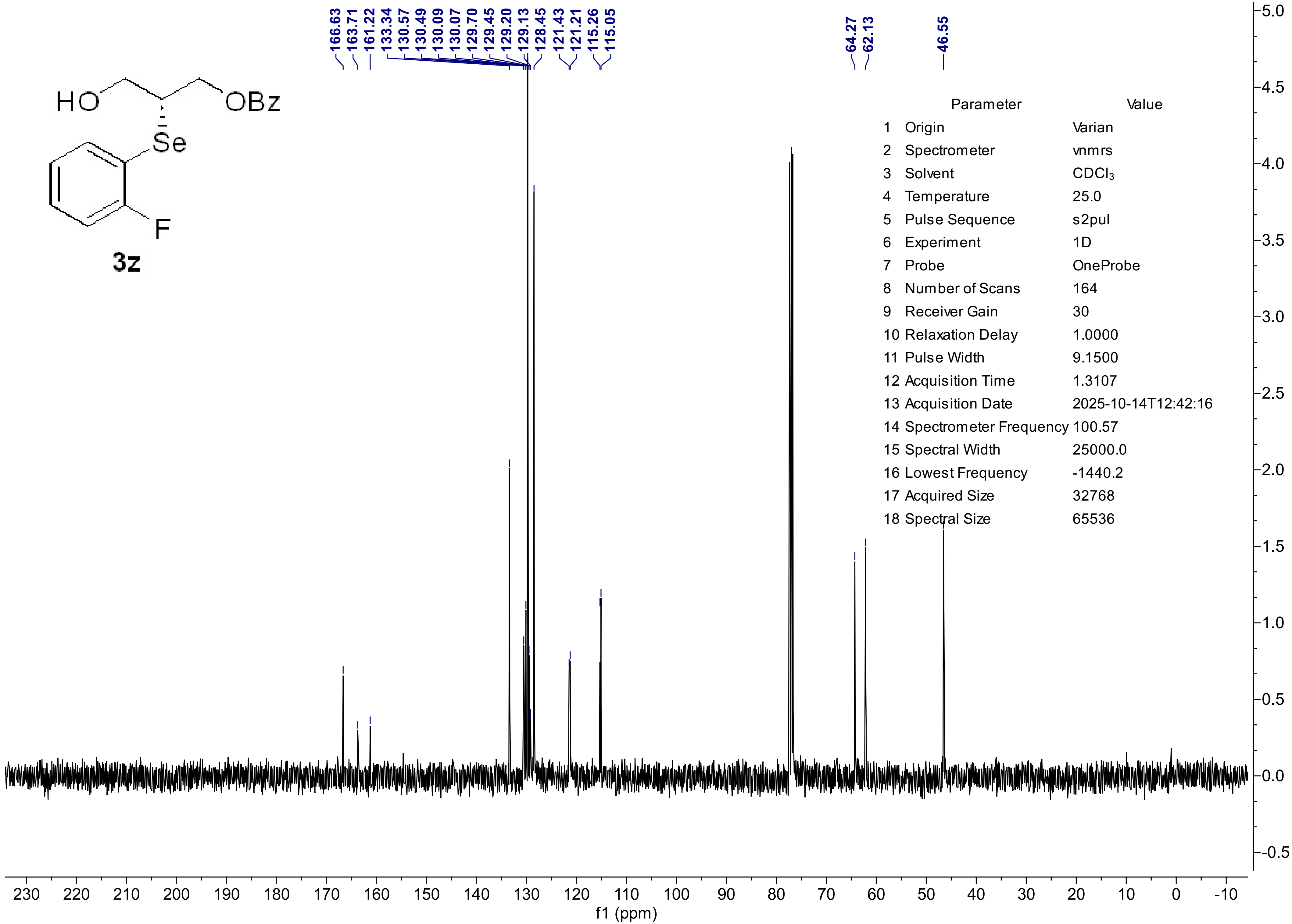

	Parameter	Value
1	Origin	Varian
2	Spectrometer	vnmrs
3	Solvent	$CDCl_3$
4	Temperature	25.0
5	Pulse Sequence	s2pul
6	Experiment	1D
7	Probe	OneProbe
8	Number of Scans	164
9	Receiver Gain	30
10	Relaxation Delay	1.0000
11	Pulse Width	9.1500
12	Acquisition Time	1.3107
13	Acquisition Date	2025-10-14T12:42:16
14	Spectrometer Frequency	100.57
15	Spectral Width	25000.0
16	Lowest Frequency	-1440.2
17	Acquired Size	32768
18	Spectral Size	65536

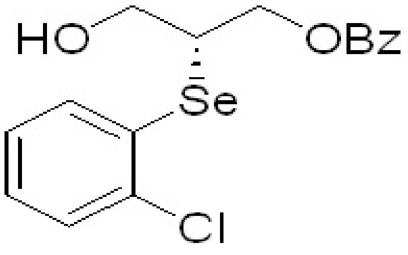

3aa

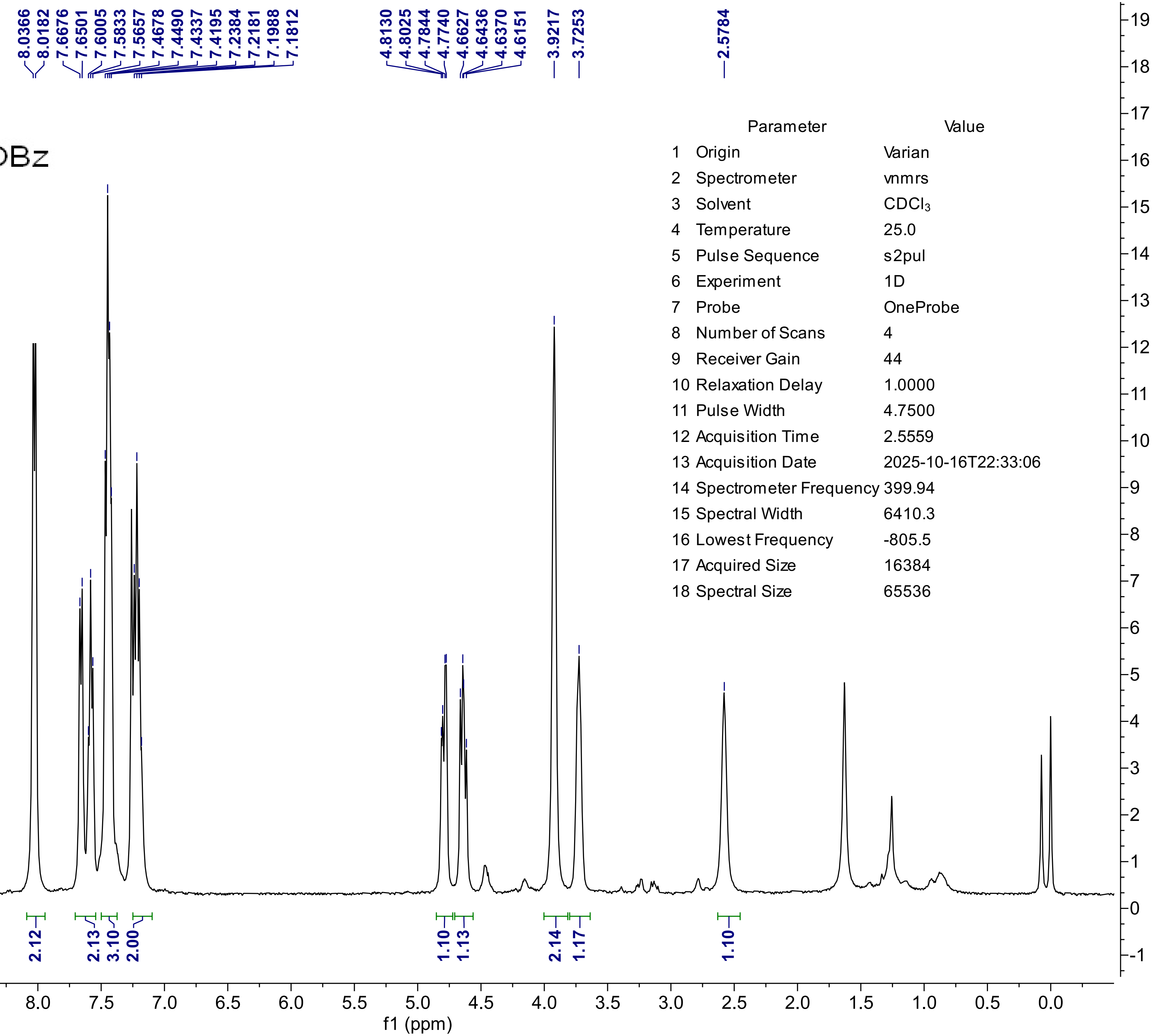

	Parameter	Value
1	Origin	Varian
2	Spectrometer	vnmrs
3	Solvent	$CDCl_3$
4	Temperature	25.0
5	Pulse Sequence	s2pul
6	Experiment	1D
7	Probe	OneProbe
8	Number of Scans	4
9	Receiver Gain	44
10	Relaxation Delay	1.0000
11	Pulse Width	4.7500
12	Acquisition Time	2.5559
13	Acquisition Date	2025-10-16T22:33:06
14	Spectrometer Frequency	399.94
15	Spectral Width	6410.3
16	Lowest Frequency	-805.5
17	Acquired Size	16384
18	Spectral Size	65536

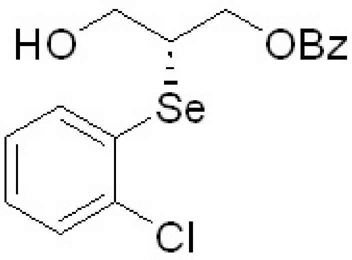

3aa

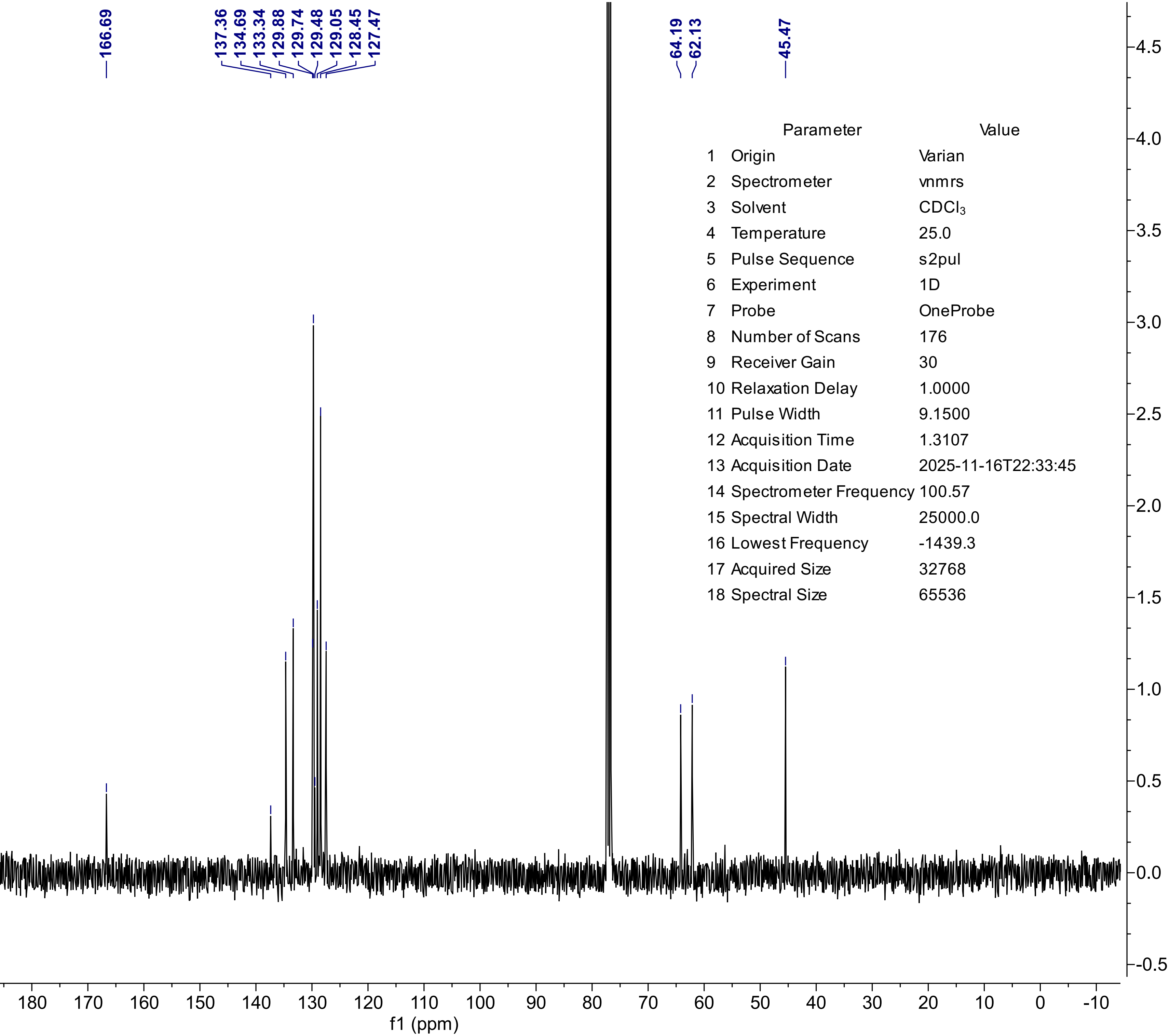

	Parameter	Value
1	Origin	Varian
2	Spectrometer	vnmrs
3	Solvent	$CDCl_3$
4	Temperature	25.0
5	Pulse Sequence	s2pul
6	Experiment	1D
7	Probe	OneProbe
8	Number of Scans	176
9	Receiver Gain	30
10	Relaxation Delay	1.0000
11	Pulse Width	9.1500
12	Acquisition Time	1.3107
13	Acquisition Date	2025-11-16T22:33:45
14	Spectrometer Frequency	100.57
15	Spectral Width	25000.0
16	Lowest Frequency	-1439.3
17	Acquired Size	32768
18	Spectral Size	65536

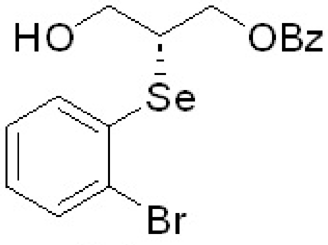

3ab

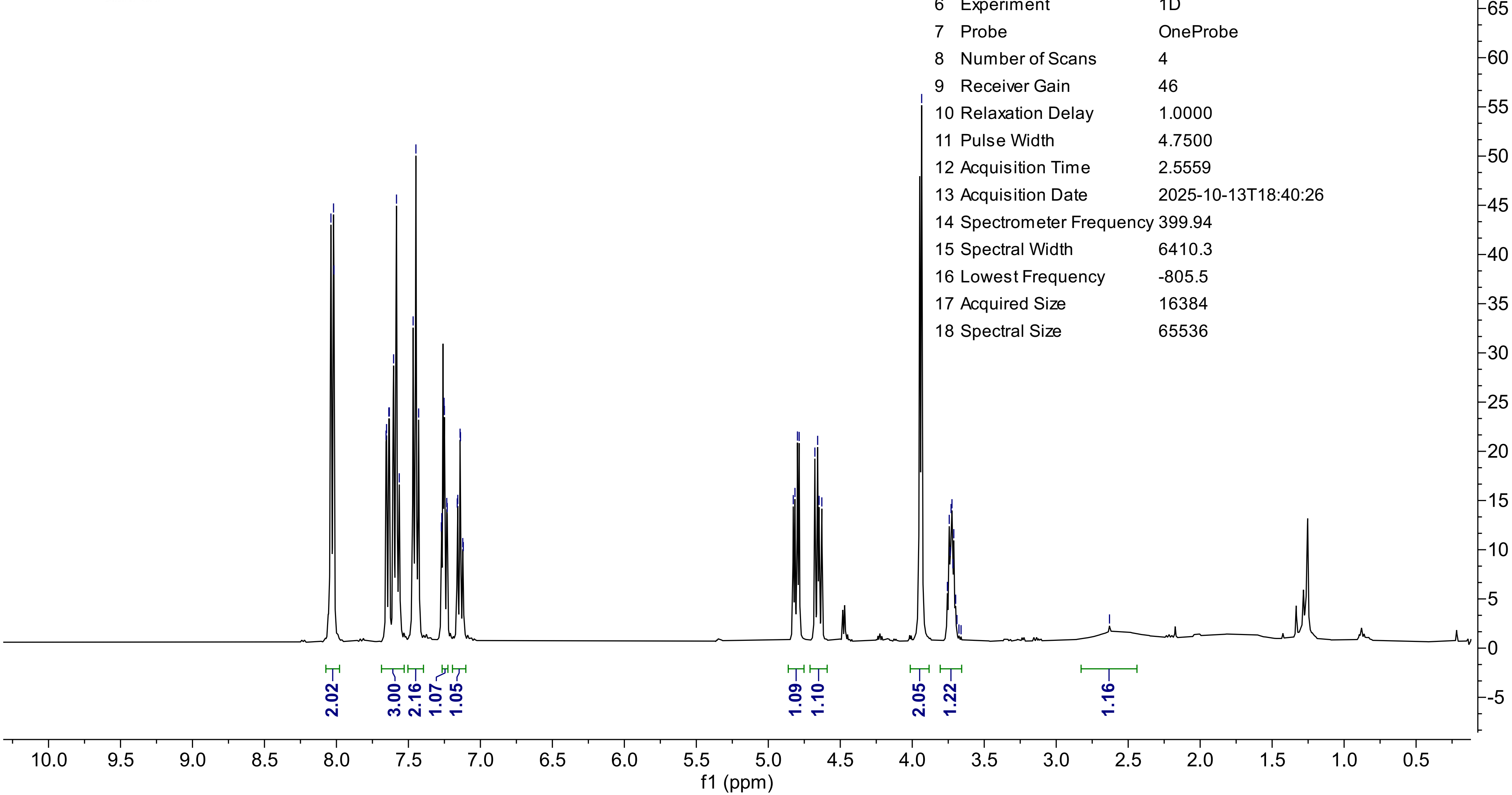

	Parameter	Value
1	Origin	Varian
2	Spectrometer	vnmrs
3	Solvent	$CDCl_3$
4	Temperature	25.0
5	Pulse Sequence	s2pul
6	Experiment	1D
7	Probe	OneProbe
8	Number of Scans	4
9	Receiver Gain	46
10	Relaxation Delay	1.0000
11	Pulse Width	4.7500
12	Acquisition Time	2.5559
13	Acquisition Date	2025-10-13T18:40:26
14	Spectrometer Frequency	399.94
15	Spectral Width	6410.3
16	Lowest Frequency	-805.5
17	Acquired Size	16384
18	Spectral Size	65536

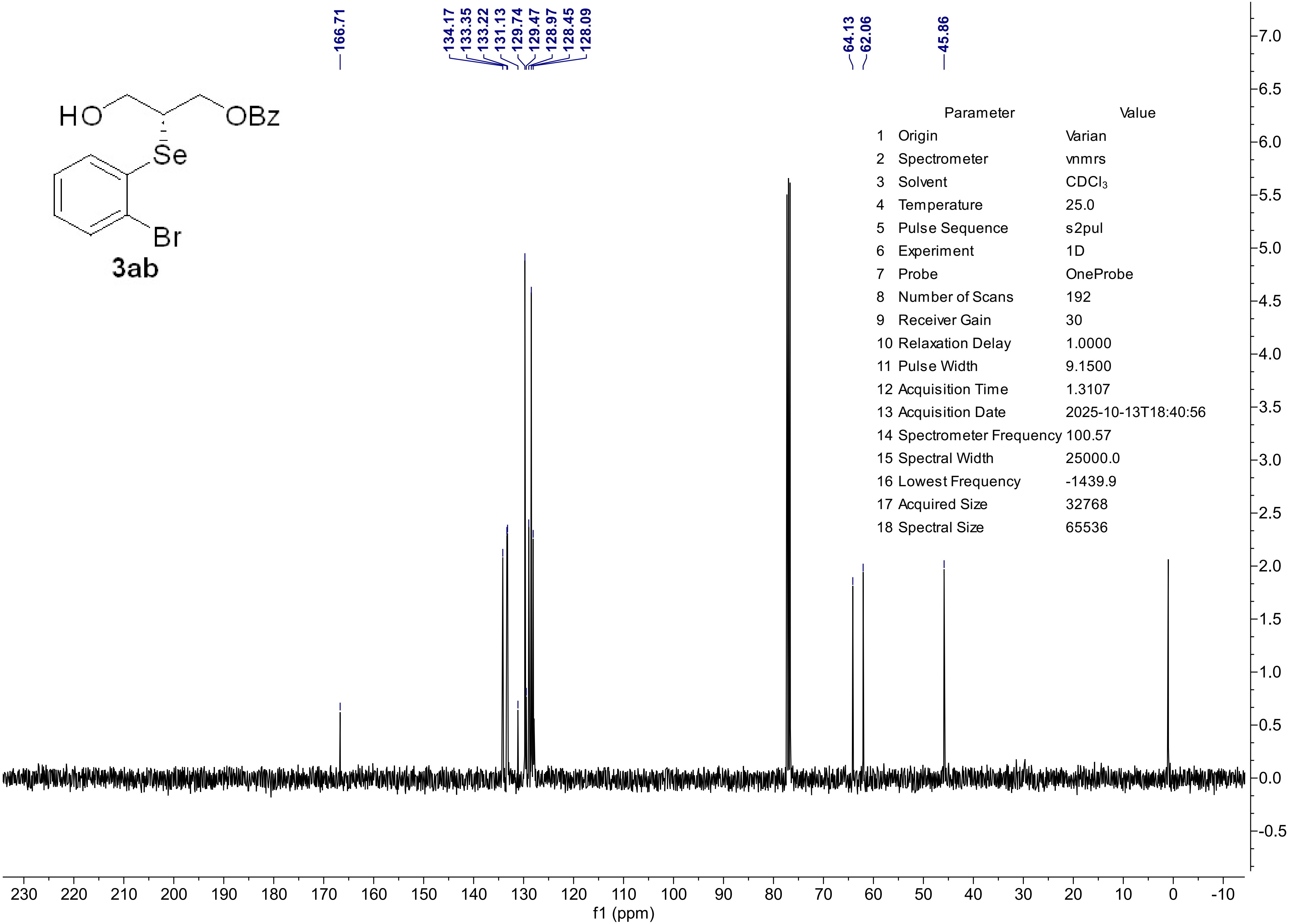
166.71
134.17
133.35
133.22
131.13
129.74
129.47
128.97
128.45
128.09
64.13
62.06
45.86
HO
OBz
Se
Br
3ab
Parameter Value
1 Origin Varian
2 Spectrometer vnmrs
3 Solvent CDCl3
4 Temperature 25.0
5 Pulse Sequence s2pul
6 Experiment 1D
7 Probe OneProbe
8 Number of Scans 192
9 Receiver Gain 30
10 Relaxation Delay 1.0000
11 Pulse Width 9.1500
12 Acquisition Time 1.3107
13 Acquisition Date 2025-10-13T18:40:56
14 Spectrometer Frequency 100.57
15 Spectral Width 25000.0
16 Lowest Frequency -1439.9
17 Acquired Size 32768
18 Spectral Size 65536
f1 (ppm)

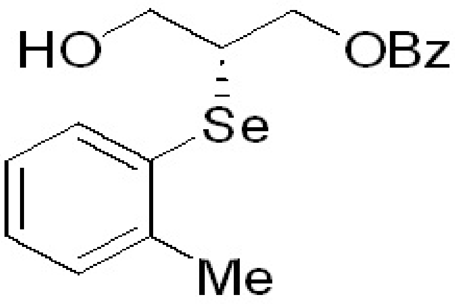

3ac

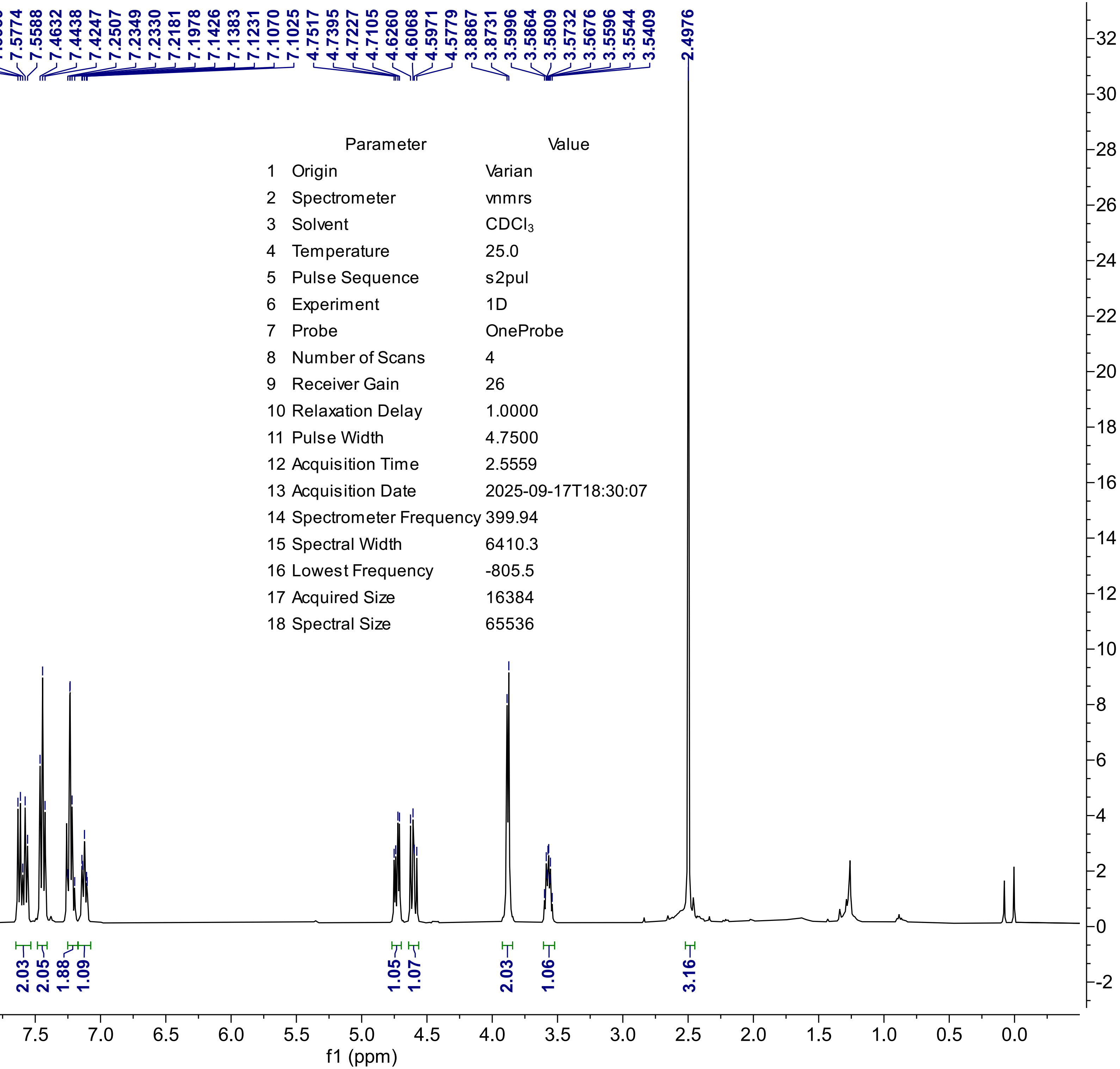

	Parameter	Value
1	Origin	Varian
2	Spectrometer	vnmrs
3	Solvent	$CDCl_3$
4	Temperature	25.0
5	Pulse Sequence	s2pul
6	Experiment	1D
7	Probe	OneProbe
8	Number of Scans	4
9	Receiver Gain	26
10	Relaxation Delay	1.0000
11	Pulse Width	4.7500
12	Acquisition Time	2.5559
13	Acquisition Date	2025-09-17T18:30:07
14	Spectrometer Frequency	399.94
15	Spectral Width	6410.3
16	Lowest Frequency	-805.5
17	Acquired Size	16384
18	Spectral Size	65536

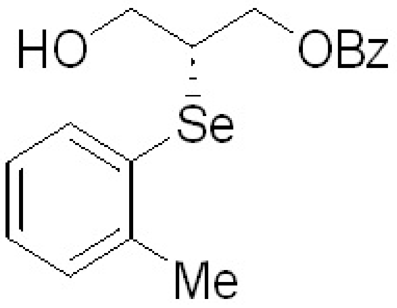

3ac

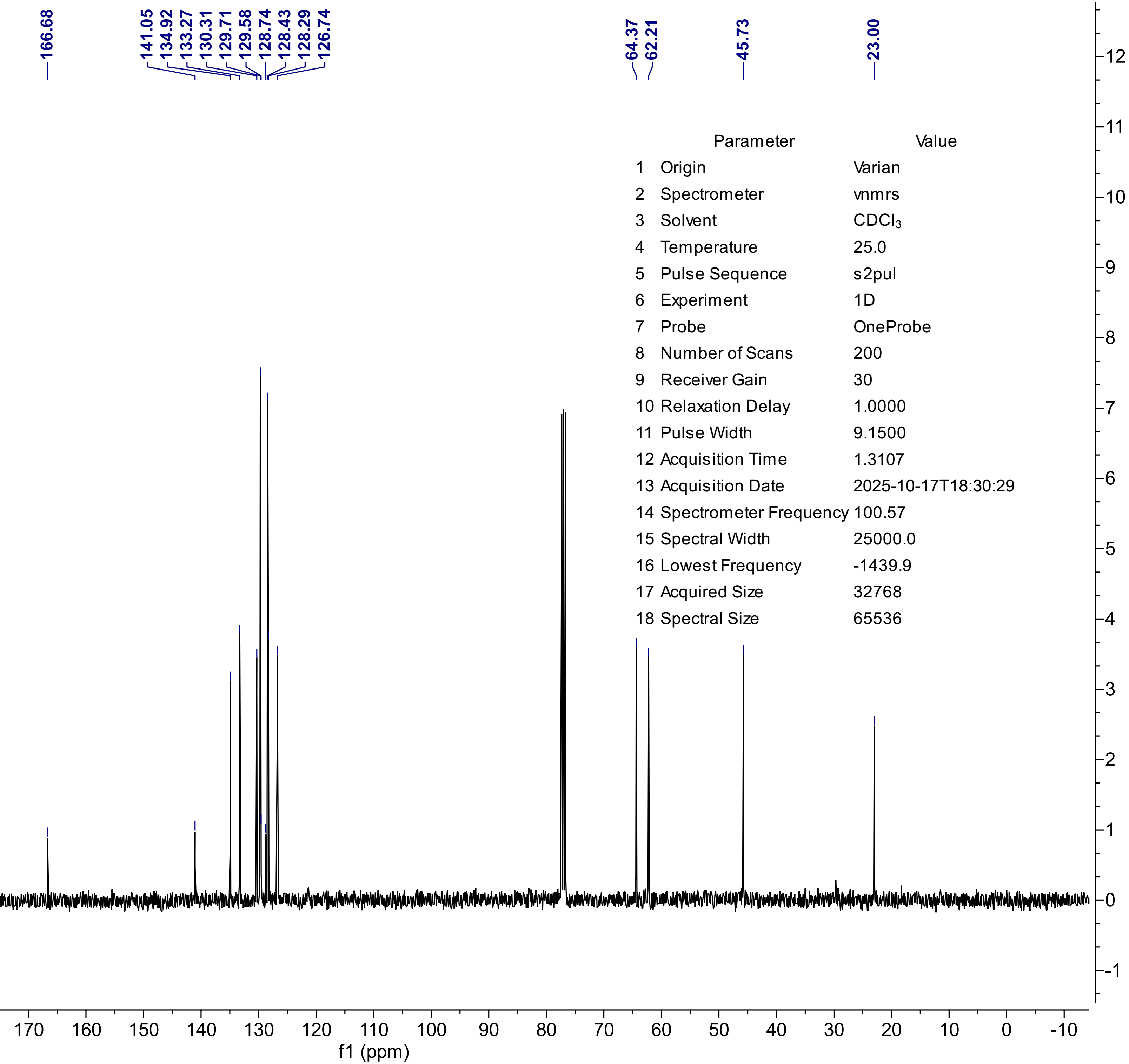

	Parameter	Value
1	Origin	Varian
2	Spectrometer	vnmrs
3	Solvent	$CDCl_3$
4	Temperature	25.0
5	Pulse Sequence	s2pul
6	Experiment	1D
7	Probe	OneProbe
8	Number of Scans	200
9	Receiver Gain	30
10	Relaxation Delay	1.0000
11	Pulse Width	9.1500
12	Acquisition Time	1.3107
13	Acquisition Date	2025-10-17T18:30:29
14	Spectrometer Frequency	100.57
15	Spectral Width	25000.0
16	Lowest Frequency	-1439.9
17	Acquired Size	32768
18	Spectral Size	65536

HQQ-3-122-H

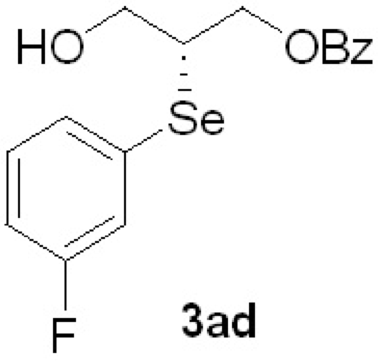

3ad

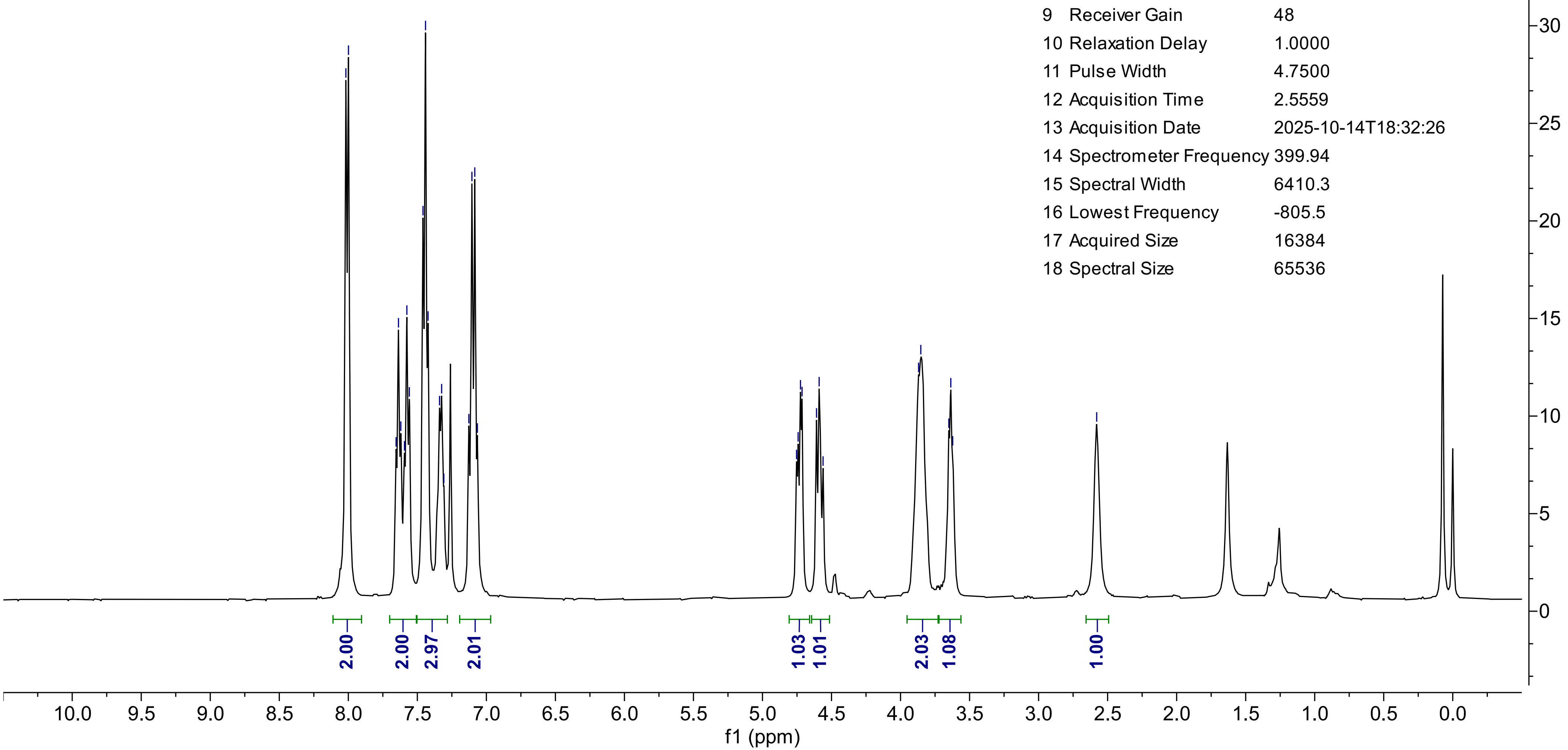

	Parameter	Value
1	Origin	Varian
2	Spectrometer	vnmrs
3	Solvent	$CDCl_3$
4	Temperature	25.0
5	Pulse Sequence	s2pul
6	Experiment	1D
7	Probe	OneProbe
8	Number of Scans	4
9	Receiver Gain	48
10	Relaxation Delay	1.0000
11	Pulse Width	4.7500
12	Acquisition Time	2.5559
13	Acquisition Date	2025-10-14T18:32:26
14	Spectrometer Frequency	399.94
15	Spectral Width	6410.3
16	Lowest Frequency	-805.5
17	Acquired Size	16384
18	Spectral Size	65536

HQQ-3-122-C

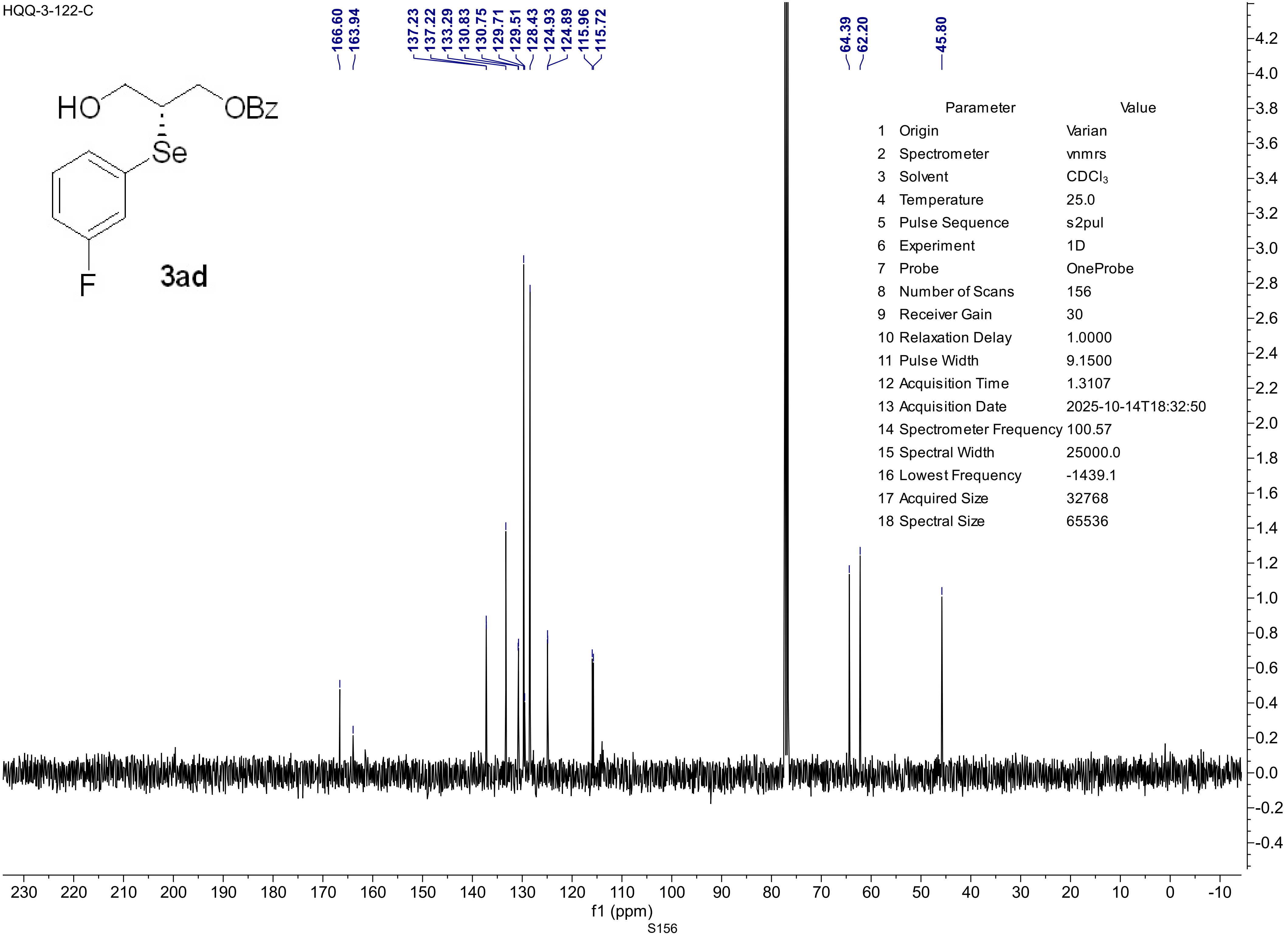

	Parameter	Value
1	Origin	Varian
2	Spectrometer	vnmrs
3	Solvent	$CDCl_3$
4	Temperature	25.0
5	Pulse Sequence	s2pul
6	Experiment	1D
7	Probe	OneProbe
8	Number of Scans	156
9	Receiver Gain	30
10	Relaxation Delay	1.0000
11	Pulse Width	9.1500
12	Acquisition Time	1.3107
13	Acquisition Date	2025-10-14T18:32:50
14	Spectrometer Frequency	100.57
15	Spectral Width	25000.0
16	Lowest Frequency	-1439.1
17	Acquired Size	32768
18	Spectral Size	65536

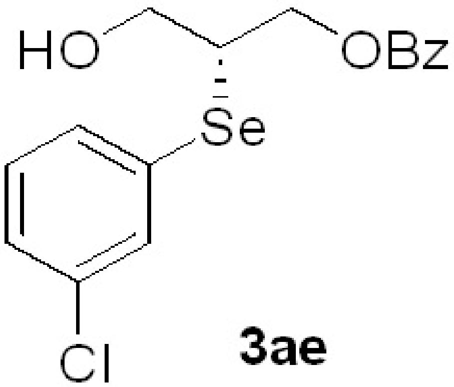

3ae

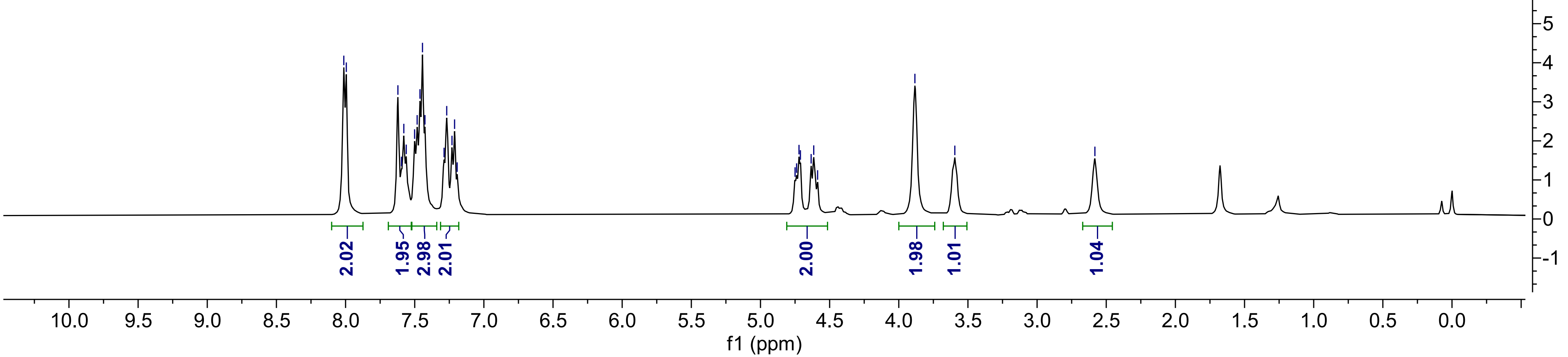

	Parameter	Value
1	Origin	Varian
2	Spectrometer	vnmrs
3	Solvent	$CDCl_3$
4	Temperature	25.0
5	Pulse Sequence	s2pul
6	Experiment	1D
7	Probe	OneProbe
8	Number of Scans	4
9	Receiver Gain	36
10	Relaxation Delay	1.0000
11	Pulse Width	4.7500
12	Acquisition Time	2.5559
13	Acquisition Date	2026-03-17 T20:50:39
14	Spectrometer Frequency	399.94
15	Spectral Width	6410.3
16	Lowest Frequency	-805.5
17	Acquired Size	16384
18	Spectral Size	65536

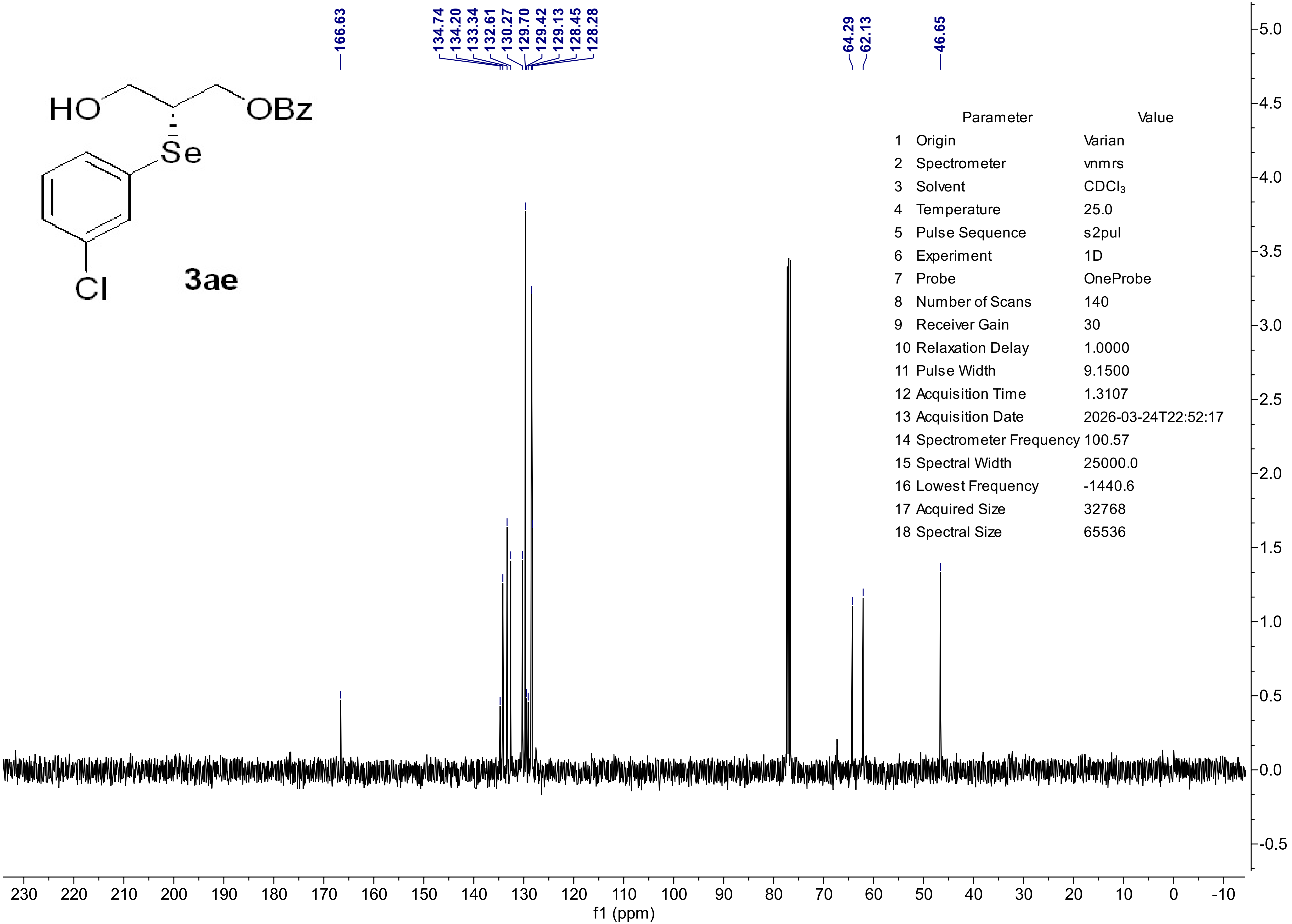

HO
OBz
Se
Cl
3ae
166.63
134.74
134.20
133.34
132.61
130.27
129.70
129.42
129.13
128.45
128.28
64.29
62.13
46.65
Parameter Value
1 Origin Varian
2 Spectrometer vnmrs
3 Solvent CDCl3
4 Temperature 25.0
5 Pulse Sequence s2pul
6 Experiment 1D
7 Probe OneProbe
8 Number of Scans 140
9 Receiver Gain 30
10 Relaxation Delay 1.0000
11 Pulse Width 9.1500
12 Acquisition Time 1.3107
13 Acquisition Date 2026-03-24T22:52:17
14 Spectrometer Frequency 100.57
15 Spectral Width 25000.0
16 Lowest Frequency -1440.6
17 Acquired Size 32768
18 Spectral Size 65536
f1 (ppm)

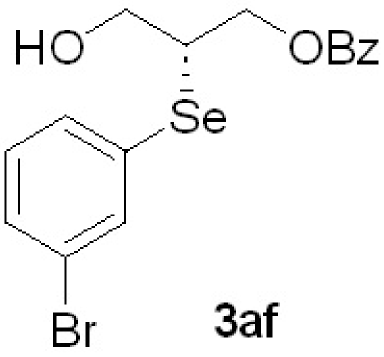

3af

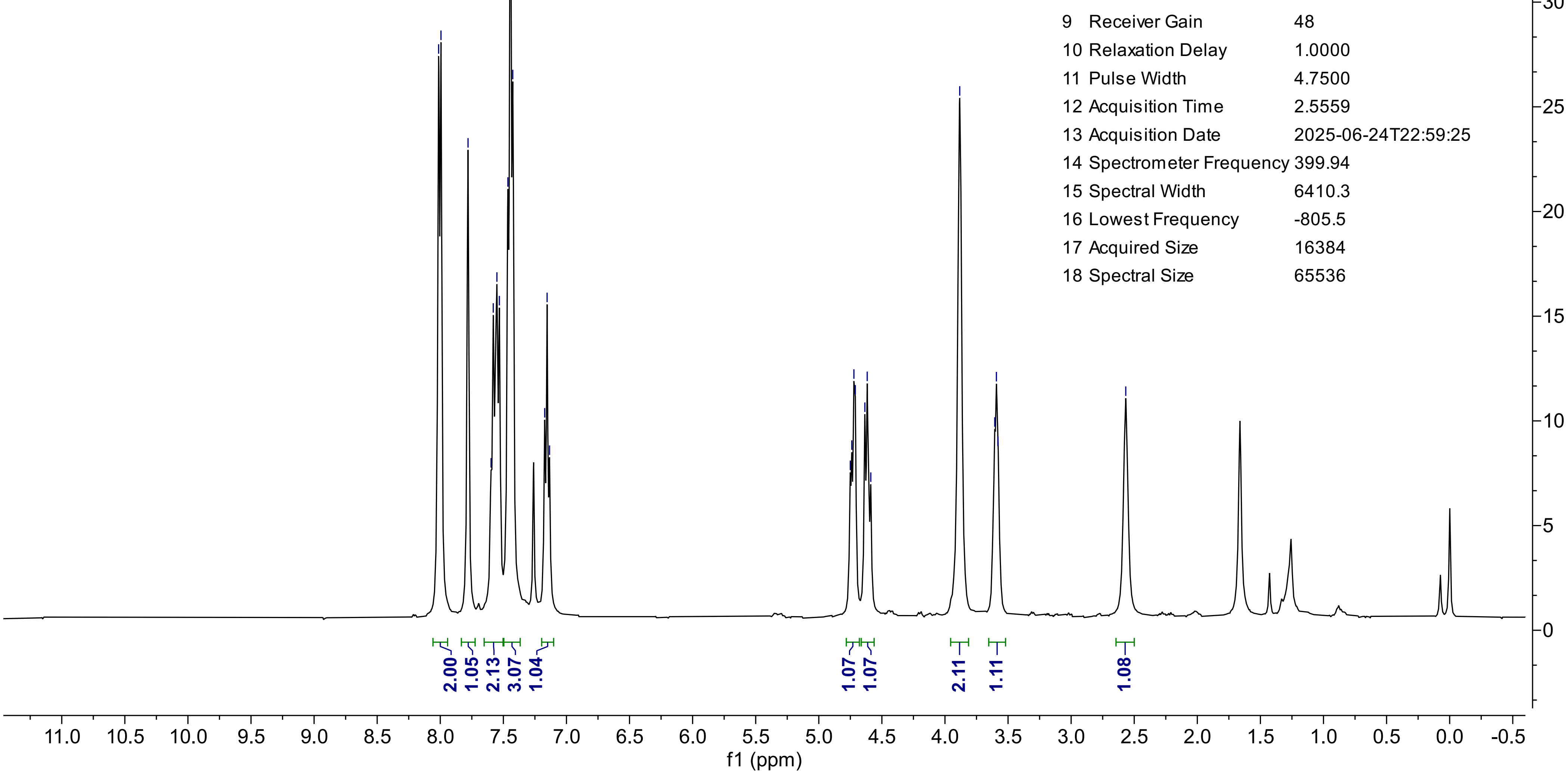

	Parameter	Value
1	Origin	Varian
2	Spectrometer	vnmrs
3	Solvent	$CDCl_3$
4	Temperature	25.0
5	Pulse Sequence	s2pul
6	Experiment	1D
7	Probe	OneProbe
8	Number of Scans	4
9	Receiver Gain	48
10	Relaxation Delay	1.0000
11	Pulse Width	4.7500
12	Acquisition Time	2.5559
13	Acquisition Date	2025-06-24T22:59:25
14	Spectrometer Frequency	399.94
15	Spectral Width	6410.3
16	Lowest Frequency	-805.5
17	Acquired Size	16384
18	Spectral Size	65536

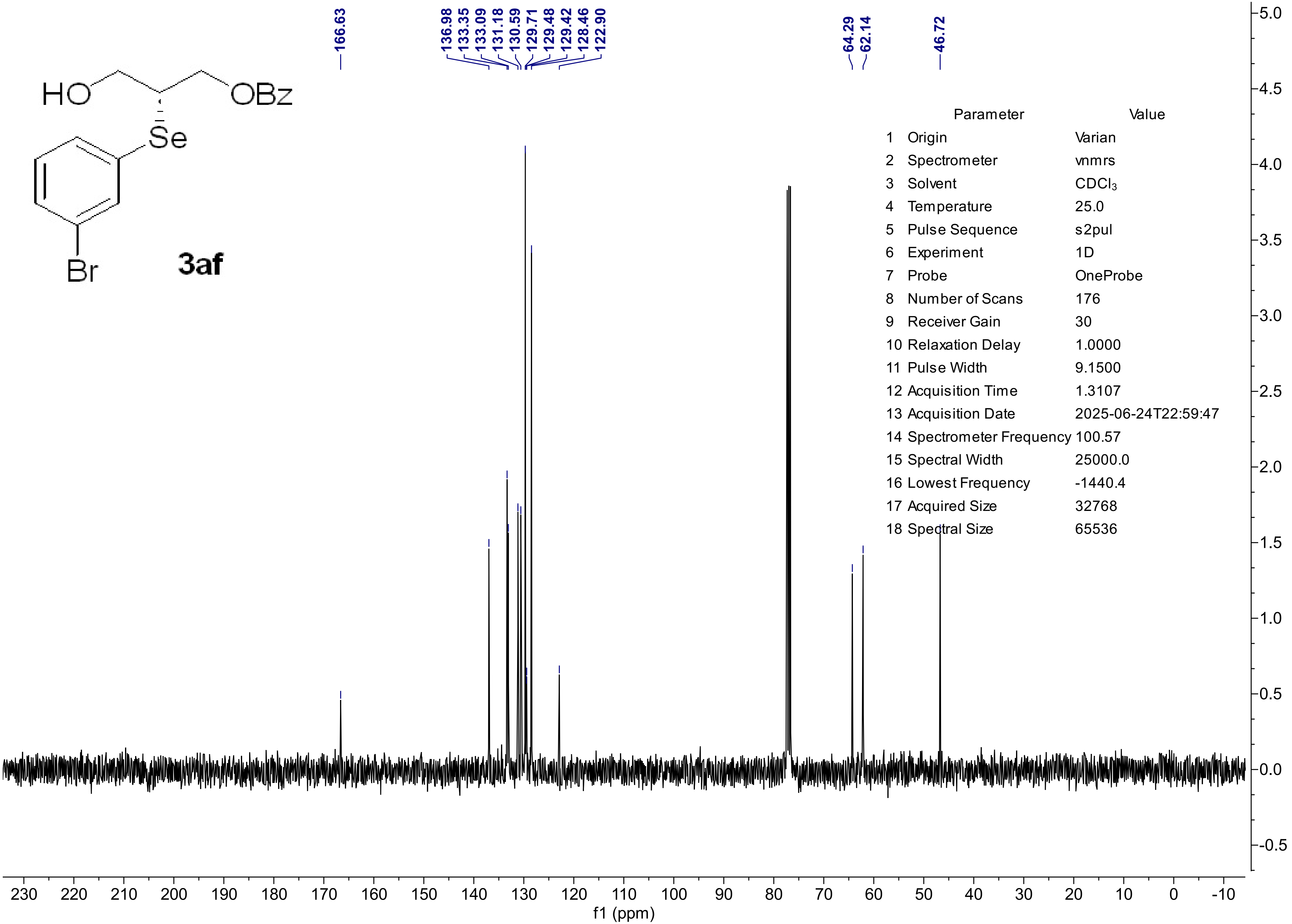

3af

	Parameter	Value
1	Origin	Varian
2	Spectrometer	vnmrs
3	Solvent	$CDCl_3$
4	Temperature	25.0
5	Pulse Sequence	s2pul
6	Experiment	1D
7	Probe	OneProbe
8	Number of Scans	176
9	Receiver Gain	30
10	Relaxation Delay	1.0000
11	Pulse Width	9.1500
12	Acquisition Time	1.3107
13	Acquisition Date	2025-06-24T22:59:47
14	Spectrometer Frequency	100.57
15	Spectral Width	25000.0
16	Lowest Frequency	-1440.4
17	Acquired Size	32768
18	Spectral Size	65536

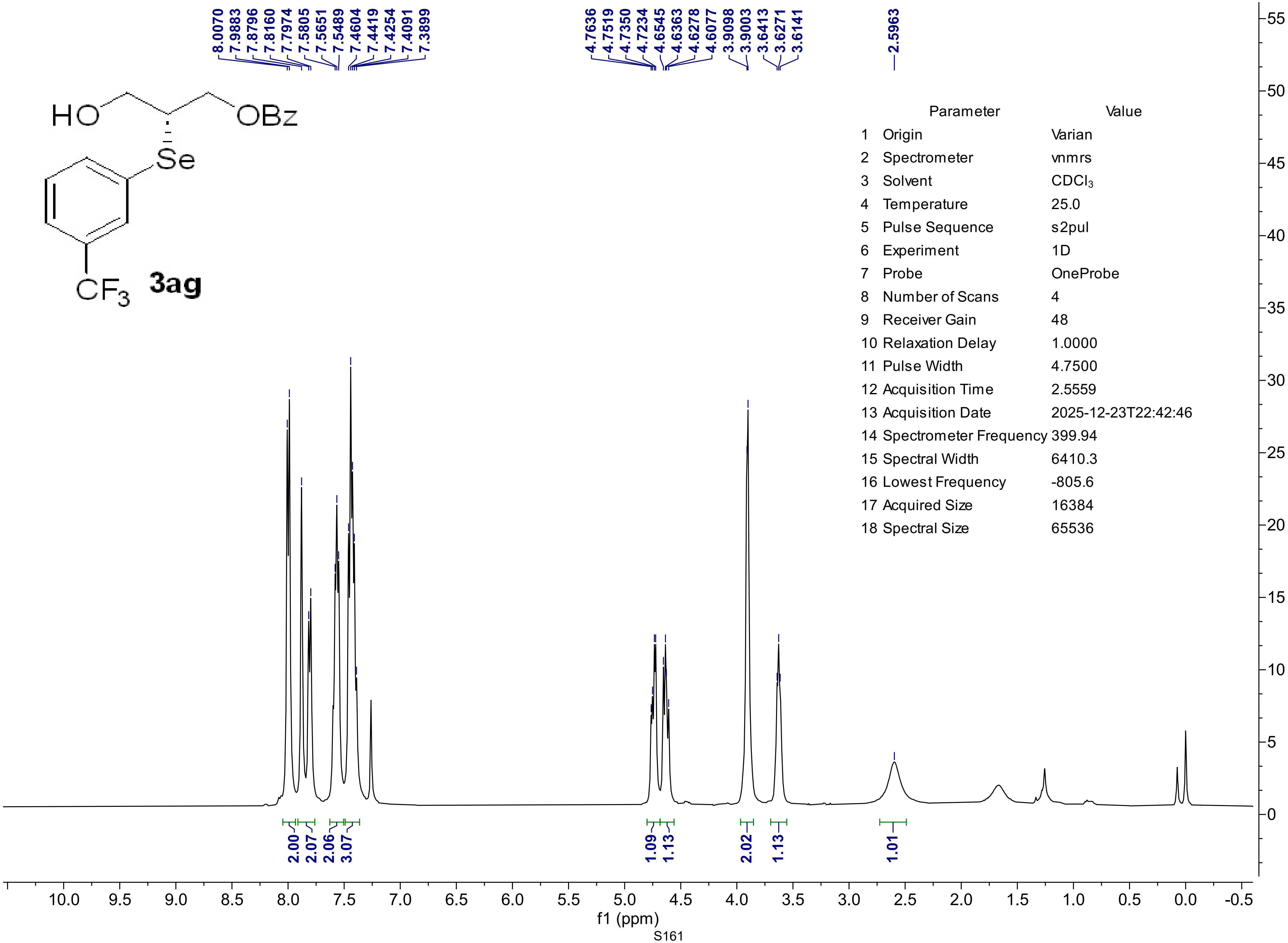

	Parameter	Value
1	Origin	Varian
2	Spectrometer	vnmrs
3	Solvent	$CDCl_3$
4	Temperature	25.0
5	Pulse Sequence	s2pul
6	Experiment	1D
7	Probe	OneProbe
8	Number of Scans	4
9	Receiver Gain	48
10	Relaxation Delay	1.0000
11	Pulse Width	4.7500
12	Acquisition Time	2.5559
13	Acquisition Date	2025-12-23T22:42:46
14	Spectrometer Frequency	399.94
15	Spectral Width	6410.3
16	Lowest Frequency	-805.6
17	Acquired Size	16384
18	Spectral Size	65536

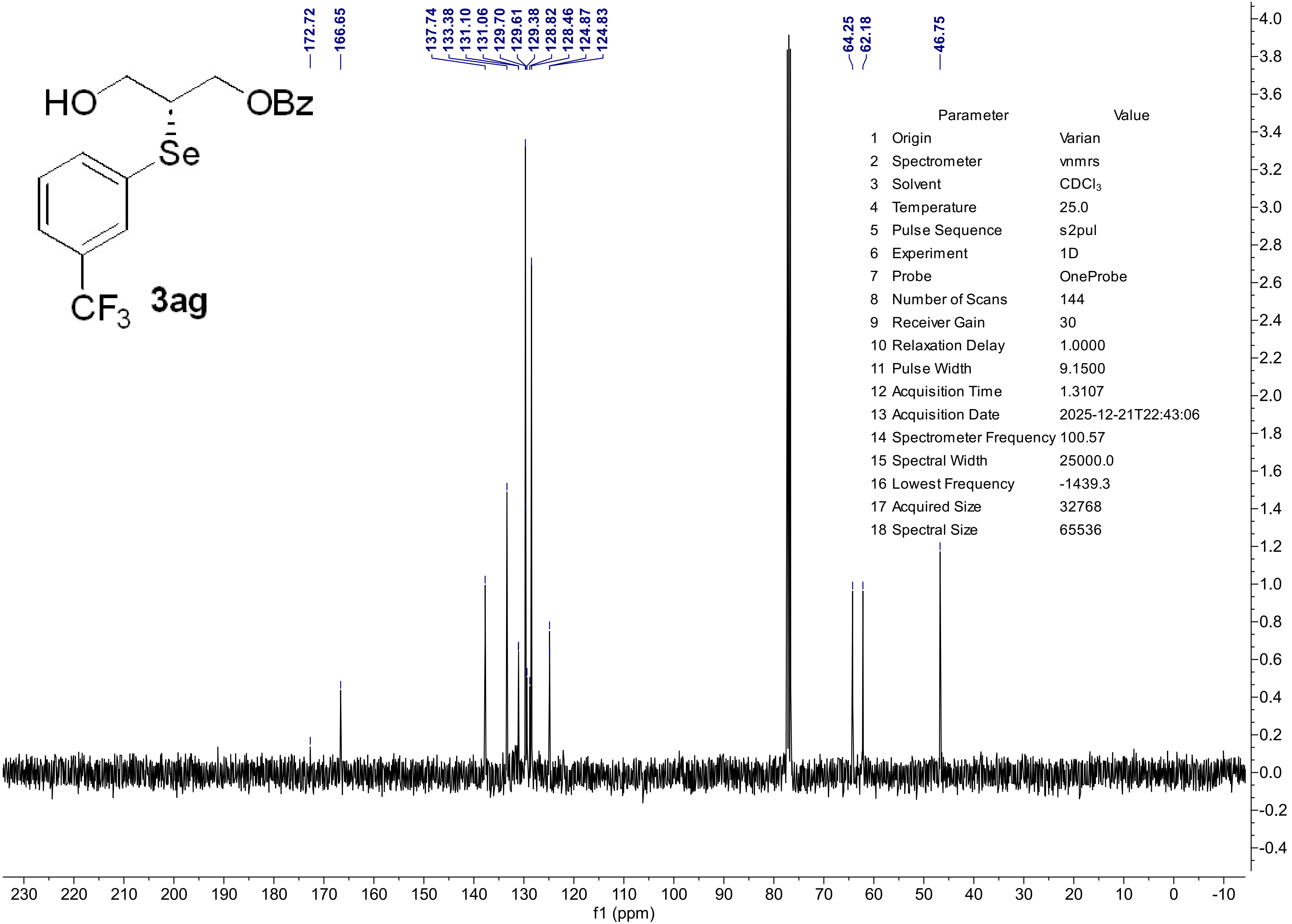

	Parameter	Value
1	Origin	Varian
2	Spectrometer	vnmrs
3	Solvent	$CDCl_3$
4	Temperature	25.0
5	Pulse Sequence	s2pul
6	Experiment	1D
7	Probe	OneProbe
8	Number of Scans	144
9	Receiver Gain	30
10	Relaxation Delay	1.0000
11	Pulse Width	9.1500
12	Acquisition Time	1.3107
13	Acquisition Date	2025-12-21T22:43:06
14	Spectrometer Frequency	100.57
15	Spectral Width	25000.0
16	Lowest Frequency	-1439.3
17	Acquired Size	32768
18	Spectral Size	65536

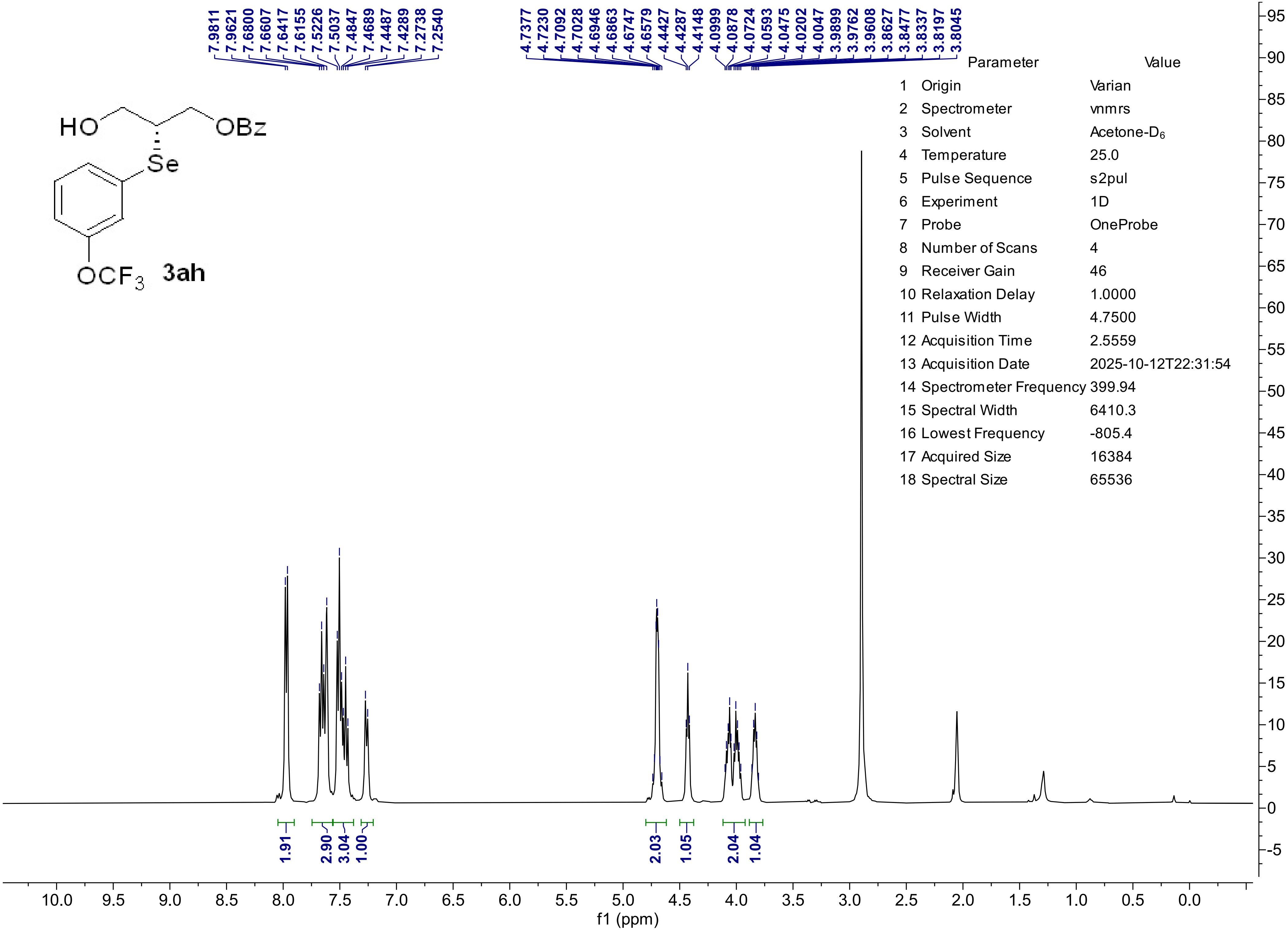

3ah

	Parameter	Value
1	Origin	Varian
2	Spectrometer	vnmrs
3	Solvent	Acetone-D_6
4	Temperature	25.0
5	Pulse Sequence	s2pul
6	Experiment	1D
7	Probe	OneProbe
8	Number of Scans	4
9	Receiver Gain	46
10	Relaxation Delay	1.0000
11	Pulse Width	4.7500
12	Acquisition Time	2.5559
13	Acquisition Date	2025-10-12T22:31:54
14	Spectrometer Frequency	399.94
15	Spectral Width	6410.3
16	Lowest Frequency	-805.4
17	Acquired Size	16384
18	Spectral Size	65536

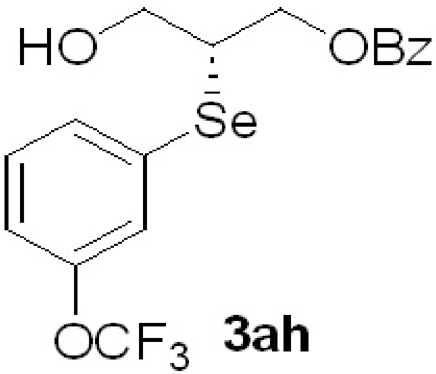

3ah

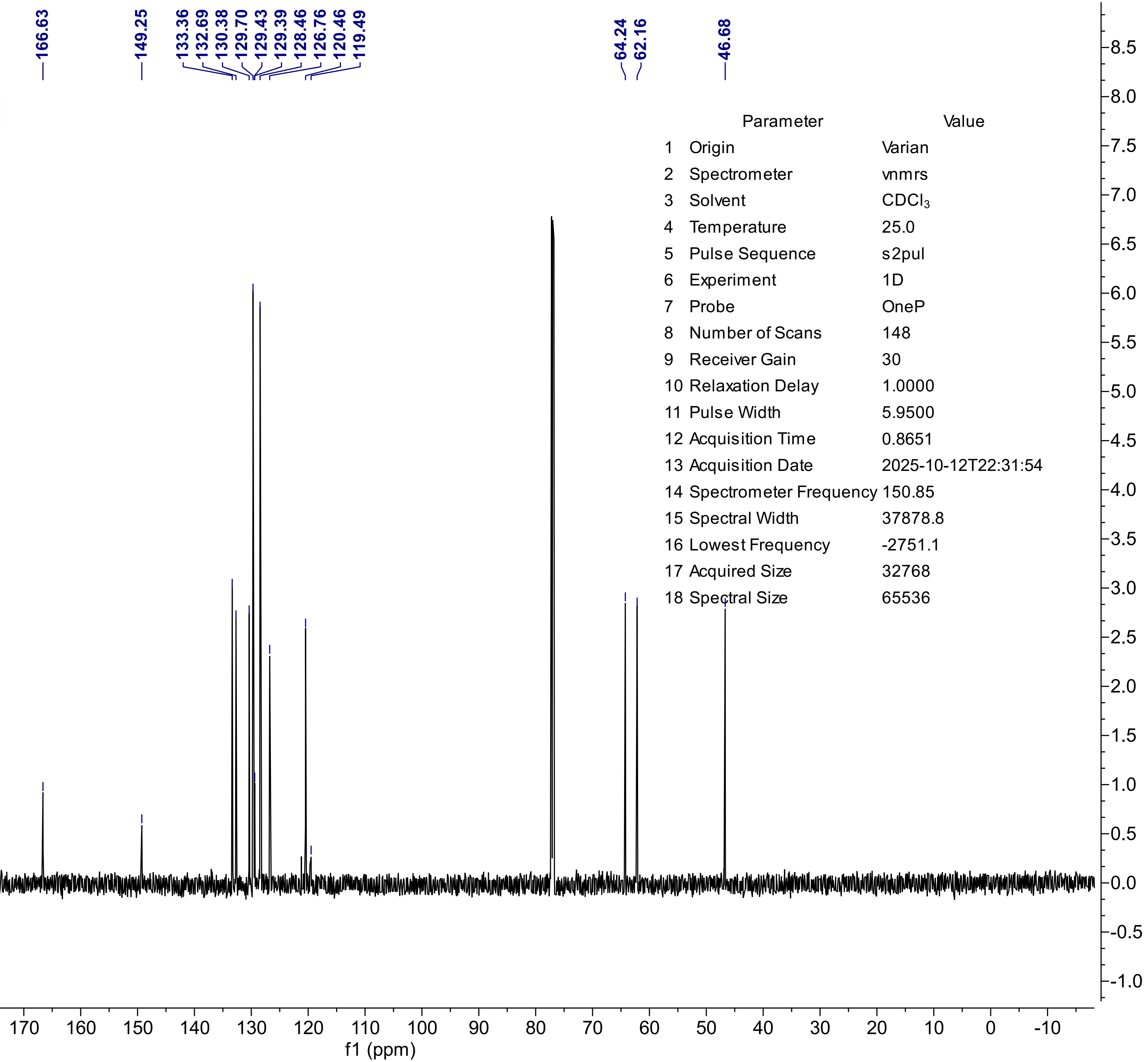

	Parameter	Value
1	Origin	Varian
2	Spectrometer	vnmrs
3	Solvent	$CDCl_3$
4	Temperature	25.0
5	Pulse Sequence	s2pul
6	Experiment	1D
7	Probe	OneP
8	Number of Scans	148
9	Receiver Gain	30
10	Relaxation Delay	1.0000
11	Pulse Width	5.9500
12	Acquisition Time	0.8651
13	Acquisition Date	2025-10-12T22:31:54
14	Spectrometer Frequency	150.85
15	Spectral Width	37878.8
16	Lowest Frequency	-2751.1
17	Acquired Size	32768
18	Spectral Size	65536

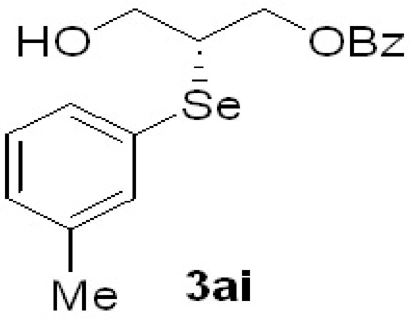

3ai

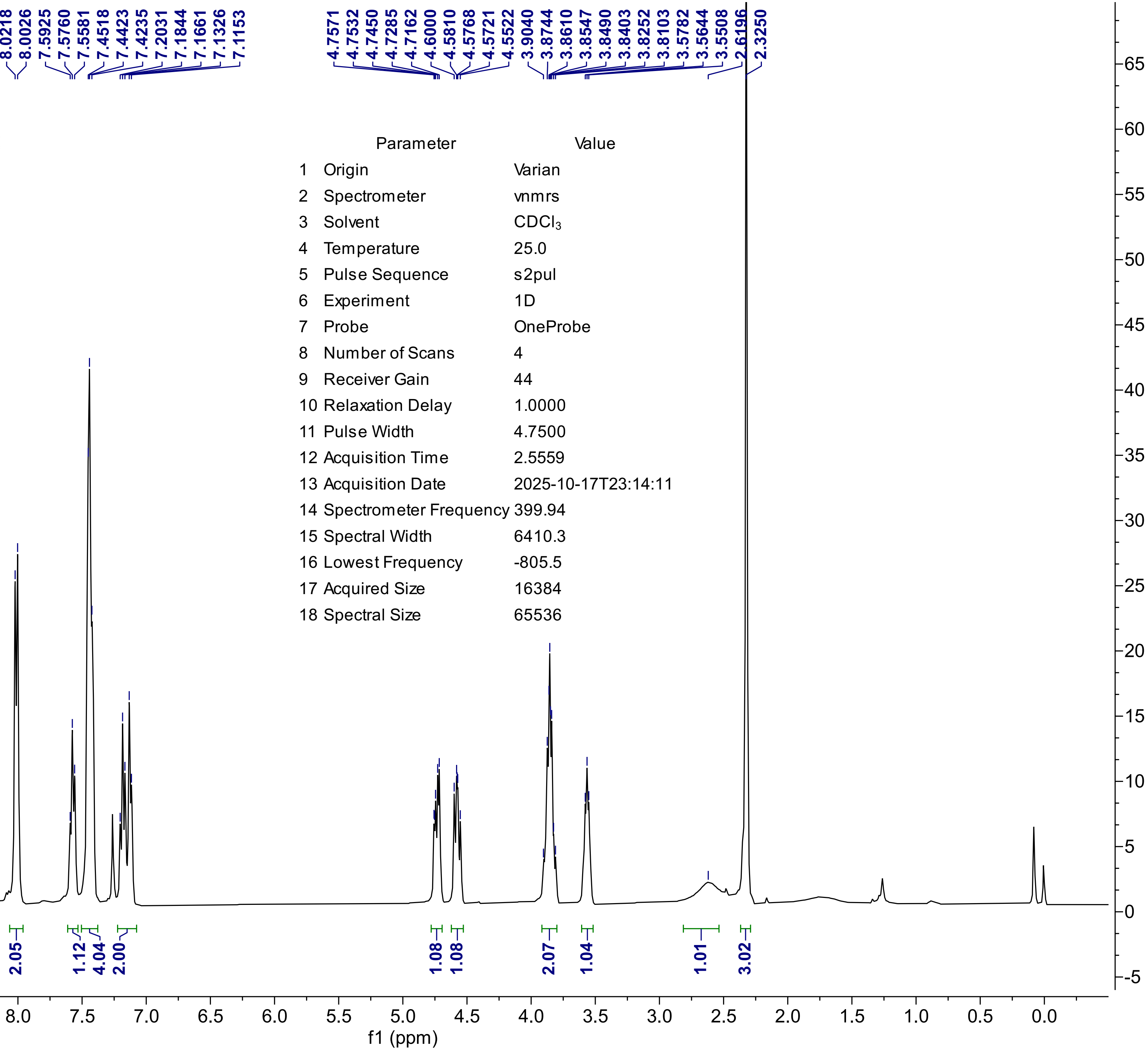

	Parameter	Value
1	Origin	Varian
2	Spectrometer	vnmrs
3	Solvent	$CDCl_3$
4	Temperature	25.0
5	Pulse Sequence	s2pul
6	Experiment	1D
7	Probe	OneProbe
8	Number of Scans	4
9	Receiver Gain	44
10	Relaxation Delay	1.0000
11	Pulse Width	4.7500
12	Acquisition Time	2.5559
13	Acquisition Date	2025-10-17T23:14:11
14	Spectrometer Frequency	399.94
15	Spectral Width	6410.3
16	Lowest Frequency	-805.5
17	Acquired Size	16384
18	Spectral Size	65536

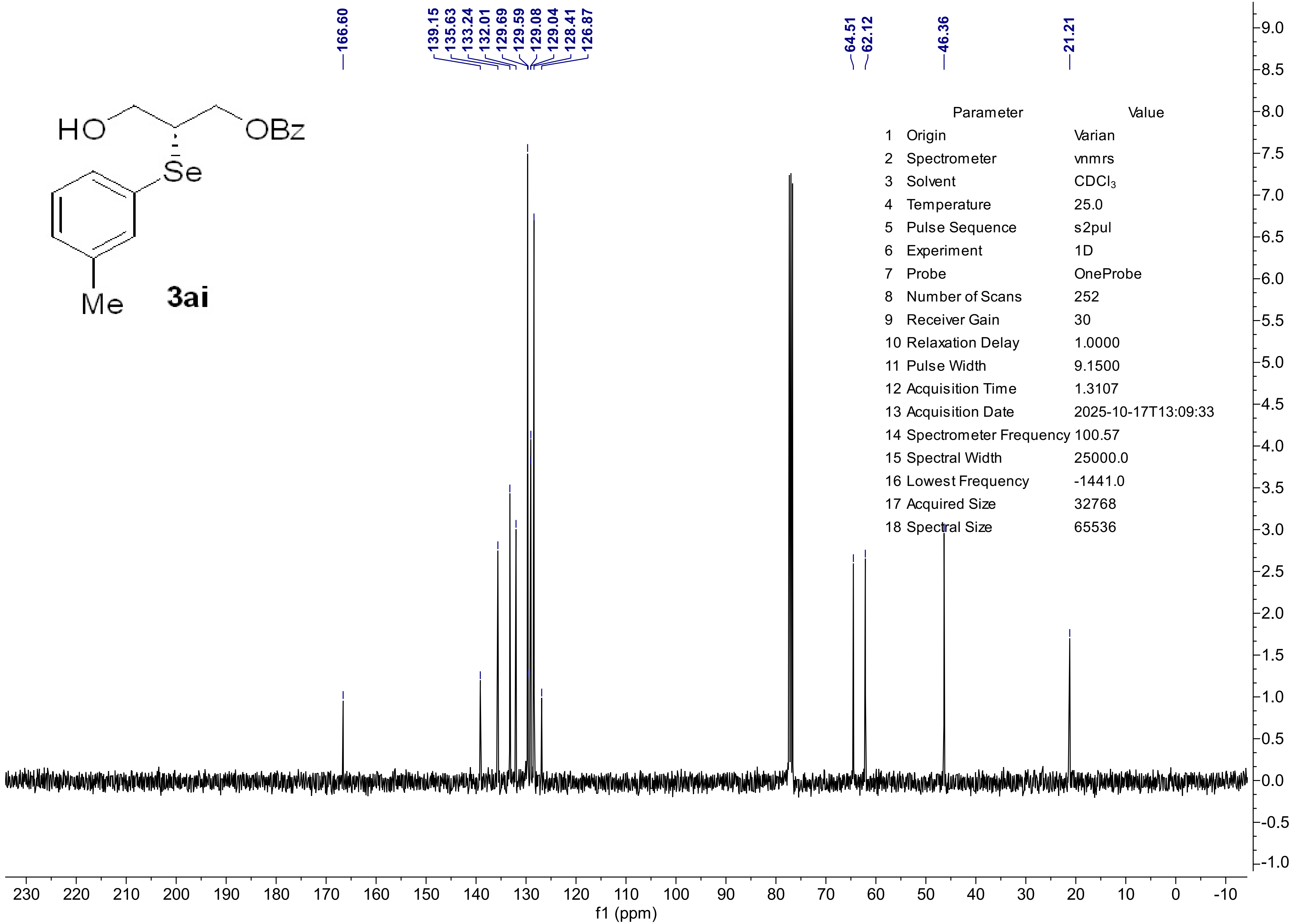
166.60
139.15
135.63
133.24
132.01
129.69
129.59
129.08
129.04
128.41
126.87
64.51
62.12
46.36
21.21
HO
OBz
Se
Me
3ai
Parameter Value
1 Origin Varian
2 Spectrometer vnmrs
3 Solvent CDCl3
4 Temperature 25.0
5 Pulse Sequence s2pul
6 Experiment 1D
7 Probe OneProbe
8 Number of Scans 252
9 Receiver Gain 30
10 Relaxation Delay 1.0000
11 Pulse Width 9.1500
12 Acquisition Time 1.3107
13 Acquisition Date 2025-10-17T13:09:33
14 Spectrometer Frequency 100.57
15 Spectral Width 25000.0
16 Lowest Frequency -1441.0
17 Acquired Size 32768
18 Spectral Size 65536
f1 (ppm)

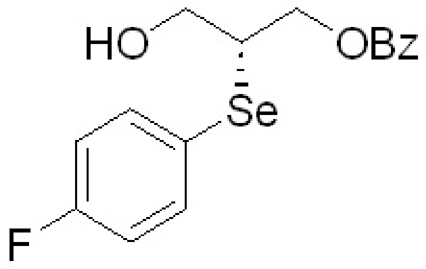

3aj

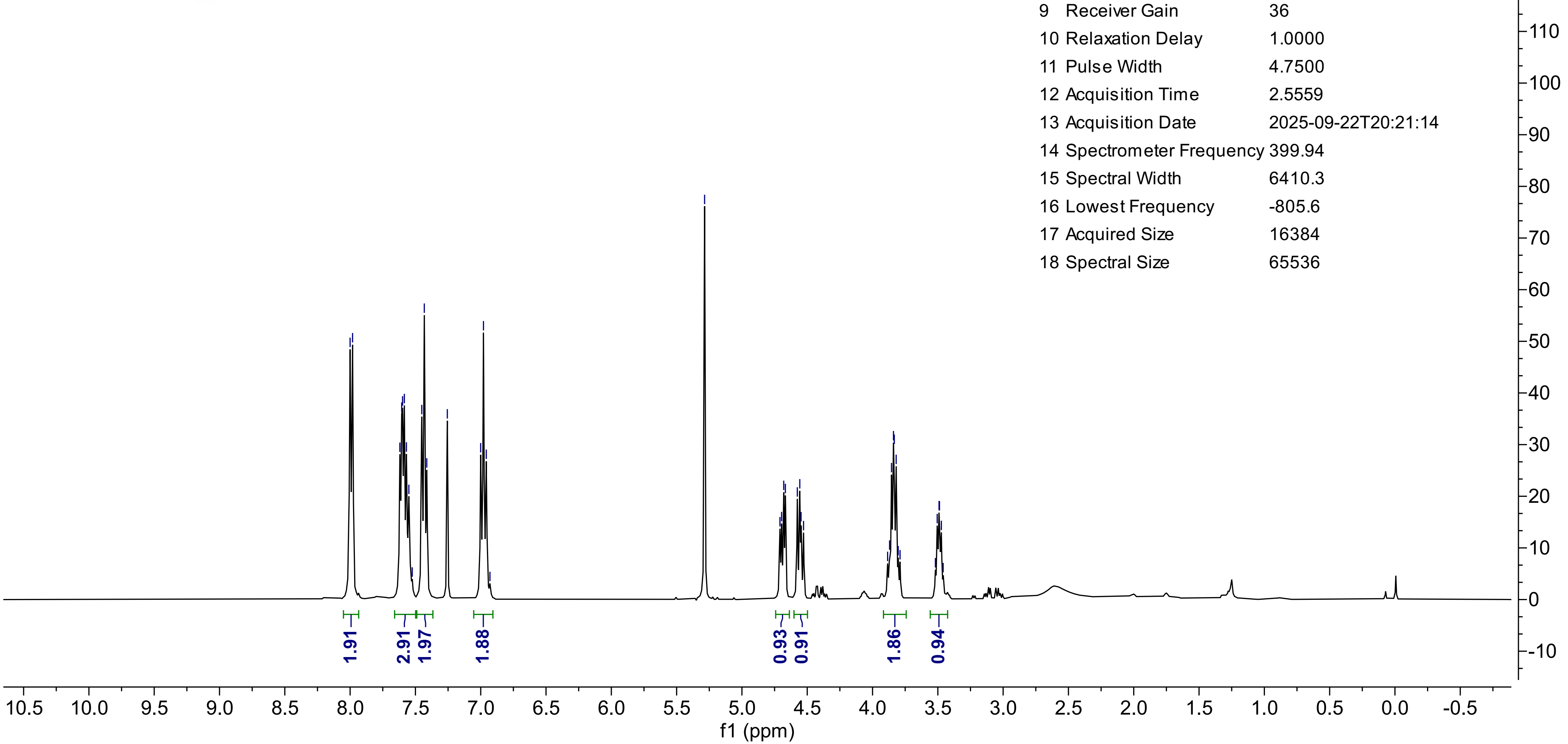

	Parameter	Value
1	Origin	Varian
2	Spectrometer	vnmrs
3	Solvent	$CDCl_3$
4	Temperature	25.0
5	Pulse Sequence	s2pul
6	Experiment	1D
7	Probe	OneProbe
8	Number of Scans	4
9	Receiver Gain	36
10	Relaxation Delay	1.0000
11	Pulse Width	4.7500
12	Acquisition Time	2.5559
13	Acquisition Date	2025-09-22T20:21:14
14	Spectrometer Frequency	399.94
15	Spectral Width	6410.3
16	Lowest Frequency	-805.6
17	Acquired Size	16384
18	Spectral Size	65536

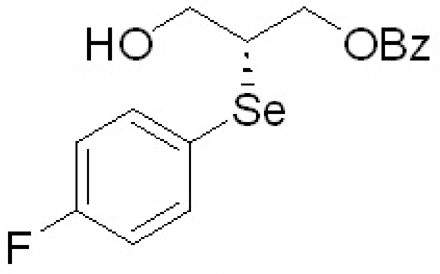

3aj

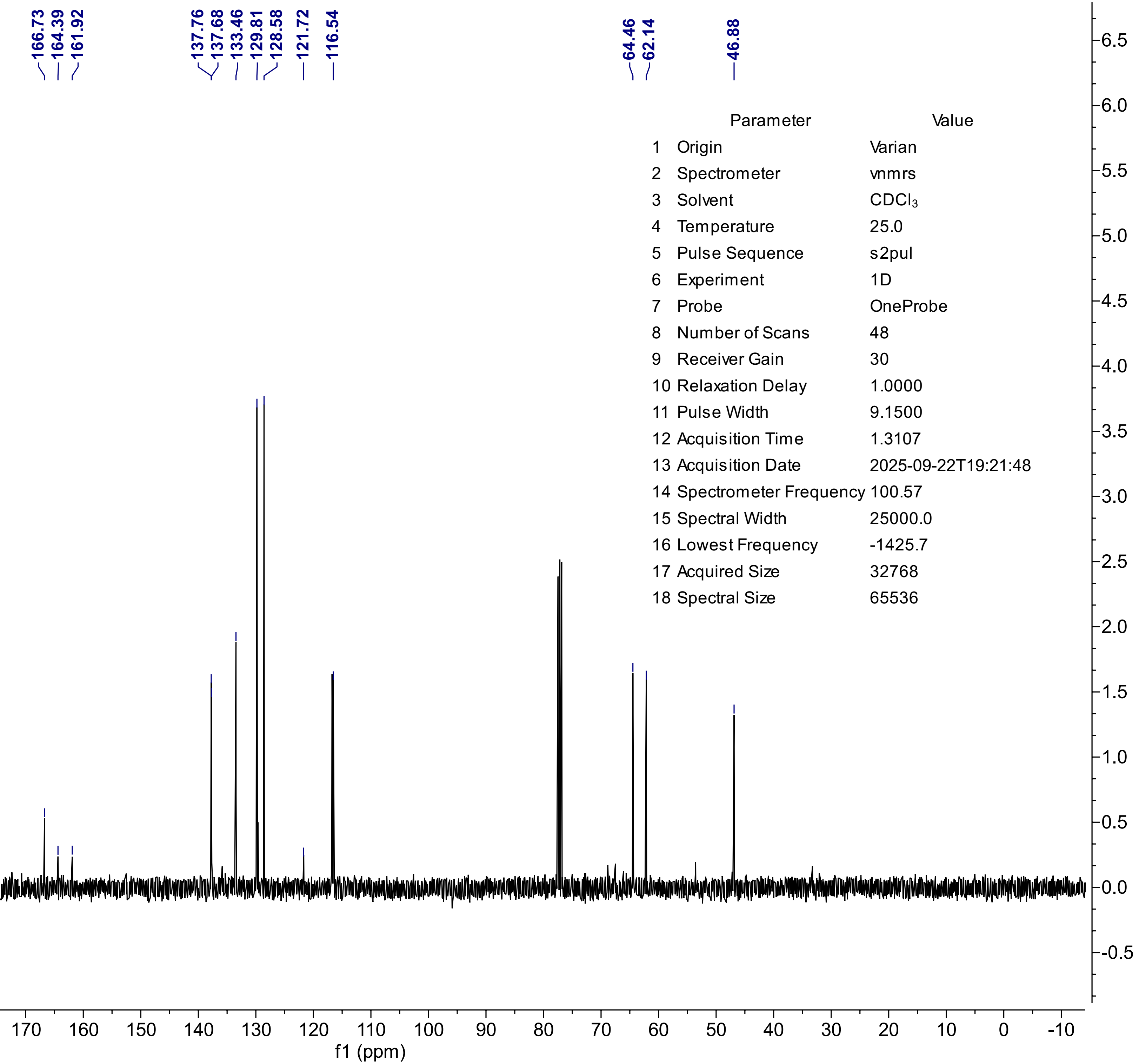

	Parameter	Value
1	Origin	Varian
2	Spectrometer	vnmrs
3	Solvent	$CDCl_3$
4	Temperature	25.0
5	Pulse Sequence	s2pul
6	Experiment	1D
7	Probe	OneProbe
8	Number of Scans	48
9	Receiver Gain	30
10	Relaxation Delay	1.0000
11	Pulse Width	9.1500
12	Acquisition Time	1.3107
13	Acquisition Date	2025-09-22T19:21:48
14	Spectrometer Frequency	100.57
15	Spectral Width	25000.0
16	Lowest Frequency	-1425.7
17	Acquired Size	32768
18	Spectral Size	65536

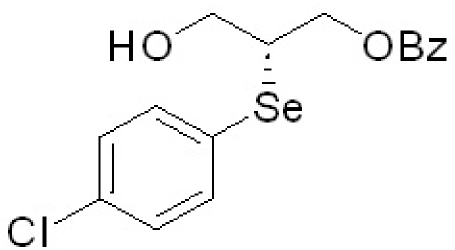

3ak

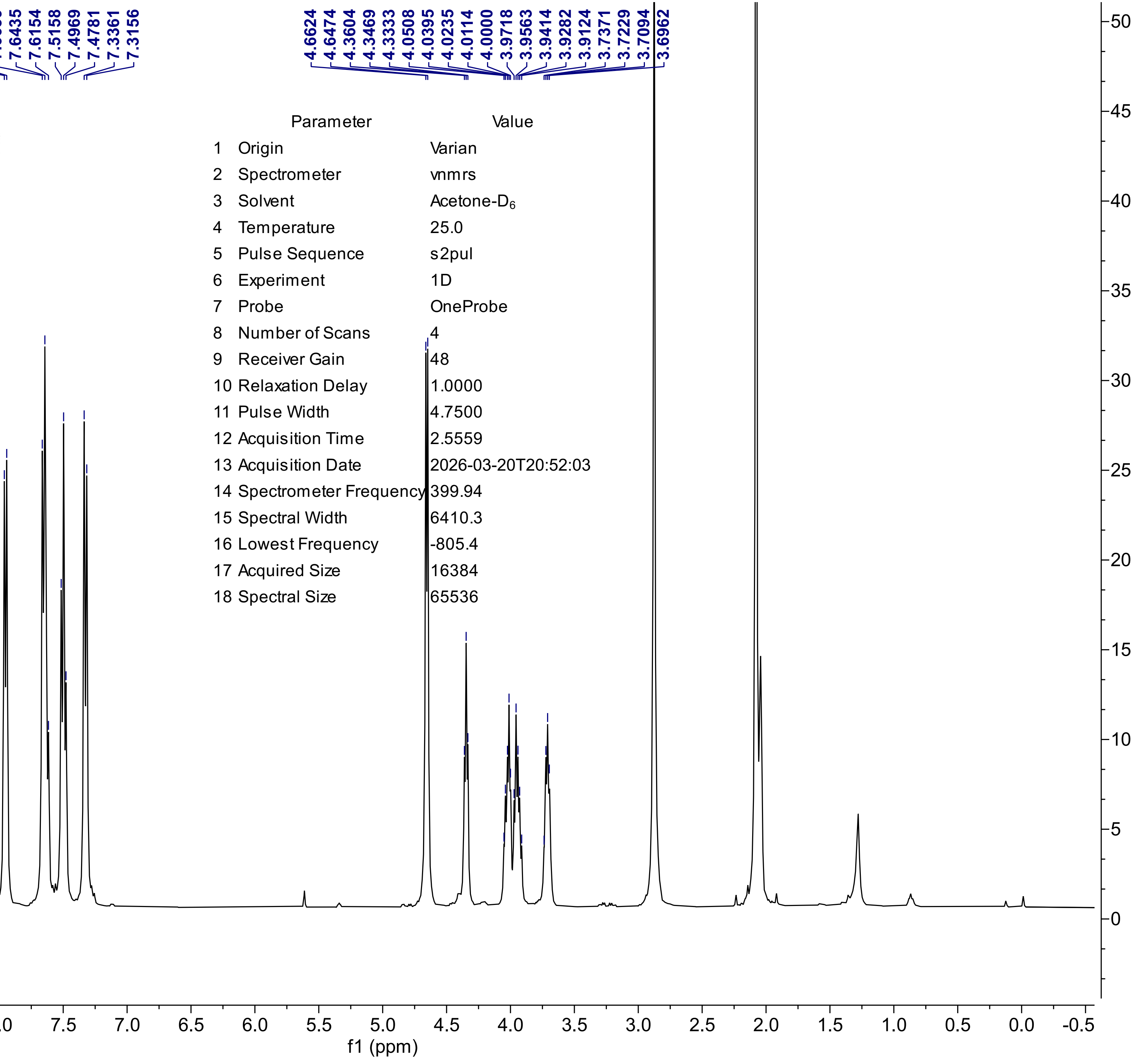

	Parameter	Value
1	Origin	Varian
2	Spectrometer	vnmrs
3	Solvent	Acetone-D_6
4	Temperature	25.0
5	Pulse Sequence	s2pul
6	Experiment	1D
7	Probe	OneProbe
8	Number of Scans	4
9	Receiver Gain	48
10	Relaxation Delay	1.0000
11	Pulse Width	4.7500
12	Acquisition Time	2.5559
13	Acquisition Date	2026-03-20T20:52:03
14	Spectrometer Frequency	399.94
15	Spectral Width	6410.3
16	Lowest Frequency	-805.4
17	Acquired Size	16384
18	Spectral Size	65536

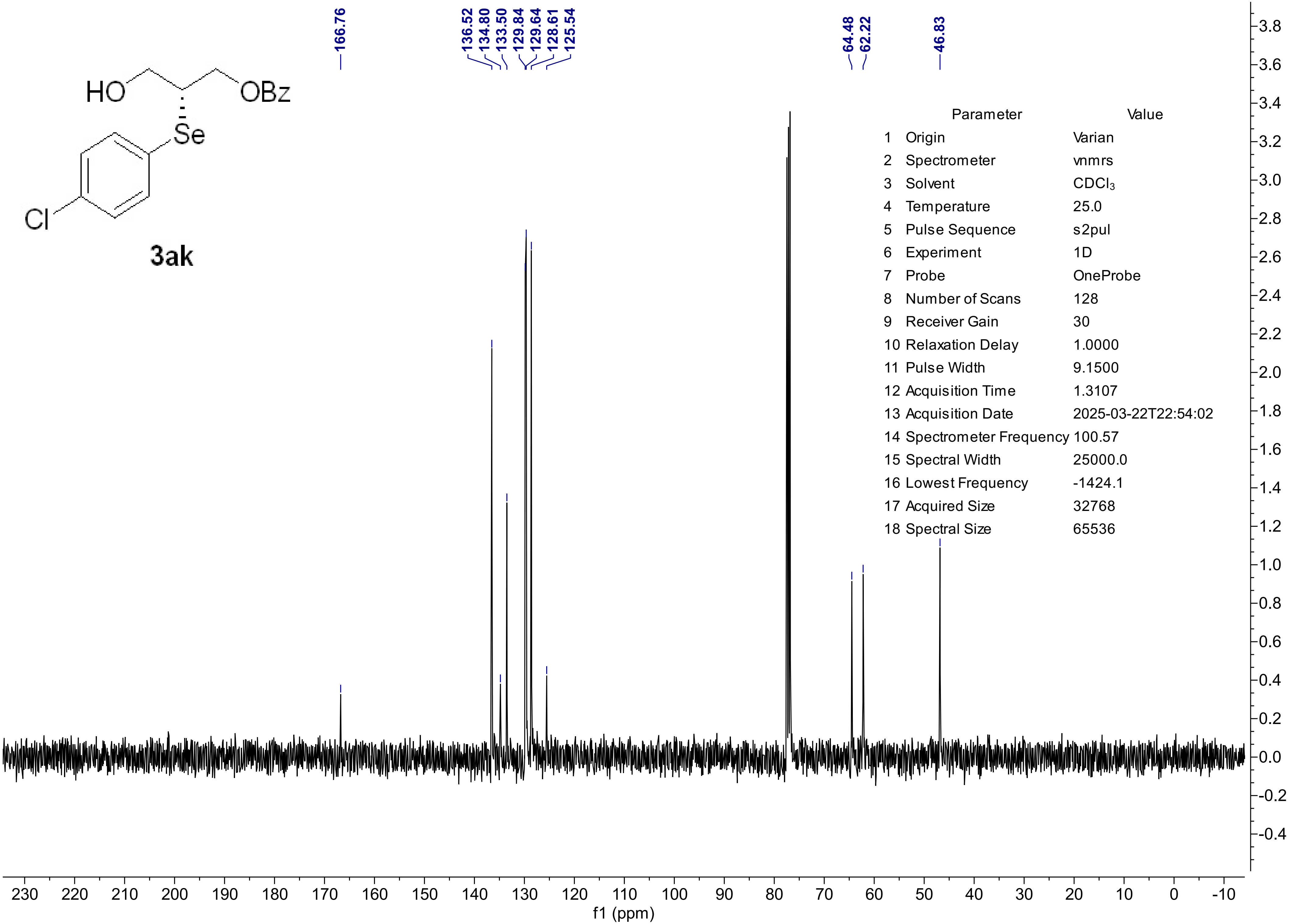

3ak

	Parameter	Value
1	Origin	Varian
2	Spectrometer	vnmrs
3	Solvent	$CDCl_3$
4	Temperature	25.0
5	Pulse Sequence	s2pul
6	Experiment	1D
7	Probe	OneProbe
8	Number of Scans	128
9	Receiver Gain	30
10	Relaxation Delay	1.0000
11	Pulse Width	9.1500
12	Acquisition Time	1.3107
13	Acquisition Date	2025-03-22T22:54:02
14	Spectrometer Frequency	100.57
15	Spectral Width	25000.0
16	Lowest Frequency	-1424.1
17	Acquired Size	32768
18	Spectral Size	65536

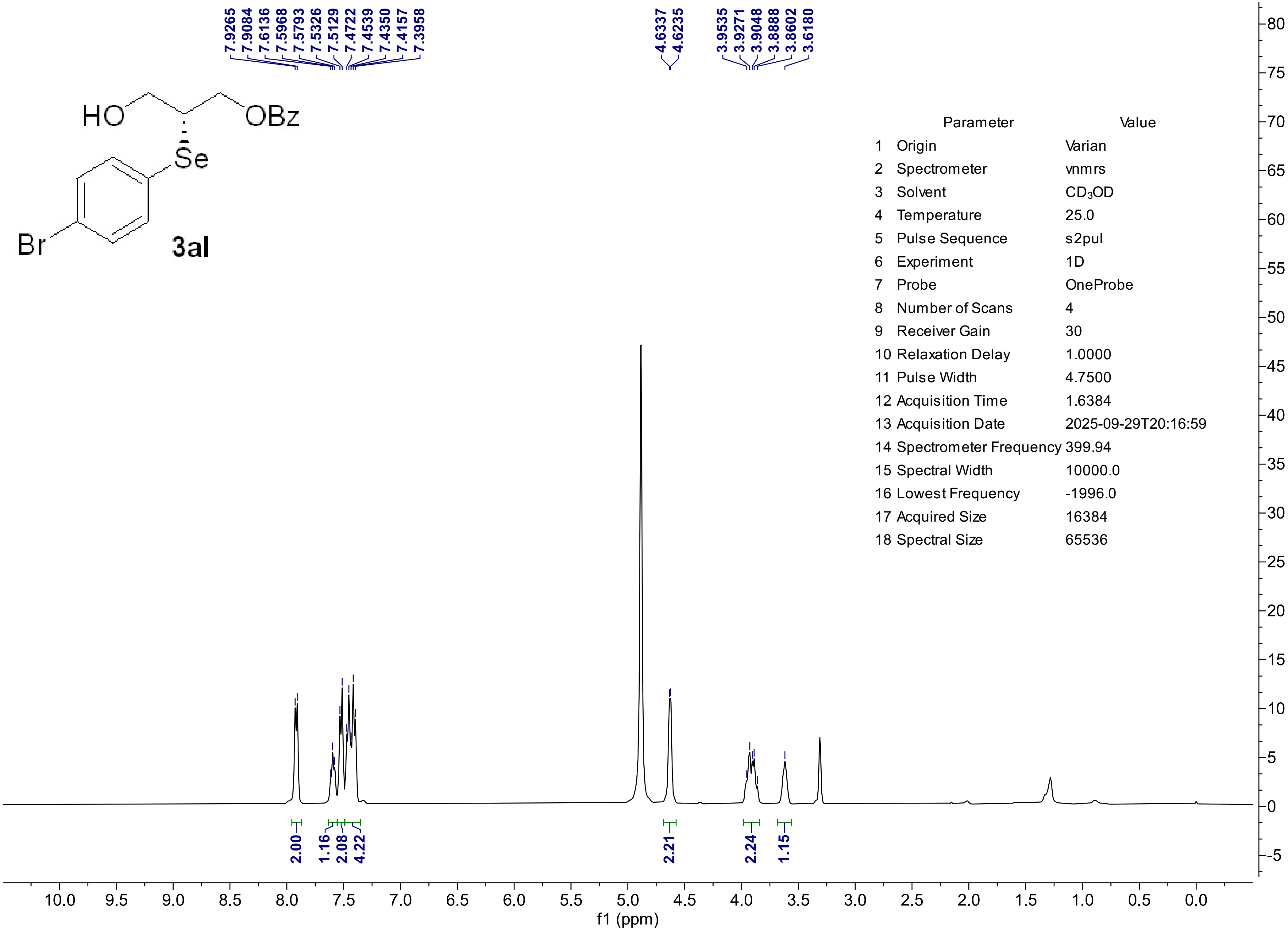

	Parameter	Value
1	Origin	Varian
2	Spectrometer	vnmrs
3	Solvent	CD_3OD
4	Temperature	25.0
5	Pulse Sequence	s2pul
6	Experiment	1D
7	Probe	OneProbe
8	Number of Scans	4
9	Receiver Gain	30
10	Relaxation Delay	1.0000
11	Pulse Width	4.7500
12	Acquisition Time	1.6384
13	Acquisition Date	2025-09-29T20:16:59
14	Spectrometer Frequency	399.94
15	Spectral Width	10000.0
16	Lowest Frequency	-1996.0
17	Acquired Size	16384
18	Spectral Size	65536

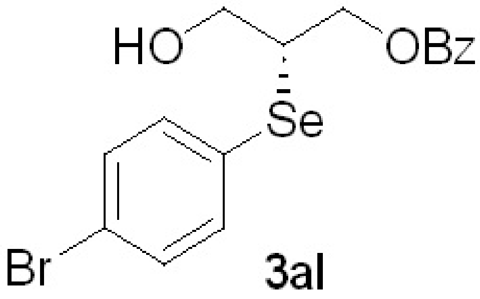

3al

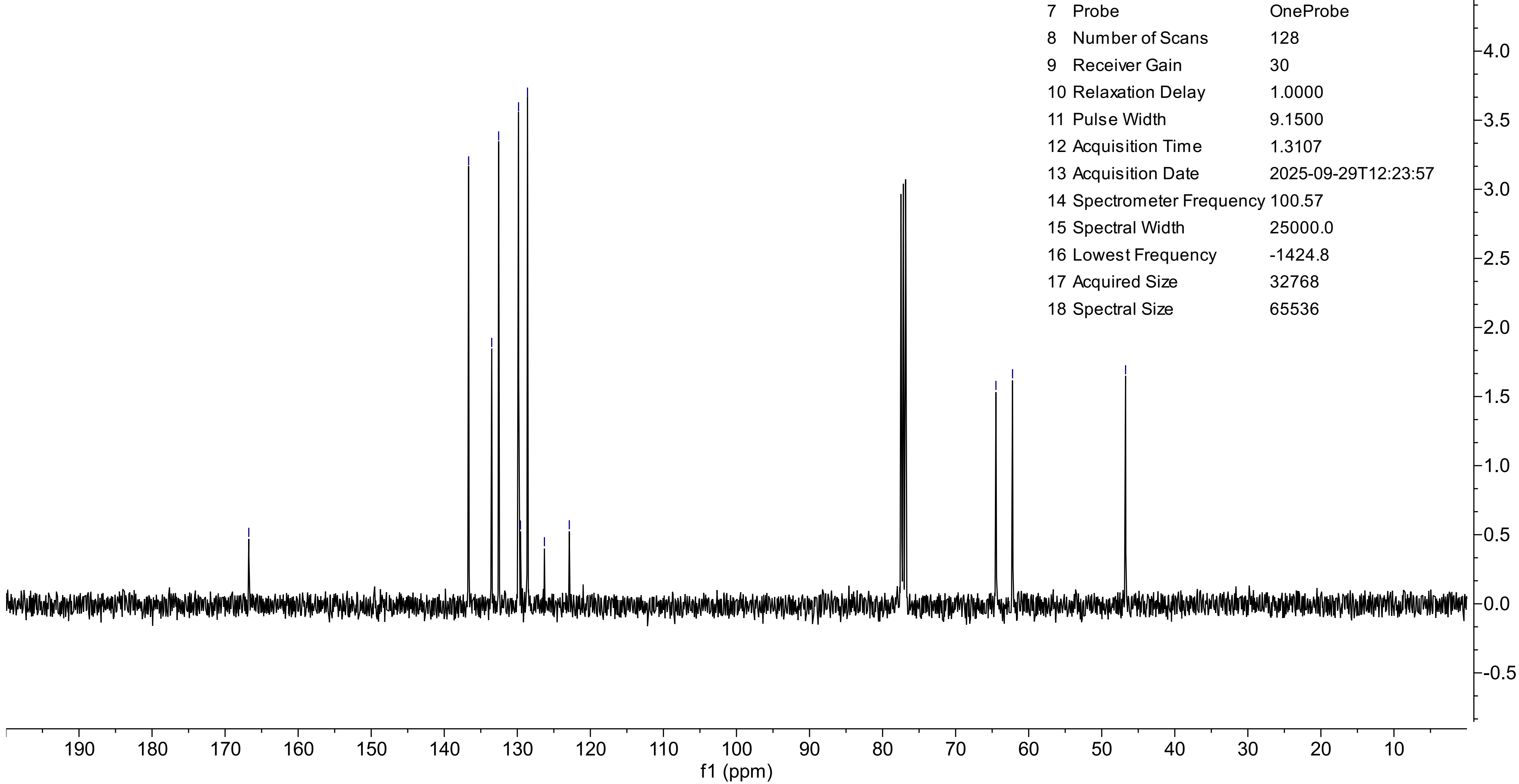

	Parameter	Value
1	Origin	Varian
2	Spectrometer	vnmrs
3	Solvent	$CDCl_3$
4	Temperature	25.0
5	Pulse Sequence	s2pul
6	Experiment	1D
7	Probe	OneProbe
8	Number of Scans	128
9	Receiver Gain	30
10	Relaxation Delay	1.0000
11	Pulse Width	9.1500
12	Acquisition Time	1.3107
13	Acquisition Date	2025-09-29T12:23:57
14	Spectrometer Frequency	100.57
15	Spectral Width	25000.0
16	Lowest Frequency	-1424.8
17	Acquired Size	32768
18	Spectral Size	65536

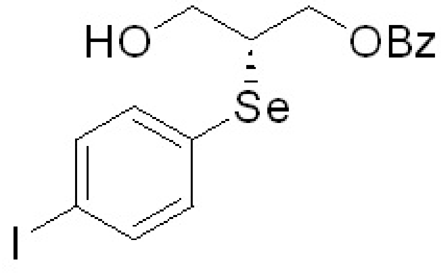

3am

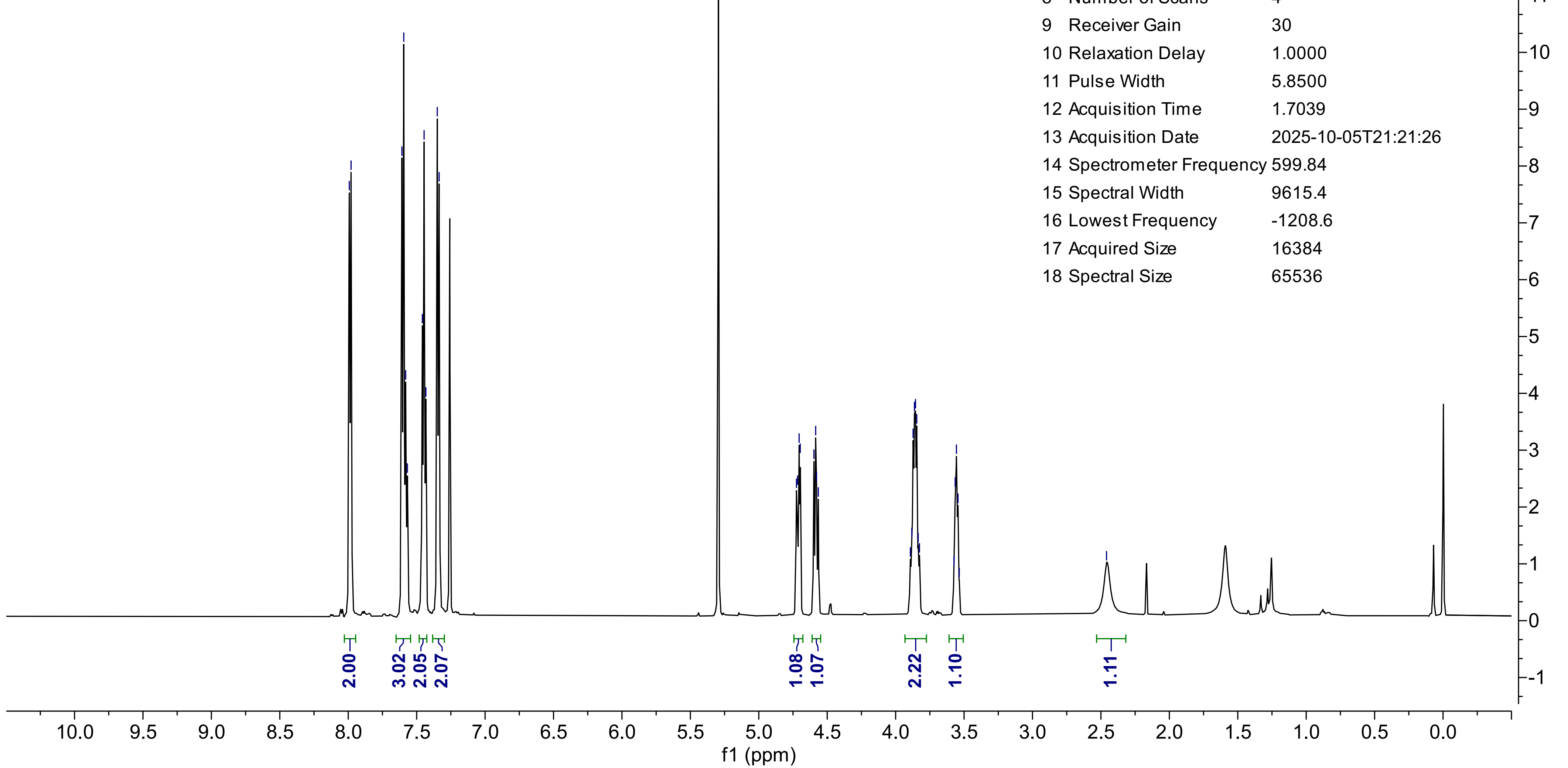

	Parameter	Value
1	Origin	Varian
2	Spectrometer	vnmrs
3	Solvent	$CDCl_3$
4	Temperature	25.0
5	Pulse Sequence	s2pul
6	Experiment	1D
7	Probe	OneP
8	Number of Scans	4
9	Receiver Gain	30
10	Relaxation Delay	1.0000
11	Pulse Width	5.8500
12	Acquisition Time	1.7039
13	Acquisition Date	2025-10-05T21:21:26
14	Spectrometer Frequency	599.84
15	Spectral Width	9615.4
16	Lowest Frequency	-1208.6
17	Acquired Size	16384
18	Spectral Size	65536

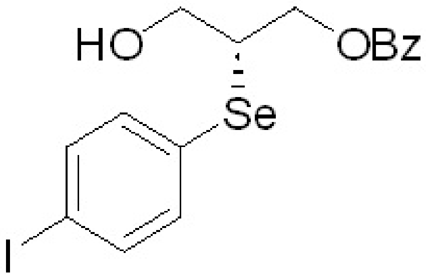

3am

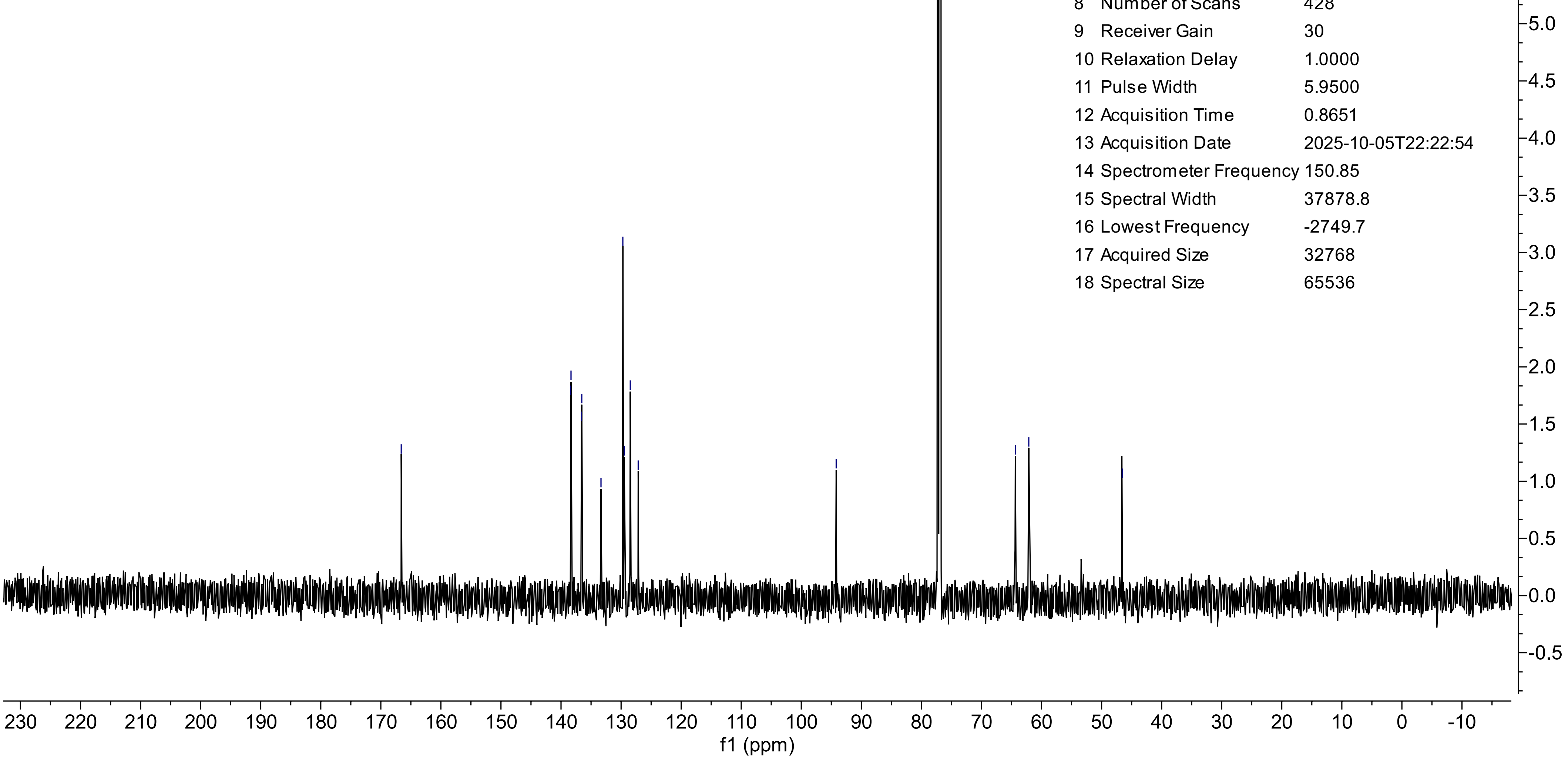

	Parameter	Value
1	Origin	Varian
2	Spectrometer	vnmrs
3	Solvent	$CDCl_3$
4	Temperature	25.0
5	Pulse Sequence	s2pul
6	Experiment	1D
7	Probe	OneP
8	Number of Scans	428
9	Receiver Gain	30
10	Relaxation Delay	1.0000
11	Pulse Width	5.9500
12	Acquisition Time	0.8651
13	Acquisition Date	2025-10-05T22:22:54
14	Spectrometer Frequency	150.85
15	Spectral Width	37878.8
16	Lowest Frequency	-2749.7
17	Acquired Size	32768
18	Spectral Size	65536

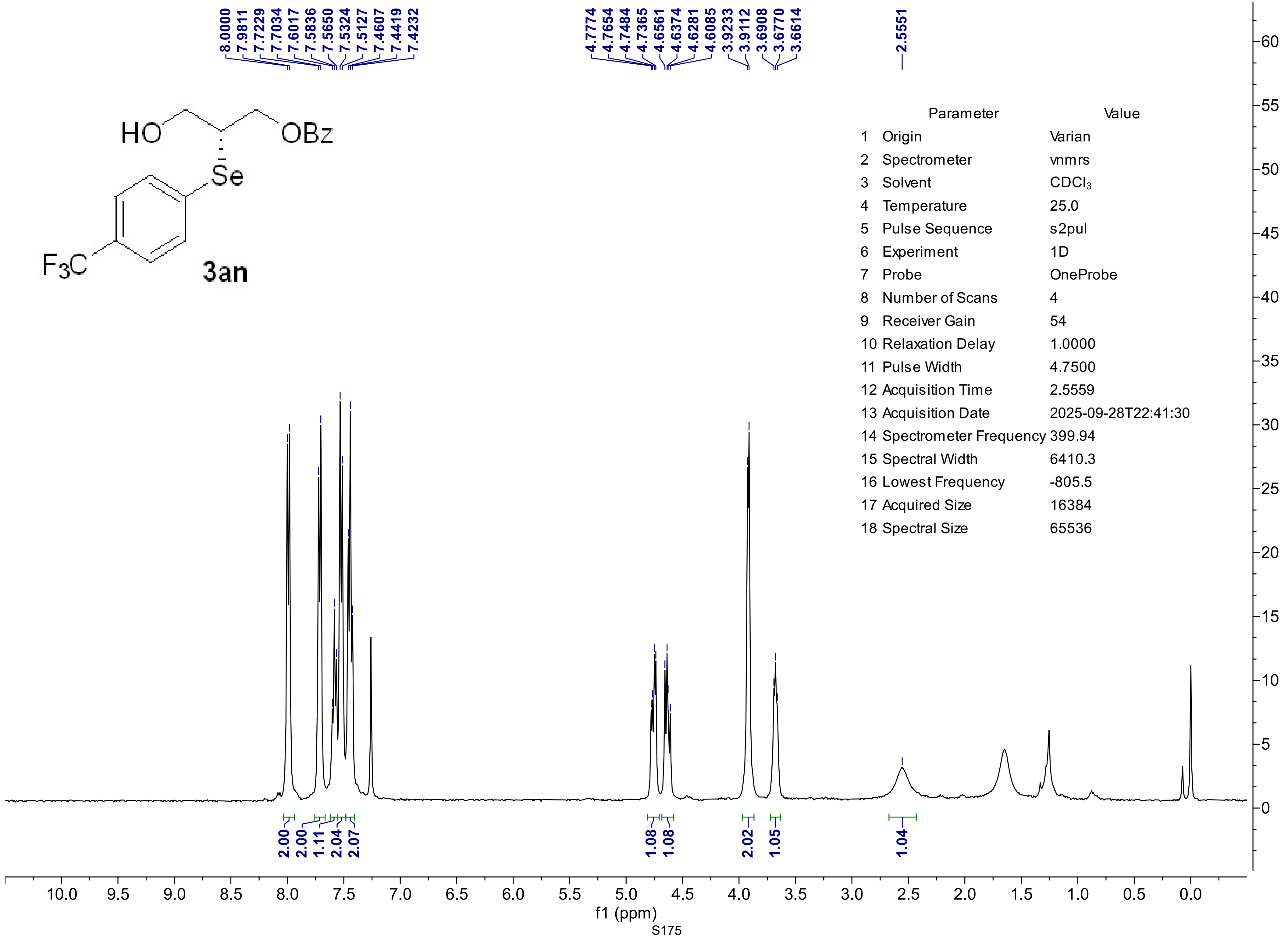

	Parameter	Value
1	Origin	Varian
2	Spectrometer	vnmrs
3	Solvent	$CDCl_3$
4	Temperature	25.0
5	Pulse Sequence	s2pul
6	Experiment	1D
7	Probe	OneProbe
8	Number of Scans	4
9	Receiver Gain	54
10	Relaxation Delay	1.0000
11	Pulse Width	4.7500
12	Acquisition Time	2.5559
13	Acquisition Date	2025-09-28T22:41:30
14	Spectrometer Frequency	399.94
15	Spectral Width	6410.3
16	Lowest Frequency	-805.5
17	Acquired Size	16384
18	Spectral Size	65536

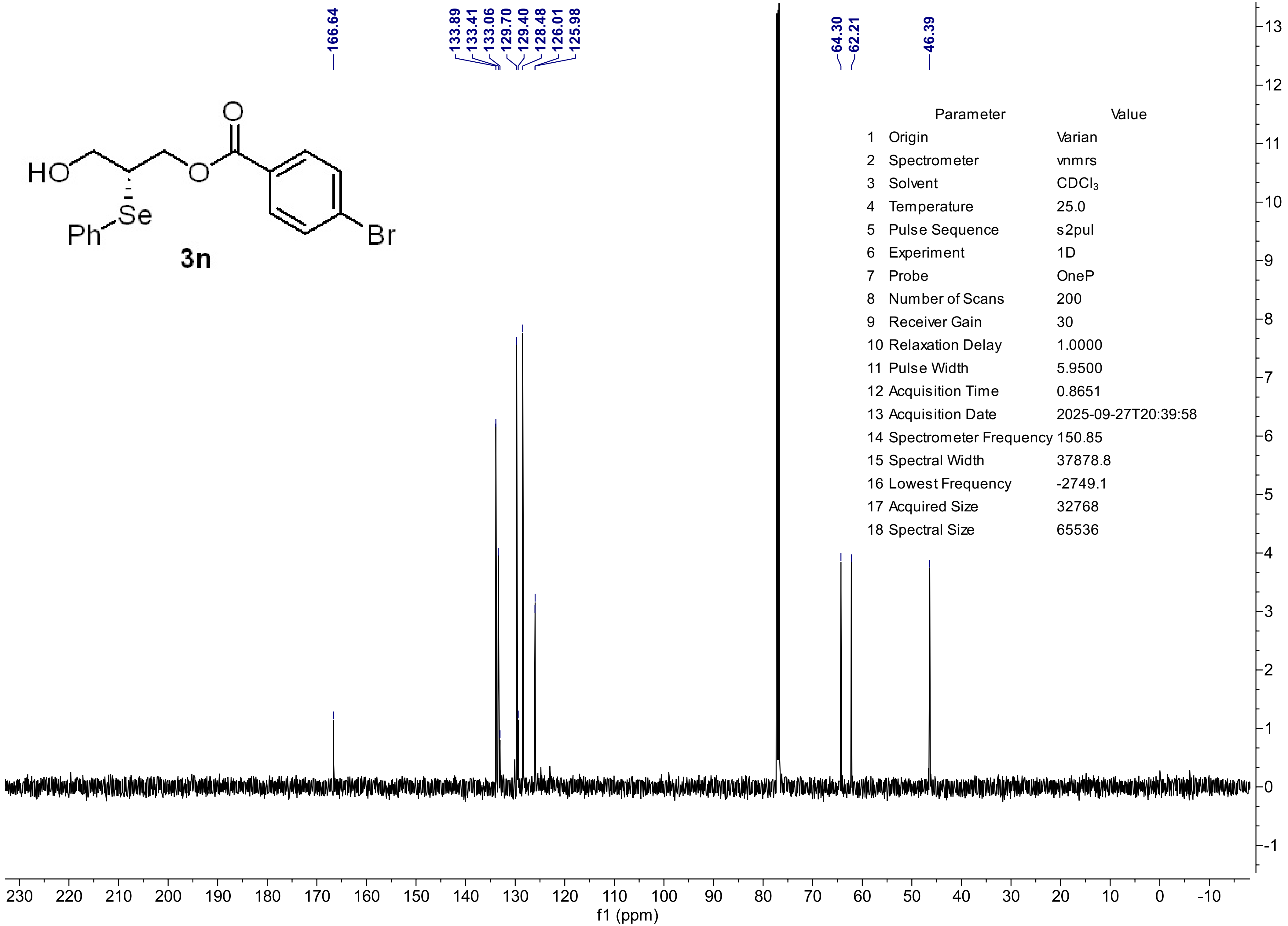
166.64
133.89
133.41
133.06
129.70
129.40
128.48
126.01
125.98
64.30
62.21
46.39
HO
O
O
Se
Ph
Br
3n
Parameter Value
1 Origin Varian
2 Spectrometer vnmrs
3 Solvent CDCl3
4 Temperature 25.0
5 Pulse Sequence s2pul
6 Experiment 1D
7 Probe OneP
8 Number of Scans 200
9 Receiver Gain 30
10 Relaxation Delay 1.0000
11 Pulse Width 5.9500
12 Acquisition Time 0.8651
13 Acquisition Date 2025-09-27T20:39:58
14 Spectrometer Frequency 150.85
15 Spectral Width 37878.8
16 Lowest Frequency -2749.1
17 Acquired Size 32768
18 Spectral Size 65536
f1 (ppm)

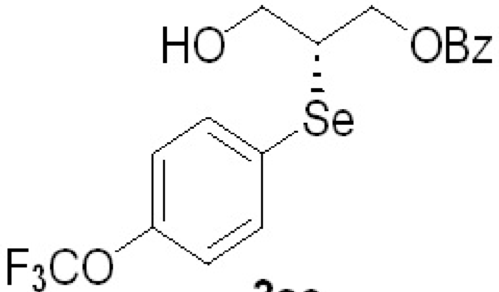

3ao

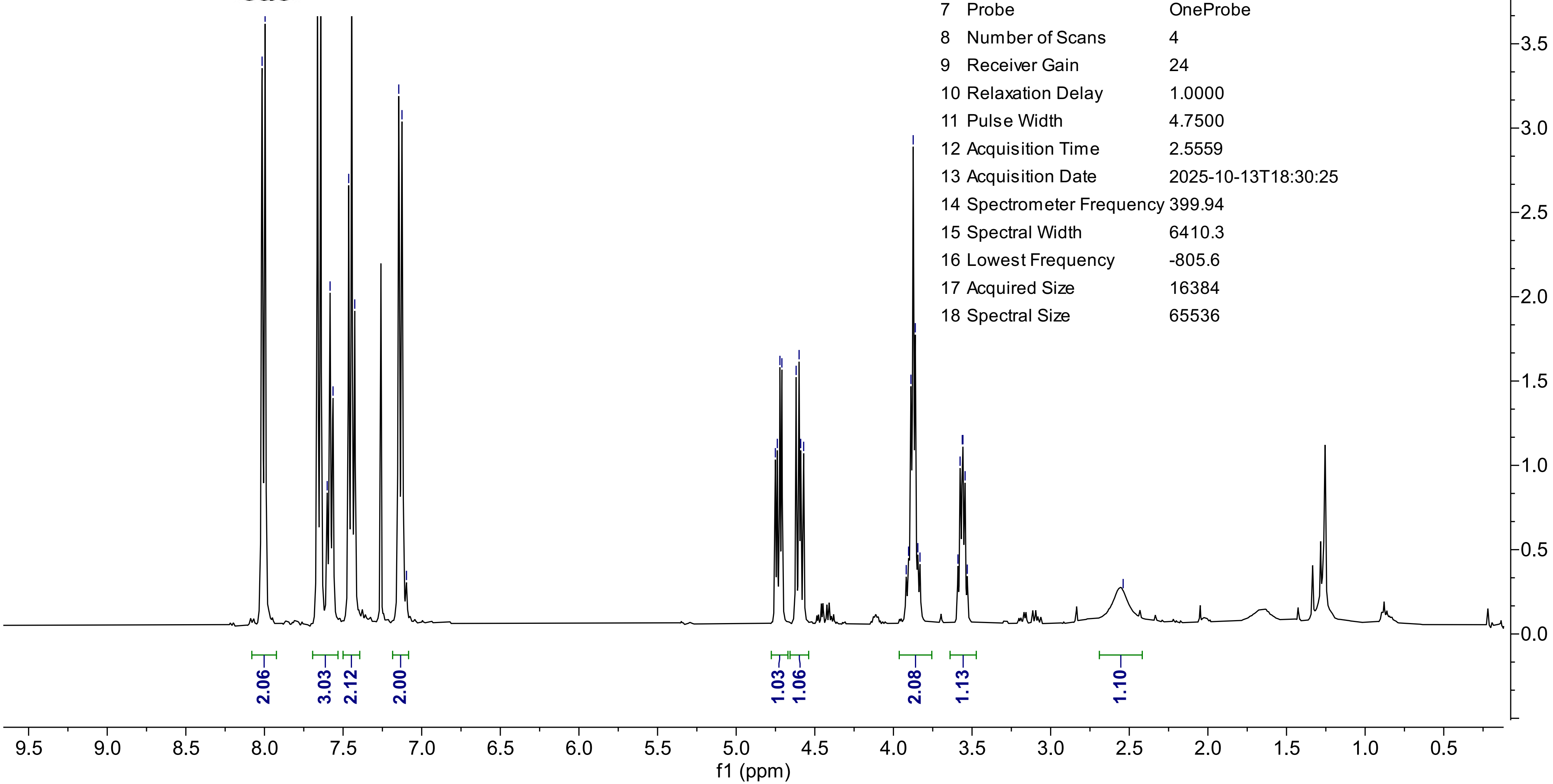

	Parameter	Value
1	Origin	Varian
2	Spectrometer	vnmrs
3	Solvent	$CDCl_3$
4	Temperature	25.0
5	Pulse Sequence	s2pul
6	Experiment	1D
7	Probe	OneProbe
8	Number of Scans	4
9	Receiver Gain	24
10	Relaxation Delay	1.0000
11	Pulse Width	4.7500
12	Acquisition Time	2.5559
13	Acquisition Date	2025-10-13T18:30:25
14	Spectrometer Frequency	399.94
15	Spectral Width	6410.3
16	Lowest Frequency	-805.6
17	Acquired Size	16384
18	Spectral Size	65536

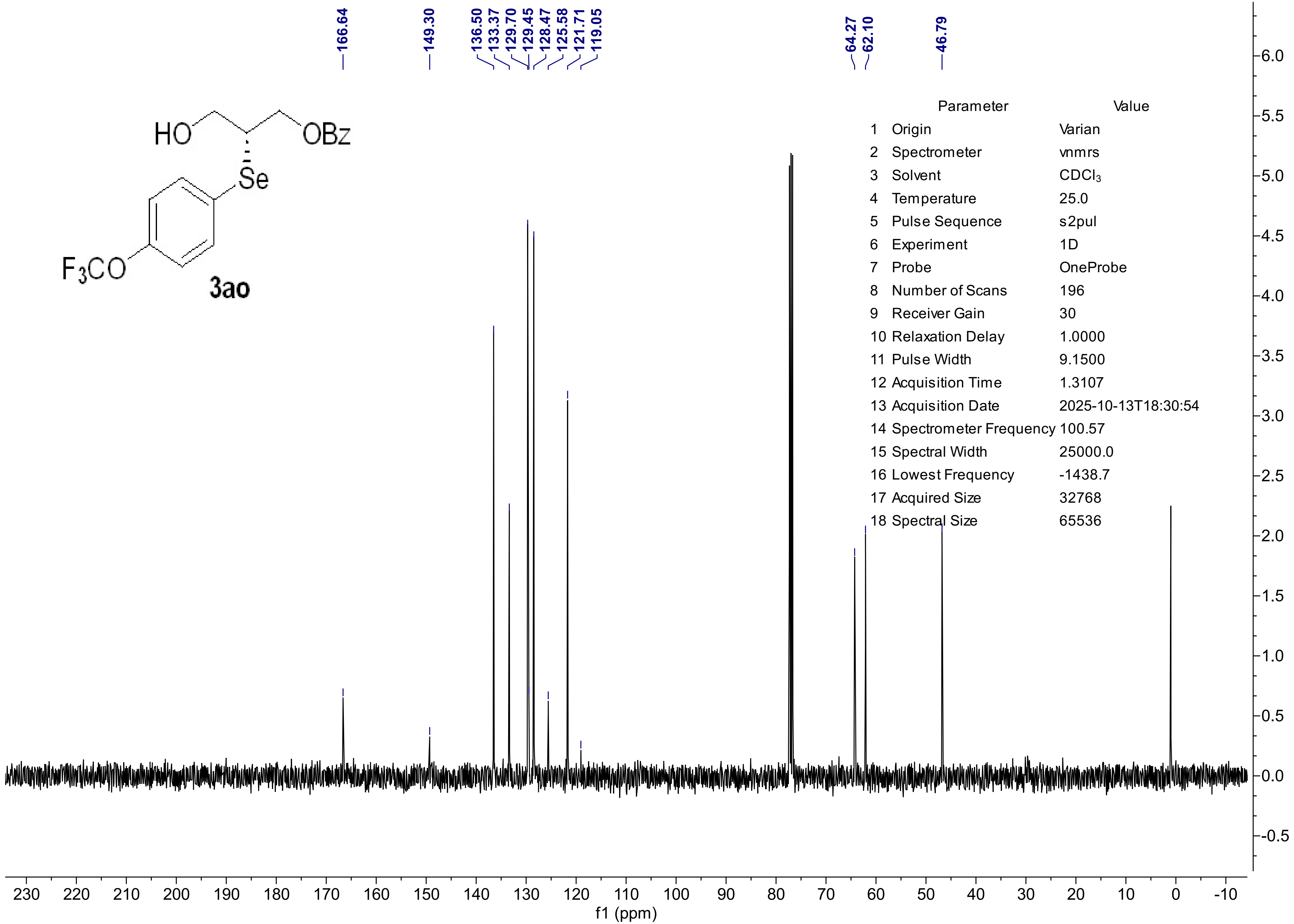
166.64
149.30
136.50
133.37
129.70
129.45
128.47
125.58
121.71
119.05
64.27
62.10
46.79
HO
OBz
Se
F3CO
3ao
Parameter Value
1 Origin Varian
2 Spectrometer vnmrs
3 Solvent CDCl3
4 Temperature 25.0
5 Pulse Sequence s2pul
6 Experiment 1D
7 Probe OneProbe
8 Number of Scans 196
9 Receiver Gain 30
10 Relaxation Delay 1.0000
11 Pulse Width 9.1500
12 Acquisition Time 1.3107
13 Acquisition Date 2025-10-13T18:30:54
14 Spectrometer Frequency 100.57
15 Spectral Width 25000.0
16 Lowest Frequency -1438.7
17 Acquired Size 32768
18 Spectral Size 65536
f1 (ppm)

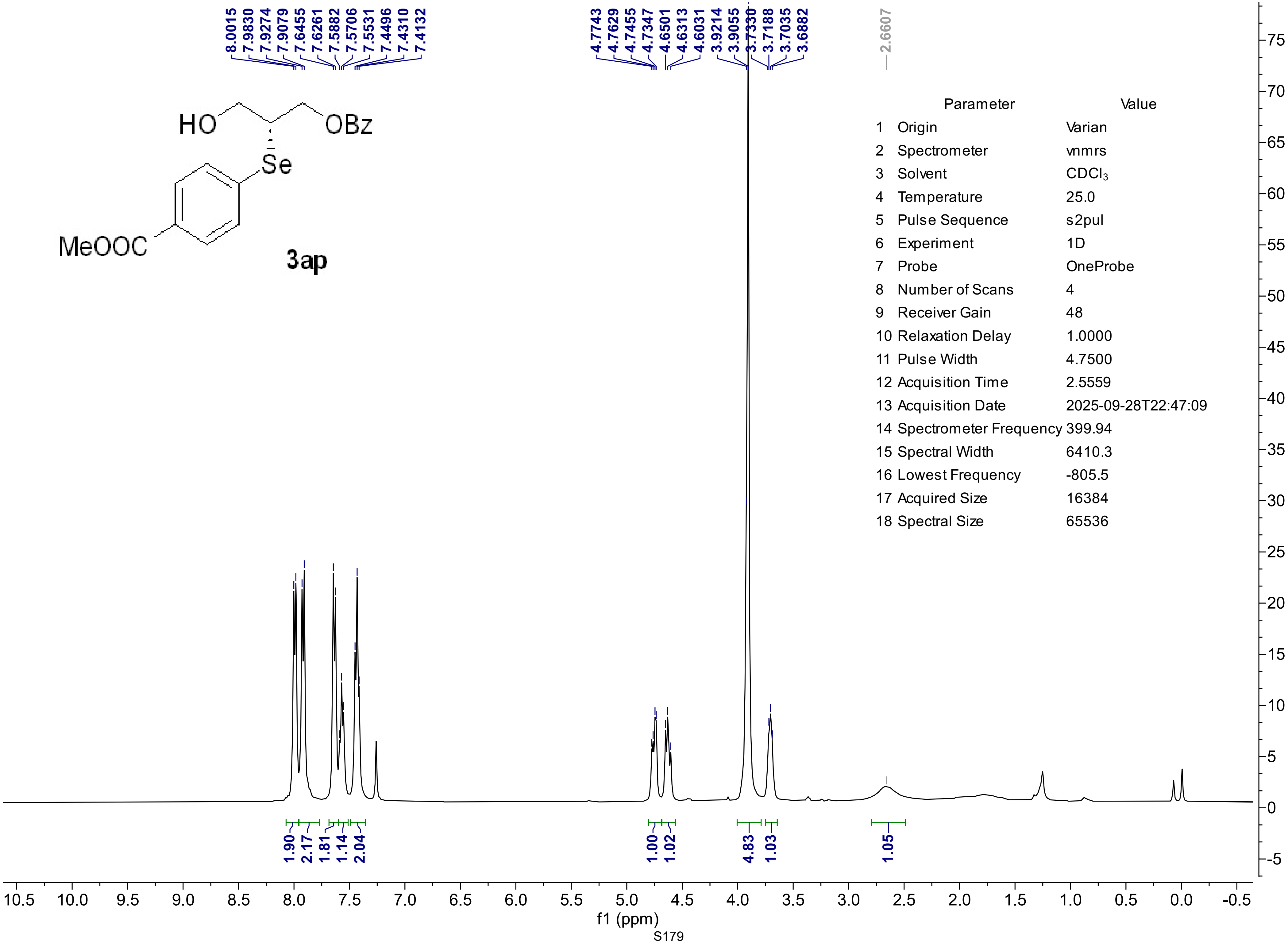

	Parameter	Value
1	Origin	Varian
2	Spectrometer	vnmrs
3	Solvent	$CDCl_3$
4	Temperature	25.0
5	Pulse Sequence	s2pul
6	Experiment	1D
7	Probe	OneProbe
8	Number of Scans	4
9	Receiver Gain	48
10	Relaxation Delay	1.0000
11	Pulse Width	4.7500
12	Acquisition Time	2.5559
13	Acquisition Date	2025-09-28T22:47:09
14	Spectrometer Frequency	399.94
15	Spectral Width	6410.3
16	Lowest Frequency	-805.5
17	Acquired Size	16384
18	Spectral Size	65536

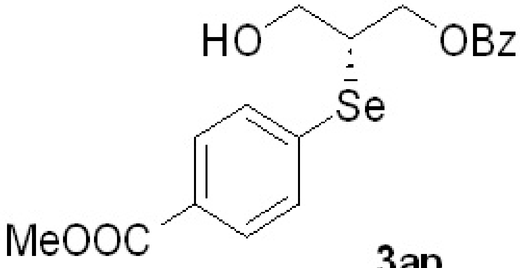

3ap

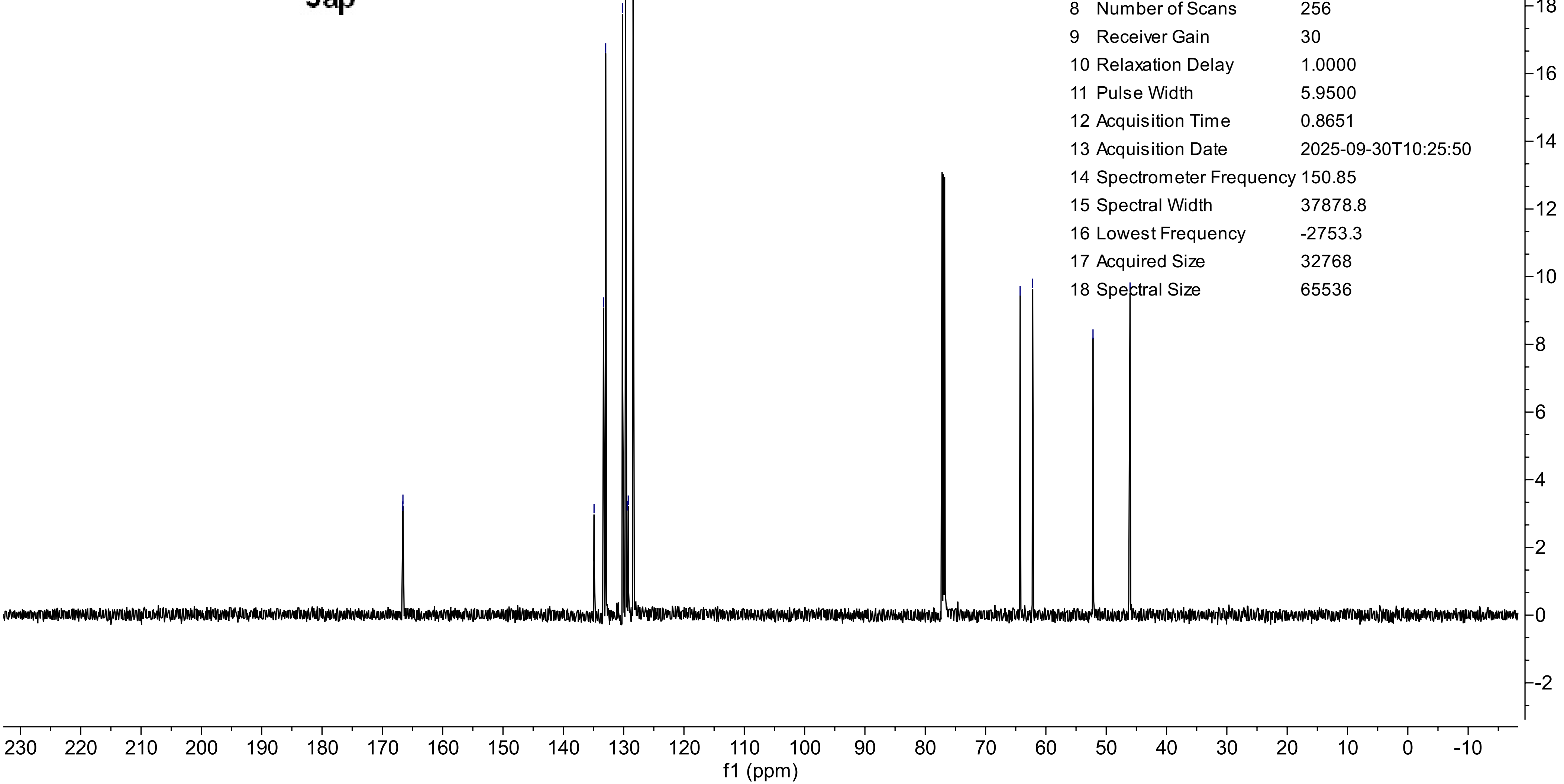

	Parameter	Value
1	Origin	Varian
2	Spectrometer	vnmrs
3	Solvent	$CDCl_3$
4	Temperature	25.0
5	Pulse Sequence	s2pul
6	Experiment	1D
7	Probe	OneP
8	Number of Scans	256
9	Receiver Gain	30
10	Relaxation Delay	1.0000
11	Pulse Width	5.9500
12	Acquisition Time	0.8651
13	Acquisition Date	2025-09-30T10:25:50
14	Spectrometer Frequency	150.85
15	Spectral Width	37878.8
16	Lowest Frequency	-2753.3
17	Acquired Size	32768
18	Spectral Size	65536

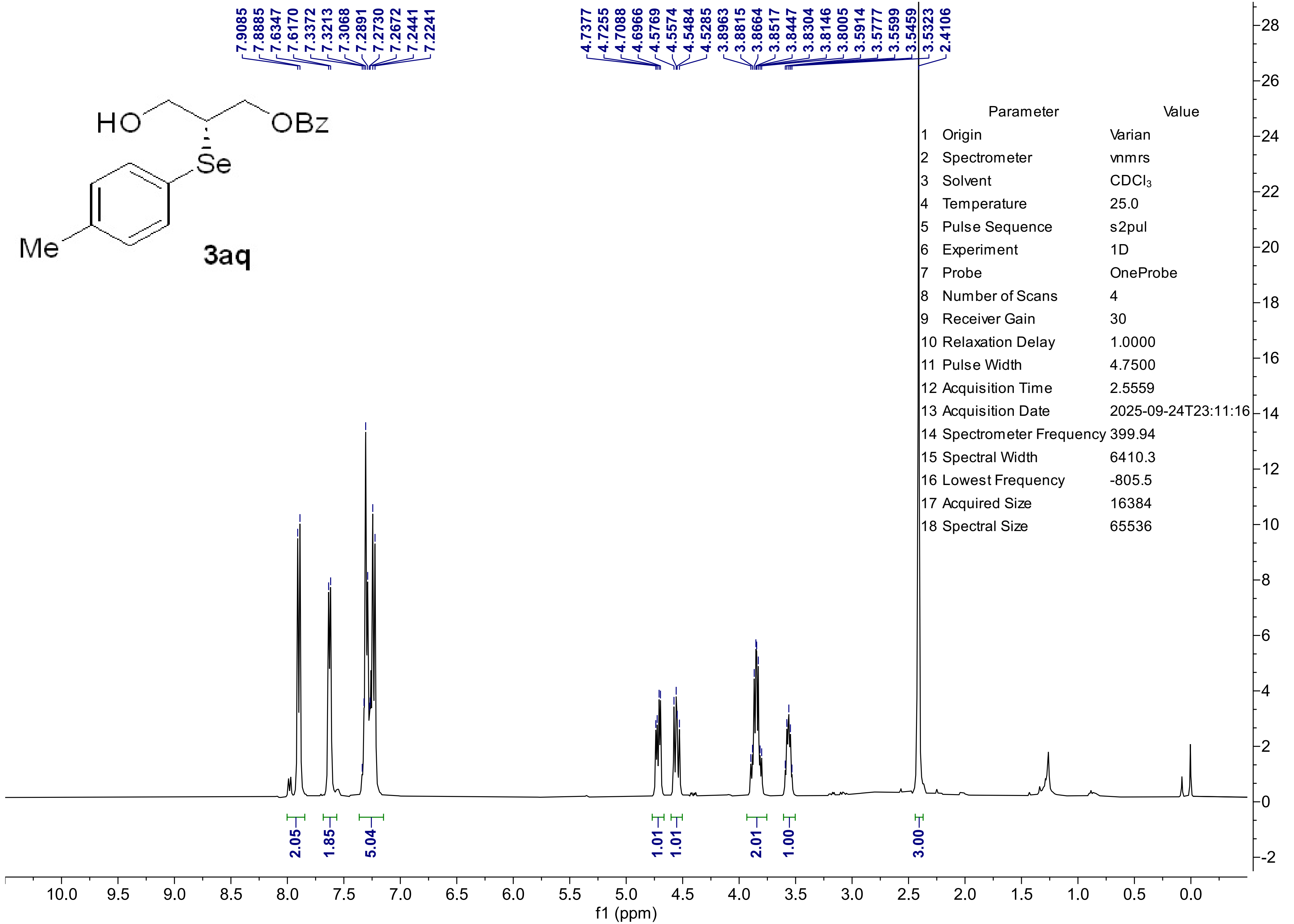
7.9085
7.8885
7.6347
7.6170
7.3372
7.3213
7.3068
7.2891
7.2730
7.2672
7.2441
7.2241
4.7377
4.7255
4.7088
4.6966
4.5769
4.5574
4.5484
4.5285
3.8963
3.8815
3.8664
3.8517
3.8447
3.8304
3.8146
3.8005
3.5914
3.5777
3.5599
3.5459
3.5323
2.4106
HO
OBz
Se
Me
3aq
Parameter
Value
1 Origin Varian
2 Spectrometer vnmrs
3 Solvent CDCl3
4 Temperature 25.0
5 Pulse Sequence s2pul
6 Experiment 1D
7 Probe OneProbe
8 Number of Scans 4
9 Receiver Gain 30
10 Relaxation Delay 1.0000
11 Pulse Width 4.7500
12 Acquisition Time 2.5559
13 Acquisition Date 2025-09-24T23:11:16
14 Spectrometer Frequency 399.94
15 Spectral Width 6410.3
16 Lowest Frequency -805.5
17 Acquired Size 16384
18 Spectral Size 65536
2.05
1.85
5.04
1.01
1.01
2.01
1.00
3.00
f1 (ppm)

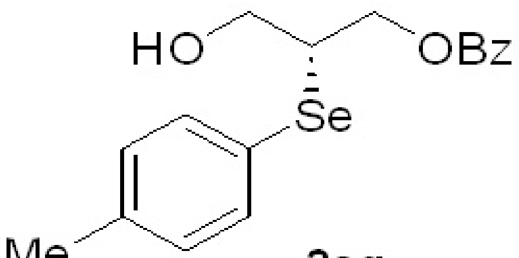

3aq

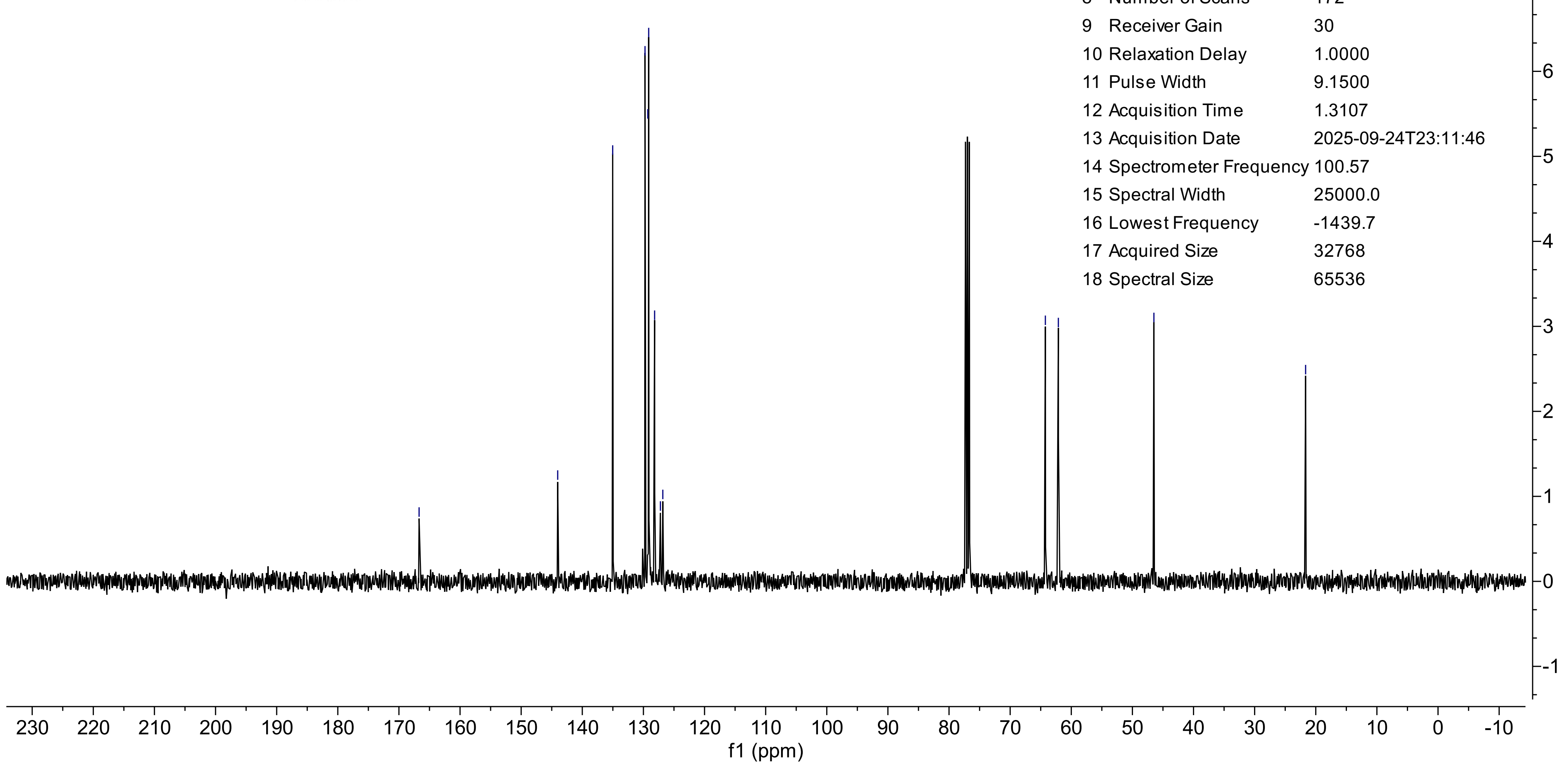

	Parameter	Value
1	Origin	Varian
2	Spectrometer	vnmrs
3	Solvent	$CDCl_3$
4	Temperature	25.0
5	Pulse Sequence	s2pul
6	Experiment	1D
7	Probe	OneProbe
8	Number of Scans	172
9	Receiver Gain	30
10	Relaxation Delay	1.0000
11	Pulse Width	9.1500
12	Acquisition Time	1.3107
13	Acquisition Date	2025-09-24T23:11:46
14	Spectrometer Frequency	100.57
15	Spectral Width	25000.0
16	Lowest Frequency	-1439.7
17	Acquired Size	32768
18	Spectral Size	65536

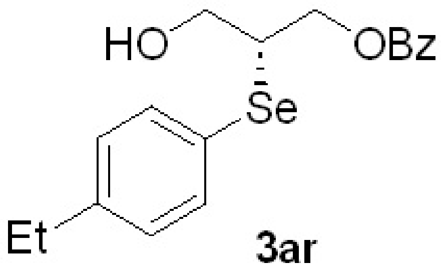

3ar

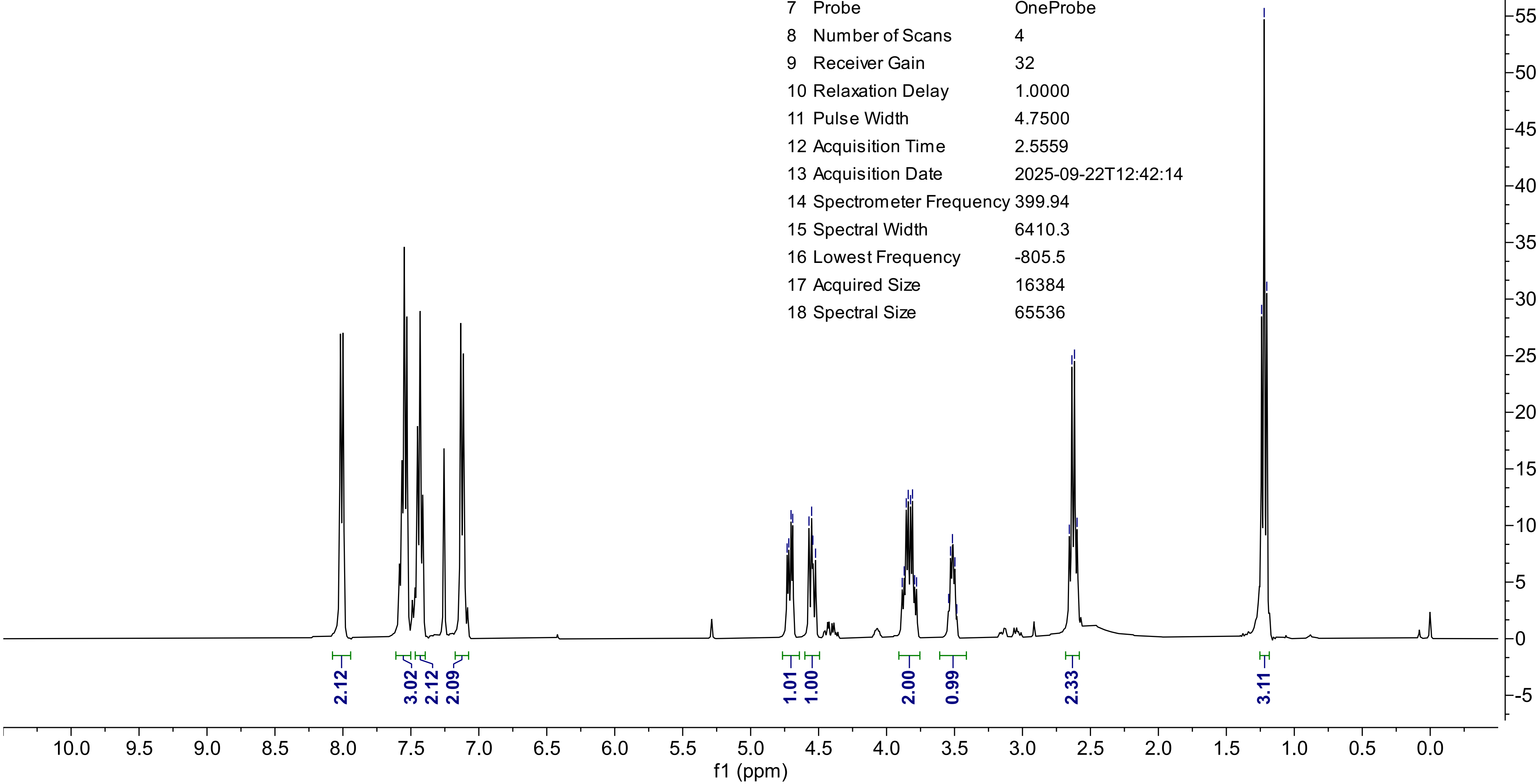

	Parameter	Value
1	Origin	Varian
2	Spectrometer	vnmrs
3	Solvent	$CDCl_3$
4	Temperature	25.0
5	Pulse Sequence	s2pul
6	Experiment	1D
7	Probe	OneProbe
8	Number of Scans	4
9	Receiver Gain	32
10	Relaxation Delay	1.0000
11	Pulse Width	4.7500
12	Acquisition Time	2.5559
13	Acquisition Date	2025-09-22T12:42:14
14	Spectrometer Frequency	399.94
15	Spectral Width	6410.3
16	Lowest Frequency	-805.5
17	Acquired Size	16384
18	Spectral Size	65536

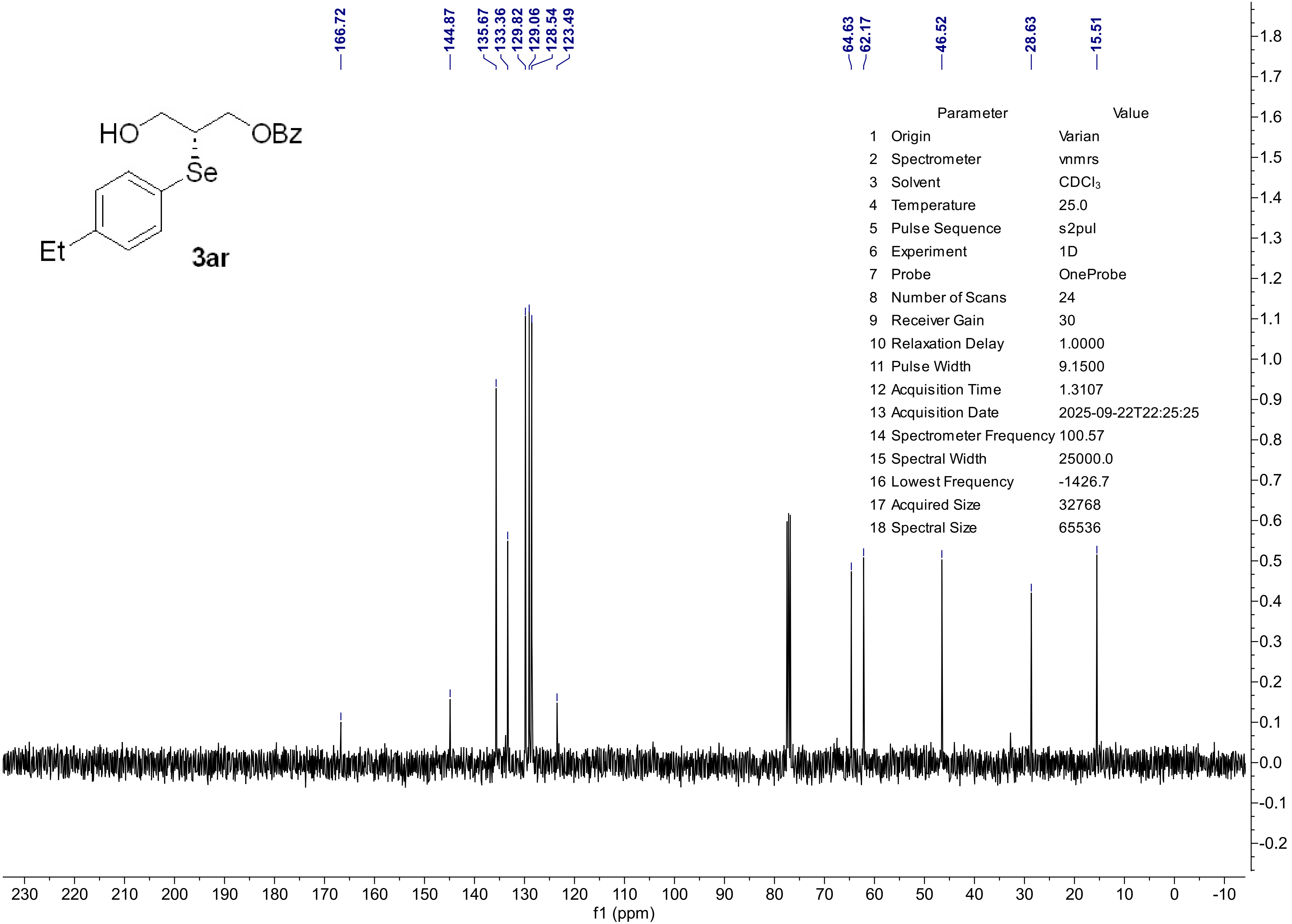

	Parameter	Value
1	Origin	Varian
2	Spectrometer	vnmrs
3	Solvent	$CDCl_3$
4	Temperature	25.0
5	Pulse Sequence	s2pul
6	Experiment	1D
7	Probe	OneProbe
8	Number of Scans	24
9	Receiver Gain	30
10	Relaxation Delay	1.0000
11	Pulse Width	9.1500
12	Acquisition Time	1.3107
13	Acquisition Date	2025-09-22T22:25:25
14	Spectrometer Frequency	100.57
15	Spectral Width	25000.0
16	Lowest Frequency	-1426.7
17	Acquired Size	32768
18	Spectral Size	65536

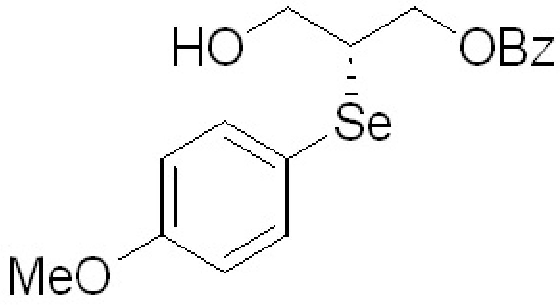

3as

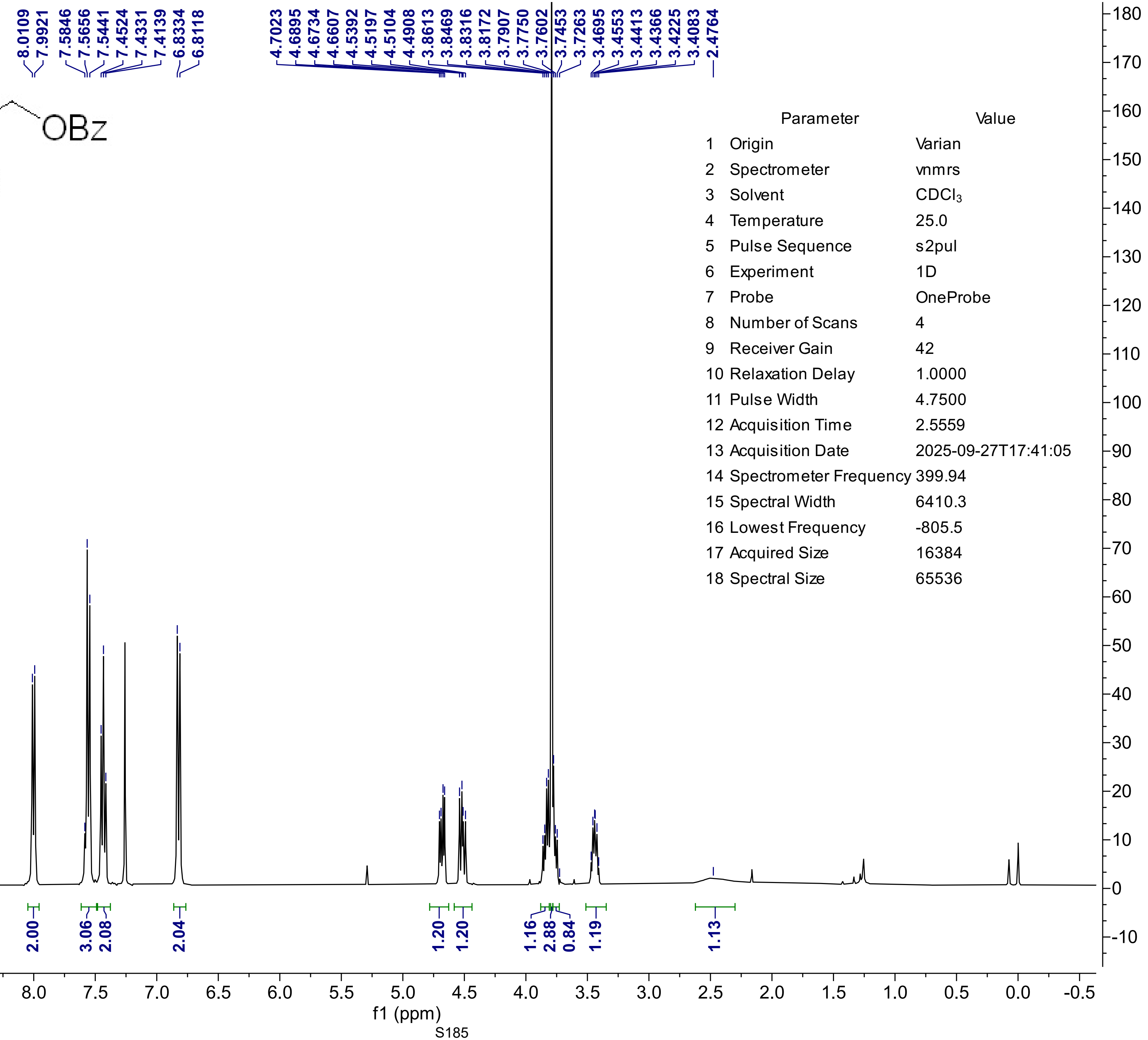

	Parameter	Value
1	Origin	Varian
2	Spectrometer	vnmrs
3	Solvent	$CDCl_3$
4	Temperature	25.0
5	Pulse Sequence	s2pul
6	Experiment	1D
7	Probe	OneProbe
8	Number of Scans	4
9	Receiver Gain	42
10	Relaxation Delay	1.0000
11	Pulse Width	4.7500
12	Acquisition Time	2.5559
13	Acquisition Date	2025-09-27T17:41:05
14	Spectrometer Frequency	399.94
15	Spectral Width	6410.3
16	Lowest Frequency	-805.5
17	Acquired Size	16384
18	Spectral Size	65536

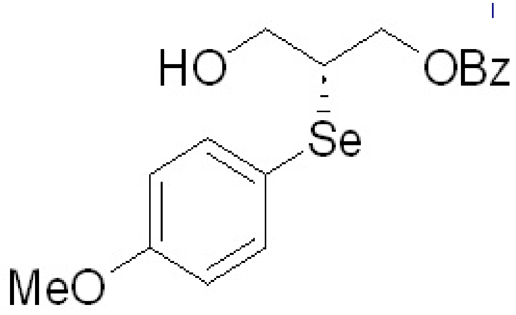

3as

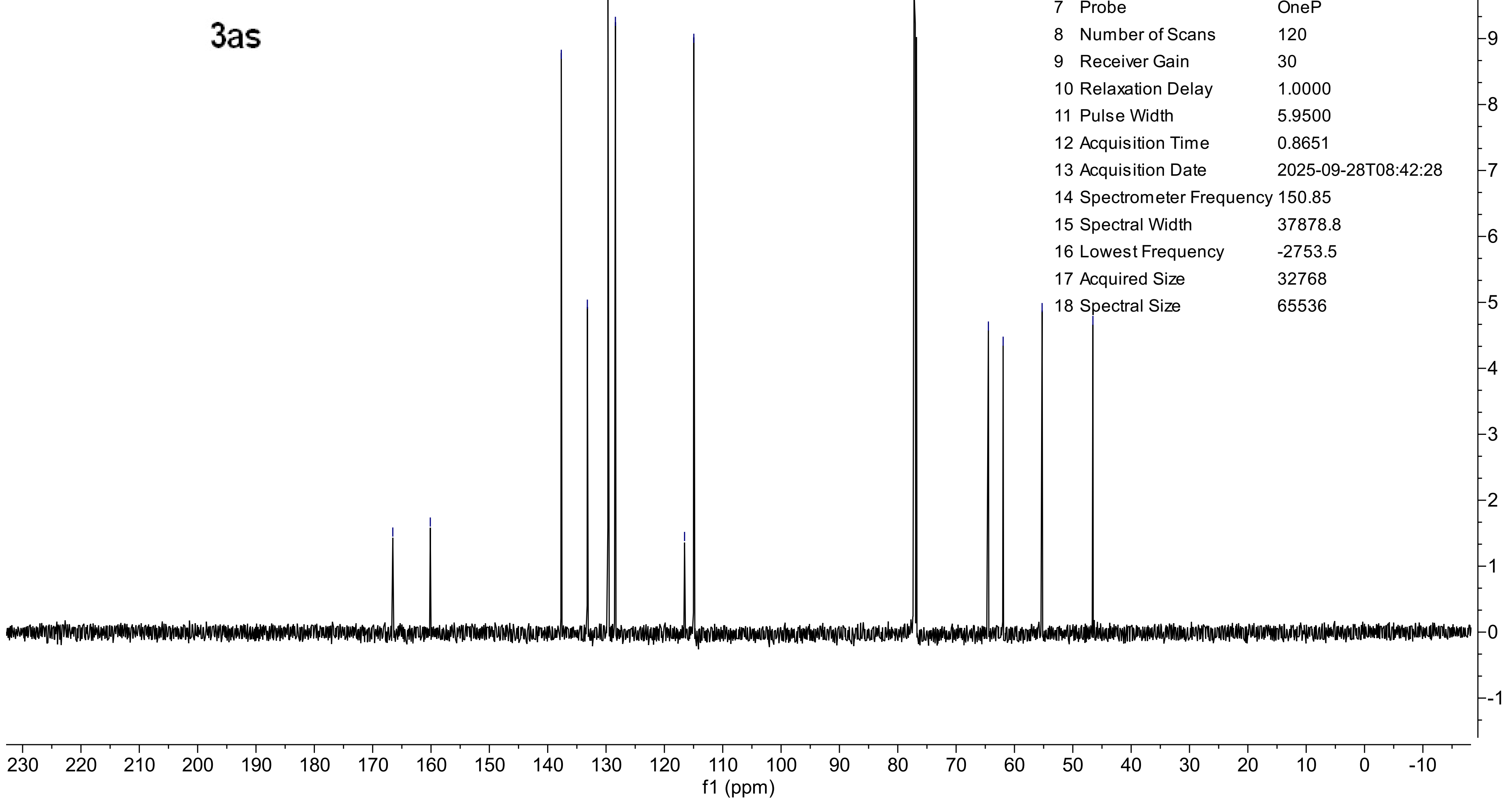

	Parameter	Value
1	Origin	Varian
2	Spectrometer	vnmrs
3	Solvent	$CDCl_3$
4	Temperature	25.0
5	Pulse Sequence	s2pul
6	Experiment	1D
7	Probe	OneP
8	Number of Scans	120
9	Receiver Gain	30
10	Relaxation Delay	1.0000
11	Pulse Width	5.9500
12	Acquisition Time	0.8651
13	Acquisition Date	2025-09-28T08:42:28
14	Spectrometer Frequency	150.85
15	Spectral Width	37878.8
16	Lowest Frequency	-2753.5
17	Acquired Size	32768
18	Spectral Size	65536

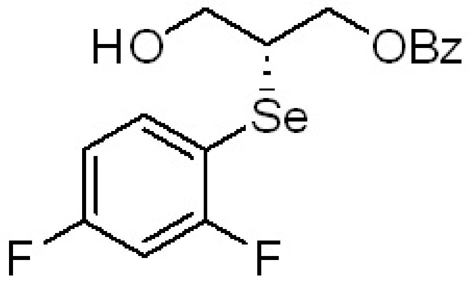

3at

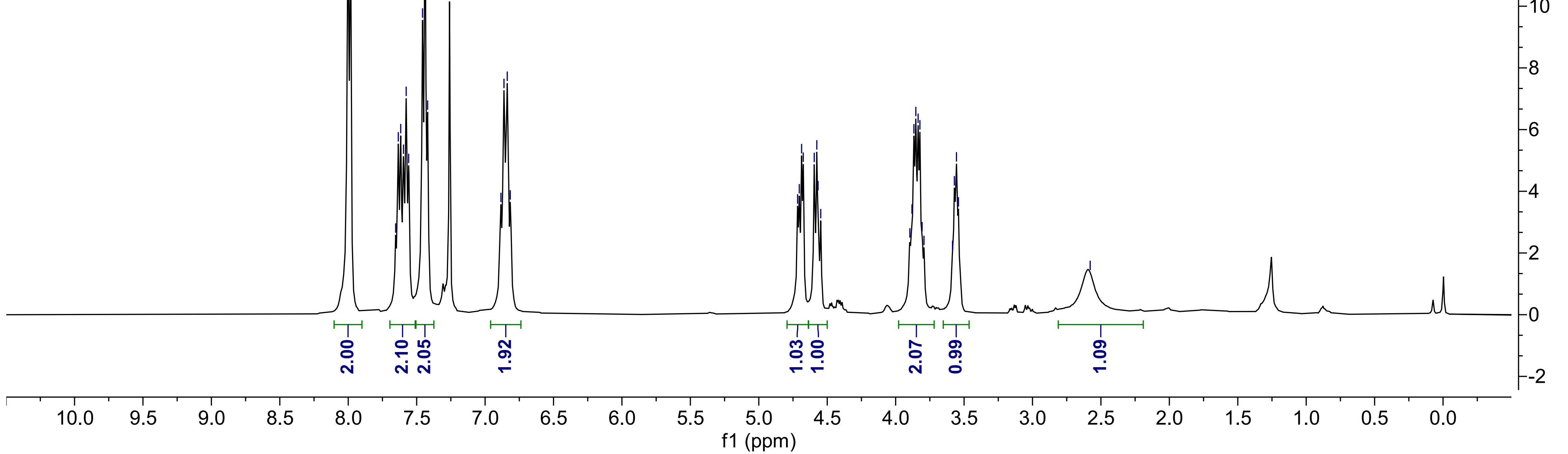

	Parameter	Value
1	Origin	Varian
2	Spectrometer	vnmrs
3	Solvent	$CDCl_3$
4	Temperature	25.0
5	Pulse Sequence	s2pul
6	Experiment	1D
7	Probe	OneProbe
8	Number of Scans	1
9	Receiver Gain	44
10	Relaxation Delay	1.0000
11	Pulse Width	4.7500
12	Acquisition Time	2.5559
13	Acquisition Date	2025-10-14T18:14:13
14	Spectrometer Frequency	399.94
15	Spectral Width	6410.3
16	Lowest Frequency	-805.5
17	Acquired Size	16384
18	Spectral Size	65536

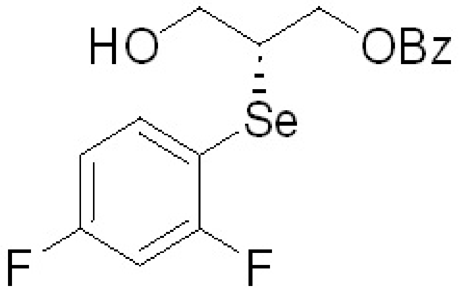

3at

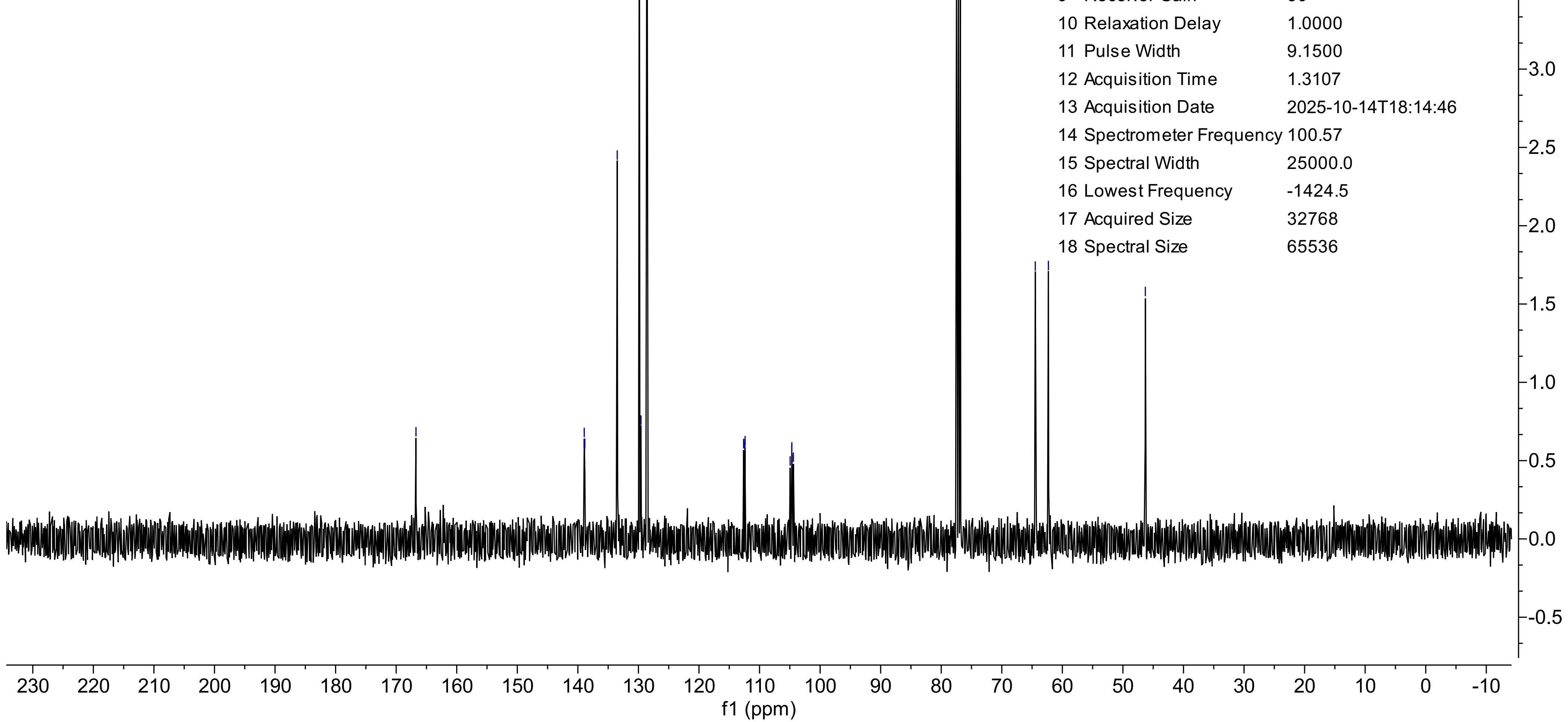

	Parameter	Value
1	Origin	Varian
2	Spectrometer	vnmrs
3	Solvent	$CDCl_3$
4	Temperature	25.0
5	Pulse Sequence	s2pul
6	Experiment	1D
7	Probe	OneProbe
8	Number of Scans	112
9	Receiver Gain	30
10	Relaxation Delay	1.0000
11	Pulse Width	9.1500
12	Acquisition Time	1.3107
13	Acquisition Date	2025-10-14T18:14:46
14	Spectrometer Frequency	100.57
15	Spectral Width	25000.0
16	Lowest Frequency	-1424.5
17	Acquired Size	32768
18	Spectral Size	65536

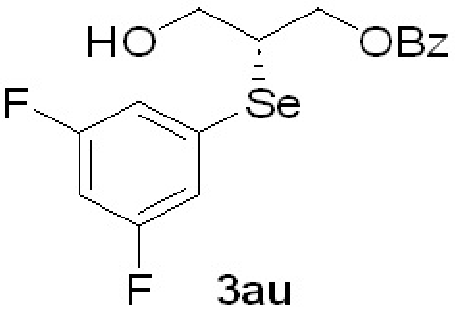

3au

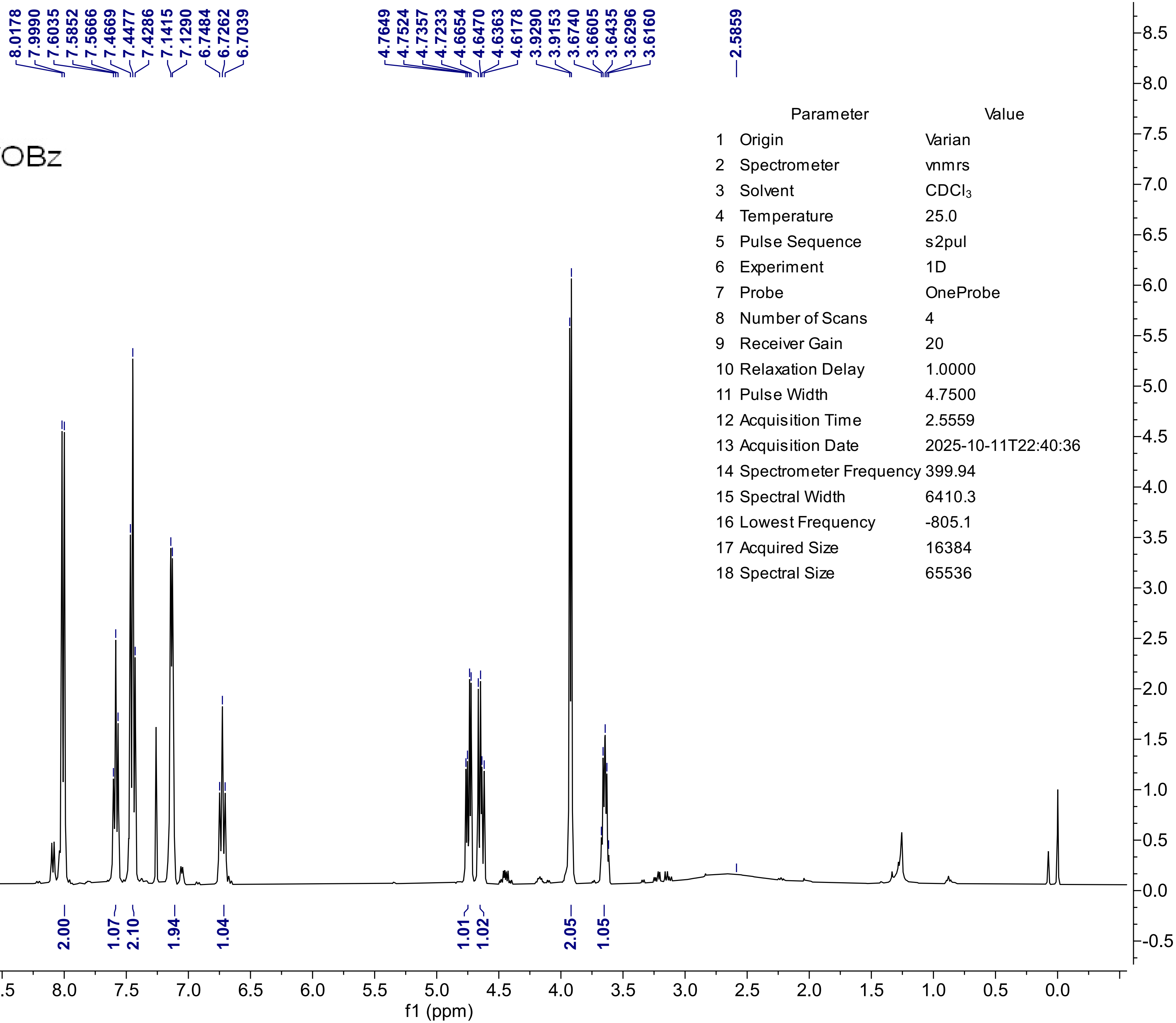

	Parameter	Value
1	Origin	Varian
2	Spectrometer	vnmrs
3	Solvent	$CDCl_3$
4	Temperature	25.0
5	Pulse Sequence	s2pul
6	Experiment	1D
7	Probe	OneProbe
8	Number of Scans	4
9	Receiver Gain	20
10	Relaxation Delay	1.0000
11	Pulse Width	4.7500
12	Acquisition Time	2.5559
13	Acquisition Date	2025-10-11T22:40:36
14	Spectrometer Frequency	399.94
15	Spectral Width	6410.3
16	Lowest Frequency	-805.1
17	Acquired Size	16384
18	Spectral Size	65536

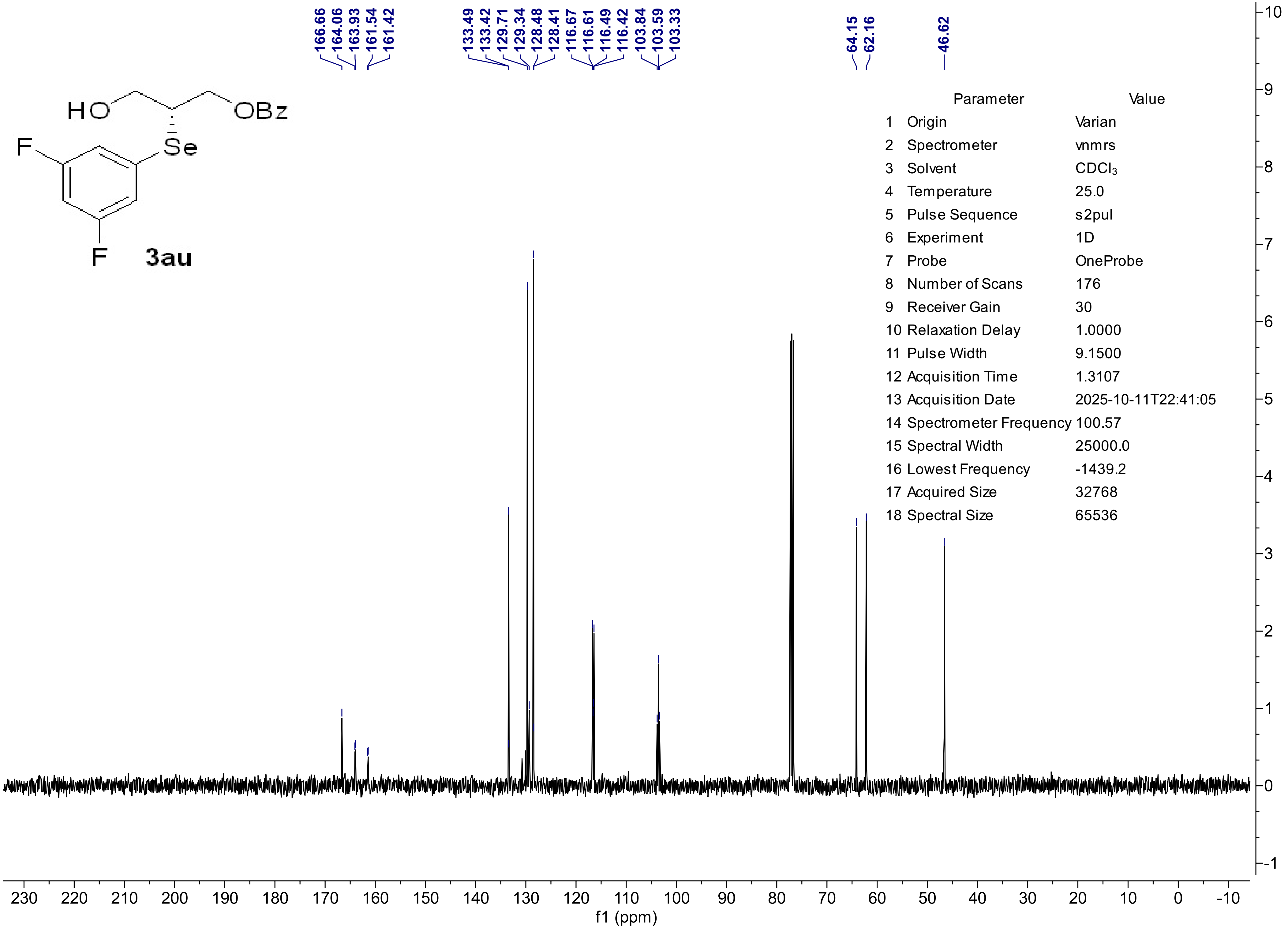

	Parameter	Value
1	Origin	Varian
2	Spectrometer	vnmrs
3	Solvent	$CDCl_3$
4	Temperature	25.0
5	Pulse Sequence	s2pul
6	Experiment	1D
7	Probe	OneProbe
8	Number of Scans	176
9	Receiver Gain	30
10	Relaxation Delay	1.0000
11	Pulse Width	9.1500
12	Acquisition Time	1.3107
13	Acquisition Date	2025-10-11T22:41:05
14	Spectrometer Frequency	100.57
15	Spectral Width	25000.0
16	Lowest Frequency	-1439.2
17	Acquired Size	32768
18	Spectral Size	65536

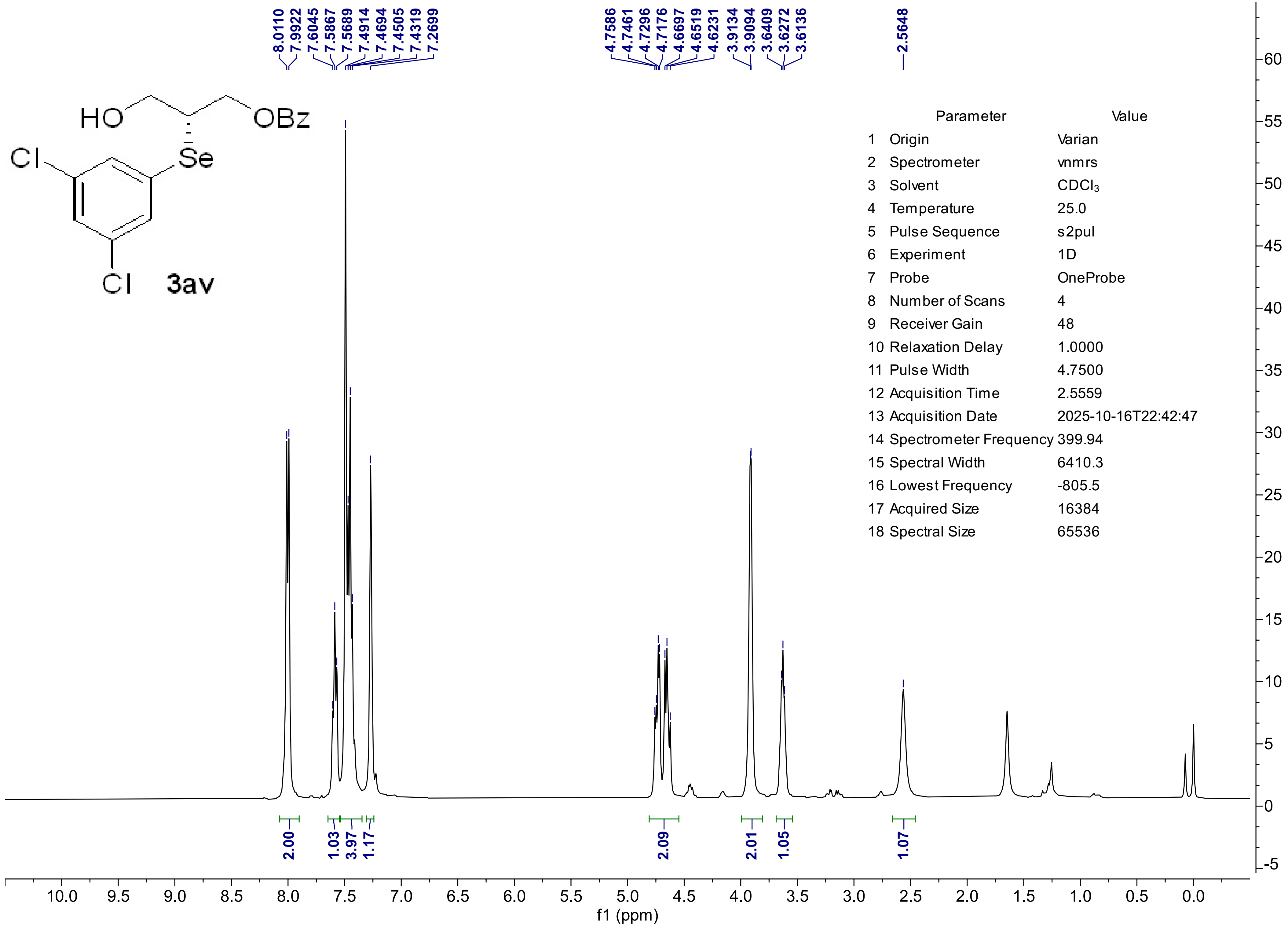

	Parameter	Value
1	Origin	Varian
2	Spectrometer	vnmrs
3	Solvent	$CDCl_3$
4	Temperature	25.0
5	Pulse Sequence	s2pul
6	Experiment	1D
7	Probe	OneProbe
8	Number of Scans	4
9	Receiver Gain	48
10	Relaxation Delay	1.0000
11	Pulse Width	4.7500
12	Acquisition Time	2.5559
13	Acquisition Date	2025-10-16T22:42:47
14	Spectrometer Frequency	399.94
15	Spectral Width	6410.3
16	Lowest Frequency	-805.5
17	Acquired Size	16384
18	Spectral Size	65536

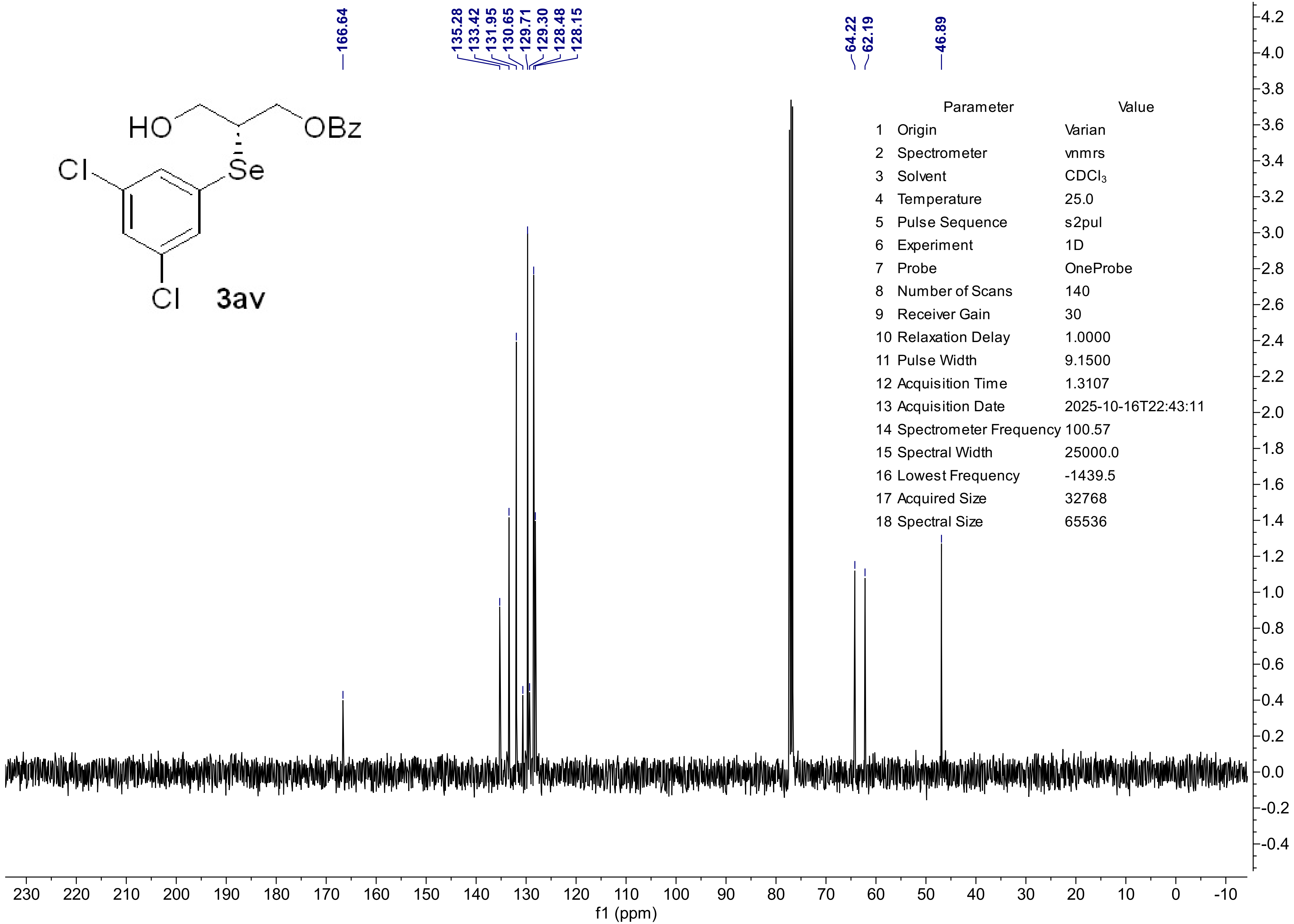
166.64
135.28
133.42
131.95
130.65
129.71
129.30
128.48
128.15
64.22
62.19
46.89
HO
OBz
Se
Cl
Cl
3av
Parameter | Value
1 Origin | Varian
2 Spectrometer | vnmrs
3 Solvent | CDCl3
4 Temperature | 25.0
5 Pulse Sequence | s2pul
6 Experiment | 1D
7 Probe | OneProbe
8 Number of Scans | 140
9 Receiver Gain | 30
10 Relaxation Delay | 1.0000
11 Pulse Width | 9.1500
12 Acquisition Time | 1.3107
13 Acquisition Date | 2025-10-16T22:43:11
14 Spectrometer Frequency | 100.57
15 Spectral Width | 25000.0
16 Lowest Frequency | -1439.5
17 Acquired Size | 32768
18 Spectral Size | 65536
f1 (ppm)

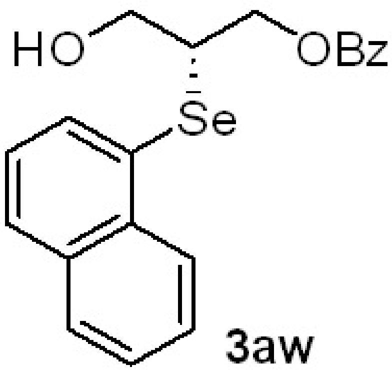

3aw

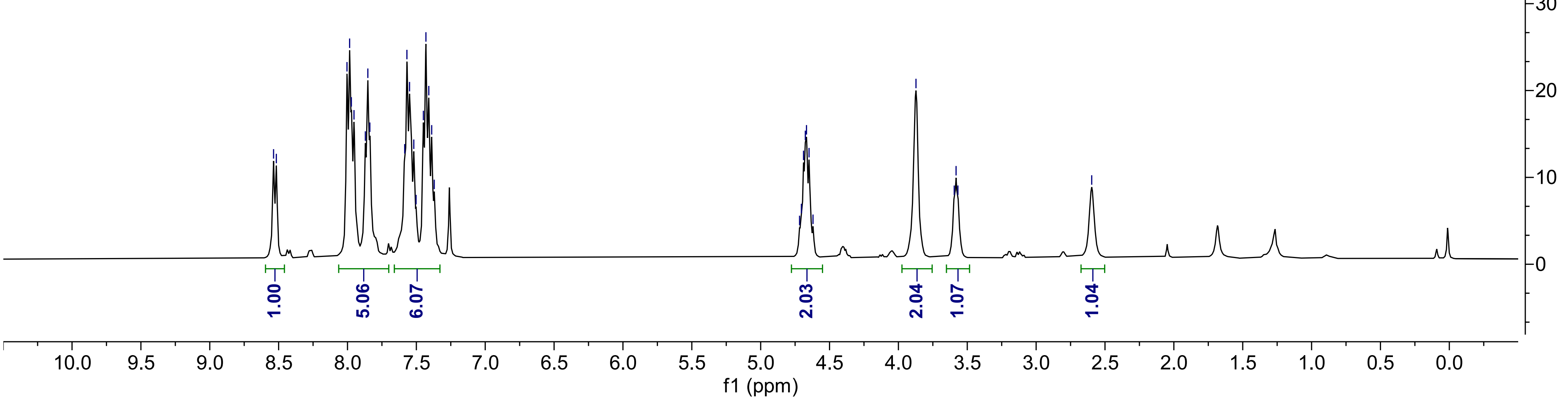

	Parameter	Value
1	Origin	Varian
2	Spectrometer	vnmrs
3	Solvent	$CDCl_3$
4	Temperature	25.0
5	Pulse Sequence	s2pul
6	Experiment	1D
7	Probe	OneProbe
8	Number of Scans	4
9	Receiver Gain	44
10	Relaxation Delay	1.0000
11	Pulse Width	4.7500
12	Acquisition Time	2.5559
13	Acquisition Date	2025-09-23T22:52:11
14	Spectrometer Frequency	399.94
15	Spectral Width	6410.3
16	Lowest Frequency	-805.5
17	Acquired Size	16384
18	Spectral Size	65536

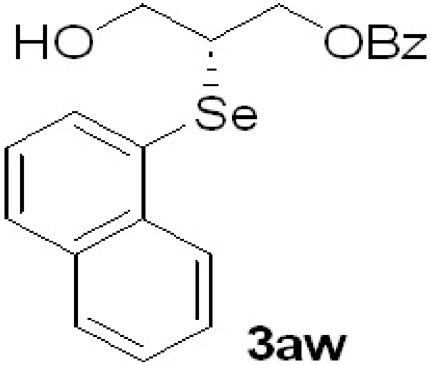

3aw

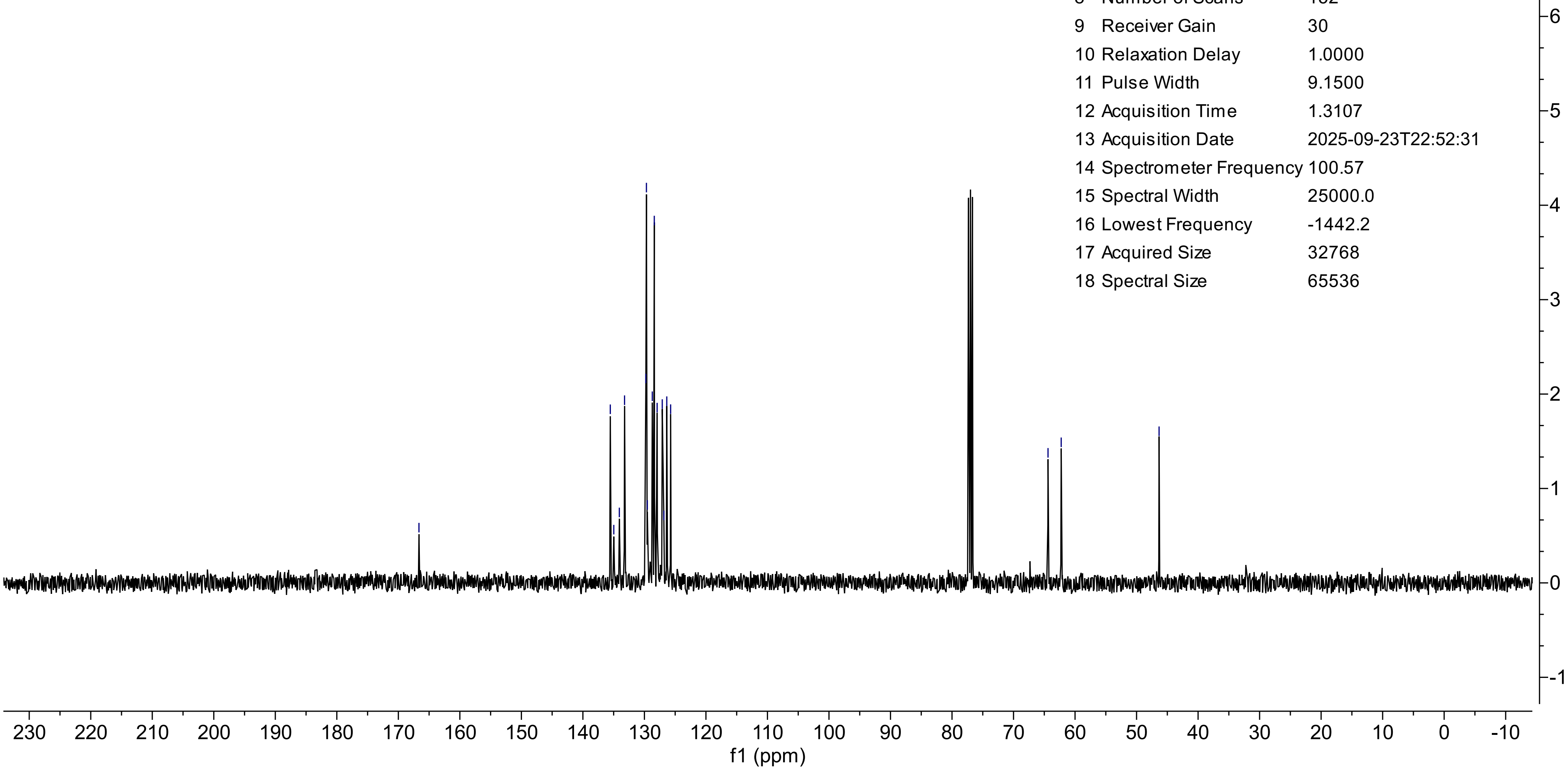

	Parameter	Value
1	Origin	Varian
2	Spectrometer	vnmrs
3	Solvent	$CDCl_3$
4	Temperature	25.0
5	Pulse Sequence	s2pul
6	Experiment	1D
7	Probe	OneProbe
8	Number of Scans	152
9	Receiver Gain	30
10	Relaxation Delay	1.0000
11	Pulse Width	9.1500
12	Acquisition Time	1.3107
13	Acquisition Date	2025-09-23T22:52:31
14	Spectrometer Frequency	100.57
15	Spectral Width	25000.0
16	Lowest Frequency	-1442.2
17	Acquired Size	32768
18	Spectral Size	65536

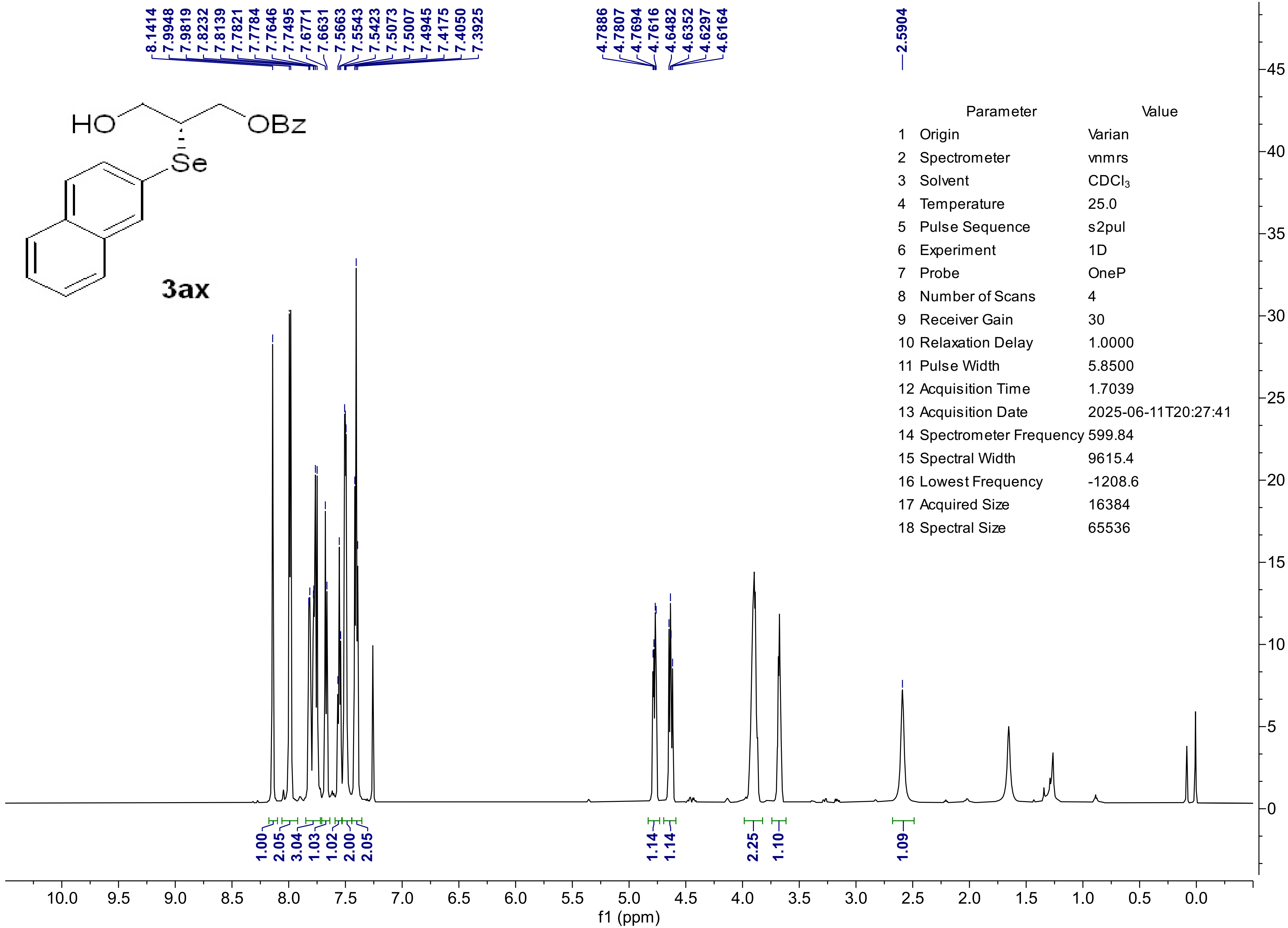

	Parameter	Value
1	Origin	Varian
2	Spectrometer	vnmrs
3	Solvent	$CDCl_3$
4	Temperature	25.0
5	Pulse Sequence	s2pul
6	Experiment	1D
7	Probe	OneP
8	Number of Scans	4
9	Receiver Gain	30
10	Relaxation Delay	1.0000
11	Pulse Width	5.8500
12	Acquisition Time	1.7039
13	Acquisition Date	2025-06-11T20:27:41
14	Spectrometer Frequency	599.84
15	Spectral Width	9615.4
16	Lowest Frequency	-1208.6
17	Acquired Size	16384
18	Spectral Size	65536

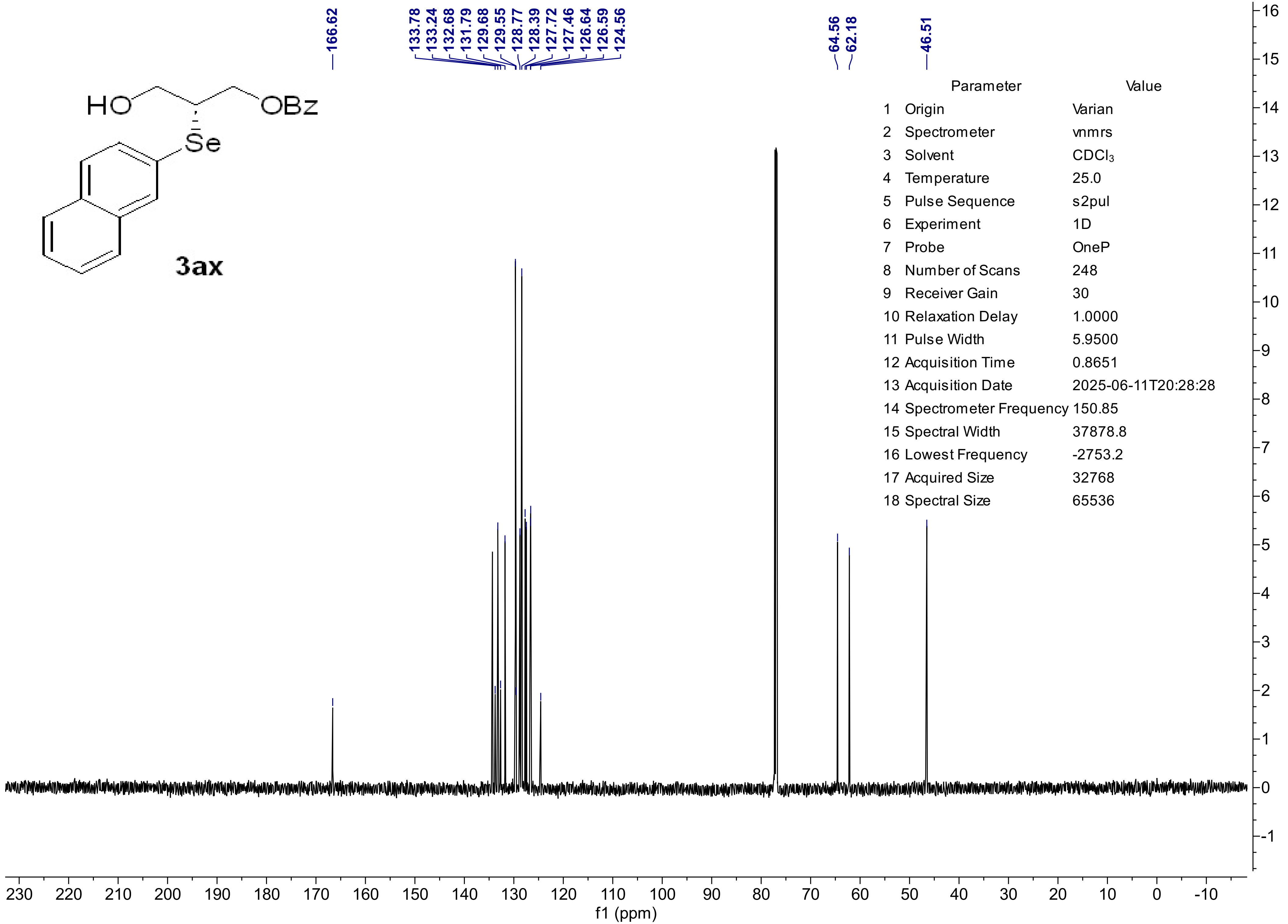

	Parameter	Value
1	Origin	Varian
2	Spectrometer	vnmrs
3	Solvent	$CDCl_3$
4	Temperature	25.0
5	Pulse Sequence	s2pul
6	Experiment	1D
7	Probe	OneP
8	Number of Scans	248
9	Receiver Gain	30
10	Relaxation Delay	1.0000
11	Pulse Width	5.9500
12	Acquisition Time	0.8651
13	Acquisition Date	2025-06-11T20:28:28
14	Spectrometer Frequency	150.85
15	Spectral Width	37878.8
16	Lowest Frequency	-2753.2
17	Acquired Size	32768
18	Spectral Size	65536

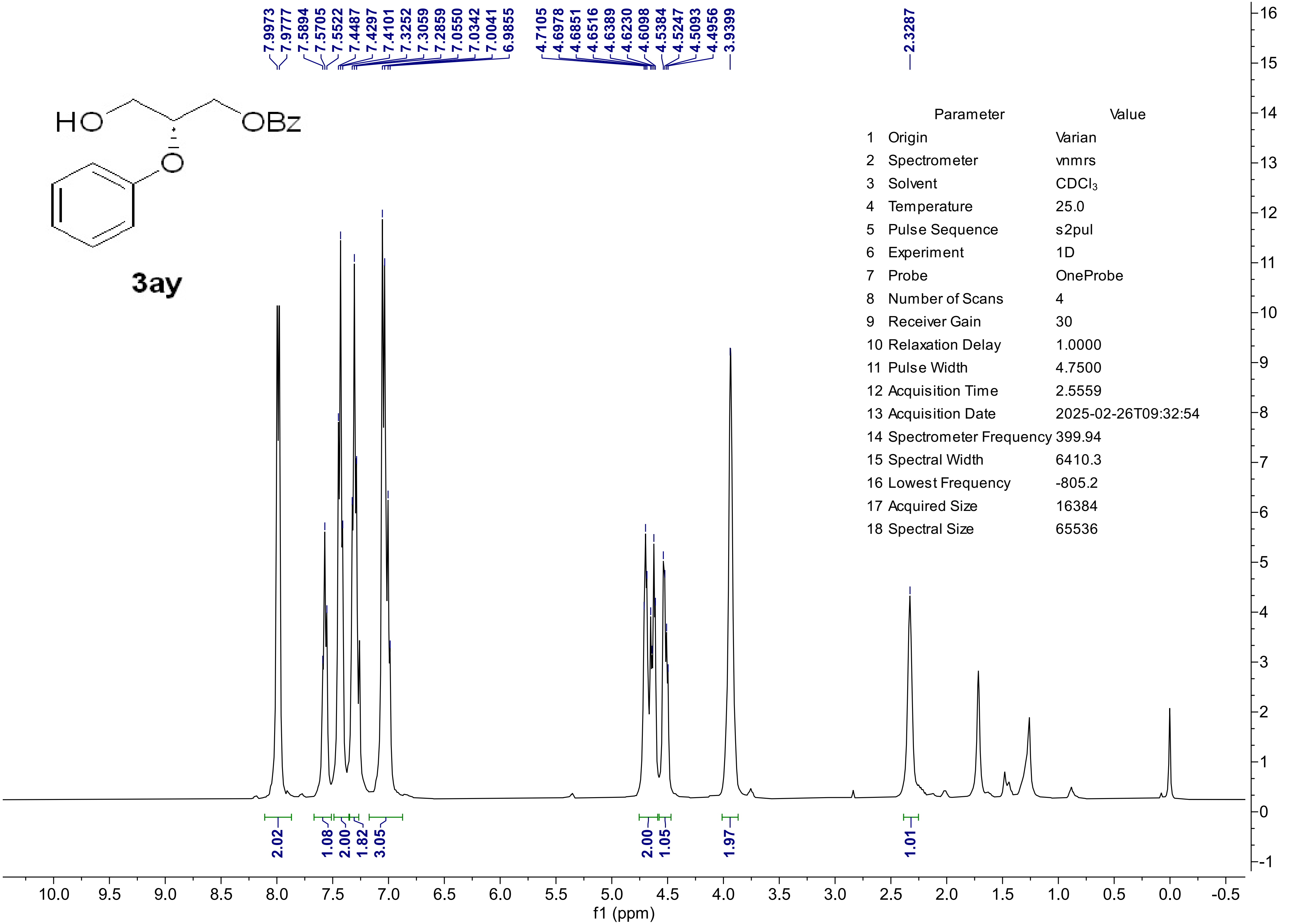

	Parameter	Value
1	Origin	Varian
2	Spectrometer	vnmrs
3	Solvent	$CDCl_3$
4	Temperature	25.0
5	Pulse Sequence	s2pul
6	Experiment	1D
7	Probe	OneProbe
8	Number of Scans	4
9	Receiver Gain	30
10	Relaxation Delay	1.0000
11	Pulse Width	4.7500
12	Acquisition Time	2.5559
13	Acquisition Date	2025-02-26T09:32:54
14	Spectrometer Frequency	399.94
15	Spectral Width	6410.3
16	Lowest Frequency	-805.2
17	Acquired Size	16384
18	Spectral Size	65536

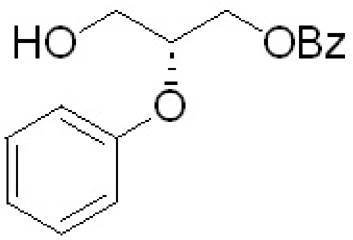

3ay

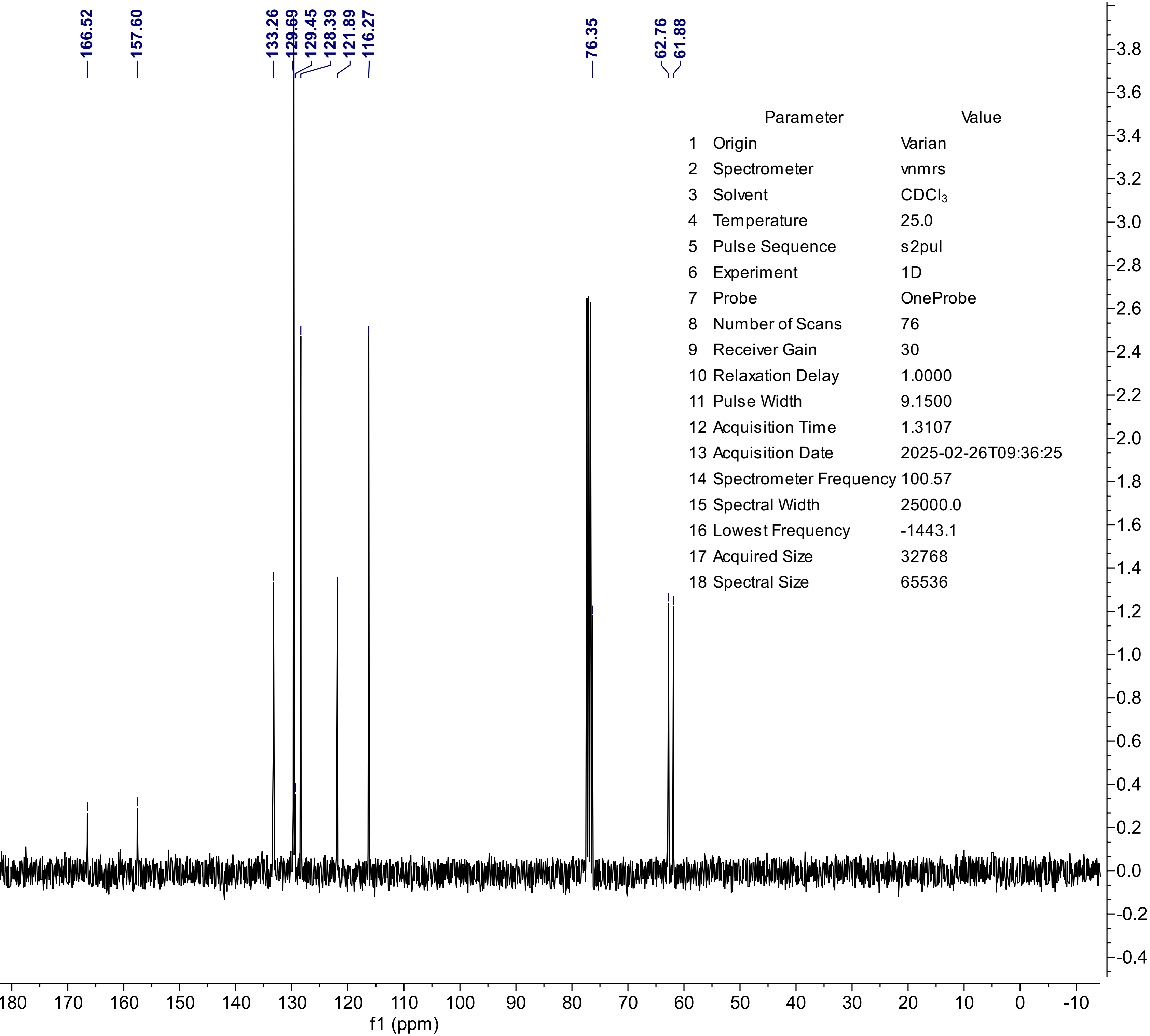

	Parameter	Value
1	Origin	Varian
2	Spectrometer	vnmrs
3	Solvent	$CDCl_3$
4	Temperature	25.0
5	Pulse Sequence	s2pul
6	Experiment	1D
7	Probe	OneProbe
8	Number of Scans	76
9	Receiver Gain	30
10	Relaxation Delay	1.0000
11	Pulse Width	9.1500
12	Acquisition Time	1.3107
13	Acquisition Date	2025-02-26T09:36:25
14	Spectrometer Frequency	100.57
15	Spectral Width	25000.0
16	Lowest Frequency	-1443.1
17	Acquired Size	32768
18	Spectral Size	65536

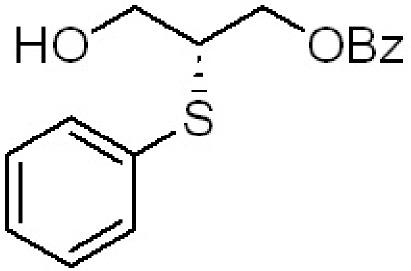

3az

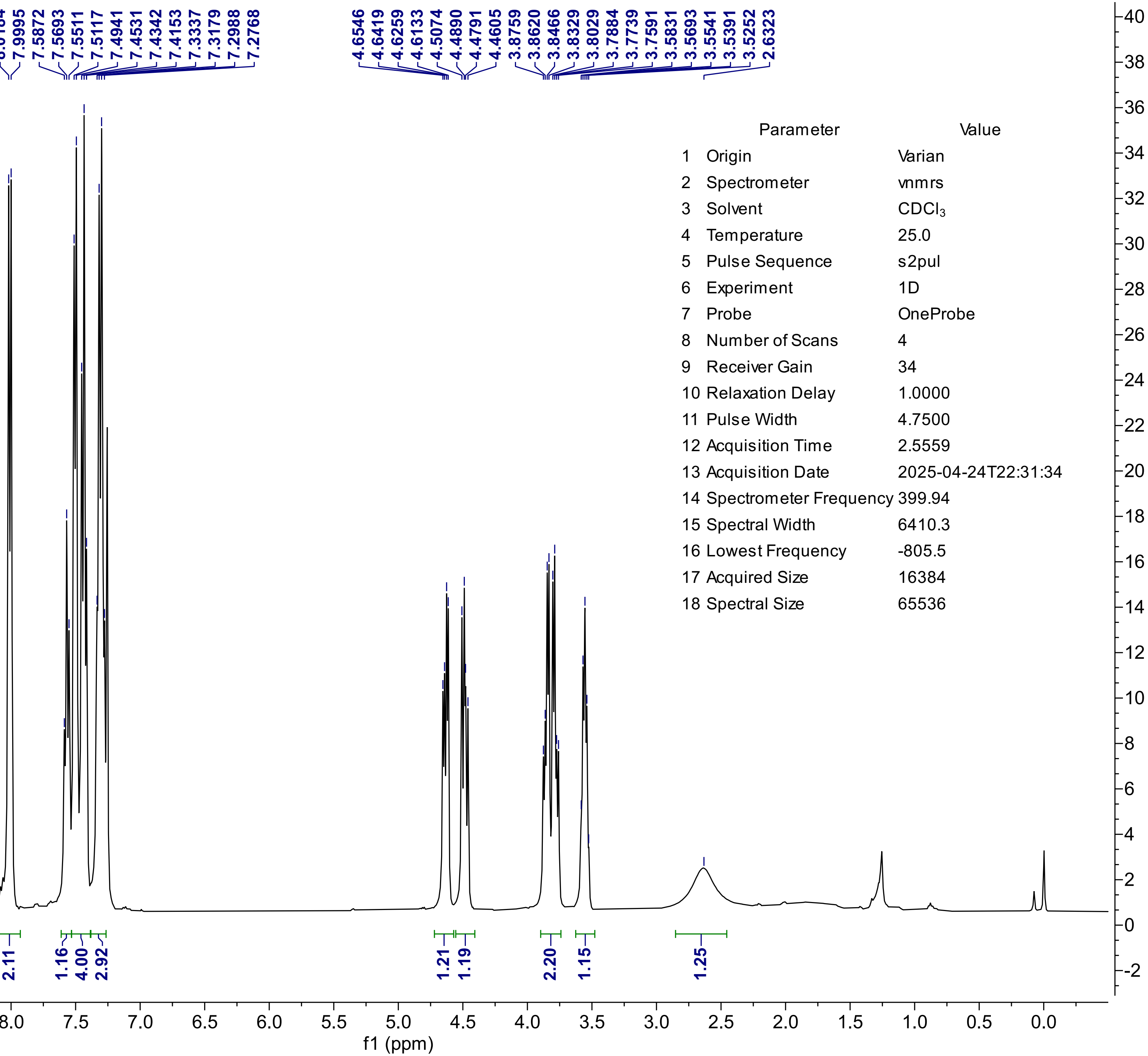

	Parameter	Value
1	Origin	Varian
2	Spectrometer	vnmrs
3	Solvent	$CDCl_3$
4	Temperature	25.0
5	Pulse Sequence	s2pul
6	Experiment	1D
7	Probe	OneProbe
8	Number of Scans	4
9	Receiver Gain	34
10	Relaxation Delay	1.0000
11	Pulse Width	4.7500
12	Acquisition Time	2.5559
13	Acquisition Date	2025-04-24T22:31:34
14	Spectrometer Frequency	399.94
15	Spectral Width	6410.3
16	Lowest Frequency	-805.5
17	Acquired Size	16384
18	Spectral Size	65536

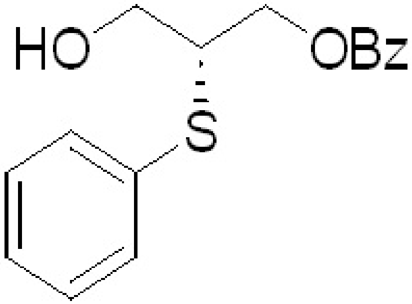

3az

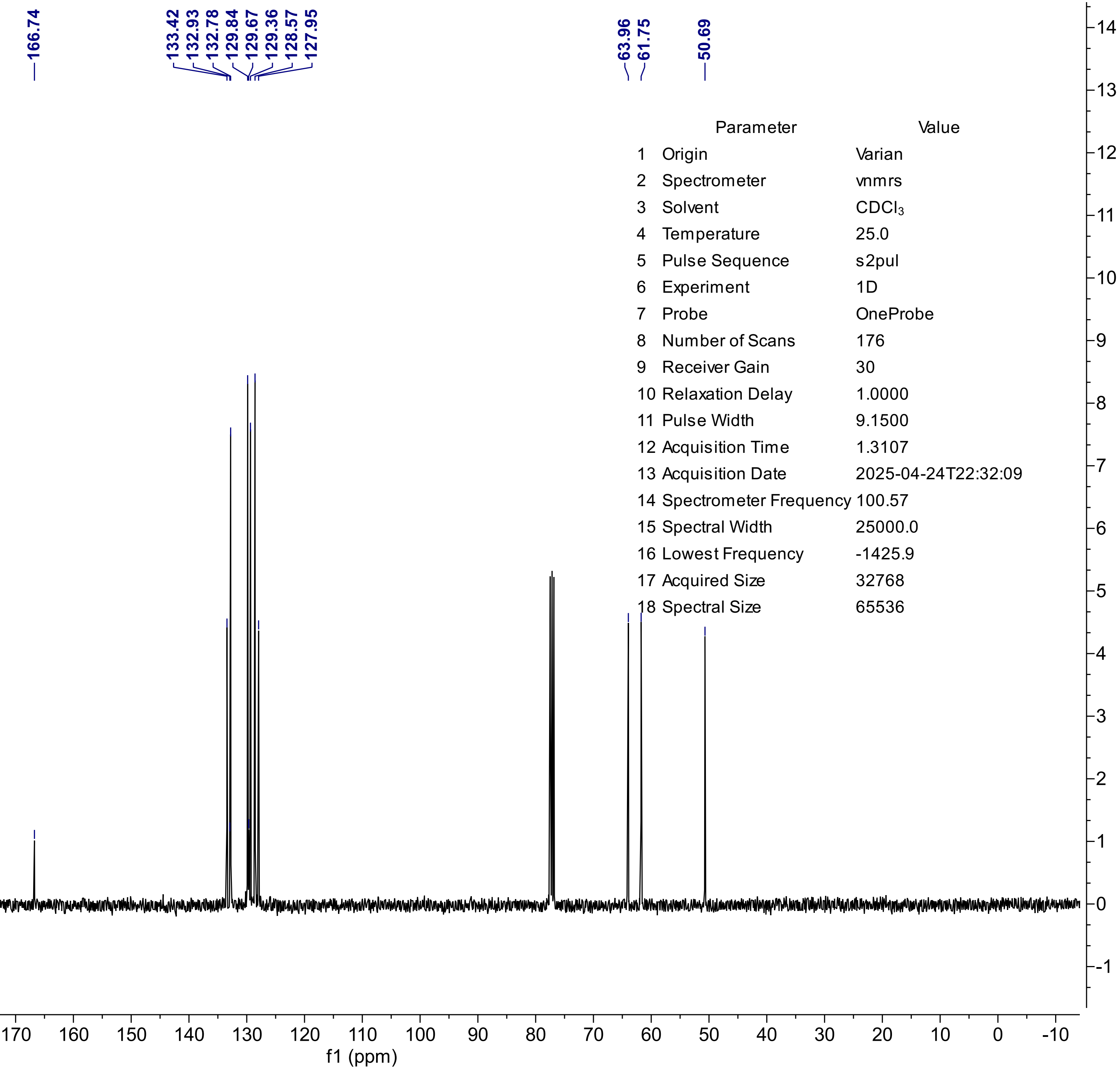

	Parameter	Value
1	Origin	Varian
2	Spectrometer	vnmrs
3	Solvent	$CDCl_3$
4	Temperature	25.0
5	Pulse Sequence	s2pul
6	Experiment	1D
7	Probe	OneProbe
8	Number of Scans	176
9	Receiver Gain	30
10	Relaxation Delay	1.0000
11	Pulse Width	9.1500
12	Acquisition Time	1.3107
13	Acquisition Date	2025-04-24T22:32:09
14	Spectrometer Frequency	100.57
15	Spectral Width	25000.0
16	Lowest Frequency	-1425.9
17	Acquired Size	32768
18	Spectral Size	65536

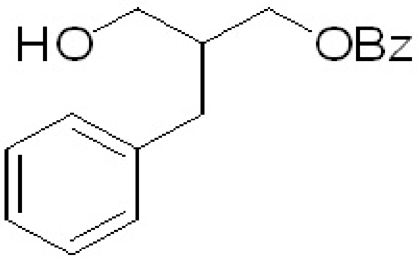

3ba

	Parameter	Value
1	Origin	Varian
2	Spectrometer	vnmrs
3	Solvent	$CDCl_3$
4	Temperature	25.0
5	Pulse Sequence	s2pul
6	Experiment	1D
7	Probe	OneProbe
8	Number of Scans	4
9	Receiver Gain	40
10	Relaxation Delay	1.0000
11	Pulse Width	4.7500
12	Acquisition Time	2.5559
13	Acquisition Date	2025-02-27T11:04:53
14	Spectrometer Frequency	399.94
15	Spectral Width	6410.3
16	Lowest Frequency	-805.2
17	Acquired Size	16384
18	Spectral Size	65536

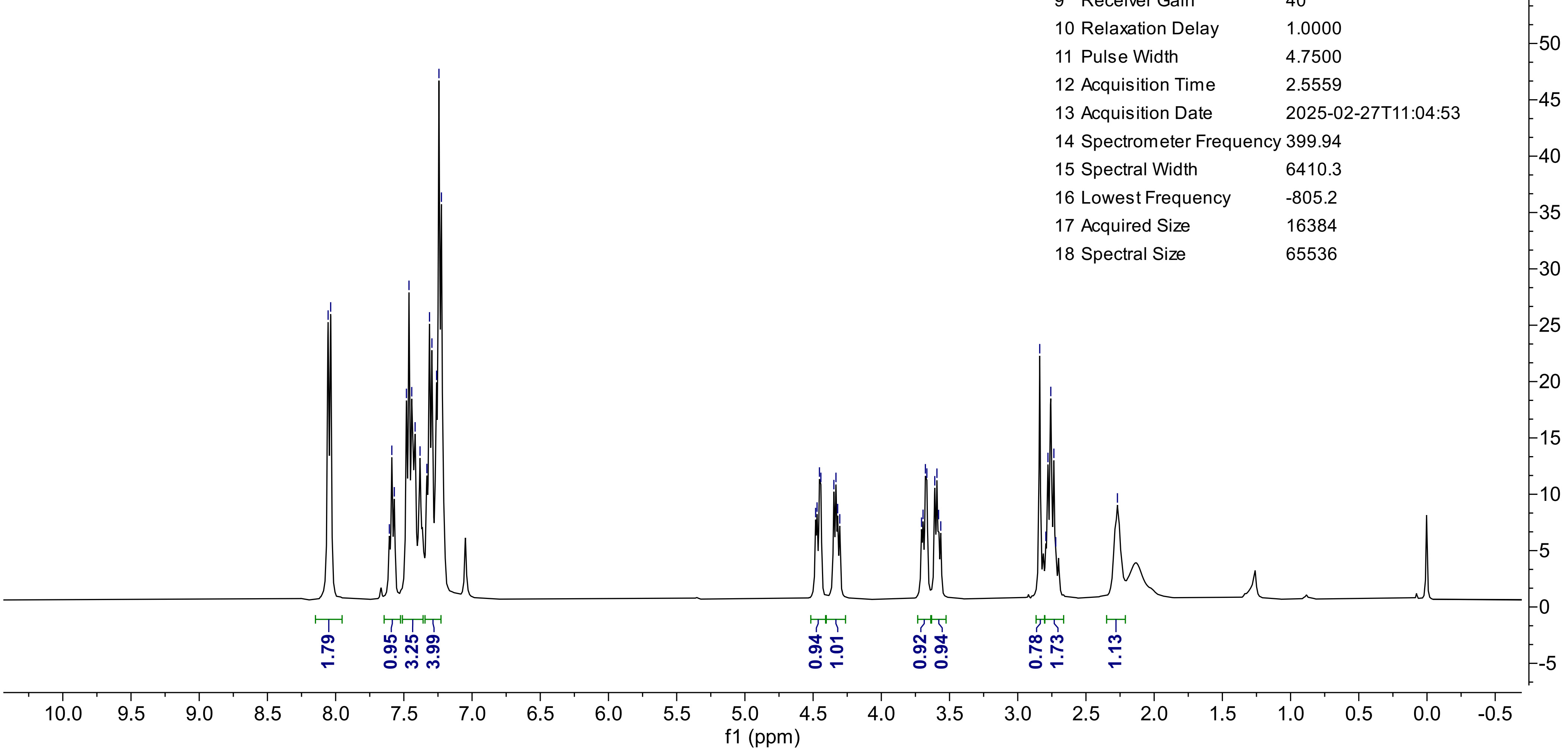

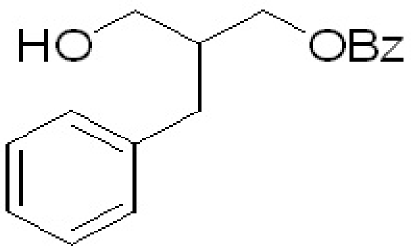

3ba

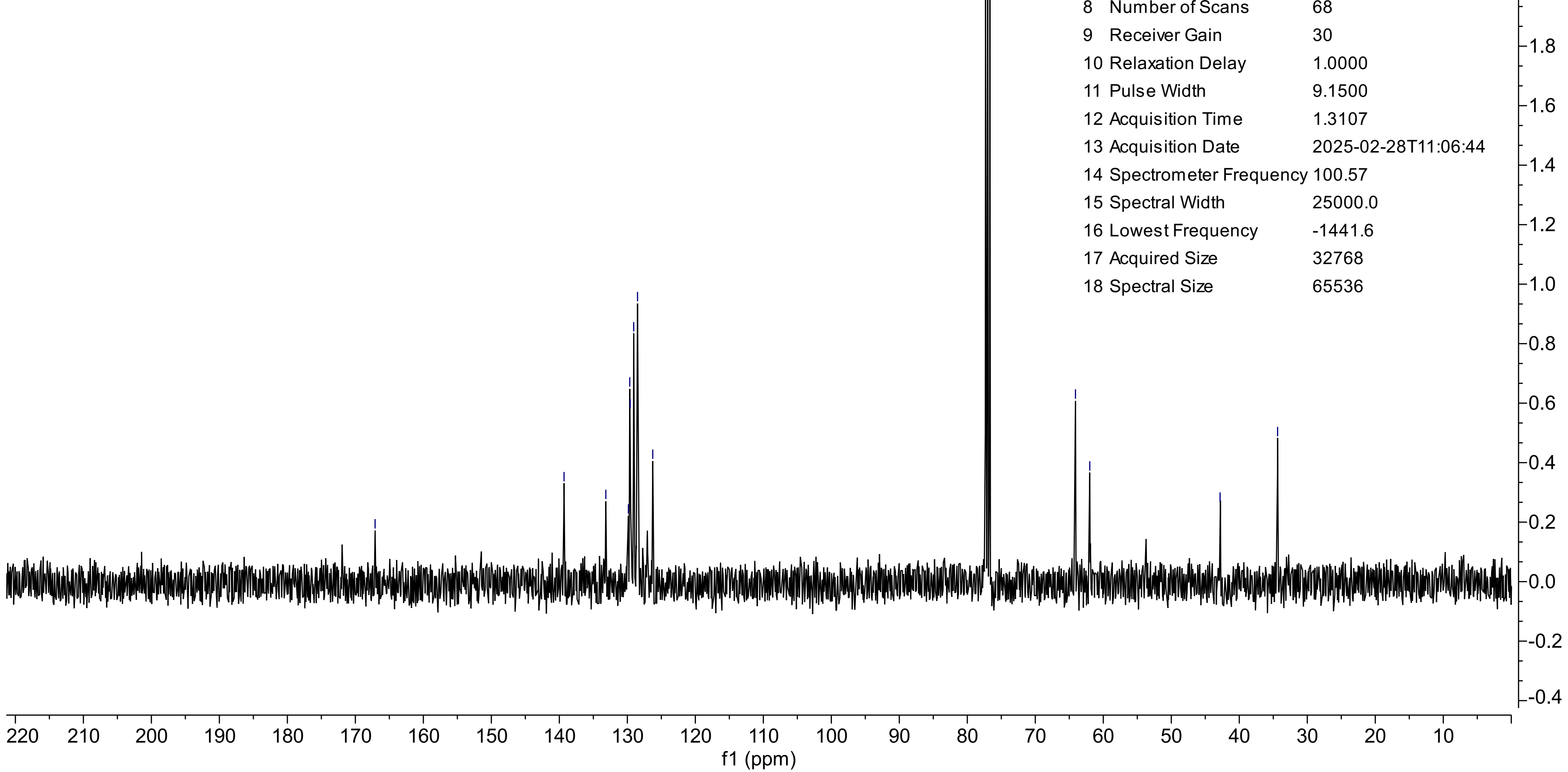

	Parameter	Value
1	Origin	Varian
2	Spectrometer	vnmrs
3	Solvent	$CDCl_3$
4	Temperature	25.0
5	Pulse Sequence	s2pul
6	Experiment	1D
7	Probe	OneProbe
8	Number of Scans	68
9	Receiver Gain	30
10	Relaxation Delay	1.0000
11	Pulse Width	9.1500
12	Acquisition Time	1.3107
13	Acquisition Date	2025-02-28T11:06:44
14	Spectrometer Frequency	100.57
15	Spectral Width	25000.0
16	Lowest Frequency	-1441.6
17	Acquired Size	32768
18	Spectral Size	65536

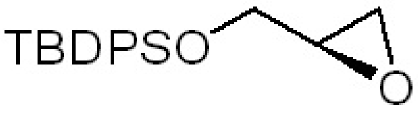

5

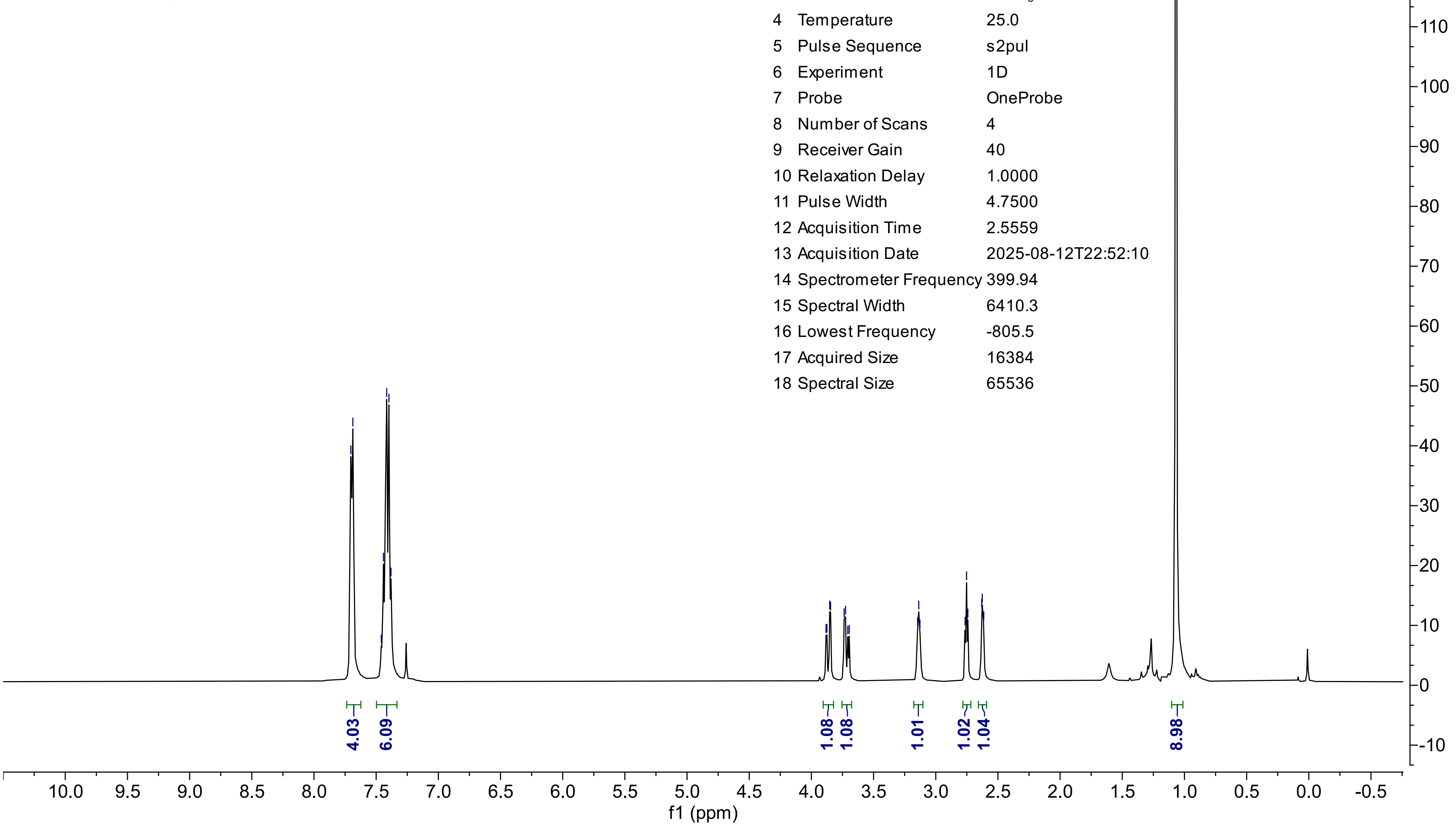

	Parameter	Value
1	Origin	Varian
2	Spectrometer	vnmrs
3	Solvent	$CDCl_3$
4	Temperature	25.0
5	Pulse Sequence	s2pul
6	Experiment	1D
7	Probe	OneProbe
8	Number of Scans	4
9	Receiver Gain	40
10	Relaxation Delay	1.0000
11	Pulse Width	4.7500
12	Acquisition Time	2.5559
13	Acquisition Date	2025-08-12T22:52:10
14	Spectrometer Frequency	399.94
15	Spectral Width	6410.3
16	Lowest Frequency	-805.5
17	Acquired Size	16384
18	Spectral Size	65536

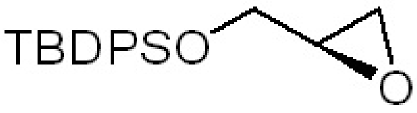

5

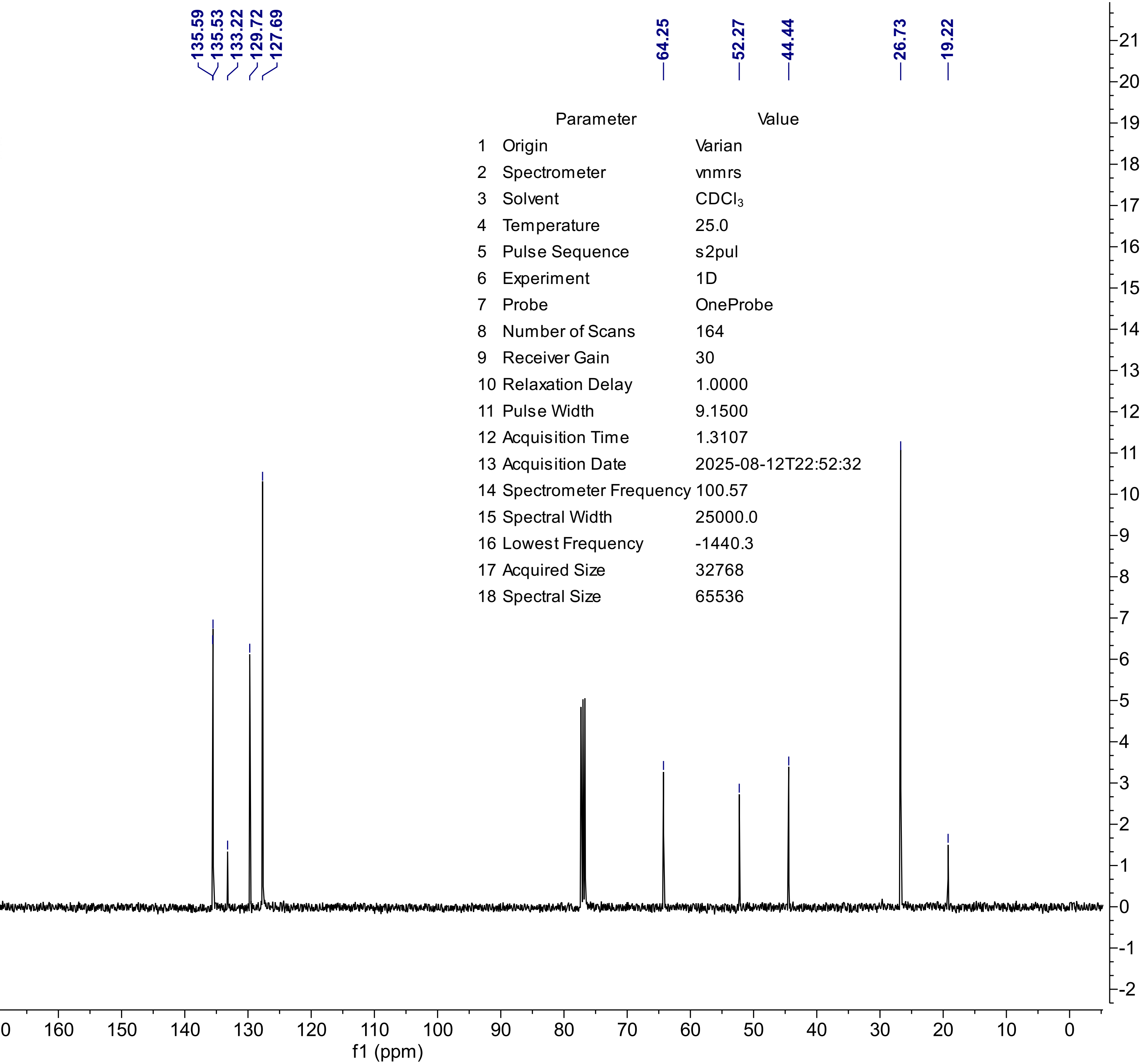

	Parameter	Value
1	Origin	Varian
2	Spectrometer	vnmrs
3	Solvent	$CDCl_3$
4	Temperature	25.0
5	Pulse Sequence	s2pul
6	Experiment	1D
7	Probe	OneProbe
8	Number of Scans	164
9	Receiver Gain	30
10	Relaxation Delay	1.0000
11	Pulse Width	9.1500
12	Acquisition Time	1.3107
13	Acquisition Date	2025-08-12T22:52:32
14	Spectrometer Frequency	100.57
15	Spectral Width	25000.0
16	Lowest Frequency	-1440.3
17	Acquired Size	32768
18	Spectral Size	65536

HQQ-S3-100-H-10.3
Gradient Shimming

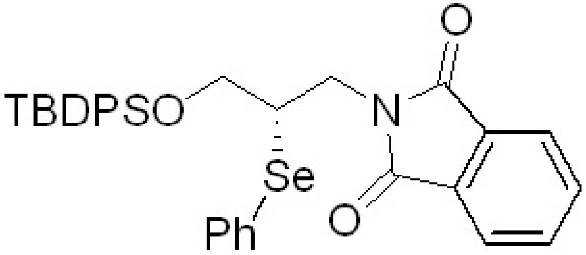

6'

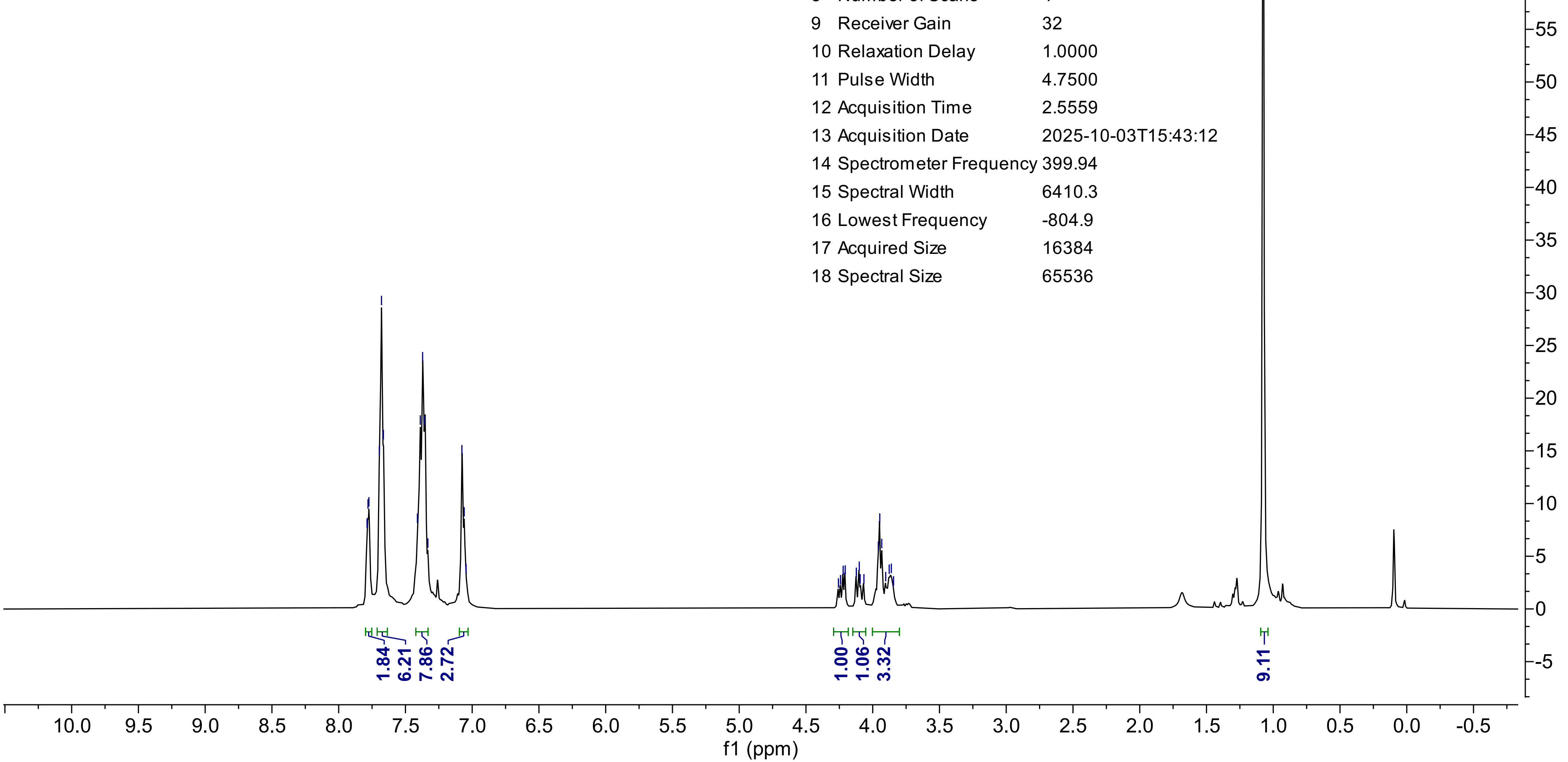

	Parameter	Value
1	Origin	Varian
2	Spectrometer	vnmrs
3	Solvent	$CDCl_3$
4	Temperature	25.0
5	Pulse Sequence	s2pul
6	Experiment	1D
7	Probe	OneProbe
8	Number of Scans	4
9	Receiver Gain	32
10	Relaxation Delay	1.0000
11	Pulse Width	4.7500
12	Acquisition Time	2.5559
13	Acquisition Date	2025-10-03T15:43:12
14	Spectrometer Frequency	399.94
15	Spectral Width	6410.3
16	Lowest Frequency	-804.9
17	Acquired Size	16384
18	Spectral Size	65536

HQQ-S3-100-C-10.3
Gradient Shimming

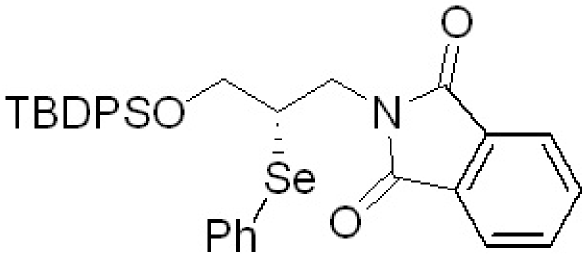

6'

	Parameter	Value
1	Origin	Varian
2	Spectrometer	vnmrs
3	Solvent	$CDCl_3$
4	Temperature	25.0
5	Pulse Sequence	s2pul
6	Experiment	1D
7	Probe	OneProbe
8	Number of Scans	120
9	Receiver Gain	30
10	Relaxation Delay	1.0000
11	Pulse Width	9.1500
12	Acquisition Time	1.3107
13	Acquisition Date	2025-10-03T15:43:32
14	Spectrometer Frequency	100.57
15	Spectral Width	25000.0
16	Lowest Frequency	-1444.4
17	Acquired Size	32768
18	Spectral Size	65536

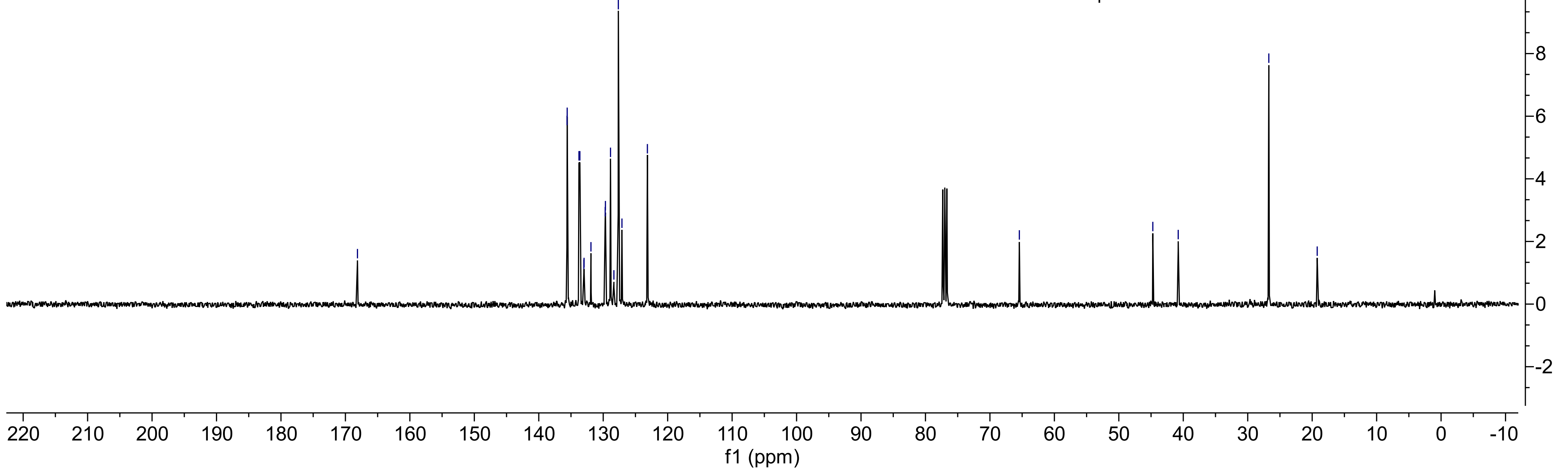

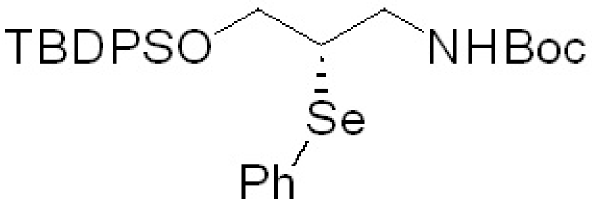

6

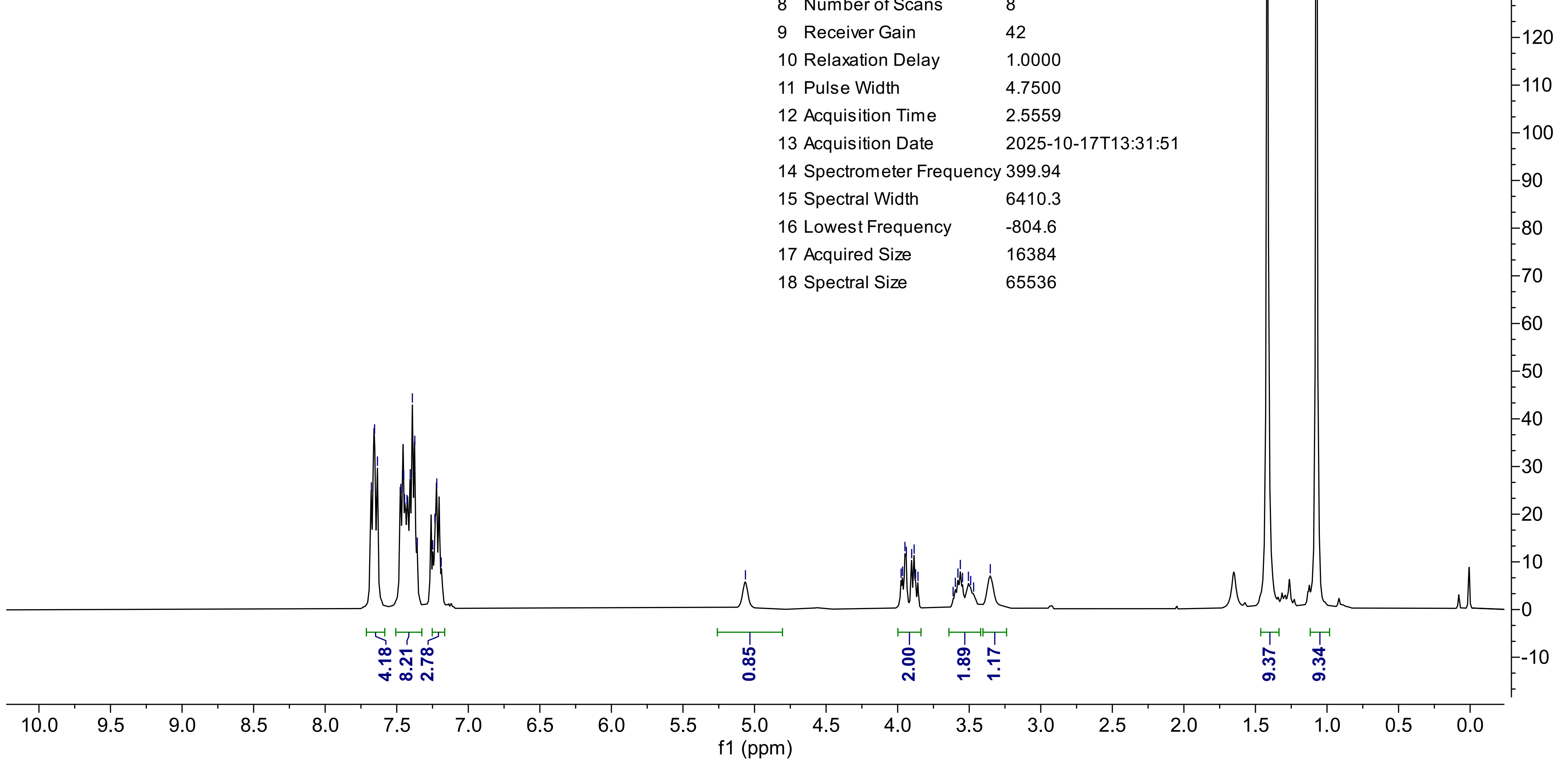

	Parameter	Value
1	Origin	Varian
2	Spectrometer	vnmrs
3	Solvent	$CDCl_3$
4	Temperature	25.0
5	Pulse Sequence	s2pul
6	Experiment	1D
7	Probe	OneProbe
8	Number of Scans	8
9	Receiver Gain	42
10	Relaxation Delay	1.0000
11	Pulse Width	4.7500
12	Acquisition Time	2.5559
13	Acquisition Date	2025-10-17T13:31:51
14	Spectrometer Frequency	399.94
15	Spectral Width	6410.3
16	Lowest Frequency	-804.6
17	Acquired Size	16384
18	Spectral Size	65536

HQQ-S3-115-NBOC-C-400M
Gradient Shimming

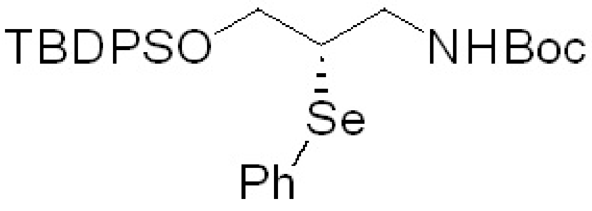

6

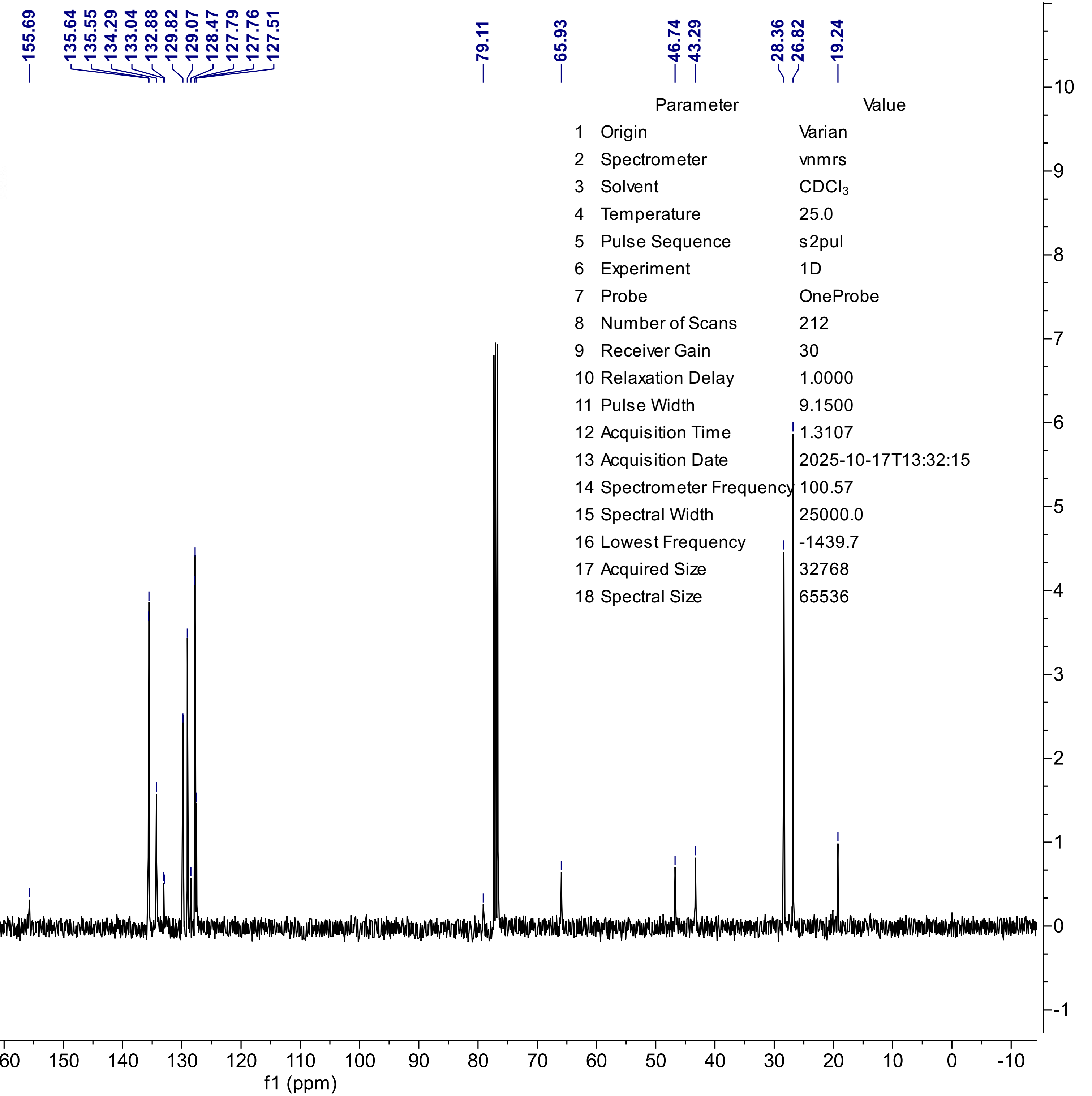

	Parameter	Value
1	Origin	Varian
2	Spectrometer	vnmrs
3	Solvent	$CDCl_3$
4	Temperature	25.0
5	Pulse Sequence	s2pul
6	Experiment	1D
7	Probe	OneProbe
8	Number of Scans	212
9	Receiver Gain	30
10	Relaxation Delay	1.0000
11	Pulse Width	9.1500
12	Acquisition Time	1.3107
13	Acquisition Date	2025-10-17T13:32:15
14	Spectrometer Frequency	100.57
15	Spectral Width	25000.0
16	Lowest Frequency	-1439.7
17	Acquired Size	32768
18	Spectral Size	65536

hqq-s3-97-stp2-2-H-600M

Selective band center: 6.87 (ppm); width: 28.7 (Hz)

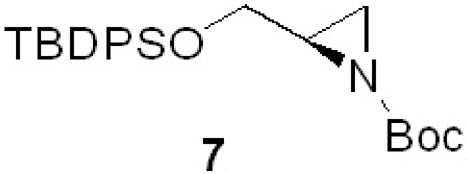

7

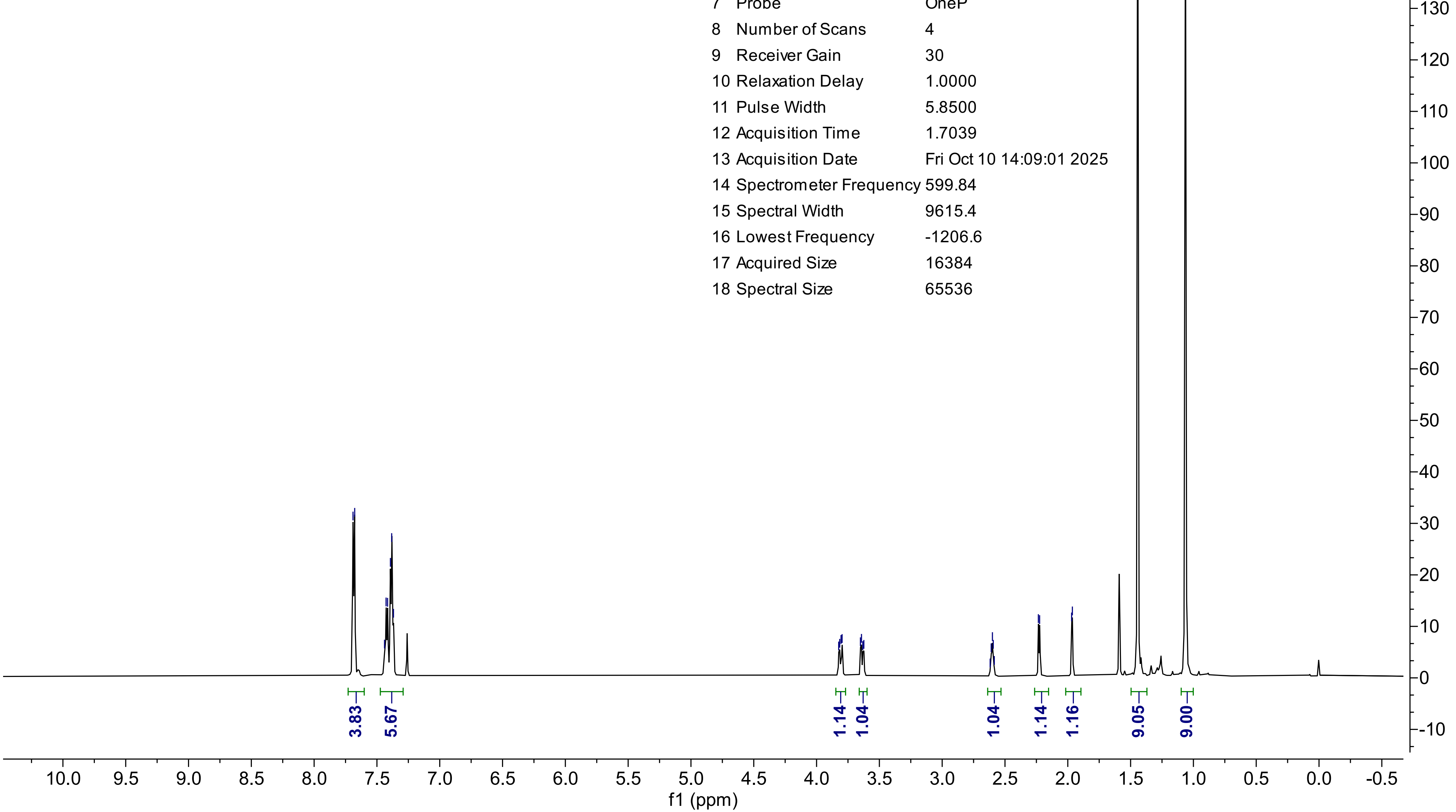

	Parameter	Value
1	Origin	Varian
2	Spectrometer	vnmrs
3	Solvent	$CDCl_3$
4	Temperature	25.0
5	Pulse Sequence	s2pul
6	Experiment	1D
7	Probe	OneP
8	Number of Scans	4
9	Receiver Gain	30
10	Relaxation Delay	1.0000
11	Pulse Width	5.8500
12	Acquisition Time	1.7039
13	Acquisition Date	Fri Oct 10 14:09:01 2025
14	Spectrometer Frequency	599.84
15	Spectral Width	9615.4
16	Lowest Frequency	-1206.6
17	Acquired Size	16384
18	Spectral Size	65536

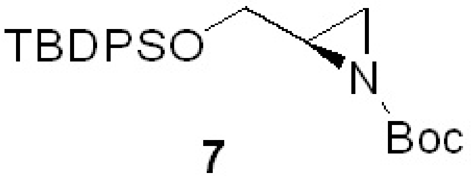
TBDPSO
N
Boc

7

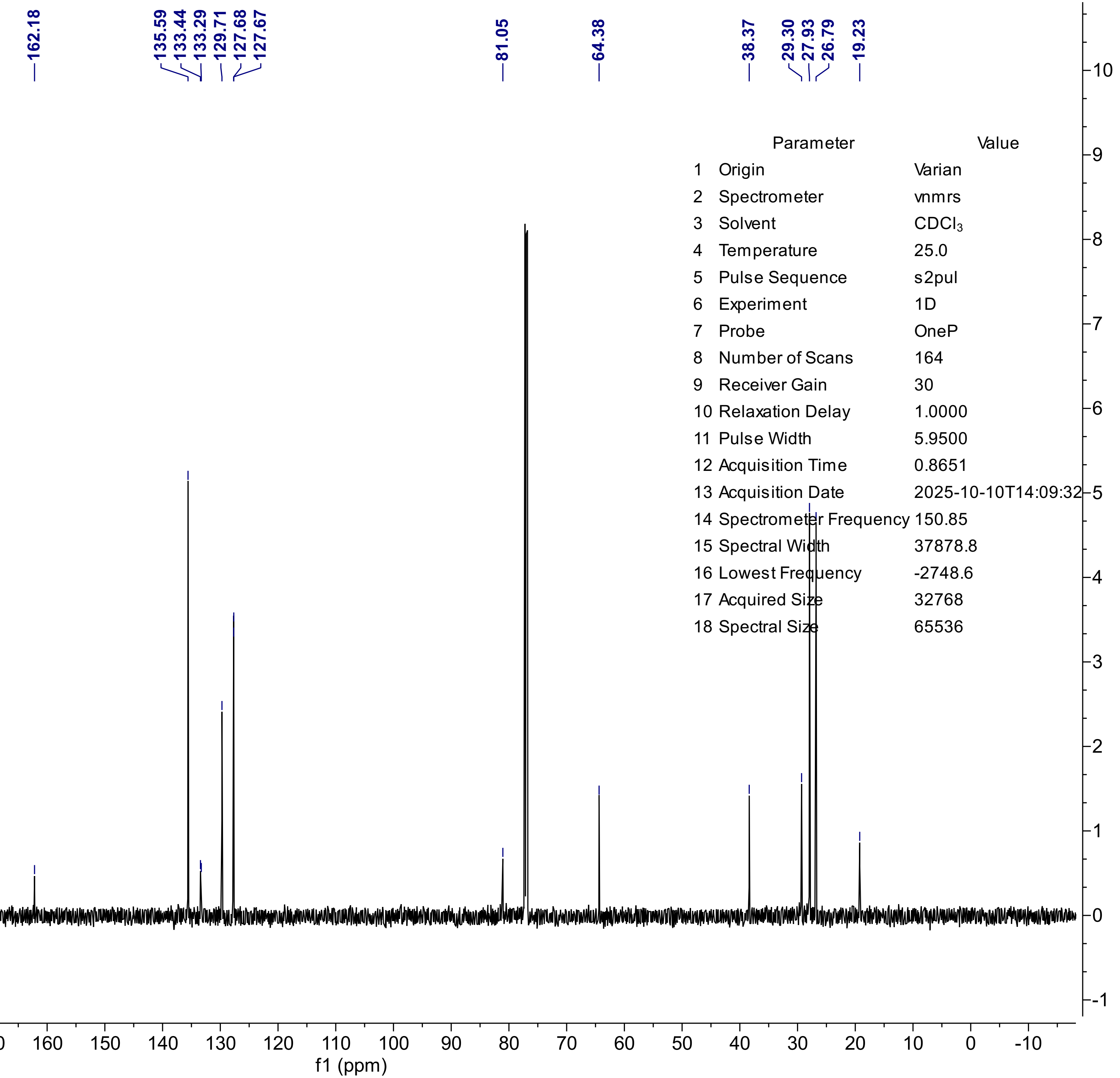
hqq-s3-97-stp2-2-C-600M
Selective band center: 6.87 (ppm); width: 28.7 (Hz)
162.18
135.59
133.44
133.29
129.71
127.68
127.67
81.05
64.38
38.37
29.30
27.93
26.79
19.23
Parameter Value
1 Origin Varian
2 Spectrometer vnmrs
3 Solvent CDCl3
4 Temperature 25.0
5 Pulse Sequence s2pul
6 Experiment 1D
7 Probe OneP
8 Number of Scans 164
9 Receiver Gain 30
10 Relaxation Delay 1.0000
11 Pulse Width 5.9500
12 Acquisition Time 0.8651
13 Acquisition Date 2025-10-10T14:09:32
14 Spectrometer Frequency 150.85
15 Spectral Width 37878.8
16 Lowest Frequency -2748.6
17 Acquired Size 32768
18 Spectral Size 65536
f1 (ppm)

hqq-s3-129-down-H

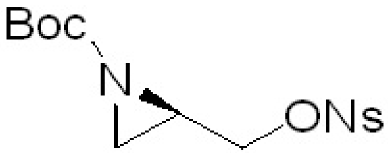

8

	Parameter	Value
1	Origin	Varian
2	Spectrometer	vnmrs
3	Solvent	$CDCl_3$
4	Temperature	25.0
5	Pulse Sequence	s2pul
6	Experiment	1D
7	Probe	OneP
8	Number of Scans	8
9	Receiver Gain	52
10	Relaxation Delay	1.0000
11	Pulse Width	5.8500
12	Acquisition Time	1.7039
13	Acquisition Date	2025-10-28T21:16:59
14	Spectrometer Frequency	599.84
15	Spectral Width	9615.4
16	Lowest Frequency	-1208.6
17	Acquired Size	16384
18	Spectral Size	65536

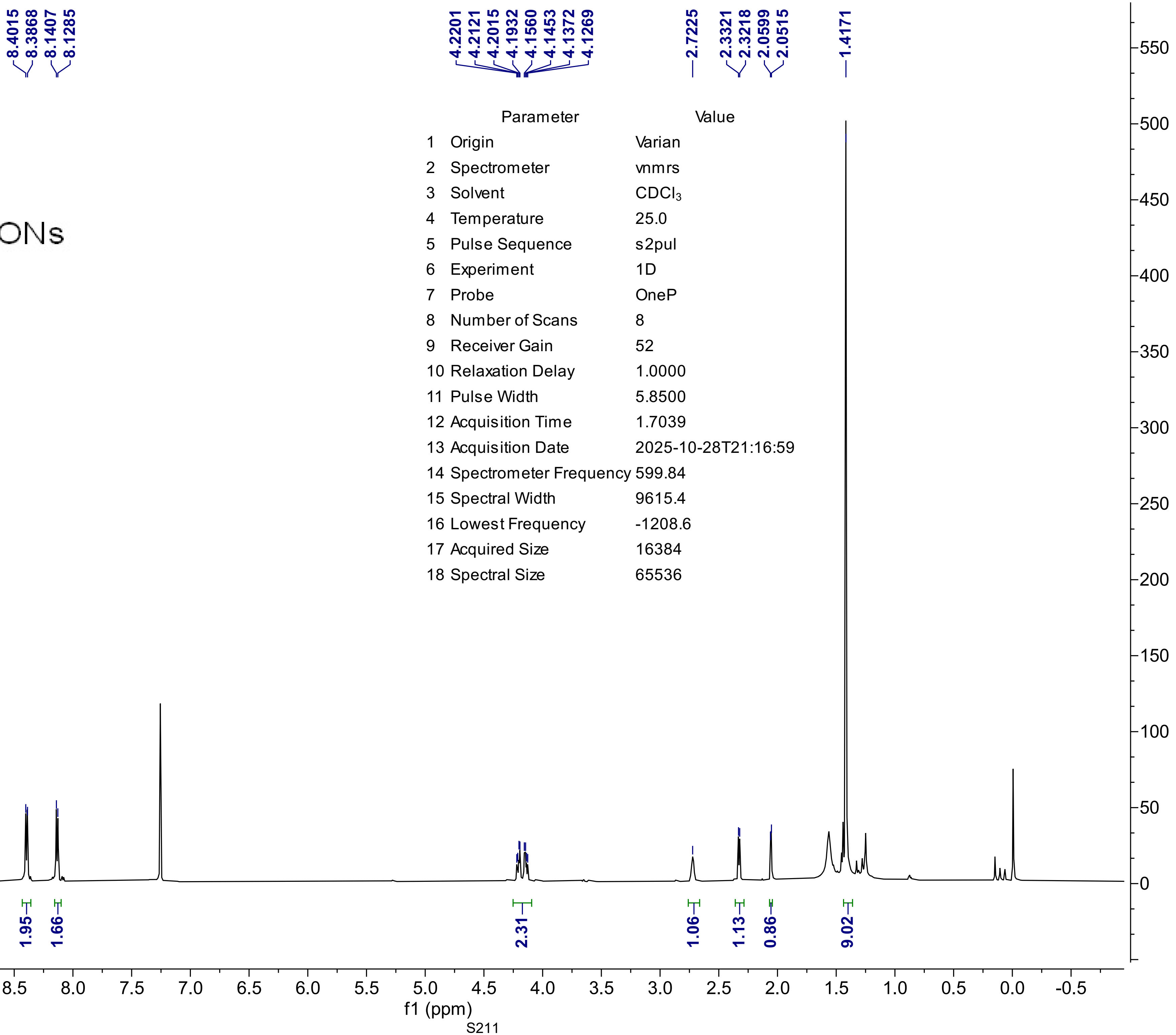

hqq-s3-98-2-H-600M

Selective band center: 6.87 (ppm); width: 28.7 (Hz)

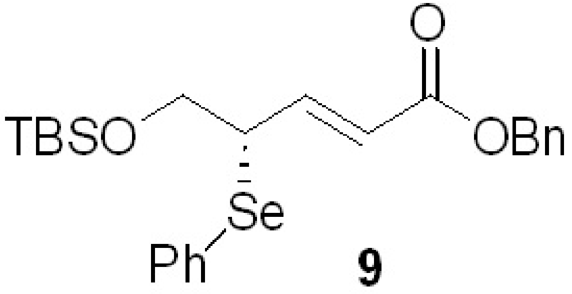

9

	Parameter	Value
1	Origin	Varian
2	Spectrometer	vnmrs
3	Solvent	$CDCl_3$
4	Temperature	25.0
5	Pulse Sequence	s2pul
6	Experiment	1D
7	Probe	OneP
8	Number of Scans	8
9	Receiver Gain	30
10	Relaxation Delay	1.0000
11	Pulse Width	5.8500
12	Acquisition Time	1.7039
13	Acquisition Date	2025-10-10T13:41:21
14	Spectrometer Frequency	599.84
15	Spectral Width	9615.4
16	Lowest Frequency	-1196.1
17	Acquired Size	16384
18	Spectral Size	65536

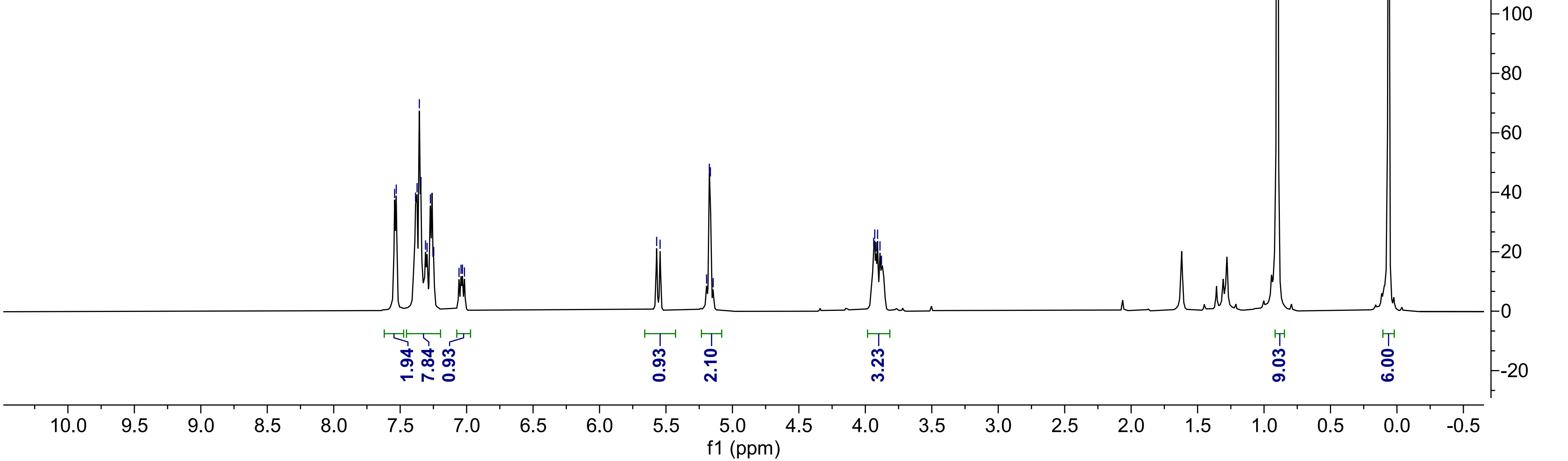

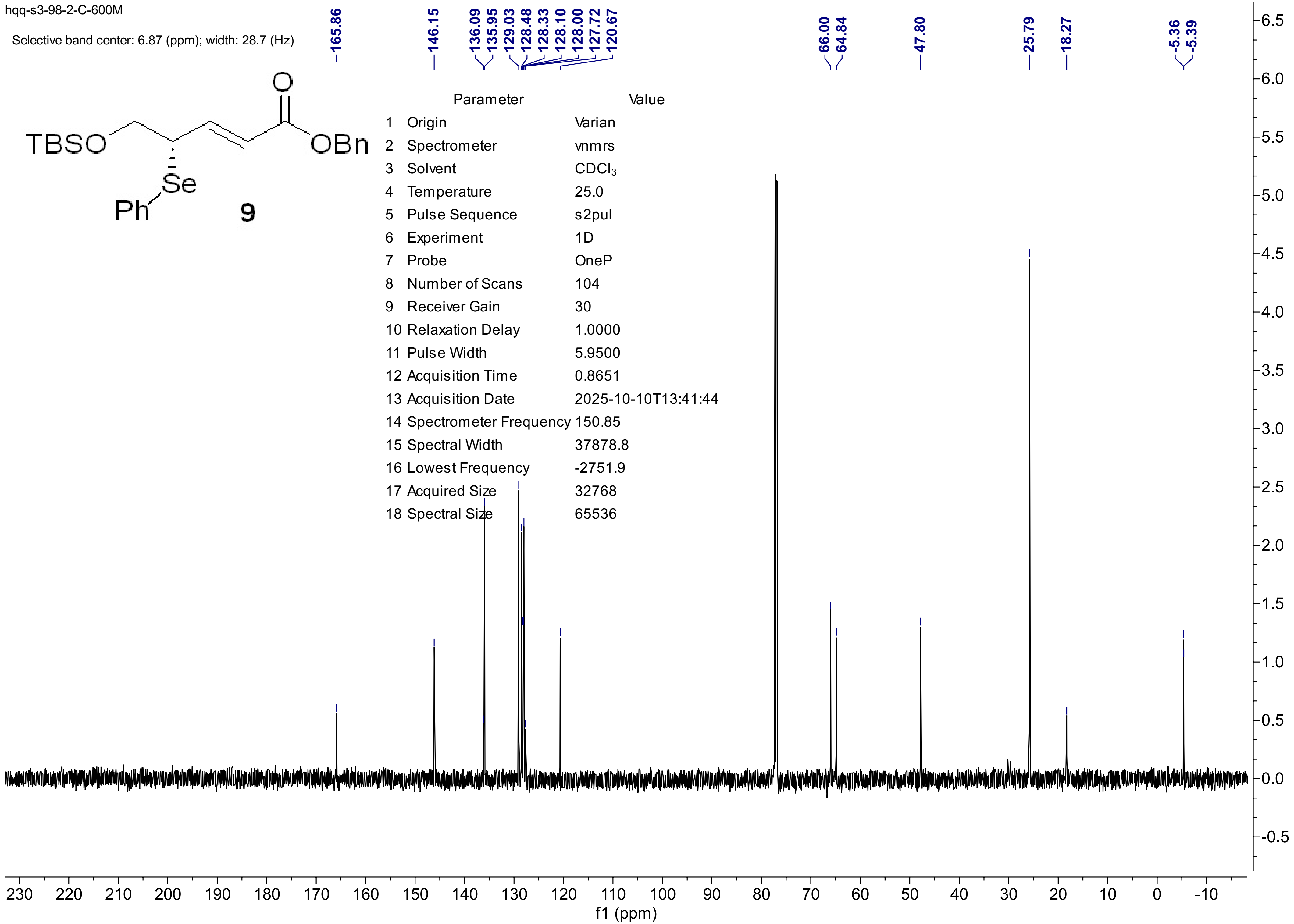
hqq-s3-98-2-C-600M
Selective band center: 6.87 (ppm); width: 28.7 (Hz)
165.86
146.15
136.09
135.95
129.03
128.48
128.33
128.10
128.00
127.72
120.67
66.00
64.84
47.80
25.79
18.27
-5.36
-5.39
TBSO
OBn
O
Se
Ph
9
Parameter Value
1 Origin Varian
2 Spectrometer vnmrs
3 Solvent $CDCl_3$
4 Temperature 25.0
5 Pulse Sequence s2pul
6 Experiment 1D
7 Probe OneP
8 Number of Scans 104
9 Receiver Gain 30
10 Relaxation Delay 1.0000
11 Pulse Width 5.9500
12 Acquisition Time 0.8651
13 Acquisition Date 2025-10-10T13:41:44
14 Spectrometer Frequency 150.85
15 Spectral Width 37878.8
16 Lowest Frequency -2751.9
17 Acquired Size 32768
18 Spectral Size 65536
f1 (ppm)

hqq-s3-sth-54-H-600M

Selective band center: 6.87 (ppm); width: 28.7 (Hz)

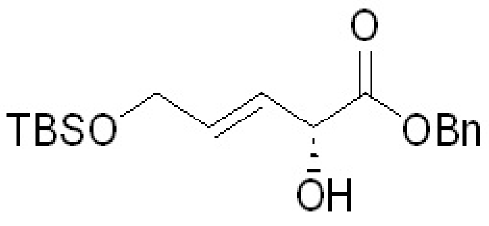

10

	Parameter	Value
1	Origin	Varian
2	Spectrometer	vnmrs
3	Solvent	$CDCl_3$
4	Temperature	25.0
5	Pulse Sequence	s2pul
6	Experiment	1D
7	Probe	OneP
8	Number of Scans	8
9	Receiver Gain	30
10	Relaxation Delay	1.0000
11	Pulse Width	5.8500
12	Acquisition Time	1.7039
13	Acquisition Date	2025-10-10T13:50:39
14	Spectrometer Frequency	599.84
15	Spectral Width	9615.4
16	Lowest Frequency	-1206.1
17	Acquired Size	16384
18	Spectral Size	65536

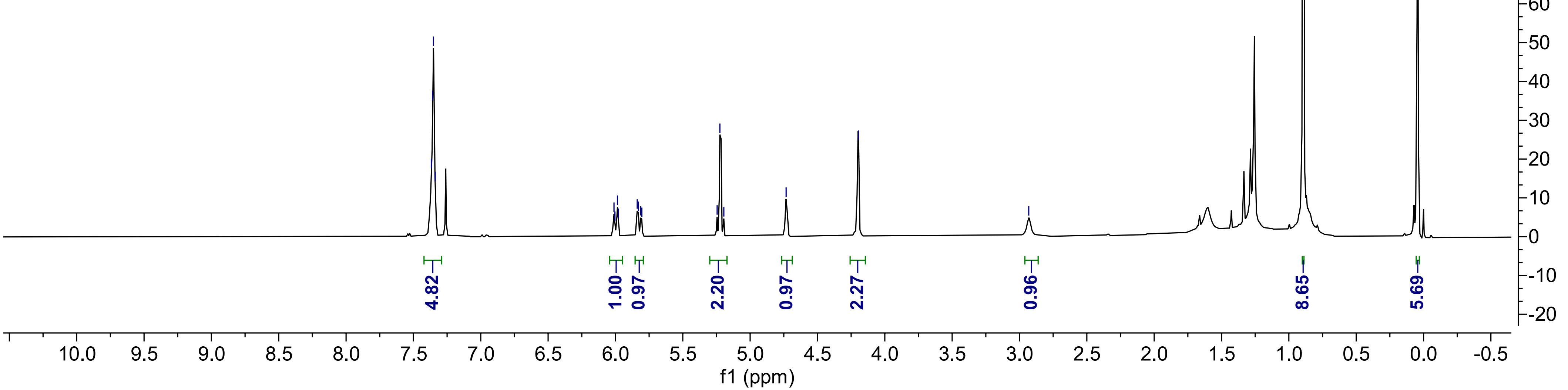

hqq-s3-sth-54-C-600M

Selective band center: 6.87 (ppm); width 28.7 (Hz)

10

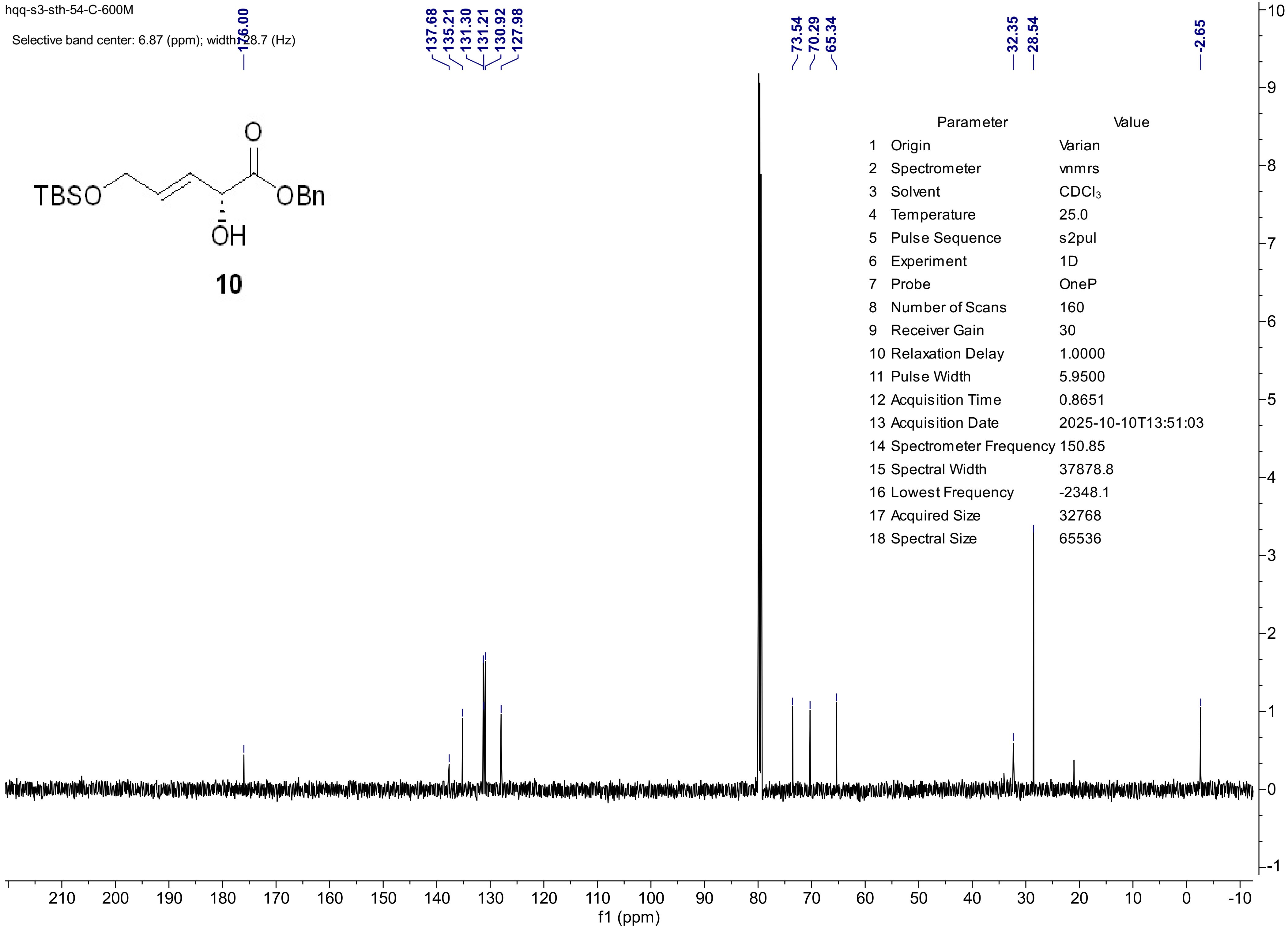

	Parameter	Value
1	Origin	Varian
2	Spectrometer	vnmrs
3	Solvent	$CDCl_3$
4	Temperature	25.0
5	Pulse Sequence	s2pul
6	Experiment	1D
7	Probe	OneP
8	Number of Scans	160
9	Receiver Gain	30
10	Relaxation Delay	1.0000
11	Pulse Width	5.9500
12	Acquisition Time	0.8651
13	Acquisition Date	2025-10-10T13:51:03
14	Spectrometer Frequency	150.85
15	Spectral Width	37878.8
16	Lowest Frequency	-2348.1
17	Acquired Size	32768
18	Spectral Size	65536

HQQ-122-UP-H

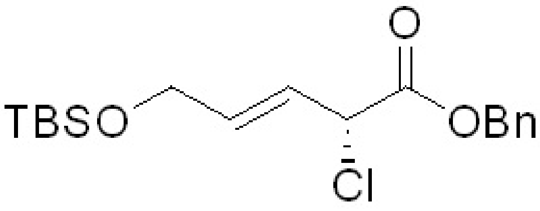

11

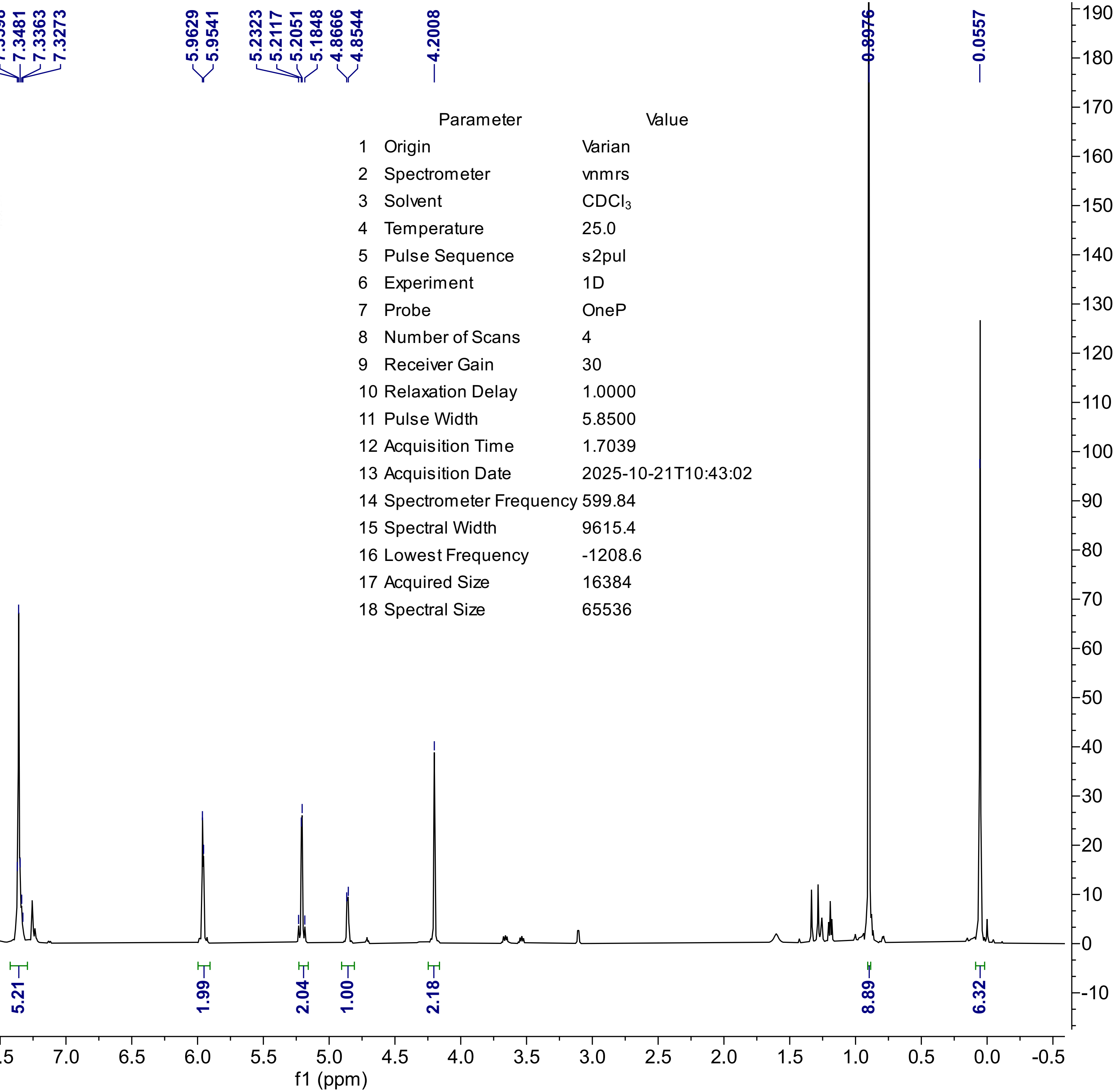

	Parameter	Value
1	Origin	Varian
2	Spectrometer	vnmrs
3	Solvent	$CDCl_3$
4	Temperature	25.0
5	Pulse Sequence	s2pul
6	Experiment	1D
7	Probe	OneP
8	Number of Scans	4
9	Receiver Gain	30
10	Relaxation Delay	1.0000
11	Pulse Width	5.8500
12	Acquisition Time	1.7039
13	Acquisition Date	2025-10-21T10:43:02
14	Spectrometer Frequency	599.84
15	Spectral Width	9615.4
16	Lowest Frequency	-1208.6
17	Acquired Size	16384
18	Spectral Size	65536

HQQ-122-UP-c-1

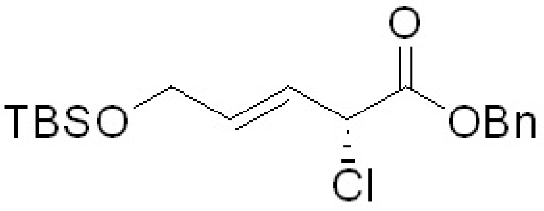

11

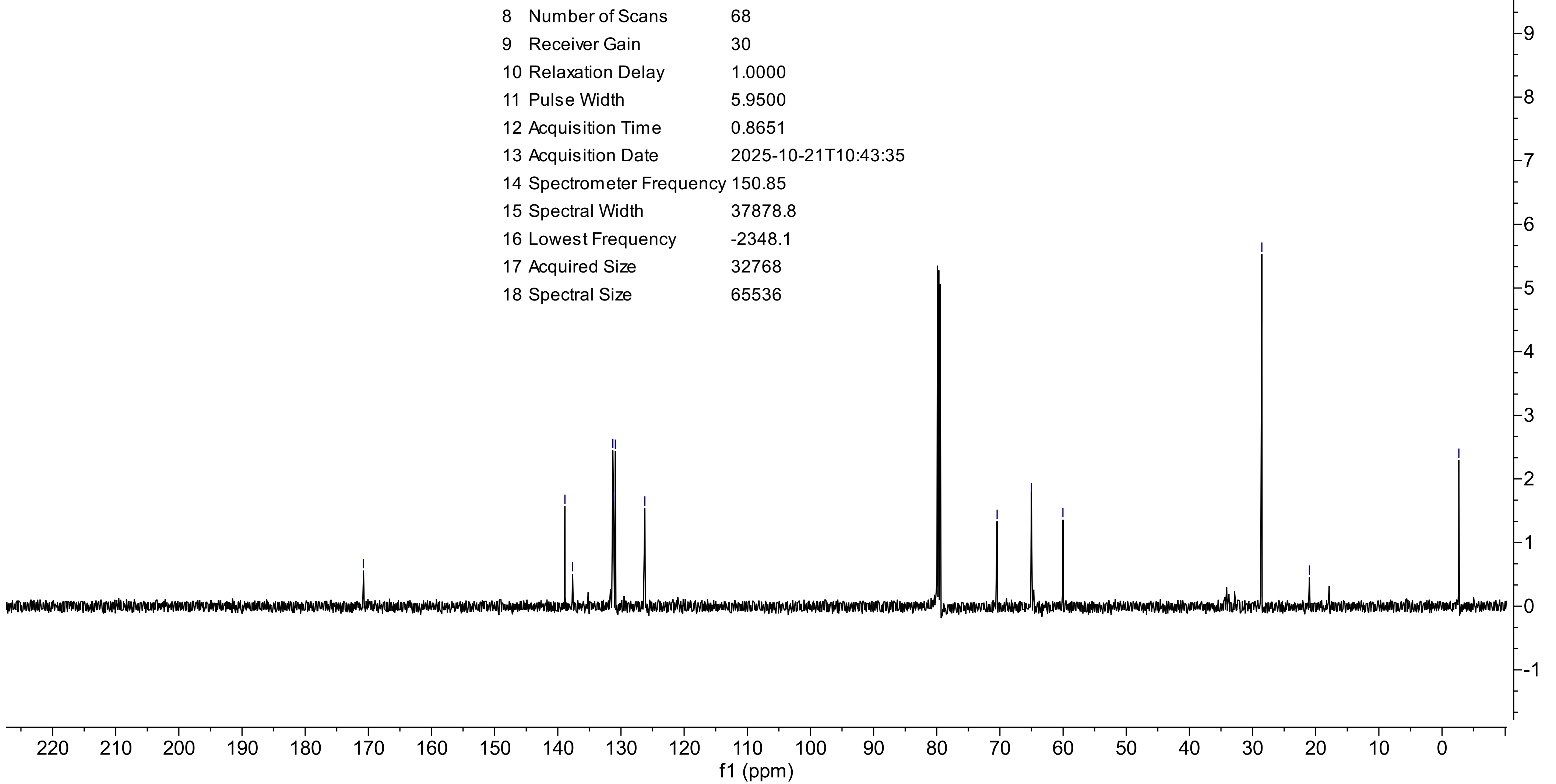

	Parameter	Value
1	Origin	Varian
2	Spectrometer	vnmrs
3	Solvent	$CDCl_3$
4	Temperature	25.0
5	Pulse Sequence	s2pul
6	Experiment	1D
7	Probe	OneP
8	Number of Scans	68
9	Receiver Gain	30
10	Relaxation Delay	1.0000
11	Pulse Width	5.9500
12	Acquisition Time	0.8651
13	Acquisition Date	2025-10-21T10:43:35
14	Spectrometer Frequency	150.85
15	Spectral Width	37878.8
16	Lowest Frequency	-2348.1
17	Acquired Size	32768
18	Spectral Size	65536

CJX-3-128-H-400M
Gradient Shimming

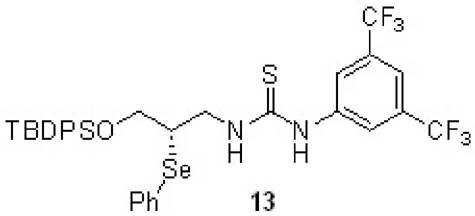

13

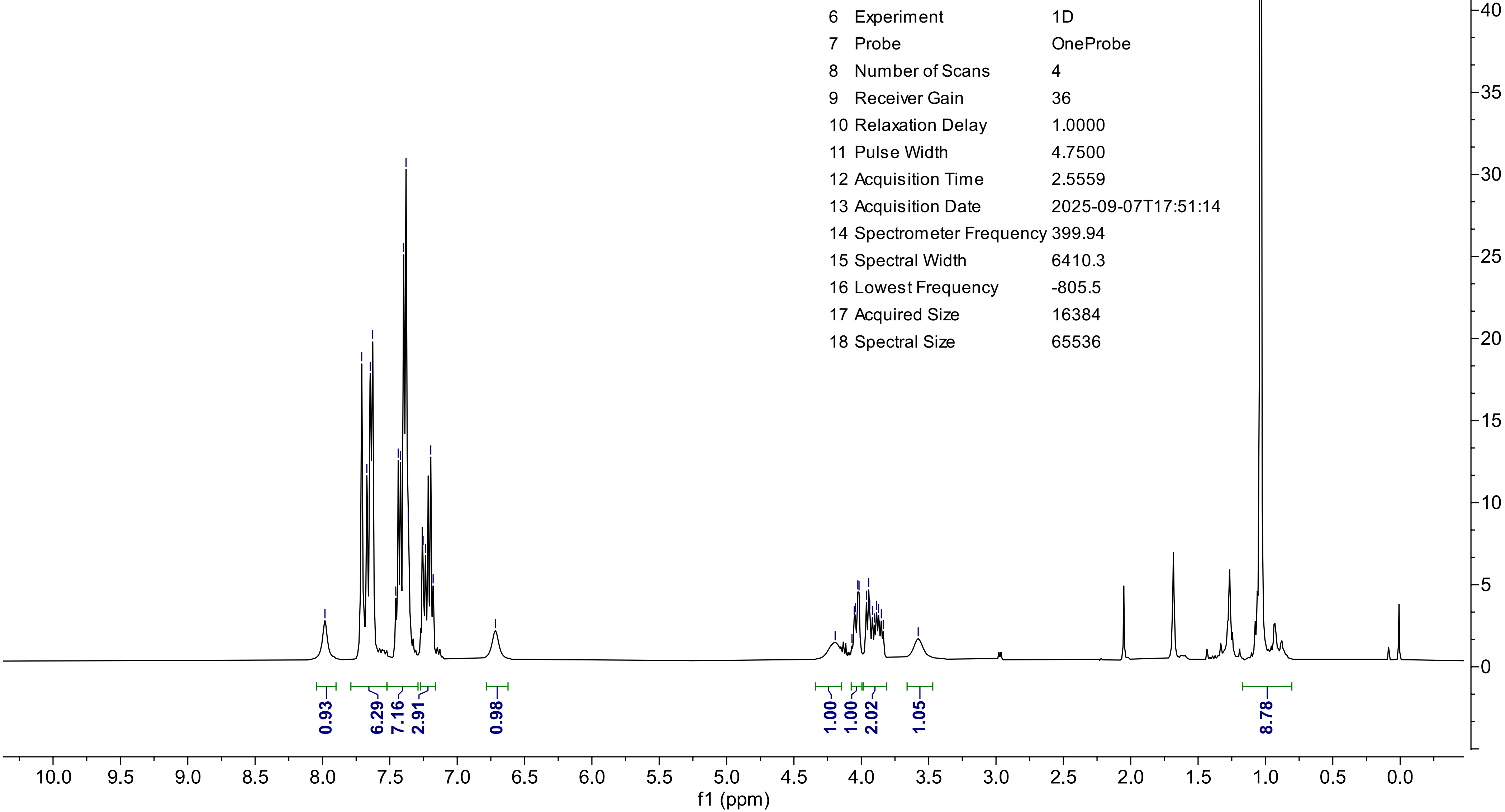

	Parameter	Value
1	Origin	Varian
2	Spectrometer	vnmrs
3	Solvent	$CDCl_3$
4	Temperature	25.0
5	Pulse Sequence	s2pul
6	Experiment	1D
7	Probe	OneProbe
8	Number of Scans	4
9	Receiver Gain	36
10	Relaxation Delay	1.0000
11	Pulse Width	4.7500
12	Acquisition Time	2.5559
13	Acquisition Date	2025-09-07T17:51:14
14	Spectrometer Frequency	399.94
15	Spectral Width	6410.3
16	Lowest Frequency	-805.5
17	Acquired Size	16384
18	Spectral Size	65536

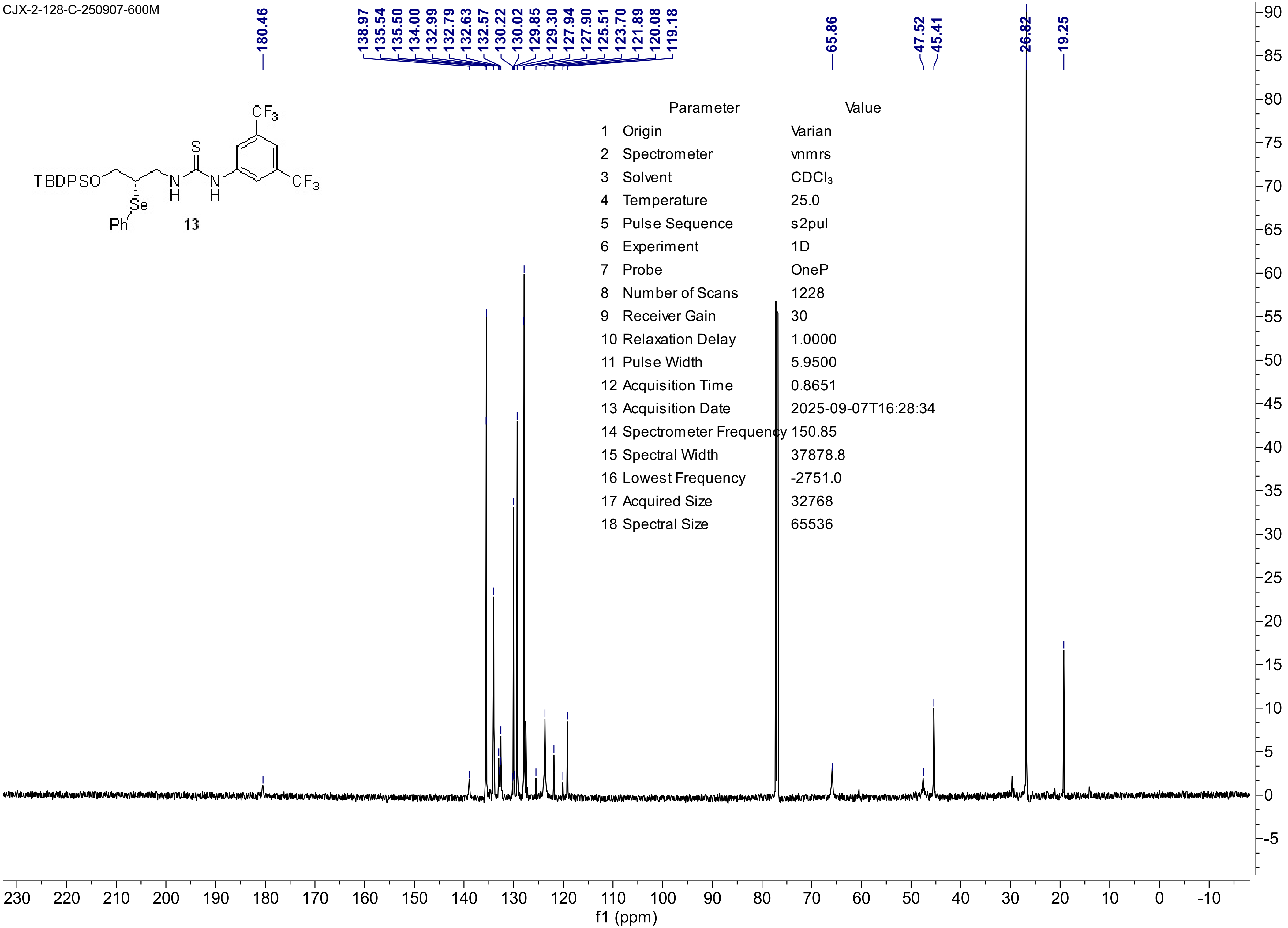
CJX-2-128-C-250907-600M
180.46
138.97
135.54
135.50
134.00
132.99
132.79
132.63
132.57
130.22
130.02
129.85
129.30
127.94
127.90
125.51
123.70
121.89
120.08
119.18
65.86
47.52
45.41
26.82
19.25
TBDPSO
S
N
H
N
H
CF3
CF3
Se
Ph
13
Parameter Value
1 Origin Varian
2 Spectrometer vnmrs
3 Solvent CDCl3
4 Temperature 25.0
5 Pulse Sequence s2pul
6 Experiment 1D
7 Probe OneP
8 Number of Scans 1228
9 Receiver Gain 30
10 Relaxation Delay 1.0000
11 Pulse Width 5.9500
12 Acquisition Time 0.8651
13 Acquisition Date 2025-09-07T16:28:34
14 Spectrometer Frequency 150.85
15 Spectral Width 37878.8
16 Lowest Frequency -2751.0
17 Acquired Size 32768
18 Spectral Size 65536
f1 (ppm)

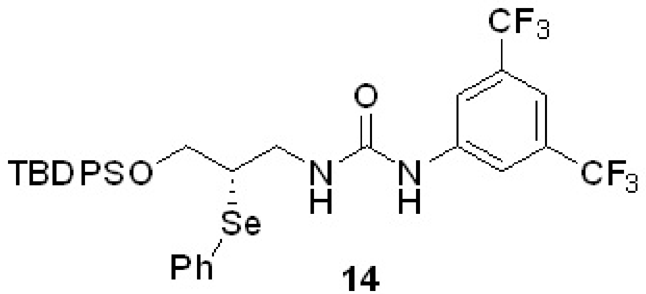

14

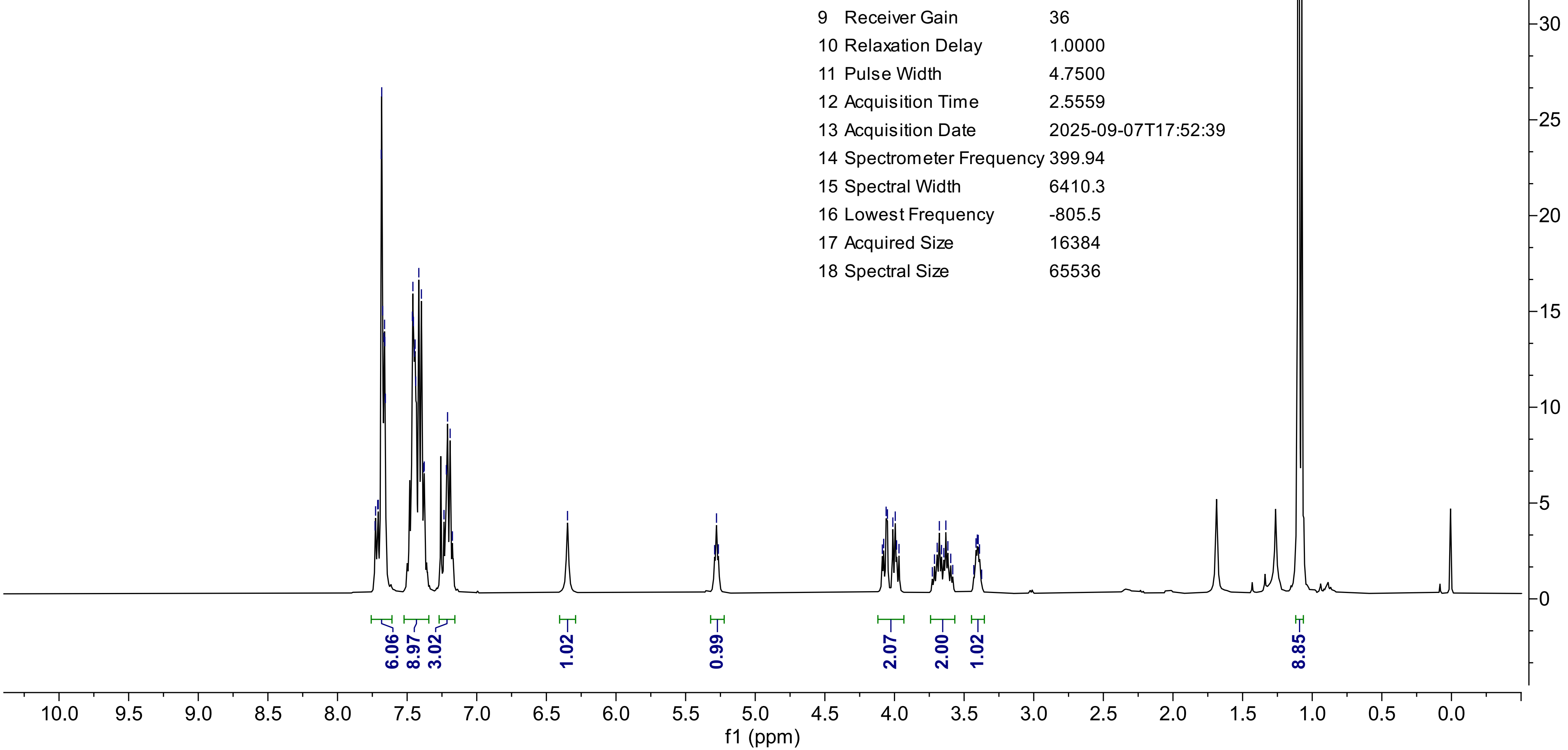

	Parameter	Value
1	Origin	Varian
2	Spectrometer	vnmrs
3	Solvent	$CDCl_3$
4	Temperature	25.0
5	Pulse Sequence	s2pul
6	Experiment	1D
7	Probe	OneProbe
8	Number of Scans	4
9	Receiver Gain	36
10	Relaxation Delay	1.0000
11	Pulse Width	4.7500
12	Acquisition Time	2.5559
13	Acquisition Date	2025-09-07T17:52:39
14	Spectrometer Frequency	399.94
15	Spectral Width	6410.3
16	Lowest Frequency	-805.5
17	Acquired Size	16384
18	Spectral Size	65536

CJX-2-129-C-250907-600M

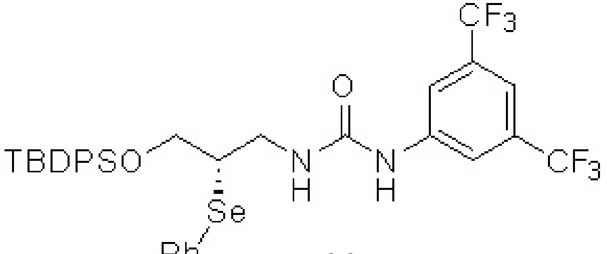

14

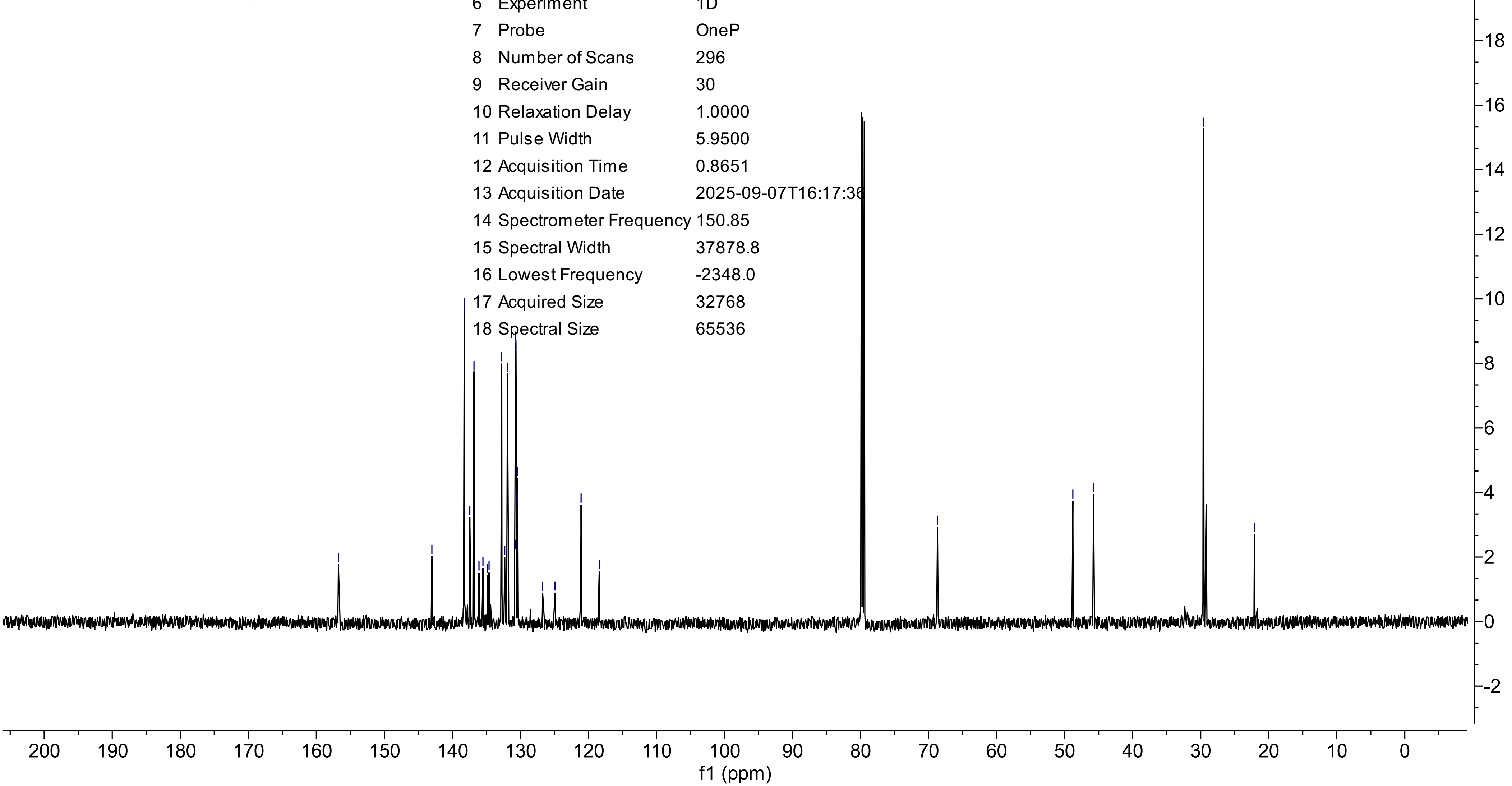

	Parameter	Value
1	Origin	Varian
2	Spectrometer	vnmrs
3	Solvent	$CDCl_3$
4	Temperature	25.0
5	Pulse Sequence	s2pul
6	Experiment	1D
7	Probe	OneP
8	Number of Scans	296
9	Receiver Gain	30
10	Relaxation Delay	1.0000
11	Pulse Width	5.9500
12	Acquisition Time	0.8651
13	Acquisition Date	2025-09-07T16:17:36
14	Spectrometer Frequency	150.85
15	Spectral Width	37878.8
16	Lowest Frequency	-2348.0
17	Acquired Size	32768
18	Spectral Size	65536

HQQ-135-1-H-11.12
Gradient Shimming

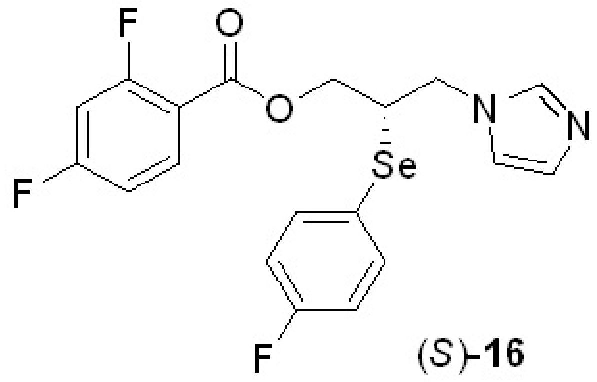

(*S*)-**16**

	Parameter	Value
1	Origin	Varian
2	Spectrometer	vnmrs
3	Solvent	$CDCl_3$
4	Temperature	25.0
5	Pulse Sequence	s2pul
6	Experiment	1D
7	Probe	OneProbe
8	Number of Scans	1
9	Receiver Gain	34
10	Relaxation Delay	1.0000
11	Pulse Width	4.7500
12	Acquisition Time	2.5559
13	Acquisition Date	2025-11-12T21:40:45
14	Spectrometer Frequency	399.94
15	Spectral Width	6410.3
16	Lowest Frequency	-804.7
17	Acquired Size	16384
18	Spectral Size	65536

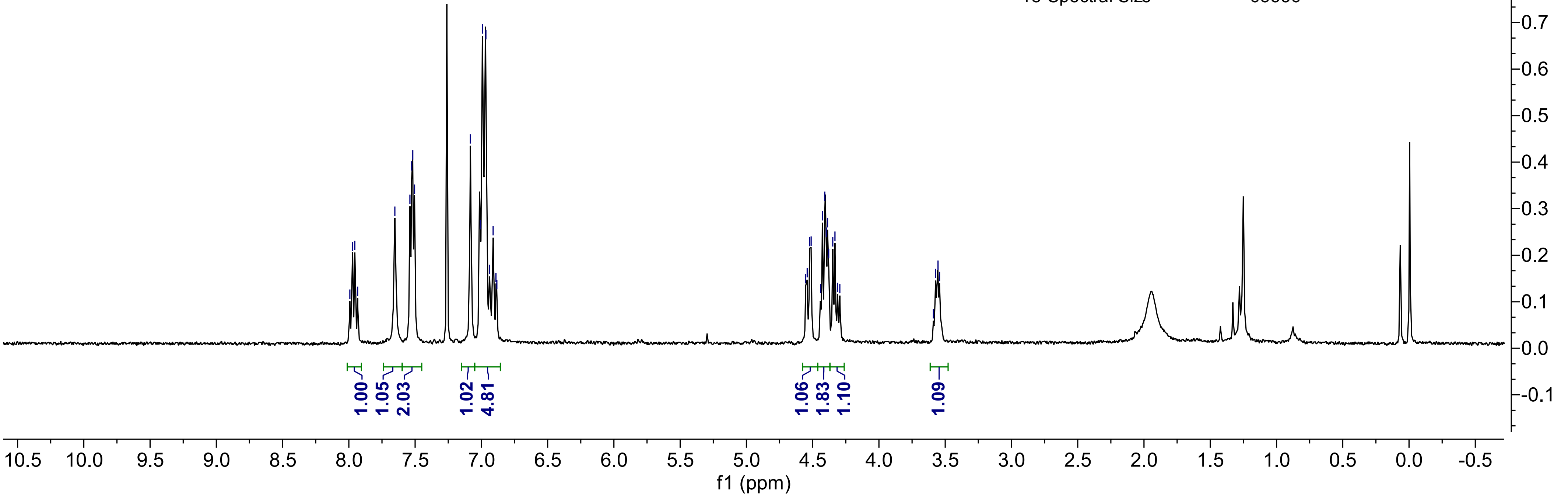

hqq-s3-135-C-600M-5

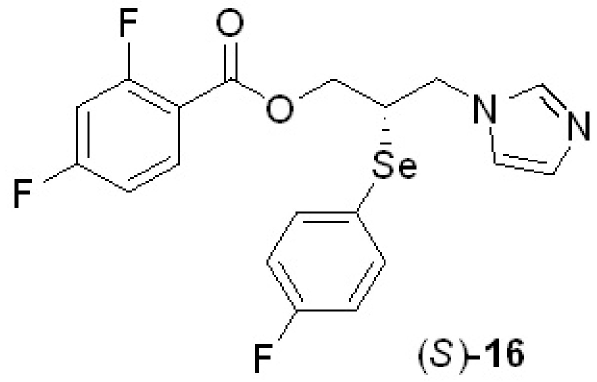

(*S*)-**16**

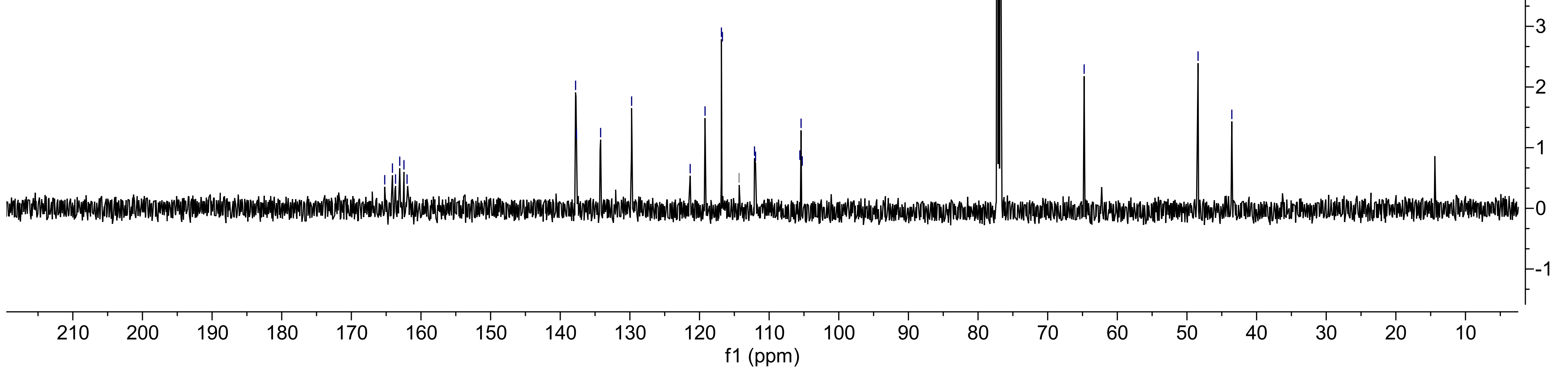

	Parameter	Value
1	Origin	Varian
2	Spectrometer	vnmrs
3	Solvent	$CDCl_3$
4	Temperature	25.0
5	Pulse Sequence	s2pul
6	Experiment	1D
7	Probe	OneP
8	Number of Scans	540
9	Receiver Gain	30
10	Relaxation Delay	1.0000
11	Pulse Width	5.9500
12	Acquisition Time	0.8651
13	Acquisition Date	2025-11-08T16:36:06
14	Spectrometer Frequency	150.85
15	Spectral Width	37878.8
16	Lowest Frequency	-2751.0
17	Acquired Size	32768
18	Spectral Size	65536

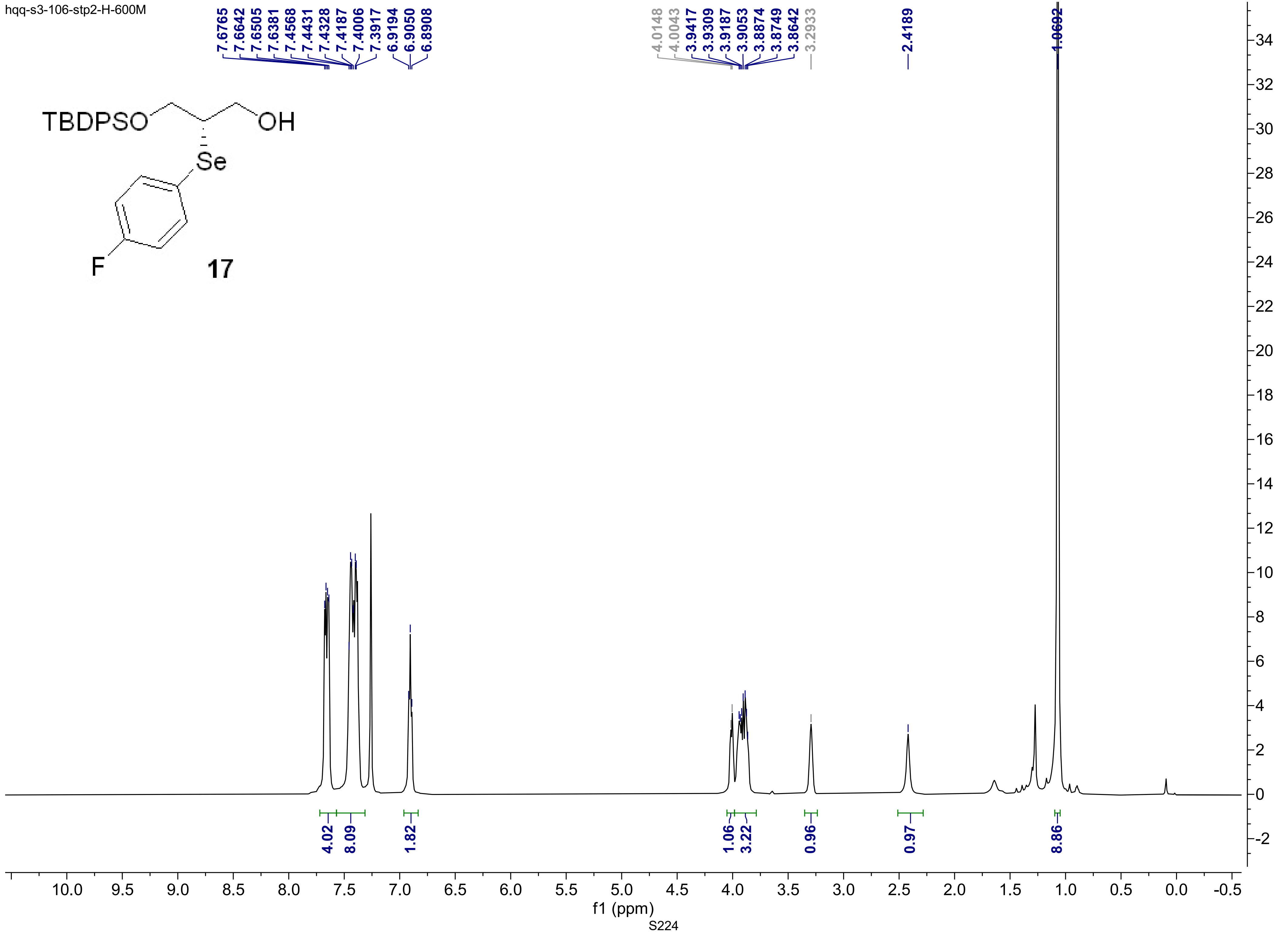
hqq-s3-106-stp2-H-600M
7.6765
7.6642
7.6505
7.6381
7.4568
7.4431
7.4328
7.4187
7.4006
7.3917
6.9194
6.9050
6.8908
4.0148
4.0043
3.9417
3.9309
3.9187
3.9053
3.8874
3.8749
3.8642
3.2933
2.4189
1.0692
TBDPSO
OH
Se
F
17
4.02
8.09
1.82
1.06
3.22
0.96
0.97
8.86
f1 (ppm)

hqq-s3-106-stp2-C-600M

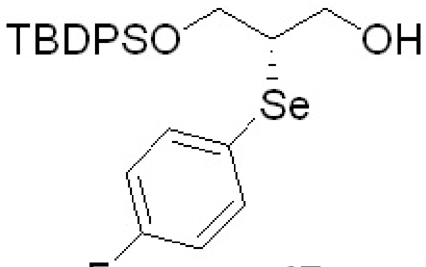

17

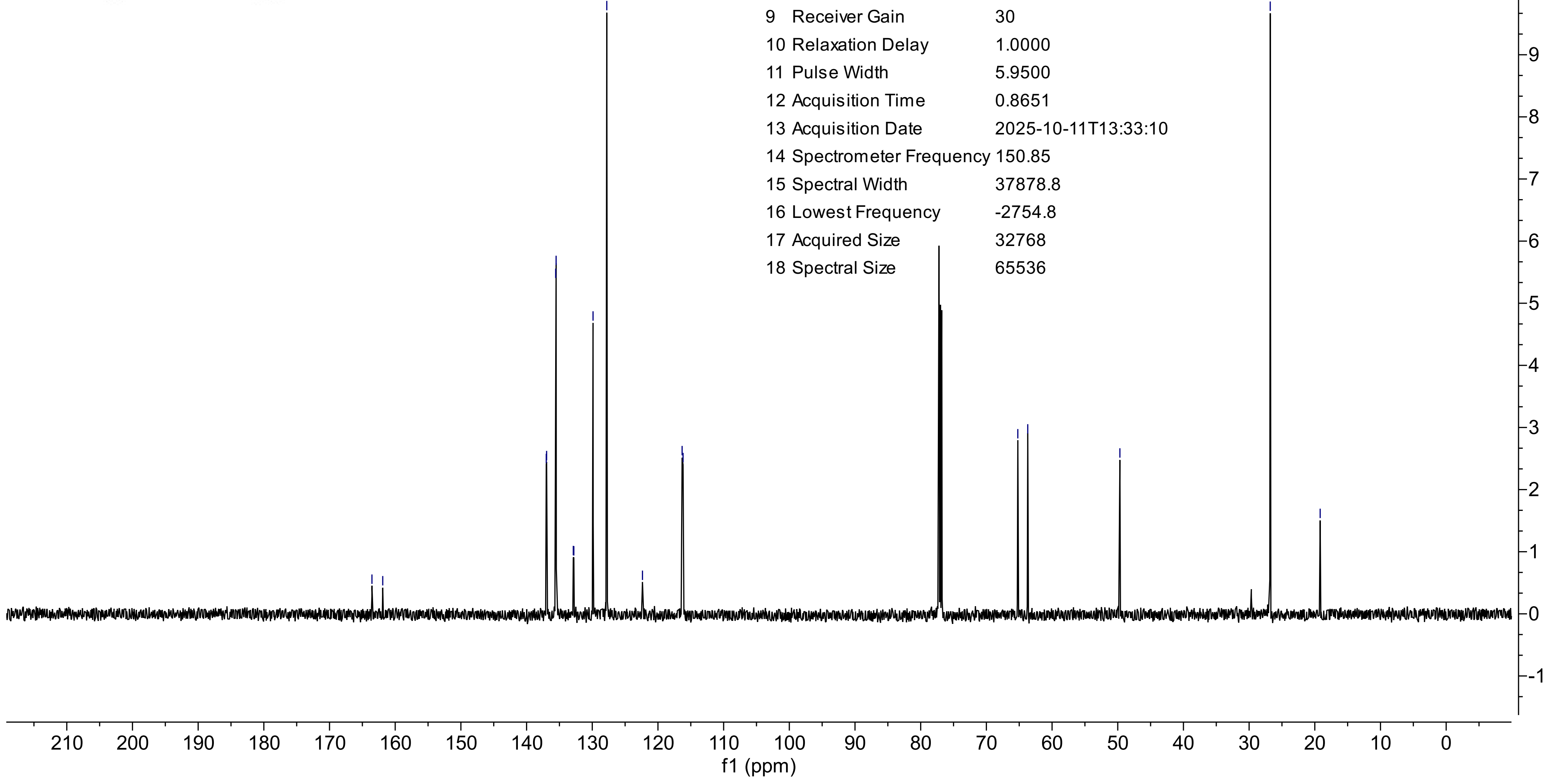

	Parameter	Value
1	Origin	Varian
2	Spectrometer	vnmrs
3	Solvent	$CDCl_3$
4	Temperature	25.0
5	Pulse Sequence	s2pul
6	Experiment	1D
7	Probe	OneP
8	Number of Scans	72
9	Receiver Gain	30
10	Relaxation Delay	1.0000
11	Pulse Width	5.9500
12	Acquisition Time	0.8651
13	Acquisition Date	2025-10-11T13:33:10
14	Spectrometer Frequency	150.85
15	Spectral Width	37878.8
16	Lowest Frequency	-2754.8
17	Acquired Size	32768
18	Spectral Size	65536

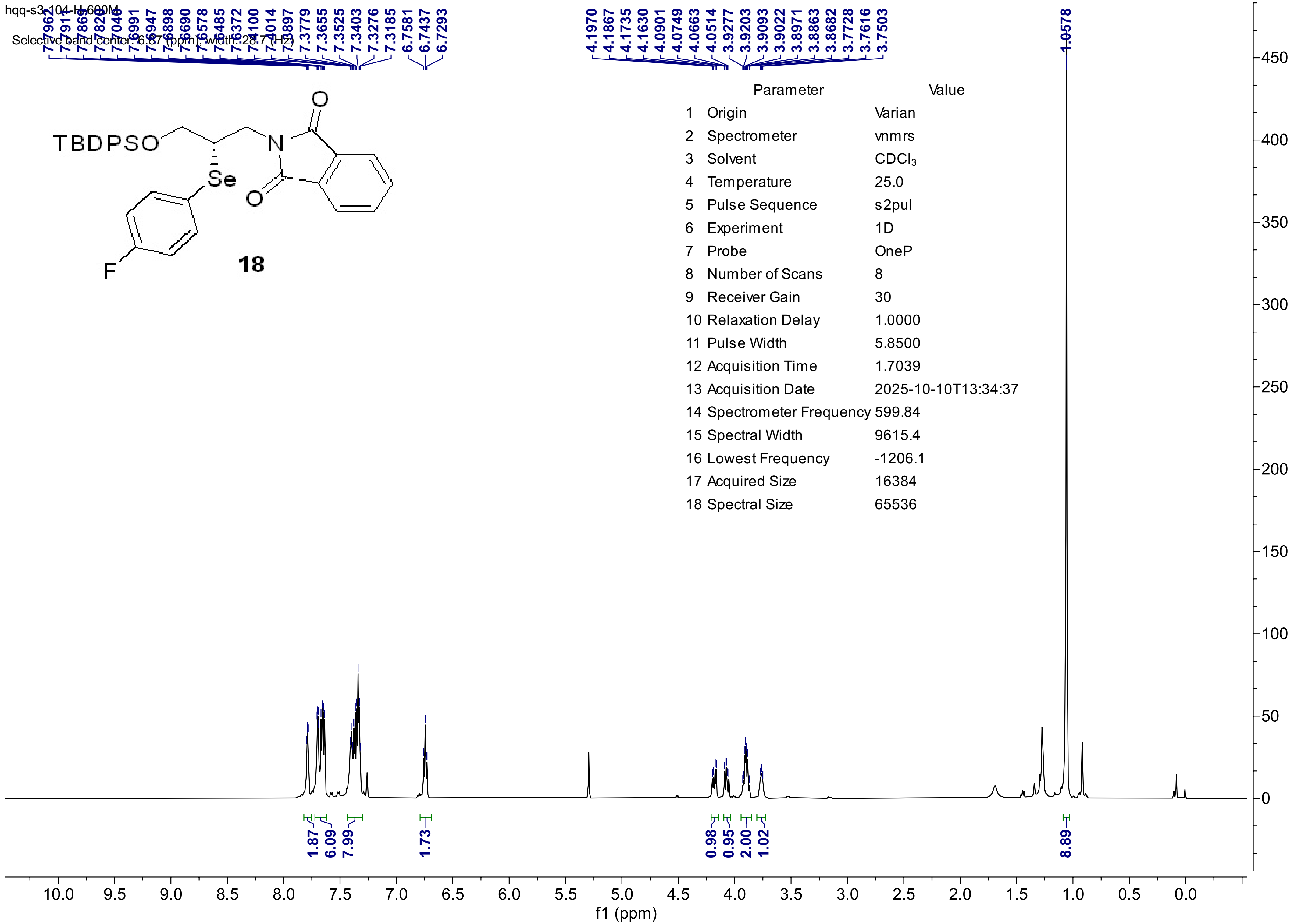
hqq-s3-104-H-600M
Selective band center: 6.87 (ppm), width: 28.7 (Hz)
7.7962 7.7911 7.7869 7.7820 7.7040 7.6991 7.6947 7.6898 7.6690 7.6578 7.6485 7.6372 7.4100 7.4014 7.3897 7.3779 7.3655 7.3525 7.3403 7.3276 7.3185 6.7581 6.7437 6.7293
4.1970 4.1867 4.1735 4.1630 4.0901 4.0749 4.0663 4.0514 3.9277 3.9203 3.9093 3.9022 3.8971 3.8863 3.8682 3.7728 3.7616 3.7503
1.0578
TBDPSO
N
O
O
Se
F
18
Parameter Value
1 Origin Varian
2 Spectrometer vnmrs
3 Solvent CDCl3
4 Temperature 25.0
5 Pulse Sequence s2pul
6 Experiment 1D
7 Probe OneP
8 Number of Scans 8
9 Receiver Gain 30
10 Relaxation Delay 1.0000
11 Pulse Width 5.8500
12 Acquisition Time 1.7039
13 Acquisition Date 2025-10-10T13:34:37
14 Spectrometer Frequency 599.84
15 Spectral Width 9615.4
16 Lowest Frequency -1206.1
17 Acquired Size 16384
18 Spectral Size 65536
1.87 6.09 7.99 1.73 0.98 0.95 2.00 1.02 8.89
f1 (ppm)

hqq-s3-104-C-600M

Selective band center: 6.87 (ppm); width: 28.7 (Hz)

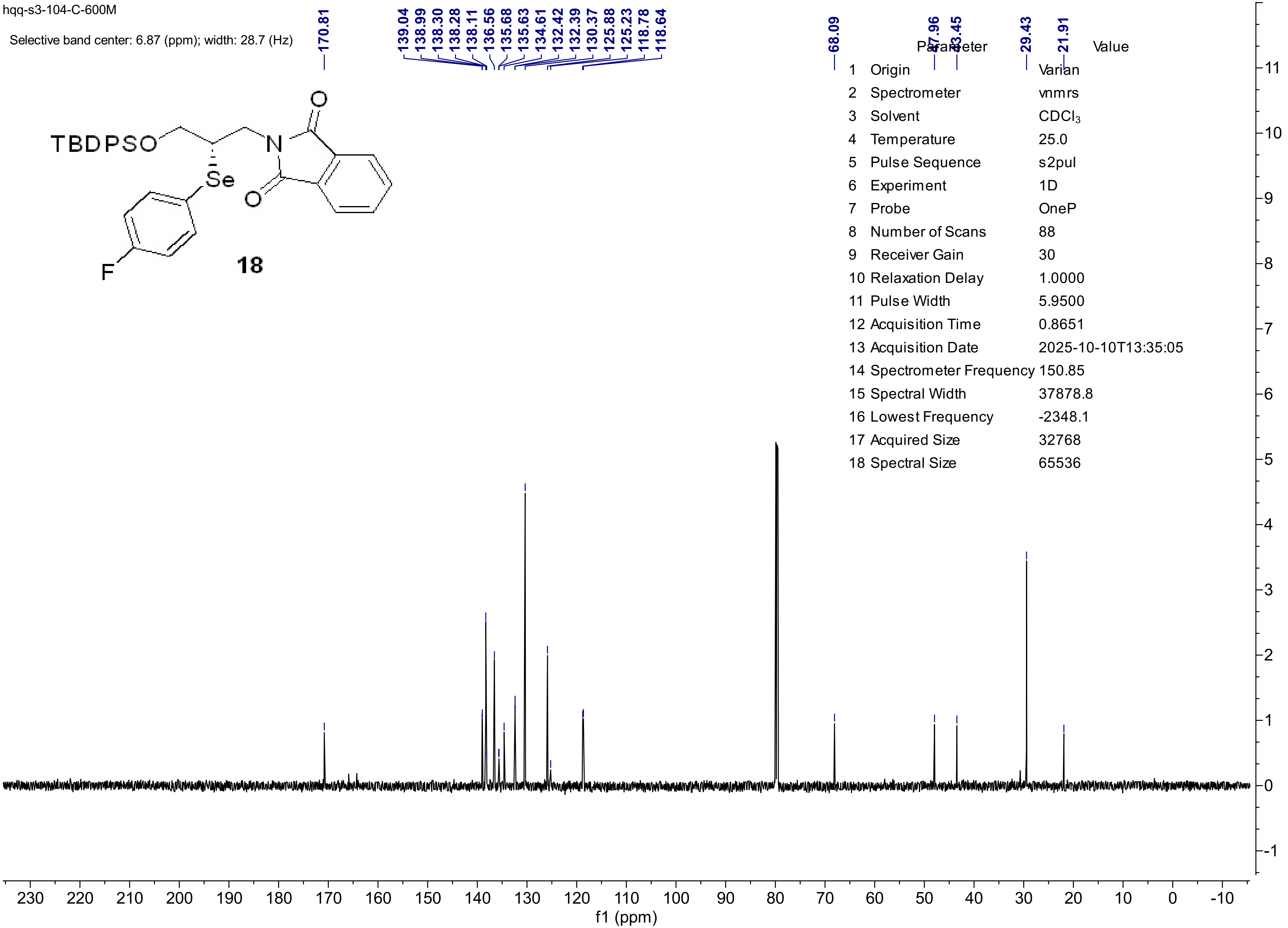

	Parameter	Value
1	Origin	Varian
2	Spectrometer	vnmrs
3	Solvent	$CDCl_3$
4	Temperature	25.0
5	Pulse Sequence	s2pul
6	Experiment	1D
7	Probe	OneP
8	Number of Scans	88
9	Receiver Gain	30
10	Relaxation Delay	1.0000
11	Pulse Width	5.9500
12	Acquisition Time	0.8651
13	Acquisition Date	2025-10-10T13:35:05
14	Spectrometer Frequency	150.85
15	Spectral Width	37878.8
16	Lowest Frequency	-2348.1
17	Acquired Size	32768
18	Spectral Size	65536

Single Injection Report

Agilent

**Data file:**	MB-RAC-IB-1.0mL-10%		
**Sequence Name:**	SingleSample	**Project Name:**	CJX
**Sample name:**	MB-RAC-IB-1.0mL-10%1260	**Operator:**	SYSTEM (SYSTEM)
**Instrument:**		**Injection date:**	2025-07-23 15:25:50+08:00
**Inj. volume:**	5.000 µL	**Location:**	P1-A1
**Acq. method:**	10%-60min-1.0ml-5uL.amx	**Type:**	Sample
**Processing method:**	GC_LC area percent_DefaultMethod.pmx	**Sample amount:**	0.00
**Manually modified:**	None		

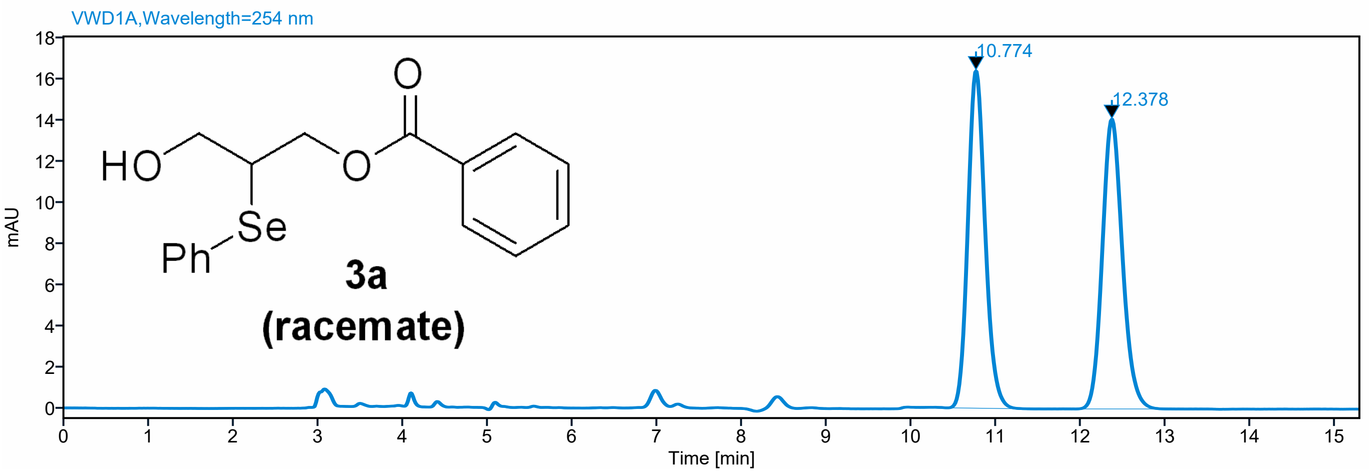

Signal: VWD1A,Wavelength=254 nm

RT [min]	Type	Width [min]	Area	Height	Area%
10.774	BB	0.93	232.20	16.41	49.94
12.378	BB	1.03	232.71	14.09	50.06
		**Sum**	**464.90**		

Single Injection Report

Agilent

**Data file:**	MB-ASY-IB-1.0mL-10%		
**Sequence Name:**	SingleSample	**Project Name:**	CJX
**Sample name:**	MB-ASY-IB-1.0mL-10%1260	**Operator:**	SYSTEM (SYSTEM)
**Instrument:**		**Injection date:**	2025-07-23 15:42:31+08:00
**Inj. volume:**	5.000 µL	**Location:**	P1-A2
**Acq. method:**	10%-60min-1.0ml-5uL.amx	**Type:**	Sample
**Processing method:**	GC_LC area percent_DefaultMethod.pmx	**Sample amount:**	0.00
**Manually modified:**	Manual Integration		

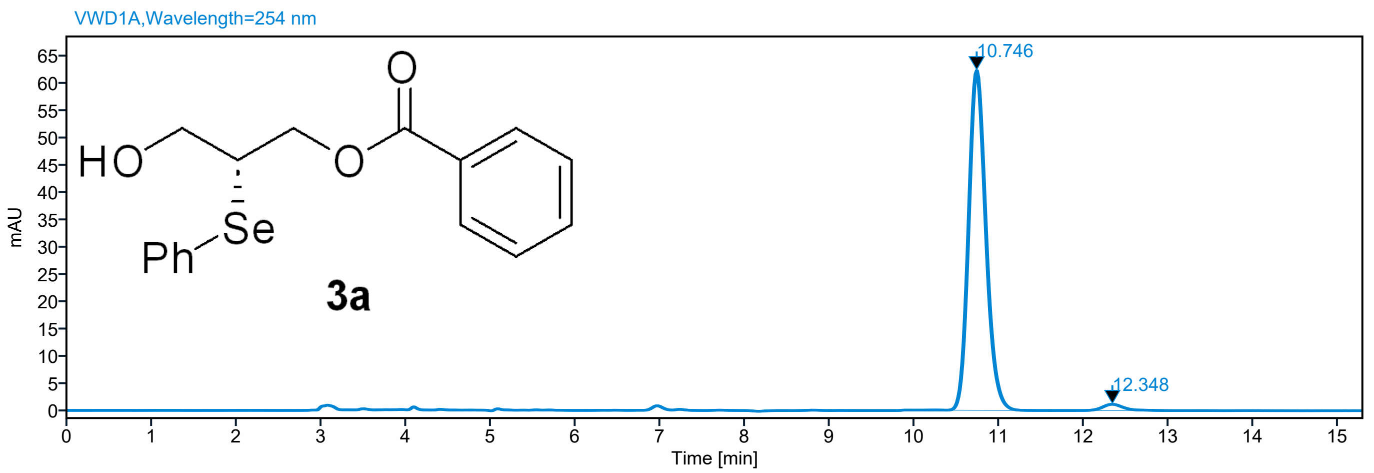

Signal: VWD1A,Wavelength=254 nm

RT [min]	Type	Width [min]	Area	Height	Area%
10.746	BB	0.95	884.42	62.23	97.93
12.348	MM m	0.83	18.70	1.13	2.07
		**Sum**	**903.11**		

Single Injection Report

Agilent

**Data file:**	CJX-3-131-ASY-IB-1.0-5%		
**Sequence Name:**	SingleSample	**Project Name:**	CJX
**Sample name:**	CJX-3-131-ASY-IB-1.0-5%	**Operator:**	SYSTEM (SYSTEM)
**Instrument:**	1260	**Injection date:**	2025-03-17 14:41:53+08:00
**Inj. volume:**	5.000 µL	**Location:**	P1-D1
**Acq. method:**	10%-30min-0.8ml-5uL.amx	**Type:**	Sample
**Processing method:**	GC_LC area percent_DefaultMethod.pmx	**Sample amount:**	0.00
**Manually modified:**	None		

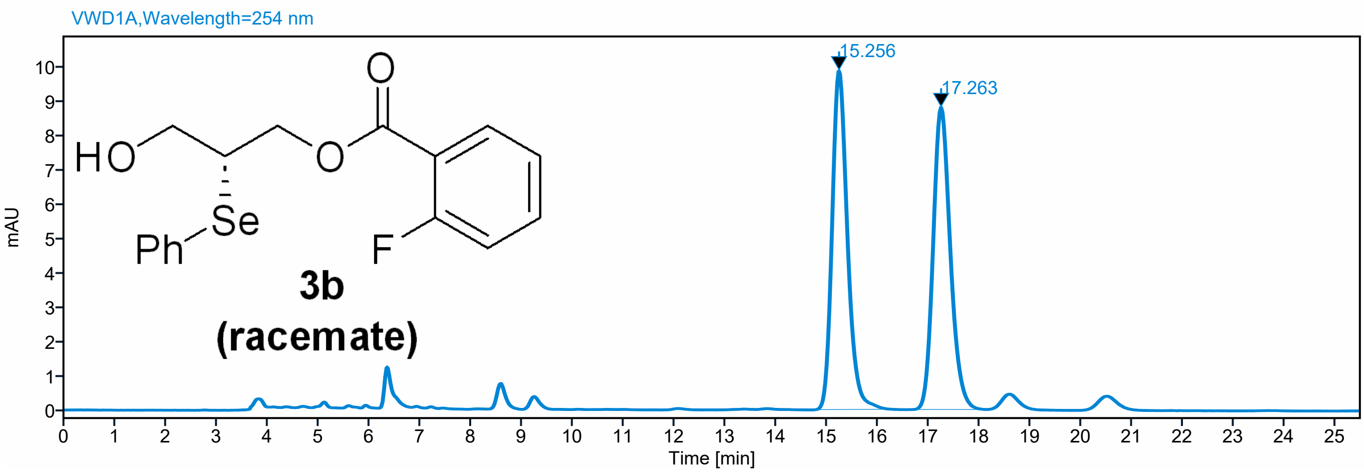

Signal: VWD1A,Wavelength=254 nm

RT [min]	Type	Width [min]	Area	Height	Area%
15.256	BB	1.47	206.92	9.87	50.06
17.263	BB	1.38	206.42	8.79	49.94
		**Sum**	**413.33**		

Single Injection Report

**Data file:**	CJX-3-131-ASY-IB-1.0-5%		
**Sequence Name:**	SingleSample	**Project Name:**	CJX
**Sample name:**	CJX-3-131-ASY-IB-1.0-5%	**Operator:**	SYSTEM (SYSTEM)
**Instrument:**	1260	**Injection date:**	2025-03-17 19:11:44+08:00
**Inj. volume:**	5.000 µL	**Location:**	P1-D2
**Acq. method:**	10%-25min-0.8ml-5uL.amx	**Type:**	Sample
**Processing method:**	GC_LC 面积百分比_DefaultMethod.pmx	**Sample amount:**	0.00
**Manually modified:**	None		

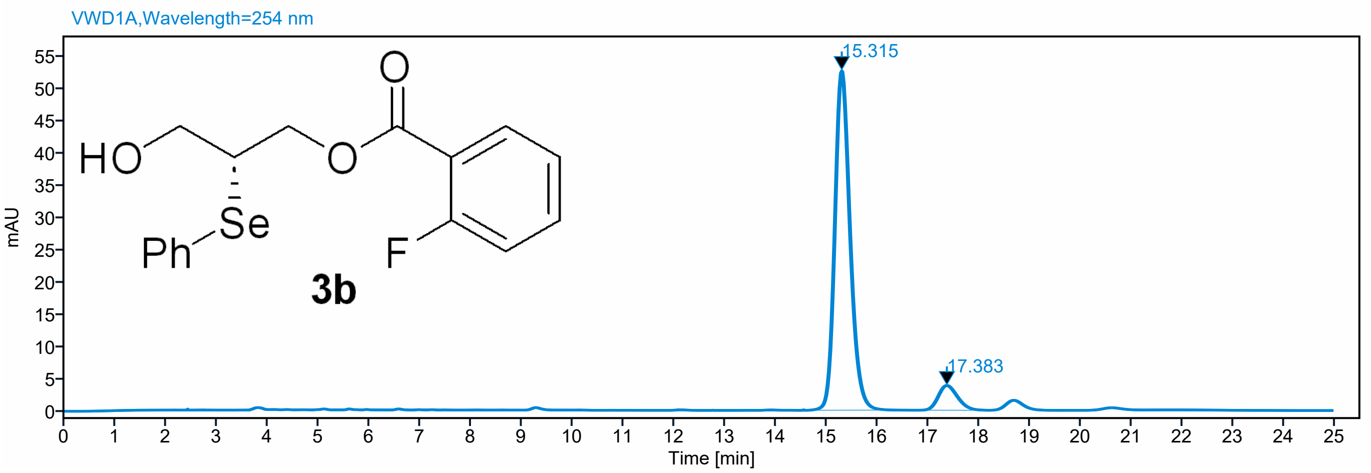

Signal: VWD1A,Wavelength=254 nm

RT [min]	Type	Width [min]	Area	Height	Area%
15.315	BB	1.68	1097.56	52.61	91.59
17.383	BB	1.32	100.82	3.83	8.41
		**Sum**	**1198.38**		

Single Injection Report

**Data file:**	CJX-3-131-ASY-IB-1.0-5%		
**Sequence Name:**	SingleSample	**Project Name:**	CJX
**Sample name:**	CJX-3-131-ASY-IB-1.0-5%	**Operator:**	SYSTEM (SYSTEM)
**Instrument:**	1260	**Injection date:**	2025-03-19 17:48:07+08:00
**Inj. volume:**	5.000 µL	**Location:**	P1-D1
**Acq. method:**	10%-30min-0.8ml-5uL.amx	**Type:**	Sample
**Processing method:**	GC_LC area percent_DefaultMethod.pmx	**Sample amount:**	0.00
**Manually modified:**	None		

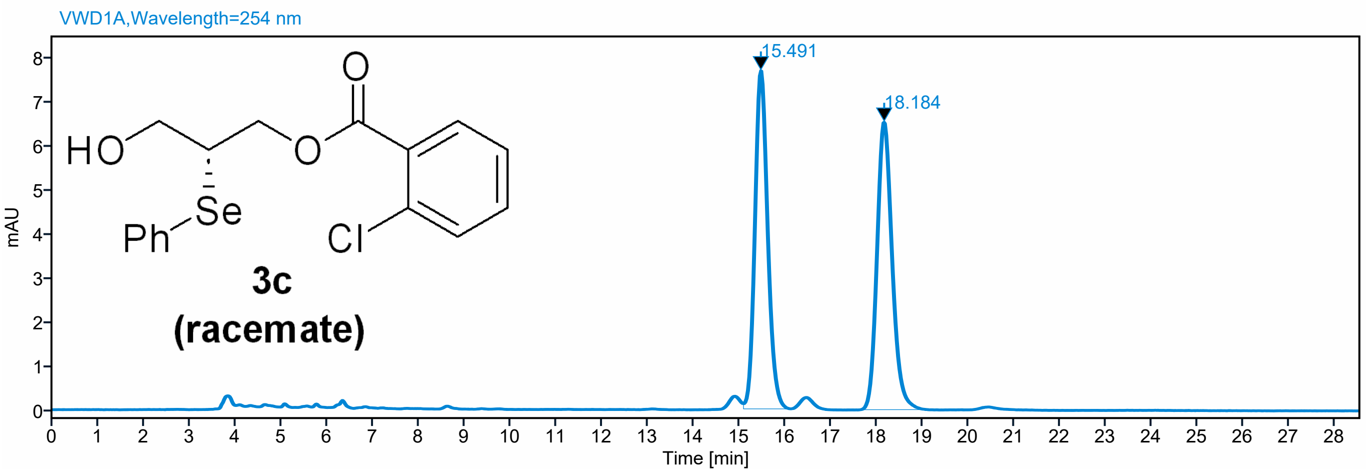

Signal: VWD1A,Wavelength=254 nm

RT [min]	Type	Width [min]	Area	Height	Area%
15.491	VB	1.03	148.43	7.67	49.74
18.184	BB	1.42	149.97	6.52	50.26
		**Sum**	**298.40**		

Single Injection Report

Data file: CJX-3-131-ASY-IB-1.0-5%

**Sequence Name:**	SingleSample	**Project Name:**	CJX
**Sample name:**	CJX-3-131-ASY-IB-1.0-5%	**Operator:**	SYSTEM (SYSTEM)
**Instrument:**	1260	**Injection date:**	2025-03-19 18:18:49+08:00
**Inj. volume:**	5.000 µL	**Location:**	P1-D2
**Acq. method:**	10%-30min-0.8ml-5uL.amx	**Type:**	Sample
**Processing method:**	GC_LC area percent_DefaultMethod.pmx	**Sample amount:**	0.00
**Manually modified:**	Manual Integration		

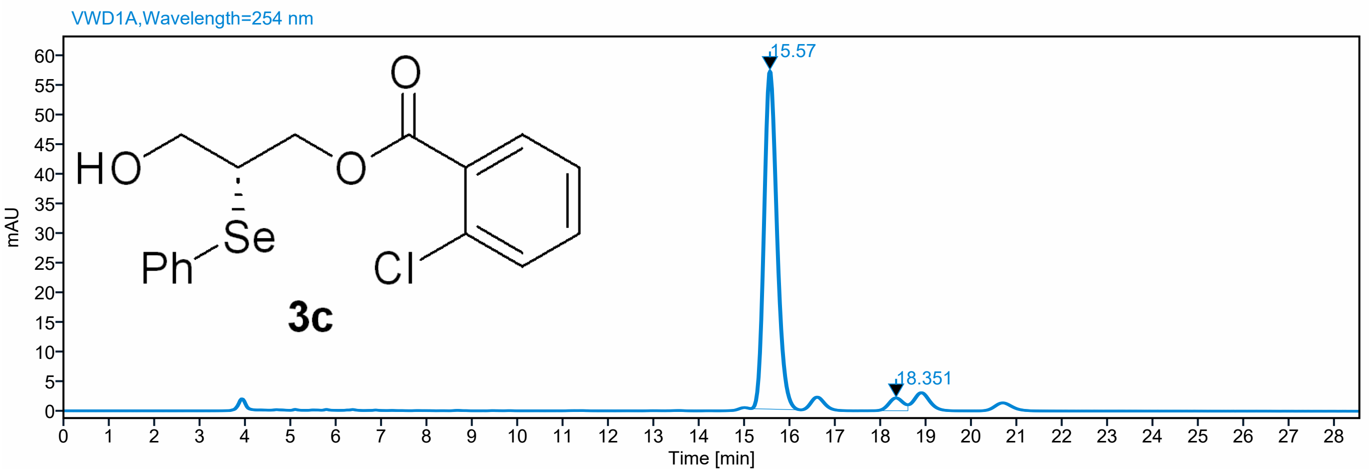

Signal: VWD1A,Wavelength=254 nm

RT [min]	Type	Width [min]	Area	Height	Area%
15.570	BB	1.11	1129.34	57.14	95.93
18.351	BV	0.72	47.96	2.16	4.07
		**Sum**	**1177.30**		

Single Injection Report

**Data file:**	CJX-2-20-RAC-IB-0.8-10%		
**Sequence Name:**	SingleSample	**Project Name:**	CJX
**Sample name:**	CJX-2-20-RAC-IB-0.8-10%	**Operator:**	SYSTEM (SYSTEM)
**Instrument:**	1260	**Injection date:**	2025-03-17 20:34:39+08:00
**Inj. volume:**	5.000 µL	**Location:**	P1-D3
**Acq. method:**	20%-40min-0.8ml-5uL.amx	**Type:**	Sample
**Processing method:**	GC_LC area percent_DefaultMethod.pmx	**Sample amount:**	0.00
**Manually modified:**	None		

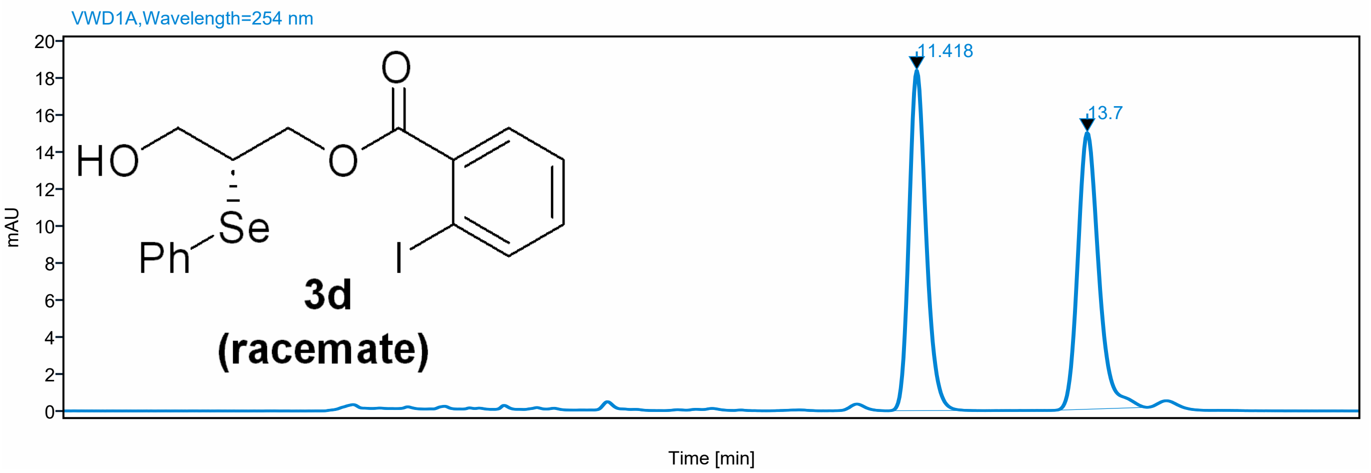

Signal: VWD1A,Wavelength=254 nm

RT [min]	Type	Width [min]	Area	Height	Area%
11.418	BB	1.06	291.71	18.38	50.04
13.700	BB	1.22	291.23	14.95	49.96
		**Sum**	**582.93**		

Single Injection Report

Agilent

Data file: CJX-2-20-ASY-IB-0.8-10%

**Sequence Name:**	SingleSample	**Project Name:**	CJX
**Sample name:**	CJX-2-20-ASY-IB-0.8-10%	**Operator:**	SYSTEM (SYSTEM)
**Instrument:**	1260	**Injection date:**	2025-05-06 10:32:28+08:00
**Inj. volume:**	5.000 µL	**Location:**	P2-D2
**Acq. method:**	10%-60min-0.8ml-5uL.amx	**Type:**	Sample
**Processing method:**	GC_LC area percent_DefaultMethod.pmx	**Sample amount:**	0.00
**Manually modified:**	Manual Integration		

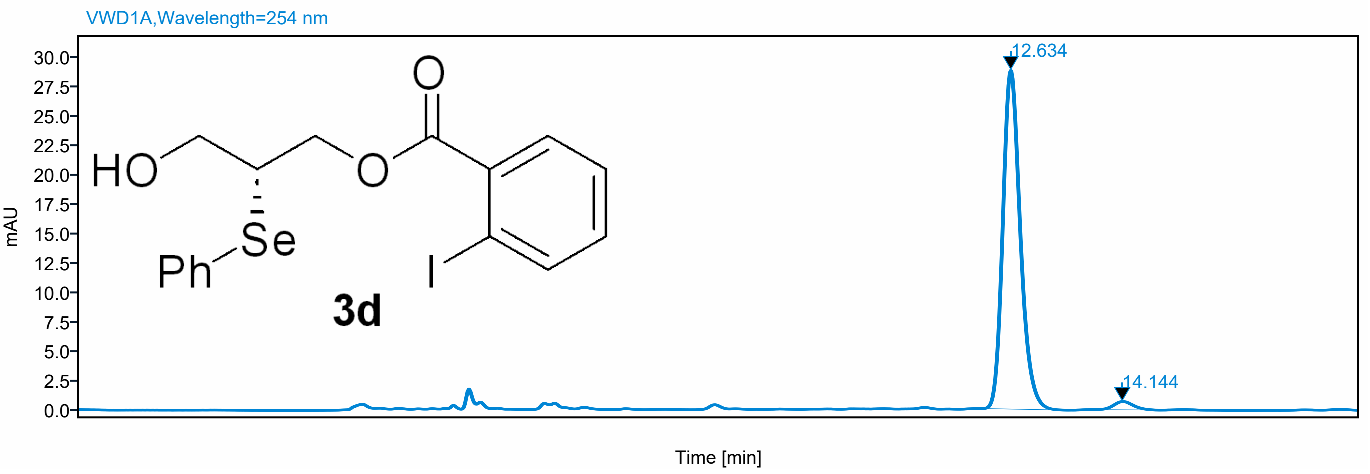

Signal: VWD1A,Wavelength=254 nm

RT [min]	Type	Width [min]	Area	Height	Area%
12.634	BB	1.15	474.72	28.79	97.31
14.144	MM m	1.00	13.10	0.71	2.69
		**Sum**	**487.83**		

Single Injection Report

Data file: CJX-2-32-RAC-OJ-0.8mL-10%

**Sequence Name:**	SingleSample	**Project Name:**	CJX
**Sample name:**	CJX-2-32-RAC-OJ-0.8mL-10%	**Operator:**	SYSTEM (SYSTEM)
**Instrument:**	1260	**Injection date:**	2025-03-26 17:13:44+08:00
**Inj. volume:**	5.000 µL	**Location:**	P1-C1
**Acq. method:**	10%-60min-0.8ml-5uL.amx	**Type:**	Sample
**Processing method:**	GC_LC area percent_DefaultMethod.pmx	**Sample amount:**	0.00
**Manually modified:**	Manual Integration		

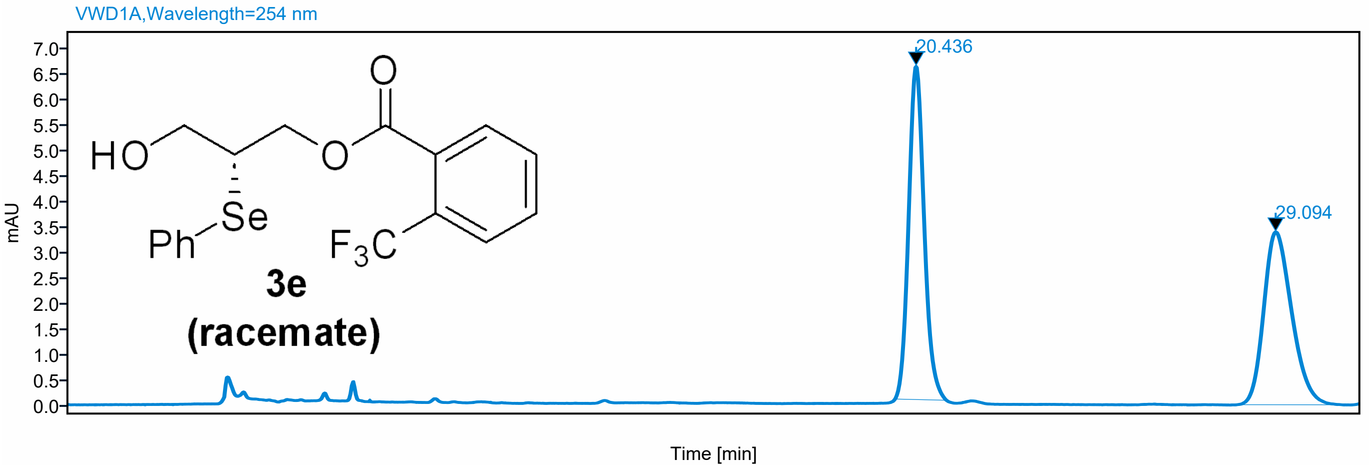

Signal: VWD1A,Wavelength=254 nm

RT [min]	Type	Width [min]	Area	Height	Area%
20.436	MM m	1.24	169.88	6.53	51.74
29.094	MM m	2.66	158.46	3.39	48.26
		**Sum**	**328.34**		

Single Injection Report

**Data file:**	CJX-2-32-ASY-OJ-0.8mL-10%		
**Sequence Name:**	SingleSample	**Project Name:**	CJX
**Sample name:**	CJX-2-32-ASY-OJ-0.8mL-10%	**Operator:**	SYSTEM (SYSTEM)
**Instrument:**	1260	**Injection date:**	2025-03-26 17:53:53+08:00
**Inj. volume:**	5.000 µL	**Location:**	P1-C2
**Acq. method:**	10%-60min-0.8ml-5uL.amx	**Type:**	Sample
**Processing method:**	GC_LC area percent_DefaultMethod.pmx	**Sample amount:**	0.00
**Manually modified:**	Manual Integration		

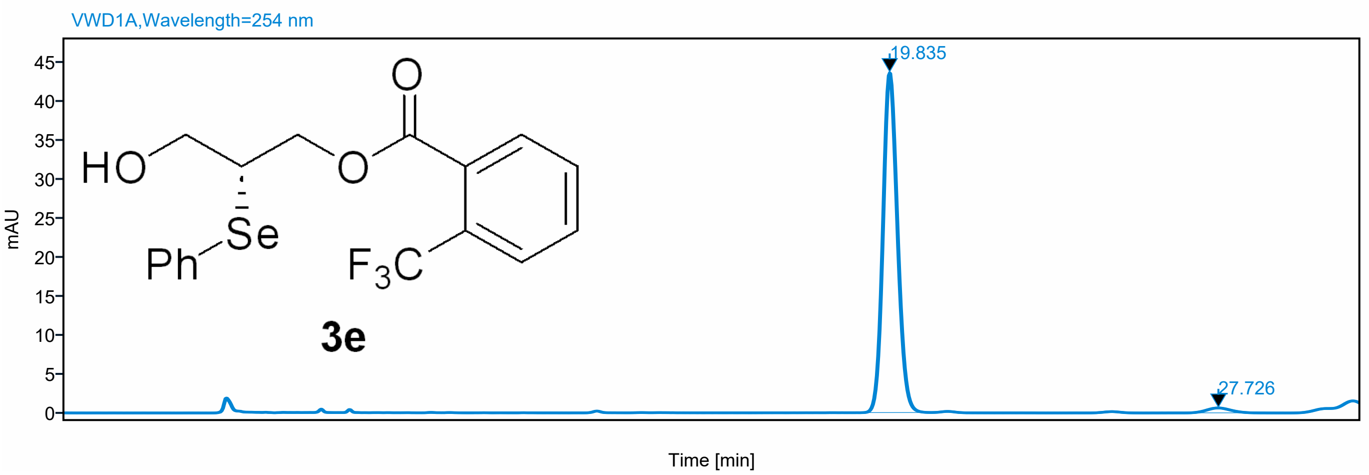

Signal: VWD1A,Wavelength=254 nm

RT [min]	Type	Width [min]	Area	Height	Area%
19.835	BB	1.60	1054.85	43.61	97.69
27.726	MM m	1.60	24.94	0.63	2.31
		**Sum**	**1079.79**		

Single Injection Report

**Data file:**	CJX-2-26-RAC-OJ-0.8mL-10%		
**Sequence Name:**	SingleSample	**Project Name:**	CJX
**Sample name:**	CJX-2-26-RAC-OJ-0.8mL-10%	**Operator:**	SYSTEM (SYSTEM)
**Instrument:**	1260	**Injection date:**	2025-03-22 08:51:25+08:00
**Inj. volume:**	5.000 µL	**Location:**	P1-B1
**Acq. method:**	10%-50min-0.8ml-5uL.amx	**Type:**	Sample
**Processing method:**	GC_LC area percent_DefaultMethod.pmx	**Sample amount:**	0.00
**Manually modified:**	Manual Integration		

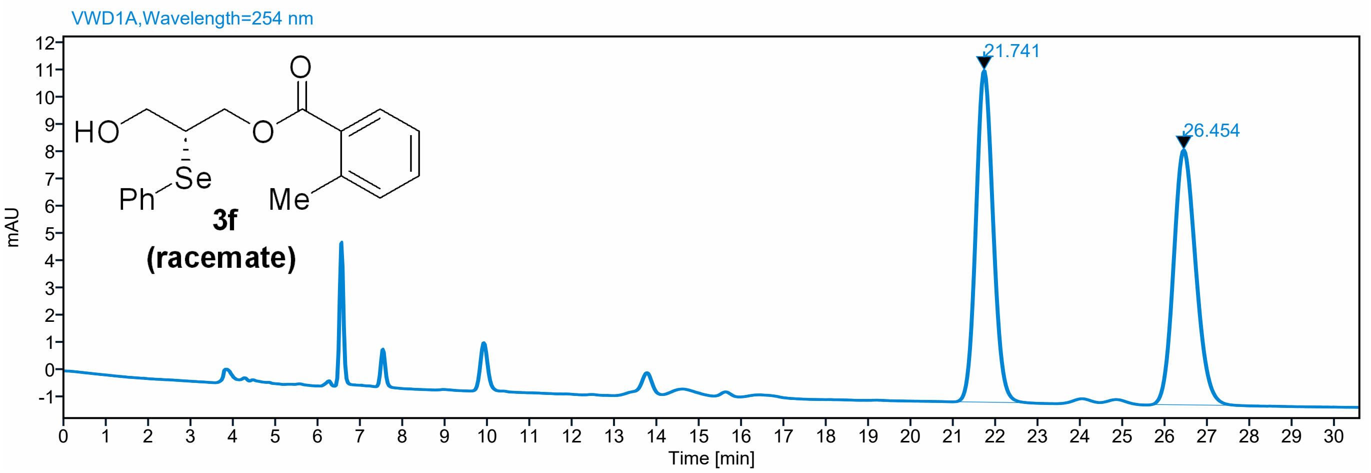

Signal: VWD1A,Wavelength=254 nm

RT [min]	Type	Width [min]	Area	Height	Area%
21.741	BB	1.69	343.61	12.17	50.18
26.454	BB	2.01	341.19	9.35	49.82
		**Sum**	**684.80**		

Single Injection Report

**Data file:**	CJX-2-26-ASY-OJ-0.8mL-10%		
**Sequence Name:**	SingleSample	**Project Name:**	CJX
**Sample name:**	CJX-2-26-ASY-OJ-0.8mL-10%	**Operator:**	SYSTEM (SYSTEM)
**Instrument:**	1260	**Injection date:**	2025-03-22 09:36:54+08:00
**Inj. volume:**	5.000 µL	**Location:**	P1-B2
**Acq. method:**	10%-50min-0.8ml-5uL.amx	**Type:**	Sample
**Processing method:**	GC_LC area percent_DefaultMethod.pmx	**Sample amount:**	0.00
**Manually modified:**	Manual Integration		

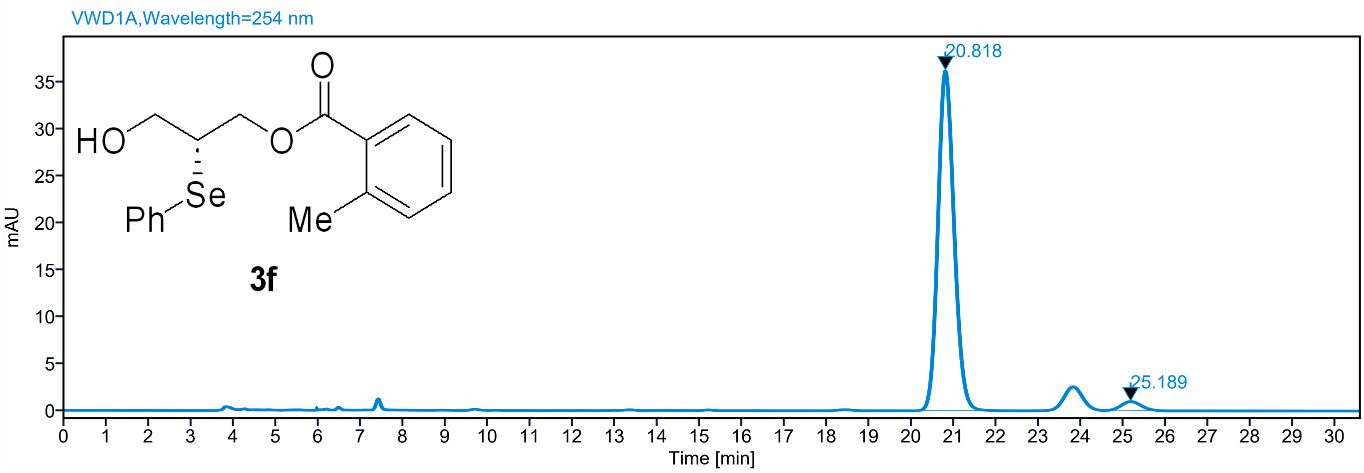

Signal: VWD1A,Wavelength=254 nm

RT [min]	Type	Width [min]	Area	Height	Area%
20.818	BB	1.74	959.03	36.20	96.95
25.189	MM m	2.01	30.13	0.95	3.05
		**Sum**	**989.16**		

Single Injection Report

Agilent

**Data file:**	CJX-2-13-RAC-IB-0.8mL-20%		
**Sequence Name:**	SingleSample	**Project Name:**	CJX
**Sample name:**	CJX-2-13-RAC-IB-0.8mL-20%	**Operator:**	SYSTEM (SYSTEM)
**Instrument:**	1260	**Injection date:**	2025-03-11 16:26:38+08:00
**Inj. volume:**	5.000 µL	**Location:**	P1-F3
**Acq. method:**	20%-30min-0.8ml-5uL.amx	**Type:**	Sample
**Processing method:**	GC_LC area percent_DefaultMethod.pmx	**Sample amount:**	0.00
**Manually modified:**	None		

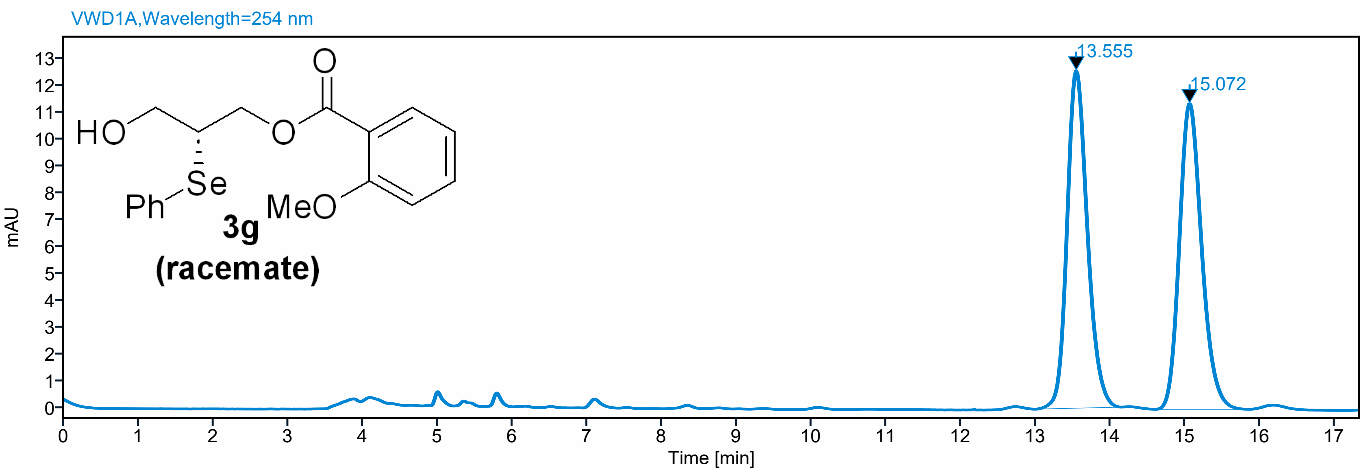

Signal: VWD1A,Wavelength=254 nm

RT [min]	Type	Width [min]	Area	Height	Area%
13.555	BB	1.14	237.26	12.56	50.09
15.072	BB	1.21	236.45	11.38	49.91
		**Sum**	**473.71**		

Single Injection Report

**Data file:**	CJX-2-13-ASY-IB-0.8mL-20%		
**Sequence Name:**	SingleSample	**Project Name:**	CJX
**Sample name:**	CJX-2-13-ASY-IB-0.8mL-20%	**Operator:**	SYSTEM (SYSTEM)
**Instrument:**	1260	**Injection date:**	2025-03-11 16:48:51+08:00
**Inj. volume:**	5.000 µL	**Location:**	P1-F4
**Acq. method:**	20%-30min-0.8ml-5uL.amx	**Type:**	Sample
**Processing method:**	GC_LC area percent_DefaultMethod.pmx	**Sample amount:**	0.00
**Manually modified:**	Manual Integration		

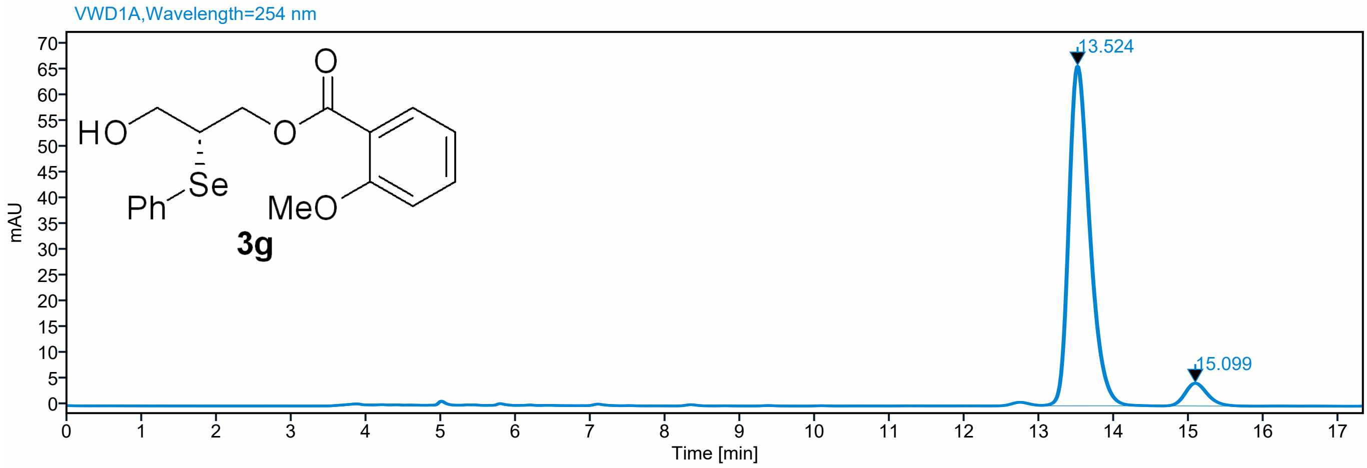

Signal: VWD1A,Wavelength=254 nm

RT [min]	Type	Width [min]	Area	Height	Area%
13.524	VB	1.51	1285.39	65.99	93.31
15.099	BB	1.14	92.18	4.42	6.69
		**Sum**	**1377.57**		

Single Injection Report

Agilent

**Data file:**	CJX-2-16-RAC-IB-0.8-10%		
**Sequence Name:**	SingleSample	**Project Name:**	CJX
**Sample name:**	CJX-2-16-RAC-IB-0.8-10%	**Operator:**	SYSTEM (SYSTEM)
**Instrument:**	1260	**Injection date:**	2025-03-15 20:42:23+08:00
**Inj. volume:**	5.000 µL	**Location:**	P1-D3
**Acq. method:**	10%-30min-0.8ml-5uL.amx	**Type:**	Sample
**Processing method:**	GC_LC area percent_DefaultMethod.pmx	**Sample amount:**	0.00
**Manually modified:**	None		

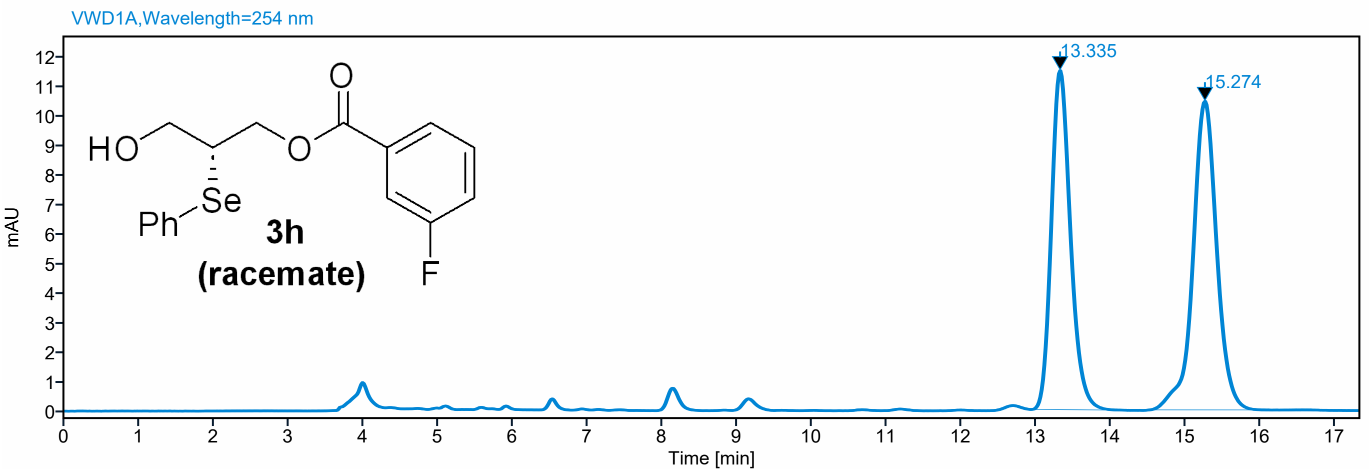

Signal: VWD1A,Wavelength=254 nm

RT [min]	Type	Width [min]	Area	Height	Area%
13.335	BB	1.17	203.99	11.47	46.88
15.274	BB	1.50	231.10	10.44	53.12
		**Sum**	**435.09**		

Single Injection Report

Agilent

**Data file:**	CJX-2-16-ASY-IB-0.8-10%		
**Sequence Name:**	SingleSample	**Project Name:**	CJX
**Sample name:**	CJX-2-16-ASY-IB-0.8-10%	**Operator:**	SYSTEM (SYSTEM)
**Instrument:**	1260	**Injection date:**	2025-05-06 11:28:59+08:00
**Inj. volume:**	5.000 µL	**Location:**	P2-D2
**Acq. method:**	10%-60min-0.8ml-5uL.amx	**Type:**	Sample
**Processing method:**	GC_LC area percent_DefaultMethod.pmx	**Sample amount:**	0.00
**Manually modified:**	Manual Integration		

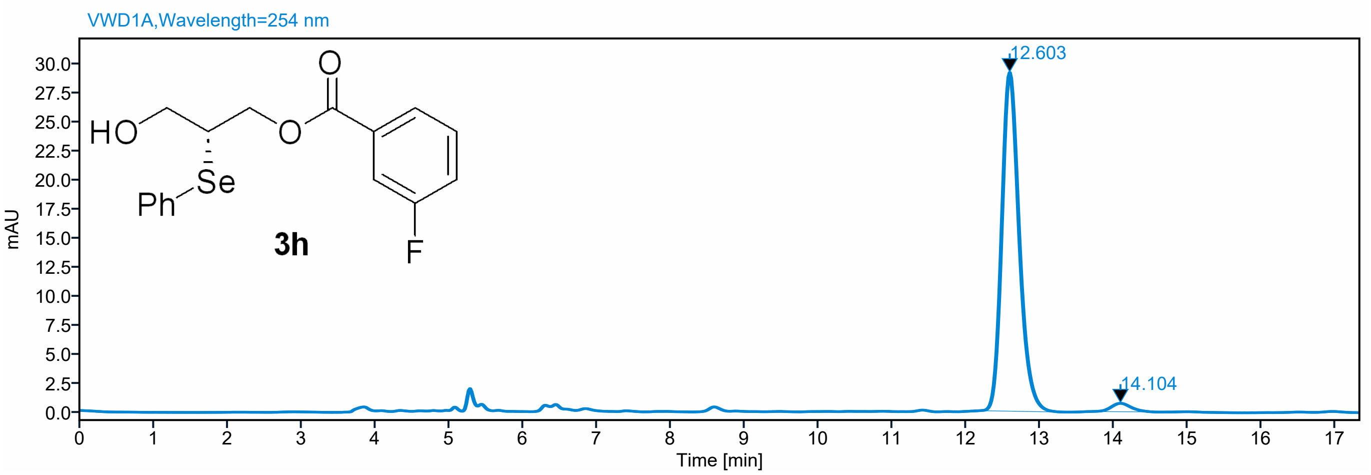

Signal: VWD1A,Wavelength=254 nm

RT [min]	Type	Width [min]	Area	Height	Area%
12.603	BB	1.17	478.36	29.15	97.22
14.104	MM m	1.06	13.68	0.73	2.78
		**Sum**	**492.03**		

Single Injection Report

**Data file:**	CJX-2-24-RAC-OJ-0.8mL-30%		
**Sequence Name:**	SingleSample	**Project Name:**	CJX
**Sample name:**	CJX-2-24-RAC-OJ-0.8mL-30%	**Operator:**	SYSTEM (SYSTEM)
**Instrument:**	1260	**Injection date:**	2025-03-21 00:17:11+08:00
**Inj. volume:**	5.000 µL	**Location:**	P1-F5
**Acq. method:**	30%-30min-0.8ml-5uL.amx	**Type:**	Sample
**Processing method:**	GC_LC area percent_DefaultMethod.pmx	**Sample amount:**	0.00
**Manually modified:**	Manual Integration		

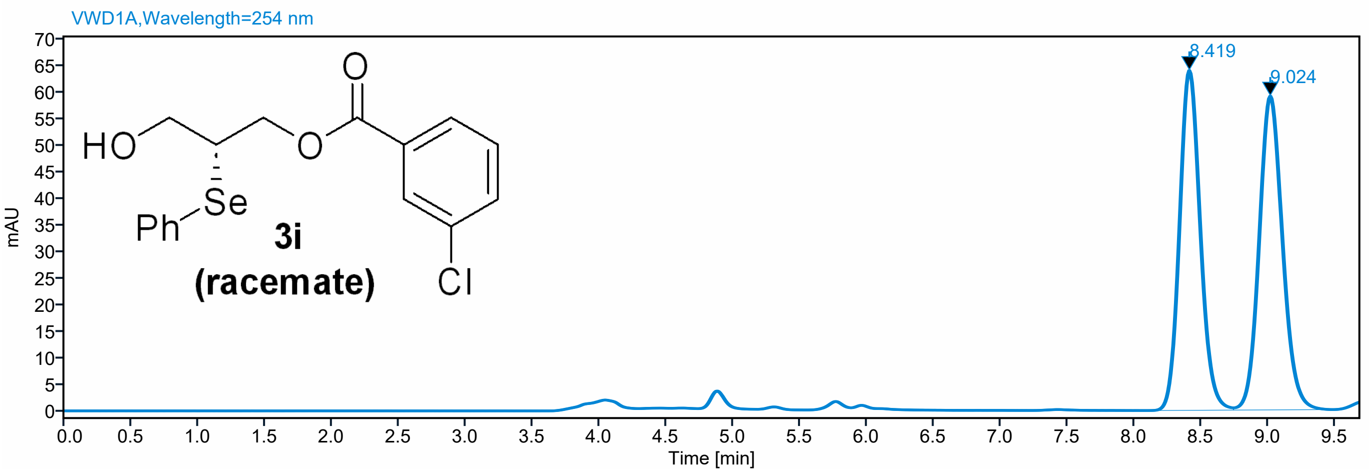

Signal: VWD1A,Wavelength=254 nm

RT [min]	Type	Width [min]	Area	Height	Area%
8.419	BV	0.65	690.00	63.91	49.05
9.024	VB	0.74	716.73	59.01	50.95
		**Sum**	**1406.73**		

Single Injection Report

**Data file:**	CJX-2-24-ASY-OJ-0.8mL-30%		
**Sequence Name:**	SingleSample	**Project Name:**	CJX
**Sample name:**	CJX-2-24-ASY-OJ-0.8mL-30%	**Operator:**	SYSTEM (SYSTEM)
**Instrument:**	1260	**Injection date:**	2025-03-21 00:47:54+08:00
**Inj. volume:**	5.000 µL	**Location:**	P1-F6
**Acq. method:**	30%-30min-0.8ml-5uL.amx	**Type:**	Sample
**Processing method:**	GC_LC area percent_DefaultMethod.pmx	**Sample amount:**	0.00
**Manually modified:**	Manual Integration		

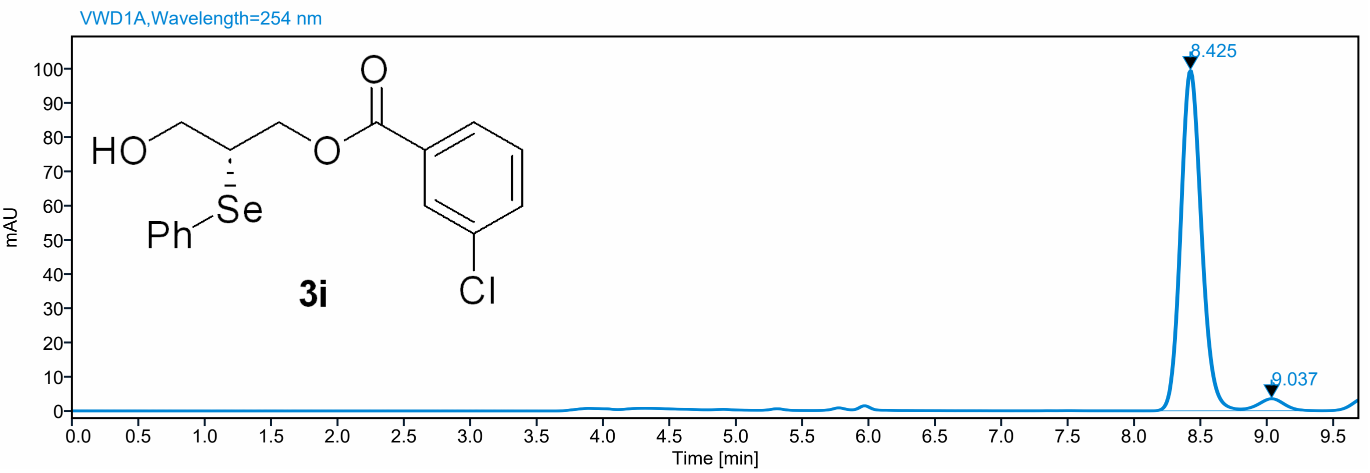

3i

Signal: VWD1A,Wavelength=254 nm

RT [min]	Type	Width [min]	Area	Height	Area%
8.425	BV	0.69	1081.46	99.42	95.69
9.037	VB	0.62	48.71	3.51	4.31
		**Sum**	**1130.16**		

**Data file:**	CJX-2-34-RAC-OJ-0.8mL-30%		
**Sequence Name:**	SingleSample	**Project Name:**	CJX
**Sample name:**	CJX-2-34-RAC-OJ-0.8mL-30%	**Operator:**	SYSTEM (SYSTEM)
**Instrument:**	1260	**Injection date:**	2025-03-26 20:42:49+08:00
**Inj. volume:**	5.000 µL	**Location:**	P1-B1
**Acq. method:**	30%-30min-0.8ml-5uL.amx	**Type:**	Sample
**Processing method:**	GC_LC area percent_DefaultMethod.pmx	**Sample amount:**	0.00
**Manually modified:**	None		

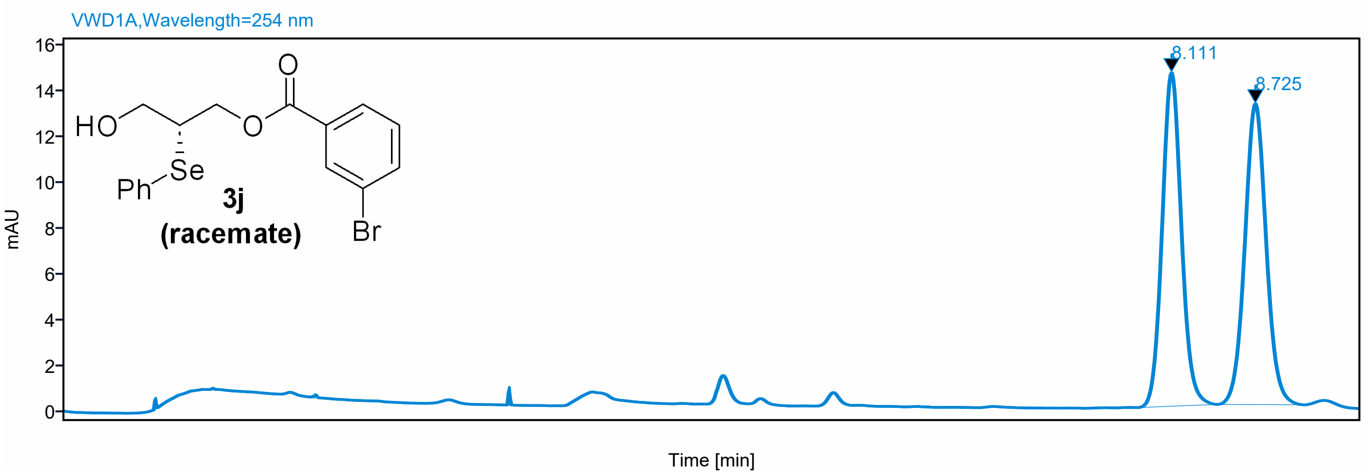

Signal: VWD1A,Wavelength=254 nm

RT [min]	Type	Width [min]	Area	Height	Area%
8.111	BB	0.58	142.62	14.56	49.96
8.725	BB	0.64	142.86	13.13	50.04
		**Sum**	**285.48**		

Single Injection Report

**Data file:**	CJX-2-34-ASY-OJ-0.8mL-30%		
**Sequence Name:**	SingleSample	**Project Name:**	CJX
**Sample name:**	CJX-2-34-ASY-OJ-0.8mL-30%	**Operator:**	SYSTEM (SYSTEM)
**Instrument:**	1260	**Injection date:**	2025-03-26 21:01:25+08:00
**Inj. volume:**	5.000 µL	**Location:**	P1-B2
**Acq. method:**	30%-30min-0.8ml-5uL.amx	**Type:**	Sample
**Processing method:**	GC_LC area percent_DefaultMethod.pmx	**Sample amount:**	0.00
**Manually modified:**	Manual Integration		

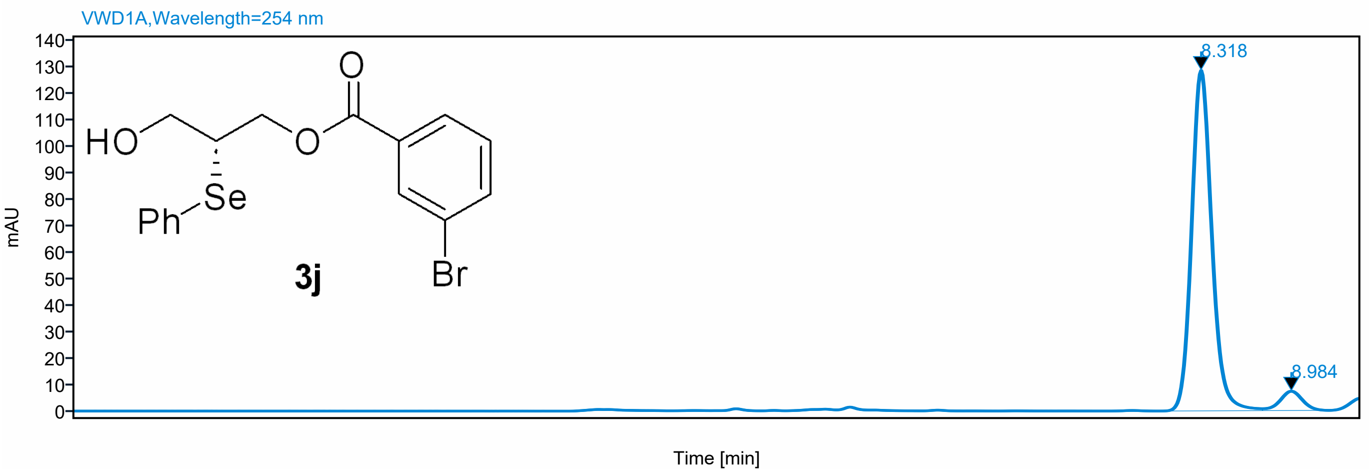

Signal: VWD1A,Wavelength=254 nm

RT [min]	Type	Width [min]	Area	Height	Area%
8.318	BV	0.76	1329.40	128.39	94.08
8.984	VB	0.48	83.60	7.28	5.92
		**Sum**	**1412.99**		

Single Injection Report

Agilent

**Data file:**	CJX-2-27-RAC-OJ-0.8mL-30%		
**Sequence Name:**	SingleSample	**Project Name:**	CJX
**Sample name:**	CJX-2-27-RAC-OJ-0.8mL-30%	**Operator:**	SYSTEM (SYSTEM)
**Instrument:**	1260	**Injection date:**	2025-03-20 22:14:21+08:00
**Inj. volume:**	5.000 µL	**Location:**	P1-F1
**Acq. method:**	30%-30min-0.8ml-5uL.amx	**Type:**	Sample
**Processing method:**	GC_LC area percent_DefaultMethod.pmx	**Sample amount:**	0.00
**Manually modified:**	None		

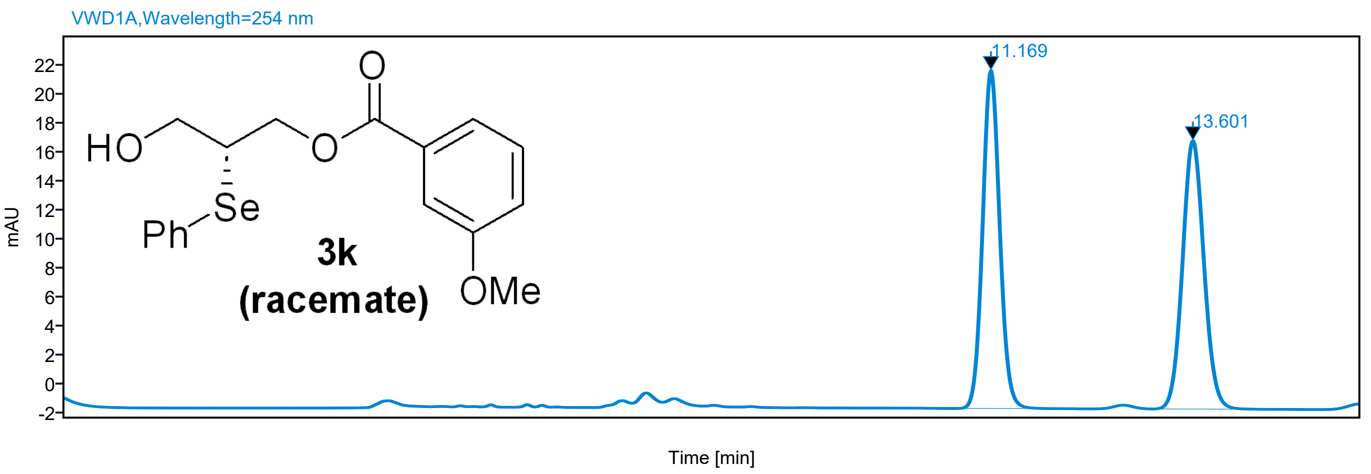

Signal: VWD1A,Wavelength=254 nm

RT [min]	Type	Width [min]	Area	Height	Area%
11.169	BB	1.00	333.49	23.33	50.01
13.601	BB	1.21	333.37	18.52	49.99
		**Sum**	**666.86**		

Single Injection Report

**Data file:**	CJX-2-27-ASY-OJ-0.8mL-30%		
**Sequence Name:**	SingleSample	**Project Name:**	CJX
**Sample name:**	CJX-2-27-ASY-OJ-0.8mL-30%	**Operator:**	SYSTEM (SYSTEM)
**Instrument:**	1260	**Injection date:**	2025-03-20 22:45:04+08:00
**Inj. volume:**	5.000 µL	**Location:**	P1-F2
**Acq. method:**	30%-30min-0.8ml-5uL.amx	**Type:**	Sample
**Processing method:**	GC_LC area percent_DefaultMethod.pmx	**Sample amount:**	0.00
**Manually modified:**	Manual Integration		

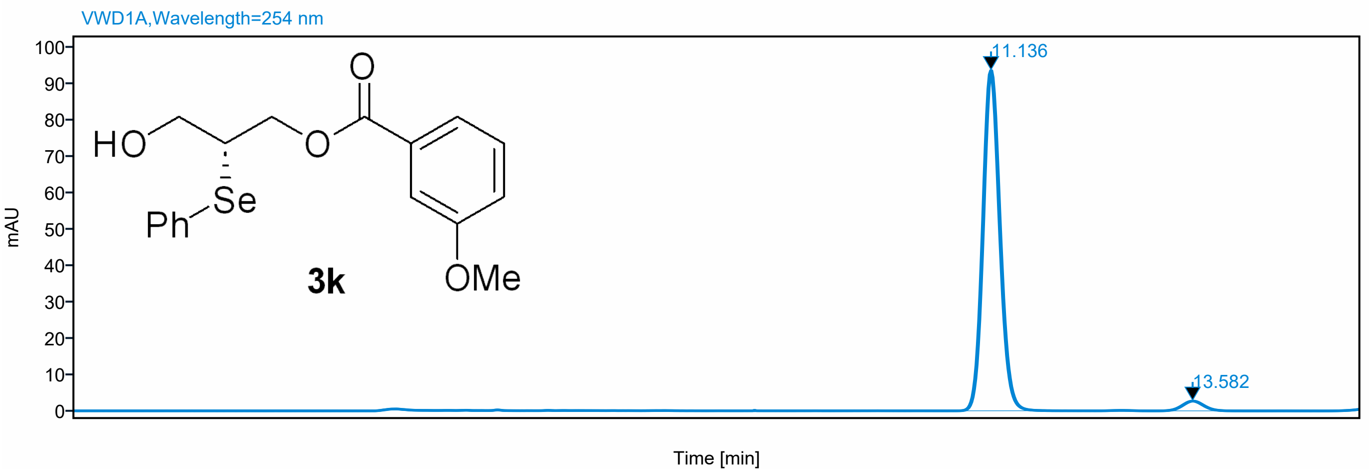

Signal: VWD1A,Wavelength=254 nm

RT [min]	Type	Width [min]	Area	Height	Area%
11.136	BB	1.24	1336.06	93.50	96.51
13.582	BB	0.93	48.35	2.69	3.49
		**Sum**	**1384.41**		

Single Injection Report

**Data file:**	CJX-2-15-RAC-IB-0.8-10%		
**Sequence Name:**	SingleSample	**Project Name:**	CJX
**Sample name:**	CJX-2-15-RAC-IB-0.8-10%	**Operator:**	SYSTEM (SYSTEM)
**Instrument:**	1260	**Injection date:**	2025-03-15 19:33:22+08:00
**Inj. volume:**	5.000 µL	**Location:**	P1-D1
**Acq. method:**	10%-30min-0.8ml-5uL.amx	**Type:**	Sample
**Processing method:**	GC_LC area percent_DefaultMethod.pmx	**Sample amount:**	0.00
**Manually modified:**	None		

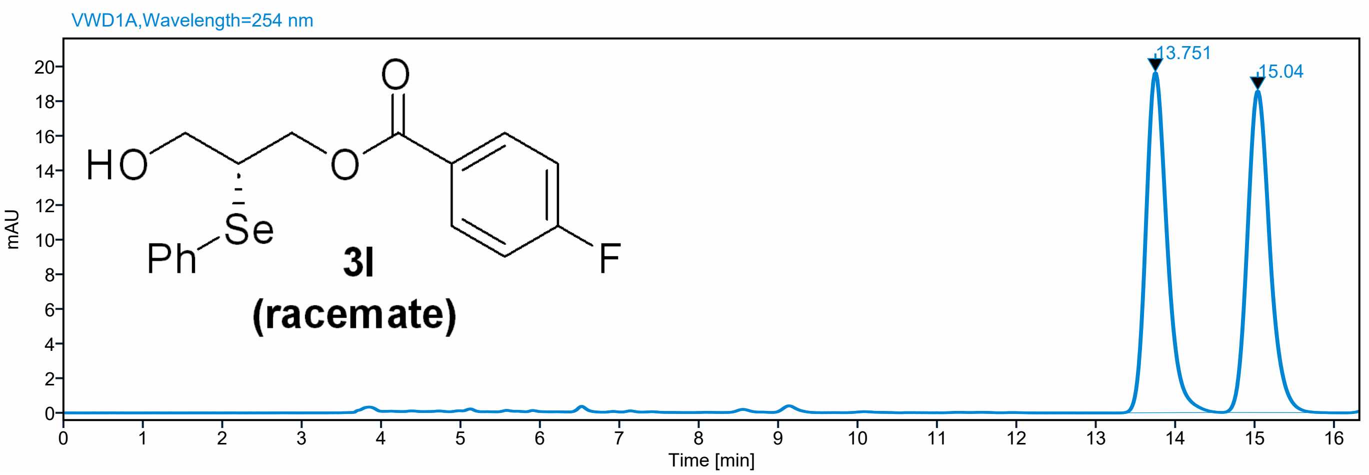

Signal: VWD1A,Wavelength=254 nm

RT [min]	Type	Width [min]	Area	Height	Area%
13.751	BB	1.26	363.39	19.65	49.98
15.040	BB	1.25	363.65	18.57	50.02
		**Sum**	**727.04**		

Single Injection Report

Agilent

**Data file:**	CJX-2-15-ASY-IB-0.8-10%		
**Sequence Name:**	SingleSample	**Project Name:**	CJX
**Sample name:**	CJX-2-15-ASY-IB-0.8-10%	**Operator:**	SYSTEM (SYSTEM)
**Instrument:**	1260	**Injection date:**	2025-05-06 10:10:48+08:00
**Inj. volume:**	5.000 µL	**Location:**	P2-D1
**Acq. method:**	10%-60min-0.8ml-5uL.amx	**Type:**	Sample
**Processing method:**	GC_LC area percent_DefaultMethod.pmx	**Sample amount:**	0.00
**Manually modified:**	Manual Integration		

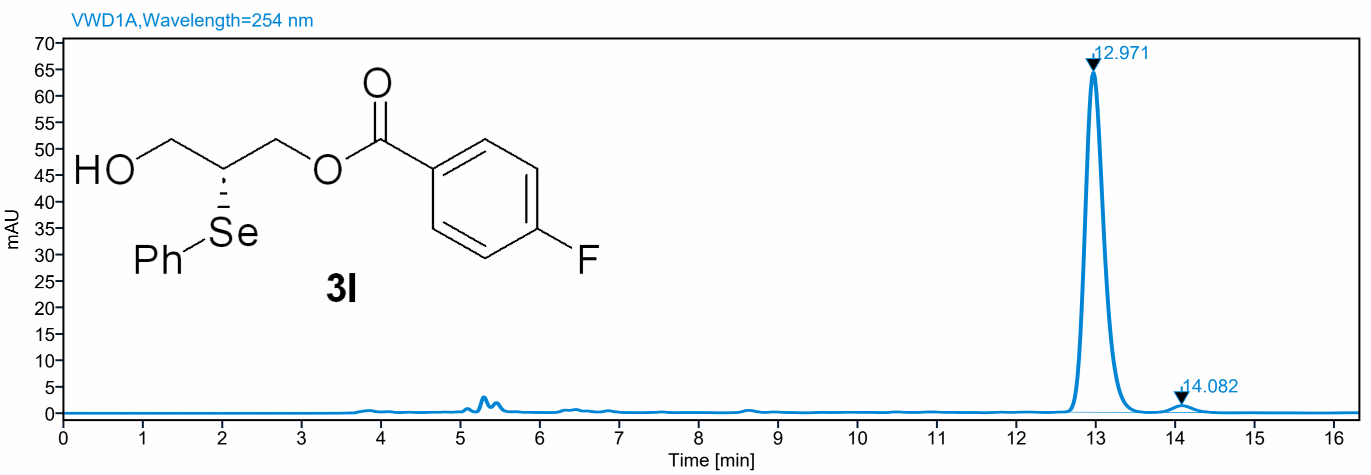

Signal: VWD1A,Wavelength=254 nm

RT [min]	Type	Width [min]	Area	Height	Area%
12.971	BM m	1.13	1069.91	64.22	97.77
14.082	MM m	1.09	24.37	1.31	2.23
		**Sum**	**1094.28**		

Single Injection Report

**Data file:**	CJX-2-35-RAC-IA-0.8-15%		
**Sequence Name:**	SingleSample	**Project Name:**	CJX
**Sample name:**	CJX-2-35-RAC-IA-0.8-15%	**Operator:**	SYSTEM (SYSTEM)
**Instrument:**	1260	**Injection date:**	2025-04-01 16:50:00+08:00
**Inj. volume:**	5.000 µL	**Location:**	P1-D1
**Acq. method:**	15%-20min-0.8ml-5uL.amx	**Type:**	Sample
**Processing method:**	GC_LC area percent_DefaultMethod.pmx	**Sample amount:**	0.00
**Manually modified:**	Manual Integration		

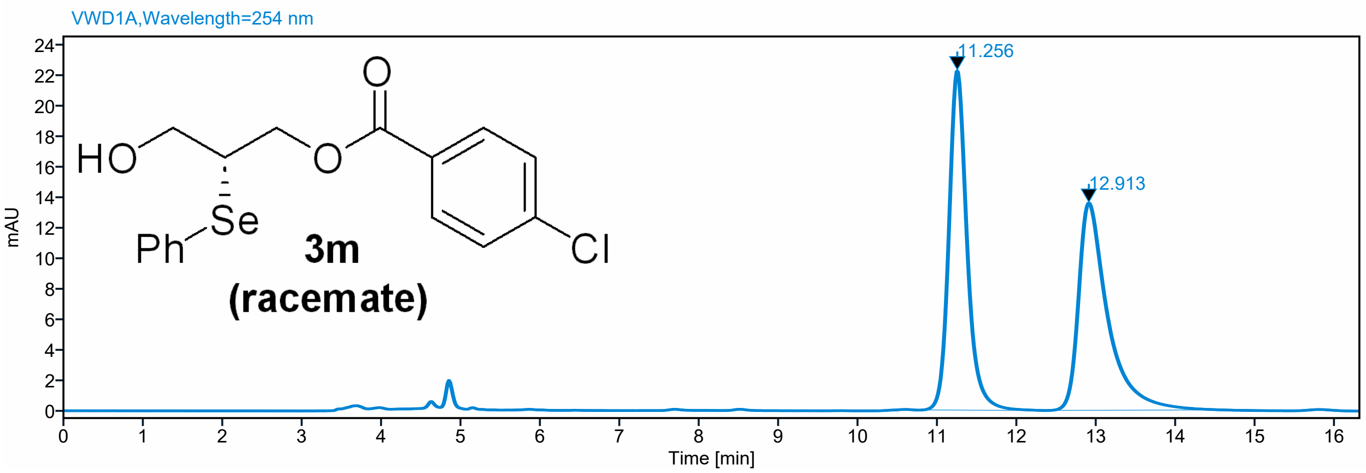

Signal: VWD1A,Wavelength=254 nm

RT [min]	Type	Width [min]	Area	Height	Area%
11.256	BB	1.53	354.22	22.26	50.43
12.913	BB	2.16	348.18	13.59	49.57
		**Sum**	**702.40**		

Single Injection Report

**Data file:**	CJX-2-35-ASY-IA-0.8-15%		
**Sequence Name:**	SingleSample	**Project Name:**	CJX
**Sample name:**	CJX-2-35-ASY-IA-0.8-15%	**Operator:**	SYSTEM (SYSTEM)
**Instrument:**	1260	**Injection date:**	2025-04-01 10:43:41+08:00
**Inj. volume:**	5.000 µL	**Location:**	P1-D1
**Acq. method:**	15%-50min-0.8ml-5uL.amx	**Type:**	Sample
**Processing method:**	GC_LC area percent_DefaultMethod.pmx	**Sample amount:**	0.00
**Manually modified:**	None		

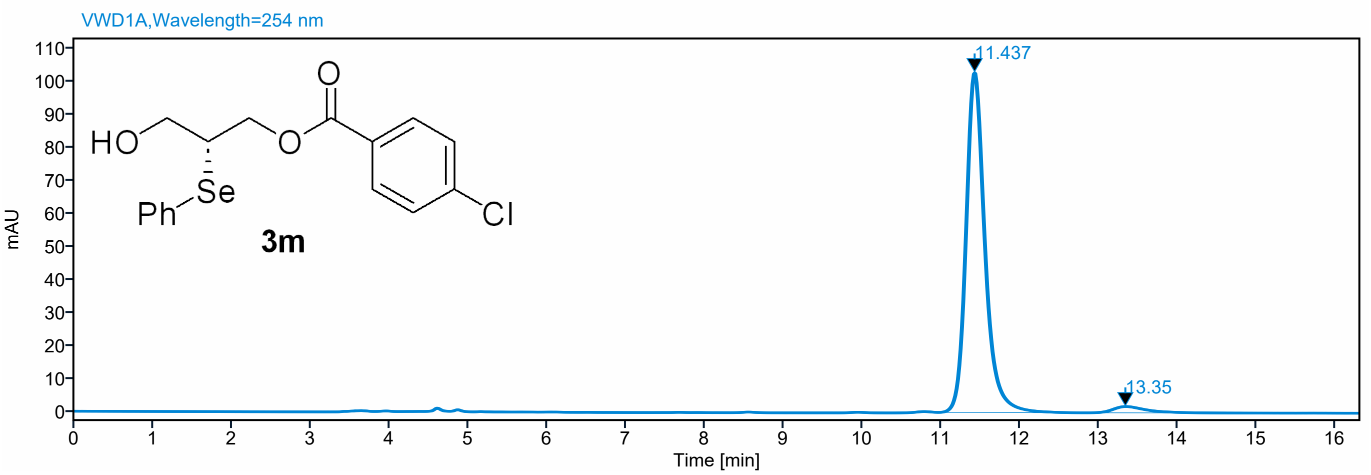

Signal: VWD1A,Wavelength=254 nm

RT [min]	Type	Width [min]	Area	Height	Area%
11.437	BB	1.75	1719.01	102.91	96.88
13.350	BB	1.60	55.43	1.90	3.12
		**Sum**	**1774.44**		

Single Injection Report

**Data file:**	CJX-2-45-RAC-IA-1.0-10%		
**Sequence Name:**	SingleSample	**Project Name:**	CJX
**Sample name:**	CJX-2-45-RAC-IA-1.0-10%	**Operator:**	SYSTEM (SYSTEM)
**Instrument:**	1260	**Injection date:**	2025-04-03 17:30:32+08:00
**Inj. volume:**	5.000 µL	**Location:**	P1-D1
**Acq. method:**	10%-60min-1.0ml-5uL.amx	**Type:**	Sample
**Processing method:**	GC_LC area percent_DefaultMethod.pmx	**Sample amount:**	0.00
**Manually modified:**	None		

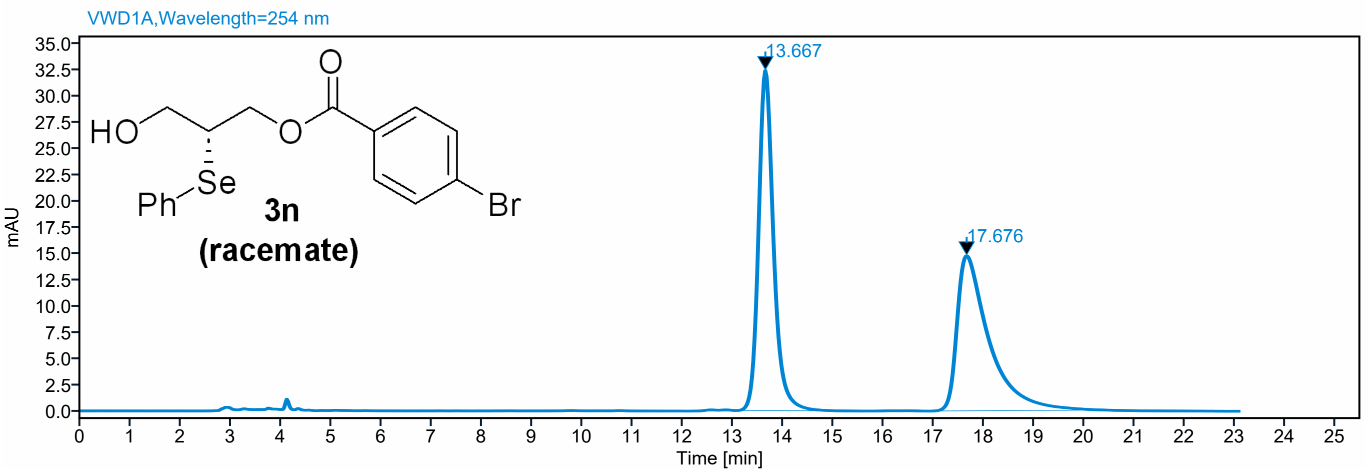

Signal: VWD1A,Wavelength=254 nm

RT [min]	Type	Width [min]	Area	Height	Area%
13.667	BB	2.01	676.25	32.36	50.77
17.676	BB	3.38	655.80	14.75	49.23
		**Sum**	**1332.05**		

Single Injection Report

**Data file:**	CJX-2-45-ASY-IA-1.0-10%		
**Sequence Name:**	SingleSample	**Project Name:**	CJX
**Sample name:**	CJX-2-45-ASY-IA-1.0-10%	**Operator:**	SYSTEM (SYSTEM)
**Instrument:**	1260	**Injection date:**	2025-04-03 17:54:32+08:00
**Inj. volume:**	5.000 µL	**Location:**	P1-D2
**Acq. method:**	10%-60min-1.0ml-5uL.amx	**Type:**	Sample
**Processing method:**	GC_LC area percent_DefaultMethod.pmx	**Sample amount:**	0.00
**Manually modified:**	None		

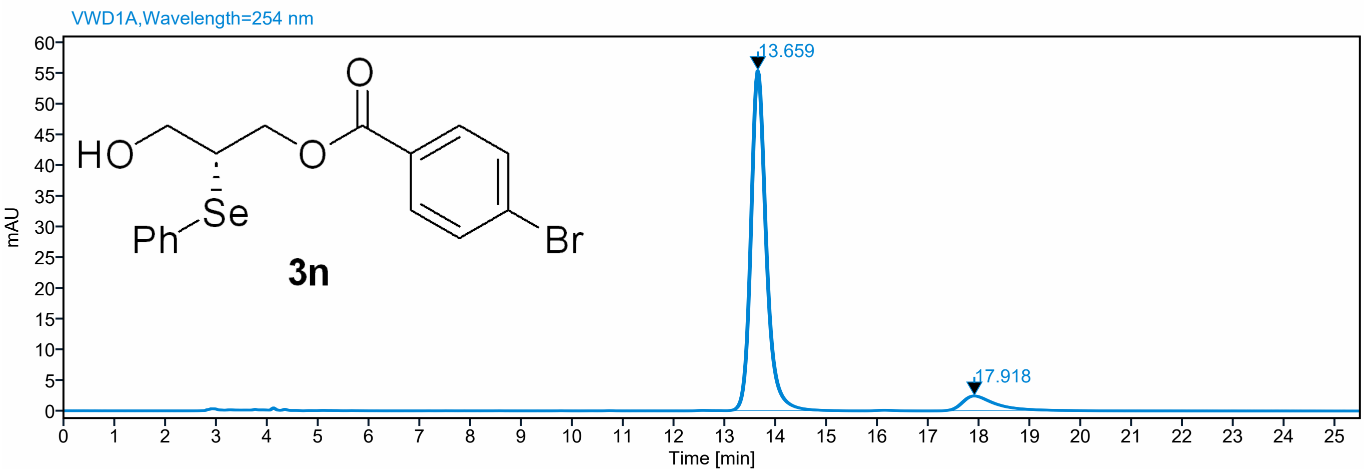

Signal: VWD1A,Wavelength=254 nm

RT [min]	Type	Width [min]	Area	Height	Area%
13.659	BB	2.07	1158.61	55.38	91.72
17.918	BB	2.14	104.53	2.38	8.28
		**Sum**	**1263.14**		

Single Injection Report

Agilent

**Data file:**	CJX-2-47-RAC-IB-1.0-10%		
**Sequence Name:**	SingleSample	**Project Name:**	CJX
**Sample name:**	CJX-2-47-RAC-IB-1.0-10%	**Operator:**	SYSTEM (SYSTEM)
**Instrument:**	1260	**Injection date:**	2025-04-04 14:30:21+08:00
**Inj. volume:**	5.000 µL	**Location:**	P1-D1
**Acq. method:**	10%-60min-1.0ml-5uL.amx	**Type:**	Sample
**Processing method:**	GC_LC area percent_DefaultMethod.pmx	**Sample amount:**	0.00
**Manually modified:**	None		

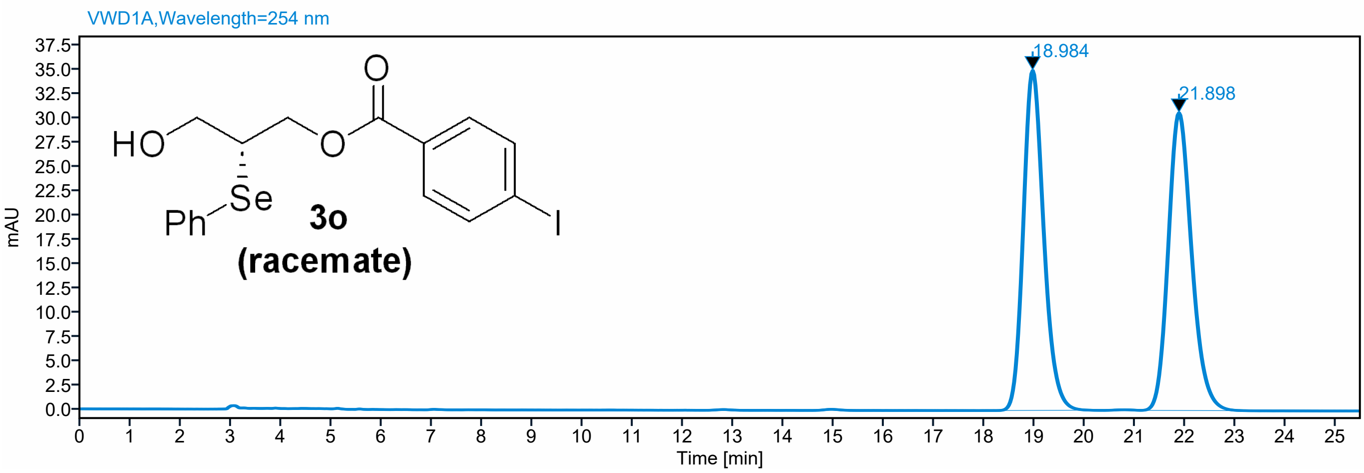

Signal: VWD1A,Wavelength=254 nm

RT [min]	Type	Width [min]	Area	Height	Area%
18.984	BB	1.81	988.86	34.98	49.83
21.898	BB	2.11	995.58	30.63	50.17
		**Sum**	**1984.44**		

Single Injection Report

**Data file:**	CJX-2-47-ASY-IB-1.0-10%		
**Sequence Name:**	SingleSample	**Project Name:**	CJX
**Sample name:**	CJX-2-47-ASY-IB-1.0-10%	**Operator:**	SYSTEM (SYSTEM)
**Instrument:**	1260	**Injection date:**	2025-04-04 15:01:03+08:00
**Inj. volume:**	5.000 µL	**Location:**	P1-D2
**Acq. method:**	10%-60min-1.0ml-5uL.amx	**Type:**	Sample
**Processing method:**	GC_LC area percent_DefaultMethod.pmx	**Sample amount:**	0.00
**Manually modified:**	None		

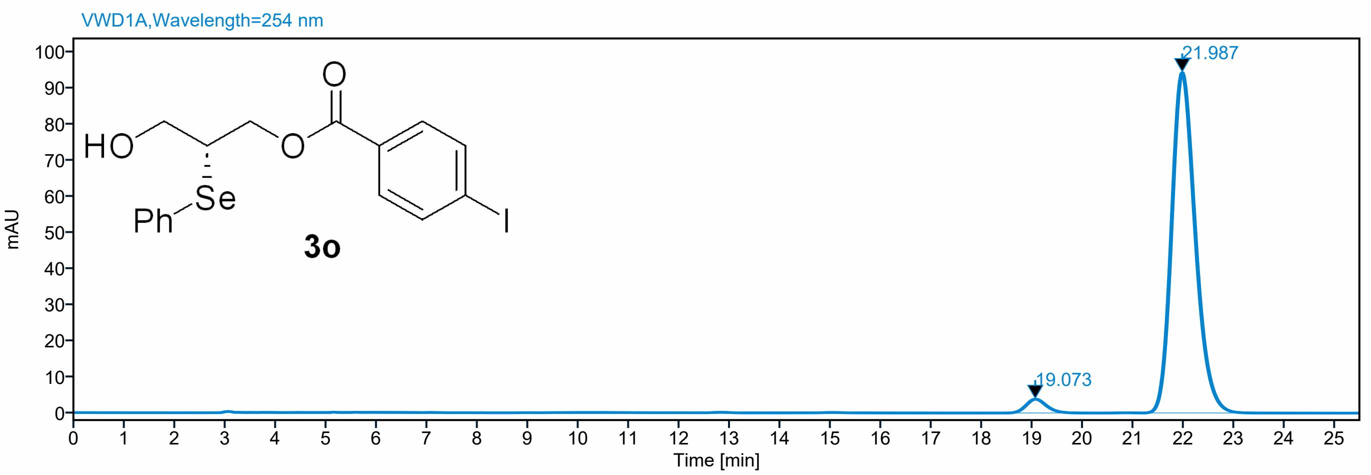

Signal: VWD1A,Wavelength=254 nm

RT [min]	Type	Width [min]	Area	Height	Area%
19.073	BB	1.47	107.88	3.82	3.38
21.987	BB	2.24	3079.18	94.29	96.62
		**Sum**	**3187.06**		

Single Injection Report

**Data file:**	CJX-3-48-3-RAC-IB-1.0mL-10%		
**Sequence Name:**	SingleSample	**Project Name:**	CJX
**Sample name:**	CJX-3-48-3-RAC-IB-1.0mL-10%	**Operator:**	SYSTEM (SYSTEM)
**Instrument:**	1260	**Injection date:**	2025-07-22 22:11:48+08:00
**Inj. volume:**	5.000 µL	**Location:**	P1-A5
**Acq. method:**	10%-60min-1.0ml-5uL.amx	**Type:**	Sample
**Processing method:**	GC_LC area percent_DefaultMethod.pmx	**Sample amount:**	0.00
**Manually modified:**	Manual Integration		

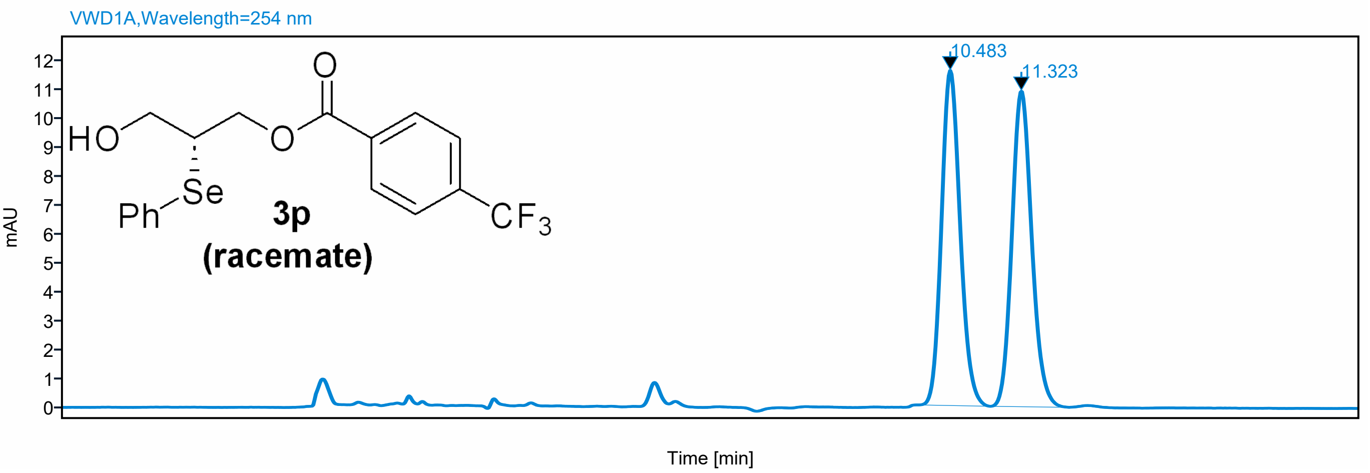

Signal: VWD1A,Wavelength=254 nm

RT [min]	Type	Width [min]	Area	Height	Area%
10.483	MM m	0.85	177.32	11.58	49.95
11.323	BB	0.92	177.65	10.91	50.05
		**Sum**	**354.97**		

Single Injection Report

**Data file:**	CJX-3-48-3-ASY-IB-1.0mL-10%		
**Sequence Name:**	SingleSample	**Project Name:**	CJX
**Sample name:**	CJX-3-48-3-ASY-IB-1.0mL-10%	**Operator:**	SYSTEM (SYSTEM)
**Instrument:**	1260	**Injection date:**	2025-07-22 21:53:09+08:00
**Inj. volume:**	5.000 µL	**Location:**	P1-A6
**Acq. method:**	10%-60min-1.0ml-5uL.amx	**Type:**	Sample
**Processing method:**	GC_LC area percent_DefaultMethod.pmx	**Sample amount:**	0.00
**Manually modified:**	Manual Integration		

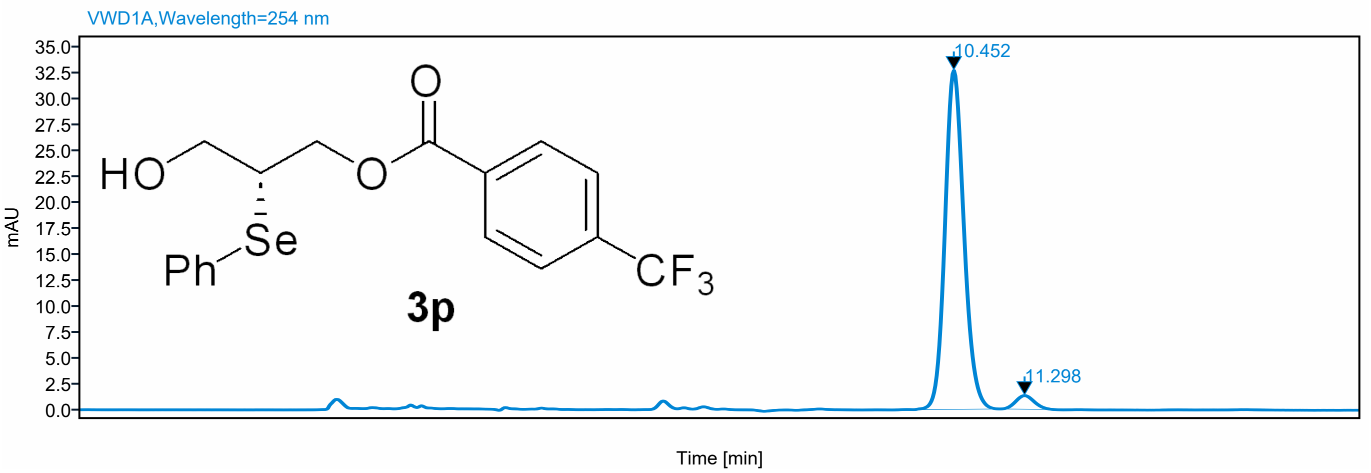

Signal: VWD1A,Wavelength=254 nm

RT [min]	Type	Width [min]	Area	Height	Area%
10.452	BB	1.02	514.81	32.66	96.25
11.298	MM m	0.90	20.06	1.31	3.75
		**Sum**	**534.87**		

Single Injection Report

**Data file:**	CJX-2-37-RAC-IB-1.0-20%		
**Sequence Name:**	SingleSample	**Project Name:**	CJX
**Sample name:**	CJX-2-37-RAC-IB-1.0-20%	**Operator:**	SYSTEM (SYSTEM)
**Instrument:**	1260	**Injection date:**	2025-04-01 20:26:04+08:00
**Inj. volume:**	5.000 µL	**Location:**	P1-D1
**Acq. method:**	20%-50min-1.0ml-5uL.amx	**Type:**	Sample
**Processing method:**	GC_LC area percent_DefaultMethod.pmx	**Sample amount:**	0.00
**Manually modified:**	None		

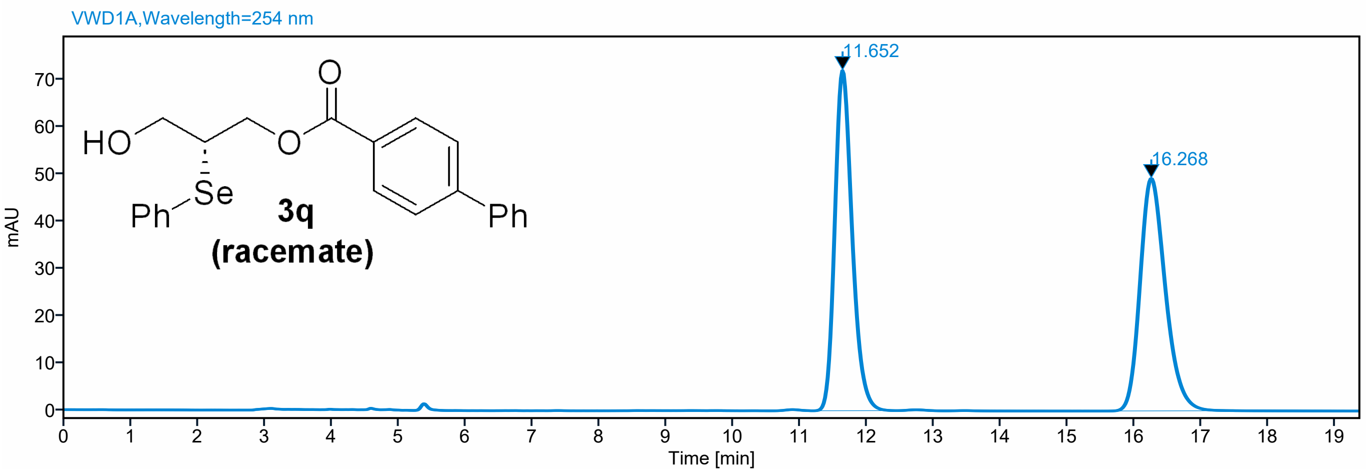

Signal: VWD1A,Wavelength=254 nm

RT [min]	Type	Width [min]	Area	Height	Area%
11.652	BB	1.25	1302.93	71.98	50.11
16.268	BB	1.86	1297.04	49.17	49.89
		**Sum**	**2599.97**		

Single Injection Report

**Data file:**	CJX-2-37-ASY-IB-1.0-20%		
**Sequence Name:**	SingleSample	**Project Name:**	CJX
**Sample name:**	CJX-2-37-ASY-IB-1.0-20%	**Operator:**	SYSTEM (SYSTEM)
**Instrument:**	1260	**Injection date:**	2025-04-01 20:50:34+08:00
**Inj. volume:**	5.000 µL	**Location:**	P1-D2
**Acq. method:**	20%-50min-1.0ml-5uL.amx	**Type:**	Sample
**Processing method:**	GC_LC area percent_DefaultMethod.pmx	**Sample amount:**	0.00
**Manually modified:**	None		

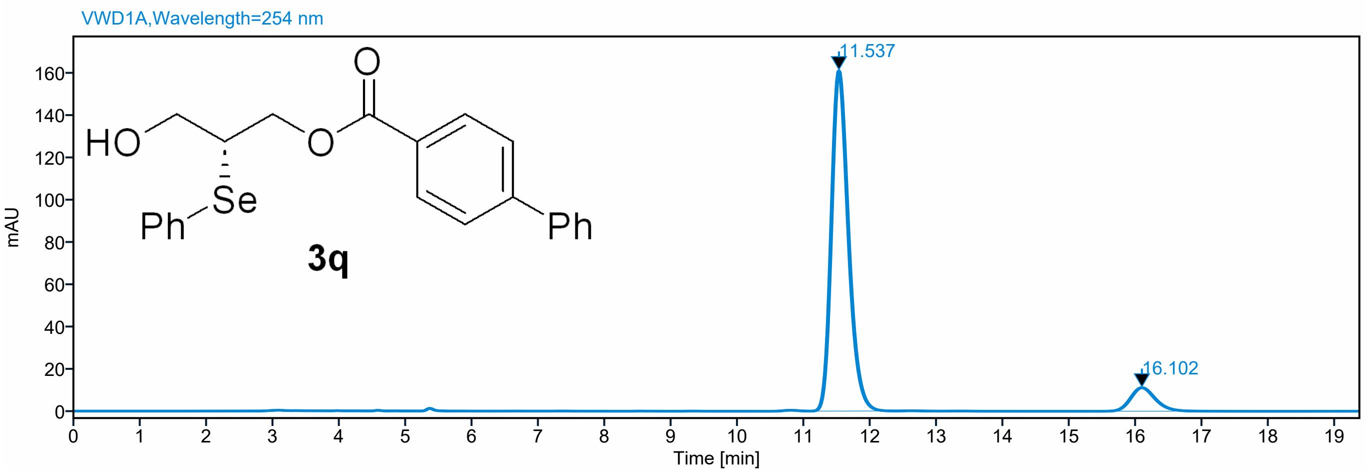

Signal: VWD1A,Wavelength=254 nm

RT [min]	Type	Width [min]	Area	Height	Area%
11.537	BB	1.26	2870.28	161.06	90.89
16.102	BB	1.49	287.61	11.12	9.11
		**Sum**	**3157.88**		

Single Injection Report

Agilent

Data file: CJX-2-12-RAC-IA-0.8mL-10%

**Sequence Name:**	SingleSample	**Project Name:**	CJX
**Sample name:**	CJX-2-12-RAC-IA-0.8mL-10%	**Operator:**	SYSTEM (SYSTEM)
**Instrument:**	1260	**Injection date:**	2025-03-11 10:58:19+08:00
**Inj. volume:**	5.000 µL	**Location:**	P1-F1
**Acq. method:**	10%-50min-0.8ml-5uL.amx	**Type:**	Sample
**Processing method:**	GC_LC area percent_DefaultMethod.pmx	**Sample amount:**	0.00
**Manually modified:**	None		

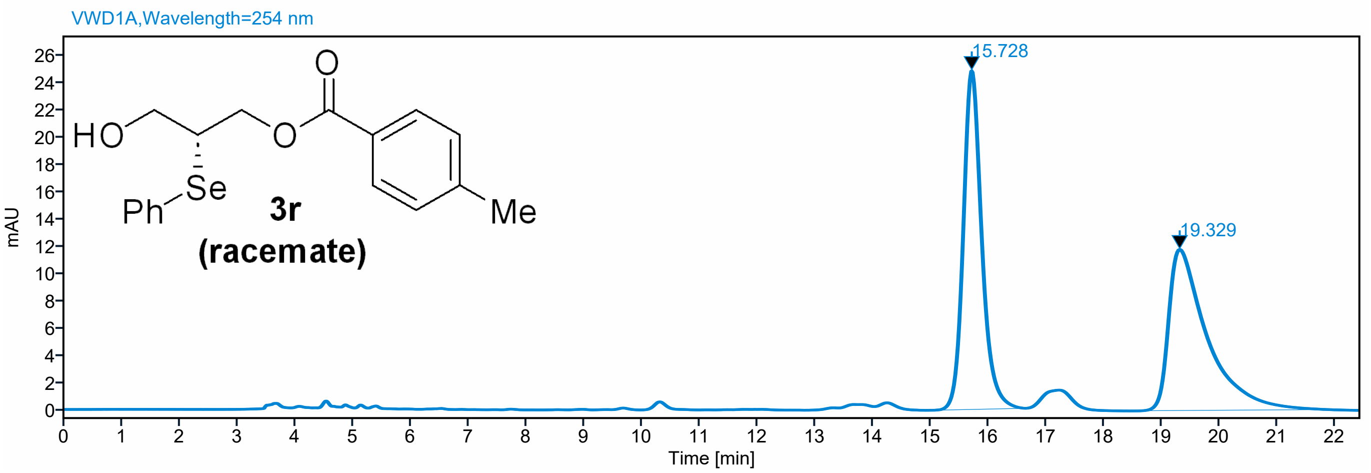

Signal: VWD1A,Wavelength=254 nm

RT [min]	Type	Width [min]	Area	Height	Area%
15.728	BB	1.59	552.21	24.83	50.24
19.329	BB	3.19	546.86	11.80	49.76
		**Sum**	**1099.06**		

Single Injection Report

**Data file:**	CJX-2-12-ASY-IA-0.8mL-10%		
**Sequence Name:**	SingleSample	**Project Name:**	CJX
**Sample name:**	CJX-2-12-ASY-IA-0.8mL-10%	**Operator:**	SYSTEM (SYSTEM)
**Instrument:**	1260	**Injection date:**	2025-03-11 11:23:21+08:00
**Inj. volume:**	5.000 µL	**Location:**	P1-F2
**Acq. method:**	10%-50min-0.8ml-5uL.amx	**Type:**	Sample
**Processing method:**	GC_LC area percent_DefaultMethod.pmx	**Sample amount:**	0.00
**Manually modified:**	Manual Integration		

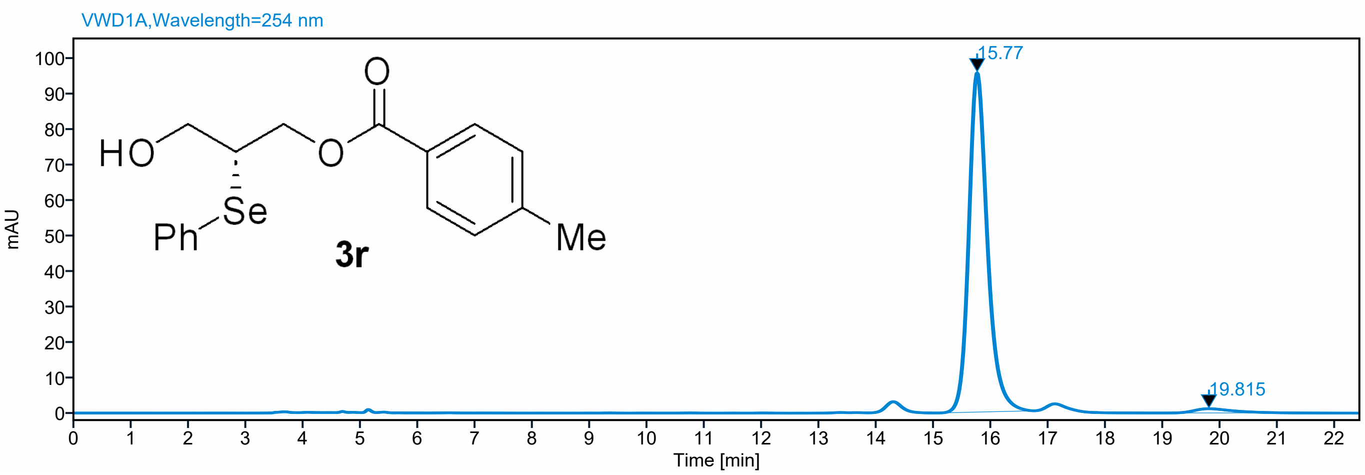

Signal: VWD1A,Wavelength=254 nm

RT [min]	Type	Width [min]	Area	Height	Area%
15.770	BB	1.67	2160.49	95.68	97.46
19.815	MM m	2.10	56.21	1.20	2.54
		**Sum**	**2216.70**		

Single Injection Report

Agilent

**Data file:**	CJX-2-36-RAC-IA-0.8-15%		
**Sequence Name:**	SingleSample	**Project Name:**	CJX
**Sample name:**	CJX-2-36-RAC-IA-0.8-15%	**Operator:**	SYSTEM (SYSTEM)
**Instrument:**	1260	**Injection date:**	2025-04-01 11:01:54+08:00
**Inj. volume:**	5.000 µL	**Location:**	P1-D2
**Acq. method:**	15%-50min-0.8ml-5uL.amx	**Type:**	Sample
**Processing method:**	GC_LC area percent_DefaultMethod.pmx	**Sample amount:**	0.00
**Manually modified:**	None		

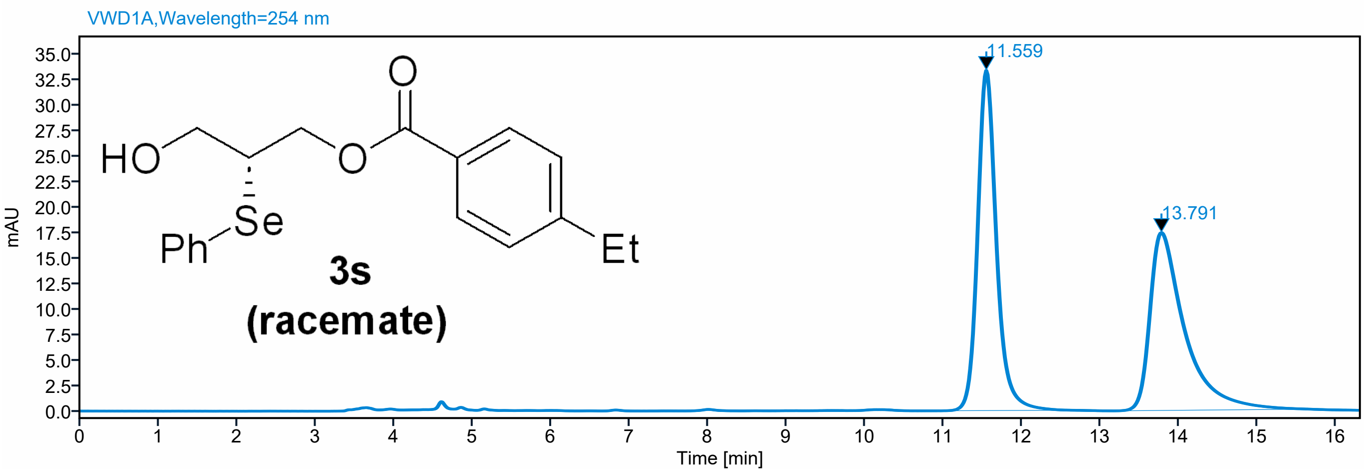

Signal: VWD1A,Wavelength=254 nm

RT [min]	Type	Width [min]	Area	Height	Area%
11.559	BB	1.60	558.17	33.31	50.96
13.791	BB	2.43	537.03	17.42	49.04
		**Sum**	**1095.20**		

Single Injection Report

Agilent

**Data file:**	CJX-2-36-ASY-IA-0.8-15%		
**Sequence Name:**	SingleSample	**Project Name:**	CJX
**Sample name:**	CJX-2-36-ASY-IA-0.8-15%	**Operator:**	SYSTEM (SYSTEM)
**Instrument:**	1260	**Injection date:**	2025-04-01 11:41:18+08:00
**Inj. volume:**	5.000 µL	**Location:**	P1-D3
**Acq. method:**	15%-50min-0.8ml-5uL.amx	**Type:**	Sample
**Processing method:**	GC_LC area percent_DefaultMethod.pmx	**Sample amount:**	0.00
**Manually modified:**	None		

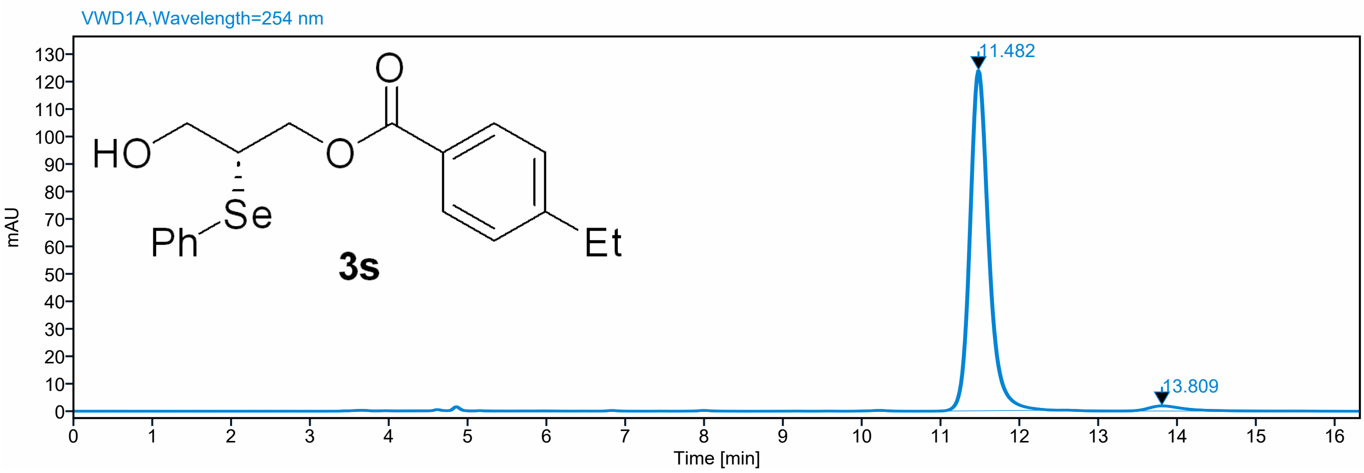

Signal: VWD1A,Wavelength=254 nm

RT [min]	Type	Width [min]	Area	Height	Area%
11.482	BB	1.55	2050.63	123.88	97.18
13.809	BB	1.79	59.52	1.88	2.82
		**Sum**	**2110.15**		

Single Injection Report

Agilent

**Data file:**	CJX-2-33-RAC-IA-0.8mL-15%		
**Sequence Name:**	SingleSample	**Project Name:**	CJX
**Sample name:**	CJX-2-33-RAC-IA-0.8mL-15%	**Operator:**	SYSTEM (SYSTEM)
**Instrument:**	1260	**Injection date:**	2025-04-01 15:52:06+08:00
**Inj. volume:**	5.000 µL	**Location:**	P1-D5
**Acq. method:**	15%-20min-0.8ml-5uL.amx	**Type:**	Sample
**Processing method:**	GC_LC area percent_DefaultMethod.pmx	**Sample amount:**	0.00
**Manually modified:**	Manual Integration		

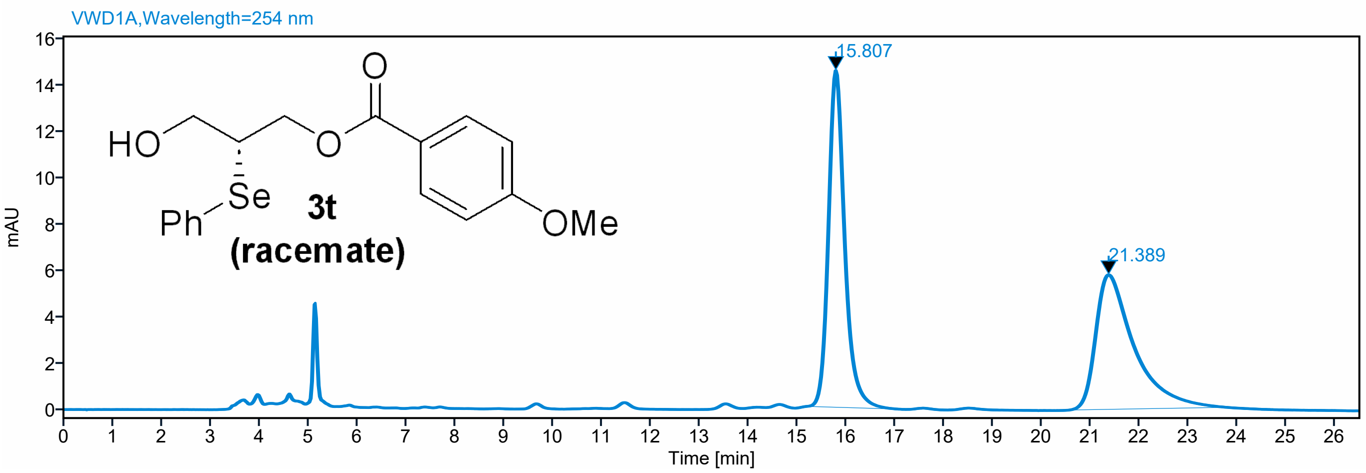

Signal: VWD1A,Wavelength=254 nm

RT [min]	Type	Width [min]	Area	Height	Area%
15.807	BB	1.85	336.52	14.52	51.48
21.389	BB	3.21	317.23	5.80	48.52
		**Sum**	**653.75**		

Single Injection Report

Agilent

**Data file:**	CJX-2-33-ASY-IA-0.8mL-15%		
**Sequence Name:**	SingleSample	**Project Name:**	CJX
**Sample name:**	CJX-2-33-ASY-IA-0.8mL-15%	**Operator:**	SYSTEM (SYSTEM)
**Instrument:**	1260	**Injection date:**	2025-04-01 16:21:11+08:00
**Inj. volume:**	5.000 µL	**Location:**	P1-D6
**Acq. method:**	15%-20min-0.8ml-5uL.amx	**Type:**	Sample
**Processing method:**	GC_LC area percent_DefaultMethod.pmx	**Sample amount:**	0.00
**Manually modified:**	Manual Integration		

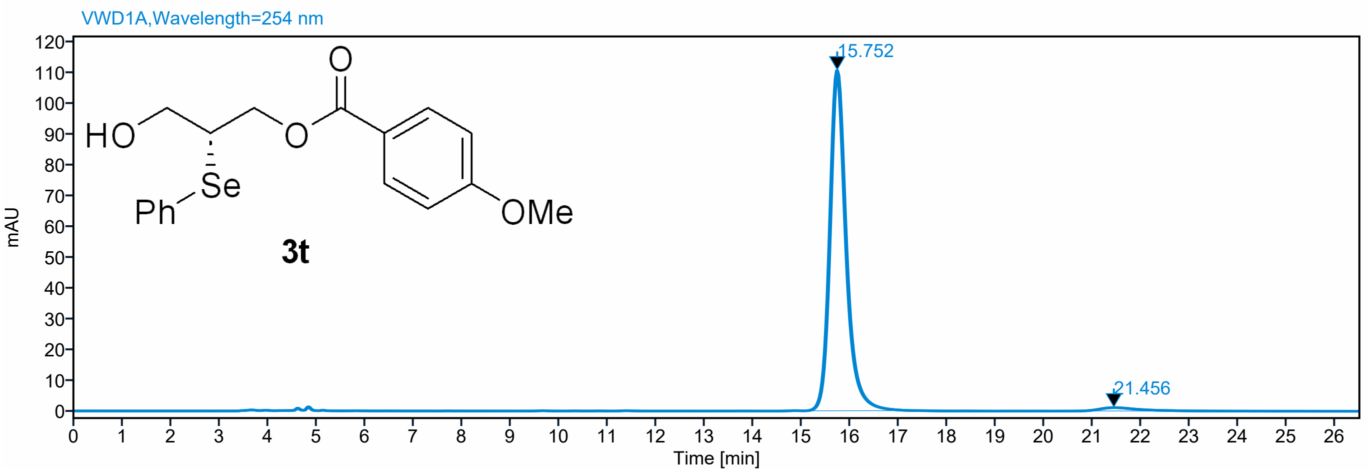

Signal: VWD1A,Wavelength=254 nm

RT [min]	Type	Width [min]	Area	Height	Area%
15.752	BB	2.30	2584.58	110.51	97.75
21.456	MM m	2.57	59.49	1.08	2.25
		**Sum**	**2644.07**		

Single Injection Report

Agilent

**Data file:**	CJX-2-43-RAC-IB-0.8-10%		
**Sequence Name:**	SingleSample	**Project Name:**	CJX
**Sample name:**	CJX-2-43-RAC-IB-0.8-10%	**Operator:**	SYSTEM (SYSTEM)
**Instrument:**	1260	**Injection date:**	2025-04-03 09:44:32+08:00
**Inj. volume:**	5.000 µL	**Location:**	P1-D1
**Acq. method:**	10%-50min-0.8ml-5uL.amx	**Type:**	Sample
**Processing method:**	GC_LC area percent_DefaultMethod.pmx	**Sample amount:**	0.00
**Manually modified:**	None		

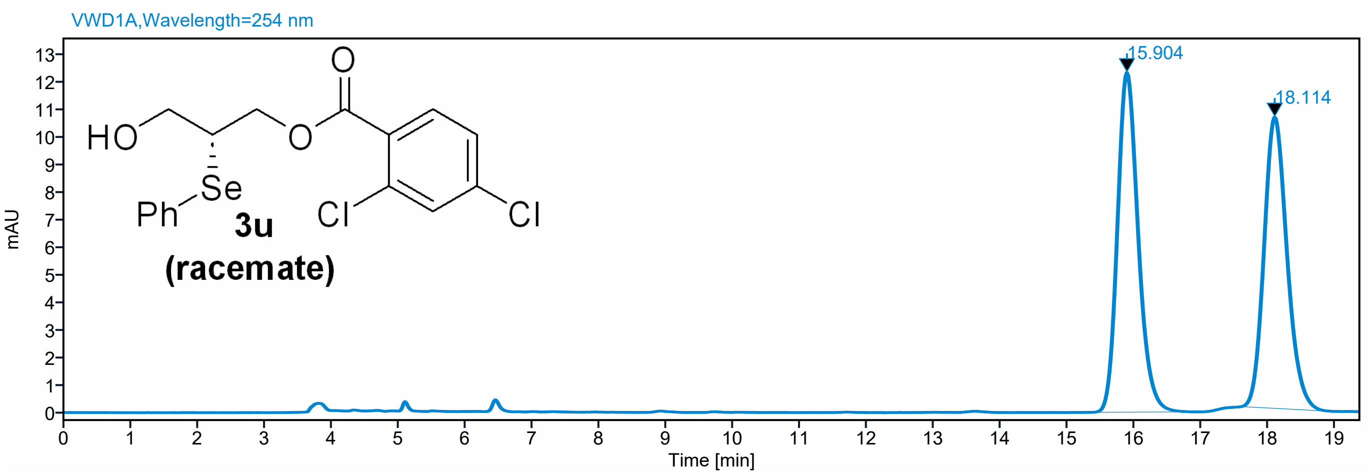

Signal: VWD1A,Wavelength=254 nm

RT [min]	Type	Width [min]	Area	Height	Area%
15.904	BB	1.44	265.41	12.32	51.22
18.114	BB	1.34	252.76	10.56	48.78
		**Sum**	**518.17**		

Single Injection Report

**Data file:**	CJX-2-43-ASY-IB-0.8-10%		
**Sequence Name:**	SingleSample	**Project Name:**	CJX
**Sample name:**	CJX-2-43-ASY-IB-0.8-10%	**Operator:**	SYSTEM (SYSTEM)
**Instrument:**	1260	**Injection date:**	2025-04-03 10:11:24+08:00
**Inj. volume:**	5.000 µL	**Location:**	P1-D2
**Acq. method:**	10%-50min-0.8ml-5uL.amx	**Type:**	Sample
**Processing method:**	GC_LC area percent_DefaultMethod.pmx	**Sample amount:**	0.00
**Manually modified:**	Manual Integration		

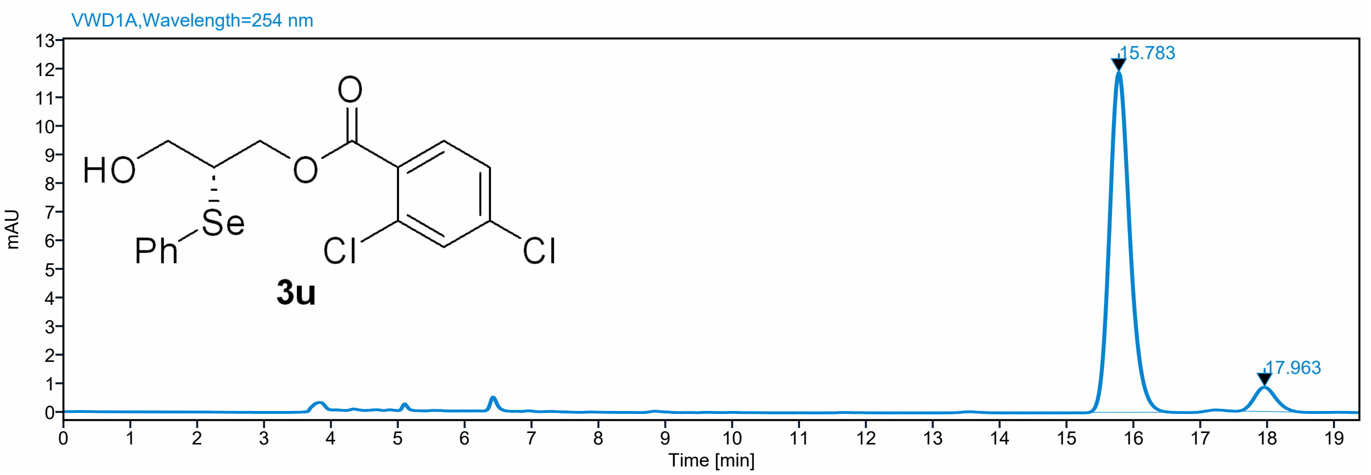

Signal: VWD1A,Wavelength=254 nm

RT [min]	Type	Width [min]	Area	Height	Area%
15.783	BB	1.20	250.59	11.88	92.87
17.963	MM m	1.03	19.25	0.85	7.13
		**Sum**	**269.84**		

Single Injection Report

Agilent

**Data file:**	CJX-2-46-RAC-IB-0.8-5%		
**Sequence Name:**	SingleSample	**Project Name:**	CJX
**Sample name:**	CJX-2-46-RAC-IB-0.8-5%	**Operator:**	SYSTEM (SYSTEM)
**Instrument:**	1260	**Injection date:**	2025-04-04 10:07:44+08:00
**Inj. volume:**	5.000 µL	**Location:**	P1-D1
**Acq. method:**	5%-60min-0.8ml-5uL.amx	**Type:**	Sample
**Processing method:**	GC_LC area percent_DefaultMethod.pmx	**Sample amount:**	0.00
**Manually modified:**	None		

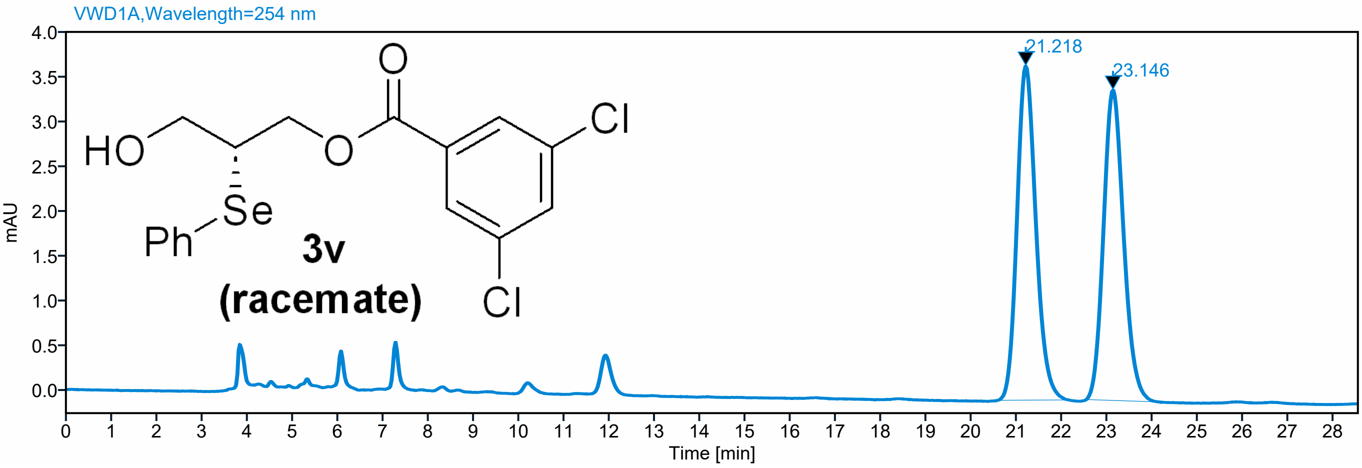

Signal: VWD1A,Wavelength=254 nm

RT [min]	Type	Width [min]	Area	Height	Area%
21.218	BB	1.53	108.20	3.73	50.24
23.146	BB	1.55	107.18	3.47	49.76
		**Sum**	**215.38**		

Single Injection Report

**Data file:**	CJX-2-46-ASY-IB-0.8-5%		
**Sequence Name:**	SingleSample	**Project Name:**	CJX
**Sample name:**	CJX-2-46-ASY-IB-0.8-5%	**Operator:**	SYSTEM (SYSTEM)
**Instrument:**	1260	**Injection date:**	2025-04-04 10:38:26+08:00
**Inj. volume:**	5.000 µL	**Location:**	P1-D2
**Acq. method:**	5%-60min-0.8ml-5uL.amx	**Type:**	Sample
**Processing method:**	GC_LC area percent_DefaultMethod.pmx	**Sample amount:**	0.00
**Manually modified:**	Manual Integration		

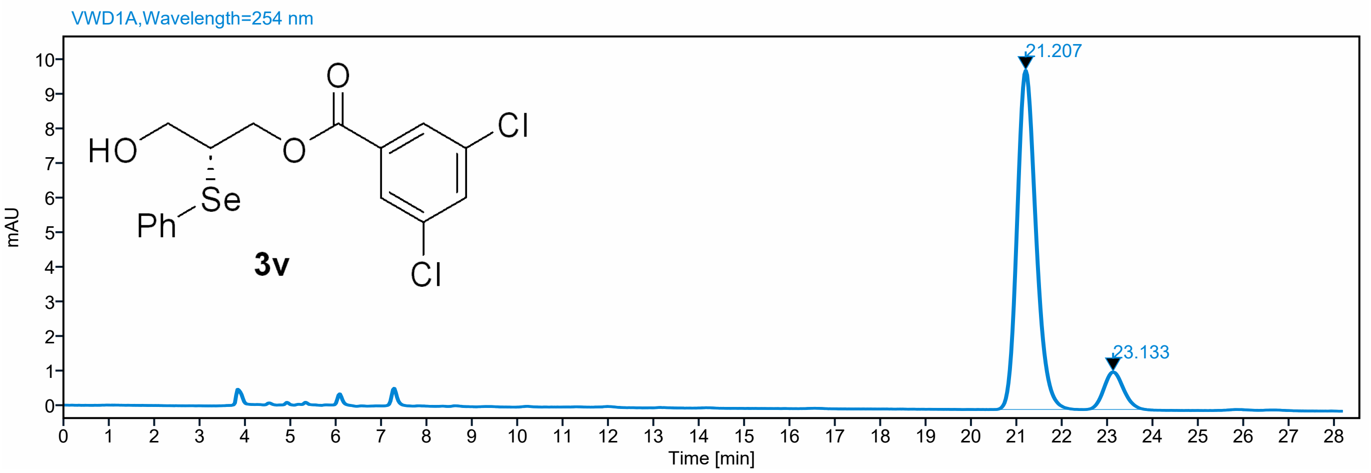

**Signal:**	VWD1A,Wavelength=254 nm				
**RT [min]**	**Type**	**Width [min]**	**Area**	**Height**	**Area%**
21.207	BB	1.73	283.00	9.80	89.65
23.133	MM m	1.31	32.66	1.09	10.35
		**Sum**	**315.66**		

Single Injection Report

**Data file:**	CJX-2-42-RAC-IB-0.8-10%		
**Sequence Name:**	SingleSample	**Project Name:**	CJX
**Sample name:**	CJX-2-42-RAC-IB-0.8-10%	**Operator:**	SYSTEM (SYSTEM)
**Instrument:**	1260	**Injection date:**	2025-04-02 15:29:57+08:00
**Inj. volume:**	5.000 µL	**Location:**	P1-D1
**Acq. method:**	10%-30min-0.8ml-5uL.amx	**Type:**	Sample
**Processing method:**	GC_LC area percent_DefaultMethod.pmx	**Sample amount:**	0.00
**Manually modified:**	None		

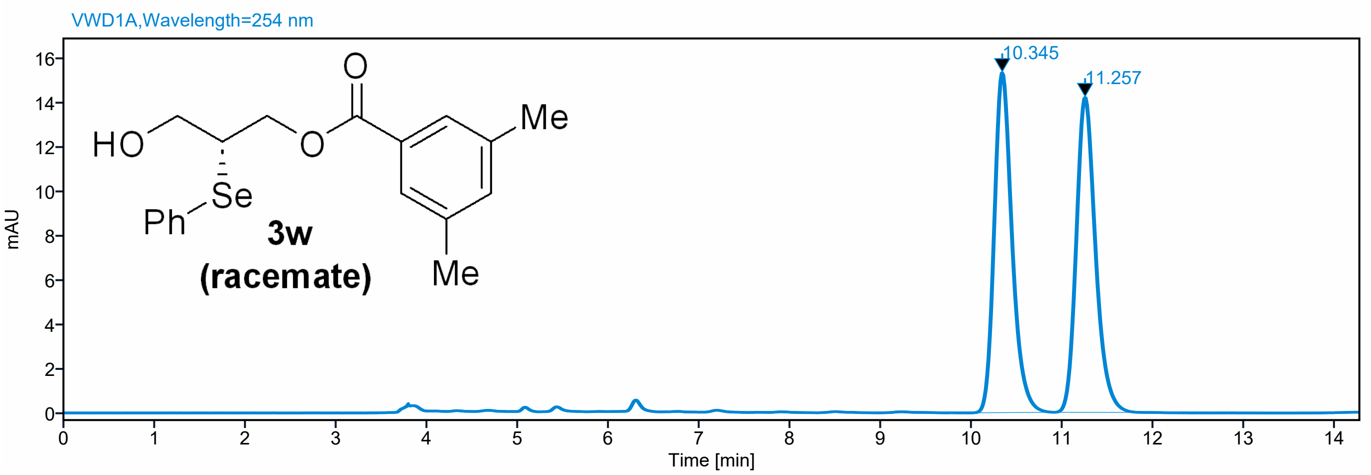

Signal: VWD1A,Wavelength=254 nm

RT [min]	Type	Width [min]	Area	Height	Area%
10.345	BB	0.90	209.67	15.33	50.00
11.257	BB	0.95	209.65	14.20	50.00
		**Sum**	**419.32**		

Single Injection Report

Agilent

**Data file:**	CJX-2-42-ASY-IB-0.8-10%		
**Sequence Name:**	SingleSample	**Project Name:**	CJX
**Sample name:**	CJX-2-42-ASY-IB-0.8-10%	**Operator:**	SYSTEM (SYSTEM)
**Instrument:**	1260	**Injection date:**	2025-04-02 15:46:38+08:00
**Inj. volume:**	5.000 µL	**Location:**	P1-D2
**Acq. method:**	10%-30min-0.8ml-5uL.amx	**Type:**	Sample
**Processing method:**	GC_LC area percent_DefaultMethod.pmx	**Sample amount:**	0.00
**Manually modified:**	Manual Integration		

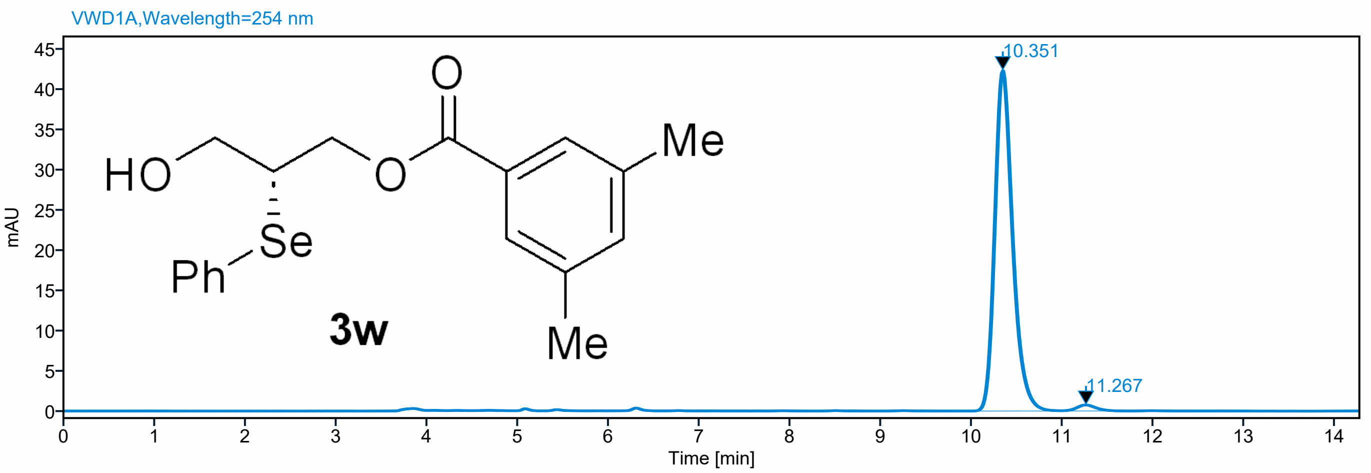

Signal: VWD1A,Wavelength=254 nm

RT [min]	Type	Width [min]	Area	Height	Area%
10.351	BM m	0.99	578.56	42.30	98.15
11.267	MM m	0.85	10.92	0.74	1.85
		**Sum**	**589.47**		

Single Injection Report

**Data file:**	CJX-2-38-RAC-IB-1.0-20%		
**Sequence Name:**	SingleSample	**Project Name:**	CJX
**Sample name:**	CJX-2-38-RAC-IB-1.0-20%	**Operator:**	SYSTEM (SYSTEM)
**Instrument:**	1260	**Injection date:**	2025-04-01 21:11:16+08:00
**Inj. volume:**	5.000 µL	**Location:**	P1-D3
**Acq. method:**	20%-50min-1.0ml-5uL.amx	**Type:**	Sample
**Processing method:**	GC_LC area percent_DefaultMethod.pmx	**Sample amount:**	0.00
**Manually modified:**	None		

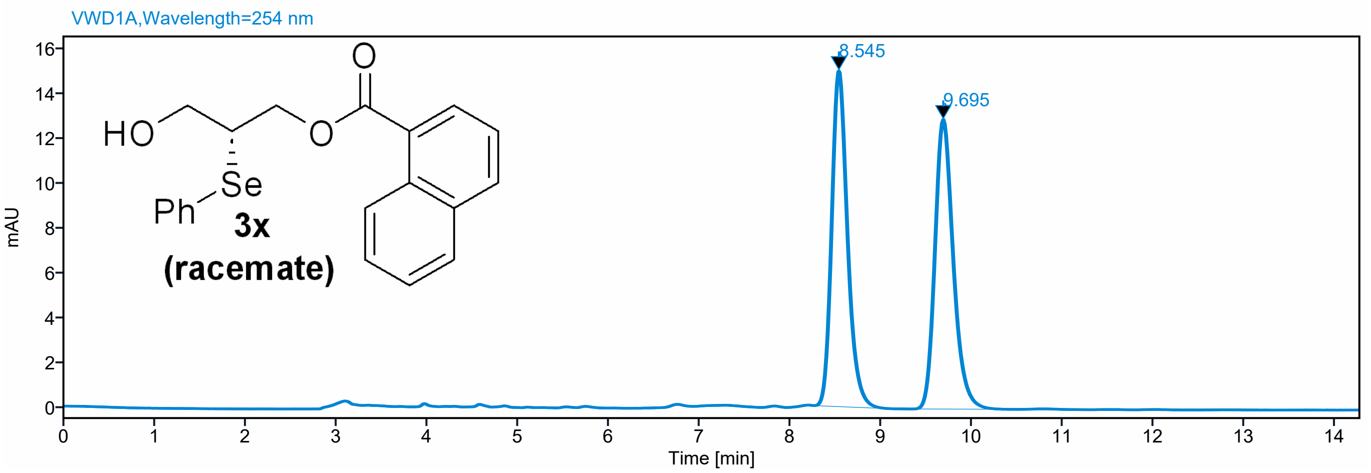

Signal: VWD1A,Wavelength=254 nm

RT [min]	Type	Width [min]	Area	Height	Area%
8.545	BB	0.83	181.08	14.99	49.88
9.695	BB	0.99	181.93	12.91	50.12
		**Sum**	**363.01**		

Single Injection Report

**Data file:**	CJX-2-38-ASY-IB-1.0-20%		
**Sequence Name:**	SingleSample	**Project Name:**	CJX
**Sample name:**	CJX-2-38-ASY-IB-1.0-20%	**Operator:**	SYSTEM (SYSTEM)
**Instrument:**	1260	**Injection date:**	2025-04-02 10:51:12+08:00
**Inj. volume:**	5.000 µL	**Location:**	P1-D1
**Acq. method:**	20%-50min-1.0ml-5uL.amx	**Type:**	Sample
**Processing method:**	GC_LC area percent_DefaultMethod.pmx	**Sample amount:**	0.00
**Manually modified:**	Manual Integration		

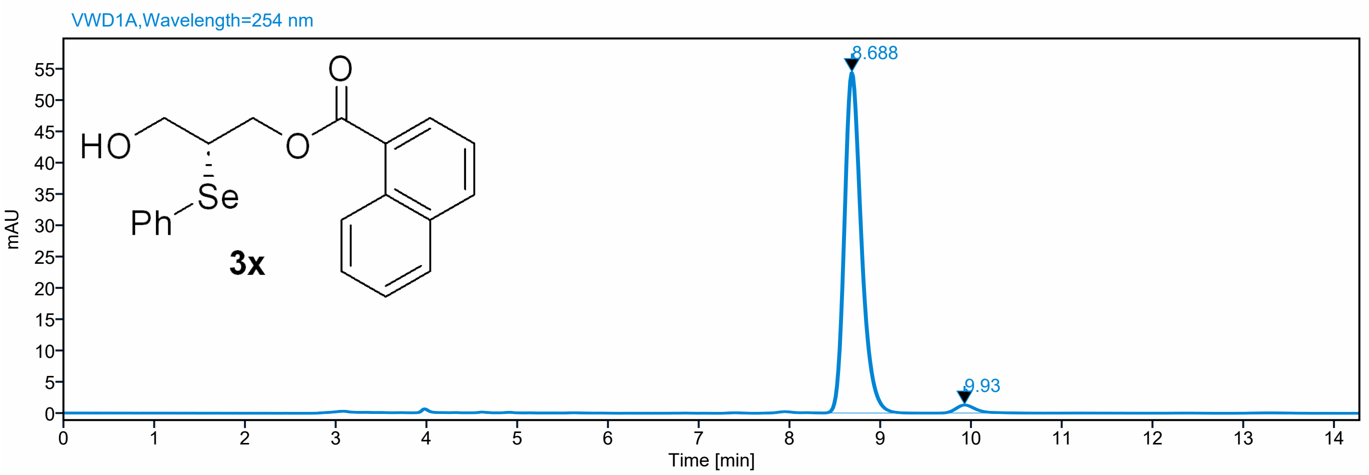

Signal: VWD1A,Wavelength=254 nm

RT [min]	Type	Width [min]	Area	Height	Area%
8.688	BB	1.07	708.43	54.39	97.32
9.930	MM m	0.87	19.54	1.28	2.68
		**Sum**	**727.97**		

Single Injection Report

**Data file:**	CJX-2-44-RAC-IA-1.0-10%		
**Sequence Name:**	SingleSample	**Project Name:**	CJX
**Sample name:**	CJX-2-44-RAC-IA-1.0-10%	**Operator:**	SYSTEM (SYSTEM)
**Instrument:**	1260	**Injection date:**	2025-04-03 17:03:05+08:00
**Inj. volume:**	5.000 µL	**Location:**	P1-D1
**Acq. method:**	10%-60min-1.0ml-5uL.amx	**Type:**	Sample
**Processing method:**	GC_LC area percent_DefaultMethod.pmx	**Sample amount:**	0.00
**Manually modified:**	Manual Integration		

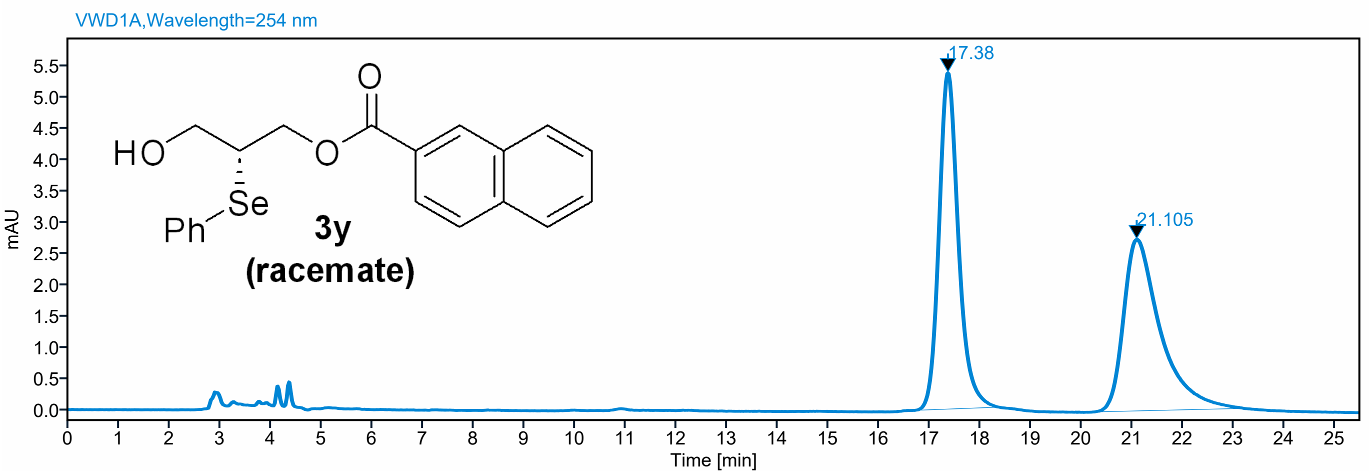

Signal: VWD1A,Wavelength=254 nm

RT [min]	Type	Width [min]	Area	Height	Area%
17.380	MM m	1.66	143.42	5.38	50.90
21.105	MM m	2.95	138.35	2.74	49.10
		**Sum**	**281.77**		

Single Injection Report

Agilent

Data file: CJX-2-44-ASY-IA-1.0-10%

**Sequence Name:**	SingleSample	**Project Name:**	CJX
**Sample name:**	CJX-2-44-ASY-IA-1.0-10%	**Operator:**	SYSTEM (SYSTEM)
**Instrument:**	1260	**Injection date:**	2025-04-03 16:36:46+08:00
**Inj. volume:**	5.000 µL	**Location:**	P1-D2
**Acq. method:**	10%-60min-1.0ml-5uL.amx	**Type:**	Sample
**Processing method:**	GC_LC area percent_DefaultMethod.pmx	**Sample amount:**	0.00
**Manually modified:**	Manual Integration		

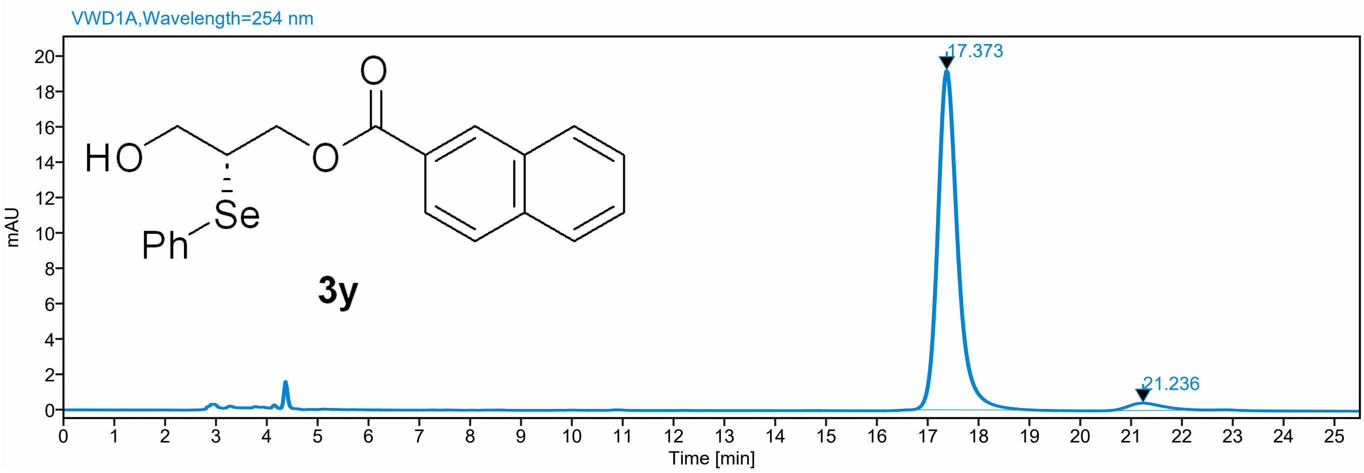

Signal: VWD1A,Wavelength=254 nm

RT [min]	Type	Width [min]	Area	Height	Area%
17.373	BB	2.41	524.58	19.19	96.30
21.236	MM m	2.06	20.13	0.42	3.70
		**Sum**	**544.71**		

Single Injection Report

**Data file:**	CJX-2-132-RAC-IB-1.0-10%		
**Sequence Name:**	SingleSample	**Project Name:**	CJX
**Sample name:**	CJX-2-132-RAC-IB-1.0-10%	**Operator:**	SYSTEM (SYSTEM)
**Instrument:**	1260	**Injection date:**	2025-05-30 22:13:37+08:00
**Inj. volume:**	5.000 µL	**Location:**	P2-D1
**Acq. method:**	10%-30min-1.0ml-5uL.amx	**Type:**	Sample
**Processing method:**	GC_LC area percent_DefaultMethod.pmx	**Sample amount:**	0.00
**Manually modified:**	None		

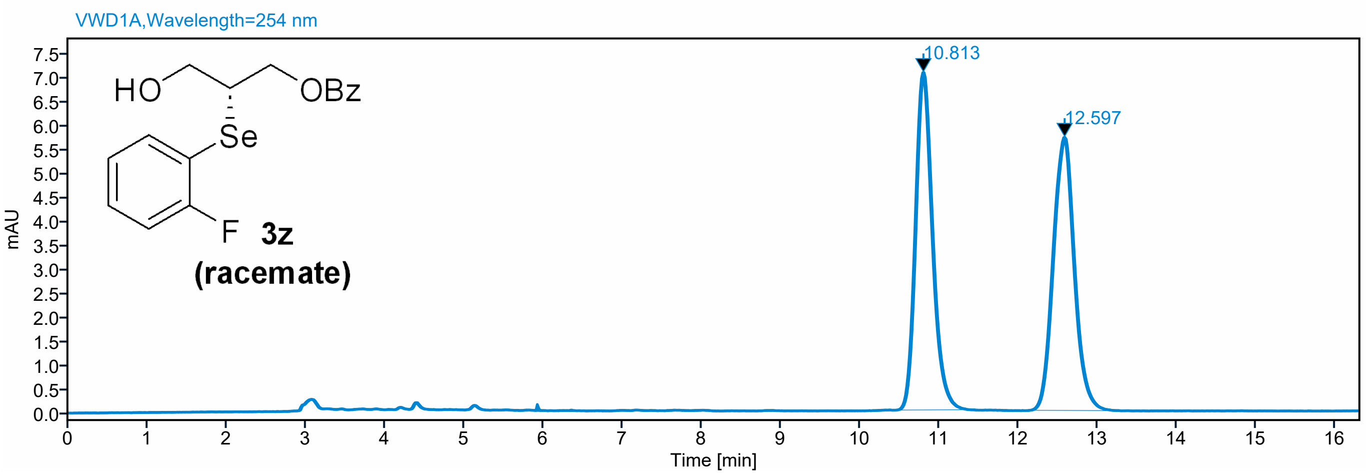

Signal: VWD1A,Wavelength=254 nm

RT [min]	Type	Width [min]	Area	Height	Area%
10.813	BB	0.90	104.81	7.03	49.86
12.597	BB	1.08	105.39	5.69	50.14
		**Sum**	**210.20**		

Single Injection Report

**Data file:**	CJX-2-132-ASY-IB-1.0-10%		
**Sequence Name:**	SingleSample	**Project Name:**	CJX
**Sample name:**	CJX-2-132-ASY-IB-1.0-10%	**Operator:**	SYSTEM (SYSTEM)
**Instrument:**	1260	**Injection date:**	2025-05-30 22:32:17+08:00
**Inj. volume:**	5.000 µL	**Location:**	P2-D2
**Acq. method:**	10%-30min-1.0ml-5uL.amx	**Type:**	Sample
**Processing method:**	GC_LC area percent_DefaultMethod.pmx	**Sample amount:**	0.00
**Manually modified:**	Manual Integration		

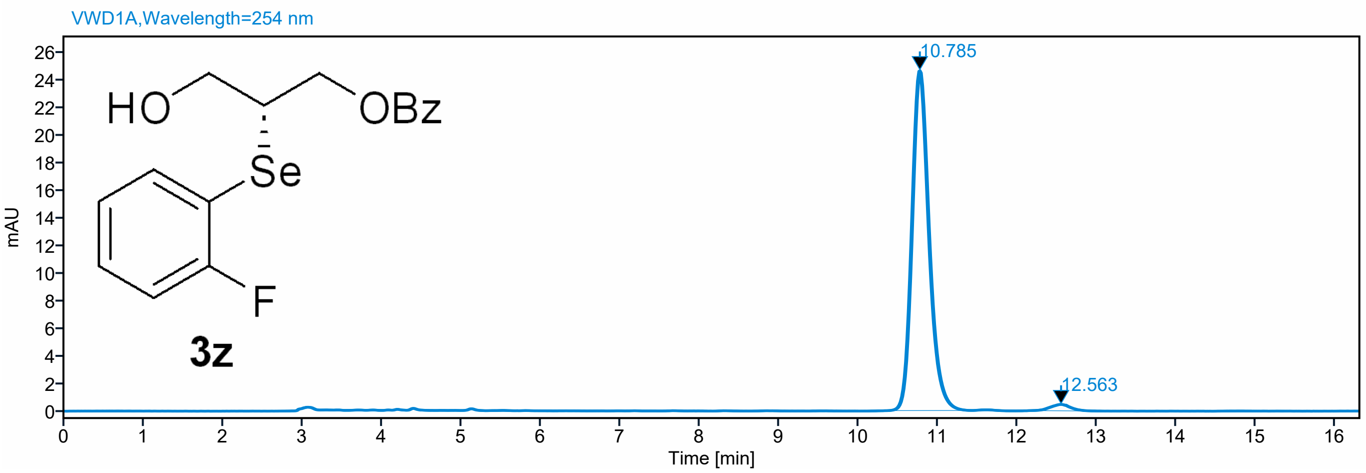

Signal: VWD1A,Wavelength=254 nm

RT [min]	Type	Width [min]	Area	Height	Area%
10.785	BB	1.05	368.24	24.62	97.83
12.563	MM m	0.83	8.16	0.46	2.17
		**Sum**	**376.40**		

Single Injection Report

**Data file:**	CJX-2-134-RAC-IC-1.0-10%		
**Sequence Name:**	SingleSample	**Project Name:**	CJX
**Sample name:**	CJX-2-134-RAC-IC-1.0-10%	**Operator:**	SYSTEM (SYSTEM)
**Instrument:**	1260	**Injection date:**	2025-05-31 23:16:31+08:00
**Inj. volume:**	5.000 µL	**Location:**	P2-D1
**Acq. method:**	10%-30min-1.0ml-5uL.amx	**Type:**	Sample
**Processing method:**	GC_LC area percent_DefaultMethod.pmx	**Sample amount:**	0.00
**Manually modified:**	None		

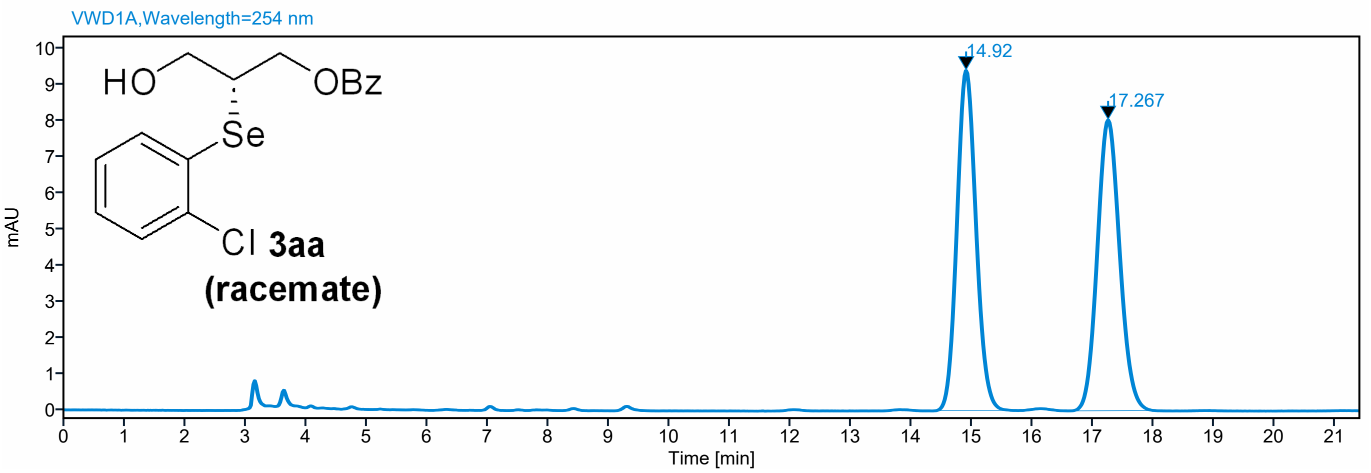

Signal: VWD1A,Wavelength=254 nm

RT [min]	Type	Width [min]	Area	Height	Area%
14.920	BB	1.23	206.31	9.40	49.93
17.267	BB	1.34	206.87	8.04	50.07
		**Sum**	**413.18**		

Single Injection Report

Agilent

**Data file:**	CJX-2-134-ASY-IC-1.0-10%		
**Sequence Name:**	SingleSample	**Project Name:**	CJX
**Sample name:**	CJX-2-134-ASY-IC-1.0-10%	**Operator:**	SYSTEM (SYSTEM)
**Instrument:**	1260	**Injection date:**	2025-05-31 23:39:10+08:00
**Inj. volume:**	5.000 µL	**Location:**	P2-D2
**Acq. method:**	10%-30min-1.0ml-5uL.amx	**Type:**	Sample
**Processing method:**	GC_LC area percent_DefaultMethod.pmx	**Sample amount:**	0.00
**Manually modified:**	Manual Integration		

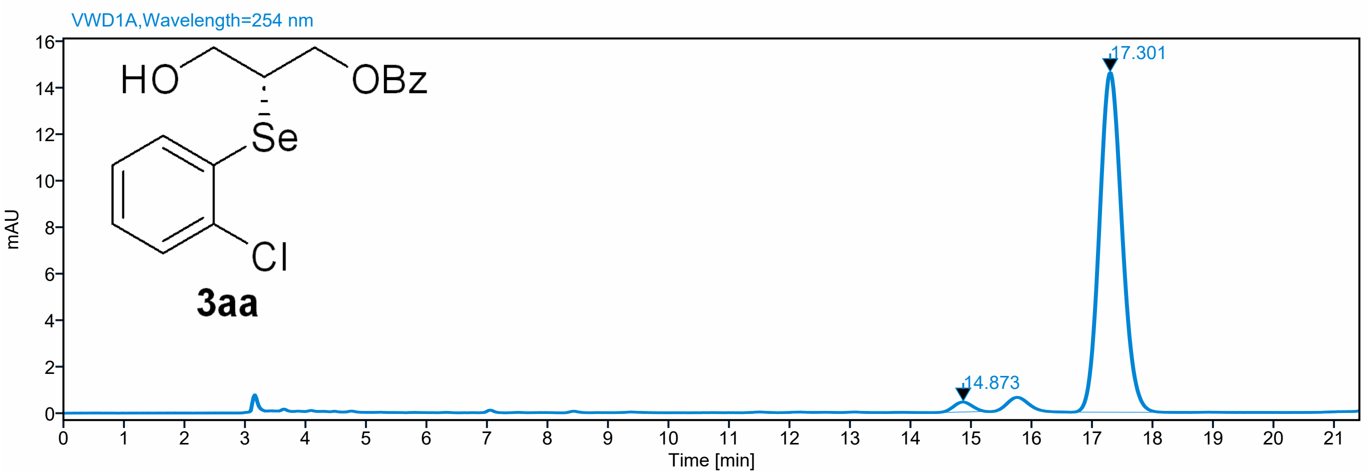

Signal: VWD1A,Wavelength=254 nm

RT [min]	Type	Width [min]	Area	Height	Area%
14.873	MM m	0.96	9.24	0.43	2.38
17.301	BB	1.47	379.55	14.61	97.62
		**Sum**	**388.79**		

Single Injection Report

Agilent

**Data file:**	CJX-2-146-RAC-IC-1.0-10%		
**Sequence Name:**	SingleSample	**Project Name:**	CJX
**Sample name:**	CJX-2-146-RAC-IC-1.0-10%	**Operator:**	SYSTEM (SYSTEM)
**Instrument:**	1260	**Injection date:**	2025-06-06 21:09:49+08:00
**Inj. volume:**	5.000 µL	**Location:**	P2-D1
**Acq. method:**	10%-60min-1.0ml-5uL.amx	**Type:**	Sample
**Processing method:**	GC_LC area percent_DefaultMethod.pmx	**Sample amount:**	0.00
**Manually modified:**	Manual Integration		

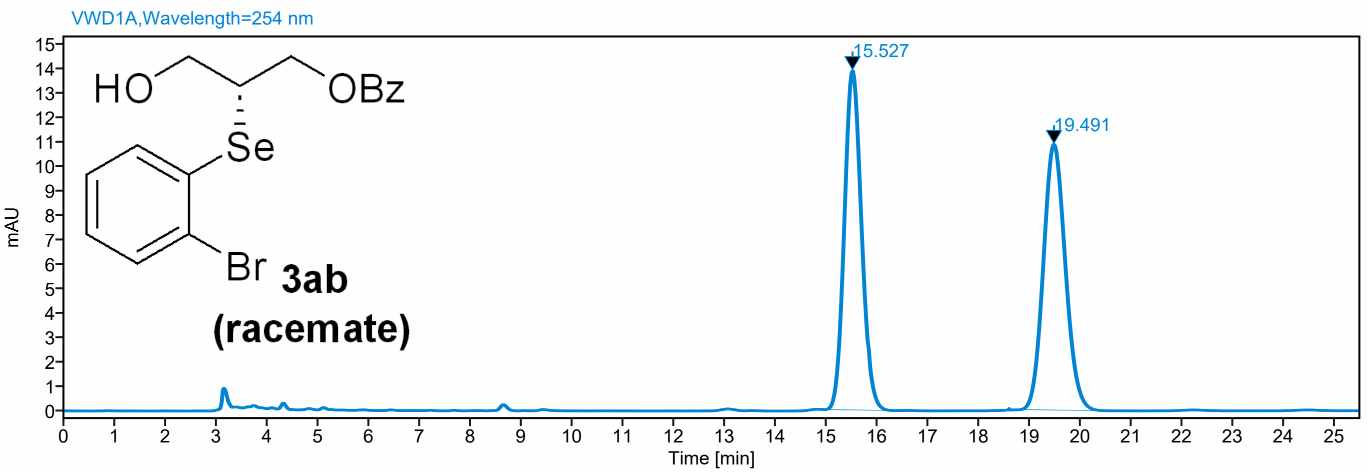

Signal: VWD1A,Wavelength=254 nm

RT [min]	Type	Width [min]	Area	Height	Area%
15.527	BM m	1.31	326.07	13.89	49.89
19.491	BB	1.60	327.46	10.87	50.11
		**Sum**	**653.52**		

Single Injection Report

Agilent

**Data file:**	CJX-2-146-ASY-IC-1.0-10%		
**Sequence Name:**	SingleSample	**Project Name:**	CJX
**Sample name:**	CJX-2-146-ASY-IC-1.0-10%	**Operator:**	SYSTEM (SYSTEM)
**Instrument:**	1260	**Injection date:**	2025-06-06 21:36:29+08:00
**Inj. volume:**	5.000 µL	**Location:**	P2-D2
**Acq. method:**	10%-60min-1.0ml-5uL.amx	**Type:**	Sample
**Processing method:**	GC_LC area percent_DefaultMethod.pmx	**Sample amount:**	0.00
**Manually modified:**	Manual Integration		

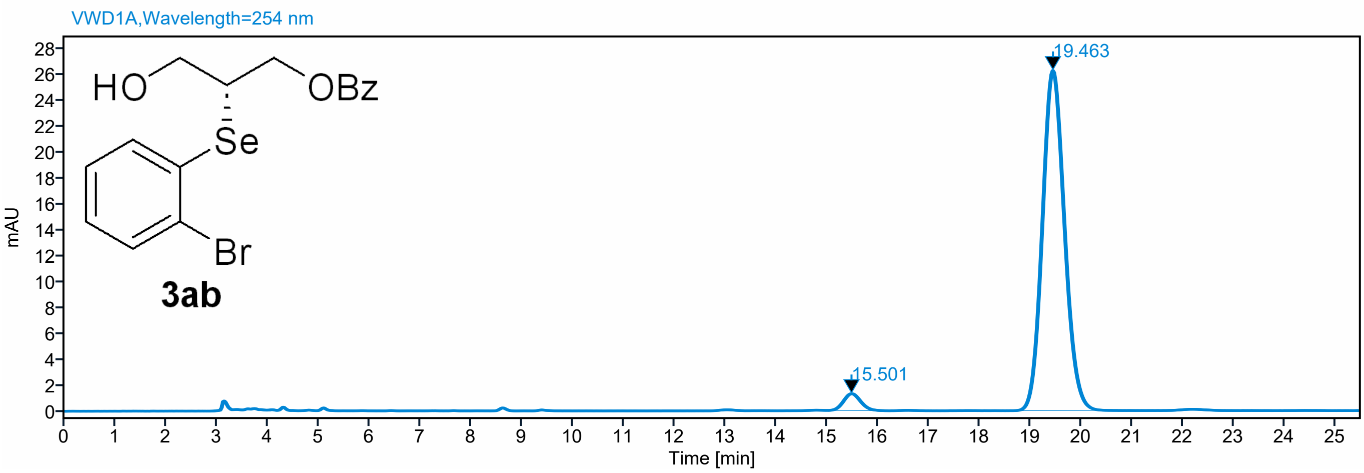

Signal: VWD1A,Wavelength=254 nm

RT [min]	Type	Width [min]	Area	Height	Area%
15.501	MM m	1.34	30.12	1.30	3.66
19.463	BB	1.76	792.79	26.23	96.34
		**Sum**	**822.91**		

Single Injection Report

Agilent

**Data file:**	CJX-2-117-RAC-IB-1.0-10%		
**Sequence Name:**	SingleSample	**Project Name:**	CJX
**Sample name:**	CJX-2-117-RAC-IB-1.0-10%	**Operator:**	SYSTEM (SYSTEM)
**Instrument:**	1260	**Injection date:**	2025-05-23 22:51:45+08:00
**Inj. volume:**	5.000 µL	**Location:**	P2-D1
**Acq. method:**	10%-30min-1.0ml-5uL.amx	**Type:**	Sample
**Processing method:**	GC_LC area percent_DefaultMethod.pmx	**Sample amount:**	0.00
**Manually modified:**	None		

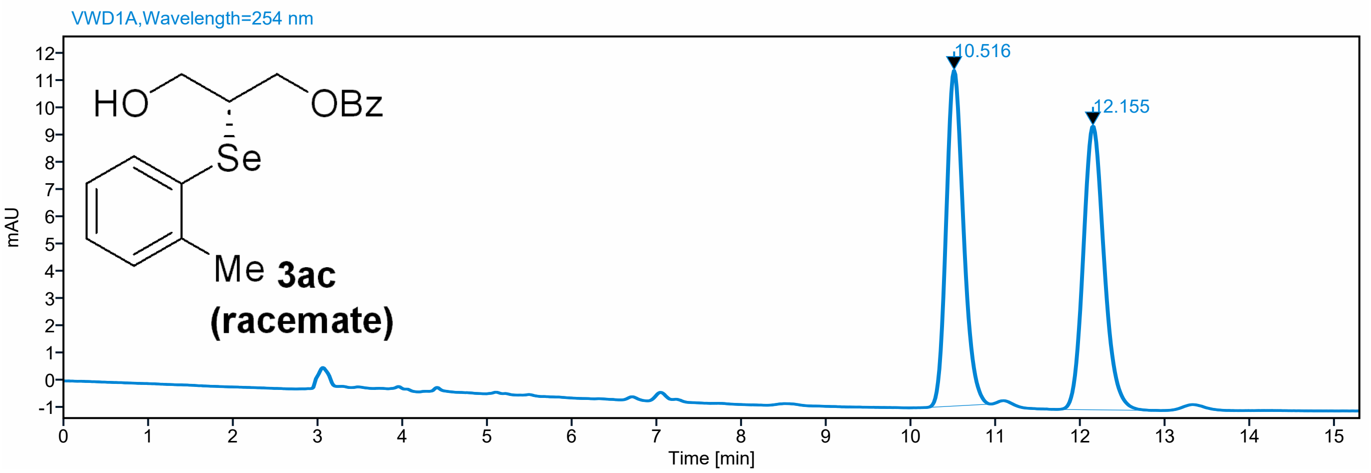

Signal: VWD1A,Wavelength=254 nm

RT [min]	Type	Width [min]	Area	Height	Area%
10.516	BB	0.76	175.23	12.32	49.48
12.155	BB	1.11	178.94	10.42	50.52
		**Sum**	**354.17**		

Single Injection Report

Agilent

**Data file:**	CJX-2-117-ASY-IB-1.0-10%		
**Sequence Name:**	SingleSample	**Project Name:**	CJX
**Sample name:**	CJX-2-117-ASY-IB-1.0-10%	**Operator:**	SYSTEM (SYSTEM)
**Instrument:**	1260	**Injection date:**	2025-05-23 23:10:23+08:00
**Inj. volume:**	5.000 µL	**Location:**	P2-D2
**Acq. method:**	10%-30min-1.0ml-5uL.amx	**Type:**	Sample
**Processing method:**	GC_LC area percent_DefaultMethod.pmx	**Sample amount:**	0.00
**Manually modified:**	Manual Integration		

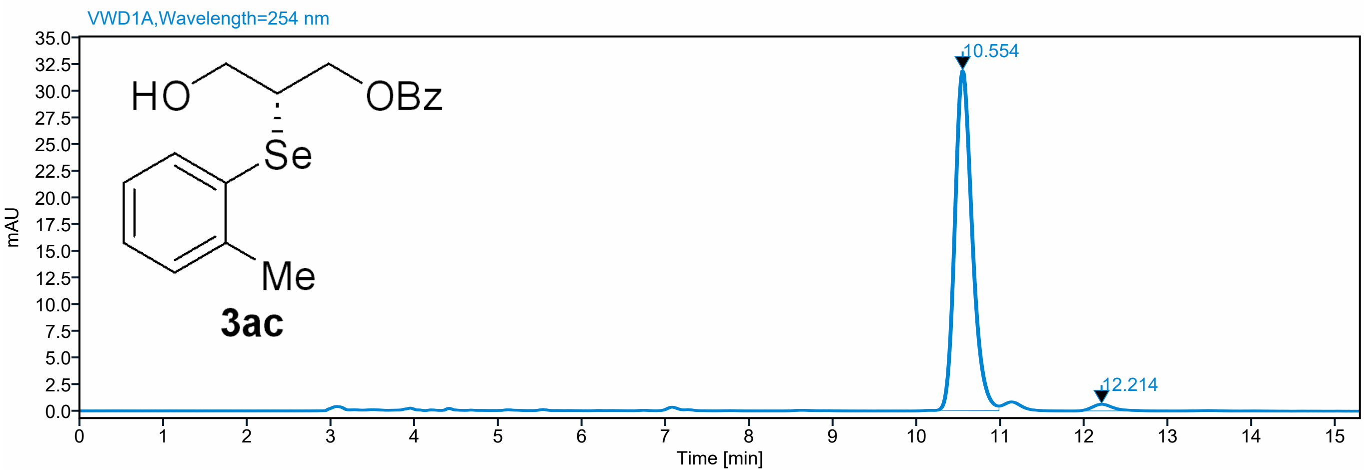

Signal: VWD1A,Wavelength=254 nm

RT [min]	Type	Width [min]	Area	Height	Area%
10.554	BV	0.76	460.29	31.87	97.65
12.214	MM m	0.88	11.06	0.63	2.35
		**Sum**	**471.35**		

Single Injection Report

**Data file:**	CJX-2-133-RAC-IB-1.0-10%		
**Sequence Name:**	SingleSample	**Project Name:**	CJX
**Sample name:**	CJX-2-133-RAC-IB-1.0-10%	**Operator:**	SYSTEM (SYSTEM)
**Instrument:**	1260	**Injection date:**	2025-05-30 22:50:54+08:00
**Inj. volume:**	5.000 µL	**Location:**	P2-D3
**Acq. method:**	10%-30min-1.0ml-5uL.amx	**Type:**	Sample
**Processing method:**	GC_LC area percent_DefaultMethod.pmx	**Sample amount:**	0.00
**Manually modified:**	None		

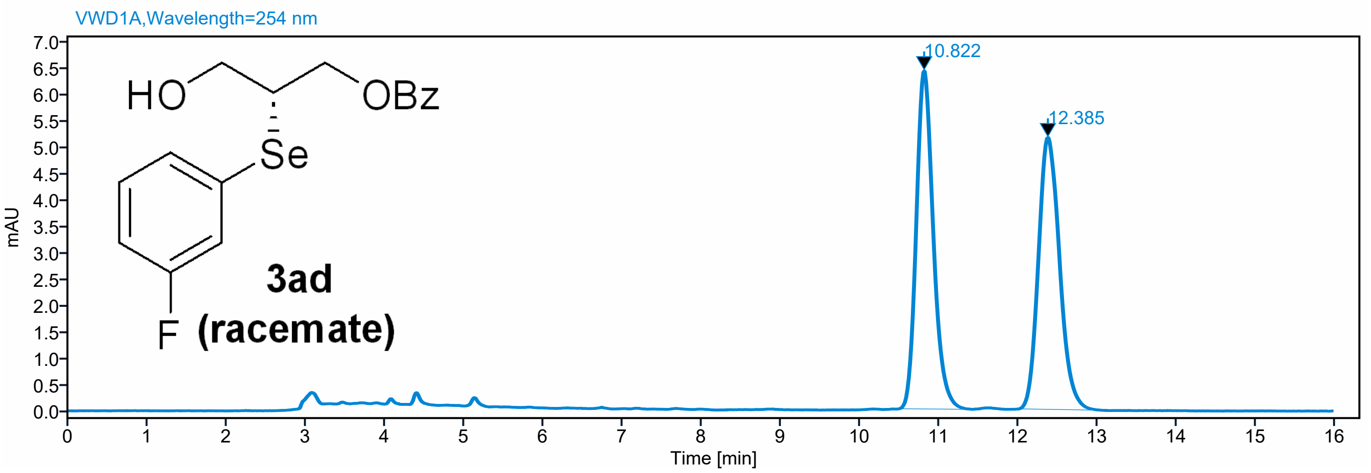

Signal: VWD1A,Wavelength=254 nm

RT [min]	Type	Width [min]	Area	Height	Area%
10.822	BB	0.90	95.77	6.41	50.03
12.385	BB	1.01	95.66	5.15	49.97
		**Sum**	**191.42**		

Single Injection Report

Agilent

**Data file:**	CJX-2-133-ASY-IB-1.0-10%		
**Sequence Name:**	SingleSample	**Project Name:**	CJX
**Sample name:**	CJX-2-133-ASY-IB-1.0-10%	**Operator:**	SYSTEM (SYSTEM)
**Instrument:**	1260	**Injection date:**	2025-05-30 23:07:32+08:00
**Inj. volume:**	5.000 µL	**Location:**	P2-D4
**Acq. method:**	10%-30min-1.0ml-5uL.amx	**Type:**	Sample
**Processing method:**	GC_LC area percent_DefaultMethod.pmx	**Sample amount:**	0.00
**Manually modified:**	Manual Integration		

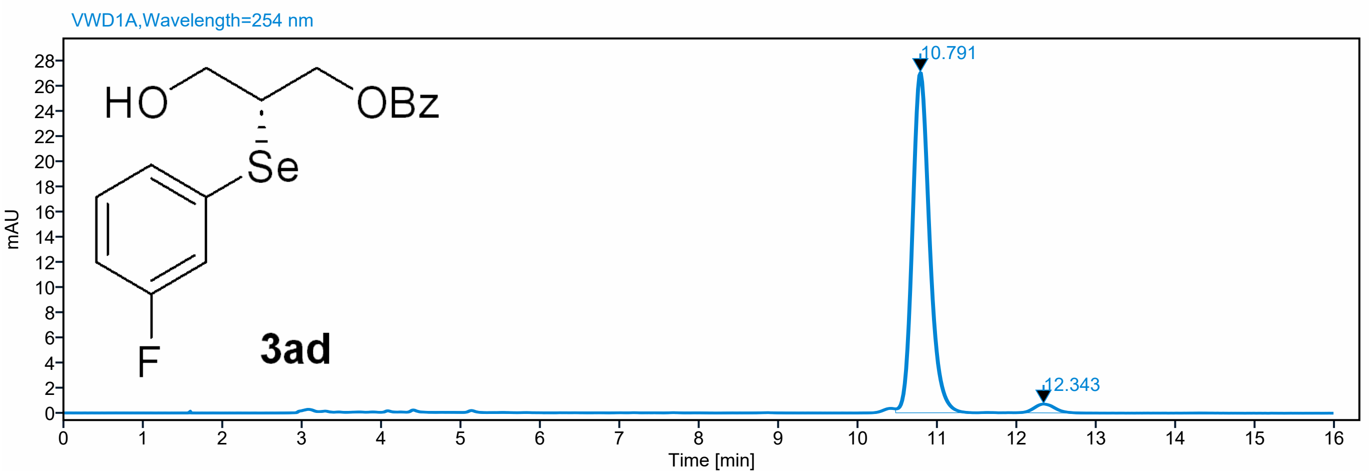

Signal: VWD1A,Wavelength=254 nm

RT [min]	Type	Width [min]	Area	Height	Area%
10.791	VB	0.97	405.00	27.05	96.95
12.343	MM m	0.88	12.73	0.70	3.05
		**Sum**	**417.73**		

Single Injection Report

**Data file:**	CJX-2-107-RAC-IB-1.0-10%		
**Sequence Name:**	SingleSample	**Project Name:**	CJX
**Sample name:**	CJX-2-107-RAC-IB-1.0-10%	**Operator:**	SYSTEM (SYSTEM)
**Instrument:**	1260	**Injection date:**	2025-06-21 10:45:46+08:00
**Inj. volume:**	5.000 µL	**Location:**	P1-E1
**Acq. method:**	10%-30min-1.0ml-5uL.amx	**Type:**	Sample
**Processing method:**	GC_LC area percent_DefaultMethod.pmx	**Sample amount:**	0.00
**Manually modified:**	None		

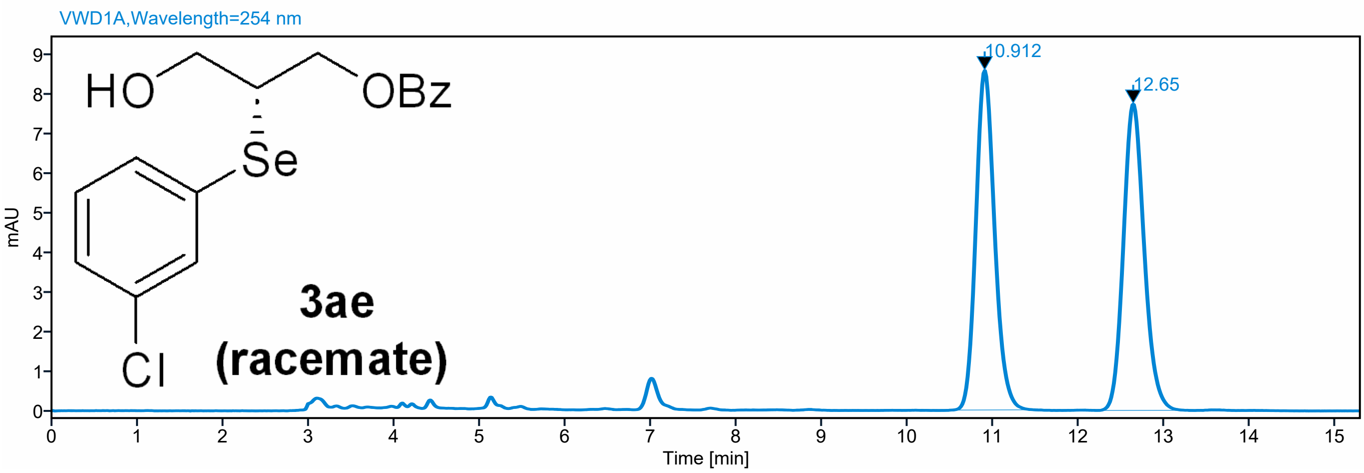

Signal: VWD1A,Wavelength=254 nm

RT [min]	Type	Width [min]	Area	Height	Area%
10.912	BB	0.94	130.83	8.57	50.06
12.650	BB	1.00	130.54	7.73	49.94
		**Sum**	**261.37**		

Single Injection Report

**Data file:**	CJX-2-107-ASY-IB-1.0-10%		
**Sequence Name:**	SingleSample	**Project Name:**	CJX
**Sample name:**	CJX-2-107-ASY-IB-1.0-10%	**Operator:**	SYSTEM (SYSTEM)
**Instrument:**	1260	**Injection date:**	2025-06-21 11:04:27+08:00
**Inj. volume:**	5.000 µL	**Location:**	P1-E2
**Acq. method:**	10%-30min-1.0ml-5uL.amx	**Type:**	Sample
**Processing method:**	GC_LC area percent_DefaultMethod.pmx	**Sample amount:**	0.00
**Manually modified:**	Manual Integration		

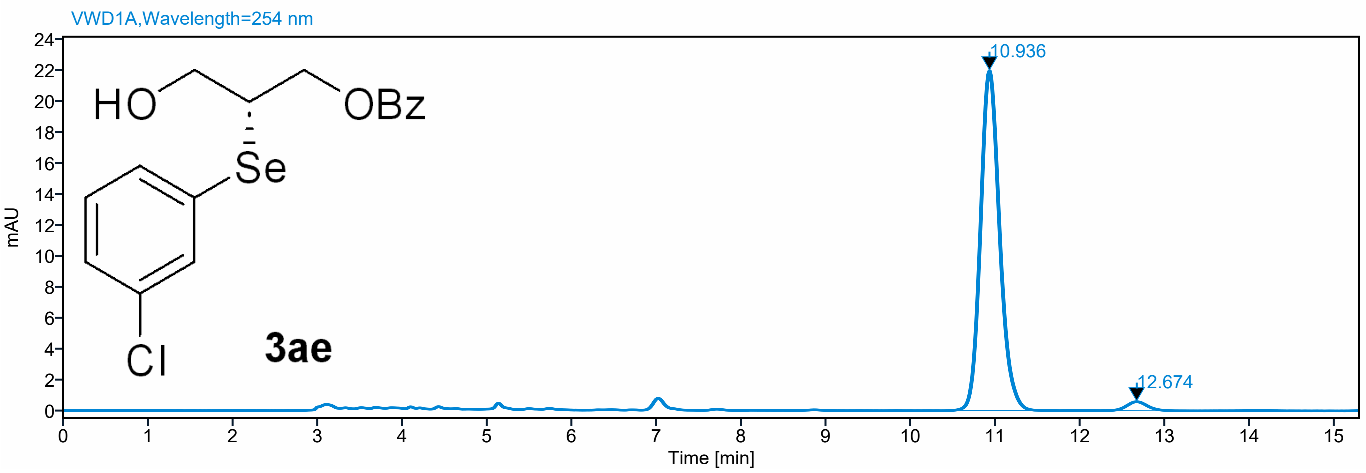

Signal: VWD1A,Wavelength=254 nm

RT [min]	Type	Width [min]	Area	Height	Area%
10.936	BB	1.09	344.39	21.95	97.27
12.674	MM m	0.77	9.68	0.58	2.73
		**Sum**	**354.07**		

Single Injection Report

Agilent

**Data file:**	CJX-2-108-RAC-IB-1.0-10%		
**Sequence Name:**	SingleSample	**Project Name:**	CJX
**Sample name:**	CJX-2-108-RAC-IB-1.0-10%	**Operator:**	SYSTEM (SYSTEM)
**Instrument:**	1260	**Injection date:**	2025-06-21 11:23:09+08:00
**Inj. volume:**	5.000 µL	**Location:**	P1-E3
**Acq. method:**	10%-30min-1.0ml-5uL.amx	**Type:**	Sample
**Processing method:**	GC_LC area percent_DefaultMethod.pmx	**Sample amount:**	0.00
**Manually modified:**	None		

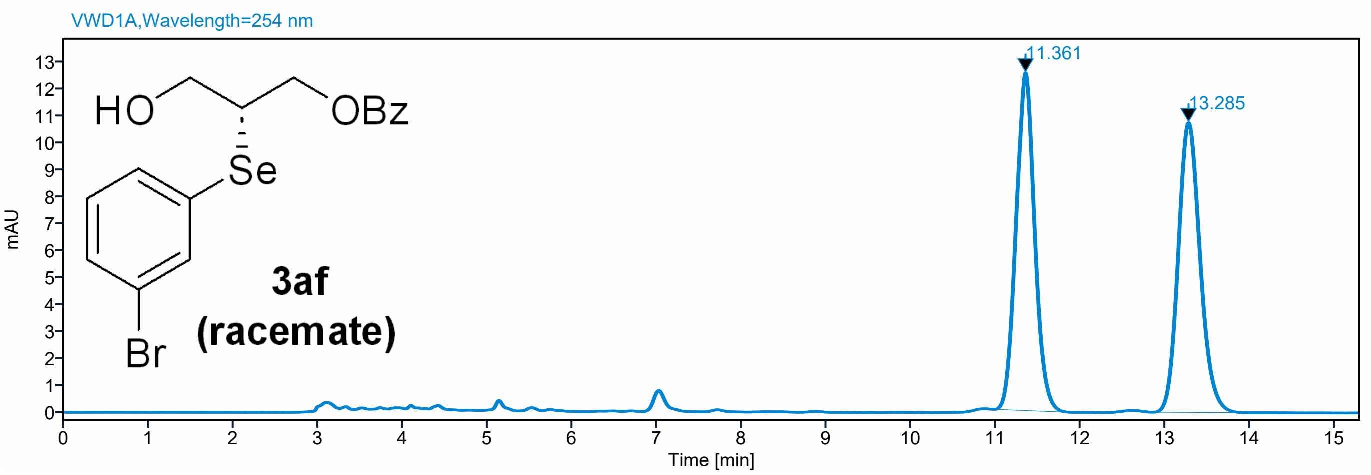

Signal: VWD1A,Wavelength=254 nm

RT [min]	Type	Width [min]	Area	Height	Area%
11.361	BB	0.93	192.02	12.53	49.83
13.285	BB	1.07	193.35	10.75	50.17
		**Sum**	**385.37**		

Single Injection Report

**Data file:**	CJX-2-108-ASY-IB-1.0-10%		
**Sequence Name:**	SingleSample	**Project Name:**	CJX
**Sample name:**	CJX-2-108-ASY-IB-1.0-10%	**Operator:**	SYSTEM (SYSTEM)
**Instrument:**	1260	**Injection date:**	2025-06-20 16:30:52+08:00
**Inj. volume:**	5.000 µL	**Location:**	P1-D2
**Acq. method:**	10%-60min-1.0ml-5uL.amx	**Type:**	Sample
**Processing method:**	GC_LC 面积百分比_DefaultMethod.pmx	**Sample amount:**	0.00
**Manually modified:**	Manual Integration		

VWD1A,Wavelength=254 nm

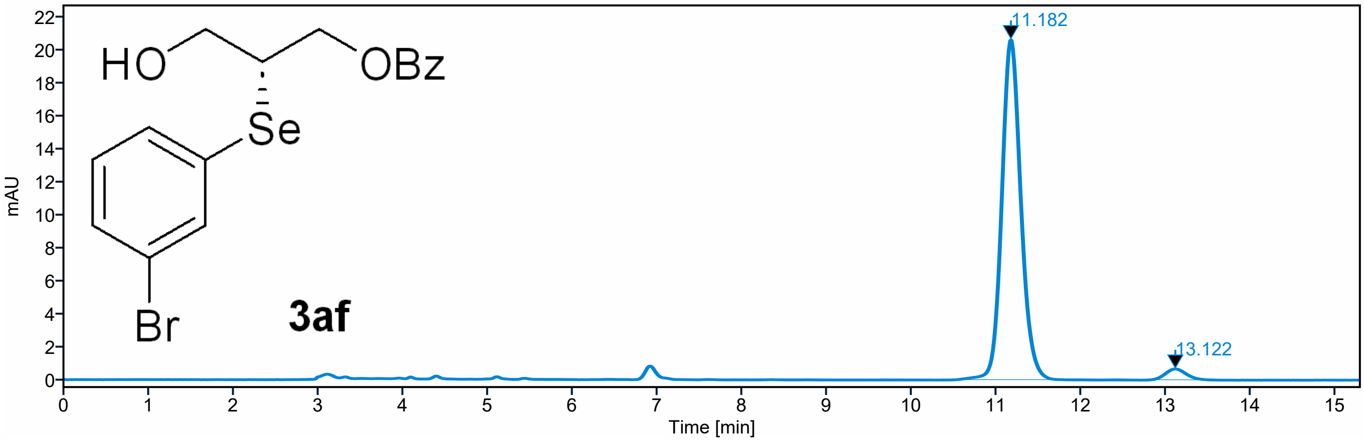

Signal: VWD1A,Wavelength=254 nm

RT [min]	Type	Width [min]	Area	Height	Area%
11.182	BB	1.22	322.48	20.62	96.70
13.122	MM m	0.68	11.01	0.66	3.30
		**Sum**	**333.49**		

Single Injection Report

**Data file:**	CJX-2-104-RAC-IB-1.0-10%		
**Sequence Name:**	SingleSample	**Project Name:**	CJX
**Sample name:**	CJX-2-104-RAC-IB-1.0-10%	**Operator:**	SYSTEM (SYSTEM)
**Instrument:**	1260	**Injection date:**	2025-06-19 10:52:20+08:00
**Inj. volume:**	5.000 µL	**Location:**	P1-C3
**Acq. method:**	10%-60min-1.0ml-5uL.amx	**Type:**	Sample
**Processing method:**	GC_LC area percent_DefaultMethod.pmx	**Sample amount:**	0.00
**Manually modified:**	None		

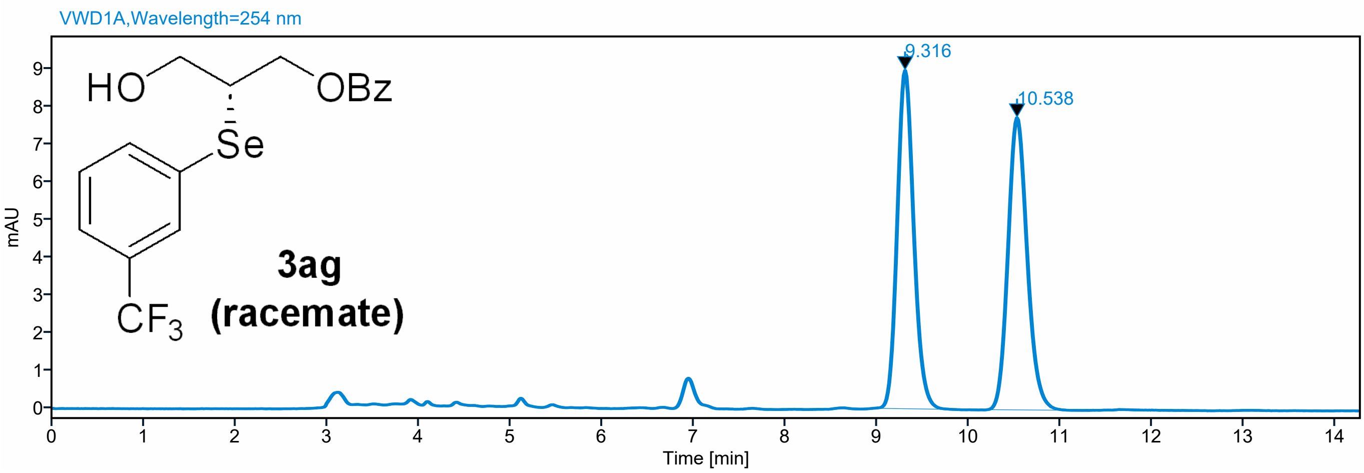

Signal: VWD1A,Wavelength=254 nm

RT [min]	Type	Width [min]	Area	Height	Area%
9.316	BB	0.82	112.75	8.97	49.96
10.538	BB	0.89	112.92	7.75	50.04
		**Sum**	**225.68**		

Single Injection Report

Agilent

Data file: CJX-2-104-ASY-IB-1.0-10%

**Sequence Name:**	SingleSample	**Project Name:**	CJX
**Sample name:**	CJX-2-104-ASY-IB-1.0-10%	**Operator:**	SYSTEM (SYSTEM)
**Instrument:**	1260	**Injection date:**	2025-06-19 11:09:01+08:00
**Inj. volume:**	5.000 µL	**Location:**	P1-C4
**Acq. method:**	10%-60min-1.0ml-5uL.amx	**Type:**	Sample
**Processing method:**	GC_LC area percent_DefaultMethod.pmx	**Sample amount:**	0.00
**Manually modified:**	Manual Integration		

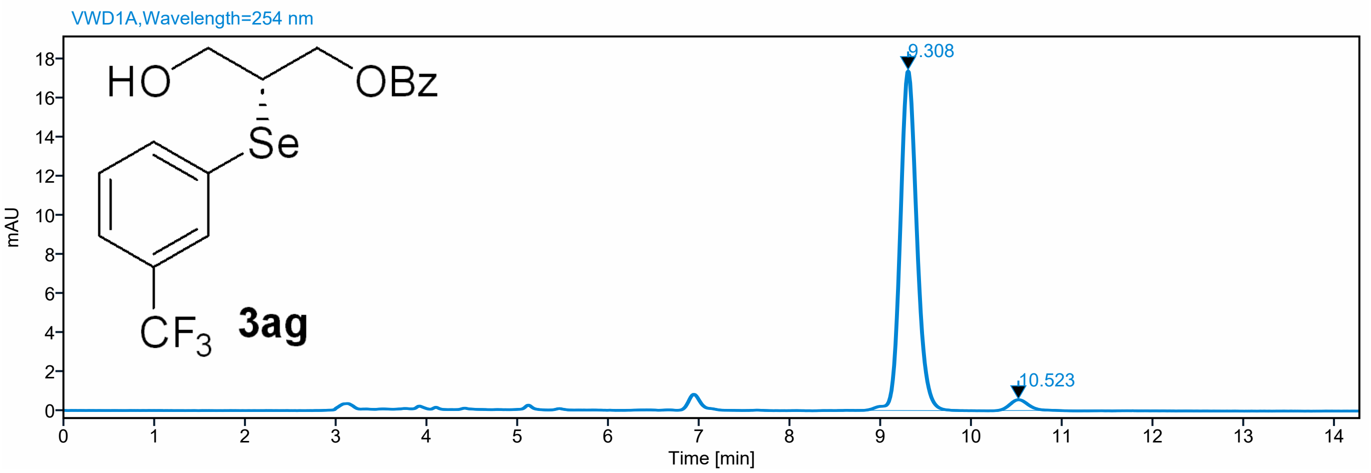

Signal: VWD1A,Wavelength=254 nm

RT [min]	Type	Width [min]	Area	Height	Area%
9.308	BB	1.11	220.80	17.40	96.59
10.523	MM m	0.66	7.79	0.55	3.41
		**Sum**	**228.59**		

Single Injection Report

**Data file:**	CJX-2-149-RAC-IB-1.0-10%		
**Sequence Name:**	SingleSample	**Project Name:**	CJX
**Sample name:**	CJX-2-149-RAC-IB-1.0-10%	**Operator:**	SYSTEM (SYSTEM)
**Instrument:**	1260	**Injection date:**	2025-06-07 19:56:00+08:00
**Inj. volume:**	5.000 µL	**Location:**	P2-D1
**Acq. method:**	10%-60min-1.0ml-5uL.amx	**Type:**	Sample
**Processing method:**	GC_LC area percent_DefaultMethod.pmx	**Sample amount:**	0.00
**Manually modified:**	None		

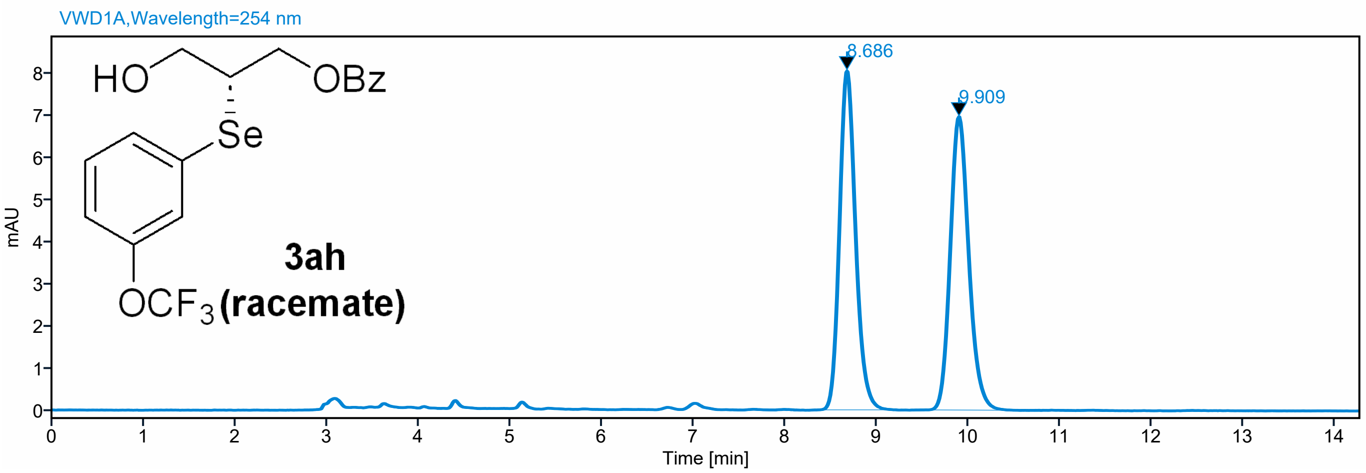

Signal: VWD1A,Wavelength=254 nm

RT [min]	Type	Width [min]	Area	Height	Area%
8.686	BB	0.81	98.17	8.05	50.11
9.909	BB	0.92	97.72	6.96	49.89
		**Sum**	**195.89**		

Single Injection Report

Agilent

**Data file:**	CJX-2-149-ASY-IB-1.0-10%		
**Sequence Name:**	SingleSample	**Project Name:**	CJX
**Sample name:**	CJX-2-149-ASY-IB-1.0-10%	**Operator:**	SYSTEM (SYSTEM)
**Instrument:**	1260	**Injection date:**	2025-06-07 20:12:39+08:00
**Inj. volume:**	5.000 µL	**Location:**	P2-D2
**Acq. method:**	10%-60min-1.0ml-5uL.amx	**Type:**	Sample
**Processing method:**	GC_LC area percent_DefaultMethod.pmx	**Sample amount:**	0.00
**Manually modified:**	Manual Integration		

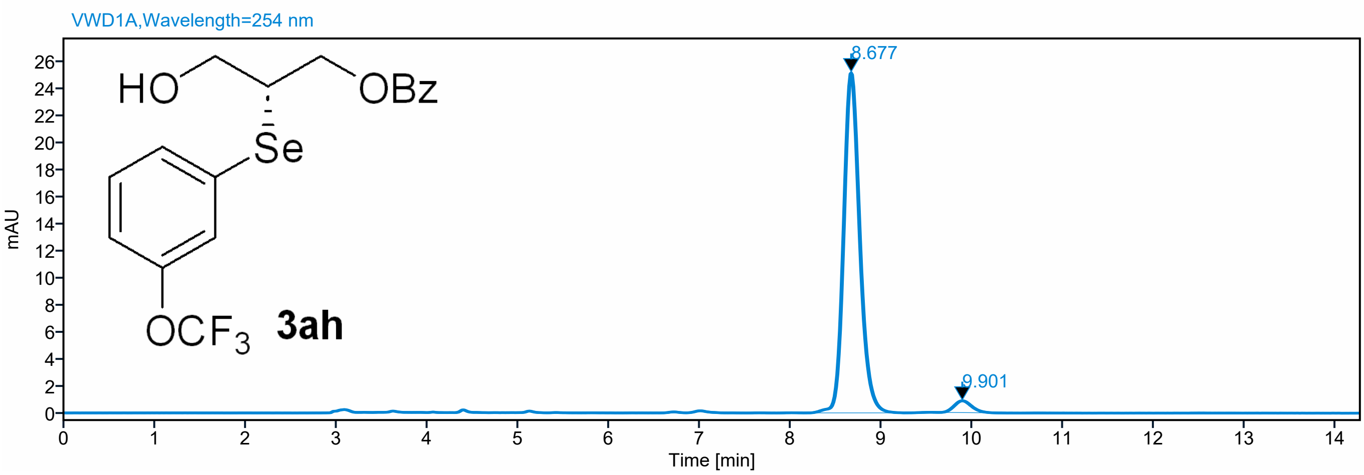

Signal: VWD1A,Wavelength=254 nm

RT [min]	Type	Width [min]	Area	Height	Area%
8.677	BB	1.04	310.34	25.16	96.36
9.901	MM m	0.61	11.73	0.87	3.64
		**Sum**	**322.07**		

Single Injection Report

**Data file:**	CJX-3-6-RAC-IB-1.0-10%		
**Sequence Name:**	SingleSample	**Project Name:**	CJX
**Sample name:**	CJX-3-6-RAC-IB-1.0-10%	**Operator:**	SYSTEM (SYSTEM)
**Instrument:**	1260	**Injection date:**	2025-06-14 15:37:13+08:00
**Inj. volume:**	5.000 µL	**Location:**	P1-E1
**Acq. method:**	10%-60min-1.0ml-5uL.amx	**Type:**	Sample
**Processing method:**	GC_LC area percent_DefaultMethod.pmx	**Sample amount:**	0.00
**Manually modified:**	None		

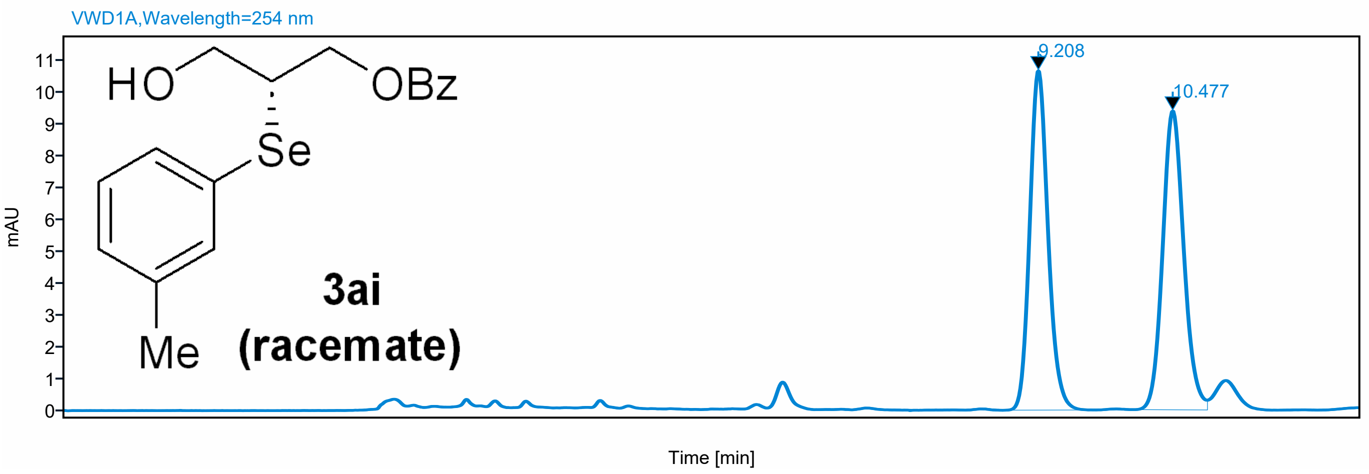

Signal: VWD1A,Wavelength=254 nm

RT [min]	Type	Width [min]	Area	Height	Area%
9.208	BB	0.77	127.92	10.66	49.55
10.477	BV	0.68	130.23	9.39	50.45
		**Sum**	**258.15**		

Single Injection Report

**Data file:**	CJX-3-6-ASY-IB-1.0-10%		
**Sequence Name:**	SingleSample	**Project Name:**	CJX
**Sample name:**	CJX-3-6-ASY-IB-1.0-10%	**Operator:**	SYSTEM (SYSTEM)
**Instrument:**	1260	**Injection date:**	2025-06-14 15:53:56+08:00
**Inj. volume:**	5.000 µL	**Location:**	P1-E2
**Acq. method:**	10%-60min-1.0ml-5uL.amx	**Type:**	Sample
**Processing method:**	GC_LC area percent_DefaultMethod.pmx	**Sample amount:**	0.00
**Manually modified:**	Manual Integration		

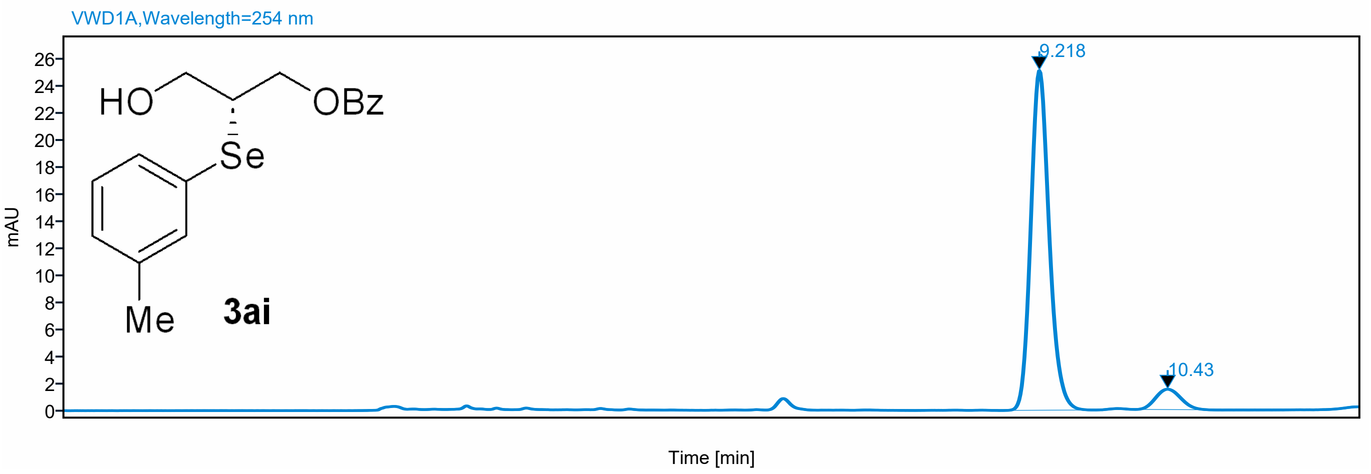

Signal: VWD1A,Wavelength=254 nm

RT [min]	Type	Width [min]	Area	Height	Area%
9.218	BB	0.80	303.81	25.10	92.46
10.430	MM m	0.73	24.78	1.50	7.54
		**Sum**	**328.59**		

Single Injection Report

**Data file:**	CJX-2-64-RAC-IB-1.0-10%		
**Sequence Name:**	SingleSample	**Project Name:**	CJX
**Sample name:**	CJX-2-64-RAC-IB-1.0-10%	**Operator:**	SYSTEM (SYSTEM)
**Instrument:**	1260	**Injection date:**	2025-04-19 20:01:33+08:00
**Inj. volume:**	5.000 µL	**Location:**	P2-A1
**Acq. method:**	10%-60min-1.0ml-5uL.amx	**Type:**	Sample
**Processing method:**	GC_LC area percent_DefaultMethod.pmx	**Sample amount:**	0.00
**Manually modified:**	None		

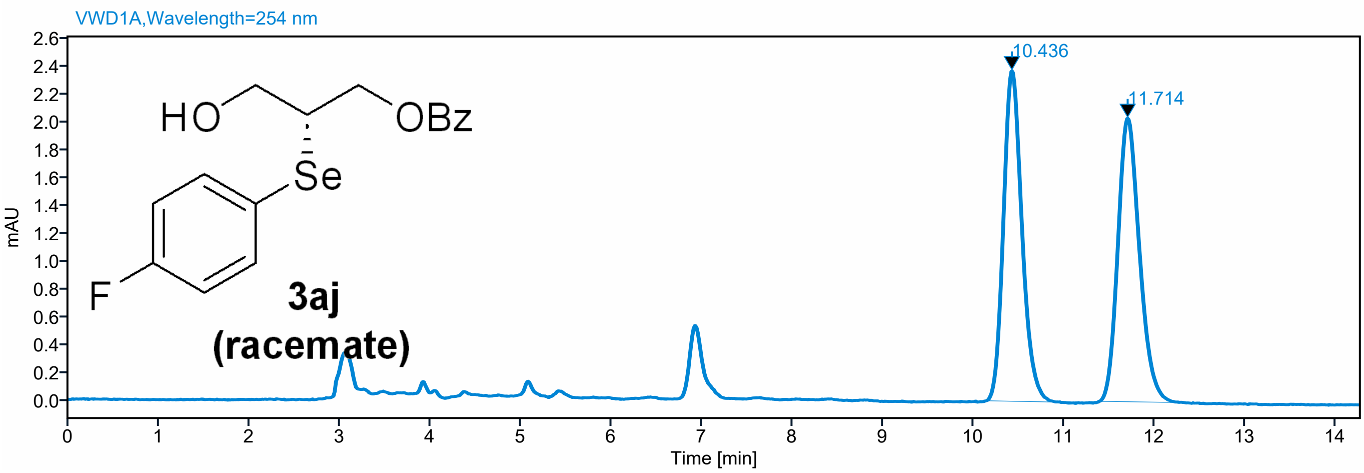

Signal: VWD1A,Wavelength=254 nm

RT [min]	Type	Width [min]	Area	Height	Area%
10.436	BB	0.76	33.74	2.37	49.95
11.714	BB	0.87	33.81	2.04	50.05
		**Sum**	**67.56**		

Single Injection Report

**Data file:**	CJX-2-64-ASY-IB-1.0-10%		
**Sequence Name:**	SingleSample	**Project Name:**	CJX
**Sample name:**	CJX-2-64-ASY-IB-1.0-10%	**Operator:**	SYSTEM (SYSTEM)
**Instrument:**	1260	**Injection date:**	2025-04-19 16:46:24+08:00
**Inj. volume:**	5.000 µL	**Location:**	P2-A2
**Acq. method:**	10%-60min-1.0ml-5uL.amx	**Type:**	Sample
**Processing method:**	GC_LC area percent_DefaultMethod.pmx	**Sample amount:**	0.00
**Manually modified:**	Manual Integration		

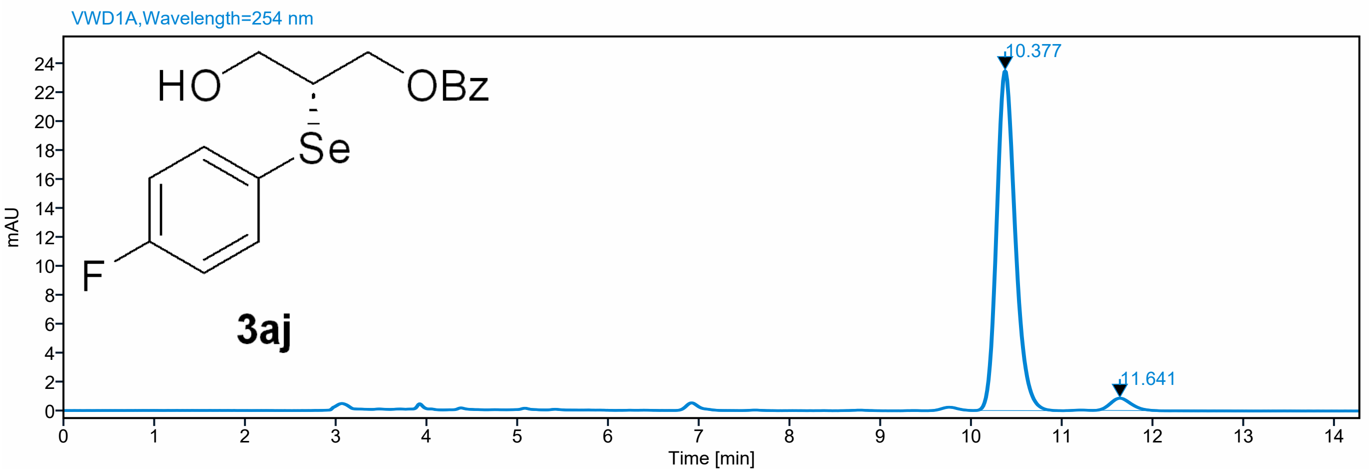

Signal: VWD1A,Wavelength=254 nm

RT [min]	Type	Width [min]	Area	Height	Area%
10.377	BB	0.95	334.56	23.49	96.08
11.641	MM m	0.83	13.66	0.85	3.92
		**Sum**	**348.22**		

Single Injection Report

**Data file:**	CJX-2-53-RAC-IB-1.0-10%		
**Sequence Name:**	SingleSample	**Project Name:**	CJX
**Sample name:**	CJX-2-53-RAC-IB-1.0-10%	**Operator:**	SYSTEM (SYSTEM)
**Instrument:**	1260	**Injection date:**	2025-04-10 17:32:00+08:00
**Inj. volume:**	5.000 µL	**Location:**	P2-E3
**Acq. method:**	10%-60min-1.0ml-5uL.amx	**Type:**	Sample
**Processing method:**	GC_LC area percent_DefaultMethod.pmx	**Sample amount:**	0.00
**Manually modified:**	None		

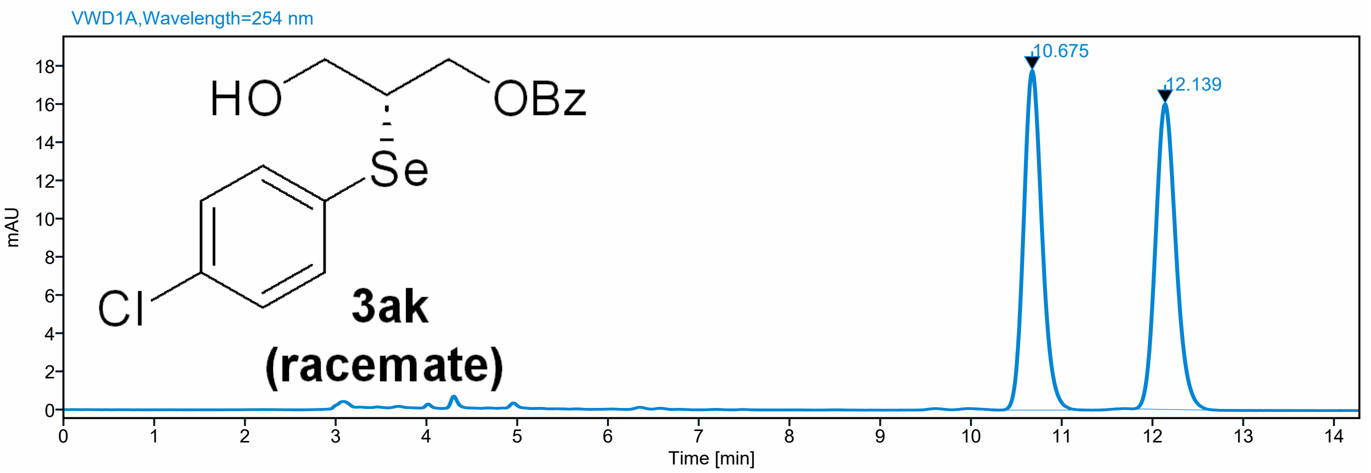

Signal: VWD1A,Wavelength=254 nm

RT [min]	Type	Width [min]	Area	Height	Area%
10.675	BB	0.95	250.27	17.78	50.03
12.139	BB	0.99	249.93	16.00	49.97
		**Sum**	**500.20**		

Single Injection Report

Agilent

**Data file:**	CJX-2-53-ASY-IB-1.0-10%		
**Sequence Name:**	SingleSample	**Project Name:**	CJX
**Sample name:**	CJX-2-53-ASY-IB-1.0-10%	**Operator:**	SYSTEM (SYSTEM)
**Instrument:**	1260	**Injection date:**	2025-04-10 17:52:46+08:00
**Inj. volume:**	5.000 µL	**Location:**	P2-E4
**Acq. method:**	10%-60min-1.0ml-5uL.amx	**Type:**	Sample
**Processing method:**	GC_LC area percent_DefaultMethod.pmx	**Sample amount:**	0.00
**Manually modified:**	Manual Integration		

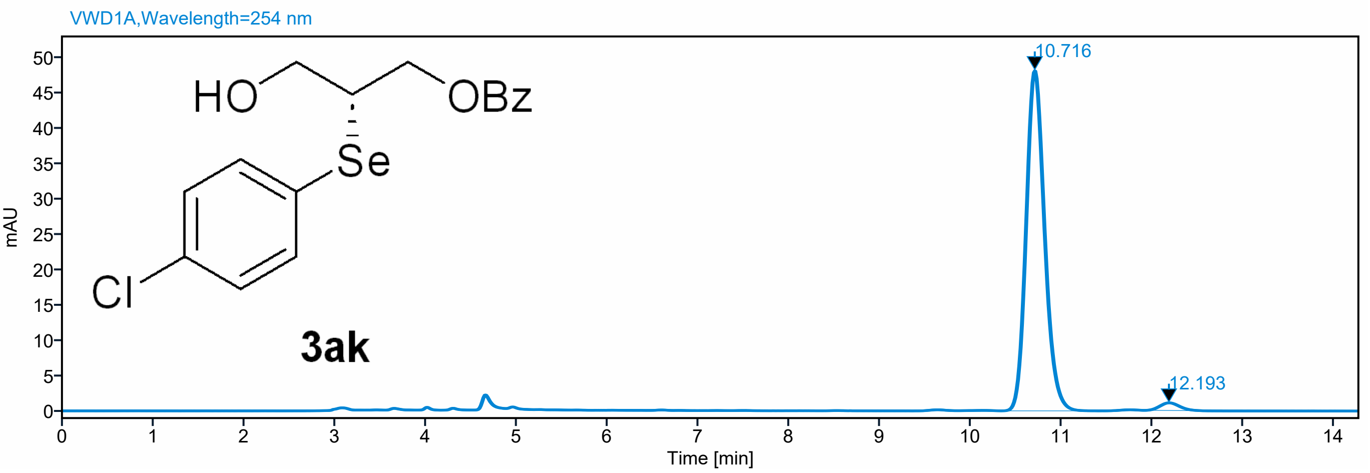

Signal: VWD1A,Wavelength=254 nm

RT [min]	Type	Width [min]	Area	Height	Area%
10.716	BB	1.08	678.95	48.10	97.68
12.193	MM m	0.62	16.15	1.08	2.32
		**Sum**	**695.10**		

Single Injection Report

Agilent

**Data file:**	CJX-2-73-RAC-IB-1.0-10%		
**Sequence Name:**	SingleSample	**Project Name:**	CJX
**Sample name:**	CJX-2-73-RAC-IB-1.0-10%	**Operator:**	SYSTEM (SYSTEM)
**Instrument:**	1260	**Injection date:**	2025-04-27 10:12:30+08:00
**Inj. volume:**	5.000 µL	**Location:**	P2-C1
**Acq. method:**	10%-60min-1.0ml-5uL.amx	**Type:**	Sample
**Processing method:**	GC_LC area percent_DefaultMethod.pmx	**Sample amount:**	0.00
**Manually modified:**	None		

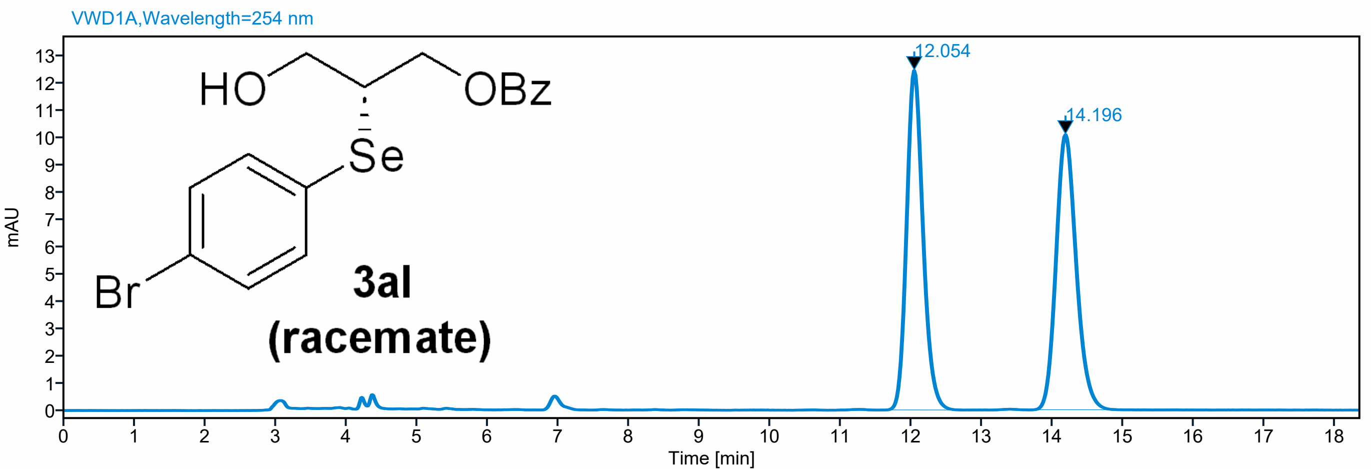

Signal: VWD1A,Wavelength=254 nm

RT [min]	Type	Width [min]	Area	Height	Area%
12.054	BB	1.02	200.80	12.44	50.03
14.196	BB	1.17	200.56	10.08	49.97
		**Sum**	**401.36**		

Single Injection Report

**Data file:**	CJX-2-73-ASY-IB-1.0-10%		
**Sequence Name:**	SingleSample	**Project Name:**	CJX
**Sample name:**	CJX-2-73-ASY-IB-1.0-10%	**Operator:**	SYSTEM (SYSTEM)
**Instrument:**	1260	**Injection date:**	2025-04-27 11:20:39+08:00
**Inj. volume:**	5.000 µL	**Location:**	P2-C2
**Acq. method:**	10%-60min-1.0ml-5uL.amx	**Type:**	Sample
**Processing method:**	GC_LC area percent_DefaultMethod.pmx	**Sample amount:**	0.00
**Manually modified:**	None		

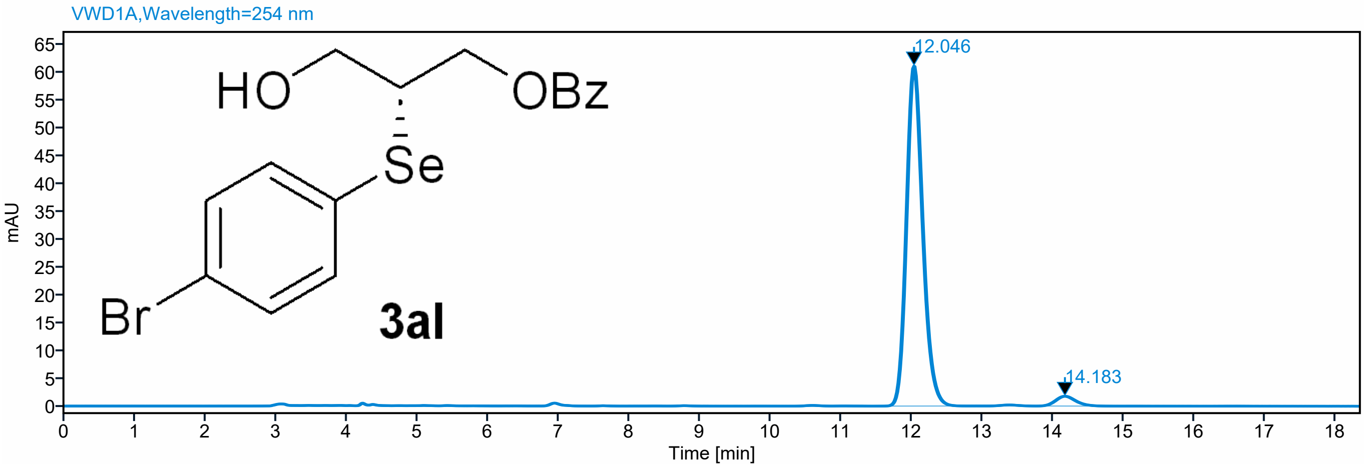

Signal: VWD1A,Wavelength=254 nm

RT [min]	Type	Width [min]	Area	Height	Area%
12.046	BB	1.18	981.40	61.06	96.56
14.183	BB	0.95	34.92	1.76	3.44
		**Sum**	**1016.32**		

Single Injection Report

**Data file:**	CJX-2-88-RAC-IB-1.0-10%		
**Sequence Name:**	SingleSample	**Project Name:**	CJX
**Sample name:**	CJX-2-88-RAC-IB-1.0-10%	**Operator:**	SYSTEM (SYSTEM)
**Instrument:**	1260	**Injection date:**	2025-05-09 21:17:43+08:00
**Inj. volume:**	5.000 µL	**Location:**	P2-E1
**Acq. method:**	10%-60min-1.0ml-5uL.amx	**Type:**	Sample
**Processing method:**	GC_LC area percent_DefaultMethod.pmx	**Sample amount:**	0.00
**Manually modified:**	None		

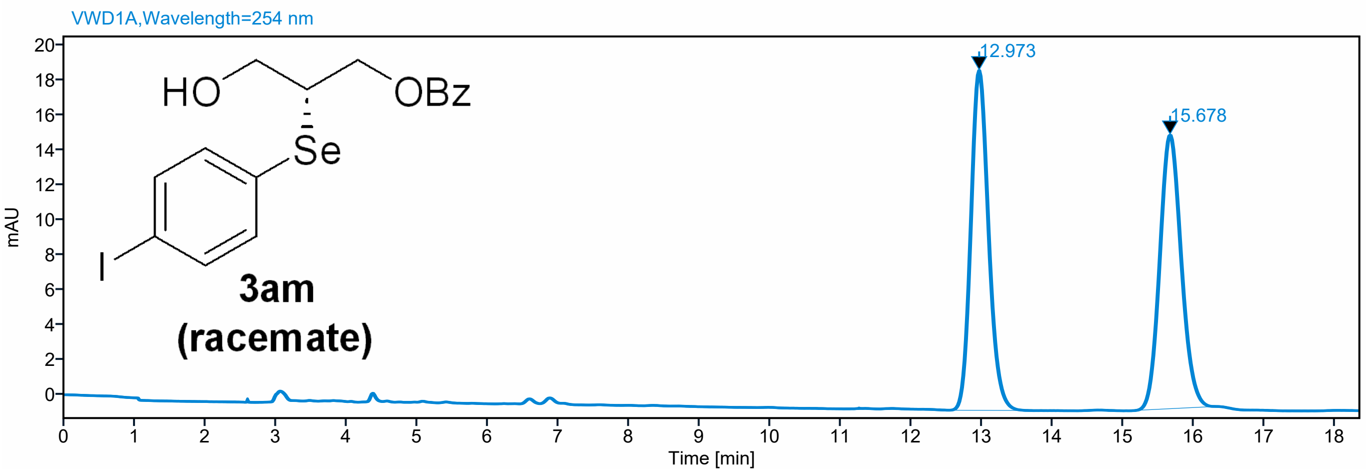

Signal: VWD1A,Wavelength=254 nm

RT [min]	Type	Width [min]	Area	Height	Area%
12.973	BB	1.10	338.90	19.45	50.53
15.678	BB	1.11	331.77	15.67	49.47
		**Sum**	**670.66**		

Single Injection Report

**Data file:**	CJX-2-88-ASY-IB-1.0-10%		
**Sequence Name:**	SingleSample	**Project Name:**	CJX
**Sample name:**	CJX-2-88-ASY-IB-1.0-10%	**Operator:**	SYSTEM (SYSTEM)
**Instrument:**	1260	**Injection date:**	2025-05-09 21:43:45+08:00
**Inj. volume:**	5.000 µL	**Location:**	P2-E2
**Acq. method:**	10%-60min-1.0ml-5uL.amx	**Type:**	Sample
**Processing method:**	GC_LC area percent_DefaultMethod.pmx	**Sample amount:**	0.00
**Manually modified:**	Manual Integration		

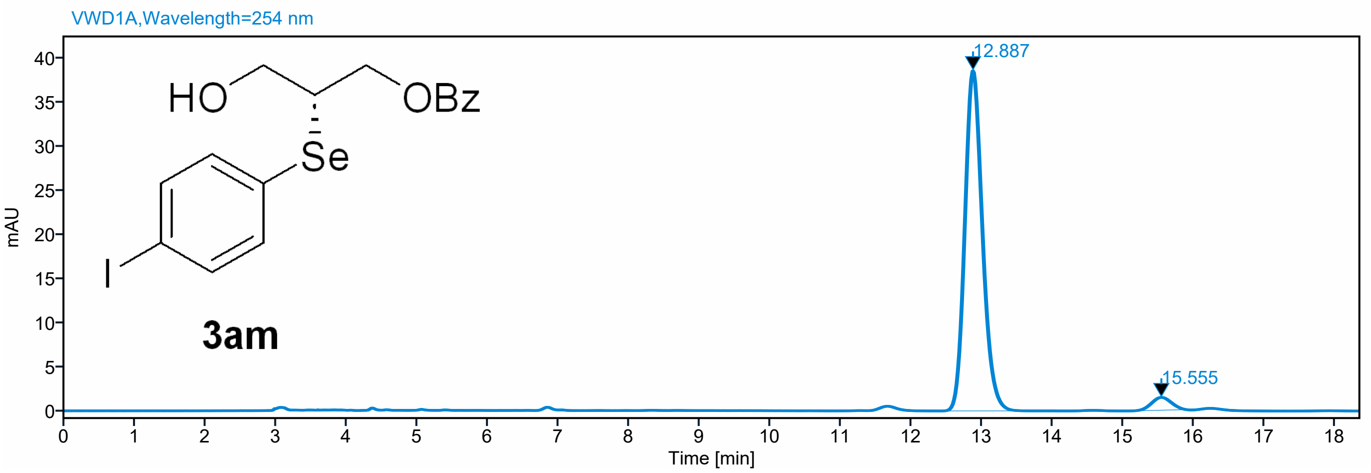

Signal: VWD1A,Wavelength=254 nm

RT [min]	Type	Width [min]	Area	Height	Area%
12.887	BB	1.17	668.40	38.57	95.98
15.555	MM m	0.97	27.99	1.45	4.02
		**Sum**	**696.39**		

Single Injection Report

**Data file:**	CJX-2-103-RAC-IB-1.0-10%		
**Sequence Name:**	SingleSample	**Project Name:**	CJX
**Sample name:**	CJX-2-103-RAC-IB-1.0-10%	**Operator:**	SYSTEM (SYSTEM)
**Instrument:**	1260	**Injection date:**	2025-06-20 15:52:20+08:00
**Inj. volume:**	5.000 µL	**Location:**	P1-C1
**Acq. method:**	10%-60min-1.0ml-5uL.amx	**Type:**	Sample
**Processing method:**	GC_LC area percent_DefaultMethod.pmx	**Sample amount:**	0.00
**Manually modified:**	Manual Integration		

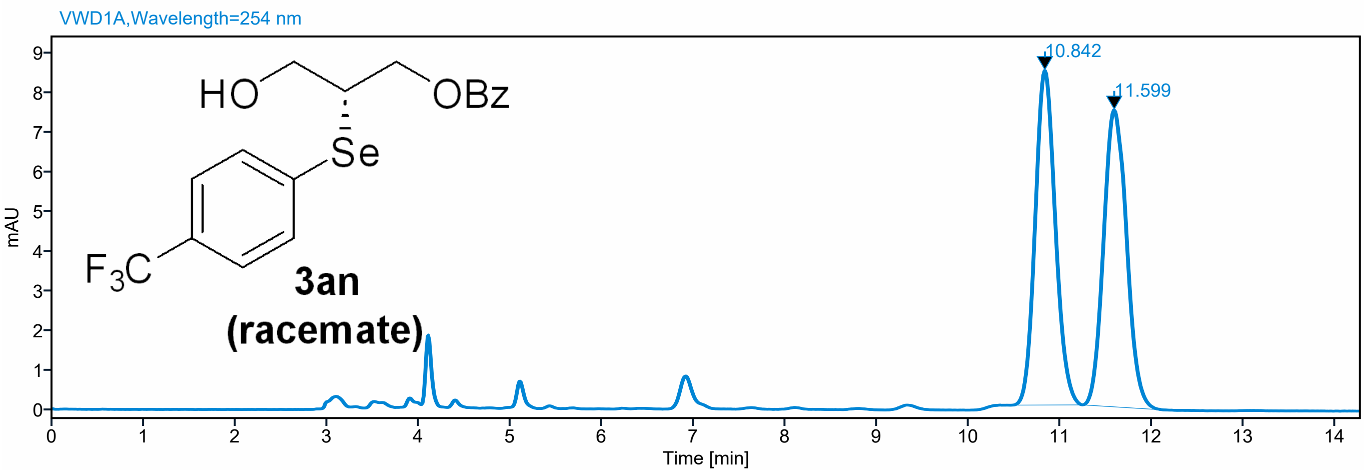

Signal: VWD1A,Wavelength=254 nm

RT [min]	Type	Width [min]	Area	Height	Area%
10.842	BB	0.75	129.12	8.44	49.65
11.599	BB	0.88	130.93	7.49	50.35
		**Sum**	**260.05**		

Single Injection Report

**Data file:**	CJX-2-103-ASY-IB-1.0-10%		
**Sequence Name:**	SingleSample	**Project Name:**	CJX
**Sample name:**	CJX-2-103-ASY-IB-1.0-10%	**Operator:**	SYSTEM (SYSTEM)
**Instrument:**	1260	**Injection date:**	2025-06-19 20:05:53+08:00
**Inj. volume:**	5.000 µL	**Location:**	P1-C2
**Acq. method:**	10%-60min-1.0ml-5uL.amx	**Type:**	Sample
**Processing method:**	GC_LC area percent_DefaultMethod.pmx	**Sample amount:**	0.00
**Manually modified:**	Manual Integration		

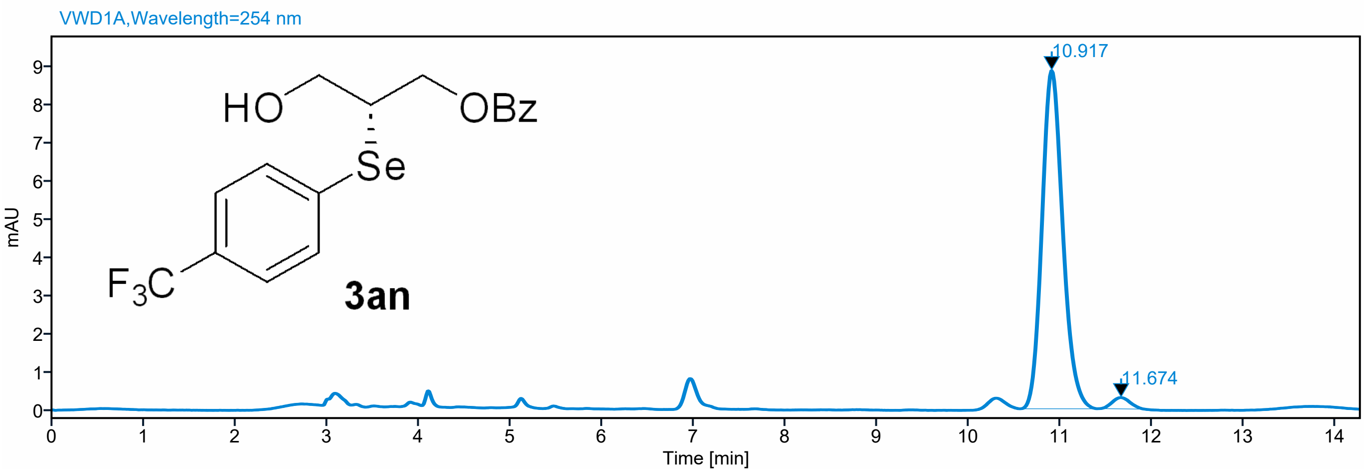

Signal: VWD1A,Wavelength=254 nm

RT [min]	Type	Width [min]	Area	Height	Area%
10.917	BB	0.84	139.62	8.85	97.21
11.674	MM m	0.50	4.01	0.29	2.79
		**Sum**	**143.63**		

Single Injection Report

Agilent

**Data file:**	CJX-2-143-RAC-IB-1.0-10%		
**Sequence Name:**	SingleSample	**Project Name:**	CJX
**Sample name:**	CJX-2-143-RAC-IB-1.0-10%	**Operator:**	SYSTEM (SYSTEM)
**Instrument:**	1260	**Injection date:**	2025-06-05 16:16:15+08:00
**Inj. volume:**	5.000 µL	**Location:**	P2-D1
**Acq. method:**	10%-30min-1.0ml-5uL.amx	**Type:**	Sample
**Processing method:**	GC_LC area percent_DefaultMethod.pmx	**Sample amount:**	0.00
**Manually modified:**	None		

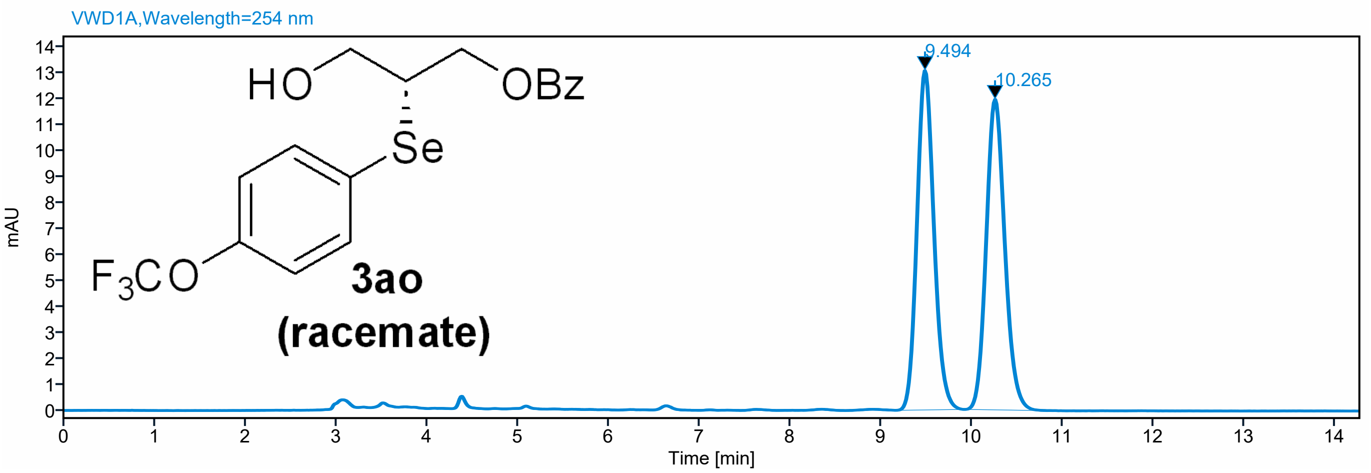

Signal: VWD1A,Wavelength=254 nm

RT [min]	Type	Width [min]	Area	Height	Area%
9.494	BB	0.75	170.22	13.06	50.02
10.265	BB	0.90	170.08	11.94	49.98
		**Sum**	**340.29**		

Single Injection Report

Agilent

**Data file:**	CJX-2-143-ASY-IB-1.0-10%		
**Sequence Name:**	SingleSample	**Project Name:**	CJX
**Sample name:**	CJX-2-143-ASY-IB-1.0-10%	**Operator:**	SYSTEM (SYSTEM)
**Instrument:**	1260	**Injection date:**	2025-06-05 16:32:55+08:00
**Inj. volume:**	5.000 µL	**Location:**	P2-D2
**Acq. method:**	10%-30min-1.0ml-5uL.amx	**Type:**	Sample
**Processing method:**	GC_LC area percent_DefaultMethod.pmx	**Sample amount:**	0.00
**Manually modified:**	None		

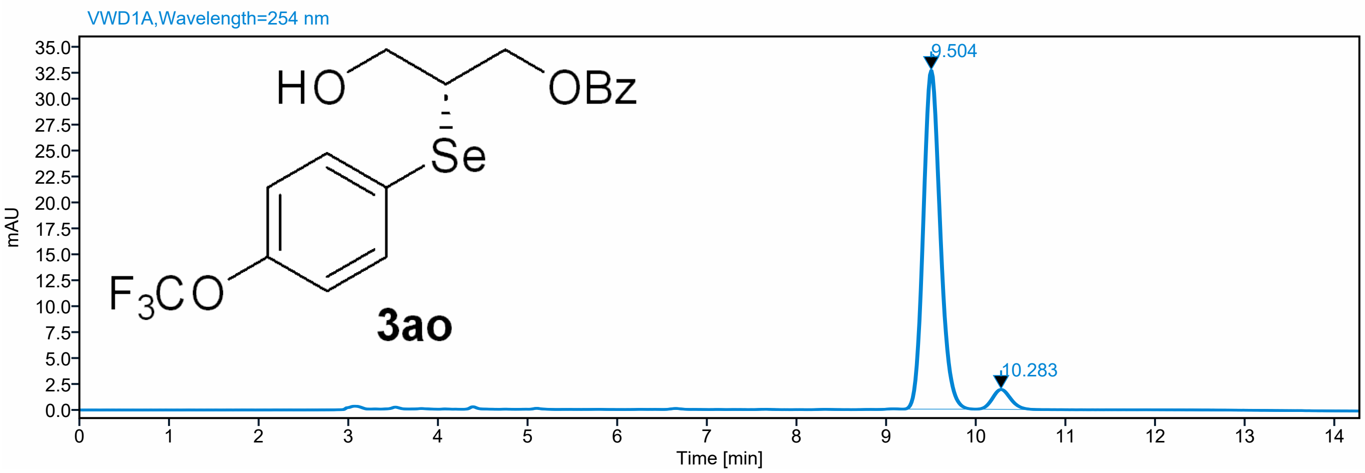

Signal: VWD1A,Wavelength=254 nm

RT [min]	Type	Width [min]	Area	Height	Area%
9.504	BB	0.80	430.10	32.64	94.10
10.283	BB	0.75	26.96	1.91	5.90
		**Sum**	**457.07**		

Single Injection Report

Agilent

**Data file:**	CJX-3-19-RAC-IB-1.0-10%		
**Sequence Name:**	SingleSample	**Project Name:**	CJX
**Sample name:**	CJX-3-19-RAC-IB-1.0-10%	**Operator:**	SYSTEM (SYSTEM)
**Instrument:**	1260	**Injection date:**	2025-06-27 22:47:41+08:00
**Inj. volume:**	5.000 µL	**Location:**	P2-C1
**Acq. method:**	20%-50min-1.0ml-5uL.amx	**Type:**	Sample
**Processing method:**	GC_LC area percent_DefaultMethod.pmx	**Sample amount:**	0.00
**Manually modified:**	None		

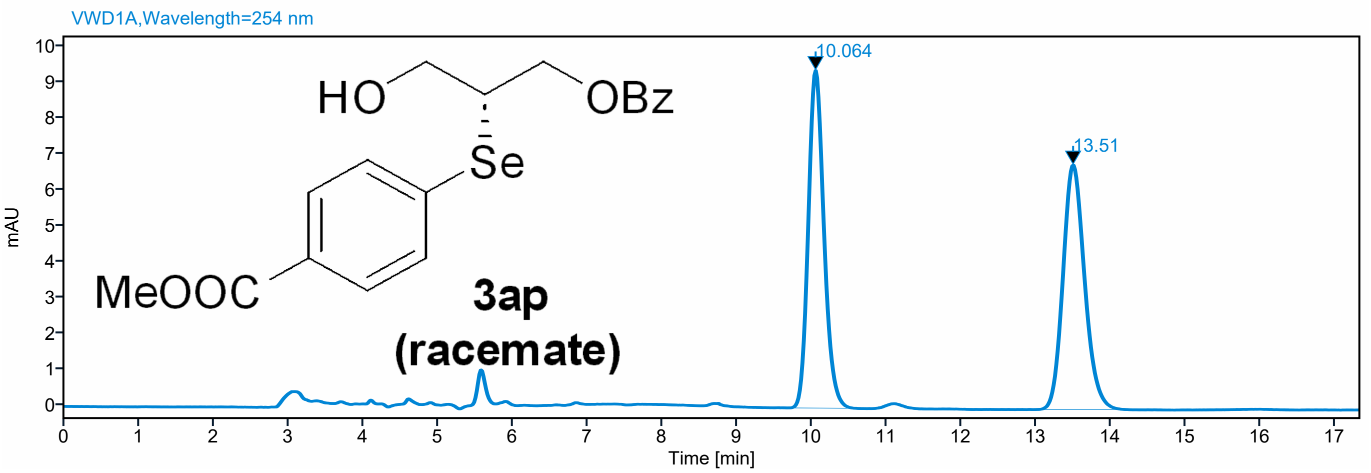

**Signal:**	VWD1A,Wavelength=254 nm				
**RT [min]**	**Type**	**Width [min]**	**Area**	**Height**	**Area%**
10.064	BB	0.85	137.06	9.41	50.00
13.510	BB	1.13	137.06	6.81	50.00
		**Sum**	**274.12**		

Single Injection Report

**Data file:**	CJX-3-19-ASY-IB-1.0-10%		
**Sequence Name:**	SingleSample	**Project Name:**	CJX
**Sample name:**	CJX-3-19-ASY-IB-1.0-10%	**Operator:**	SYSTEM (SYSTEM)
**Instrument:**	1260	**Injection date:**	2025-06-27 22:26:59+08:00
**Inj. volume:**	5.000 µL	**Location:**	P2-C2
**Acq. method:**	20%-50min-1.0ml-5uL.amx	**Type:**	Sample
**Processing method:**	GC_LC area percent_DefaultMethod.pmx	**Sample amount:**	0.00
**Manually modified:**	Manual Integration		

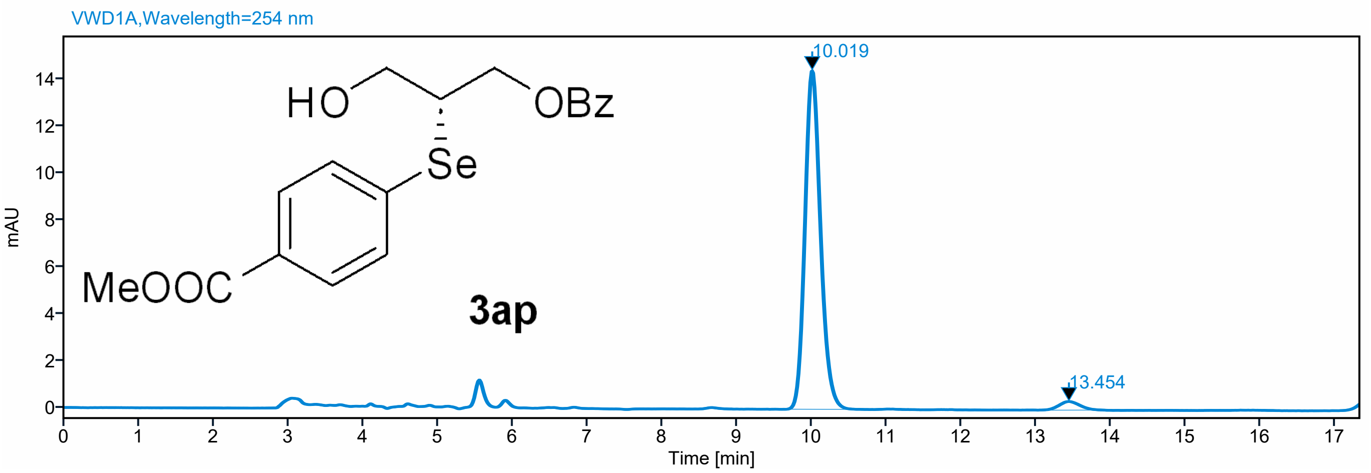

Signal: VWD1A,Wavelength=254 nm

RT [min]	Type	Width [min]	Area	Height	Area%
10.019	BB	0.95	209.20	14.43	96.67
13.454	MM m	0.88	7.20	0.37	3.33
		**Sum**	**216.40**		

Single Injection Report

**Data file:**	CJX-2-60-RAC-IB-1.0-10%		
**Sequence Name:**	SingleSample	**Project Name:**	CJX
**Sample name:**	CJX-2-60-RAC-IB-1.0-10%	**Operator:**	SYSTEM (SYSTEM)
**Instrument:**	1260	**Injection date:**	2025-04-17 16:29:18+08:00
**Inj. volume:**	5.000 µL	**Location:**	P2-A1
**Acq. method:**	10%-60min-1.0ml-5uL.amx	**Type:**	Sample
**Processing method:**	GC_LC area percent_DefaultMethod.pmx	**Sample amount:**	0.00
**Manually modified:**	None		

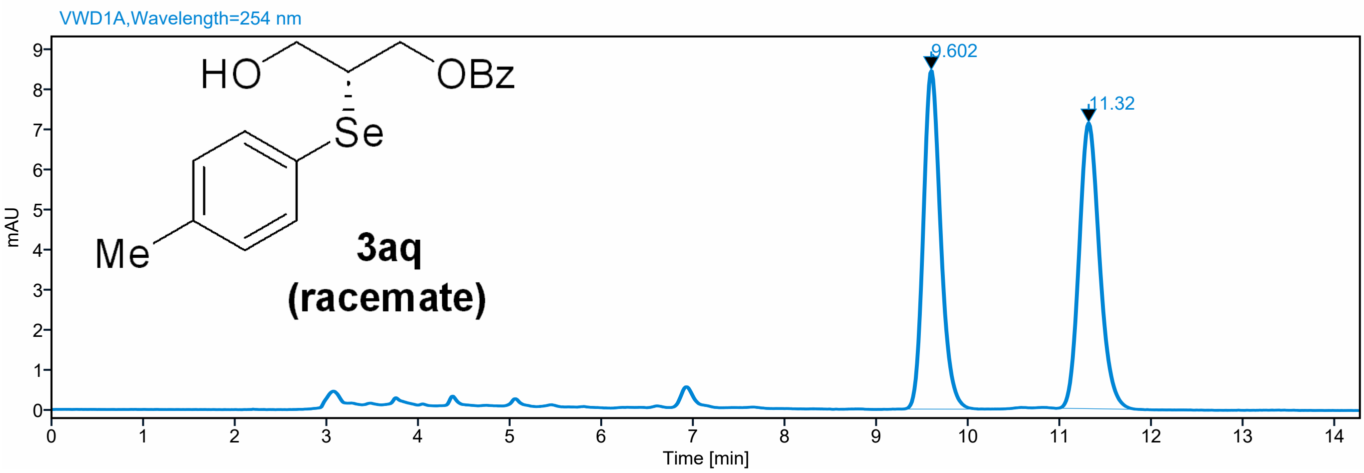

Signal: VWD1A,Wavelength=254 nm

RT [min]	Type	Width [min]	Area	Height	Area%
9.602	BB	0.82	111.04	8.45	50.12
11.320	BB	0.87	110.52	7.13	49.88
		**Sum**	**221.56**		

Single Injection Report

Agilent

**Data file:**	CJX-2-60-ASY-IB-1.0-10%		
**Sequence Name:**	SingleSample	**Project Name:**	CJX
**Sample name:**	CJX-2-60-ASY-IB-1.0-10%	**Operator:**	SYSTEM (SYSTEM)
**Instrument:**	1260	**Injection date:**	2025-04-17 16:10:41+08:00
**Inj. volume:**	5.000 µL	**Location:**	P2-A2
**Acq. method:**	10%-60min-1.0ml-5uL.amx	**Type:**	Sample
**Processing method:**	GC_LC area percent_DefaultMethod.pmx	**Sample amount:**	0.00
**Manually modified:**	Manual Integration		

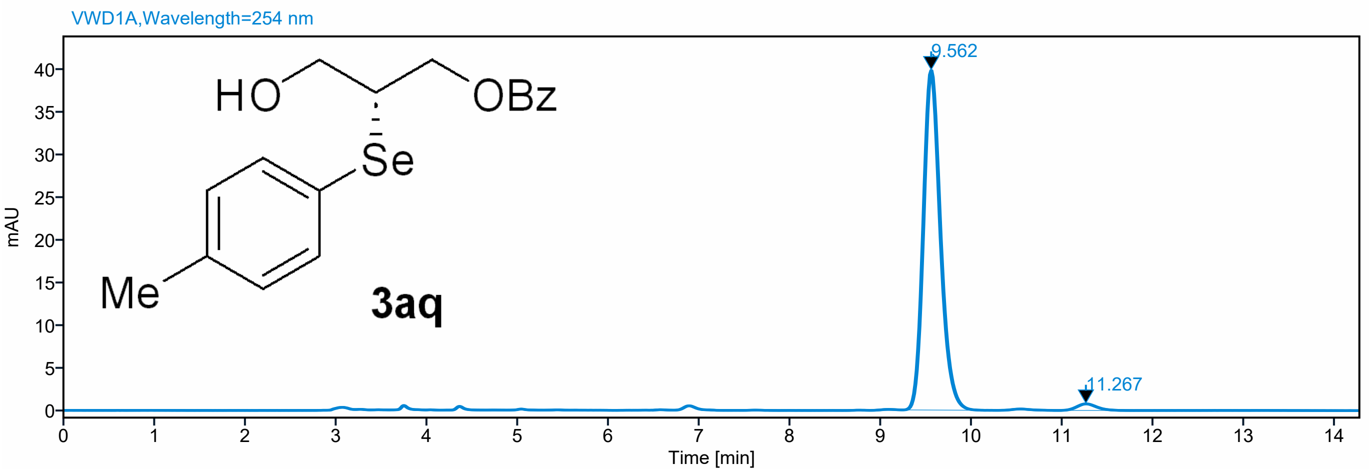

Signal: VWD1A,Wavelength=254 nm

RT [min]	Type	Width [min]	Area	Height	Area%
9.562	BB	0.90	521.29	39.79	97.81
11.267	MM m	0.94	11.69	0.77	2.19
		**Sum**	**532.98**		

Single Injection Report

**Data file:**	CJX-2-63-RAC-IB-1.0-10%		
**Sequence Name:**	SingleSample	**Project Name:**	CJX
**Sample name:**	CJX-2-63-RAC-IB-1.0-10%	**Operator:**	SYSTEM (SYSTEM)
**Instrument:**	1260	**Injection date:**	2025-04-19 17:36:02+08:00
**Inj. volume:**	5.000 µL	**Location:**	P2-A3
**Acq. method:**	10%-60min-1.0ml-5uL.amx	**Type:**	Sample
**Processing method:**	GC_LC area percent_DefaultMethod.pmx	**Sample amount:**	0.00
**Manually modified:**	None		

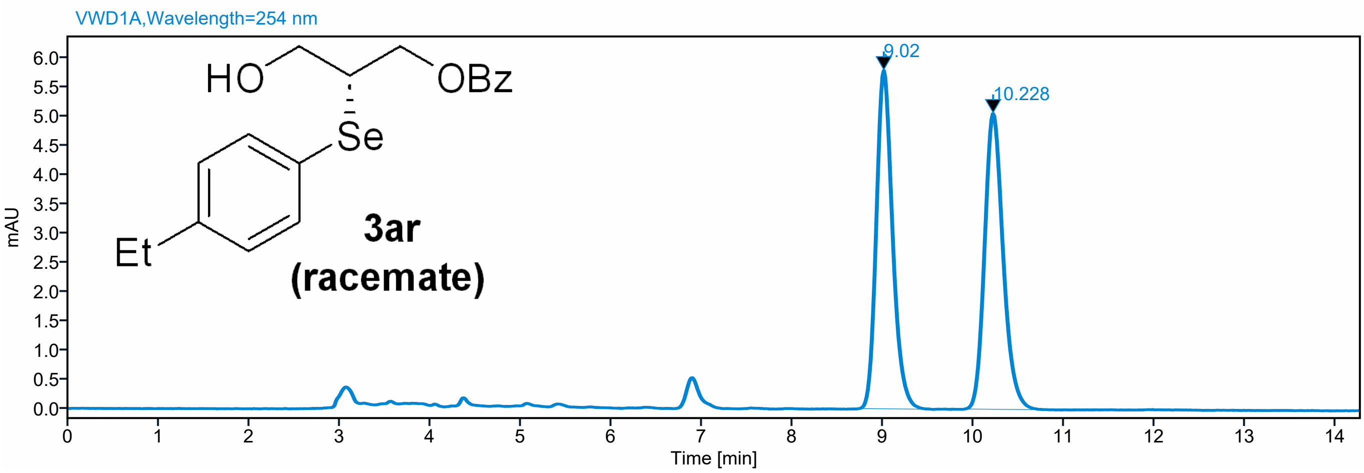

Signal: VWD1A,Wavelength=254 nm

RT [min]	Type	Width [min]	Area	Height	Area%
9.020	BB	0.80	72.31	5.79	50.04
10.228	BB	0.87	72.19	5.06	49.96
		**Sum**	**144.50**		

Single Injection Report

**Data file:**	CJX-2-63-ASY-IB-1.0-10%		
**Sequence Name:**	SingleSample	**Project Name:**	CJX
**Sample name:**	CJX-2-63-ASY-IB-1.0-10%	**Operator:**	SYSTEM (SYSTEM)
**Instrument:**	1260	**Injection date:**	2025-04-19 17:52:41+08:00
**Inj. volume:**	5.000 µL	**Location:**	P2-A4
**Acq. method:**	10%-60min-1.0ml-5uL.amx	**Type:**	Sample
**Processing method:**	GC_LC area percent_DefaultMethod.pmx	**Sample amount:**	0.00
**Manually modified:**	Manual Integration		

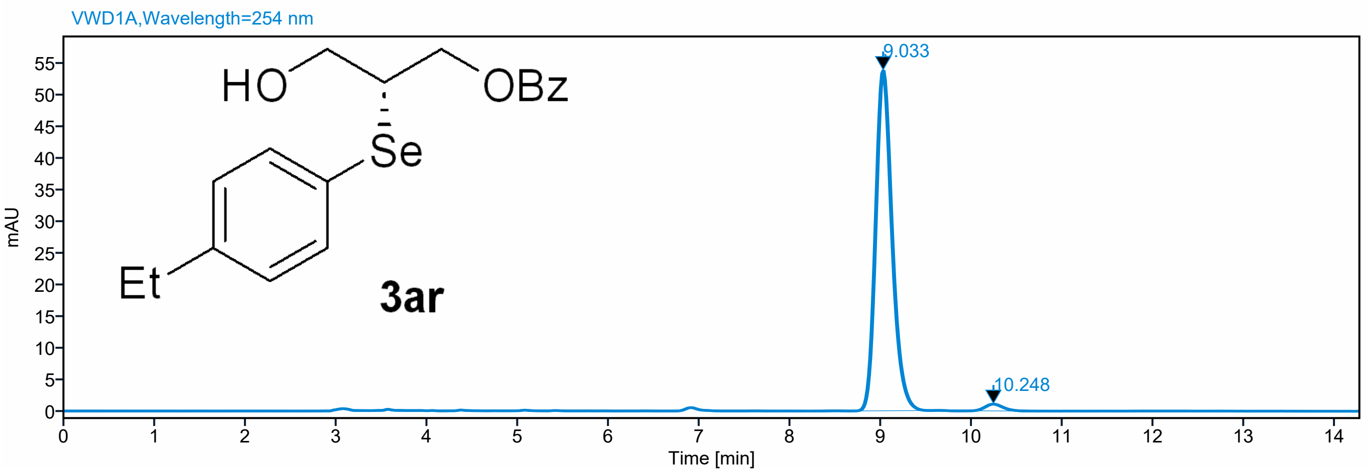

Signal: VWD1A,Wavelength=254 nm

RT [min]	Type	Width [min]	Area	Height	Area%
9.033	BB	0.83	676.48	53.76	97.87
10.248	MM m	0.68	14.74	1.07	2.13
		**Sum**	**691.21**		

Single Injection Report

Agilent

**Data file:**	CJX-2-119-RAC-IB-1.0-10%		
**Sequence Name:**	SingleSample	**Project Name:**	CJX
**Sample name:**	CJX-2-119-RAC-IB-1.0-10%	**Operator:**	SYSTEM (SYSTEM)
**Instrument:**	1260	**Injection date:**	2025-05-24 20:35:08+08:00
**Inj. volume:**	5.000 µL	**Location:**	P2-D3
**Acq. method:**	10%-60min-1.0ml-5uL.amx	**Type:**	Sample
**Processing method:**	GC_LC area percent_DefaultMethod.pmx	**Sample amount:**	0.00
**Manually modified:**	None		

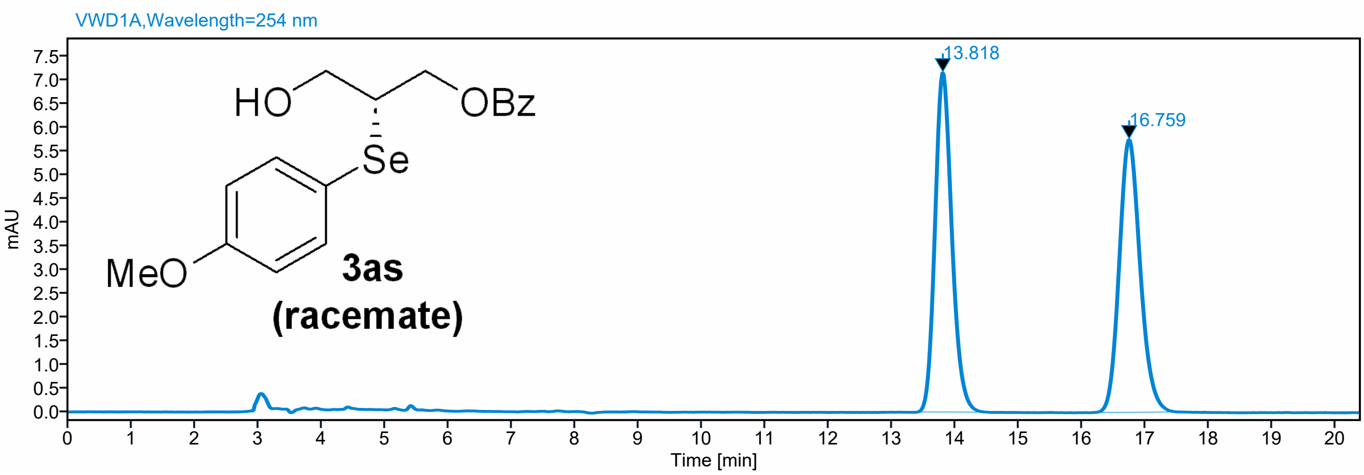

Signal: VWD1A,Wavelength=254 nm

RT [min]	Type	Width [min]	Area	Height	Area%
13.818	BB	1.09	132.61	7.16	50.04
16.759	BB	1.29	132.39	5.75	49.96
		**Sum**	**265.00**		

Single Injection Report

Agilent

**Data file:**	CJX-2-119-ASY-IB-1.0-10%		
**Sequence Name:**	SingleSample	**Project Name:**	CJX
**Sample name:**	CJX-2-119-ASY-IB-1.0-10%	**Operator:**	SYSTEM (SYSTEM)
**Instrument:**	1260	**Injection date:**	2025-05-24 20:57:47+08:00
**Inj. volume:**	5.000 µL	**Location:**	P2-D4
**Acq. method:**	10%-60min-1.0ml-5uL.amx	**Type:**	Sample
**Processing method:**	GC_LC area percent_DefaultMethod.pmx	**Sample amount:**	0.00
**Manually modified:**	Manual Integration		

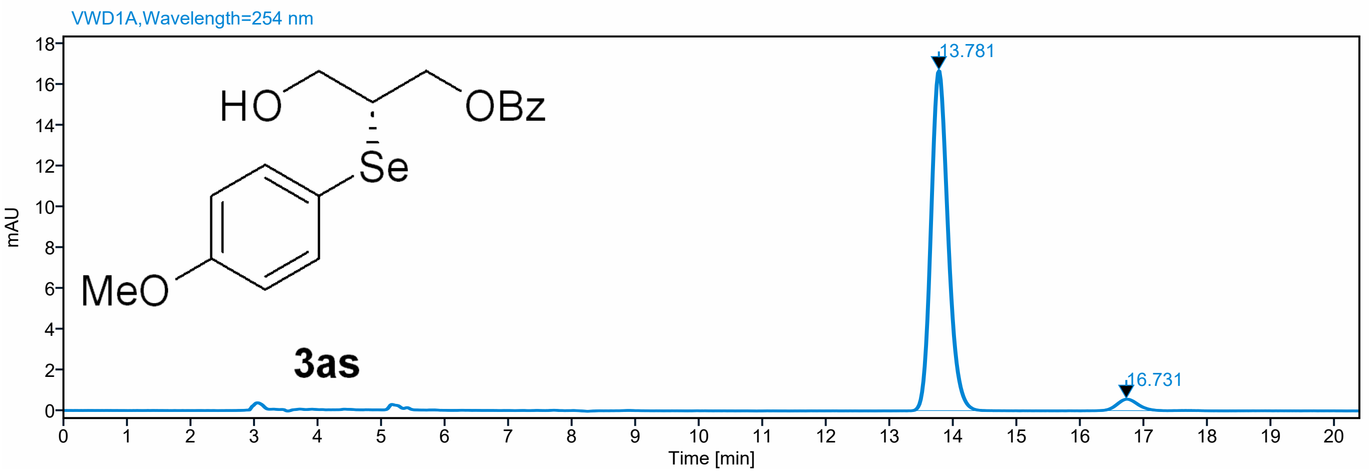

Signal: VWD1A,Wavelength=254 nm

RT [min]	Type	Width [min]	Area	Height	Area%
13.781	BB	1.16	310.74	16.68	96.04
16.731	MM m	1.05	12.82	0.56	3.96
		**Sum**	**323.56**		

**Data file:**	CJX-2-52-RAC-IB-1.0-10%		
**Sequence Name:**	SingleSample	**Project Name:**	CJX
**Sample name:**	CJX-2-52-RAC-IB-1.0-10%	**Operator:**	SYSTEM (SYSTEM)
**Instrument:**	1260	**Injection date:**	2025-04-11 17:20:53+08:00
**Inj. volume:**	5.000 µL	**Location:**	P2-E1
**Acq. method:**	10%-60min-1.0ml-5uL.amx	**Type:**	Sample
**Processing method:**	GC_LC area percent_DefaultMethod.pmx	**Sample amount:**	0.00
**Manually modified:**	None		

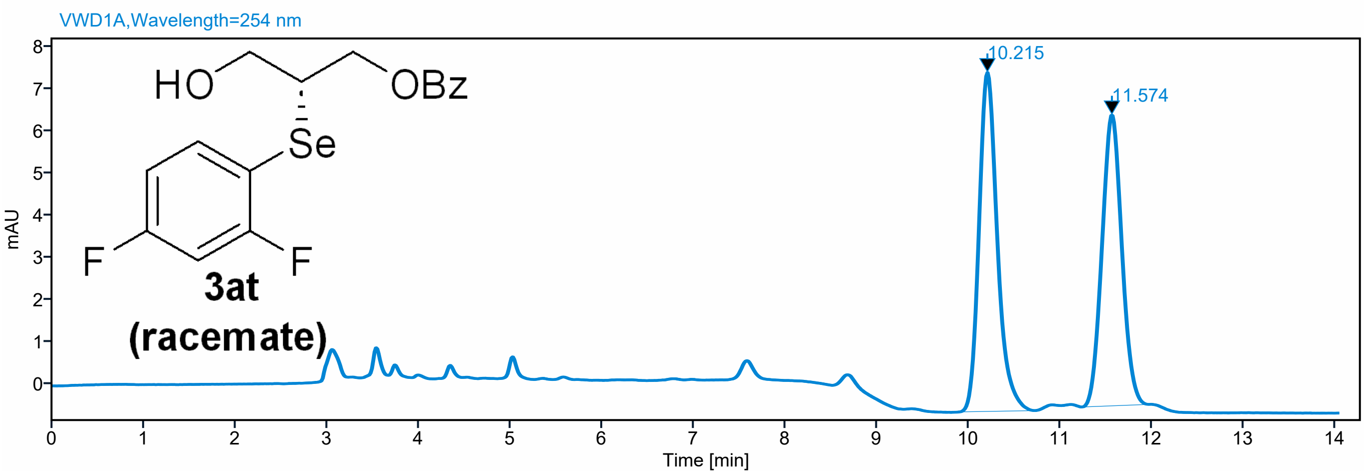

Signal: VWD1A,Wavelength=254 nm

RT [min]	Type	Width [min]	Area	Height	Area%
10.215	BB	0.80	111.78	8.04	51.88
11.574	BB	0.74	103.69	6.90	48.12
		**Sum**	**215.47**		

Single Injection Report

**Data file:**	CJX-2-52-ASY-IB-1.0-10%		
**Sequence Name:**	SingleSample	**Project Name:**	CJX
**Sample name:**	CJX-2-52-ASY-IB-1.0-10%	**Operator:**	SYSTEM (SYSTEM)
**Instrument:**	1260	**Injection date:**	2025-04-10 17:09:50+08:00
**Inj. volume:**	5.000 µL	**Location:**	P2-E2
**Acq. method:**	10%-60min-1.0ml-5uL.amx	**Type:**	Sample
**Processing method:**	GC_LC area percent_DefaultMethod.pmx	**Sample amount:**	0.00
**Manually modified:**	Manual Integration		

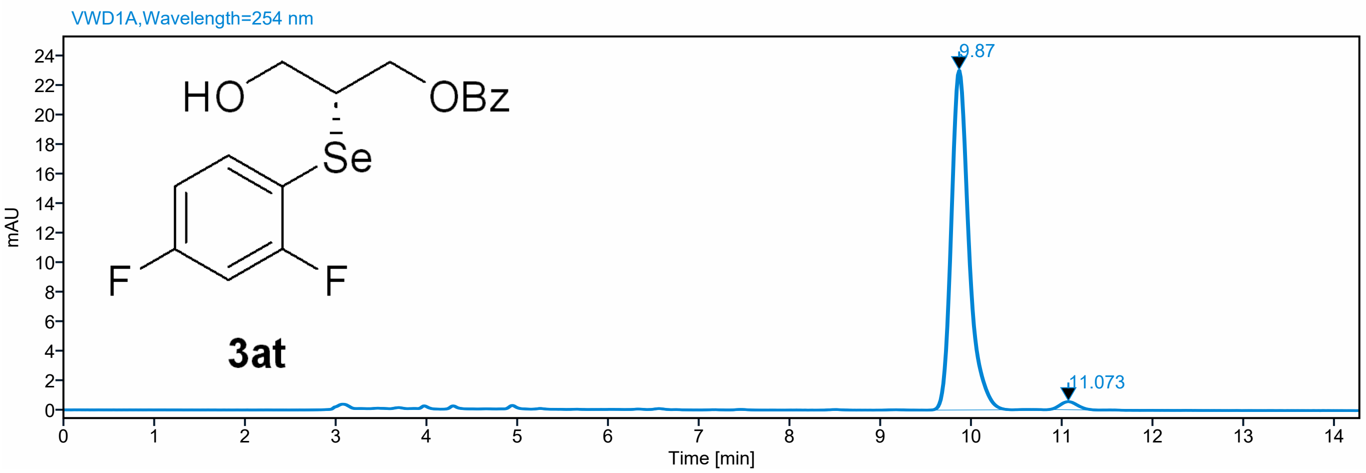

Signal: VWD1A,Wavelength=254 nm

RT [min]	Type	Width [min]	Area	Height	Area%
9.870	BB	0.94	310.21	23.00	97.69
11.073	MM m	0.56	7.33	0.55	2.31
		**Sum**	**317.54**		

Single Injection Report

**Data file:**	CJX-3-33-RAC-IB-1.0-10%		
**Sequence Name:**	SingleSample	**Project Name:**	CJX
**Sample name:**	CJX-3-33-RAC-IB-1.0-10%	**Operator:**	SYSTEM (SYSTEM)
**Instrument:**	1260	**Injection date:**	2025-07-09 21:32:13+08:00
**Inj. volume:**	5.000 µL	**Location:**	P1-C1
**Acq. method:**	10%-60min-1.0ml-5uL.amx	**Type:**	Sample
**Processing method:**	GC_LC area percent_DefaultMethod.pmx	**Sample amount:**	0.00
**Manually modified:**	None		

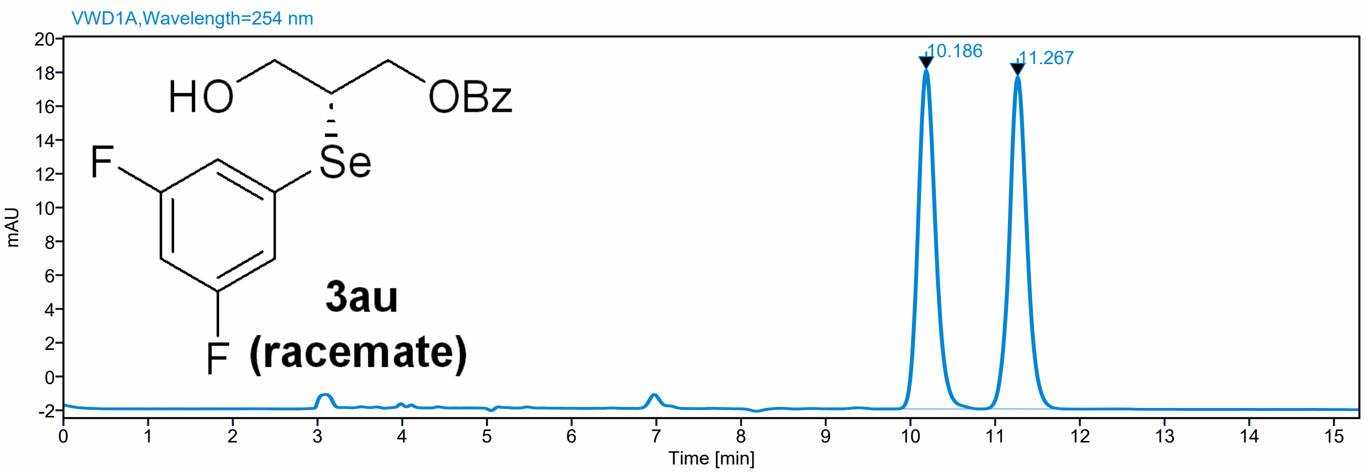

Signal: VWD1A,Wavelength=254 nm

RT [min]	Type	Width [min]	Area	Height	Area%
10.186	BB	0.99	286.83	20.03	50.22
11.267	BB	0.95	284.37	19.63	49.78
		**Sum**	**571.20**		

Single Injection Report

Agilent

**Data file:**	CJX-3-33-ASY-IB-1.0-10%		
**Sequence Name:**	SingleSample	**Project Name:**	CJX
**Sample name:**	CJX-3-33-ASY-IB-1.0-10%	**Operator:**	SYSTEM (SYSTEM)
**Instrument:**	1260	**Injection date:**	2025-07-09 21:48:54+08:00
**Inj. volume:**	5.000 µL	**Location:**	P1-C2
**Acq. method:**	10%-60min-1.0ml-5uL.amx	**Type:**	Sample
**Processing method:**	GC_LC area percent_DefaultMethod.pmx	**Sample amount:**	0.00
**Manually modified:**	Manual Integration		

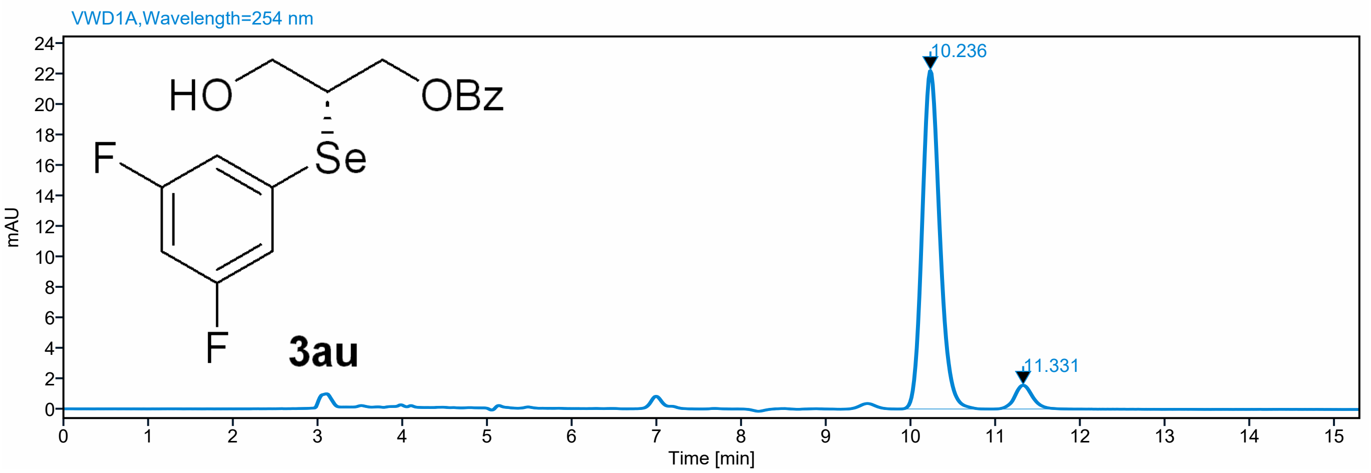

Signal: VWD1A,Wavelength=254 nm

RT [min]	Type	Width [min]	Area	Height	Area%
10.236	BB	0.98	321.12	22.21	93.35
11.331	MM m	0.82	22.86	1.56	6.65
		**Sum**	**343.98**		

Single Injection Report

**Data file:**	CJX-2-135-RAC-IB-1.0-10%		
**Sequence Name:**	SingleSample	**Project Name:**	CJX
**Sample name:**	CJX-2-135-RAC-IB-1.0-10%	**Operator:**	SYSTEM (SYSTEM)
**Instrument:**	1260	**Injection date:**	2025-05-31 22:06:03+08:00
**Inj. volume:**	5.000 µL	**Location:**	P2-D3
**Acq. method:**	10%-30min-1.0ml-5uL.amx	**Type:**	Sample
**Processing method:**	GC_LC area percent_DefaultMethod.pmx	**Sample amount:**	0.00
**Manually modified:**	None		

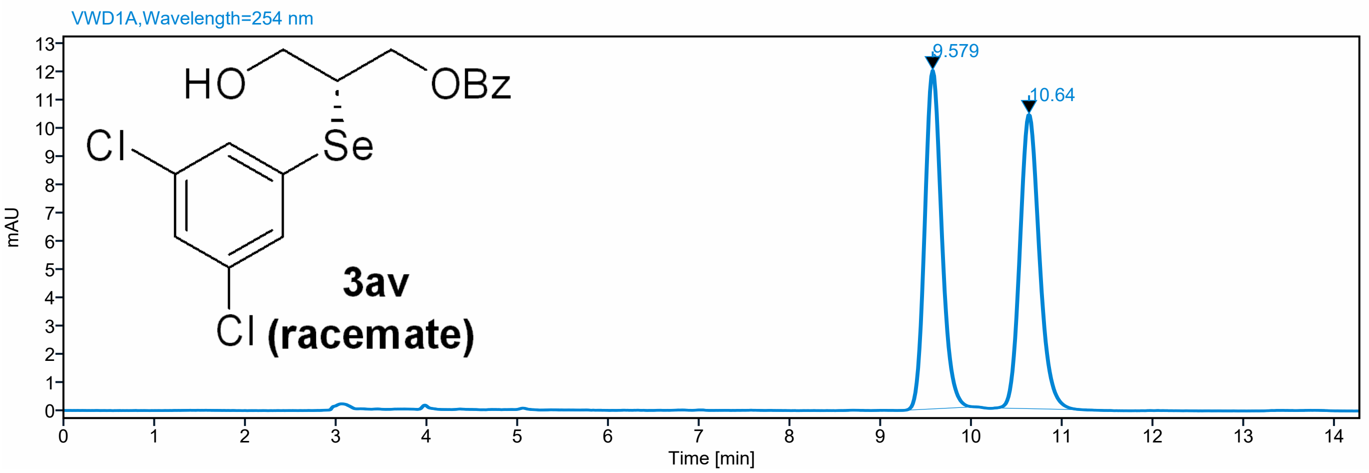

Signal: VWD1A,Wavelength=254 nm

RT [min]	Type	Width [min]	Area	Height	Area%
9.579	BB	0.76	155.73	11.98	50.04
10.640	BB	0.96	155.47	10.42	49.96
		**Sum**	**311.20**		

Single Injection Report

**Data file:**	CJX-2-135-ASY-IB-1.0-10%		
**Sequence Name:**	SingleSample	**Project Name:**	CJX
**Sample name:**	CJX-2-135-ASY-IB-1.0-10%	**Operator:**	SYSTEM (SYSTEM)
**Instrument:**	1260	**Injection date:**	2025-05-31 22:23:41+08:00
**Inj. volume:**	5.000 µL	**Location:**	P2-D4
**Acq. method:**	10%-30min-1.0ml-5uL.amx	**Type:**	Sample
**Processing method:**	GC_LC area percent_DefaultMethod.pmx	**Sample amount:**	0.00
**Manually modified:**	Manual Integration		

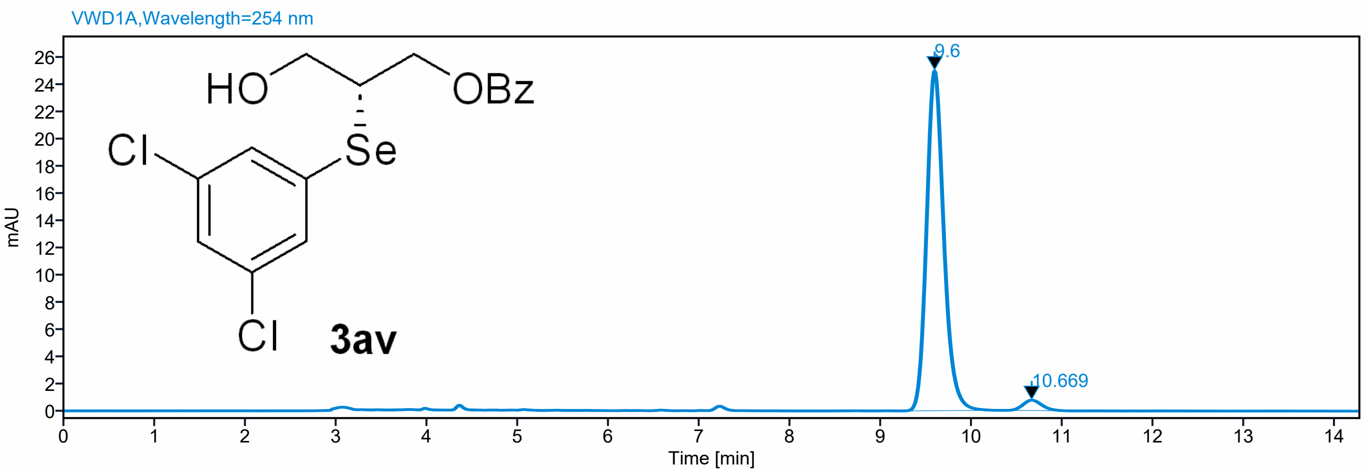

Signal: VWD1A,Wavelength=254 nm

RT [min]	Type	Width [min]	Area	Height	Area%
9.600	BB	1.01	330.47	25.00	96.75
10.669	MM m	0.92	11.11	0.77	3.25
		**Sum**	**341.58**		

Single Injection Report

Agilent

**Data file:**	CJX-2-105-RAC-IB-1.0-10%		
**Sequence Name:**	SingleSample	**Project Name:**	CJX
**Sample name:**	CJX-2-105-RAC-IB-1.0-10%	**Operator:**	SYSTEM (SYSTEM)
**Instrument:**	1260	**Injection date:**	2025-06-19 21:45:22+08:00
**Inj. volume:**	5.000 µL	**Location:**	P1-C5
**Acq. method:**	10%-60min-1.0ml-5uL.amx	**Type:**	Sample
**Processing method:**	GC_LC area percent_DefaultMethod.pmx	**Sample amount:**	0.00
**Manually modified:**	None		

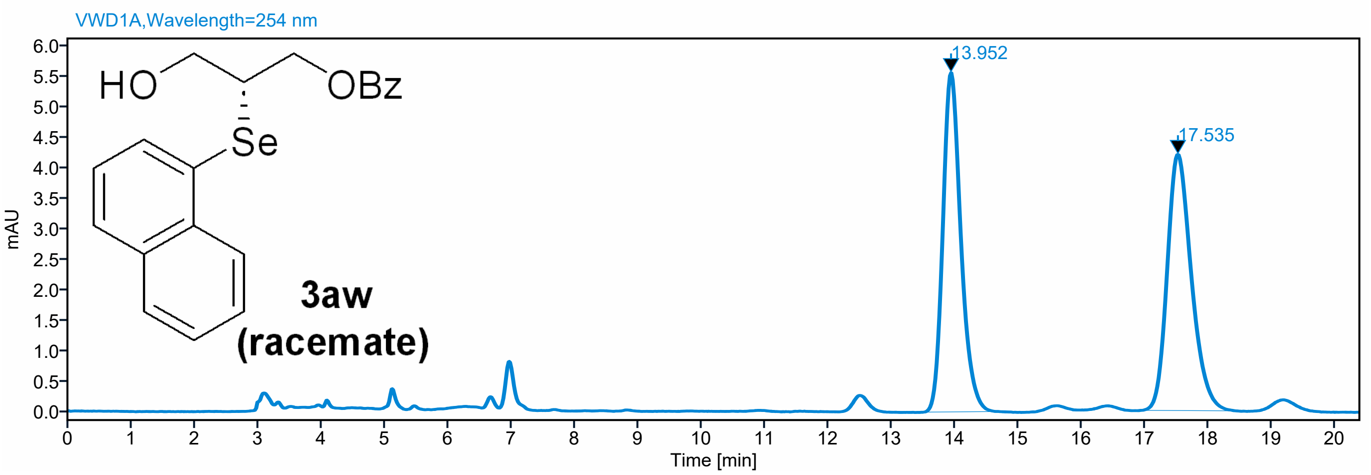

Signal: VWD1A,Wavelength=254 nm

RT [min]	Type	Width [min]	Area	Height	Area%
13.952	BB	1.14	109.86	5.56	49.65
17.535	BB	1.44	111.40	4.20	50.35
		**Sum**	**221.26**		

Single Injection Report

Agilent

**Data file:**	CJX-2-105-ASY-IB-1.0-10%		
**Sequence Name:**	SingleSample	**Project Name:**	CJX
**Sample name:**	CJX-2-105-ASY-IB-1.0-10%	**Operator:**	SYSTEM (SYSTEM)
**Instrument:**	1260	**Injection date:**	2025-06-19 11:59:40+08:00
**Inj. volume:**	5.000 µL	**Location:**	P1-C6
**Acq. method:**	10%-60min-1.0ml-5uL.amx	**Type:**	Sample
**Processing method:**	GC_LC area percent_DefaultMethod.pmx	**Sample amount:**	0.00
**Manually modified:**	Manual Integration		

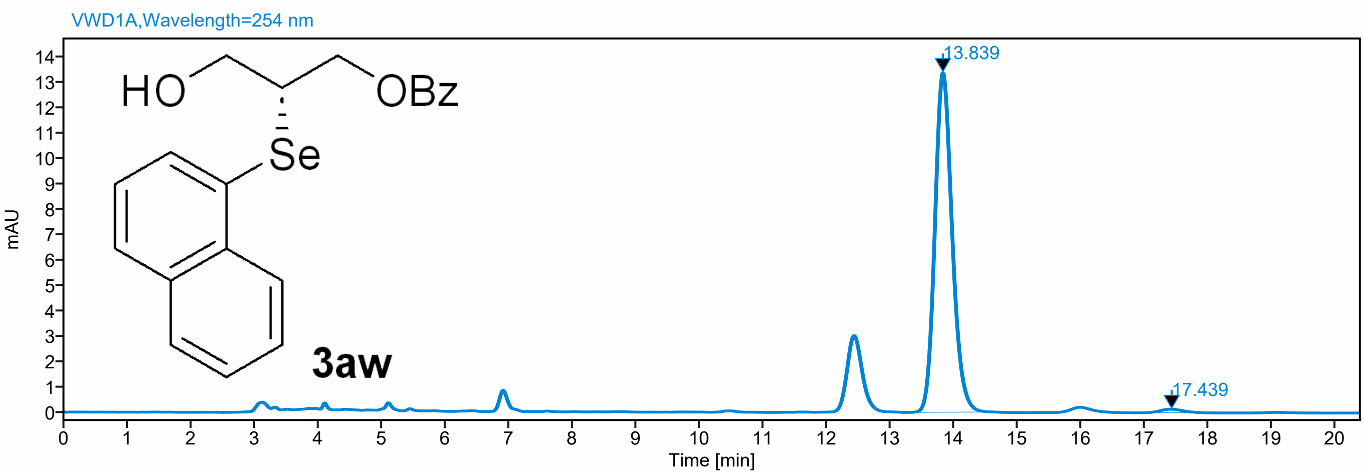

Signal: VWD1A,Wavelength=254 nm

RT [min]	Type	Width [min]	Area	Height	Area%
13.839	BB	1.21	252.84	13.37	98.59
17.439	MM m	1.11	3.61	0.14	1.41
		**Sum**	**256.45**		

Single Injection Report

**Data file:**	CJX-3-1-RAC-IB-1.0-10%		
**Sequence Name:**	SingleSample	**Project Name:**	CJX
**Sample name:**	CJX-3-1-RAC-IB-1.0-10%	**Operator:**	SYSTEM (SYSTEM)
**Instrument:**	1260	**Injection date:**	2025-06-11 09:56:02+08:00
**Inj. volume:**	5.000 µL	**Location:**	P2-D1
**Acq. method:**	10%-60min-1.0ml-5uL.amx	**Type:**	Sample
**Processing method:**	GC_LC area percent_DefaultMethod.pmx	**Sample amount:**	0.00
**Manually modified:**	None		

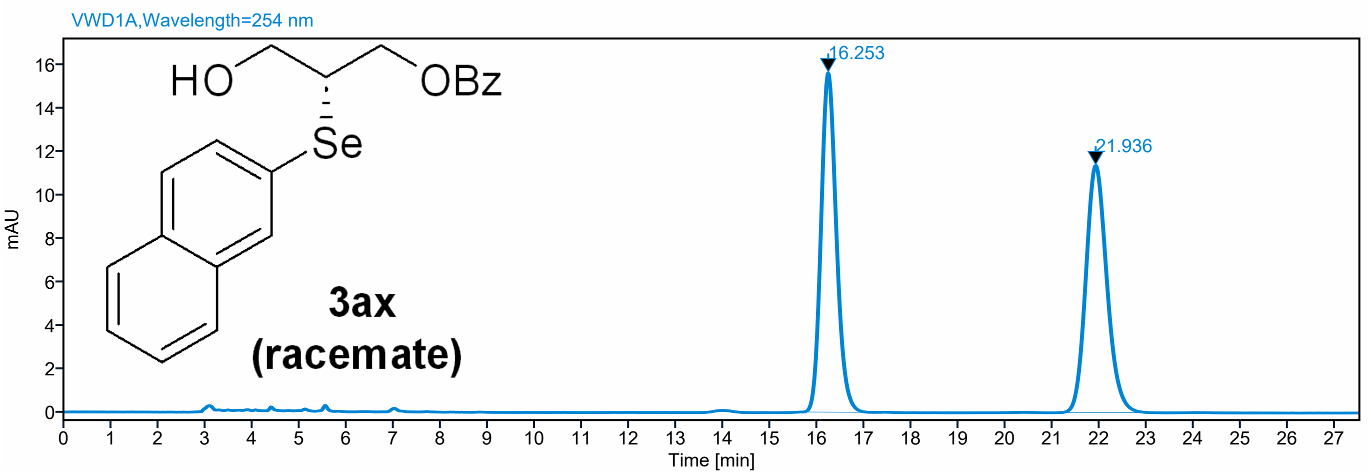

Signal: VWD1A,Wavelength=254 nm

RT [min]	Type	Width [min]	Area	Height	Area%
16.253	BB	1.41	354.66	15.63	50.06
21.936	BB	1.80	353.76	11.38	49.94
		**Sum**	**708.42**		

Single Injection Report

**Data file:**	CJX-3-1-ASY-IB-1.0-10%		
**Sequence Name:**	SingleSample	**Project Name:**	CJX
**Sample name:**	CJX-3-1-ASY-IB-1.0-10%	**Operator:**	SYSTEM (SYSTEM)
**Instrument:**	1260	**Injection date:**	2025-06-11 10:26:41+08:00
**Inj. volume:**	5.000 µL	**Location:**	P2-D2
**Acq. method:**	10%-60min-1.0ml-5uL.amx	**Type:**	Sample
**Processing method:**	GC_LC area percent_DefaultMethod.pmx	**Sample amount:**	0.00
**Manually modified:**	Manual Integration		

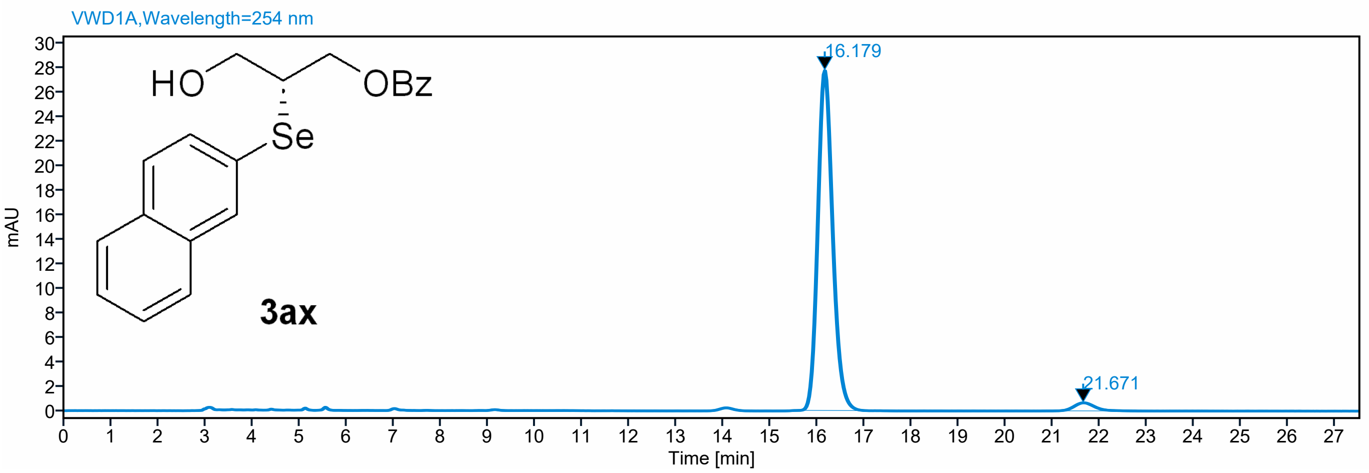

Signal: VWD1A,Wavelength=254 nm

RT [min]	Type	Width [min]	Area	Height	Area%
16.179	BB	1.36	610.74	27.72	96.84
21.671	MM m	1.88	19.93	0.66	3.16
		**Sum**	**630.67**		

Single Injection Report

Agilent

**Data file:**	CJX-3-131-ASY-IB-1.0-5%		
**Sequence Name:**	SingleSample	**Project Name:**	CJX
**Sample name:**	CJX-3-131-ASY-IB-1.0-5%	**Operator:**	SYSTEM (SYSTEM)
**Instrument:**	1260	**Injection date:**	2025-02-25 14:57:46+08:00
**Inj. volume:**	5.000 µL	**Location:**	P1-F1
**Acq. method:**	10%-60min-0.8ml-5uL.amx	**Type:**	Sample
**Processing method:**	GC_LC area percent_DefaultMethod.pmx	**Sample amount:**	0.00
**Manually modified:**	None		

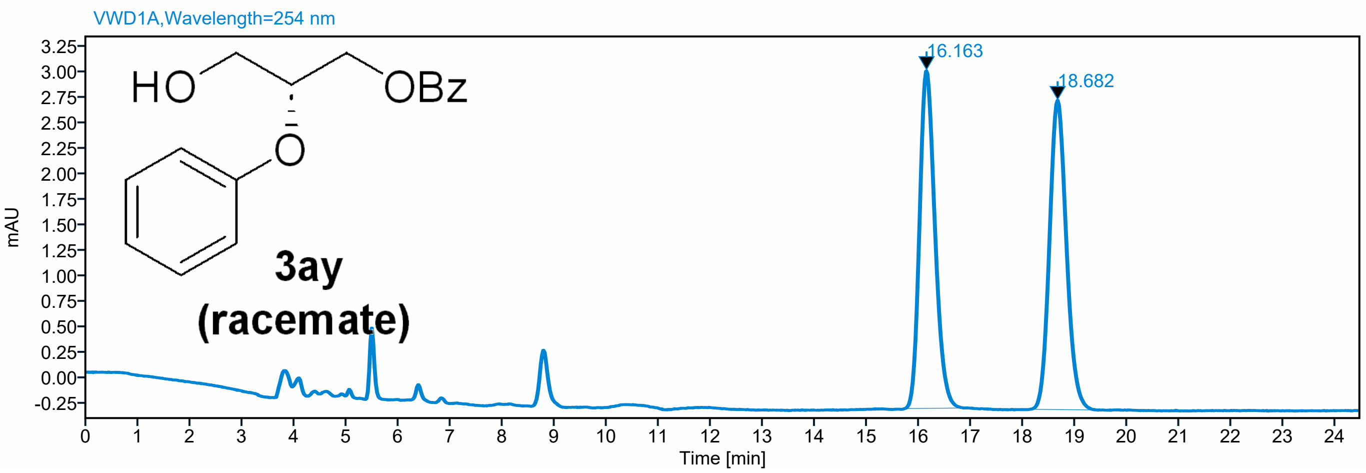

Signal: VWD1A,Wavelength=254 nm

RT [min]	Type	Width [min]	Area	Height	Area%
16.163	BB	1.12	68.21	3.31	49.86
18.682	BB	1.22	68.61	3.03	50.14
		**Sum**	**136.82**		

Single Injection Report

Agilent

**Data file:**	CJX-3-131-ASY-IB-1.0-5%		
**Sequence Name:**	SingleSample	**Project Name:**	CJX
**Sample name:**	CJX-3-131-ASY-IB-1.0-5%	**Operator:**	SYSTEM (SYSTEM)
**Instrument:**	1260	**Injection date:**	2025-02-25 15:31:48+08:00
**Inj. volume:**	5.000 µL	**Location:**	P1-F2
**Acq. method:**	10%-60min-0.8ml-5uL.amx	**Type:**	Sample
**Processing method:**	GC_LC area percent_DefaultMethod.pmx	**Sample amount:**	0.00
**Manually modified:**	Manual Integration		

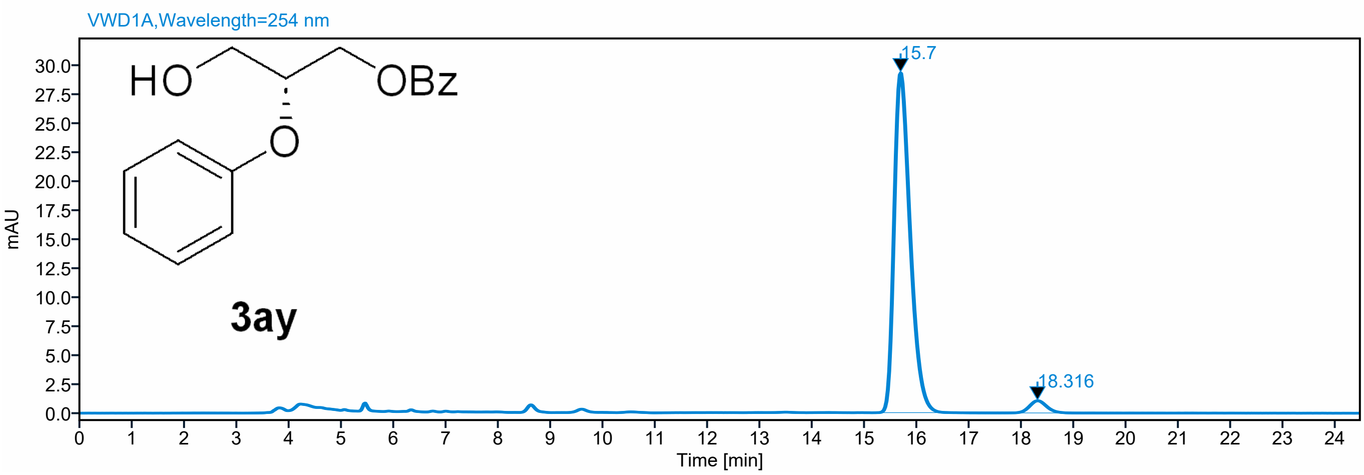

Signal: VWD1A,Wavelength=254 nm

RT [min]	Type	Width [min]	Area	Height	Area%
15.700	BB	1.36	624.48	29.32	96.30
18.316	MM m	1.37	24.02	1.05	3.70
		**Sum**	**648.50**		

Single Injection Report

Agilent

**Data file:**	CJX-3-131-ASY-IB-1.0-5%		
**Sequence Name:**	SingleSample	**Project Name:**	CJX
**Sample name:**	CJX-3-131-ASY-IB-1.0-5%	**Operator:**	SYSTEM (SYSTEM)
**Instrument:**	1260	**Injection date:**	2025-04-23 15:30:08+08:00
**Inj. volume:**	5.000 µL	**Location:**	P2-A1
**Acq. method:**	10%-60min-1.0ml-5uL.amx	**Type:**	Sample
**Processing method:**	GC_LC area percent_DefaultMethod.pmx	**Sample amount:**	0.00
**Manually modified:**	Manual Integration		

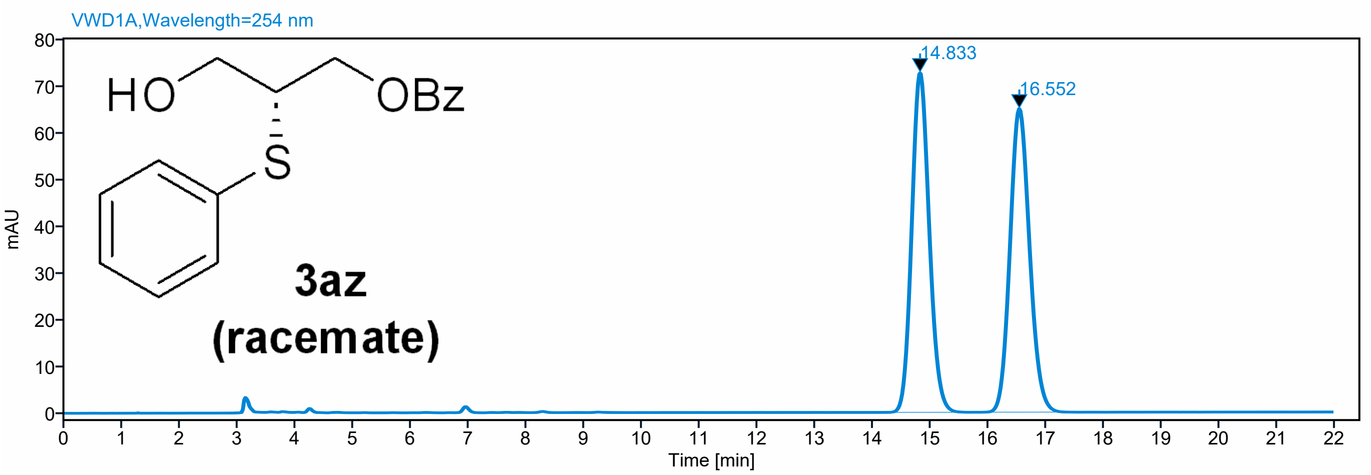

Signal: VWD1A,Wavelength=254 nm

RT [min]	Type	Width [min]	Area	Height	Area%
14.833	BB	1.37	1531.11	72.73	49.96
16.552	BB	1.41	1533.47	64.98	50.04
		**Sum**	**3064.59**		

Single Injection Report

Agilent

**Data file:**	CJX-3-131-ASY-IB-1.0-5%		
**Sequence Name:**	SingleSample	**Project Name:**	CJX
**Sample name:**	CJX-3-131-ASY-IB-1.0-5%	**Operator:**	SYSTEM (SYSTEM)
**Instrument:**	1260	**Injection date:**	2025-04-23 15:52:48+08:00
**Inj. volume:**	5.000 µL	**Location:**	P2-A2
**Acq. method:**	10%-60min-1.0ml-5uL.amx	**Type:**	Sample
**Processing method:**	GC_LC area percent_DefaultMethod.pmx	**Sample amount:**	0.00
**Manually modified:**	Manual Integration		

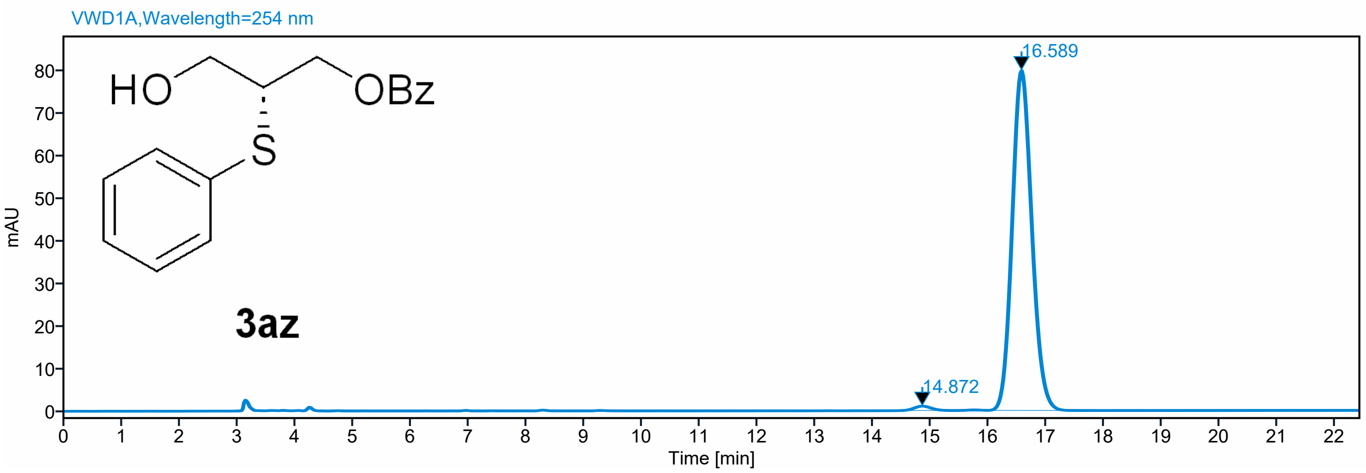

Signal: VWD1A,Wavelength=254 nm

RT [min]	Type	Width [min]	Area	Height	Area%
14.872	MM m	1.43	19.76	1.03	1.04
16.589	BB	1.47	1887.70	79.74	98.96
		**Sum**	**1907.45**		

Single Injection Report

Agilent

**Data file:**	CJX-3-131-ASY-IB-1.0-5%		
**Sequence Name:**	SingleSample	**Project Name:**	CJX
**Sample name:**	CJX-3-131-ASY-IB-1.0-5%	**Operator:**	SYSTEM (SYSTEM)
**Instrument:**	1260	**Injection date:**	2025-02-26 09:02:34+08:00
**Inj. volume:**	5.000 µL	**Location:**	P1-F3
**Acq. method:**	30%-60min-0.8ml-5uL.amx	**Type:**	Sample
**Processing method:**	GC_LC area percent_DefaultMethod.pmx	**Sample amount:**	0.00
**Manually modified:**	None		

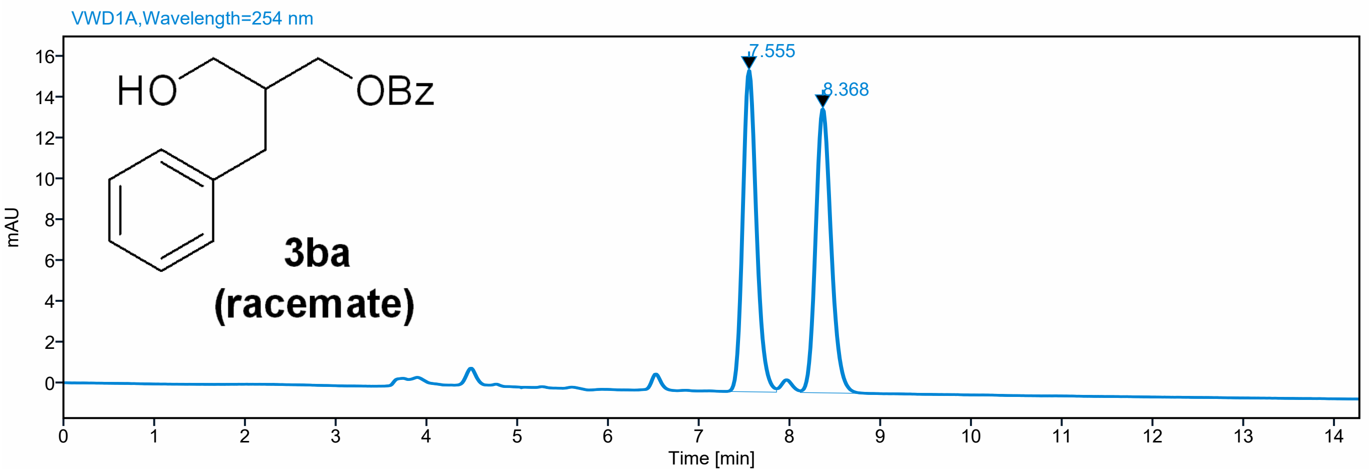

Signal: VWD1A,Wavelength=254 nm

RT [min]	Type	Width [min]	Area	Height	Area%
7.555	BV	0.56	165.44	15.74	49.93
8.368	VB	0.78	165.90	13.91	50.07
		**Sum**	**331.34**		

Single Injection Report

**Data file:**	CJX-3-131-ASY-IB-1.0-5%		
**Sequence Name:**	SingleSample	**Project Name:**	CJX
**Sample name:**	CJX-3-131-ASY-IB-1.0-5%	**Operator:**	SYSTEM (SYSTEM)
**Instrument:**	1260	**Injection date:**	2025-02-26 16:38:41+08:00
**Inj. volume:**	5.000 µL	**Location:**	P1-F4
**Acq. method:**	30%-60min-0.8ml-5uL.amx	**Type:**	Sample
**Processing method:**	GC_LC area percent_DefaultMethod.pmx	**Sample amount:**	0.00
**Manually modified:**	Manual Integration		

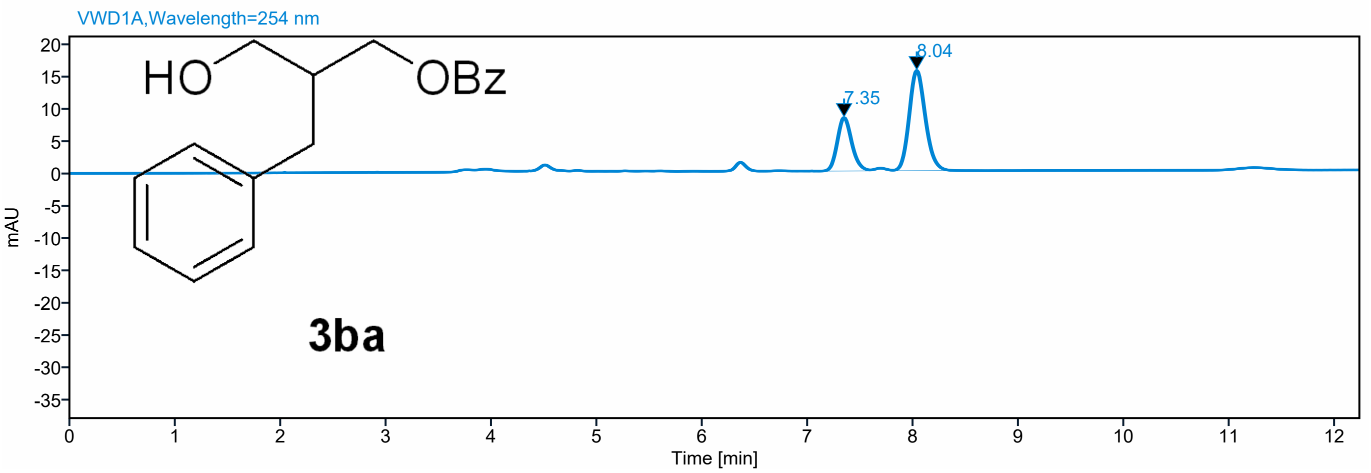

Signal: VWD1A,Wavelength=254 nm

RT [min]	Type	Width [min]	Area	Height	Area%
7.350	BV	0.49	79.20	8.27	32.23
8.040	VB	0.66	166.53	15.52	67.77
		**Sum**	**245.73**		

Single Injection Report

**Data file:**	CJX-3-70-RAC-AS-H-0.5-1%		
**Sequence Name:**	SingleSample	**Project Name:**	CJX
**Sample name:**	CJX-3-70-RAC-AS-H-0.5-1%	**Operator:**	SYSTEM (SYSTEM)
**Instrument:**	1260	**Injection date:**	2025-08-24 16:12:18+08:00
**Inj. volume:**	5.000 µL	**Location:**	P2-E1
**Acq. method:**	1%-50min-0.5ml-5uL.amx	**Type:**	Sample
**Processing method:**	GC_LC area percent_DefaultMethod.pmx	**Sample amount:**	0.00
**Manually modified:**	None		

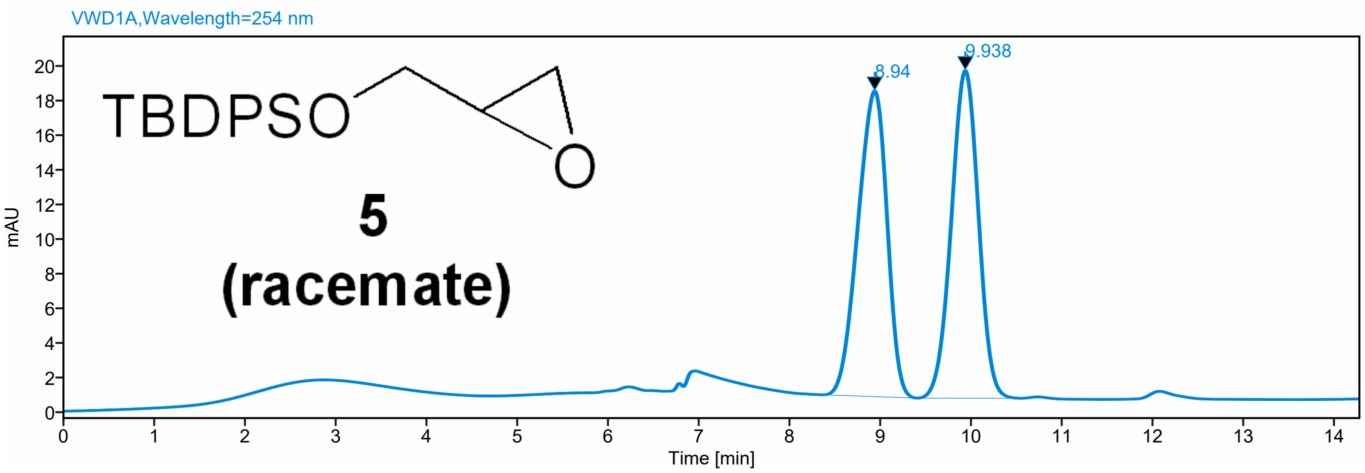

Signal: VWD1A,Wavelength=254 nm

RT [min]	Type	Width [min]	Area	Height	Area%
8.940	BB	1.03	394.15	17.66	50.05
9.938	BB	1.13	393.31	18.93	49.95
		**Sum**	**787.47**		

Single Injection Report

**Data file:**	CJX-3-90-ASY-AS-H-0.5-1%		
**Sequence Name:**	SingleSample	**Project Name:**	CJX
**Sample name:**	CJX-3-90-ASY-AS-H-0.5-1%	**Operator:**	SYSTEM (SYSTEM)
**Instrument:**	1260	**Injection date:**	2025-08-25 19:54:07+08:00
**Inj. volume:**	5.000 µL	**Location:**	P2-E2
**Acq. method:**	1%-50min-0.5ml-5uL.amx	**Type:**	Sample
**Processing method:**	GC_LC area percent_DefaultMethod.pmx	**Sample amount:**	0.00
**Manually modified:**	Manual Integration		

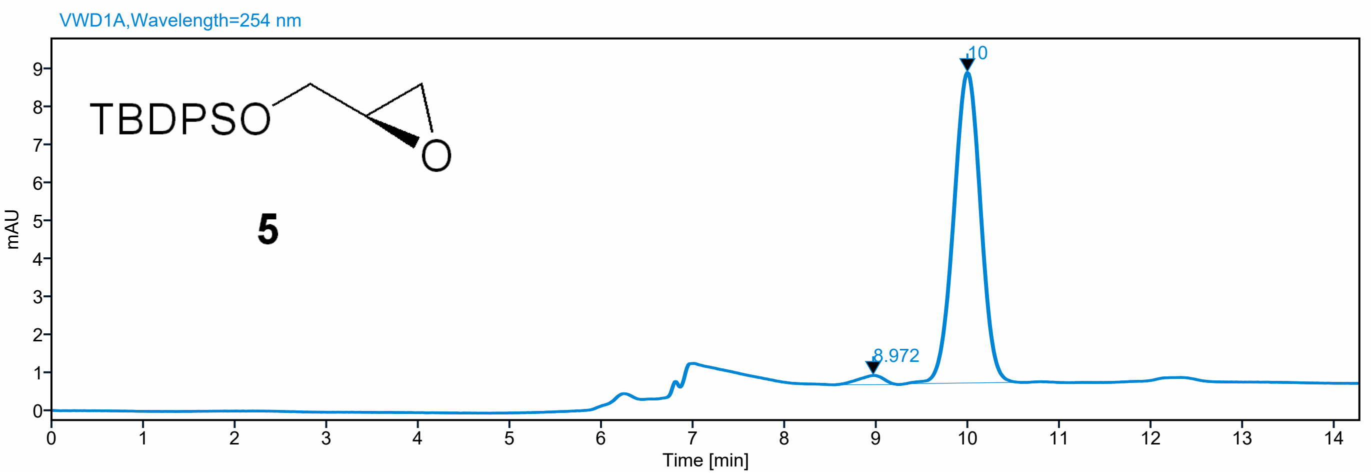

Signal: VWD1A,Wavelength=254 nm

RT [min]	Type	Width [min]	Area	Height	Area%
8.972	MM m	0.72	4.70	0.24	2.74
10.000	MM m	1.37	167.15	8.17	97.26
		**Sum**	**171.86**		

Single Injection Report

**Data file:**	HQQ-12C-ASY		
**Sequence Name:**	SingleSample	**Project Name:**	HQQ
**Sample name:**	hqq-s3-115-ib-2%-1ml20251017 215530	**Operator:**	SYSTEM (SYSTEM)
**Instrument:**	1260	**Injection date:**	2025-10-17 22:10:18+08:00
**Inj. volume:**	5.000 µL	**Location:**	P2-F1
**Acq. method:**	2%-20min-1.0ml-5ul.amx	**Type:**	Sample
**Processing method:**	GC_LC area percent_DefaultMethod.pmx	**Sample amount:**	0.00
**Manually modified:**	None		

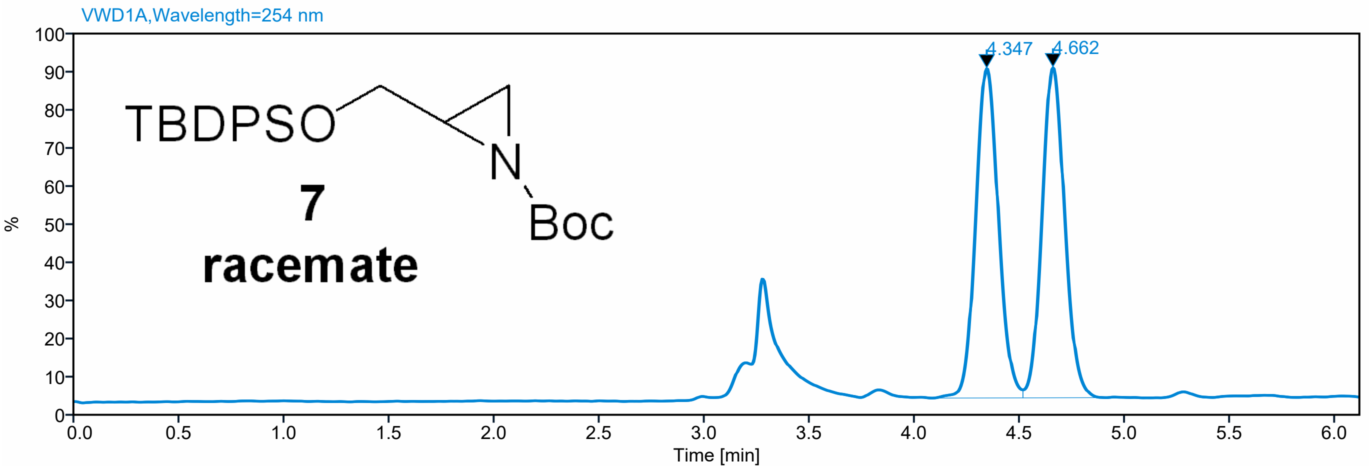

Signal: VWD1A,Wavelength=254 nm

RT [min]	Type	Width [min]	Area	Height	Area%
4.347	BV	0.43	14.36	1.89	50.11
4.662	VB	0.39	14.30	1.90	49.89
		**Sum**	**28.66**		

Single Injection Report

Agilent

**Data file:**	HQQ-12C-ASY		
**Sequence Name:**	SingleSample	**Project Name:**	HQQ
**Sample name:**	hqq-s3-115-asy-ib-2%-1ml20251018 211243	**Operator:**	SYSTEM (SYSTEM)
**Instrument:**	1260	**Injection date:**	2025-10-18 21:20:54+08:00
**Inj. volume:**	5.000 µL	**Location:**	P2-F1
**Acq. method:**	2%-20min-1.0ml-5ul.amx	**Type:**	Sample
**Processing method:**	GC_LC area percent_DefaultMethod.pmx	**Sample amount:**	0.00
**Manually modified:**	Manual Integration		

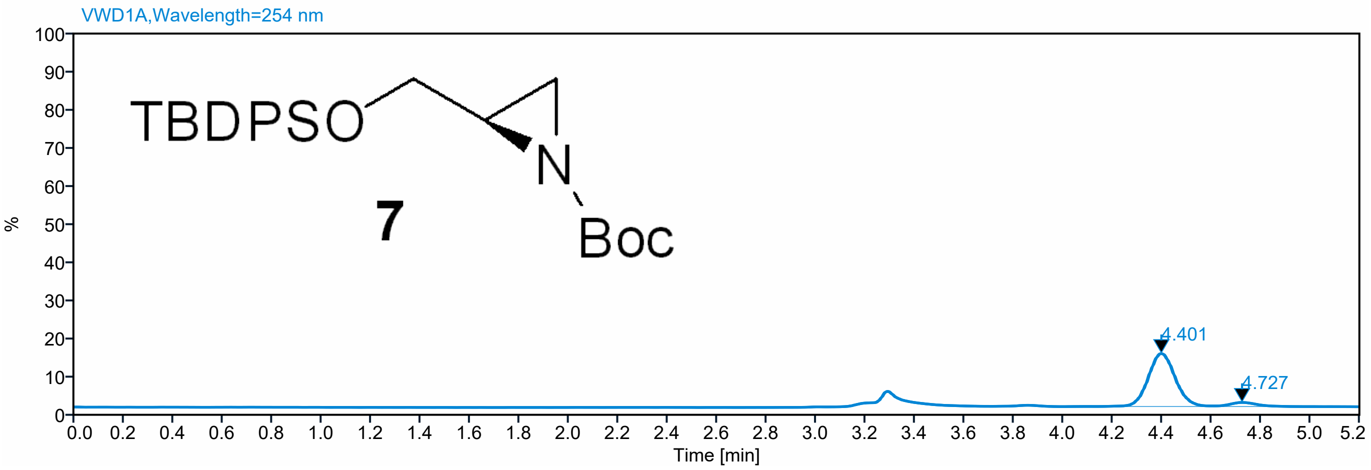

Signal: VWD1A,Wavelength=254 nm

RT [min]	Type	Width [min]	Area	Height	Area%
4.401	BM m	0.45	19.11	2.51	92.70
4.727	MM m	0.33	1.51	0.19	7.30
		**Sum**	**20.62**		

Single Injection Report

Agilent

**Data file:**	HQQ-12C-ASY		
**Sequence Name:**	SingleSample	**Project Name:**	HQQ
**Sample name:**	HQQ-s3-95-rac-ad-h-2%-1ML-20251001 165457	**Operator:**	SYSTEM (SYSTEM)
**Instrument:**	1260	**Injection date:**	2025-10-01 16:55:39+08:00
**Inj. volume:**	2.000 µL	**Location:**	P2-F1
**Acq. method:**	2%-30min-1.0ml-2ul.amx	**Type:**	Sample
**Processing method:**	GC_LC area percent_DefaultMethod.pmx	**Sample amount:**	0.00
**Manually modified:**	None		

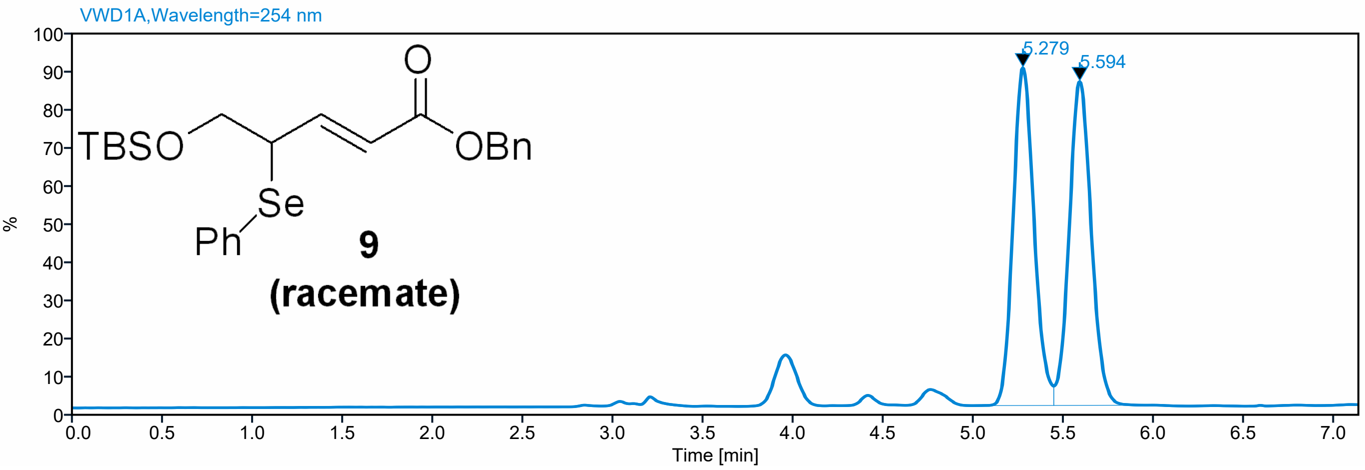

Signal: VWD1A,Wavelength=254 nm

RT [min]	Type	Width [min]	Area	Height	Area%
5.279	BV	0.45	69.68	8.57	49.86
5.594	VB	0.52	70.07	8.22	50.14
		**Sum**	**139.75**		

Single Injection Report

**Data file:**	HQQ-12C-ASY		
**Sequence Name:**	SingleSample	**Project Name:**	HQQ
**Sample name:**	hqq-s3-3-100-AD-H-2%-1ml-20251003 145812	**Operator:**	SYSTEM (SYSTEM)
**Instrument:**	1260	**Injection date:**	2025-10-03 14:58:56+08:00
**Inj. volume:**	2.000 µL	**Location:**	P2-F1
**Acq. method:**	2%-30min-1.0ml-2ul.amx	**Type:**	Sample
**Processing method:**	GC_LC area percent_DefaultMethod.pmx	**Sample amount:**	0.00
**Manually modified:**	Manual Integration		

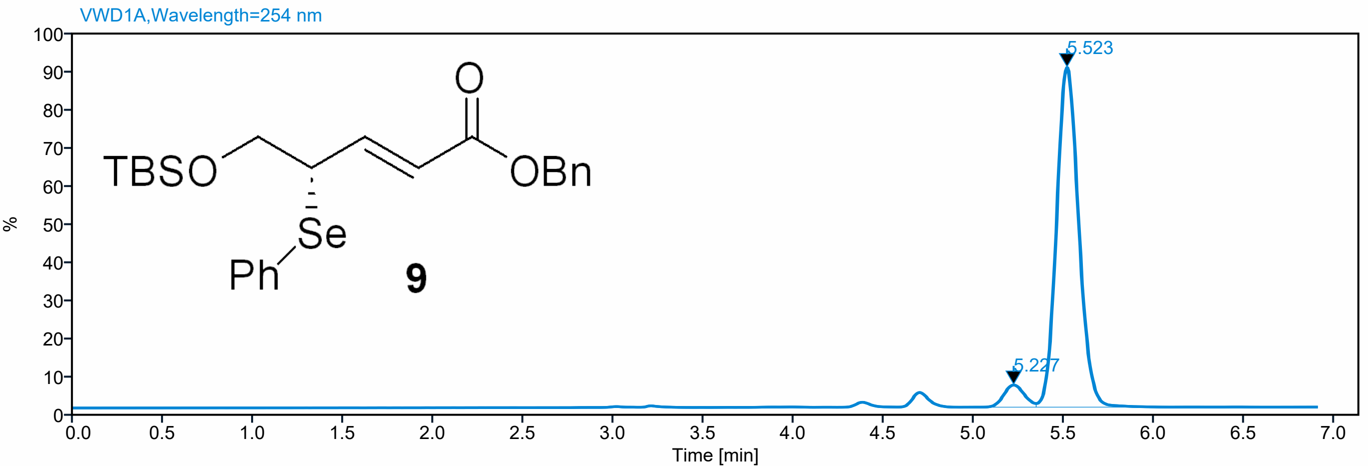

Signal: VWD1A,Wavelength=254 nm

RT [min]	Type	Width [min]	Area	Height	Area%
5.227	BV	0.42	23.79	2.98	5.74
5.523	VB	0.75	390.81	45.59	94.26
		**Sum**	**414.60**		

Single Injection Report

**Data file:**	HQQ-12C-ASY		
**Sequence Name:**	SingleSample	**Project Name:**	HQQ
**Sample name:**	HQQ-S3-114-RAC-ADH-10%-1ML-210NM020251015 200449	**Operator:**	SYSTEM (SYSTEM)
**Instrument:**	1260	**Injection date:**	2025-10-15 20:05:38+08:00
**Inj. volume:**	5.000 µL	**Location:**	P2-F1
**Acq. method:**	10%-15min-1.0ml-5UL-210nm.amx	**Type:**	Sample
**Processing method:**	GC_LC area percent_DefaultMethod.pmx	**Sample amount:**	0.00
**Manually modified:**	Manual Integration		

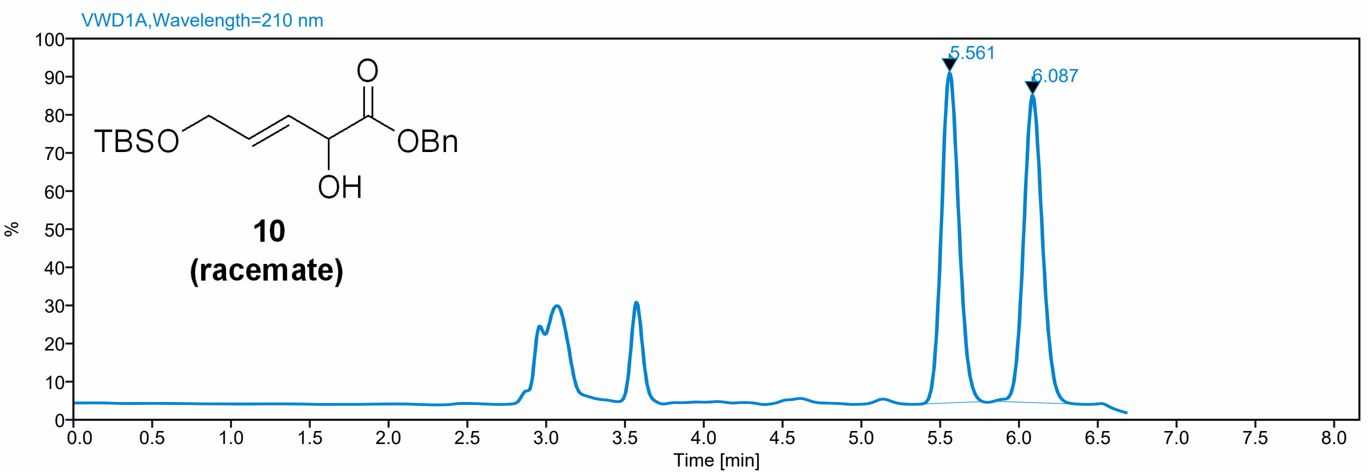

10
(racemate)

Signal: VWD1A,Wavelength=210 nm

RT [min]	Type	Width [min]	Area	Height	Area%
5.561	MM m	0.39	386.67	50.08	50.05
6.087	MM m	0.55	385.94	46.55	49.95
		**Sum**	**772.61**		

Single Injection Report

Agilent

**Data file:**	HQQ-12C-ASY		
**Sequence Name:**	SingleSample	**Project Name:**	HQQ
**Sample name:**	HQQ-S3-114-ADH-10%-1ML-210NM020251016 112416-REre	**Operator:**	SYSTEM (SYSTEM)
**Instrument:**	1260	**Injection date:**	2025-10-16 11:24:59+08:00
**Inj. volume:**	5.000 µL	**Location:**	P2-F1
**Acq. method:**	10%-15min-1.0ml-5UL-210nm.amx	**Type:**	Sample
**Processing method:**	GC_LC area percent_DefaultMethod.pmx	**Sample amount:**	0.00
**Manually modified:**	Manual Integration		

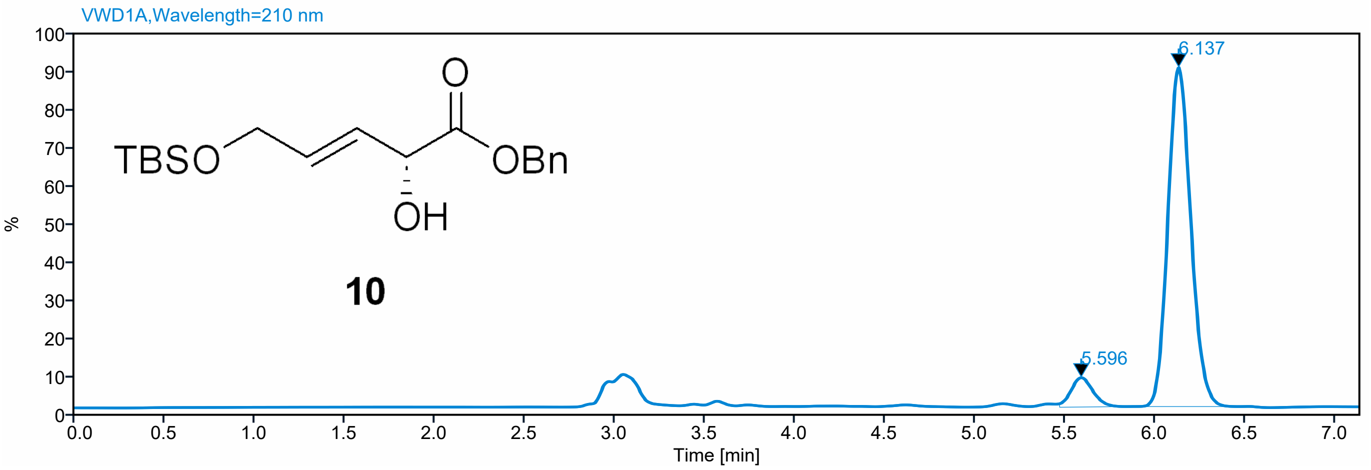

Signal: VWD1A,Wavelength=210 nm

RT [min]	Type	Width [min]	Area	Height	Area%
5.596	MB m	0.37	134.08	15.70	7.71
6.137	BB	0.63	1605.29	180.94	92.29
		**Sum**	**1739.38**		

Single Injection Report

Agilent

**Data file:**	CJX-3-111-RAC-IB-1.0-5%		
**Sequence Name:**	SingleSample	**Project Name:**	CJX
**Sample name:**	CJX-3-111-RAC-IB-1.0-5%	**Operator:**	SYSTEM (SYSTEM)
**Instrument:**	1260	**Injection date:**	2025-09-05 11:33:24+08:00
**Inj. volume:**	5.000 µL	**Location:**	P1-A1
**Acq. method:**	5%-50min-1ml.amx	**Type:**	Sample
**Processing method:**	GC_LC area percent_DefaultMethod.pmx	**Sample amount:**	0.00
**Manually modified:**	Manual Integration		

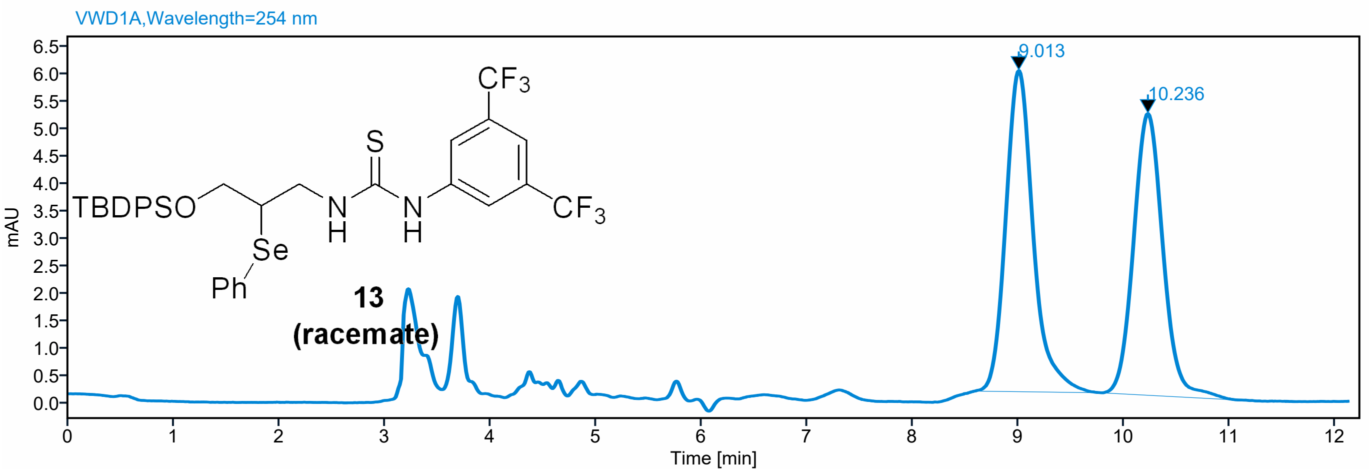

Signal: VWD1A,Wavelength=254 nm

RT [min]	Type	Width [min]	Area	Height	Area%
9.013	BB	1.19	106.02	5.85	51.58
10.236	BB	1.28	99.53	5.13	48.42
		**Sum**	**205.55**		

Single Injection Report

Agilent

**Data file:**	CJX-3-125-ASY-IB-1.0-5%		
**Sequence Name:**	SingleSample	**Project Name:**	CJX
**Sample name:**	CJX-3-125-ASY-IB-1.0-5%	**Operator:**	SYSTEM (SYSTEM)
**Instrument:**	1260	**Injection date:**	2025-09-05 17:00:43+08:00
**Inj. volume:**	5.000 µL	**Location:**	P1-A1
**Acq. method:**	5%-50min-1ml.amx	**Type:**	Sample
**Processing method:**	GC_LC area percent_DefaultMethod.pmx	**Sample amount:**	0.00
**Manually modified:**	None		

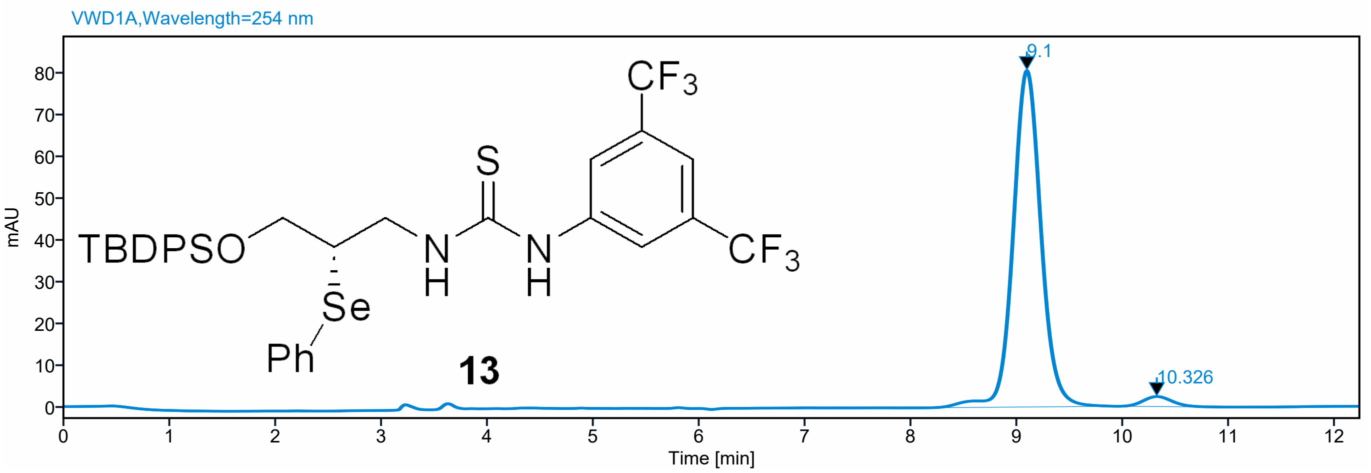

Signal: VWD1A,Wavelength=254 nm

RT [min]	Type	Width [min]	Area	Height	Area%
9.100	BB	1.77	1479.18	80.53	96.99
10.326	BB	0.99	45.98	2.39	3.01
		**Sum**	**1525.16**		

Single Injection Report

Agilent

**Data file:**	CJX-3-129-RAC-IB-1.0-5%		
**Sequence Name:**	SingleSample	**Project Name:**	CJX
**Sample name:**	CJX-3-129-RAC-IB-1.0-5%	**Operator:**	SYSTEM (SYSTEM)
**Instrument:**	1260	**Injection date:**	2025-09-06 15:19:38+08:00
**Inj. volume:**	5.000 µL	**Location:**	P1-A1
**Acq. method:**	5%-50min-1ml.amx	**Type:**	Sample
**Processing method:**	GC_LC area percent_DefaultMethod.pmx	**Sample amount:**	0.00
**Manually modified:**	Manual Integration		

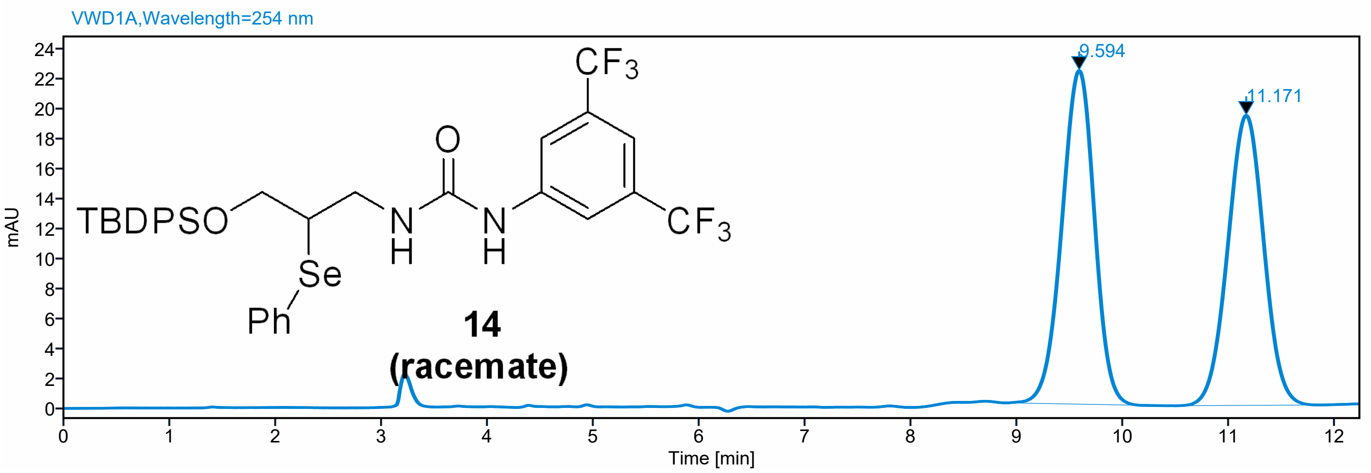

Signal: VWD1A,Wavelength=254 nm

RT [min]	Type	Width [min]	Area	Height	Area%
9.594	MM m	1.28	477.53	22.25	51.03
11.171	BB	1.33	458.28	19.34	48.97
		**Sum**	**935.81**		

Single Injection Report

Agilent

**Data file:**	CJX-3-131-ASY-IB-1.0-5%		
**Sequence Name:**	SingleSample	**Project Name:**	CJX
**Sample name:**	CJX-3-131-ASY-IB-1.0-5%	**Operator:**	SYSTEM (SYSTEM)
**Instrument:**	1260	**Injection date:**	2025-09-06 23:16:28+08:00
**Inj. volume:**	5.000 µL	**Location:**	P1-A2
**Acq. method:**	5%-50min-1ml.amx	**Type:**	Sample
**Processing method:**	GC_LC area percent_DefaultMethod.pmx	**Sample amount:**	0.00
**Manually modified:**	Manual Integration		

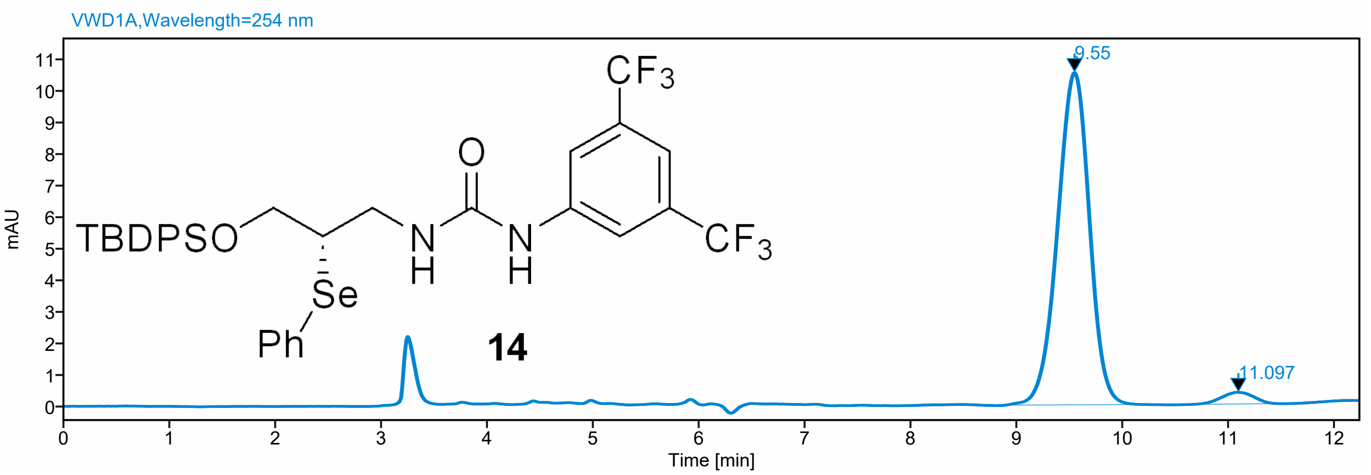

Signal: VWD1A,Wavelength=254 nm

RT [min]	Type	Width [min]	Area	Height	Area%
9.550	BB	1.30	220.29	10.53	96.56
11.097	MM m	0.93	7.86	0.37	3.44
		**Sum**	**228.15**		

Single Injection Report

Agilent

**Data file:**	HQQ-12C-ASY		
**Sequence Name:**	SingleSample	**Project Name:**	HQQ
**Sample name:**	hqq-s3-125-RAC-AD-H-30-%-1ML-254NM-20251105 210906	**Operator:**	SYSTEM (SYSTEM)
**Instrument:**	1260	**Injection date:**	2025-11-05 21:19:46+08:00
**Inj. volume:**	5.000 µL	**Location:**	P2-F5
**Acq. method:**	30%-60min-1.0ml-5ul-210.amx	**Type:**	Sample
**Processing method:**	GC_LC area percent_DefaultMethod.pmx	**Sample amount:**	0.00
**Manually modified:**	Manual Integration		

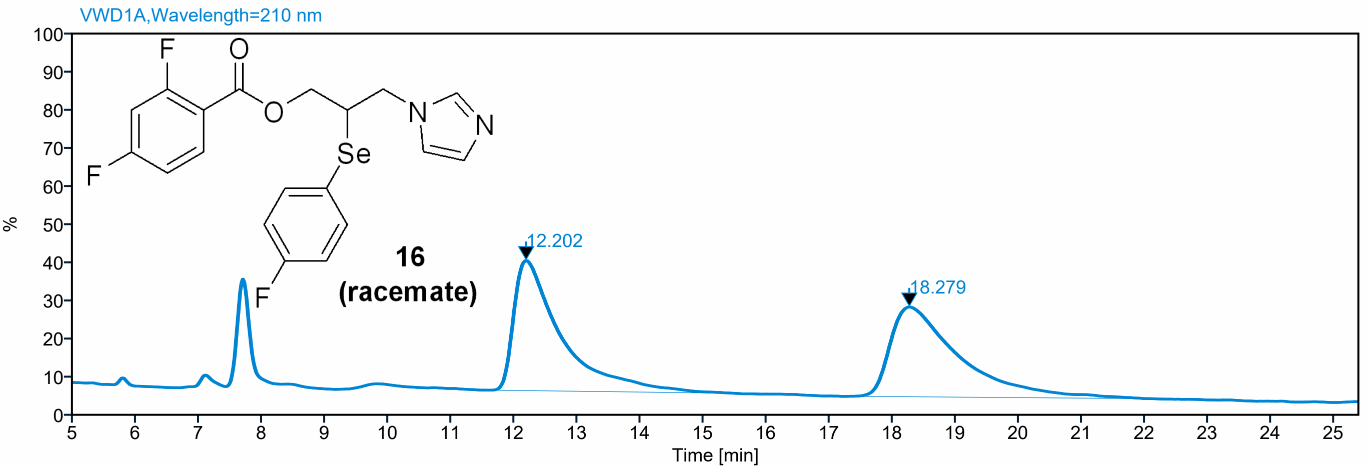

Signal: VWD1A,Wavelength=210 nm

RT [min]	Type	Width [min]	Area	Height	Area%
12.202	BM m	3.84	274.08	5.23	50.06
18.279	BM m	4.66	273.46	3.61	49.94
		**Sum**	**547.54**		

**Data file:**	HQQ-12C-ASY		
**Sequence Name:**	SingleSample	**Project Name:**	HQQ
**Sample name:**	hqq-s3-135-asy-AD-H-30-%-1ML-254NM-20251105 215632	**Operator:**	SYSTEM (SYSTEM)
**Instrument:**	1260	**Injection date:**	2025-11-05 21:57:17+08:00
**Inj. volume:**	5.000 µL	**Location:**	P2-F6
**Acq. method:**	30%-60min-1.0ml-5ul-210.amx	**Type:**	Sample
**Processing method:**	GC_LC area percent_DefaultMethod.pmx	**Sample amount:**	0.00
**Manually modified:**	Manual Integration		

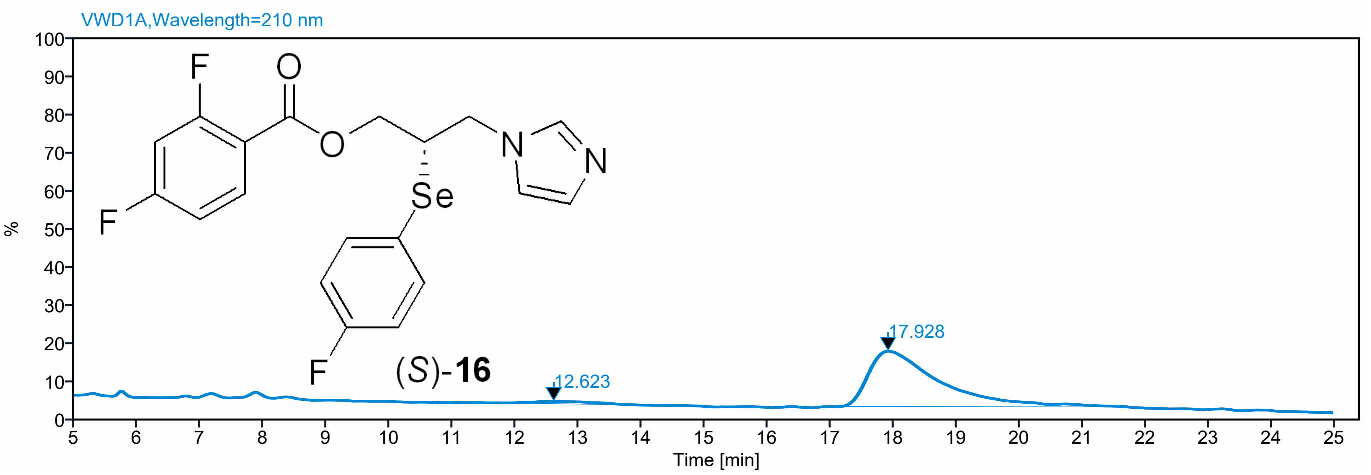

Signal: VWD1A,Wavelength=210 nm

RT [min]	Type	Width [min]	Area	Height	Area%
12.623	MM m	3.05	6.18	0.08	3.95
17.928	BM m	4.32	150.21	1.95	96.05
		**Sum**	**156.39**		
